# Supplementary material for: Rhodium-catalyzed selective direct arylation of phosphines with aryl bromides
Source: Nat Commun. 2022 May 25;13:2934. doi: 10.1038/s41467-022-30697-7 (PMC9132997; doi:10.1038/s41467-022-30697-7)
Supplement: Supplementary file 1 — Supplementary Information [file 41467_2022_30697_MOESM1_ESM.pdf]

Supplementary Information for

Rhodium-Catalysed Selective Direct Arylation of Phosphines with Aryl Bromides

Dingyi Wang[1,†], Mingjie Li[1,†], Chengdong Shuang[2]*, Yong Liang[1], Yue Zhao[1], Minyan Wang[1]*, and Zhuangzhi Shi[1,3]*

[1]State Key Laboratory of Coordination Chemistry, Chemistry and Biomedicine Innovation Center (ChemBIC), School of Chemistry and Chemical Engineering, Nanjing University, Nanjing 210093, China

[2]State Key Laboratory of Pollution Control and Resources Reuse, School of the Environment, Nanjing University, Nanjing 210093, China

[3]School of Chemistry and Chemical Engineering, Henan Normal University, Xinxiang, Henan 453007, China

[†]D.W and M.L contributed equally to this work.

*Corresponding authors. E-mail: shuangchendong@nju.edu.cn; wangmy@nju.edu.cn; shiz@nju.edu.cn

Table of Contents

1 Supplementary Methods 3

1.1 General Information 3

2 Supplementary Discussion..4

2.1 General Procedure for Direct Arylation of Arylphosphines with Arylbromides through One-Fold C–H Activation...4

2.2 General Procedure for Arylation of Arylphosphines with Arylbromides through Two-Fold C–H Activation 13

2.3 General Procedure for Arylation of Arylphosphines with Arylbromides through Three-Fold C–H Activation 16

2.4 Mechanistic Experiments 21

2.5 Computational Details...22

2.6 NMR spectroscopic data 34

3 Supplementary References...78

1 Supplementary Methods

1.1 General Information

All new compounds were fully characterized. Compounds were visualized by exposure to UV-light. All reactions and manipulations involving air- or moisture-sensitive compounds were performed using standard Schlenk techniques or in a glovebox. Toluene was purified using Pure Solv MD-5 solvent purification system, from Innovative Technology, Inc., by passing the solvent through two activated alumina columns after purging with argon. ^{1}H, ^{13}C, ^{31}P and ^{19}F NMR spectra were recorded on a Bruker AVANCE III 400 MHz or 500 MHz spectrometer. Chemical shifts (δ values) were reported in ppm with $CDCl_3$ (7.26 and 77.16 ppm for ^{1}H and ^{13}C respectively). Mass spectra were conducted at Agilent 6540 Ultra-High-Definition (UHD) Accurate-Mass Quadrupole Time-of-Flight (Q-TOF) liquid chromatography/mass spectrometry (LC/MS) system andThermo Scientific TRACE 1300 ISQ LT gas chromatography/mass spectrometry (GC/MS) system. IR spectra were recorded on a Bruker FT-IR spectrometer. Chiral HPLC analyses were performed on a Thermo Scientific UltiMate 3000 liquid chromatography system. Unless otherwise noted, materials obtained from commercial suppliers were used without further purification. All reactions were carried out in flame-dried 25-mL Schlenk tubes with Teflon screw capsunder argon. $[Rh(cod)Cl]_2$ were purchased from Strem. **1d**[2], **1e**[2] and **D-1a**[2] were synthesized using standard procedures. Unless otherwise noted, materials obtained from commercial suppliers were used without further purification.

2 Supplementary Discussion

2.1 General Procedure for Direct Arylation of Arylphosphines with Arylbromides through One-Fold C–H Activation

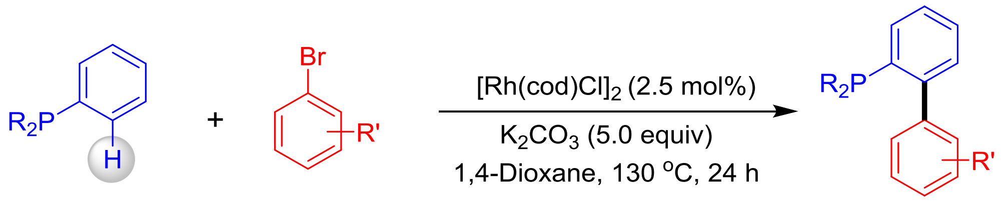

1 2 3

To an oven-dried Schlenk tube, arylphosphines **1** (1.0 equiv, 0.20 mmol), arylbromides **2** (3.0 equiv, 0.60 mmol), $[Rh(cod)Cl]_2$ (2.5 mol%, 2.5 mg, 0.005 mmol), K_2CO_3 (5.0 equiv, 138 mg, 1.0 mmol) were dissolved in 1,4-dioxane (0.3 mL). The mixture was stirred at 130 °C under argon for 24 hours. Upon the completion of the reaction, the solvent was removed. The crude mixture was directly subjected to column chromatography on silica gel using petroleum-ether/EtOAc as eluent to give the desired products **3**.

(5'-(*Tert*-butyl)-2'-methoxy-[1,1'-biphenyl]-2-yl)diphenylphosphane (3aa)

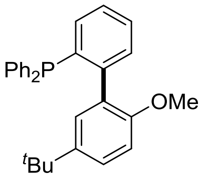

Following the general procedure, the reaction of **1a** (52.4 mg, 0.20 mmol), **2a** (145.2 mg, 0.6 mmol), $[Rh(cod)Cl]_2$ (2.5 mol%, 2.5 mg, 0.005 mmol), K_2CO_3 (5.0 equiv, 138 mg, 1.0 mmol), in 1,4-dioxane (0.3 mL) at 130 °C. After 24 h, purification by column chromatography on silica gel (petroleum-ether: ethyl acetate = 200:1) yield **3aa** (61.1 mg, 72%) as a colorless oil. **^{1}H NMR (500 MHz, Chloroform-*d*)** δ 7.39 (td, J = 7.5, 1.4 Hz, 1H), 7.34 – 7.32 (m, 1H), 7.28 – 7.23 (m, 8H), 7.22 – 7.16 (m, 4H), 7.11 – 7.07 (m, 1H), 6.94 (d, J = 2.5 Hz, 1H), 6.80 (d, J = 8.6 Hz, 1H), 3.56 (s, 3H), 1.10 (s, 9H). **^{13}C NMR (126 MHz, Chloroform-*d*)** δ 154.1, 145.4 (d, J = 31.7 Hz), 142.2, 138.1 (d, J = 12.6 Hz), 136.8 (d, J = 11.6 Hz), 134.1, 133.8 (d, J = 10.2 Hz), 133.6 (d,

J = 9.8 Hz), 130.5 (d, *J* = 5.6 Hz), 129.7 (d, *J* = 7.0 Hz), 128.8 (d, *J* = 3.4 Hz), 128.5, 128.3, 128.2, 128.1, 128.02, 127.95, 127.2, 125.5, 109.9, 55.1, 33.8, 31.2. **^{31}P NMR (162 MHz, CDCl$_3$)** δ -12.6. ATR-FTIR (cm^{-1}): 3053, 2930, 1600, 1440, 1355, 1125, 1047, 698. HRMS m/z (ESI): calcd for $C_{29}H_{30}OP$ $(M + H)^+$ 425.2029, found 425.2023.

(5'-Benzhydryl-2'-methoxy-[1,1'-biphenyl]-2-yl)diphenylphosphane (3ab)

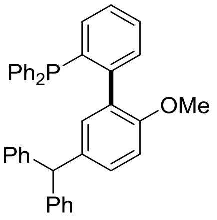

Following the general procedure, the reaction of **1a** (52.4 mg, 0.20 mmol), **2b** (211.2 mg, 0.6 mmol), [Rh(cod)Cl]$_2$ (2.5 mol%, 2.5 mg, 0.005 mmol), K_2CO_3 (5.0 equiv, 138 mg, 1.0 mmol), in 1,4-dioxane (0.3 mL) at 130 ^{o}C. After 18 h, purification by column chromatography on silica gel (petroleum-ether: ethyl acetate = 200:1) yield **3ab** (71.5 mg, 67%) as a white solid; mp = 222−223 °C. **^{1}H NMR (500 MHz, Chloroform-*d*)** δ 7.33 (t, *J* = 7.4 Hz, 1H), 7.28 – 7.20 (m, 14H), 7.18 – 7.11 (m, 4H), 7.08 – 6.97 (m, 6H), 6.81 – 6.73 (m, 2H), 5.34 (s, 1H), 3.51 (s, 3H). **^{13}C NMR (126 MHz, Chloroform-*d*)** δ 155.0, 145.0 (d, *J* = 31.6 Hz), 144.2 (d, *J* = 16.2 Hz), 138.1 (d, *J* = 12.7 Hz), 137.0 (d, *J* = 12.1 Hz), 135.1, 134.1, 133.7 (d, *J* = 12.1 Hz), 133.6 (d, *J* = 11.6 Hz), 132.5 (d, *J* = 3.0 Hz), 130.4 (d, *J* = 5.7 Hz), 130.3, 130.3, 129.8, 129.5, 128.6, 128.32, 128.25 (d, *J* = 3.6 Hz), 128.2, 128.1 (d, *J* = 6.4 Hz), 128.0, 127.4, 126.1, 110.2, 56.0, 55.1. **^{31}P NMR (162 MHz, CDCl$_3$)** δ -12.8. ATR-FTIR (cm^{-1}): 3047, 2907, 1751, 1470, 1100, 856, 748, 456. HRMS m/z (ESI): calcd for $C_{38}H_{32}OP$ $(M + H)^+$ 535.2185, found 535.2181.

(2'-Methoxy-5'-((triisopropylsilyl)oxy)-[1,1'-biphenyl]-2-yl)diphenylphosphane (3ac)

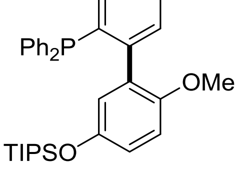

Following the general procedure, the reaction of **1a** (52.4 mg, 0.20 mmol), **2c** (215.4 mg, 0.6 mmol), [Rh(cod)Cl]$_2$ (5 mol%, 4.9 mg, 0.01 mmol), K_2CO_3 (5.0 equiv, 138 mg, 1.0 mmol), in 1,4-dioxane (0.3 mL) at 130 ^{o}C. After 24 h, purification by column chromatography on silica gel (petroleum-ether: ethyl acetate = 300:1) yield

3ac (73.4 mg, 68%) as a colorless oil. **^{1}H NMR (500 MHz, Chloroform-*d*)** δ 7.39 (t, *J* = 8.0 Hz, 1H), 7.28 – 7.24 (m, 10H), 7.15 – 7.07 (m, 3H), 6.80 (dd, *J* = 8.8, 3.0 Hz, 1H), 6.69 (d, *J* = 8.8 Hz, 1H), 6.62 (d, *J* = 3.0 Hz, 1H), 3.44 (s, 3H), 1.15 – 1.08 (m, 3H), 1.03 (d, *J* = 5.7 Hz, 18H). **^{13}C NMR (126 MHz, Chloroform-*d*)** δ 151.0, 148.9, 145.1 (d, *J* = 32.9 Hz), 138.5, 138.3, 136. 9 (d, *J* = 12.4 Hz), 134.3 (d, *J* = 2.0 Hz), 133.6 (d, *J* = 8.8 Hz), 133.44 (d, *J* = 9.0 Hz), 131.42 (d, *J* = 6.9 Hz), 130.2 (d, *J* = 6.0 Hz), 128.8, 128.2 (d, *J* = 6.3 Hz), 128.1, 128.0, 127.9, 127.4, 122.8 (d, *J* = 2.3 Hz), 119.4, 111.0, 55.3, 17.9, 12.5. **^{31}P NMR (162 MHz, $CDCl_3$)** δ -13.2. ATR-FTIR (cm^{-1}): 3551, 2970, 1362, 1121, 952, 795, 664, 521. HRMS m/z (ESI): calcd for $C_{34}H_{42}O_2PSi$ $(M + H)^+$ 541.2686, found 541.2687.

(2'-Methoxy-5'-methyl-[1,1'-biphenyl]-2-yl)diphenylphosphane (3ad)

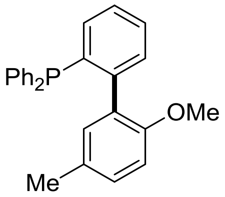

Following the general procedure, the reaction of **1a** (52.4 mg, 0.20 mmol), **2d** (119.4 mg, 0.6 mmol), $[Rh(cod)Cl]_2$ (2.5 mol%, 2.5 mg, 0.005 mmol), K_2CO_3 (5.0 equiv, 138 mg, 1.0 mmol), in 1,4-dioxane (0.3 mL) at 120 ^oC. After 12 h, purification by column chromatography on silica gel (petroleum-ether: ethyl acetate = 200:1) yield **3ad** (39.7 mg, 52%) as a colorless oil. **^{1}H NMR (500 MHz, Chloroform-*d*)** δ 7.38 (td, *J* = 7.5, 1.4 Hz, 1H), 7.27 (q, *J* = 4.4, 3.1 Hz, 10H), 7.21 – 7.14 (m, 2H), 7.15 – 7.05 (m, 2H), 6.77 (d, *J* = 2.3 Hz, 1H), 6.72 (d, *J* = 8.3 Hz, 1H), 3.38 (s, 3H), 2.18 (s, 3H). **^{13}C NMR (126 MHz, Chloroform-*d*)** δ 154.5, 145.2 (d, *J* = 32.5 Hz), 138.6 (d, *J* = 12.4 Hz), 137.8 (d, *J* = 15.6 Hz), 137.1 (d, *J* = 11.8 Hz), 134.2 (d, *J* = 1.8 Hz), 133.8 (d, *J* = 13.5 Hz), 133.6 (d, *J* = 12.6 Hz), 132.2 (d, *J* = 2.5 Hz), 130.4, 130.3, 129.2, 128.9, 128.7, 128.2, 128.13, 128.11, 128.0, 127.3, 110.1, 54.9, 20.4. **^{31}P NMR (162 MHz, $CDCl_3$)** δ -12.9. ATR-FTIR (cm-1): 3041, 2890, 1621, 1545, 1266, 1154, 785, 696. HRMS m/z (ESI): calcd for $C_{26}H_{24}OP$ $(M + H)^+$ 383.1559, found 383.1564.

(2'-Methoxy-5'-(trifluoromethyl)-[1,1'-biphenyl]-2-yl)diphenylphosphane (3ae)

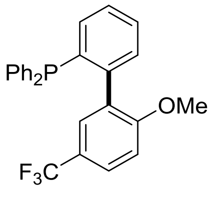

Following the general procedure, the reaction of **1a** (52.4 mg, 0.20 mmol), **2e** (151.8 mg, 0.6 mmol), $[Rh(cod)Cl]_2$ (2.5 mol%, 2.5 mg,

0.005 mmol), K_2CO_3 (5.0 equiv, 138 mg, 1.0 mmol), in 1,4-dioxane (0.3 mL) at 120 ^oC. After 12 h, purification by column chromatography on silica gel (petroleum-ether: ethyl acetate = 300:1) yield **3ae** (43.6 mg, 50%) as a colorless oil. **^{1}H NMR (500 MHz, Chloroform-*d*)** δ 7.54 (dd, *J* = 8.6, 1.8 Hz, 1H), 7.40 (td, *J* = 7.5, 1.3 Hz, 1H), 7.32 – 7.22 (m, 11H), 7.19 – 7.11 (m, 3H), 6.87 (d, *J* = 8.6 Hz, 1H), 3.49 (s, 3H). **^{13}C NMR (126 MHz, Chloroform-*d*)** δ 159.1, 143.3 (d, *J* = 31.3 Hz), 137.5 (d, *J* = 12.4 Hz), 137.4 (d, *J* = 12.9 Hz), 137.2, 134.0, 133.8 (d, *J* = 16.4 Hz), 133.6 (d, *J* = 15.5 Hz), 130.9 (d, *J* = 6.8 Hz), 130.3 (d, *J* = 5.5 Hz), 128.8, 128.6, 128.4, 128.3, 128.22, 128.20, 127.9, 126.3 (q, *J* = 3.9 Hz), 122.1 (d, *J* = 32.7 Hz), 110.1, 55.2. **^{31}P NMR (162 MHz, $CDCl_3$)** δ -12.2. **^{19}F NMR (376 MHz, Chloroform-*d*)** δ 61.4. ATR-FTIR (cm^{-1}): 3405, 3108, 2756, 1684, 1500, 1325, 1161, 613. HRMS m/z (ESI): calcd for $C_{26}H_{21}F_3OP$ $(M + H)^+$ 437.1277, found 437.1279.

(2-(3-Methoxynaphthalen-2-yl)phenyl)diphenylphosphane (3af)

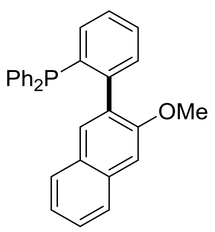

Following the general procedure, the reaction of **1a** (52.4 mg, 0.20 mmol), **2f** (142.2 mg, 0.6 mmol), $[Rh(cod)Cl]_2$ (2.5 mol%, 2.5 mg, 0.005 mmol), K_2CO_3 (5.0 equiv, 138 mg, 1.0 mmol), in 1,4-dioxane (0.3 mL) at 120 ^oC. After 12 h, purification by column chromatography on silica gel (petroleum-ether: ethyl acetate = 200:1) yield **3af** (37.6 mg, 45%) as a white solid; mp = 107−108 °C. **^{1}H NMR (500 MHz, Chloroform-*d*)** δ 7.73 (d, *J* = 8.1 Hz, 1H), 7.58 (d, *J* = 8.1 Hz, 1H), 7.46 – 7.39 (m, 3H), 7.38 – 7.24 (m, 11H), 7.21 – 7.13 (m, 3H), 7.08 (s, 1H), 3.53 (s, 3H). **^{13}C NMR (126 MHz, Chloroform-*d*)** δ 155.5, 144.8 (d, *J* = 32.5 Hz), 137.4 (d, *J* = 12.2 Hz), 134.34, 134.31 (d, *J* = 1.8 Hz), 133.8, 133.6, 133.5, 130.7 (d, *J* = 2.7 Hz), 130.5 (d, *J* = 6.0 Hz), 128.8, 128.33, 128.31, 128.2 (d, *J* = 5.5 Hz), 128.0 (d, *J* = 8.2 Hz), 127.7 (d, *J* = 15.5 Hz), 126.5, 126.2, 123.6, 104.9, 54.9. **^{31}P NMR (162 MHz, $CDCl_3$)** δ -13.0. ATR-FTIR (cm^{-1}): 3400, 2851, 1684, 1612, 1513, 1173, 1110, 578. HRMS m/z (ESI): calcd for $C_{29}H_{24}OP$ $(M + H)^+$ 419.1559, found 419.1512.

(2-(Phenanthren-9-yl)phenyl)diphenylphosphane (3ag)

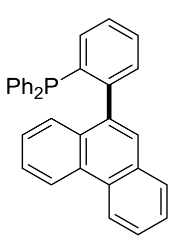

Following the general procedure, the reaction of **1a** (52.4 mg, 0.20 mmol), **2g** (153.0 mg, 0.6 mmol), [Rh(cod)Cl]$_2$ (2.5 mol%, 2.5 mg, 0.005 mmol), K_2CO_3 (5.0 equiv, 138 mg, 1.0 mmol), in 1,4-dioxane (0.3 mL) at 120 °C. After 24 h, purification by column chromatography on silica gel (petroleum-ether) yield **3ag** (52.5 mg, 60%) as a white solid; mp = 102−103 °C. **^{1}H NMR (500 MHz, Chloroform-*d*)** δ 8.71 (dd, *J* = 15.6, 8.3 Hz, 2H), 7.65 – 7.59 (m, 2H), 7.55 – 7.26 (m, 10H), 7.25 – 7.05 (m, 9H). **^{13}C NMR (126 MHz, Chloroform-*d*)** δ 146.2 (d, *J* = 31.3 Hz), 138.2 (d, *J* = 12.8 Hz), 137.8, 137.6 (d, *J* = 4.6 Hz), 137.5, 134.1 (d, *J* = 20.1 Hz), 133.9, 133.7 (d, *J* = 19.9 Hz), 131.8, 131.02, 131.01, 130.2 (d, *J* = 8.7 Hz), 129.2 (d, *J* = 3.6 Hz), 128.7, 128.6 (d, *J* = 7.2 Hz), 128.4, 128.3 (d, *J* = 4.2 Hz), 128.2 (d, *J* = 3.9 Hz), 127.8, 127.6, 127.2, 126.9 (d, *J* = 11.9 Hz), 126.72, 126.70 (d, *J* = 4.5 Hz), 126.4 (d, *J* = 5.4 Hz), 122.82 (d, *J* = 15.2 Hz), 122.81, 122.5. **^{31}P NMR (162 MHz, $CDCl_3$)** δ -14.2. ATR-FTIR (cm^{-1}): 3407, 2931, 1681, 1510, 1414, 1083, 766, 674. HRMS m/z (ESI): calcd for $C_{32}H_{24}P$ $(M + H)^+$ 439.1610, found 439.1608.

(3',5'-Di-tert-butyl-[1,1'-biphenyl]-2-yl)diphenylphosphane (3ah)

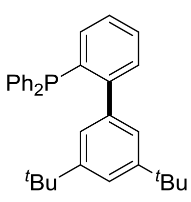

Following the general procedure, the reaction of **1a** (52.4 mg, 0.20 mmol), **2h** (161.4 mg, 0.6 mmol), [Rh(cod)Cl]$_2$ (2.5 mol%, 2.5 mg, 0.005 mmol), K_2CO_3 (5.0 equiv, 138 mg, 1.0 mmol), in 1,4-dioxane (0.3 mL) at 130 °C. After 24 h, purification by column chromatography on silica gel (petroleum-ether) yield **3ah** (67.5 mg, 75%) as a white solid; mp = 127−128 °C. **^{1}H NMR (500 MHz, Chloroform-*d*)** δ 7.39 (dd, *J* = 4.5, 2.1 Hz, 2H), 7.33 – 7.25 (m, 8H), 7.23 – 7.19 (m, 4H), 7.06 – 7.03 (m, 2H), 7.01 (dd, *J* = 7.6, 3.9 Hz, 1H), 1.19 (s, 18H). **^{13}C NMR (126 MHz, Chloroform-*d*)** δ 149.8, 149.3 (d, *J* = 28.1 Hz), 140.7 (d, *J* = 5.8 Hz), 138.1 (d, *J* = 12.6 Hz), 135.6 (d, *J* = 14.7 Hz), 134.3, 134.1, 133.9, 133.5, 130.3 (d, *J* = 4.8 Hz), 128.7, 128.6, 128.5 (d, *J* = 6.9 Hz), 128.4, 128.3, 127.0, 124.0 (d, *J* = 4.0 Hz), 121.0, 34.7, 31.3. **^{31}P NMR (162 MHz, $CDCl_3$)** δ -12.3. ATR-FTIR (cm^{-1}): 3400, 2933, 1715, 1623, 1415, 761, 679, 555. HRMS m/z (ESI): calcd for $C_{32}H_{36}P$ $(M + H)^+$ 451.2549, found 451.2534.

(3',5'-Di-tert-butyl-4'-methoxy-[1,1'-biphenyl]-2-yl)diphenylphosphane (3ai)

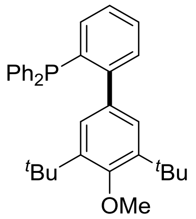

Following the general procedure, the reaction of **1a** (52.4 mg, 0.20 mmol), **2i** (179.4 mg, 0.6 mmol), $[Rh(cod)Cl]_2$ (2.5 mol%, 2.5 mg, 0.005 mmol), K_2CO_3 (5.0 equiv, 138 mg, 1.0 mmol), in 1,4-dioxane (0.3 mL) at 130 ^oC. After 24 h, purification by column chromatography on silica gel (petroleum-ether: ethyl acetate = 200:1) yield **3ai** (68.2 mg, 71%) as a white solid; mp = 165−167 °C. **^{1}H NMR (500 MHz, Chloroform-*d*)** δ 7.39 – 7.33 (m, 2H), 7.29 – 7.23 (m, 6H), 7.20 (dt, *J* = 7.0, 2.6 Hz, 5H), 7.09 (d, *J* = 1.1 Hz, 2H), 6.95 (dd, *J* = 7.5, 4.1 Hz, 1H), 3.63 (s, 3H), 1.29 (s, 18H). **^{13}C NMR (126 MHz, Chloroform-*d*)** δ 158.6, 148.4 (d, *J* = 25.9 Hz), 142.5, 137.7 (d, *J* = 12.6 Hz), 136.1, 136.0, 135.8 (d, *J* = 5.3 Hz), 134.2, 134.1, 134.0, 133.7, 130.2 (d, *J* = 4.3 Hz), 128.5, 128.4, 128.31, 128.25, 128.0 (d, *J* = 4.1 Hz), 126.9, 64.1, 35.6, 31.9. **^{31}P NMR (162 MHz, $CDCl_3$)** δ -10.7. ATR-FTIR (cm^{-1}): 3400, 2935, 1860, 1677, 1523, 753, 660, 534. HRMS m/z (ESI): calcd for $C_{33}H_{38}OP$ $(M + H)^+$ 481.2655, found 481.2657.

(5'-(Tert-butyl)-2'-methoxy-5-methyl-[1,1'-biphenyl]-2-yl)di-p-tolylphosphane (3ba)

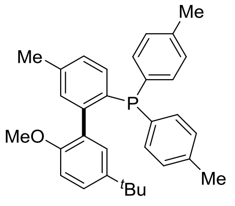

Following the general procedure, the reaction of **1b** (60.8 mg, 0.20 mmol), **2a** (145.2 mg, 0.6 mmol), $[Rh(cod)Cl]_2$ (2.5 mol%, 2.5 mg, 0.005 mmol), K_2CO_3 (5.0 equiv, 138 mg, 1.0 mmol), in 1,4-dioxane (0.3 mL) at 130 ^oC. After 24 h, purification by column chromatography on silica gel (petroleum-ether: ethyl acetate = 200:1) yield **3ba** (68.9 mg, 74%) as a colorless oil. **^{1}H NMR (500 MHz, Chloroform-*d*)** δ 7.26 (dd, *J* = 8.6, 2.6 Hz, 1H), 7.14 – 7.03 (m, 10H), 6.99 (dd, *J* = 7.8, 3.7 Hz, 1H), 6.89 (d, *J* = 2.5 Hz, 1H), 6.80 (d, *J* = 8.6 Hz, 1H), 3.57 (s, 3H), 2.37 (s, 3H), 2.31 (d, *J* = 7.5 Hz, 6H), 1.10 (s, 9H). **^{13}C NMR (101 MHz, Chloroform-*d*)** δ 154.2, 145.3 (d, *J* = 31.8 Hz), 142.2, 138.3, 137.8 (d, *J* = 45.4 Hz), 135.1 (d, *J* = 11.1 Hz), 134.02, 133.99, 133.8, 133.6 (d, *J* = 2.9 Hz), 133.5, 131.2 (d, *J* = 5.8 Hz), 130.0 (d, *J* = 6.9 Hz), 129.0 (d, *J* = 7.0 Hz), 128.93, 128.90, 128.8, 128.2, 125.3, 109.9, 55.3, 33.9, 31.3, 21.4,

21.30, 21.27. **^{31}P NMR (162 MHz, $CDCl_3$)** δ -15.3. ATR-FTIR (cm^{-1}): 3406, 3111, 2460, 1590, 1512, 1245, 671, 561. HRMS m/z (ESI): calcd for $C_{32}H_{36}OP$ $(M + H)^+$ 467.2498, found 467.2486.

(5'-(Tert-butyl)-2'-methoxy-4-methyl-[1,1'-biphenyl]-2-yl)di-m-tolylphosphane (3ca)

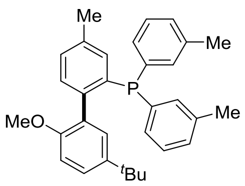

Following the general procedure, the reaction of **1c** (60.8 mg, 0.20 mmol), **2a** (145.2 mg, 0.6 mmol), $[Rh(cod)Cl]_2$ (2.5 mol%, 2.5 mg, 0.005 mmol), K_2CO_3 (5.0 equiv, 138 mg, 1.0 mmol), in 1,4-dioxane (0.3 mL) at 130 ^{o}C. After 24 h, purification by column chromatography on silica gel (petroleum-ether: ethyl acetate = 200:1) yield **3ca** (56.8 mg, 74%) as a white solid; mp = 128−129 °C. **^{1}H NMR (500 MHz, Chloroform-*d*)** δ 7.25 (dd, *J* = 8.6, 2.6 Hz, 1H), 7.21 – 7.12 (m, 4H), 7.06 (t, *J* = 7.3 Hz, 4H), 6.97 (t, *J* = 6.7 Hz, 2H), 6.91 (d, *J* = 2.3 Hz, 2H), 6.79 (d, *J* = 8.6 Hz, 1H), 3.59 (s, 3H), 2.26 (s, 9H), 1.09 (s, 9H). **^{13}C NMR (101 MHz, Chloroform-*d*)** δ 154.2, 142.3, 142.1, 138.1, 138.0 (d, *J* = 5.5 Hz), 137.5 (d, *J* = 20.9 Hz), 136.8, 136.7, 134.6 (d, *J* = 15.5 Hz), 134.5, 134.4 (d, *J* = 9.8 Hz), 130.8 (d, *J* = 8.4 Hz), 130.7 (d, *J* = 8.0 Hz), 130.3 (d, *J* = 5.8 Hz), 129.7 (d, *J* = 7.1 Hz), 129.5, 129.4, 129.1 (d, *J* = 3.3 Hz), 128.7, 128.3 (d, *J* = 6.4 Hz), 128.1 (d, *J* = 5.4 Hz), 127.9 (d, *J* = 5.6 Hz), 125.3, 109.9, 55.3, 33.8, 31.2, 21.44, 21.40. **^{31}P NMR (162 MHz, $CDCl_3$)** δ -12.4. ATR-FTIR (cm^{-1}): 3502, 2794, 1865, 1589, 1450, 1096, 858, 566. HRMS m/z (ESI): calcd for $C_{32}H_{36}OP$ $(M + H)^+$ 467.2498, found 467.2493.

(5'-(Tert-butyl)-2',5-dimethoxy-[1,1'-biphenyl]-2-yl)bis(4-methoxyphenyl)phos-phane (3da)

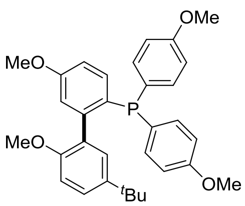

Following the general procedure, the reaction of **1d** (70.4 mg, 0.20 mmol), **2a** (145.2 mg, 0.6 mmol), $[Rh(cod)Cl]_2$ (2.5 mol%, 2.5 mg, 0.005 mmol), K_2CO_3 (5.0 equiv, 138 mg, 1.0 mmol), in 1,4-dioxane (0.3 mL) at 130 ^{o}C. After 24 h, purification by column chromatography on silica gel (petroleum-ether: ethyl acetate = 100:1) yield

3da (67.8 mg, 66%) as a colorless oil. **^{1}H NMR (500 MHz, Chloroform-*d*)** δ 7.31 (dd, *J* = 8.6, 2.5 Hz, 1H), 7.14 (q, *J* = 8.7 Hz, 4H), 7.02 (dd, *J* = 8.4, 3.5 Hz, 1H), 6.95 (d, *J* = 2.5 Hz, 1H), 6.90 (dd, *J* = 3.6, 2.7 Hz, 1H), 6.88 – 6.81 (m, 6H), 3.83 (s, 3H), 3.81 (s, 6H), 3.62 (s, 3H), 1.14 (s, 9H). **^{13}C NMR (101 MHz, Chloroform-*d*)** δ 159.8, 159.62, 159.55, 154.1, 146.6 (d, *J* = 33.0 Hz), 142.2, 135.2, 135.0 (d, *J* = 9.2 Hz), 134.9 (d, *J* = 8.4 Hz), 129.9, 129.8 (d, *J* = 6.7 Hz), 128.7 (d, *J* = 3.1 Hz), 126.4, 125.5, 115.8 (d, *J* = 6.1 Hz), 114.7, 113.9 (d, *J* = 7.5 Hz), 113.8 (d, *J* = 7.0 Hz), 113.4, 110.0, 55.3, 55.19, 55.17, 33.9, 31.3. **^{31}P NMR (162 MHz, $CDCl_3$)** δ -17.7. ATR-FTIR (cm^{-1}): 3455, 2849, 1710, 1512, 1331, 1069, 834, 568. HRMS m/z (ESI): calcd for $C_{32}H_{36}O_4P$ $(M + H)^+$ 515.2346, found 515.2345.

(5'-(Tert-butyl)-2',4-dimethoxy-[1,1'-biphenyl]-2-yl)bis(3-methoxyphenyl)phos-phane (3ea)

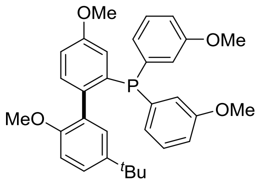

Following the general procedure, the reaction of **1e** (70.4 mg, 0.20 mmol), **2a** (145.2 mg, 0.6 mmol), $[Rh(cod)Cl]_2$ (2.5 mol%, 2.5 mg, 0.005 mmol), K_2CO_3 (5.0 equiv, 138 mg, 1.0 mmol), in 1,4-dioxane (0.3 mL) at 130 °C. After 24 h, purification by column chromatography on silica gel (petroleum-ether: ethyl acetate = 100:1) yield **3ea** (62.7 mg, 61%) as a colorless oil. **^{1}H NMR (500 MHz, Chloroform-*d*)** δ 7.25 – 7.16 (m, 4H), 6.93 (dd, *J* = 8.2, 2.7 Hz, 2H), 6.83 – 6.75 (m, 7H), 6.64 (dd, *J* = 3.8, 2.7 Hz, 1H), 3.70 (s, 6H), 3.67 (s, 3H), 3.60 (s, 3H), 1.09 (s, 9H). **^{13}C NMR (101 MHz, Chloroform-*d*)** δ 159.4, 159.3, 159.2, 158.4, 142.1, 139.3 (d, *J* = 12.9 Hz), 138.0 (d, *J* = 13.3 Hz), 137.6, 137.4, 131.5 (d, *J* = 6.0 Hz), 129.3 (d, *J* = 3.5 Hz), 129.3, 129.2, 129.14, 129.09, 126.2 (d, *J* = 18.4 Hz), 125.3, 119.5, 119.3, 119.1, 119.0, 114.0 (d, *J* = 13.1 Hz), 113.8, 110.0, 55.3, 55.12, 55.11, 33.8, 31.2. **^{31}P NMR (162 MHz, $CDCl_3$)** δ -9.8. ATR-FTIR (cm^{-1}): 3406, 2937, 1625, 1510, 1110, 1042, 861, 571. HRMS m/z (ESI): calcd for $C_{32}H_{36}O_4P$ $(M + H)^+$ 515.2346, found 515.2355.

(5'-(Tert-butyl)-5-fluoro-2'-methoxy-[1,1'-biphenyl]-2-yl)bis(4-fluorophenyl)-phos-phane (3fa)

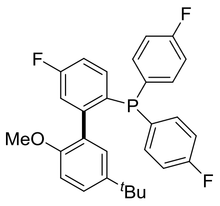

Following the general procedure, the reaction of **1f** (63.2 mg, 0.20 mmol), **2a** (145.2 mg, 0.6 mmol), $[Rh(cod)_2]OTf$ (10 mol%, 9.4 mg, 0.02 mmol), K_2CO_3 (5.0 equiv, 138 mg, 1.0 mmol), in 1,4-dioxane (0.3 mL) at 130 ^{o}C. After 24 h, purification by column chromatography on silica gel (petroleum-ether: ethyl acetate = 300:1) yield **3fa** (55.4 mg, 58%) as a white solid; mp = 152−153 °C. **^{1}H NMR (500 MHz, Chloroform-*d*)** δ 7.29 (dd, *J* = 8.6, 2.5 Hz, 1H), 7.13 (dt, *J* = 8.6, 6.6 Hz, 4H), 7.06 – 7.03 (m, 1H), 6.98 (t, *J* = 8.3 Hz, 6H), 6.92 (d, *J* = 2.5 Hz, 1H), 6.79 (d, *J* = 8.6 Hz, 1H), 3.57 (s, 3H), 1.13 (s, 9H). **^{13}C NMR (101 MHz, Chloroform-*d*)** δ 163.3 (d, *J* = 248.6 Hz), 163.0 (d, *J* = 249.6 Hz), 153.9, 147.6 (d, *J* = 8.4 Hz), 147.3 (d, *J* = 8.1 Hz), 142.5, 135.4 (d, *J* = 8.5 Hz), 133.3, 132.4 (d, *J* = 2.9 Hz), 132.3 (d, *J* = 2.9 Hz), 128.4 (d, *J* = 3.6 Hz), 126.0, 117.9 (d, *J* = 5.8 Hz), 117.8, 117.8, 115.6 (d, *J* = 43.6 Hz), 115.5, 114.7, 114.5, 110.0, 55.1, 33.9, 31.3. **^{31}P NMR (162 MHz, $CDCl_3$)** δ -15.9. **^{19}F NMR (376 MHz, Chloroform-*d*)** δ -112.7, -112.90, -112.90, -113.1. ATR-FTIR (cm-1): 3410, 3123, 2910, 1666, 1612, 1418, 1151, 732, 687. HRMS m/z (ESI): calcd for $C_{29}H_{27}F_3OP$ $(M + H)^+$ 479.1746, found 479.1744.

(5'-(Tert-butyl)-4-chloro-2'-methoxy-[1,1'-biphenyl]-2-yl)bis(3-chlorophenyl)-phosphane (3ga)

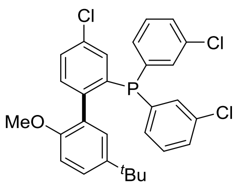

Following the general procedure, the reaction of **1g** (72.6 mg, 0.20 mmol), **2a** (145.2 mg, 0.6 mmol), $[Rh(cod)_2]OTf$ (10 mol%, 9.4 mg, 0.02 mmol), K_2CO_3 (5.0 equiv, 138 mg, 1.0 mmol), in 1,4-dioxane (0.3 mL) at 130 ^{o}C. After 24 h, purification by column chromatography on silica gel (petroleum-ether: ethyl acetate = 100:1) yield **3ga** (54.7 mg, 52%) as a white solid; mp = 198−199 °C. **^{1}H NMR (500 MHz, Chloroform-*d*)** δ 7.39 (dd, *J* = 8.2, 2.2 Hz, 1H), 7.31 – 7.24 (m, 6H), 7.13 (d, *J* = 7.2 Hz, 2H), 7.07 – 7.03 (m, 2H), 6.97 – 6.92 (m, 2H), 6.75 (d, *J* = 8.6 Hz, 1H), 3.57 (s, 3H), 1.15 (s, 9H). **^{13}C NMR (101 MHz, Chloroform-*d*)** δ 154.0, 143.5 (d, *J* = 30.3 Hz), 142.5, 139.0 (d, *J* = 17.0 Hz), 138.2 (d, *J* = 15.8 Hz), 134.8 (d, *J* = 8.2 Hz), 134.5, 133.6 (d, *J* = 4.6 Hz), 133.3, 132.9, 132.2 (d, *J* = 5.1 Hz), 131.9 (d, *J* = 10.2 Hz),

131.7 (d, J = 3.0 Hz), 131.6, 130.2, 129.9 (d, J = 6.6 Hz), 129.6 (d, J = 7.0 Hz), 129.3, 129.2, 128.9, 128.5 (d, J = 3.6 Hz), 128.0 (d, J = 6.5 Hz), 126.1, 110.0, 55.1, 33.9, 31.3. **^{31}P NMR (162 MHz, CDCl$_3$)** δ -10.2. ATR-FTIR (cm^{-1}): 3498, 2810, 1960, 1755, 1566, 1062, 916, 698. HRMS m/z (ESI): calcd for $C_{29}H_{27}Cl_3OP$ $(M + H)^+$ 527.0860, found 527.0851.

2.2 General Procedure for Arylation of Arylphosphines with Arylbromides through Two-Fold C–H Activation

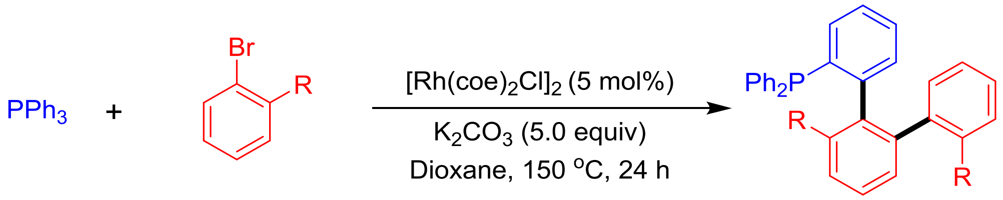

1a **2** **3**

In an oven-dried Schlenk tube, arylphosphines **1** (1.0 equiv, 0.20 mmol), arylbromides **2** (5.0 equiv, 1.0 mmol), $[Rh(coe)_2Cl]_2$ (5 mol%, 7.17 mg, 0.01 mmol), K_2CO_3 (5.0 equiv, 138 mg, 1.0 mmol) were dissolved in 1,4-dioxane (0.5 mL). The mixture was stirred at 150 °C under argon for 24 hours. Upon the completion of the reaction, the solvent was removed. The crude mixture was directly subjected to column chromatography on silica gel using petrol ether/EtOAc as eluent to give the desired products **3**.

(2'',6'-Dimethoxy-[1,1':2',1''-terphenyl]-2-yl)diphenylphosphane (3aj)

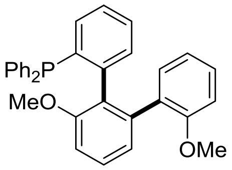

Following the general procedure, the reaction of **1a** (52.4 mg, 0.20 mmol), **2j** (185 mg, 1.0 mmol), $[Rh(coe)_2Cl]_2$ (5 mol%, 7.17 mg, 0.01 mmol), K_2CO_3 (5.0 equiv, 138 mg, 1.0 mmol), in 1,4-dioxane (0.5 mL) at 150 °C. After 24 h, purification by column chromatography on silica gel (petroleum-ether: ethyl acetate = 30:1) yield **3aj** (72.9 mg, 77%) as a white solid; mp = 202−204 °C. **^{1}H NMR (500 MHz, Chloroform-*d*)** δ 7.38 (t, J = 7.9 Hz, 1H), 7.30 – 7.15 (m, 10H), 7.14 – 6.92 (m, 7H), 6.79 (d, J = 8.3 Hz, 1H), 6.72 – 6.60 (m, 2H), 3.63 (s, 3H), 3.20 (s, 3H). **^{13}C NMR**

(101 MHz, Chloroform-*d*) δ 156.7 (d, *J* = 66.8 Hz), 144.2 (d, *J* = 34.6 Hz), 139.3, 139.0 (d, *J* = 13.5 Hz), 136.8 (d, *J* = 9.8 Hz), 134.2, 133.9, 133.7, 133.1, 132.9, 131.7, 130.9, 130.3, 130.1 (d, *J* = 7.1 Hz), 128.2, 128.1, 128.0, 127.97, 127.94, 127.91, 127.6, 126.9, 122.6, 119.5, 110.0, 108.8, 55.0, 54.6. **^{31}P NMR (162 MHz, $CDCl_3$)** δ -14.1. ATR-FTIR (cm^{-1}): 3566, 2871, 1710, 1498, 1214, 1032, 815, 561. HRMS m/z (ESI): calcd for $C_{32}H_{28}O_2P$ $(M + H)^+$ 475.1821, found 475.1813.

3',5''-Difluoro-2'',6'-dimethoxy-[1,1':2',1''-terphenyl]-2-yl)diphenylphosphane (3ak)

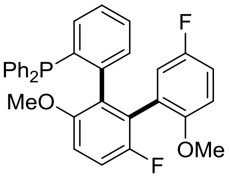

Following the general procedure, the reaction of **1a** (52.4 mg, 0.20 mmol), **2k** (203 mg, 1.0 mmol), $[Rh(coe)_2Cl]_2$ (5 mol%, 7.17 mg, 0.01 mmol), K_2CO_3 (5.0 equiv, 138 mg, 1.0 mmol), in 1,4-dioxane (0.5 mL) at 150 ^oC. After 24 h, purification by column chromatography on silica gel (petroleum-ether: ethyl acetate = 30:1) yield **3ak** (73.4 mg, 72%) as a white solid; mp = 188−190 °C. **^{1}H NMR (500 MHz, Chloroform-*d*)** δ 7.31 – 7.25 (m, 8H), 7.15 – 7.02 (m, 7H), 6.83 – 6.67 (m, 4H), 3.73 (s, 3H), 3.13 (s, 3H). **^{13}C NMR (101 MHz, Chloroform-*d*)** δ 156.1 (d, *J* = 238.7 Hz), 153.2, 154.3 (d, *J* = 237.9 Hz), 143.0, 142.6, 138.3 (d, *J* = 12.1 Hz), 137.4 (d, *J* = 12.0 Hz), 136.9, 136.8, 134.4 (d, *J* = 2.6 Hz), 133.9, 133.7, 133.1, 130.0 (d, *J* = 6.8 Hz), 128.4, 128.3 (d, *J* = 6.2 Hz), 128.14, 128.11 (d, *J* = 4.5 Hz), 127.4, 118.3 (d, *J* = 4.5 Hz), 118.1 (d, *J* = 4.4 Hz), 115.0, 114.7 (d, *J* = 3.6 Hz), 114.5, 111.0 (d, *J* = 8.3 Hz), 109.8 (d, *J* = 8.6 Hz), 56.0, 54.8. **^{31}P NMR (162 MHz, $CDCl_3$)** δ -14.1. **^{19}F NMR (376 MHz, Chloroform-*d*)** δ -124.5, -124.7. ATR-FTIR (cm-1): 3510, 2974, 1855, 1681, 1412, 1122, 862, 554. HRMS m/z (ESI): calcd for $C_{32}H_{26}F_2O_2P$ $(M + H)^+$ 511.1633, found 511.1630.

3',5''-Dichloro-2'',6'-dimethoxy-[1,1':2',1''-terphenyl]-2-yl)diphenylphosphane (3al)

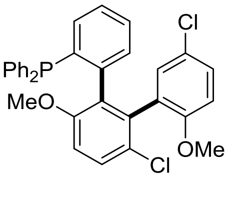

Following the general procedure, the reaction of **1a** (52.4 mg, 0.20 mmol), **2l** (219 mg, 1.0 mmol), $[Rh(coe)_2Cl]_2$ (5 mol%, 7.17

mg, 0.01 mmol), K_2CO_3 (5.0 equiv, 138 mg, 1.0 mmol), in 1,4-dioxane (0.5 mL) at 150 °C. After 24 h, purification by column chromatography on silica gel (petroleum-ether: ethyl acetate = 30:1) yield **3al** (66.1 mg, 61%) as a white solid; mp = 211−213 °C. **^{1}H NMR (500 MHz, Chloroform-*d*)** δ 7.43 (d, *J* = 8.8 Hz, 1H), 7.31 – 7.23 (m, 9H), 7.15 – 7.02 (m, 7H), 6.69 (d, *J* = 8.8 Hz, 2H), 3.76 (s, 3H), 2.97 (s, 3H). **^{13}C NMR (101 MHz, Chloroform-*d*)** δ 155.7 (d, *J* = 65.8 Hz), 143.2 (d, *J* = 34.5 Hz), 138.7 (d, *J* = 11.9 Hz), 136.9 (d, *J* = 12.1 Hz), 136.7 (d, *J* = 10.0 Hz), 136.0, 134.4 (d, *J* = 2.4 Hz), 134.1 (d, *J* = 20.4 Hz), 133.1, 132.9, 131.8 (d, *J* = 6.9 Hz), 130.2 (d, *J* = 5.1 Hz), 129.6 (d, *J* = 6.6 Hz), 129.4, 129.1, 128.7, 128.4, 128.3 (d, *J* = 1.9 Hz), 128.13, 128.11, 127.9, 127.5, 125.5, 124.7, 111.4, 110.3, 55.7, 54.4. **^{31}P NMR (162 MHz, $CDCl_3$)** δ -14.7. ATR-FTIR (cm^{-1}): 3510, 2795, 1812, 1630, 1490, 1062, 864, 630. HRMS m/z (ESI): calcd for $C_{32}H_{26}Cl_2O_2P$ $(M + H)^+$ 543.1042, found 543.1039.

Diphenyl(2-(9,9,9',9'-tetramethyl-9H,9'H-[3,4'-bifluoren]-4-yl)phenyl)phosphane (3am)

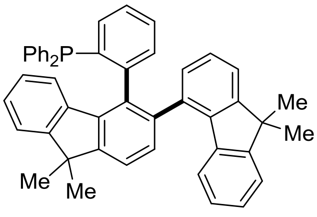

Following the general procedure, the reaction of **1a** (52.4 mg, 0.20 mmol), **2m** (272 mg, 1.0 mmol), $[Rh(coe)_2Cl]_2$ (5 mol%, 7.17 mg, 0.01 mmol), K_2CO_3 (5.0 equiv, 138 mg, 1.0 mmol), in 1,4-dioxane (0.5 mL) at 150 °C. After 24 h, purification by column chromatography on silica gel (petroleum-ether) yield **3am** (80.1 mg, 62%) as a white solid; mp = 176−179 °C. **^{1}H NMR (500 MHz, Chloroform-*d*)** δ 7.61 (d, *J* = 7.6 Hz, 1H), 7.39 – 7.35 (m, 2H), 7.30 – 7.24 (m, 5H), 7.17 – 6.85 (m, 16H), 6.56 (dd, *J* = 17.3, 7.6 Hz, 2H), 5.66 (d, *J* = 7.7 Hz, 1H), 1.62 (s, 3H), 1.59 (s, 3H), 1.47 (s, 3H), 1.33 (s, 3H). **^{13}C NMR (101 MHz, Chloroform-*d*)** δ 153.8 (d, *J* = 28.5 Hz), 153.2, 145.6 (d, *J* = 34.4 Hz), 139.6 (d, *J* = 58.7 Hz), 138.8 (d, *J* = 10.4 Hz), 138.5 (d, *J* = 2.7 Hz), 137.6 (d, *J* = 11.8 Hz), 137.3, 136.8, 136.4 (d, *J* = 13.3 Hz), 135.5 (d, *J* = 7.6 Hz), 134.7 (d, *J* = 22.3 Hz), 134.4, 133.9 (d, *J* = 2.6 Hz), 133.0 (d, *J* = 17.2 Hz), 132.3 (d, *J* = 18.0 Hz), 130.7 (d, *J* = 94.8 Hz), 129.0 (d, *J* = 6.1 Hz), 128.6, 128.5 (d, *J* = 5.3 Hz), 128.2 (d, *J* = 3.3 Hz), 128.0 (d, *J* = 5.0 Hz), 127.8, 127.7 (d, *J* = 7.8 Hz), 127.5 (d, *J* = 4.2 Hz), 127.0, 126.6

(d, J = 10.3 Hz), 126.4 (d, J = 12.6 Hz), 126.0, 125.3, 123.5, 123.1 (d, J = 16.9 Hz), 122.1 (d, J = 10.7 Hz), 121.7, 121.5 (d, J = 15.0 Hz), 121.0, 120.9, 120.6, 46.2, 46.1, 28.0, 27.8, 27.3, 26.7. **^{31}P NMR (162 MHz, CDCl$_3$)** δ -15.3. ATR-FTIR (cm^{-1}): 3505, 2862, 1947, 1776, 1521, 1032, 991, 651. HRMS m/z (ESI): calcd for $C_{48}H_{40}P$ (M + H)$^+$ 647.2862, found 647.2863.

2.3 General Procedure for Arylation of Arylphosphines with Arylbromides through Three-Fold C–H Activation

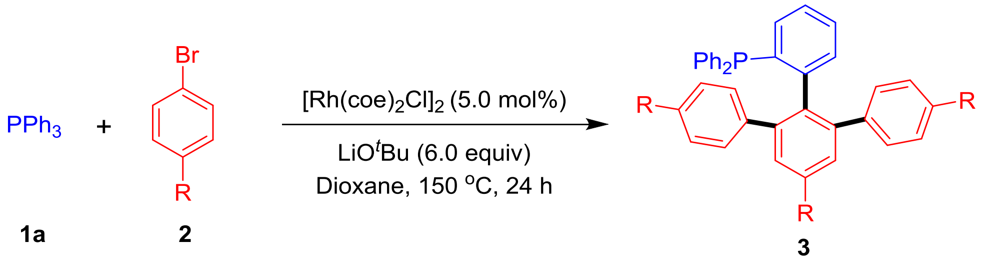

In an oven-dried Schlenk tube, arylphosphines **1a** (1.0 equiv, 0.20 mmol), arylbromides **2** (6.0 equiv, 1.2 mmol), [Rh(coe)$_2$Cl]$_2$ (5 mol%, 7.17 mg, 0.01 mmol), LiO^{t}Bu (6.0 equiv, 96 mg, 1.2 mmol) were dissolved in 1,4-dioxane (0.5 mL). The mixture was stirred at 150 °C under argon for 24 hours. Upon the completion of the reaction, the solvent was removed. The crude mixture was directly subjected to column chromatography on silica gel using petrol ether/EtOAc as eluent to give the desired products **3**.

Diphenyl(6'-phenyl-[1,1':2',1''-terphenyl]-2-yl)phosphane (3an)[2]

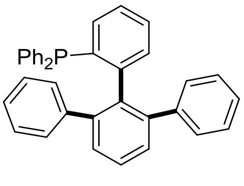

Following the general procedure, the reaction of **1a** (52.4 mg, 0.20 mmol), **2n** (186 mg, 1.2 mmol), [Rh(coe)$_2$Cl]$_2$ (5 mol%, 7.17 mg, 0.01 mmol), LiO^{t}Bu (6.0 equiv, 96 mg, 1.2 mmol), in 1,4-dioxane (0.5 mL) at 150 °C. After 24 h, purification by column chromatography on silica gel (petroleum-ether) yield **3an** (64.7 mg, 66%) as a white solid. **^{1}H NMR (500 MHz, Chloroform-*d*)** δ 7.52 (dd, J = 8.1, 7.2 Hz, 1H), 7.40 (d, J = 7.5 Hz, 2H), 7.22 (t, J = 7.3 Hz, 2H), 7.18 – 7.05 (m, 9H), 7.00 (t, J = 7.6 Hz, 5H), 6.93 – 6.88 (m, 4H), 6.75 – 6.66 (m, 4H). **^{13}C NMR (101 MHz,**

Chloroform-*d*) δ 146.3 (d, J = 33.6 Hz), 142.4 (d, J = 1.9 Hz), 141.9 (d, J = 0.9 Hz), 138.4 (d, J = 6.2 Hz), 137.9, 137.8, 136.8 (d, J = 12.8 Hz), 135.1 (d, J = 2.7 Hz), 133.5 (d, J = 19.5 Hz), 133.2 (d, J = 6.7 Hz), 130.3, 129.5, 128.0, 128.0, 127.9, 127.9, 127.6, 127.4, 126.9, 125.9. **^{31}P NMR (162 MHz, $CDCl_3$)** δ -14.6. ATR-FTIR (cm^{-1}): 3405, 3056, 2924, 1960, 1684, 1494, 811, 588. HRMS m/z (ESI): calcd for $C_{36}H_{40}P$ $(M + H)^+$ 503.2862, found 503.2862.

(4',4''-Dimethyl-6'-(p-tolyl)-[1,1':2',1''-terphenyl]-2-yl)diphenylphosphane (3ao)

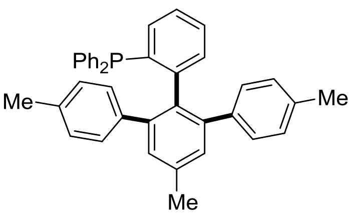

Following the general procedure, the reaction of **1a** (52.4 mg, 0.20 mmol), **2o** (205 mg, 1.2 mmol), $[Rh(coe)_2Cl]_2$ (5 mol%, 7.17 mg, 0.01 mmol), LiO^{t}Bu (6.0 equiv, 96 mg, 1.2 mmol), in 1,4-dioxane (0.5 mL) at 150 °C. After 24 h, purification by column chromatography on silica gel (petroleum-ether) yield **3ao** (62.8 mg, 59%) as a white solid; mp = 162−164 °C. **^{1}H NMR (500 MHz, Chloroform-*d*)** δ 7.19 (d, *J* = 7.2 Hz, 4H), 7.14 – 7.07 (m, 7H), 7.04 – 6.99 (m, 2H), 6.77 (d, *J* = 3.8 Hz, 7H), 6.74 – 6.70 (m, 4H), 2.46 (s, 3H), 2.23 (s, 6H). **^{13}C NMR (101 MHz, Chloroform-*d*)** δ 146.9, 146.7, 142.1 (d, *J* = 2.2 Hz), 139.1, 138.3, 138.2, 137.1, 136.8, 136.7, 135.7 (d, *J* = 6.0 Hz), 135.4, 135.3 (d, *J* = 2.9 Hz), 133.5, 133.4 (d, *J* = 6.8 Hz), 133.4, 130.23, 130.21, 128.1, 128.0, 127.9 (d, *J* = 6.3 Hz), 127.7, 126.7, 21.3, 21.0. **^{31}P NMR (162 MHz, $CDCl_3$)** δ -15.1. ATR-FTIR (cm^{-1}): 3502, 2974, 1866, 1731, 1630, 1294, 768, 571. HRMS m/z (ESI): calcd for $C_{39}H_{34}P$ $(M + H)^+$ 533.2393, found 533.2389.

(4',4''-Dimethoxy-6'-(4-methoxyphenyl)-[1,1':2',1''-terphenyl]-2-yl)diphenyl-phosphane (3ap)

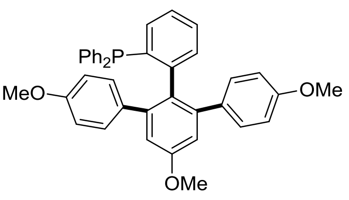

Following the general procedure, the reaction of **1a** (52.4 mg, 0.20 mmol), **2p** (222 mg, 1.2 mmol), $[Rh(coe)_2Cl]_2$ (5 mol%, 7.17 mg, 0.01 mmol), LiO^{t}Bu (6.0 equiv, 96 mg, 1.2 mmol), in 1,4-dioxane (0.5 mL) at 150 °C. After 24 h, purification by column chromatography on silica gel

(petroleum-ether: ethyl acetate = 20:1) yield **3ap** (84.7 mg, 73%) as a white solid; mp = 183−185 °C. **^{1}H NMR (500 MHz, Chloroform-*d*)** δ 7.19 – 7.07 (m, 8H), 7.03 – 6.97 (m, 2H), 6.90 (d, *J* = 1.7 Hz, 2H), 6.84 – 6.80 (m, 4H), 6.79 – 6.65 (m, 4H), 6.52 – 6.46 (m, 4H), 3.86 (s, 3H), 3.70 (s, 6H). **^{13}C NMR (101 MHz, Chloroform-*d*)** δ 158.4, 157.9, 146.5 (d, *J* = 33.9 Hz), 143.2 (d, *J* = 2.1 Hz), 138.2 (d, *J* = 14.4 Hz), 137.0 (d, *J* = 11.3 Hz), 135.2 (d, *J* = 3.0 Hz), 134.4, 133.6, 133.52, 133.51, 133.4 (d, *J* = 19.4 Hz), 131.4 (d, *J* = 6.3 Hz), 131.2, 128.0, 127.9, 127.8, 127.74, 127.66, 126.7, 114.5, 112.7, 55.2, 55.0. **^{31}P NMR (162 MHz, $CDCl_3$)** δ -14.8. ATR-FTIR (cm^{-1}): 3498, 2810, 1960, 1755, 1566, 1062, 916, 698. HRMS m/z (ESI): calcd for $C_{39}H_{34}O_3P$ $(M + H)^+$ 581.2240, found 581.2234.

(4',4''-Difluoro-6'-(4-fluorophenyl)-[1,1':2',1''-terphenyl]-2-yl)diphenylphos-phane (3aq)

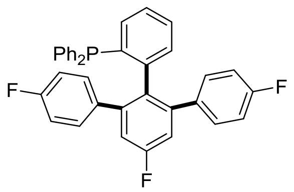

Following the general procedure, the reaction of **1a** (52.4 mg, 0.20 mmol), **2q** (210 mg, 1.2 mmol), $[Rh(coe)_2Cl]_2$ (5 mol%, 7.17 mg, 0.01 mmol), LiO^{t}Bu (6.0 equiv, 96 mg, 1.2 mmol), in 1,4-dioxane (0.5 mL) at 150 ^{o}C. After 24 h, purification by column chromatography on silica gel (petroleum-ether) yield **3aq** (56.6 mg, 52%) as a white solid; mp = 166−167 °C. **^{1}H NMR (500 MHz, Chloroform-*d*)** δ 7.24 – 7.20 (m, 2H), 7.20 – 7.02 (m, 10H), 6.83 – 6.78 (m, 4H), 6.76 – 6.69 (m, 4H), 6.69 – 6.62 (m, 4H). **^{13}C NMR (101 MHz, Chloroform-*d*)** δ 161.7 (d, *J* = 246.4 Hz), 161.6 (d, *J* = 246.8 Hz), 145.1, 144.9, 143.5 (d, *J* = 8.2 Hz), 137.4 (d, *J* = 13.3 Hz), 137.0 (d, *J* = 12.7 Hz), 136.7, 135.3 (d, *J* = 2.6 Hz), 133.4, 133.28 (d, *J* = 6.6 Hz), 133.27, 131.62, 131.55, 128.3, 128.22, 128.18 (d, *J* = 2.8 Hz), 127.4, 116.2, 116.1, 114.5, 114.3. **^{31}P NMR (162 MHz, $CDCl_3$)** δ -14.8. **^{19}F NMR (471 MHz, Chloroform-*d*) δ** -115.1, -116.0. ATR-FTIR (cm^{-1}): 3498, 2810, 1960, 1755, 1566, 1062, 916, 698. HRMS m/z (ESI): calcd for $C_{36}H_{25}F_3P$ $(M + H)^+$ 545.1640, found 545.1632.

(4',4''-Dichloro-6'-(4-chlorophenyl)-[1,1':2',1''-terphenyl]-2-yl)diphenylphosphane (3ar)

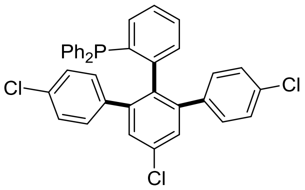

Following the general procedure, the reaction of **1a** (52.4 mg, 0.20 mmol), **2r** (229 mg, 1.2 mmol), $[Rh(coe)_2Cl]_2$ (5 mol%, 7.17 mg, 0.01 mmol), LiO^{t}Bu (6.0 equiv, 96 mg, 1.2 mmol), in 1,4-dioxane (0.5 mL) at 150 ^{o}C. After 24 h, purification by column chromatography on silica gel (petroleum-ether: ethyl acetate = 20:1) yield **3ar** (60.4 mg, 51%) as a white solid; mp = 191−193 °C. **^{1}H NMR (500 MHz, Chloroform-*d*)** δ 7.35 (s, 2H), 7.25 – 7.23 (m, 2H), 7.19 (td, *J* = 7.3, 1.4 Hz, 4H), 7.12 (td, *J* = 7.1, 1.4 Hz, 2H), 7.05 – 7.00 (m, 2H), 6.95 – 6.92 (m, 4H), 6.79 – 6.75 (m, 4H), 6.71 (td, *J* = 7.6, 1.2 Hz, 4H). **^{13}C NMR (101 MHz, Chloroform-*d*)** δ 144.7, 144.4, 142.9 (d, *J* = 2.2 Hz), 138.9, 137.2, 137.1, 137.0, 136.9, 136.8, 135.5 (d, *J* = 2.8 Hz), 133.4, 133.2, 133.0 (d, *J* = 6.6 Hz), 132.8, 131.3, 129.3, 128.5, 128.28, 128.25, 128.2, 127.8, 127.7. **^{31}P NMR (162 MHz, $CDCl_3$)** δ -14.9. ATR-FTIR (cm^{-1}): 3532, 2998, 1869, 1675, 1485, 1230, 776, 858. HRMS m/z (ESI): calcd for $C_{36}H_{25}Cl_3P$ $(M + H)^+$ 593.0754, found 593.0761.

(4',4''-Bis(trifluoromethyl)-6'-(4-(trifluoromethyl)phenyl)-[1,1':2',1''-terphenyl]-2-yl) diphenylphosphane (3as)

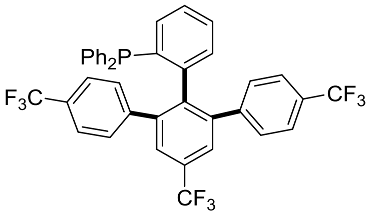

Following the general procedure, the reaction of **1a** (52.4 mg, 0.20 mmol), **2s** (269 mg, 1.2 mmol), $[Rh(coe)_2Cl]_2$ (5 mol%, 7.17 mg, 0.01 mmol), LiO^{t}Bu (6.0 equiv, 96 mg, 1.2 mmol), in 1,4-dioxane (0.5 mL) at 150 ^{o}C. After 24 h, purification by column chromatography on silica gel (petroleum-ether) yield **3as** (87.4 mg, 63%) as a white solid; mp = 144−145 °C. **^{1}H NMR (500 MHz, Chloroform-*d*)** δ 7.65 (s, 2H), 7.27 –7.24 (m, 6H), 7.19 – 7.14 (m, 6H), 7.07 – 7.02 (m, 2H), 6.98 (d, *J* = 8.1 Hz, 4H), 6.66 (t, *J* = 7.2 Hz, 4H). **^{13}C NMR (101 MHz, Chloroform-*d*)** δ 143.9, 143.8, 143.6, 142.1, 136.7, 136.6, 136.5, 135.5 (d, *J* = 2.7 Hz), 133.3, 133.2, 132.6 (d, *J* = 6.7 Hz), 130.4, 129.0 (d, *J* = 32.1 Hz), 128.6, 128.5, 128.3 (d, *J* = 6.5 Hz), 126.6 (d, *J* = 3.6 Hz), 124.6 (q, *J* = 3.6 Hz). **^{31}P NMR (162 MHz, $CDCl_3$)** δ -14.8 (dd, *J* = 20.6, 9.8 Hz). **^{19}F NMR (376 MHz,**

Chloroform-*d*) δ -62.4, -62.5. ATR-FTIR (cm^{-1}): 3421, 2632, 1755, 1614, 1597, 1030, 880, 612. HRMS m/z (ESI): calcd for $C_{39}H_{25}F_9P$ $(M + H)^+$ 695.1545, found 695.1533.

2,5-Diferrocenyl-1-(diphenylphosphino)ferrocene (3at)

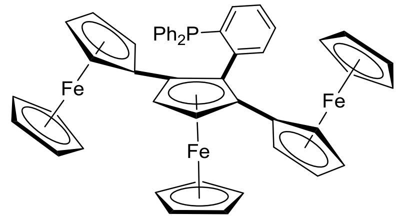

Following the general procedure, the reaction of **1a** (52.4 mg, 0.20 mmol), **2t** (317 mg, 1.2 mmol), $[Rh(coe)_2Cl]_2$ (10 mol%, 7.17 mg, 0.02 mmol), LiO^tBu (6.0 equiv, 96 mg, 1.2 mmol), in 1,4-dioxane (0.5 mL) at 150 °C. After 24 h, purification by column chromatography on silica gel (petroleum-ether) yield **3at** (42.3 mg, 26%) as a yellow solid; mp = 121−123 °C. **^{1}H NMR (500 MHz, Chloroform-*d*)** δ 8.10 – 8.04 (m, 1H), 7.29 (d, *J* = 7.2 Hz, 1H), 7.18 – 7.10 (m, 7H), 6.94 (ddd, *J* = 7.6, 3.6, 1.1 Hz, 1H), 6.87 – 6.81 (m, 4H), 4.69 (s, 2H), 4.37 (s, 5H), 4.20 – 4.17 (m, 2H), 4.02 (s, 10H), 3.98 (td, *J* = 2.5, 1.3 Hz, 2H), 3.71 (td, *J* = 2.4, 1.3 Hz, 2H), 3.59 (dt, *J* = 2.5, 1.2 Hz, 2H). **^{13}C NMR (101 MHz, Chloroform-*d*)** δ 142.6 (d, *J* = 33.5 Hz), 138.9 (d, *J* = 16.0 Hz), 138.1 (d, *J* = 14.1 Hz), 135.8 (d, *J* = 2.8 Hz), 135.3 (d, *J* = 6.4 Hz), 133.0 (d, *J* = 18.7 Hz), 128.3, 127.9 (d, *J* = 5.7 Hz), 127.5, 126.8, 86.1 (d, *J* = 2.3 Hz), 84.6 (d, *J* = 1.8 Hz), 71.4 (d, *J* = 3.6 Hz), 71.3, 70.5, 68.9, 68.4, 66.9 (d, J = 17.8 Hz). **^{31}P NMR (162 MHz, $CDCl_3$)** δ -16.1. ATR-FTIR (cm^{-1}): 3530, 2947, 2903, 2774, 1650, 1563, 1210, 1023, 893. HRMS m/z (ESI): calcd for $C_{48}H_{40}Fe_3P$ $(M + H)^+$ 815.0910, found 815.0864.

(2-(3-methoxy-1-(p-tolyl)naphthalen-2-yl)phenyl)diphenylphosphane (3afo)

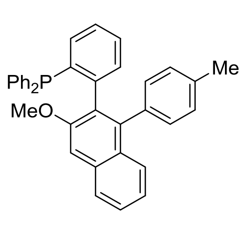

The reaction of **3af** (41.8 mg, 0.10 mmol), 4-Bromotoluene (51.3 mg, 0.30 mmol), $[Rh(coe)_2Cl]_2$ (5 mol%, 2.5 mg, 0.005 mmol), K_2CO_3 (5.0 equiv, 69 mg, 0.5 mmol), in 1,4-dioxane (0.25 mL) at 150 °C. After 36 h, purification by column chromatography on silica gel (petroleum-ether: ethyl acetate = 50:1) yield **3afo** (31.1 mg, 61%) as a white soild; mp = 206−208 °C. **^{1}H NMR (500 MHz, Chloroform-*d*)** δ 7.84 (dd, *J* = 8.2, 1.2 Hz, 1H), 7.51 – 7.45 (m, 2H), 7.35 – 7.25 (m, 10H), 7.20 – 7.07 (m, 8H), 7.02 (dd, *J* = 7.7, 1.9 Hz, 1H), 6.92 (dd, *J* = 7.9, 1.8 Hz, 1H), 3.40 (s, 3H), 2.35 (s, 3H). **^{13}C NMR**

(101 MHz, Chloroform-*d*) δ 155.6, 144.5 (d, *J* = 35.7 Hz), 140.0, 138.7 (d, *J* = 13.2 Hz), 138.1 (d, *J* = 12.8 Hz), 137.1 (d, *J* = 9.8 Hz), 136.0, 135.7, 134.6 (d, *J* = 2.8 Hz), 134.5, 133.7 (d, *J* = 19.7 Hz), 133.3, 133.2, 131.8 (d, *J* = 7.0 Hz), 131.0, 130.6 (d, *J* = 3.2 Hz), 128.7, 128.5, 128.3, 128.2, 128.1 (d, *J* = 6.9 Hz), 127.9 (d, *J* = 12.8 Hz), 127.1, 127.0, 126.7, 126.1, 123.5, 104.2, 54.7, 21.3. **^{31}P NMR (162 MHz, $CDCl_3$)** δ -14.6. ATR-FTIR (cm^{-1}): 3024, 2954, 1755, 1630, 1450, 1023, 965, 842. HRMS m/z (ESI): calcd for $C_{36}H_{30}OP$ $(M + H)^+$ 509.2029, found 509.2023.

2.4 Mechanistic Experiments

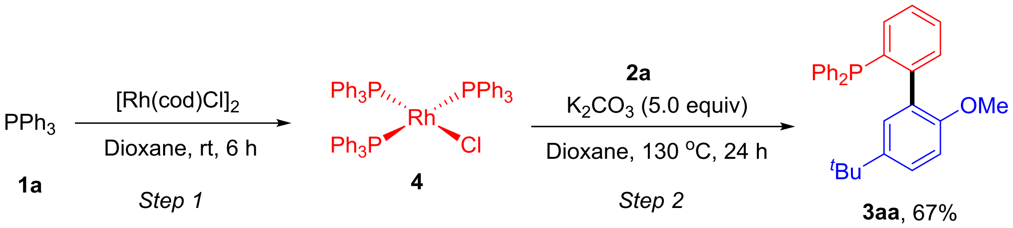

Step 1: Triphenylphosphine **1a** (78.6 mg, 0.3 mmol) was dissolved in 1,4-dioxane (0.5 mL). Then $[Rh(cod)Cl]_2$ (24.5 mg, 0.05 mmol) was added. The solution was stirred at room temperature for 6 h. The solvent was removed under reduced pressure and the crude product **4** was characterized by ^{31}P NMR. **^{31}P NMR (162 MHz, $CDCl_3$)** δ 29.2.

Step 2: the reaction of **4**, aryl bromide **2a** (72.5 mg, 0.3 mmol), K_2CO_3 (5.0 equiv, 69 mg, 0.5 mmol), in 1,4-dioxane (0.2 mL) at 130 °C. After 24 h, purification by column chromatography on silica gel (petroleum-ether: ethyl acetate = 200:1) yield **3aa** (28.4 mg, 67%).

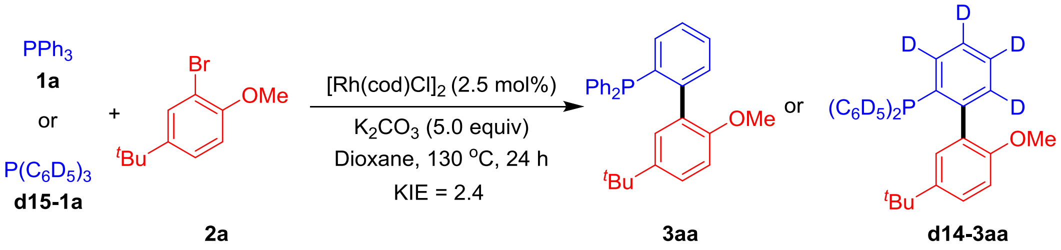

To a flame dried Schlenk tube was added substrates **1a** or **d15-1a** (0.2 mmol), $[Rh(cod)Cl]_2$ (2.5 mol%, 2.5 mg, 0.005 mmol), K_2CO_3 (5.0 equiv, 138 mg, 1.0 mmol),

and 1,4-dioxane (0.3 mL) was added to the Schlenk tube under Ar atmosphere. Then **2a** (145.2 mg, 0.6 mmol) was added to the reaction mixture and stirred at 130 °C for the indicated time (five parallel runs). After cooled to room temperature it was passed through a short pad of silica and the filtrate was concentrated in vacuo. The yield was determined by ^{31}P NMR analysis of the crude product using triphenyl phosphite as the internal standard. For **1a**, y = 5.5x + 0.3, R^2 = 0.986; for **d15-1a**, y = 2.3x + 0.9, R^2 = 0.9796. KIE value (2.4) was determined by comparing the relative initial rates.

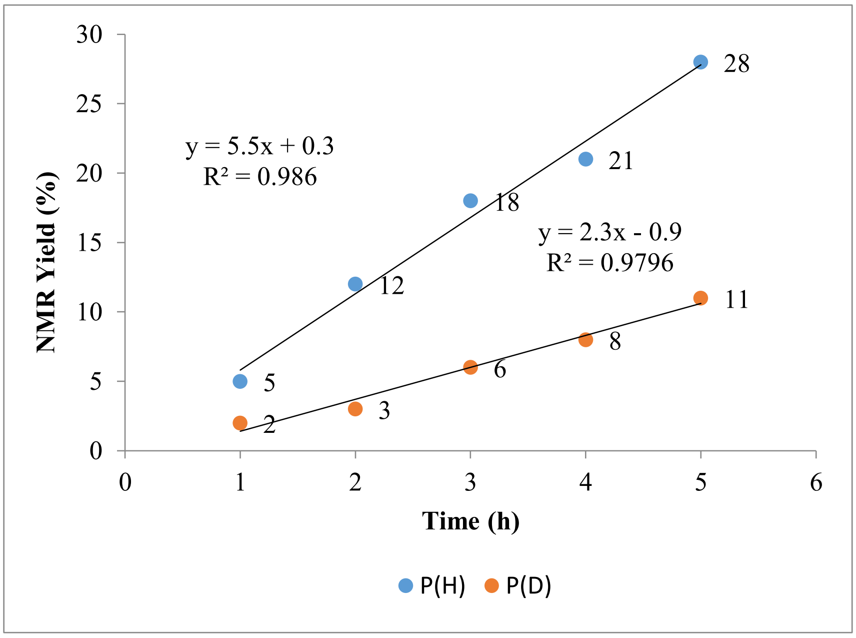

2.5 Computational Details

The detailed mechanism of the rhodium-catalyzed tunable direct arylation of arylphosphines was carried out using the Gaussian 09 program package[3]. The geometries of intermediates and transition states were optimized at the B3LYP[4] level of theory with a mixed basis set of LANL2DZ[5] for Rh atom and 6-31G(d)[6] for all other atoms in gas phase. Vibrational frequencies calculation was performed at the same level to confirm that the optimized structure was either a local minimum or a transition state, and to obtain all the thermal corrections to free energies at 298.15 K and 1 atm pressure. Single-point solvation energies were calculated with M06[7] functional and a mixed basis set of SDD[8] for Rh and 6-311++G(d,p)[9] for all other

atoms with continuum model SMD[10] (solvent = 1,4-dioxane). All energies are reported here in kcal/mol. The calculated optimized structures are visualized using CYLview.[11]

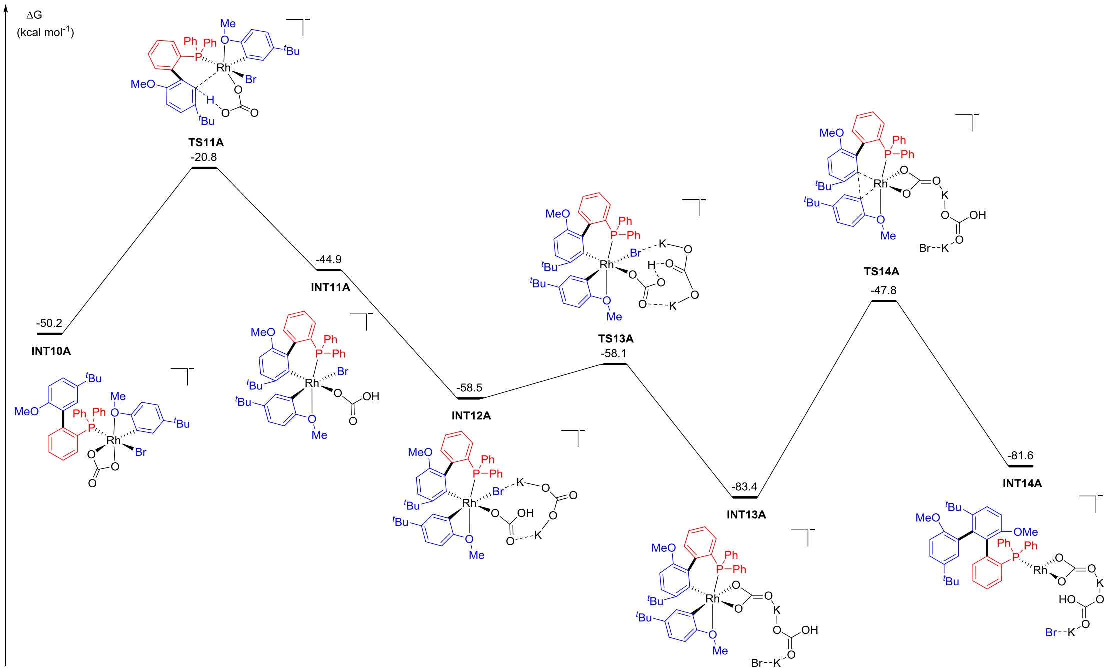

Supplementary Figure 1. DFT-calculated energy profile for the second-fold direct arylation of PPh_3 with **2b.**

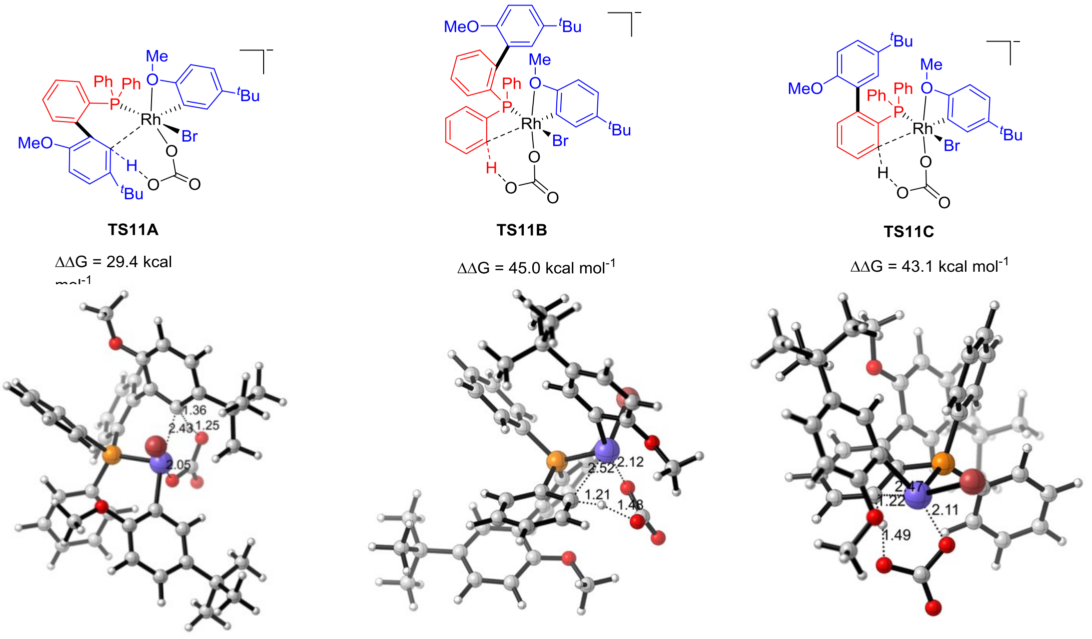

Supplementary Figure 2. DFT-computed free energies for the three competitive C-H metalation pathways in the second-fold direct arylation of PPh_3 with **2b.** Bond lengths are in Å.

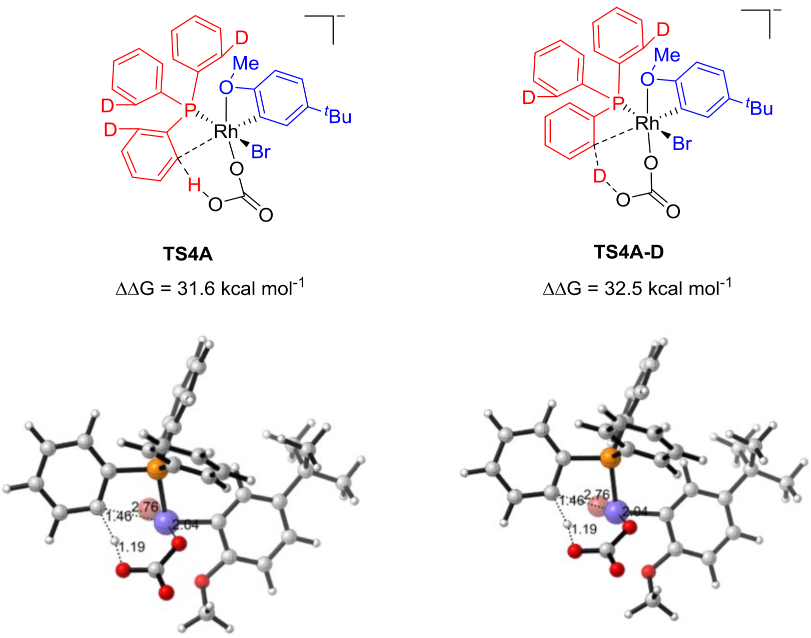

Supplementary Figure 3. The calculated transition state **TS4A** and the corresponding deuterated transition state **TS4A-D**. Bond lengths are in Å.

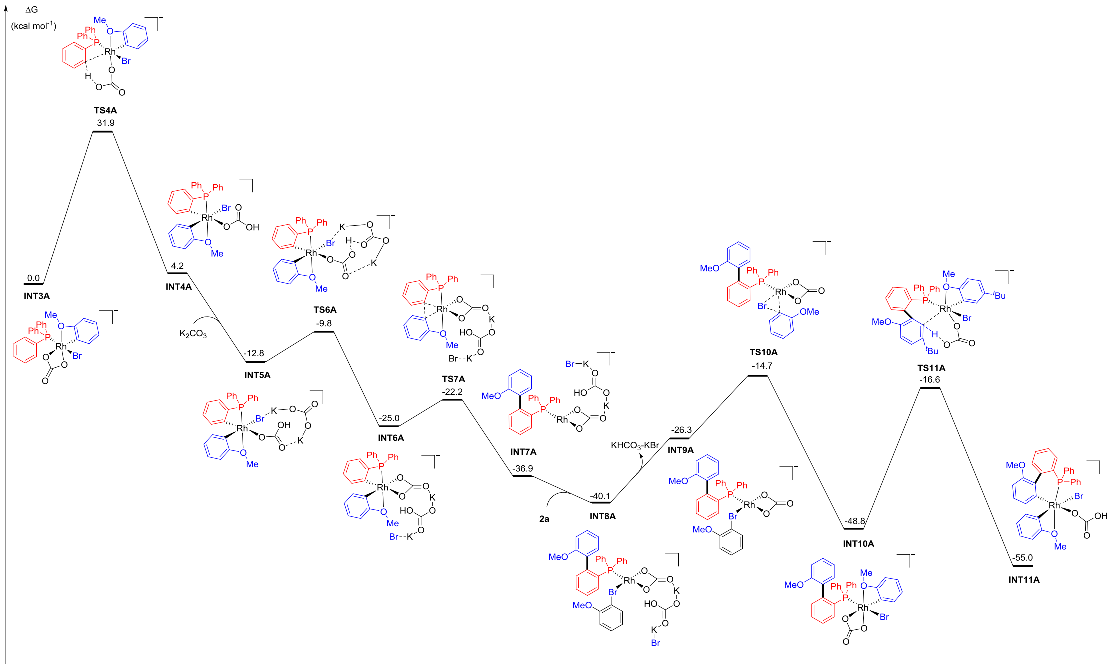

Supplementary Figure 4. DFT-calculated energy profile for the two-fold direct

arylation of PPh_3 with **2j** (Part 1).

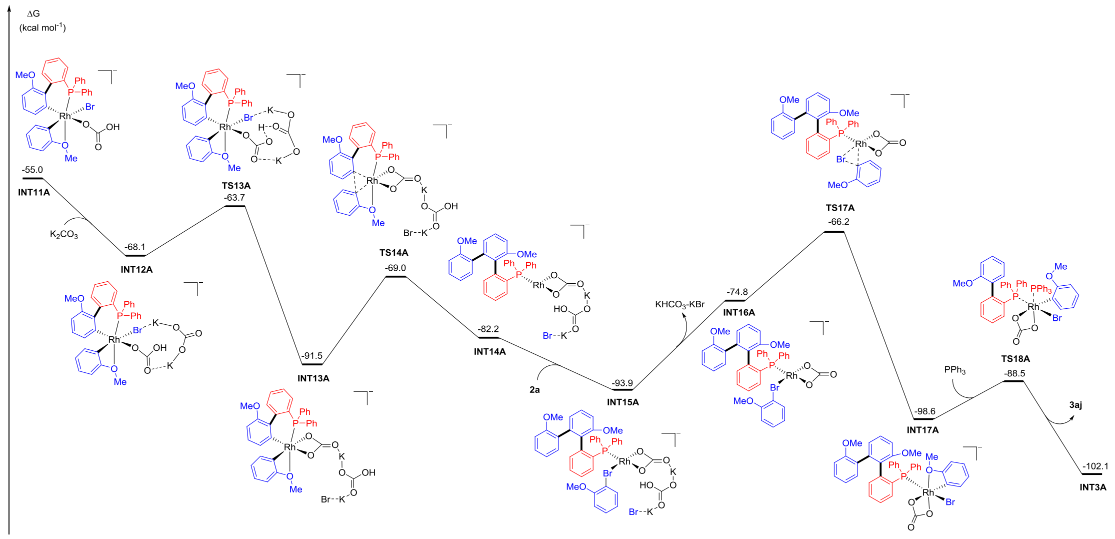

Supplementary Figure 5. DFT-calculated energy profile for the two-fold direct arylation of PPh_3 with **2j** (Part 2).

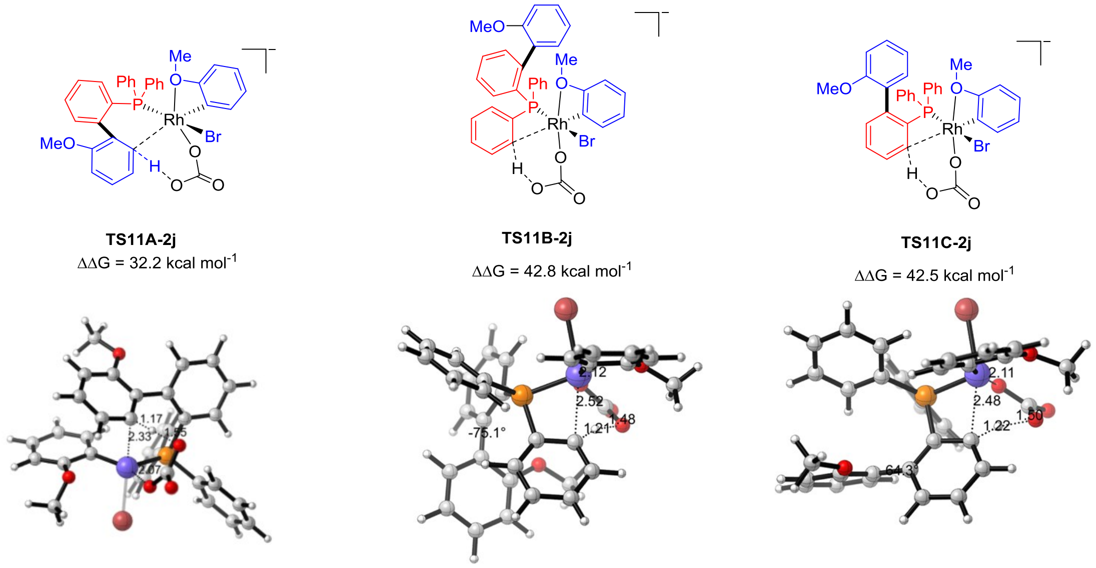

Supplementary Figure 6. DFT-computed free energies for the three competitive C-H metalation pathways in the second-fold direct arylation of PPh_3 with **2j.** Bond lengths are in Å.

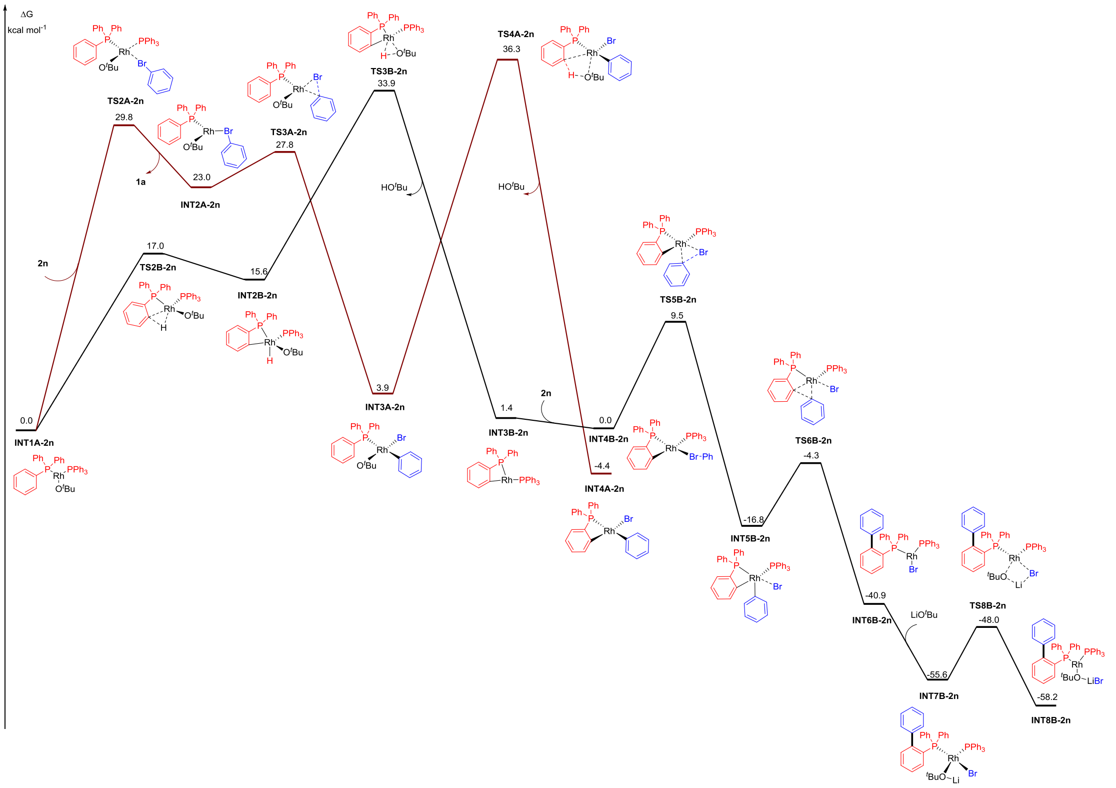

Supplementary Figure 7. DFT-calculated energy profile for the three-fold direct arylation of PPh_3 with **2n** (Part 1).

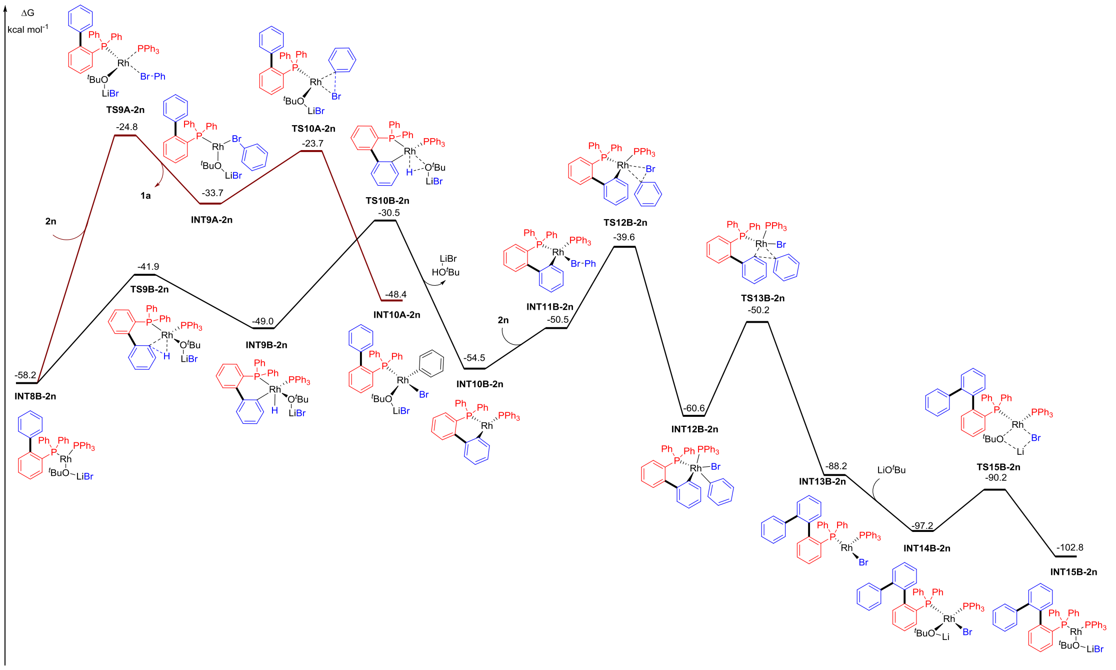

Supplementary Figure 8. DFT-calculated energy profile for the three-fold direct arylation of PPh_3 with **2n** (Part 2).

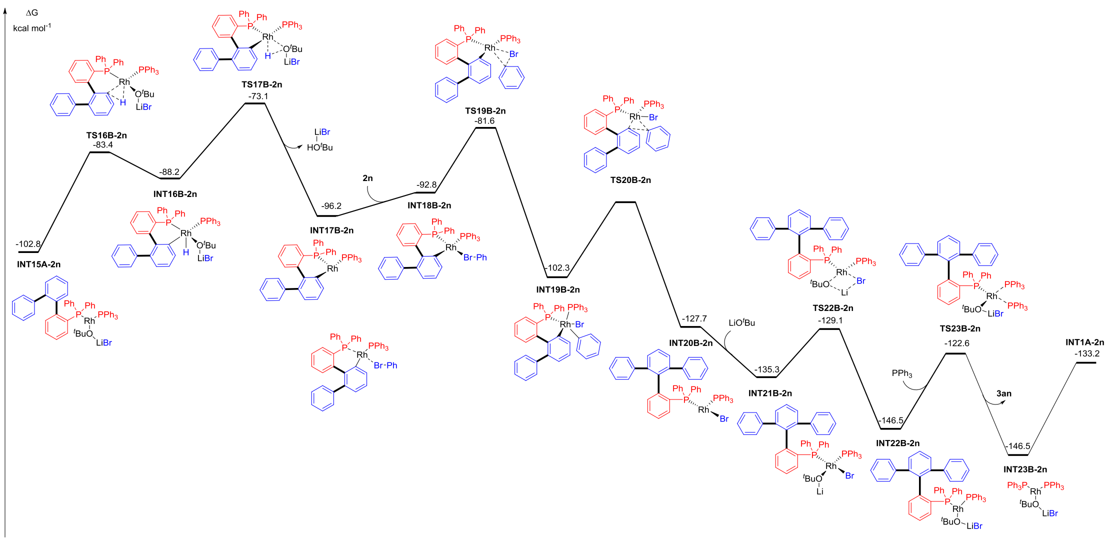

Supplementary Figure 9. DFT-calculated energy profile for the third-fold direct arylation of PPh_3 with **2n** (Part 3).

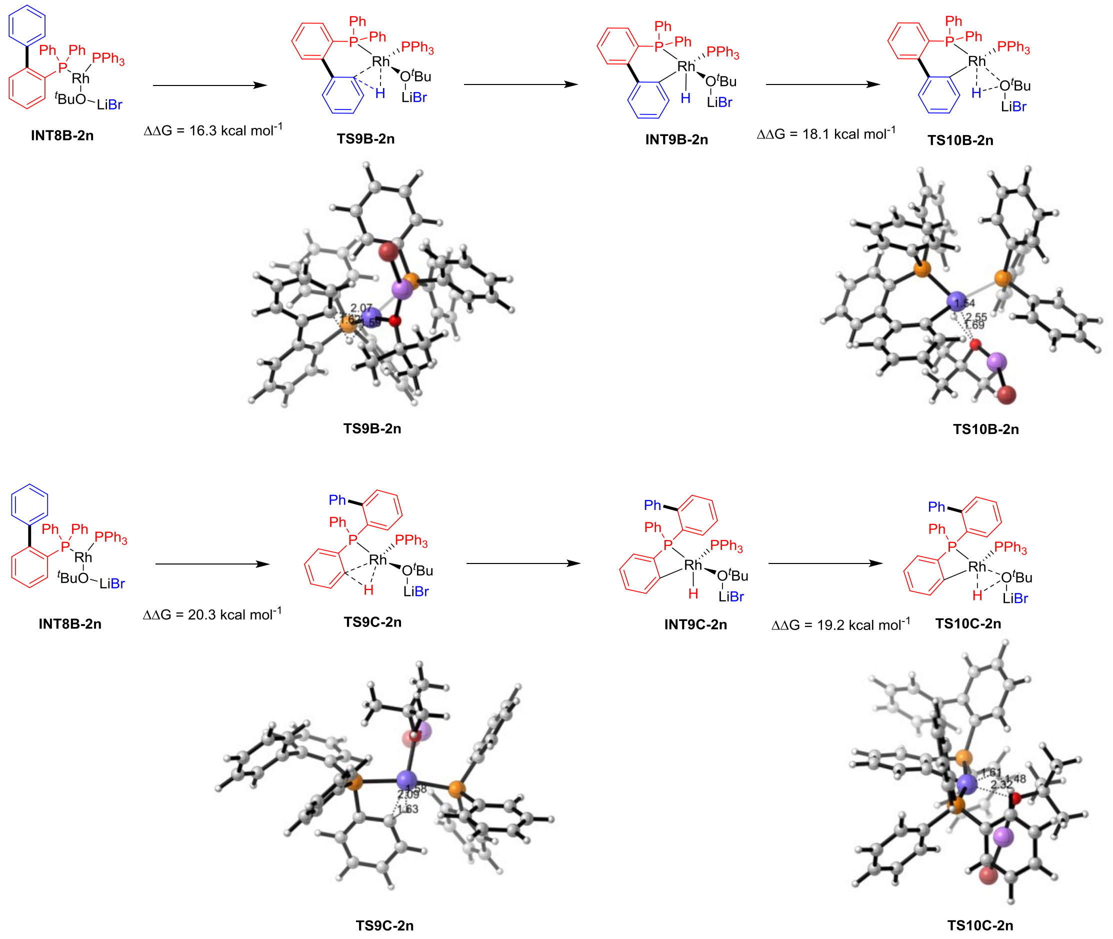

Supplementary Figure 10 DFT-computed free energies for the two competitive C-H oxidative addition pathways in the second-fold direct arylation of PPh_3 with **2n.**

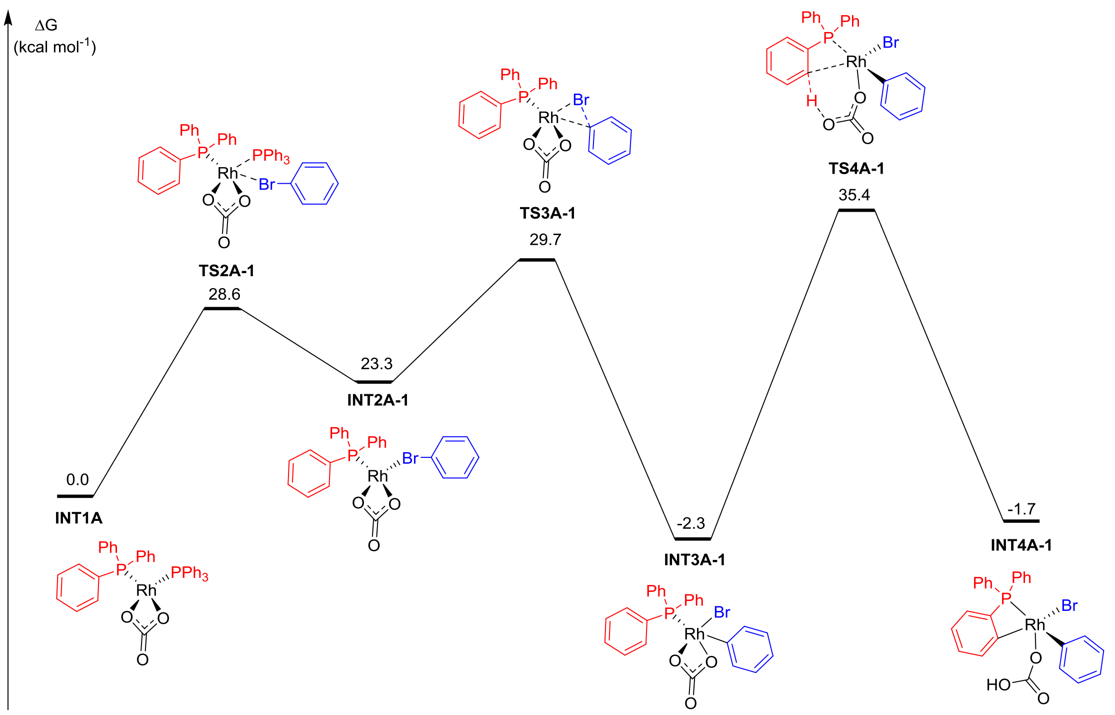

Supplementary Figure 11 CO_3^{2-} promoted C-H activation process in three-fold direct arylation of PPh_3 with **2n.**

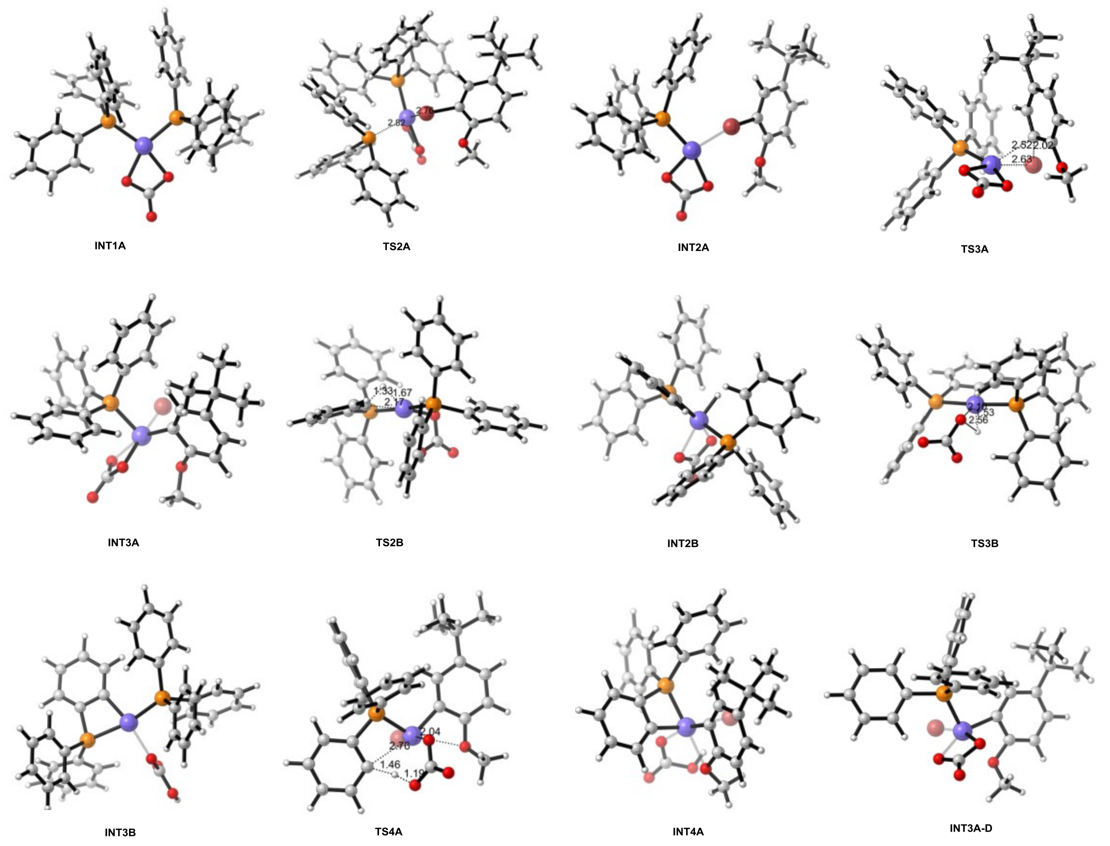

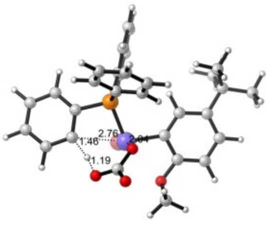

TS4A-D

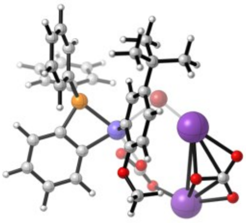

INT5A

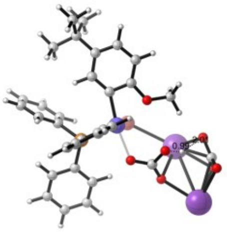

TS6A

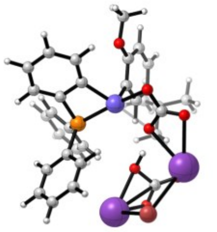

INT6A

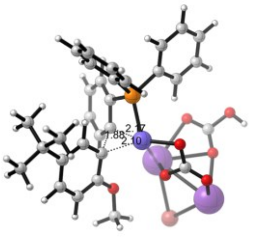

TS7A

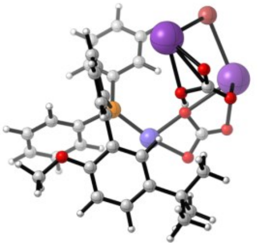

INT7A

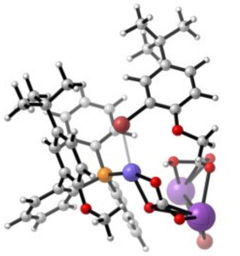

INT8A

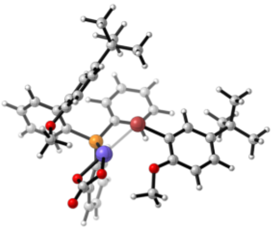

INT9A

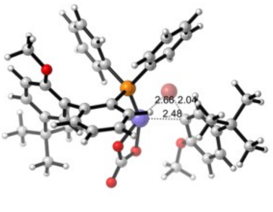

TS10A

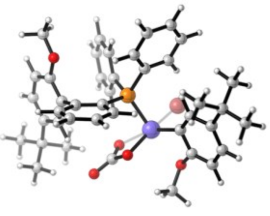

INT10A

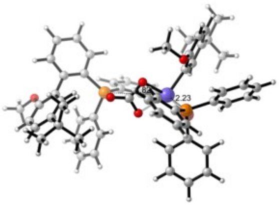

TS11

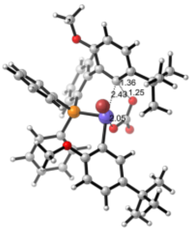

TS11A

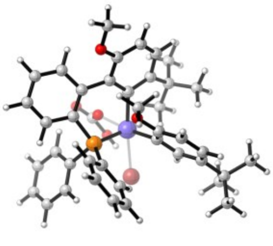

INT11A

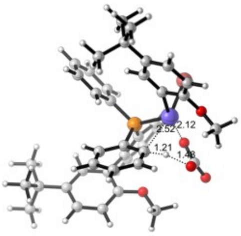

TS11B

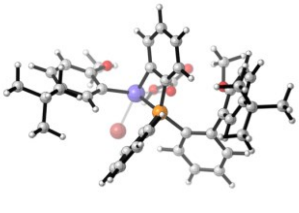

INT11B

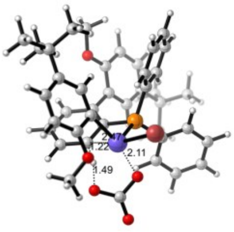

TS11C

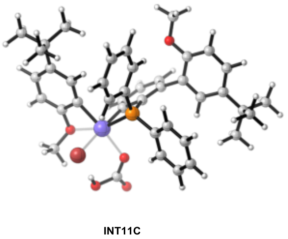

INT11C

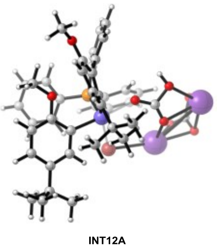

INT12A

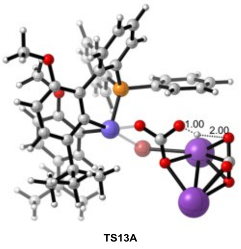

TS13A

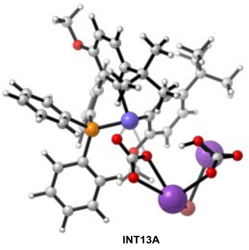

INT13A

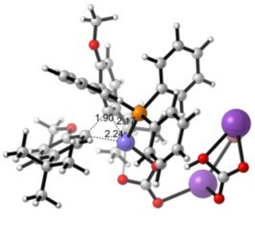

TS14A

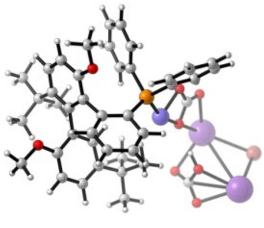

INT14A

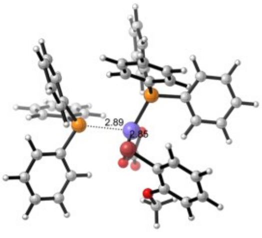

TS2A-2j

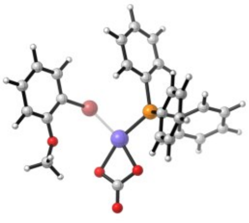

INT2A-2j

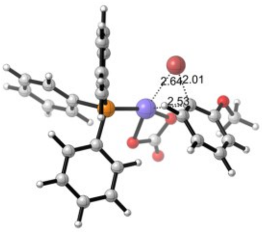

TS3A-2j

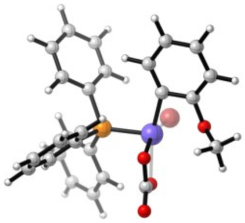

INT3A-2j

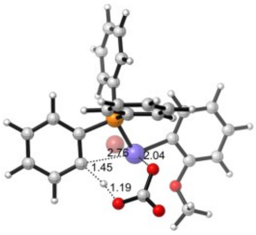

TS4A-2j

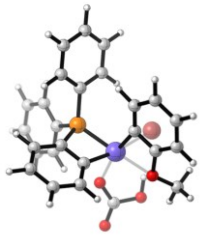

INT4A-2j

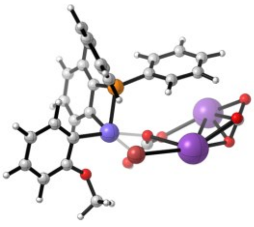

INT5A-2j

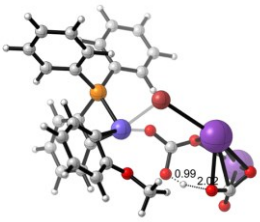

TS6A-2j

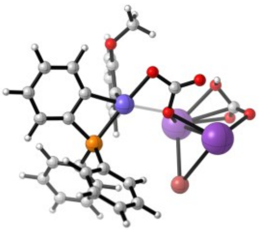

INT6A-2j

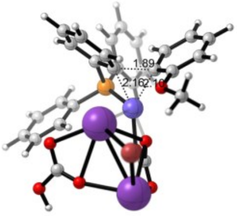

TS7A-2j

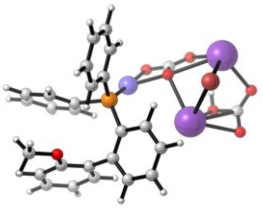

INT7A-2j

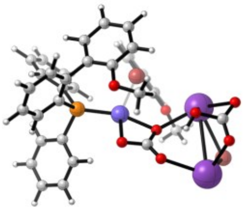

INT8A-2j

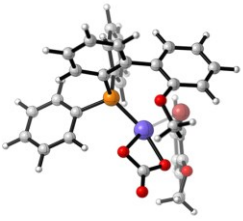

INT9A-2j

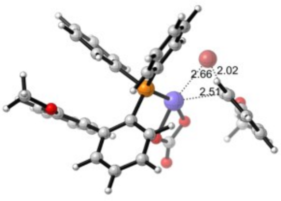

TS10A-2j

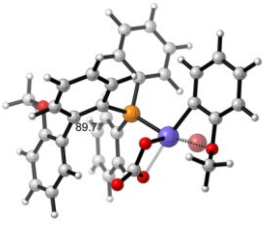

INT10A-2j

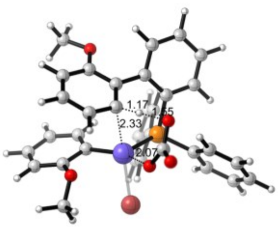

TS11A-2j

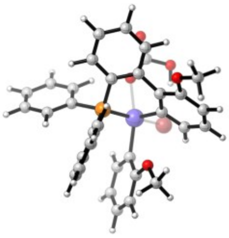

INT11A-2j

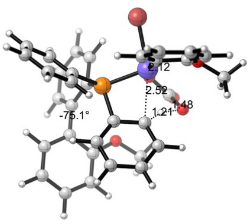

TS11B-2j

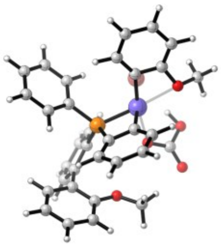

INT11B-2j

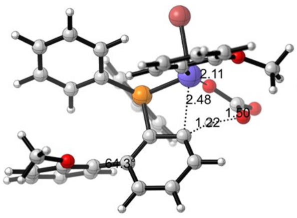

TS11C-2j

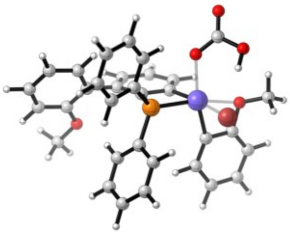

INT11C-2j

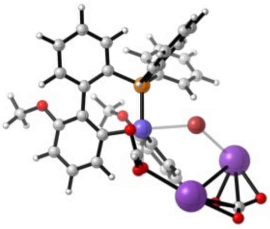

INT12A-2j

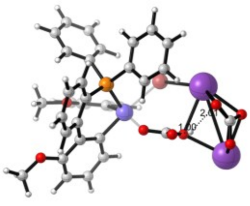

TS13A-2j

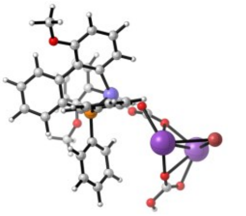

INT13A-2j

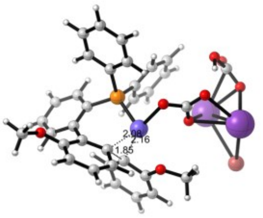

TS14A-2j

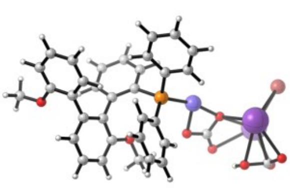

INT14A-2j

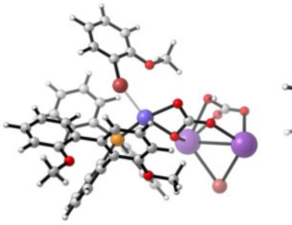

INT15A-2j

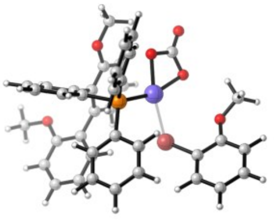

INT16A-2j

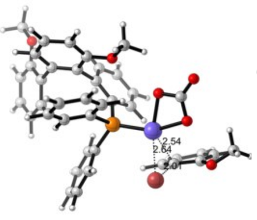

TS17A-2j

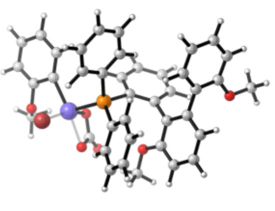

INT17A-2j

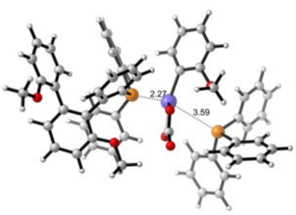

TS18-2j

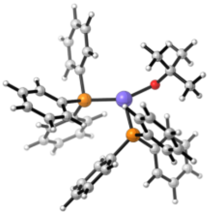

INT1A-2n

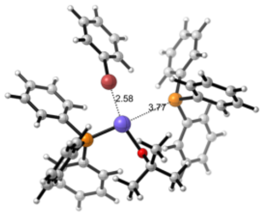

TS2A-2n

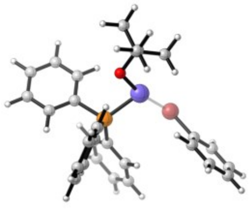

INT2A-2n

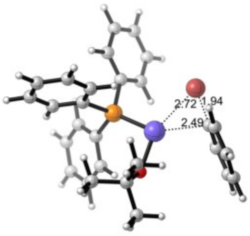

TS3A-2n

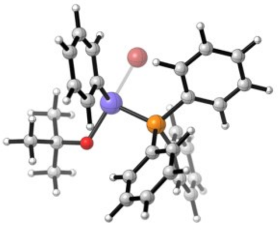

INT3A-2n

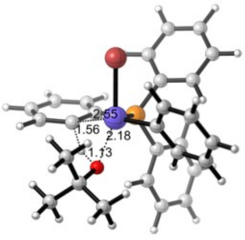

TS4A-2n

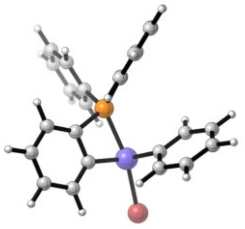

INT4A-2n

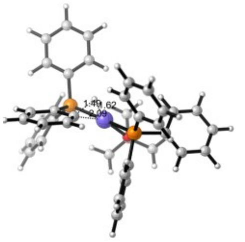

TS2B-2n

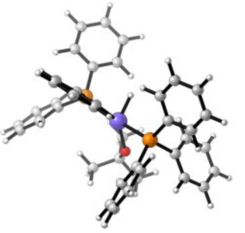

INT2B-2n

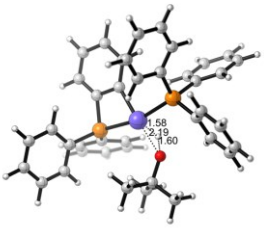

TS3B-2n

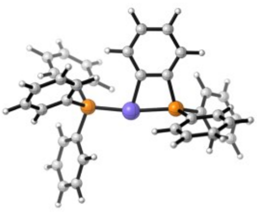

INT3B-2n

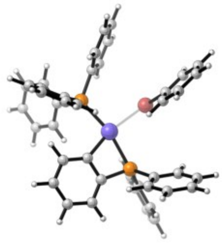

INT4B-2n

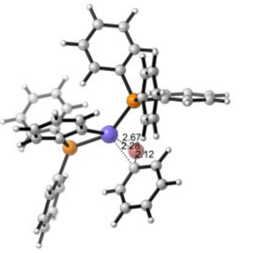

TS5B-2n

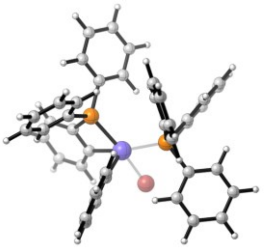

INT5B-2n

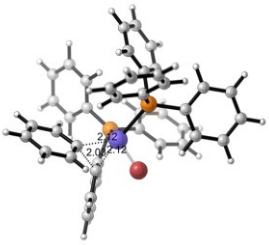

TS6B-2n

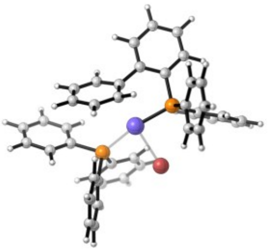

INT6B-2n

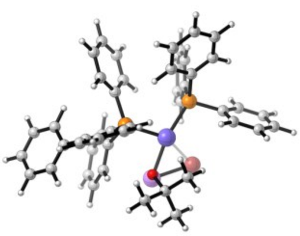

INT7B-2n

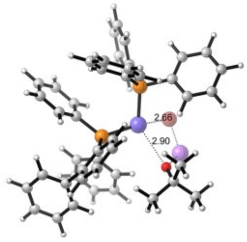

TS8B-2n

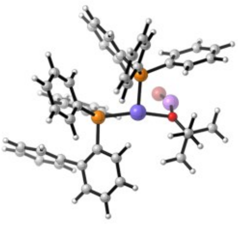

INT8B-2n

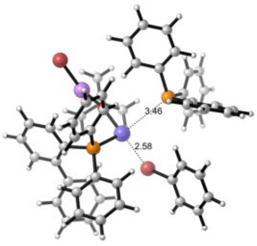

TS9A-2n

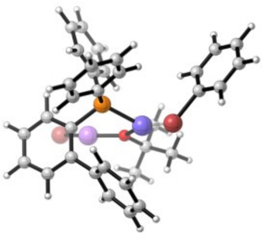

INT9A-2n

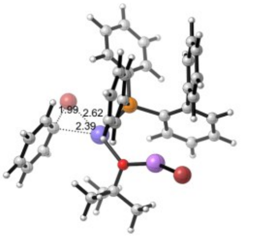

TS10A-2n

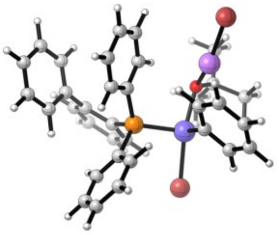

INT10A-2n

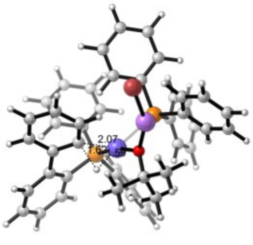

TS9B-2n

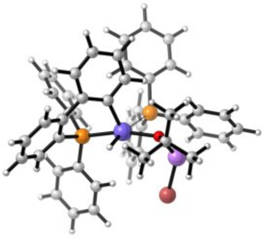

INT9B-2n

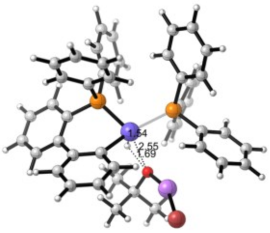

TS10B-2n

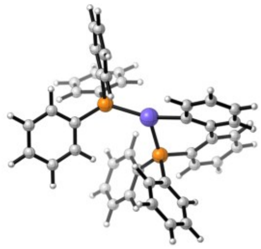

INT10B-2n

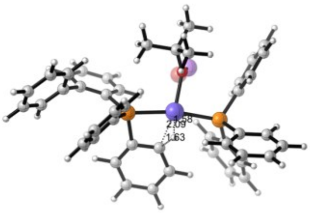

TS9C-2n

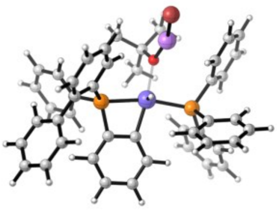

INT9C-2n

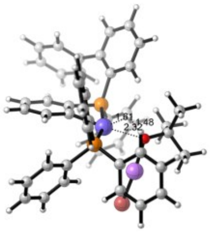

TS10C-2n

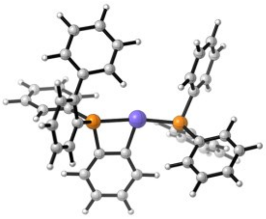

INT10C-2n

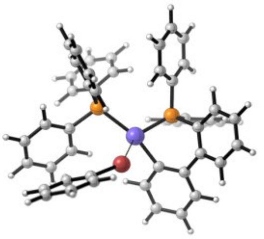

INT11B-2n

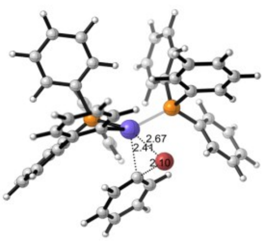

TS12B-2n

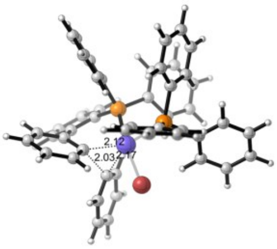

INT12B-2n

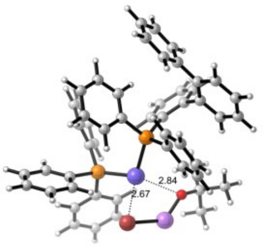

TS13B-2n

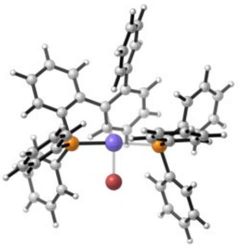

INT13B-2n

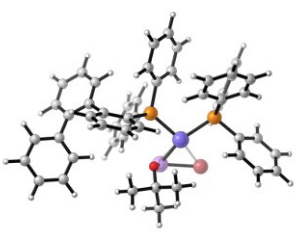

INT14B-2n

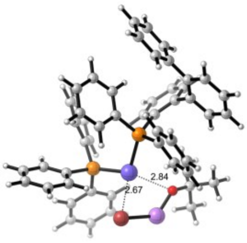

TS15B-2n

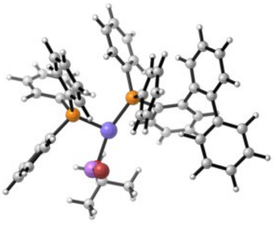

INT15B-2n

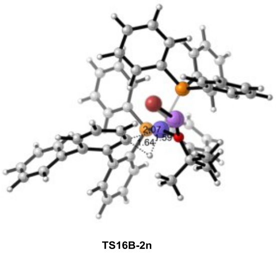

TS16B-2n

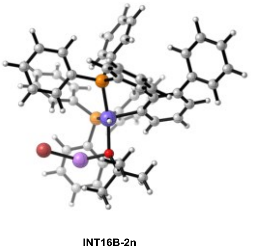

INT16B-2n

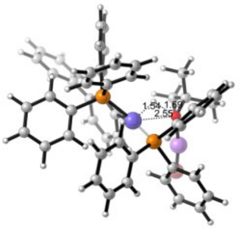

TS17B-2n

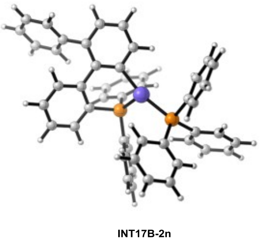

INT17B-2n

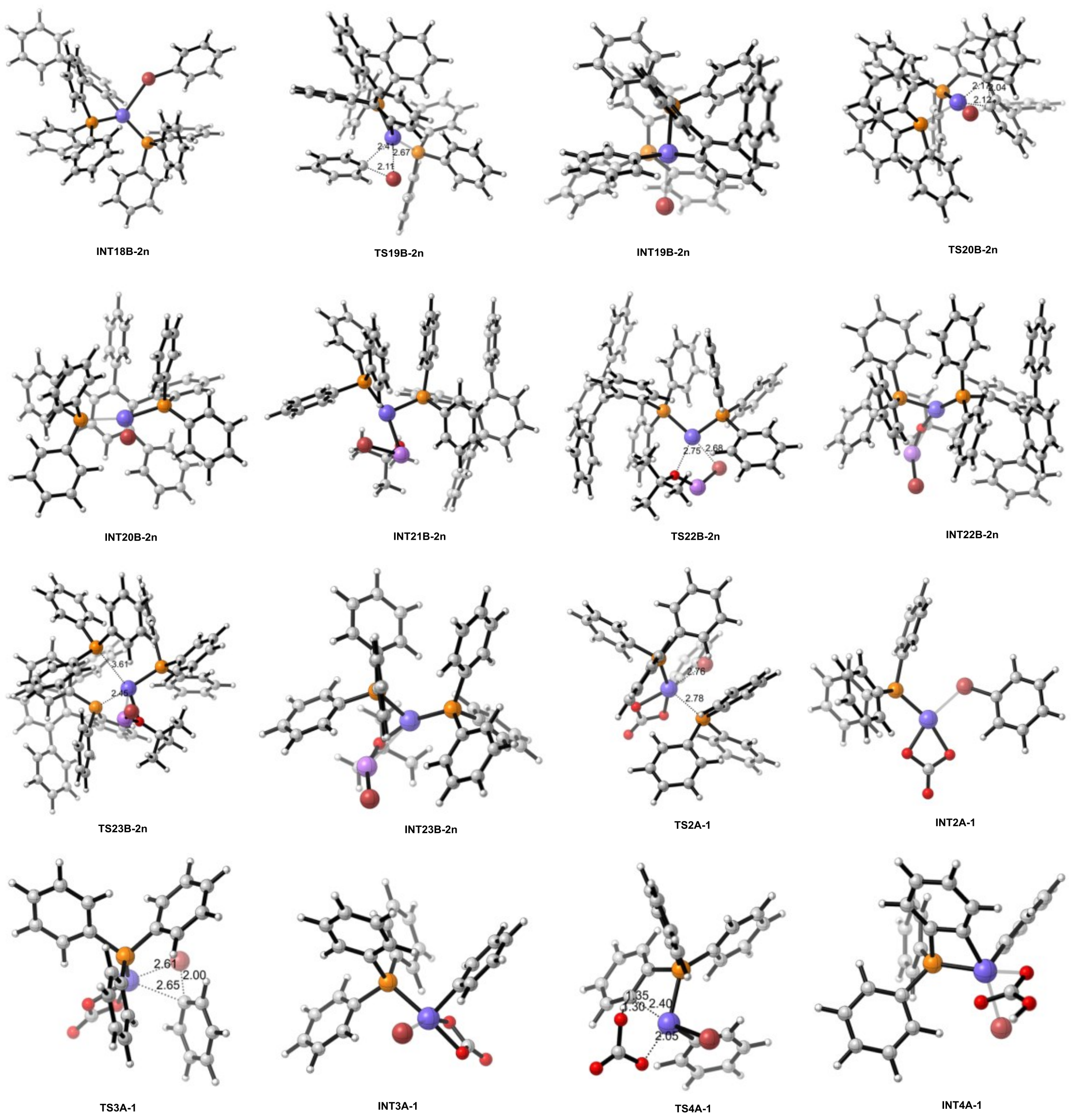

Supplementary Figure 12. The calculated optimized structures of the key intermediates and transition states. Distances are in Å.

2.6 NMR spectroscopic data

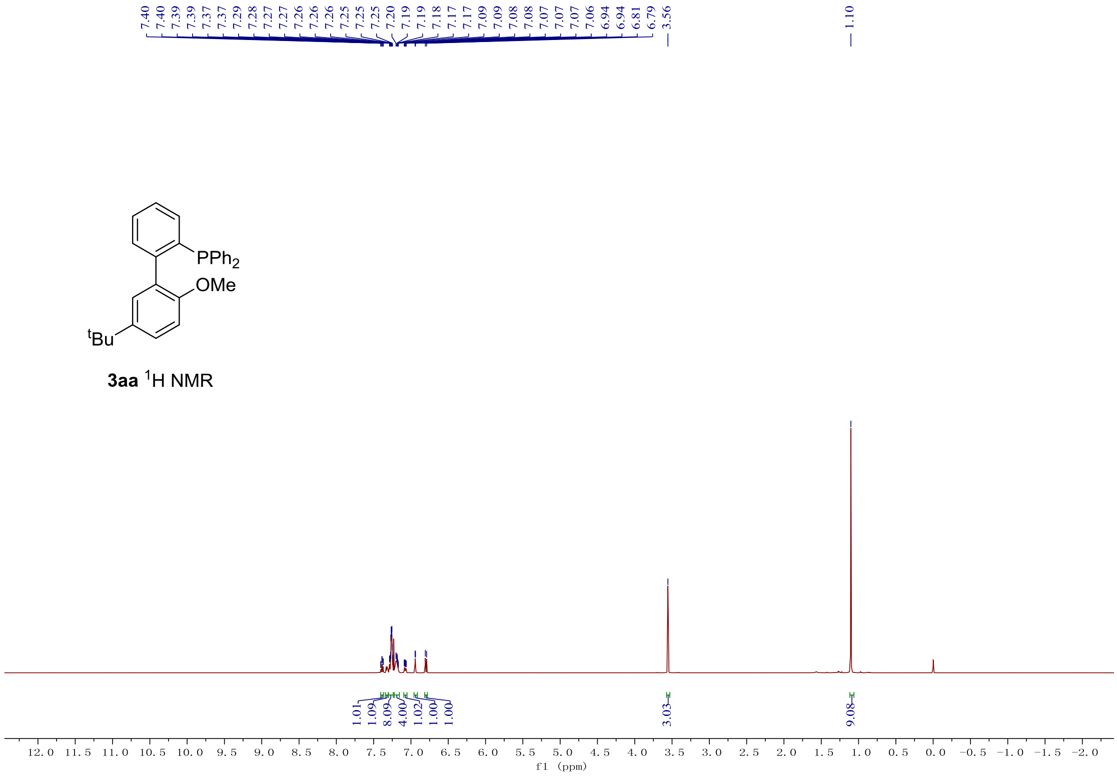

Supplementary Figure 12. ^{1}H NMR spectrum of **3aa**

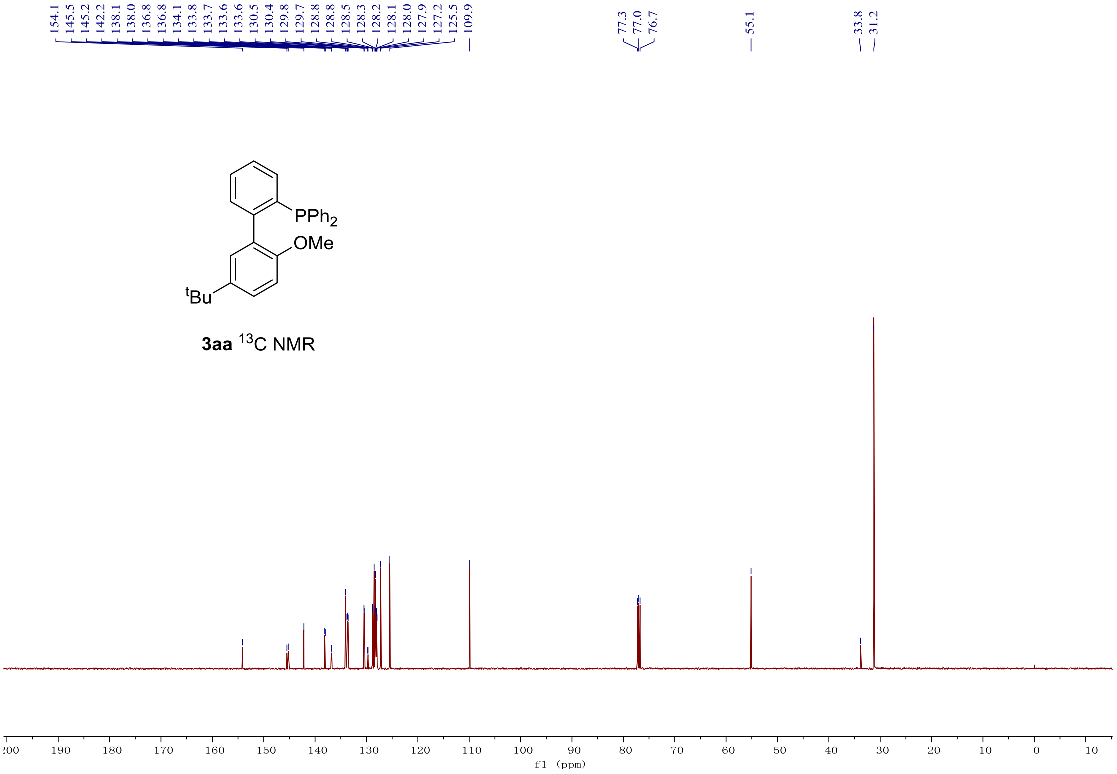

Supplementary Figure 13. ^{13}C NMR spectrum of **3aa**

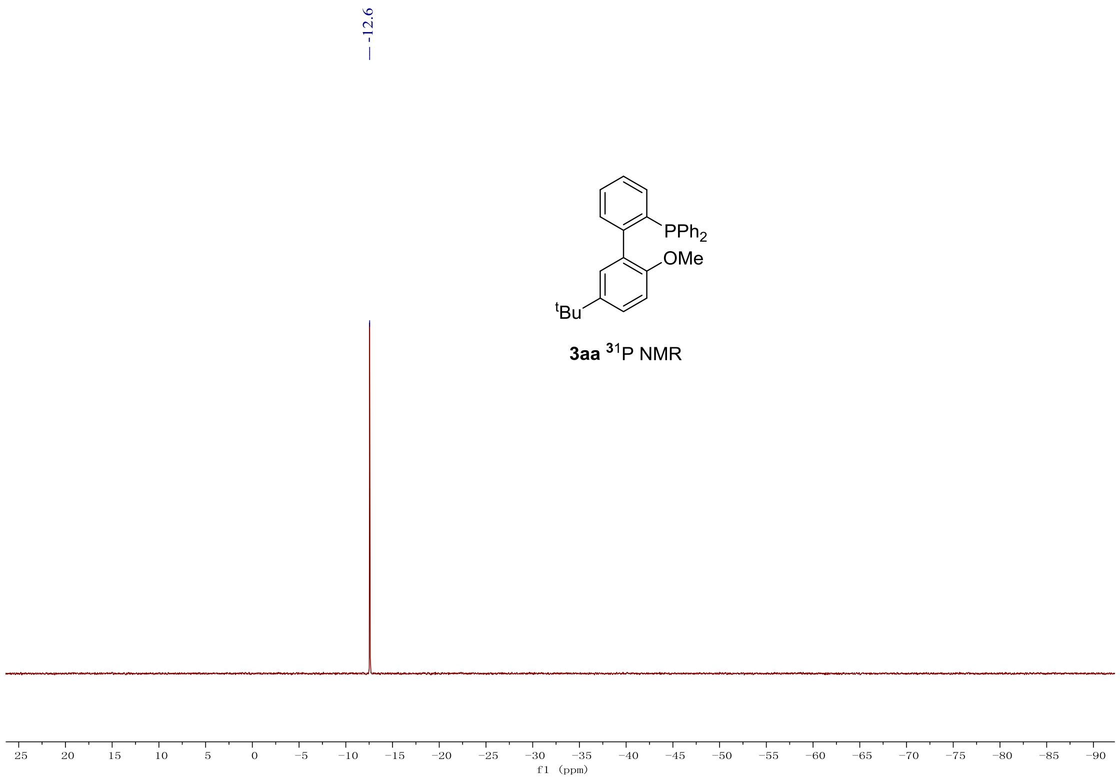

Supplementary Figure 14. ^{31}P NMR spectrum of **3aa**

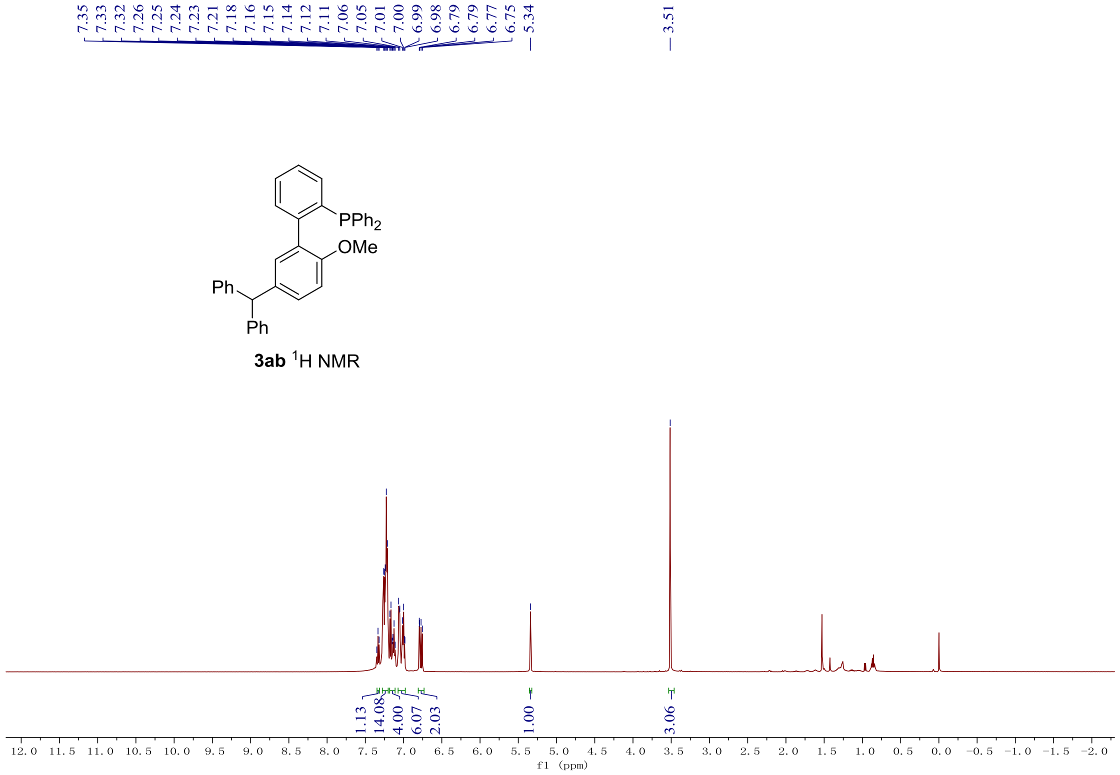

Supplementary Figure 15. ^{1}H NMR spectrum of **3ab**

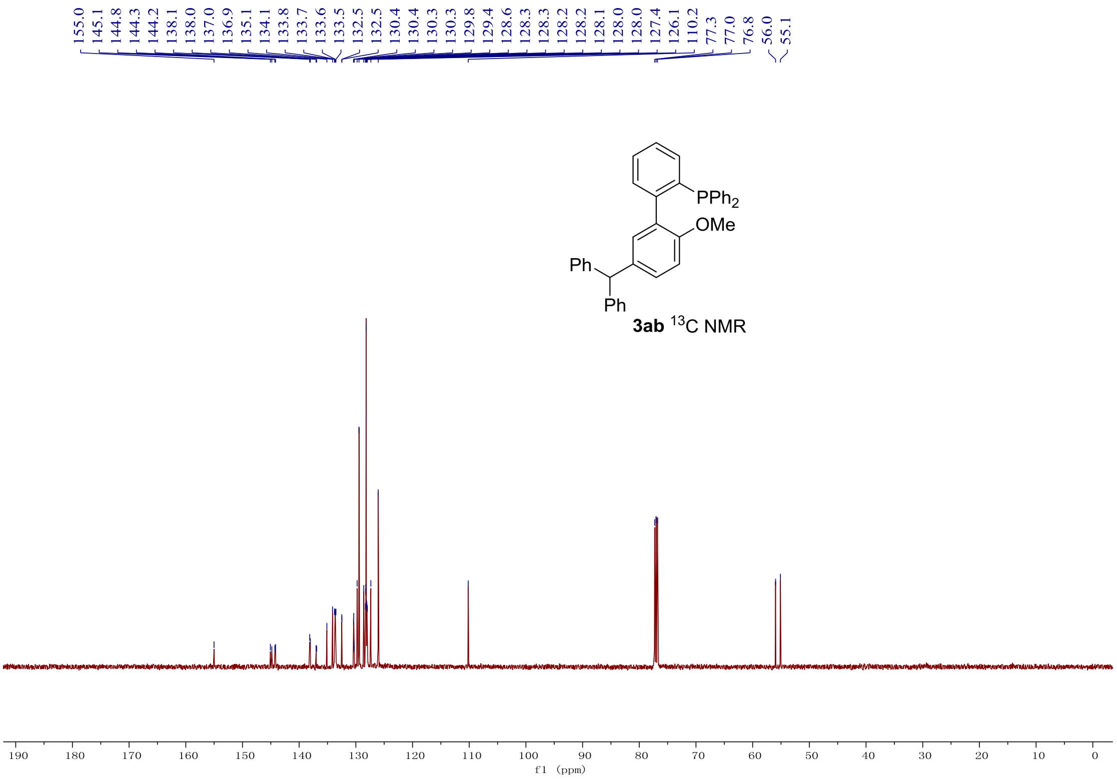

Supplementary Figure 16. ^{13}C NMR spectrum of **3ab**

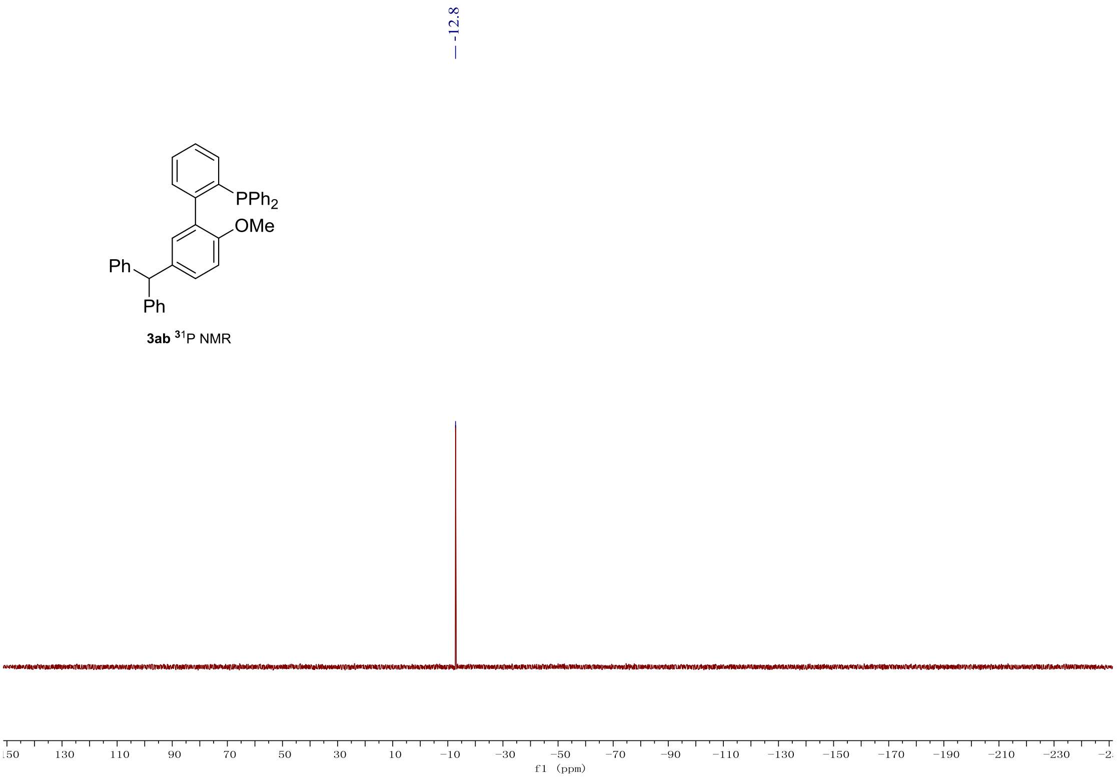

Supplementary Figure 17. ^{31}P NMR spectrum of **3ab**

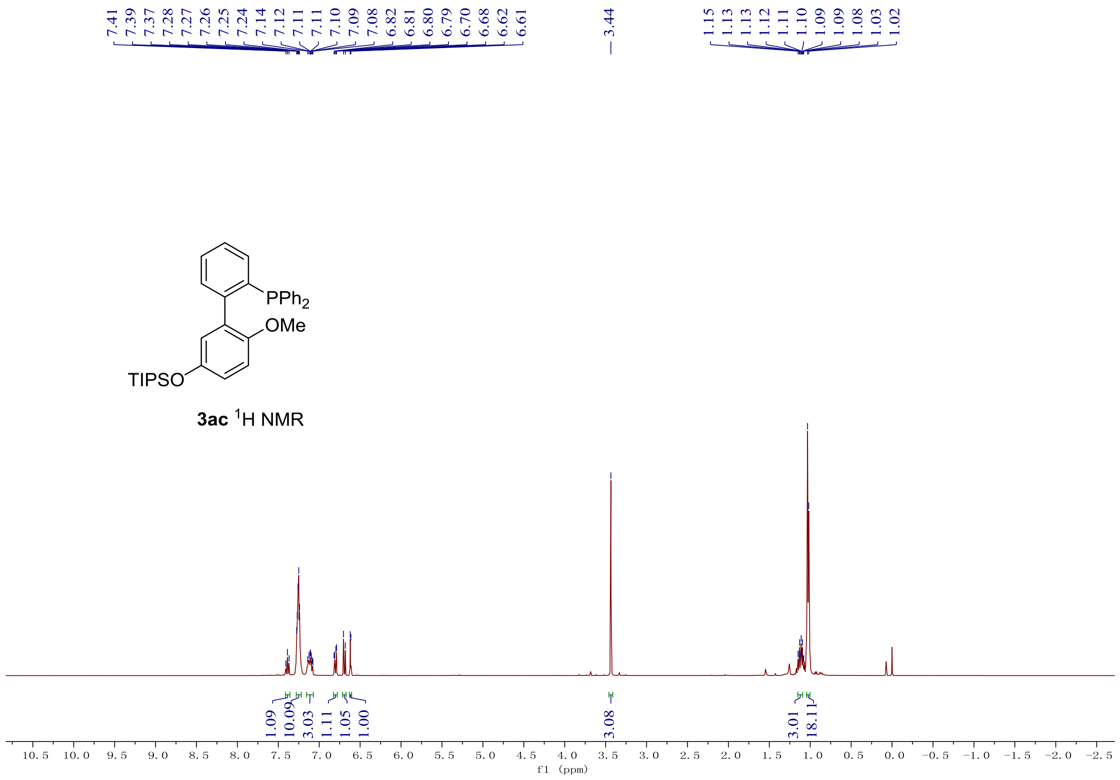

Supplementary Figure 18. ^{1}H NMR spectrum of **3ac**

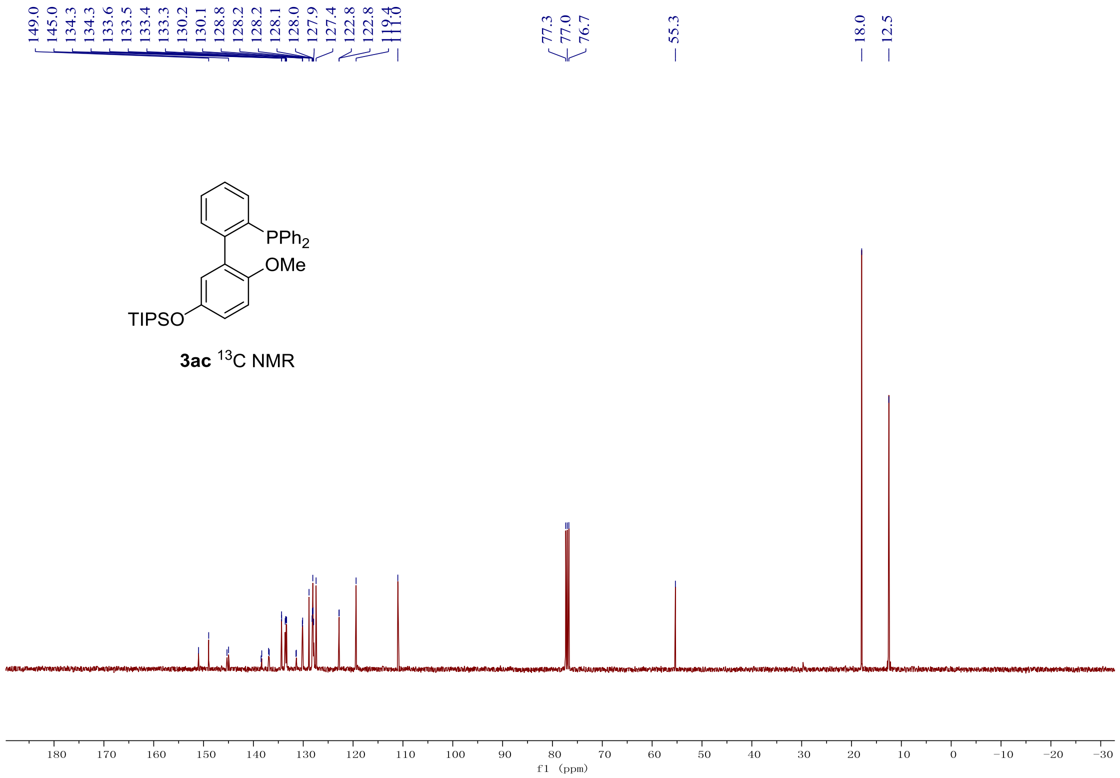

Supplementary Figure 19. ^{13}C NMR spectrum of **3ac**

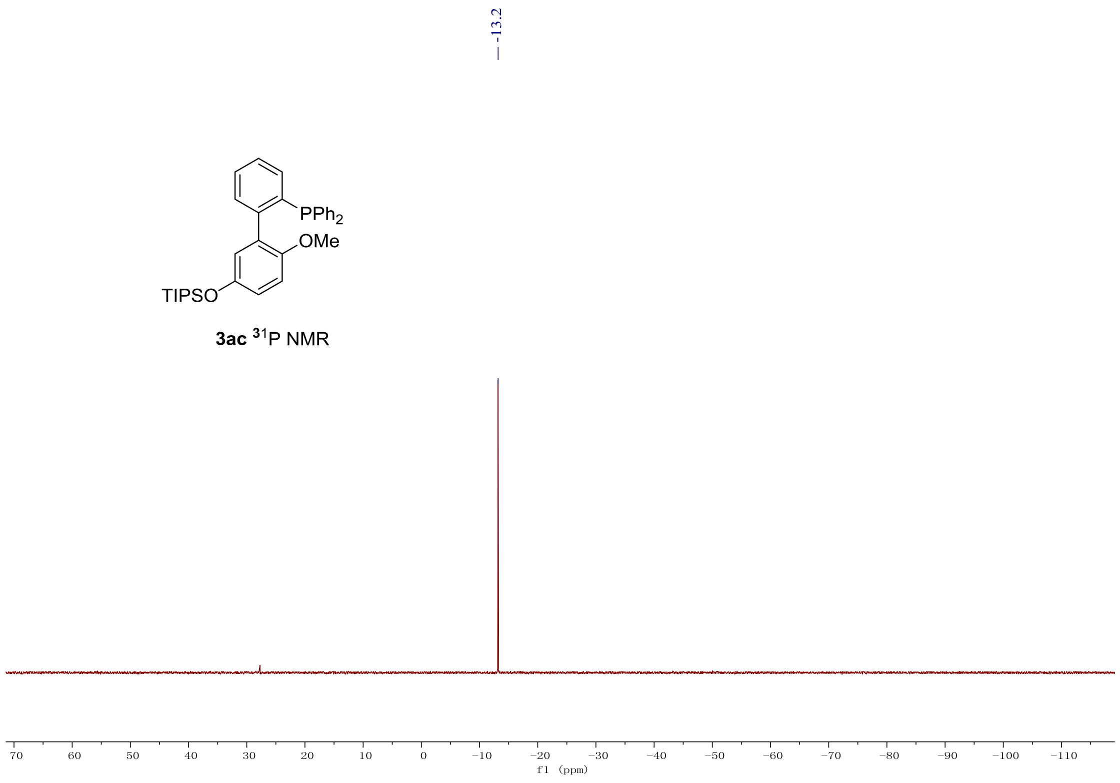

Supplementary Figure 20. ^{31}P NMR spectrum of **3ac**

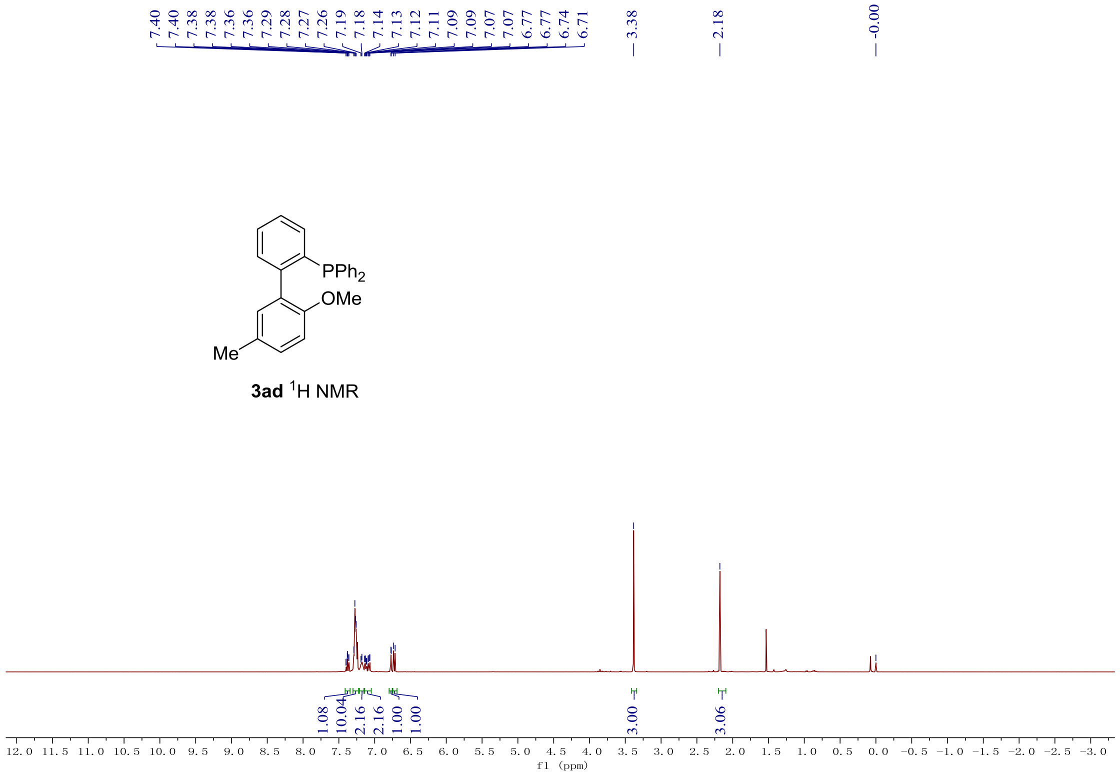

Supplementary Figure 21. ^{1}H NMR spectrum of **3ad**

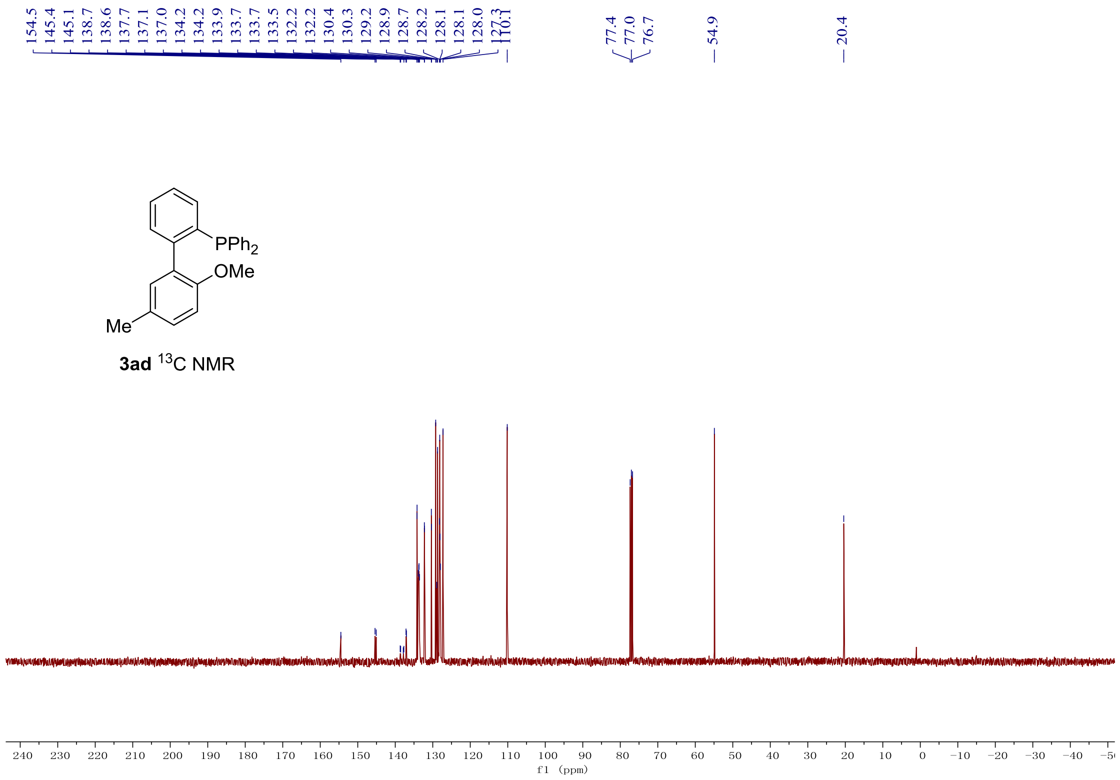

Supplementary Figure 22. ^{13}C NMR spectrum of **3ad**

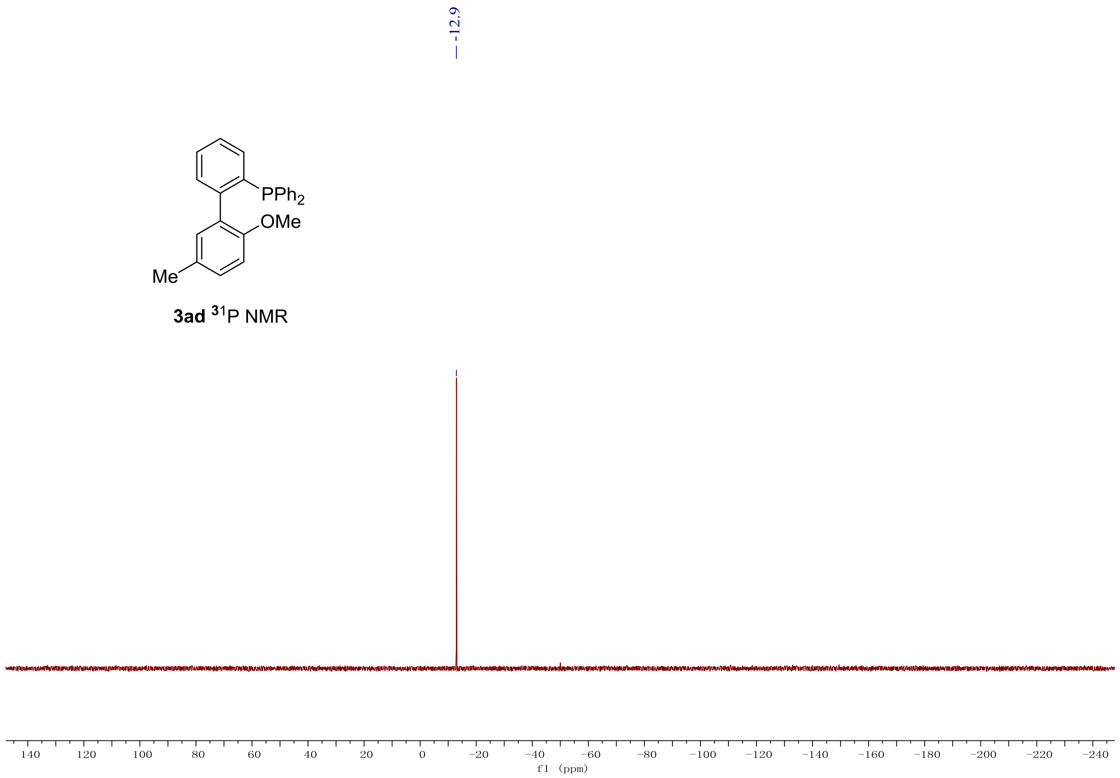

Supplementary Figure 23. ^{31}P NMR spectrum of **3ad**

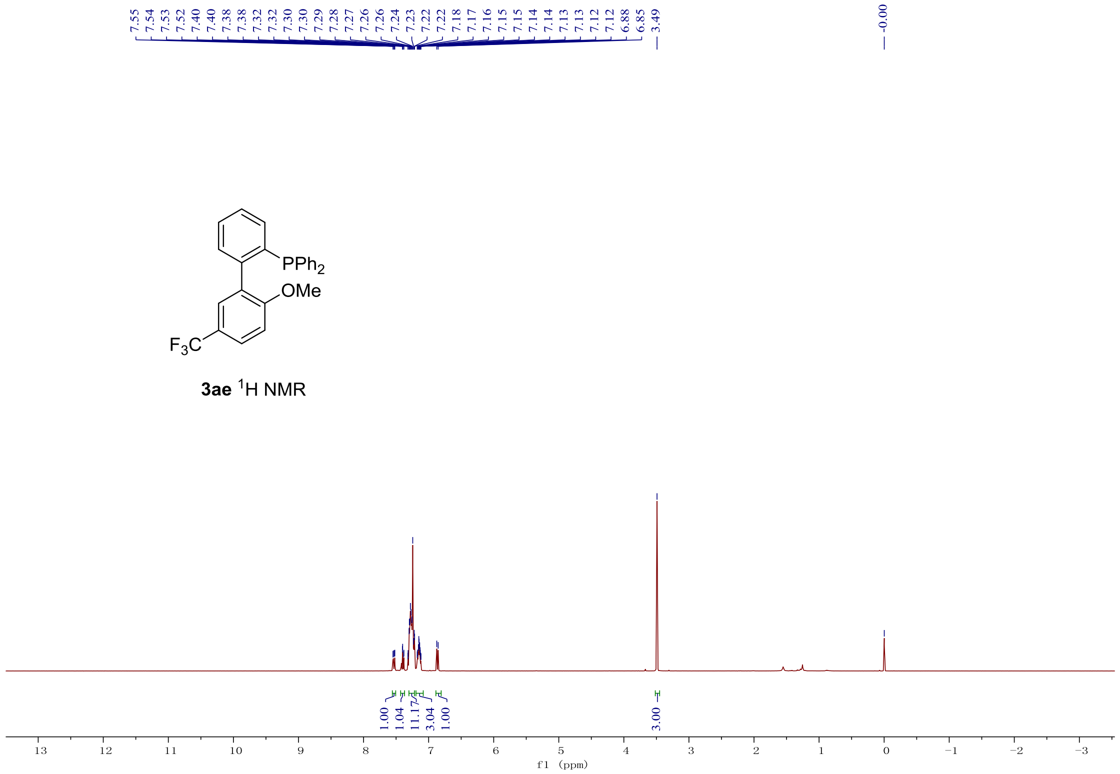

Supplementary Figure 24. ^{1}H NMR spectrum of **3ae**

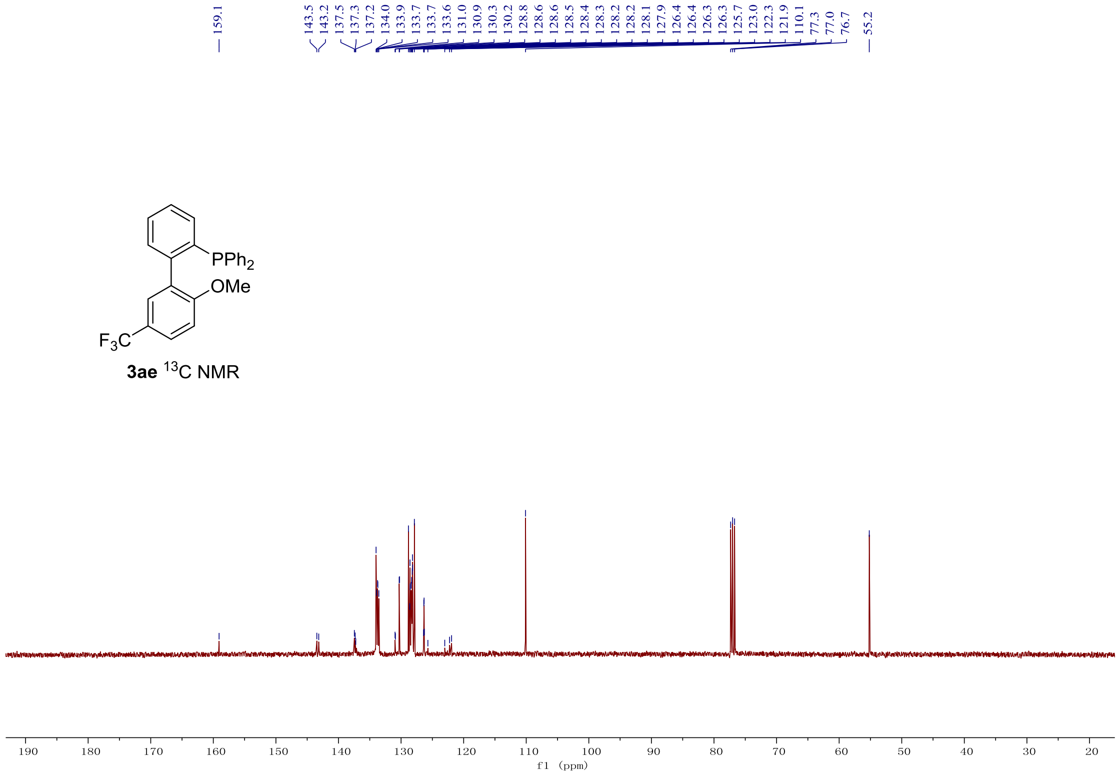

Supplementary Figure 25. ^{13}C NMR spectrum of **3ae**

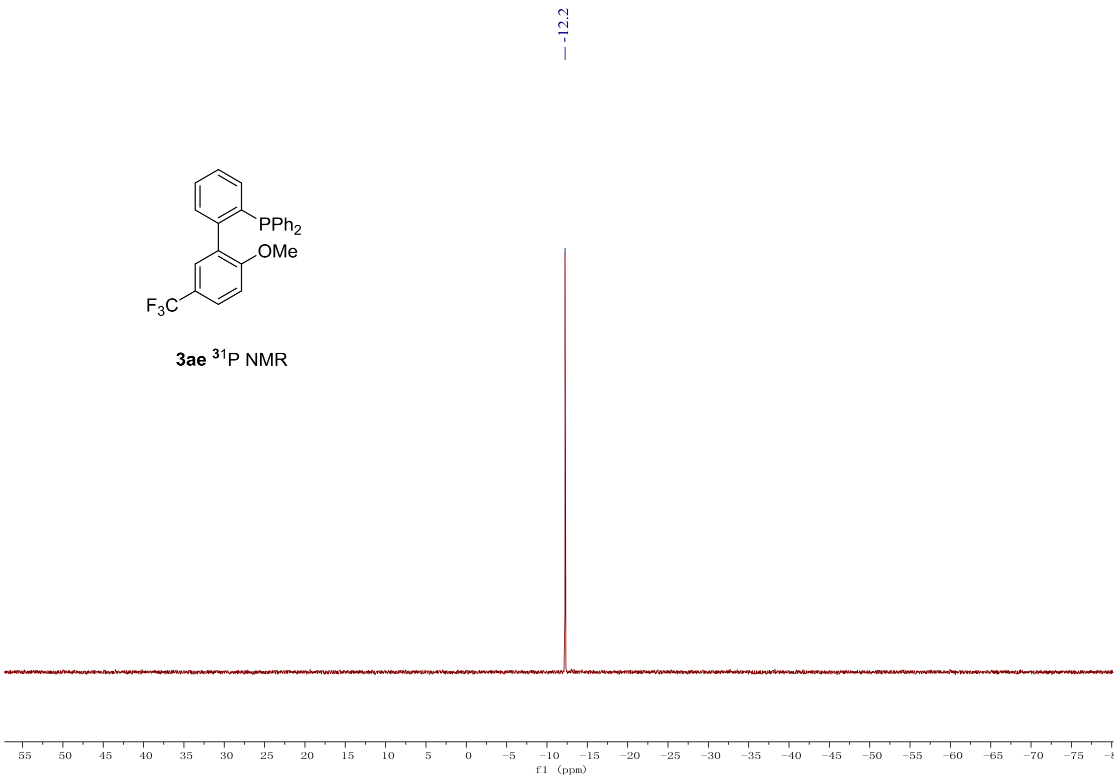

Supplementary Figure 26. ^{31}P NMR spectrum of **3ae**

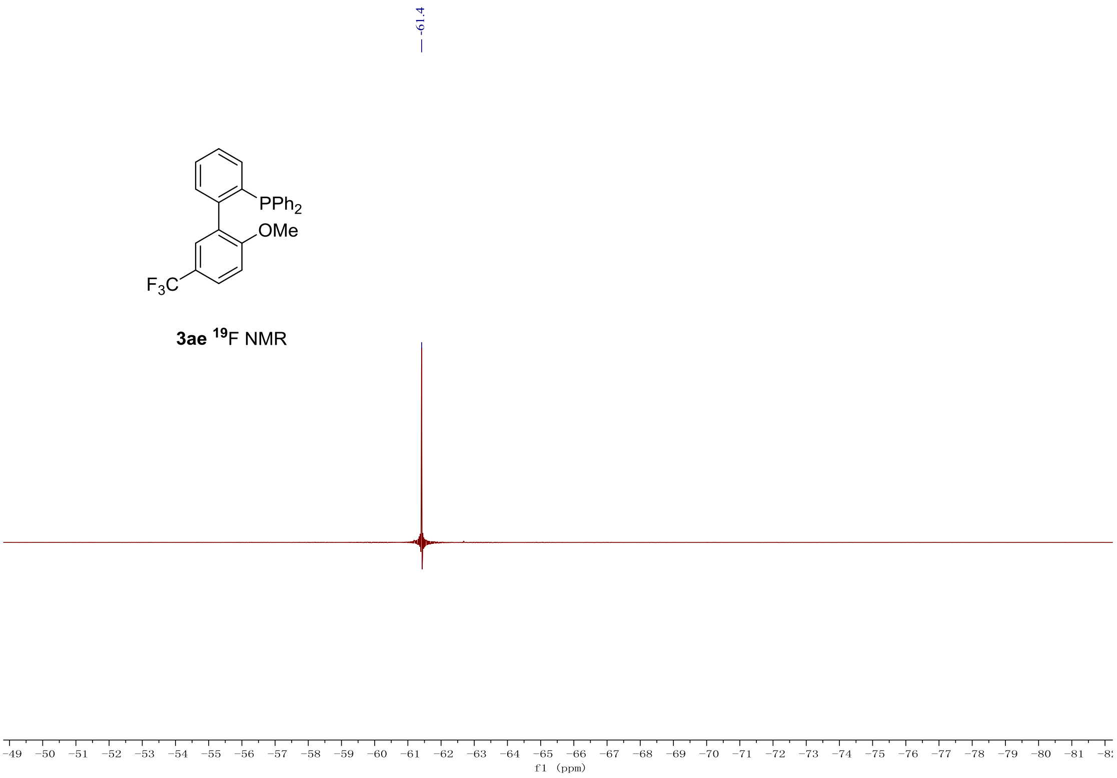

Supplementary Figure 27. ^{19}F NMR spectrum of **3ae**

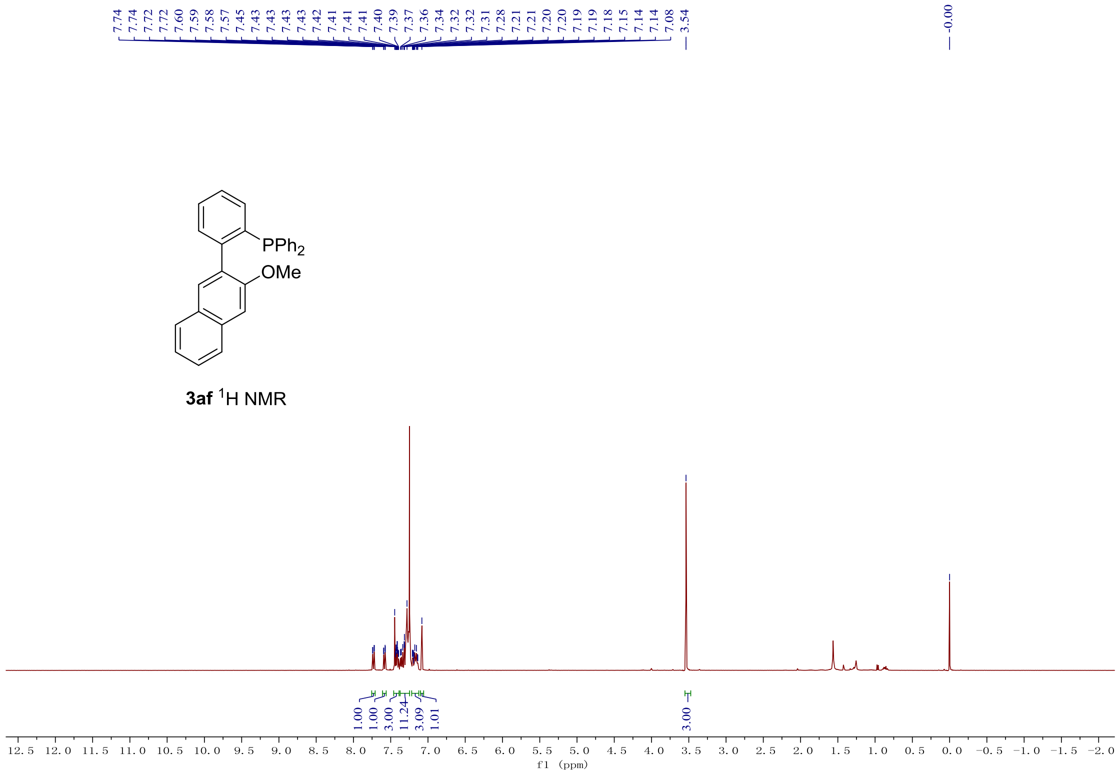

Supplementary Figure 28. ^{1}H NMR spectrum of **3af**

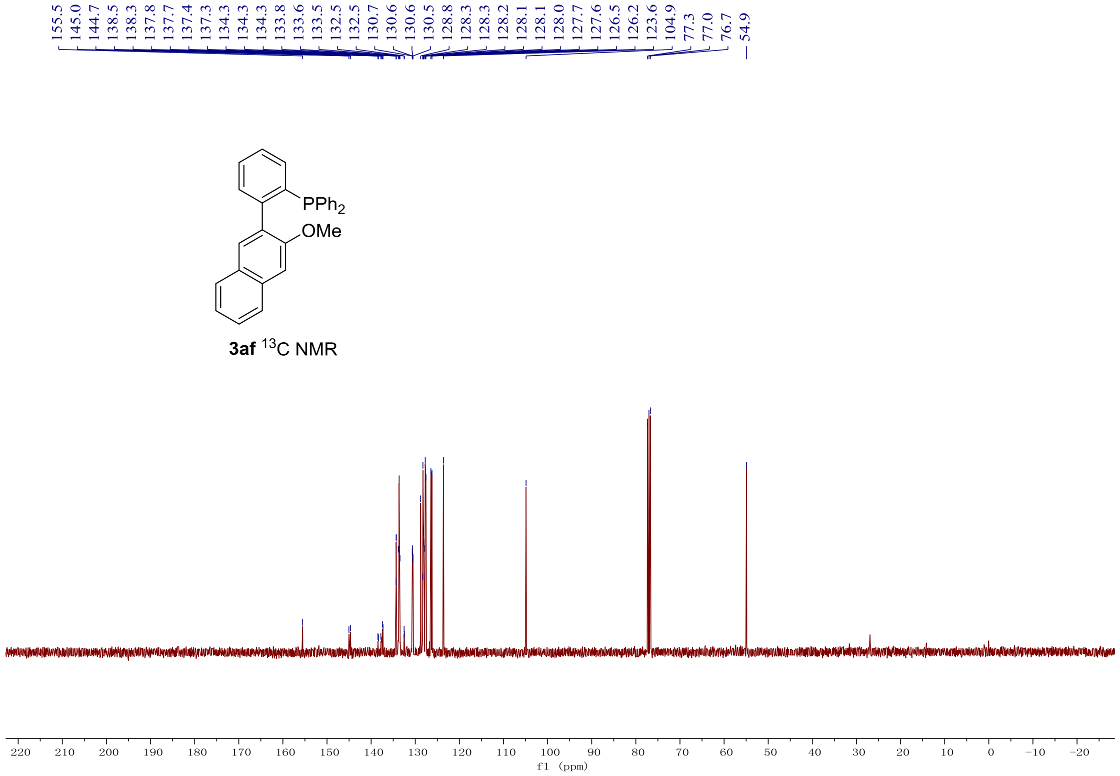

Supplementary Figure 29. ^{13}C NMR spectrum of **3af**

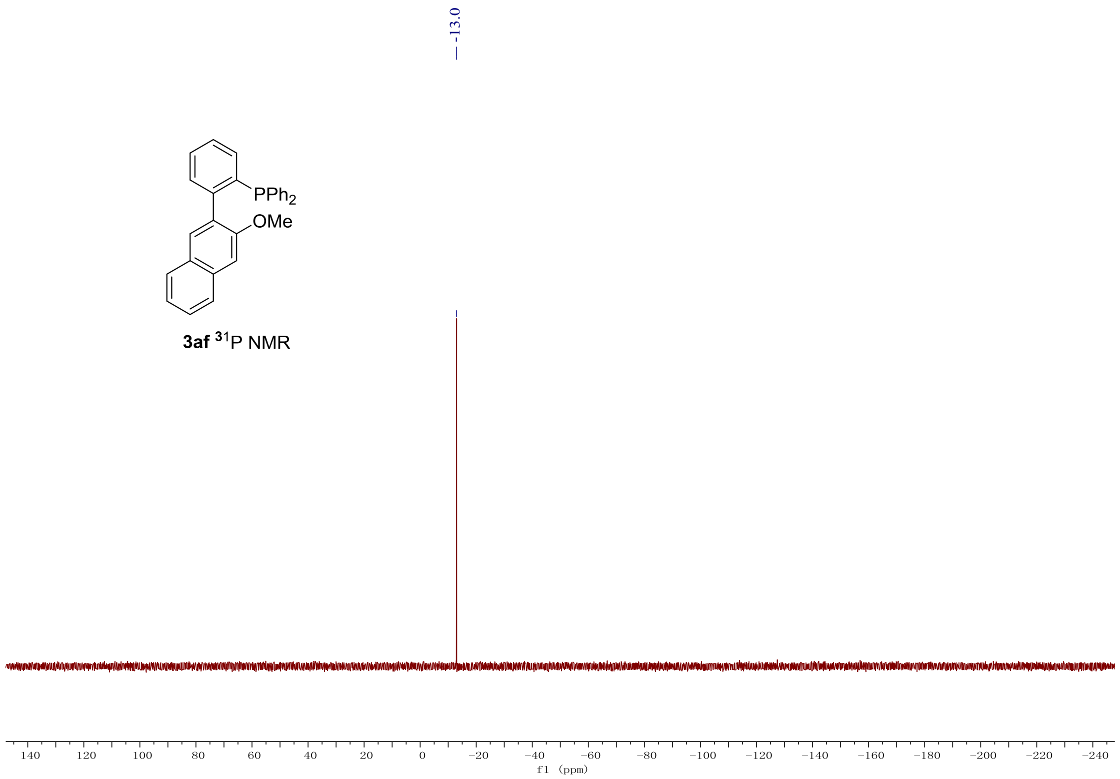

Supplementary Figure 30. ^{31}P NMR spectrum of **3af**

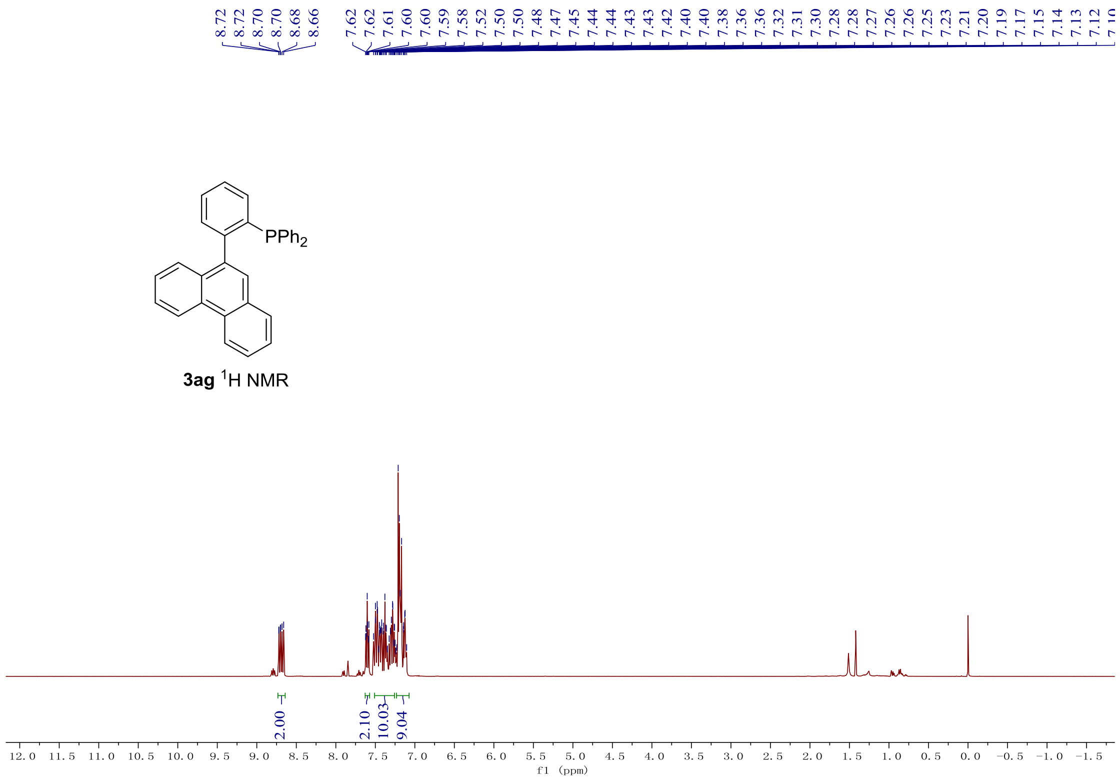

Supplementary Figure 31. ^{1}H NMR spectrum of **3ag**

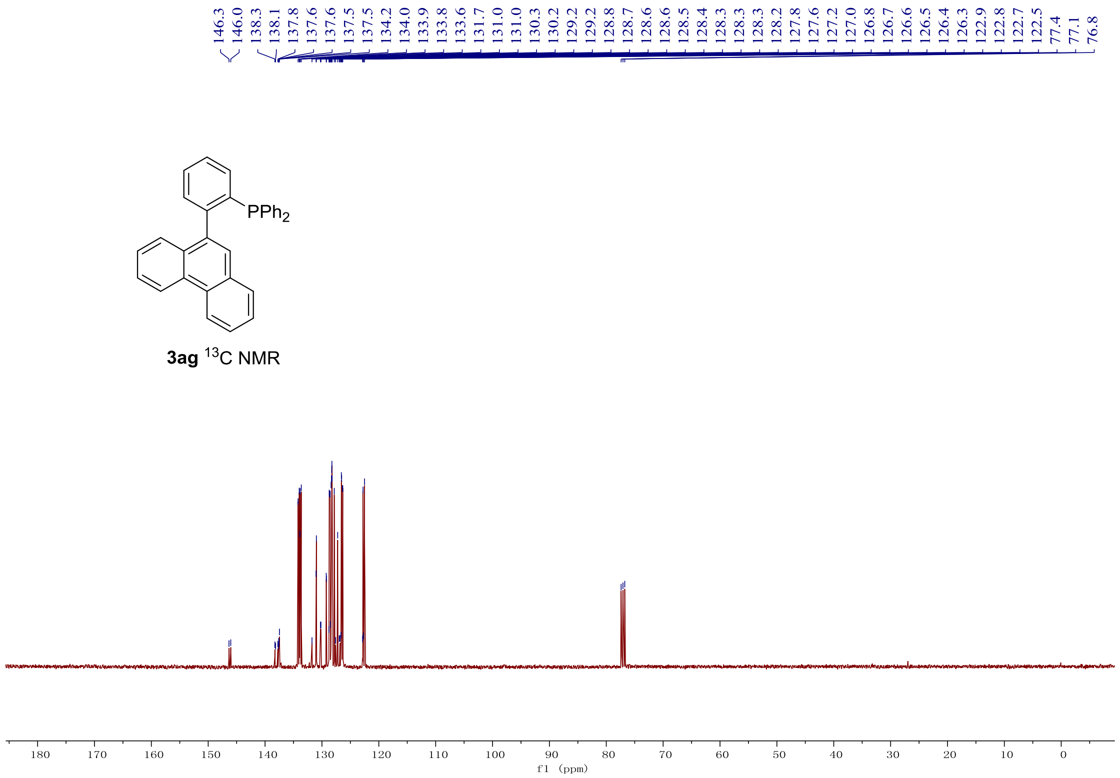

Supplementary Figure 32. ^{13}C NMR spectrum of **3ag**

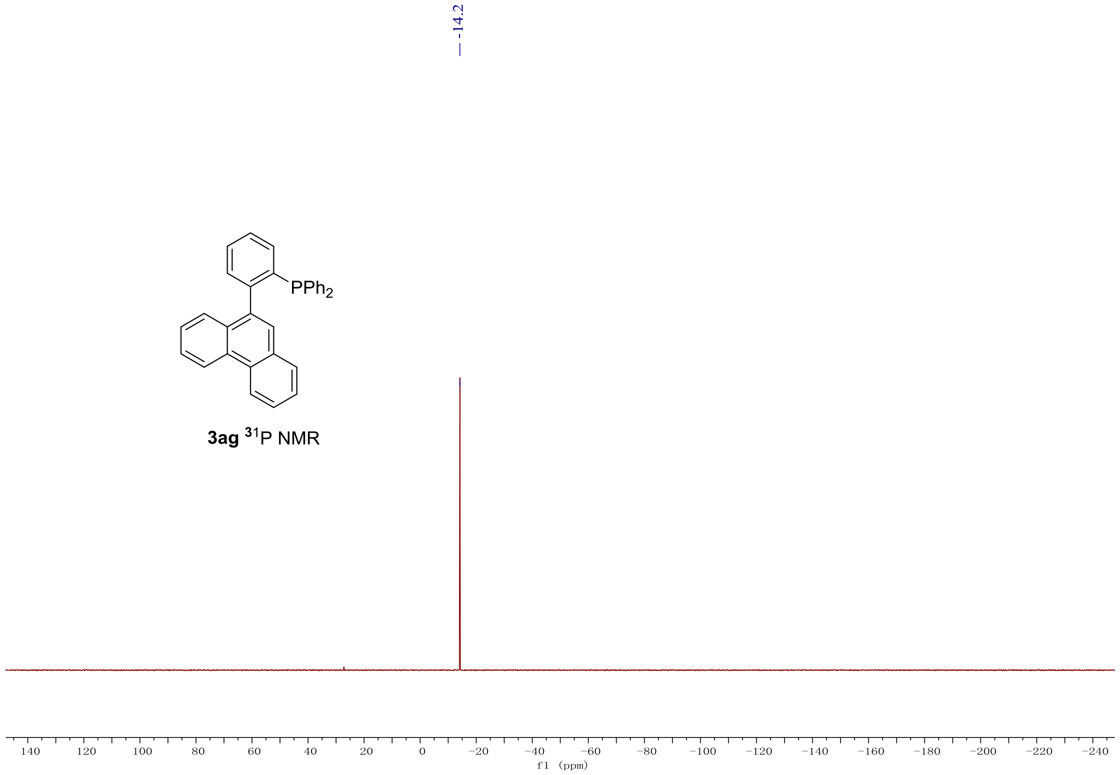

Supplementary Figure 33. ^{31}P NMR spectrum of **3ag**

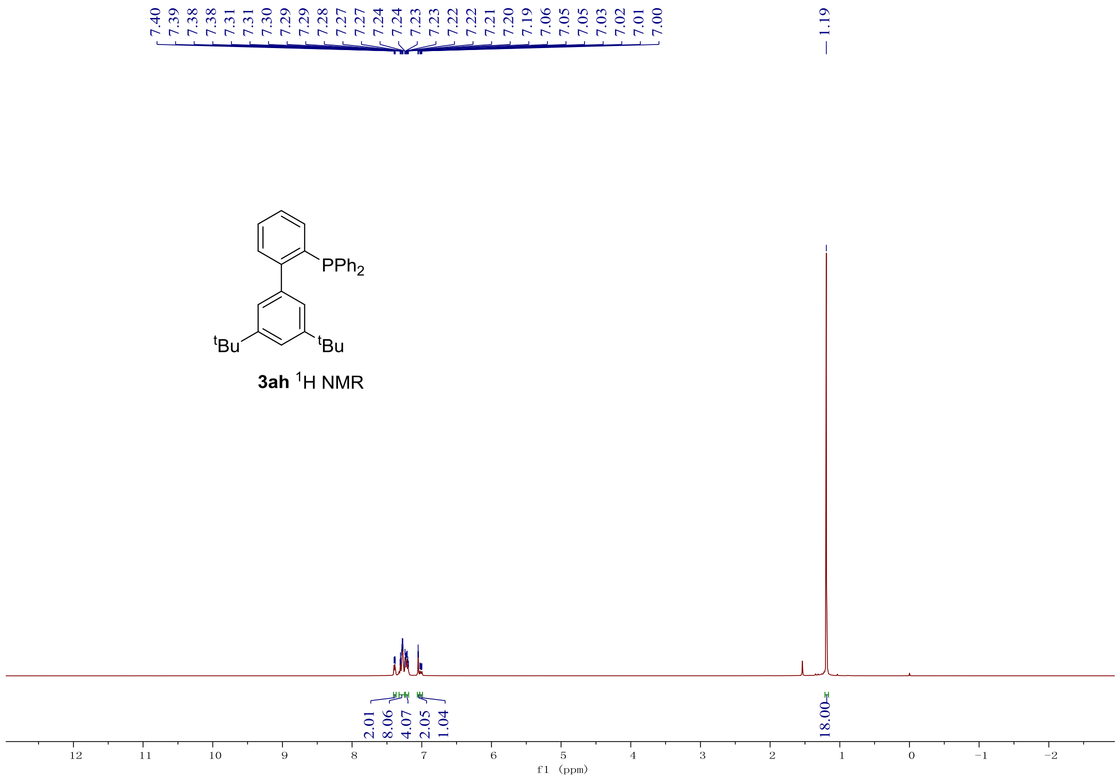

Supplementary Figure 34. ^{1}H NMR spectrum of **3ah**

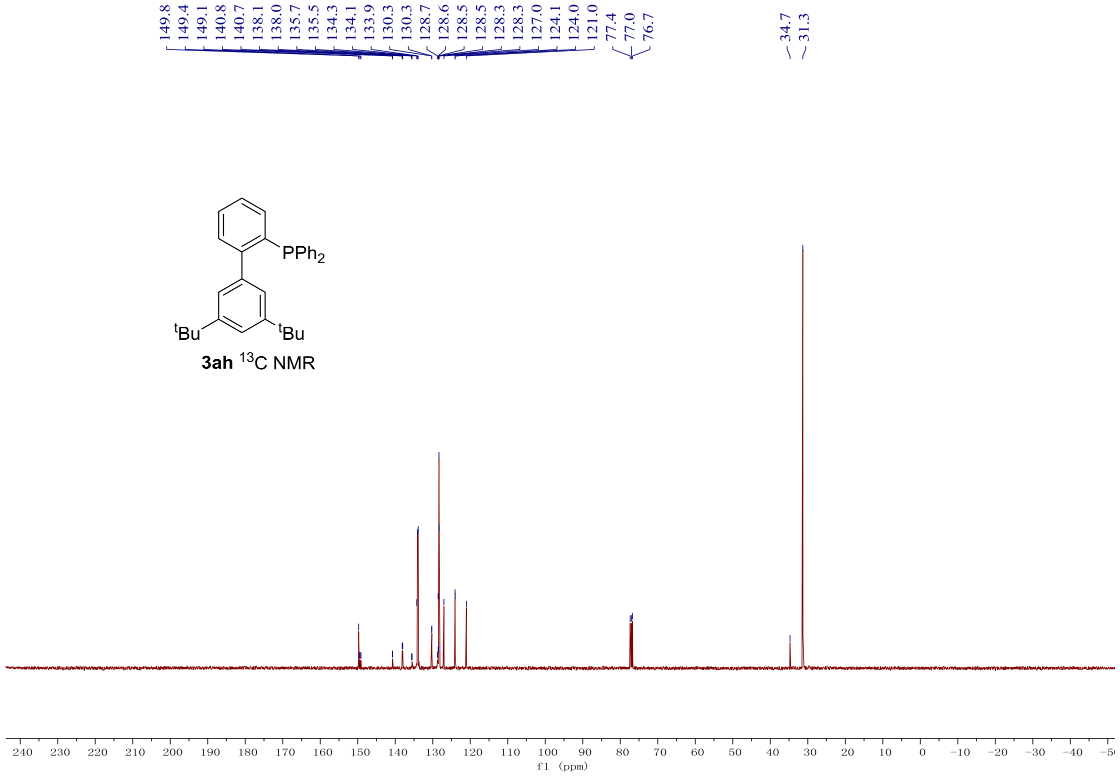

Supplementary Figure 35. ^{13}C NMR spectrum of **3ah**

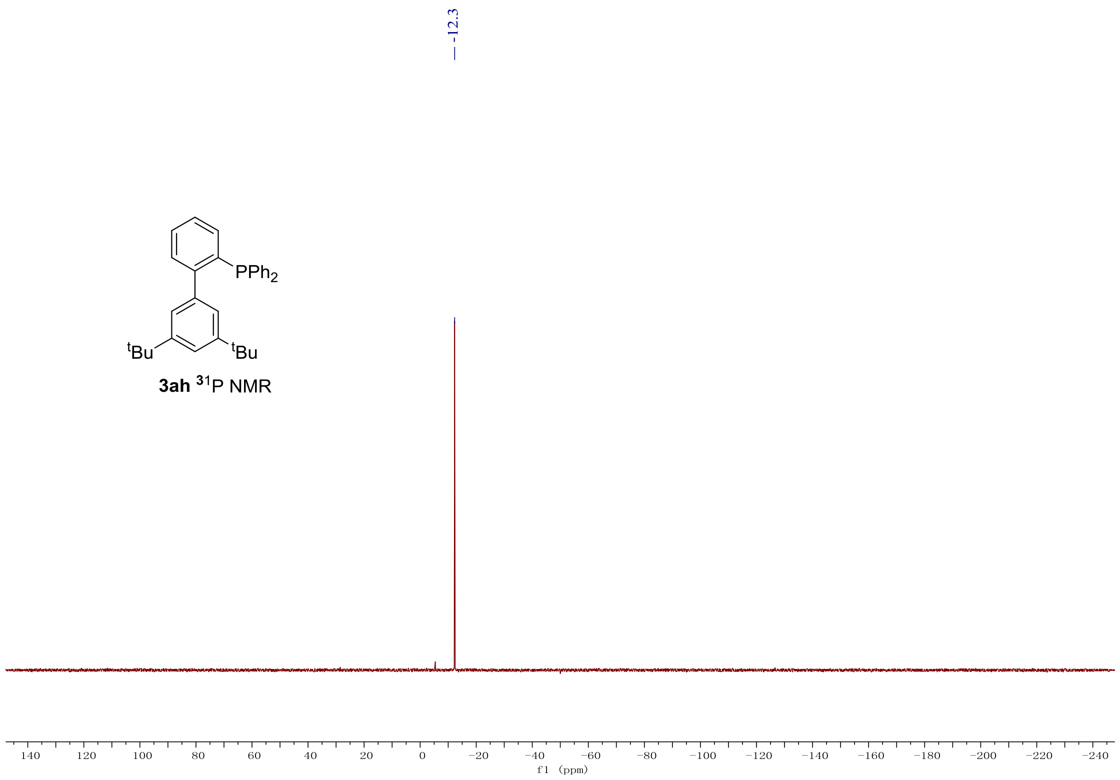

Supplementary Figure 36. ^{31}P NMR spectrum of **3ah**

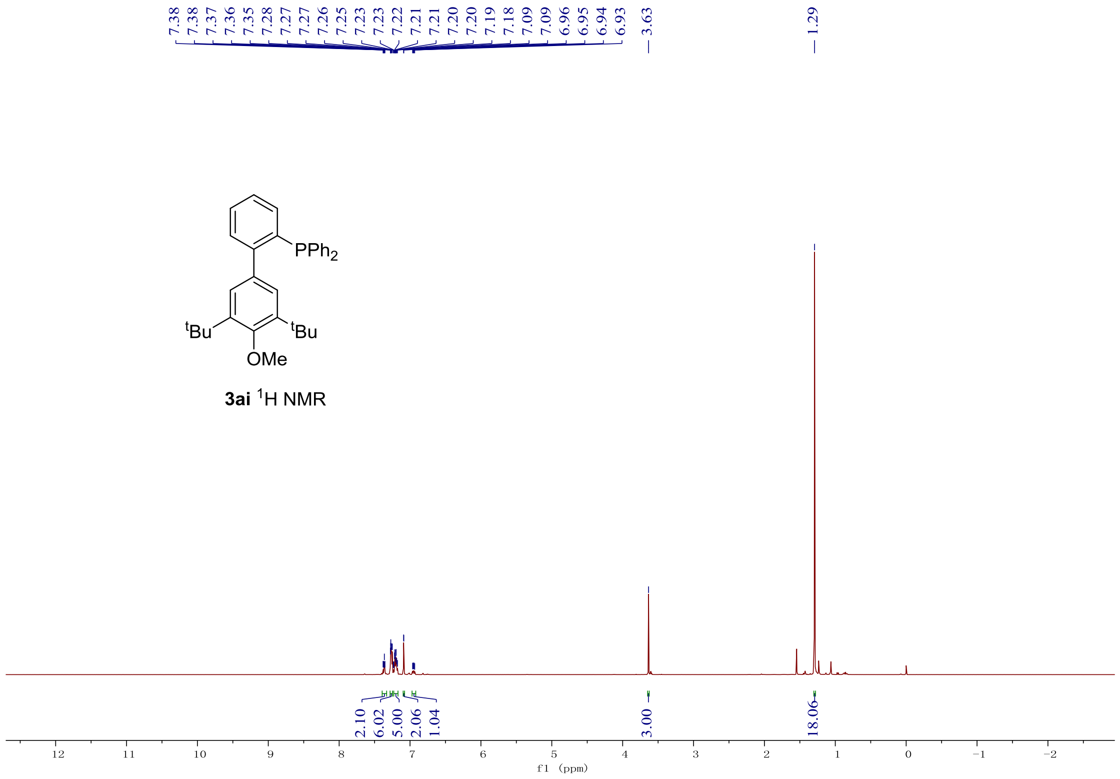

Supplementary Figure 37. ^{1}H NMR spectrum of **3ai**

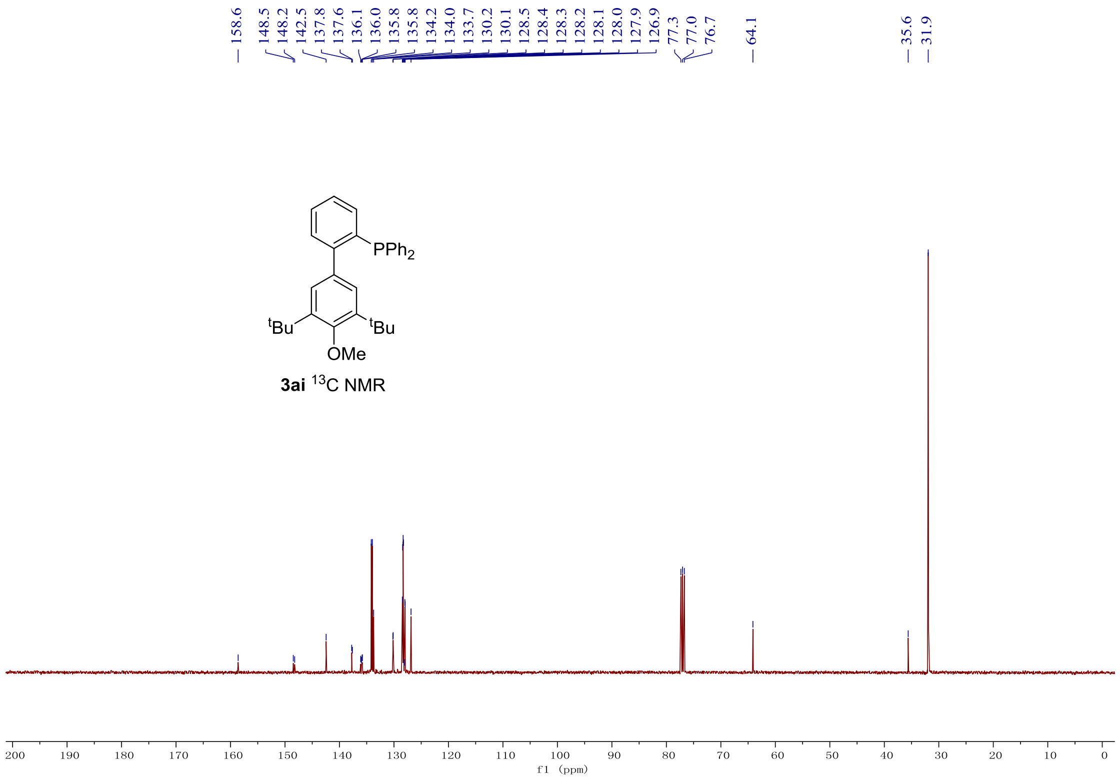

Supplementary Figure 38. ^{13}C NMR spectrum of **3ai**

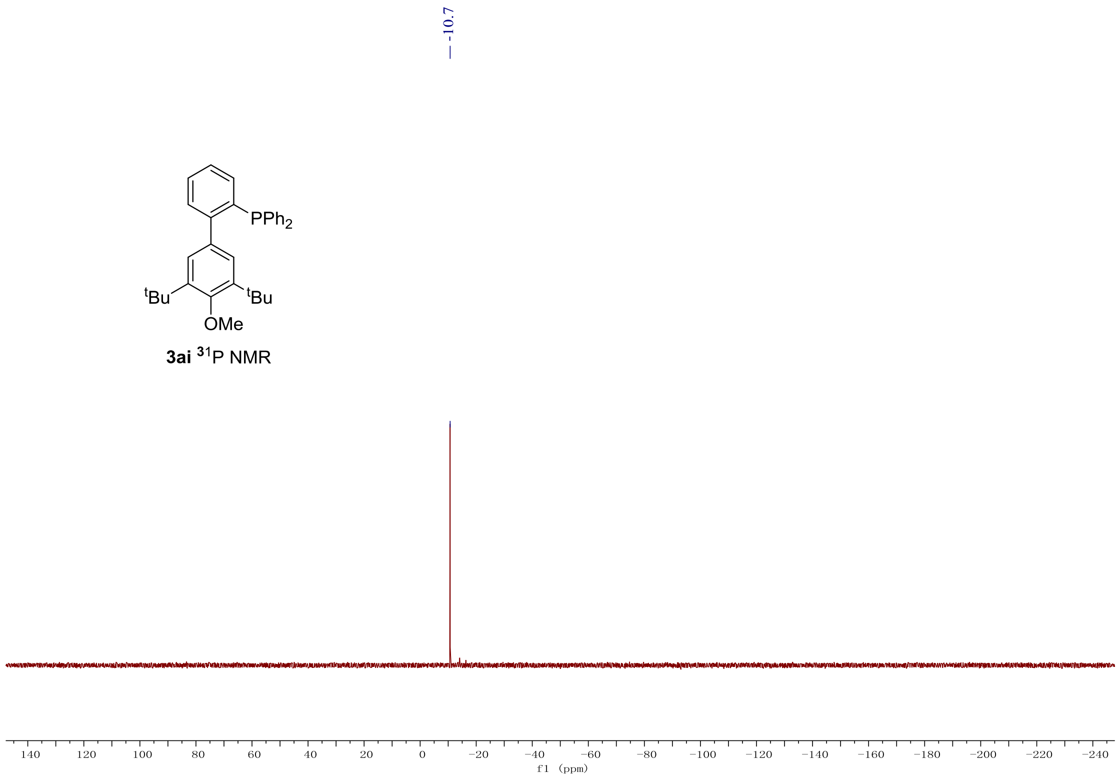

Supplementary Figure 39. ^{31}P NMR spectrum of **3ai**

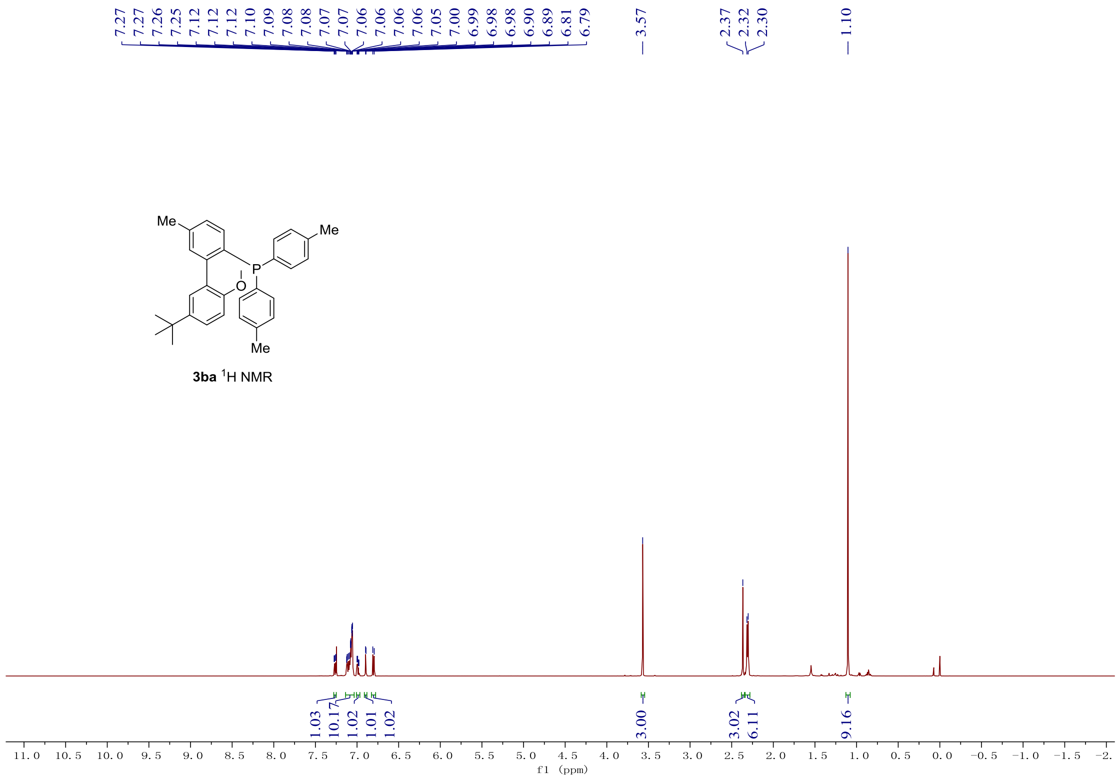

Supplementary Figure 40. ^{1}H NMR spectrum of **3ba**

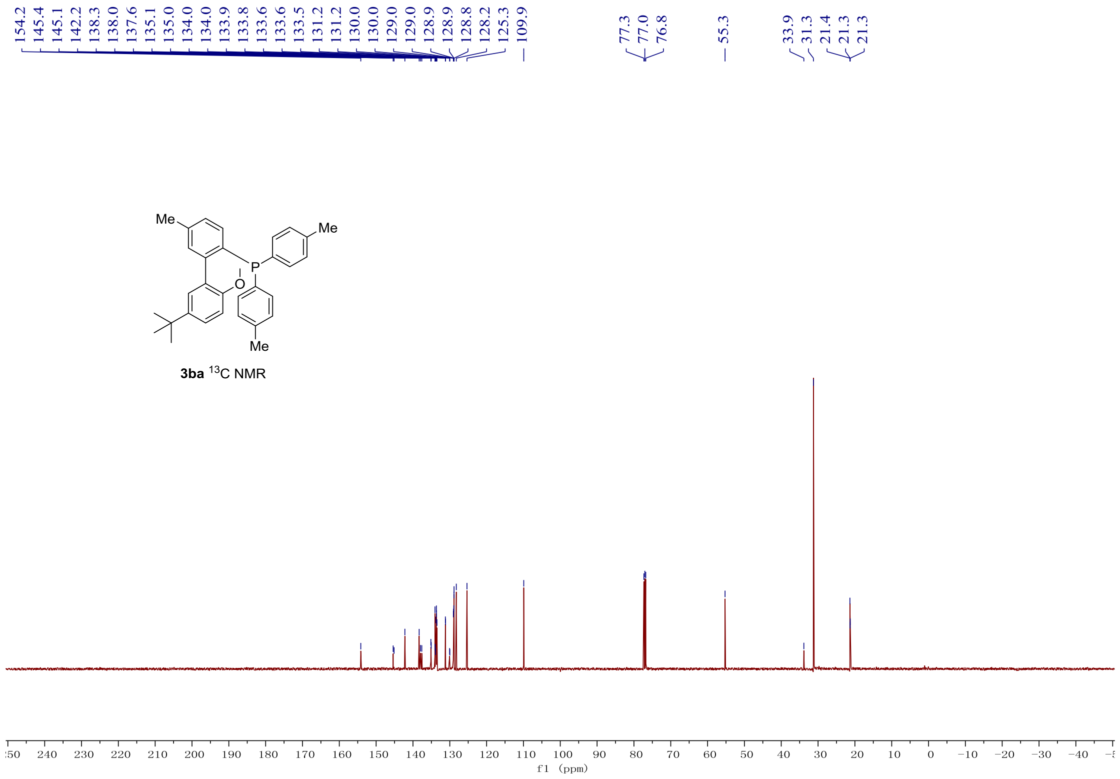

Supplementary Figure 41. ^{13}C NMR spectrum of **3ba**

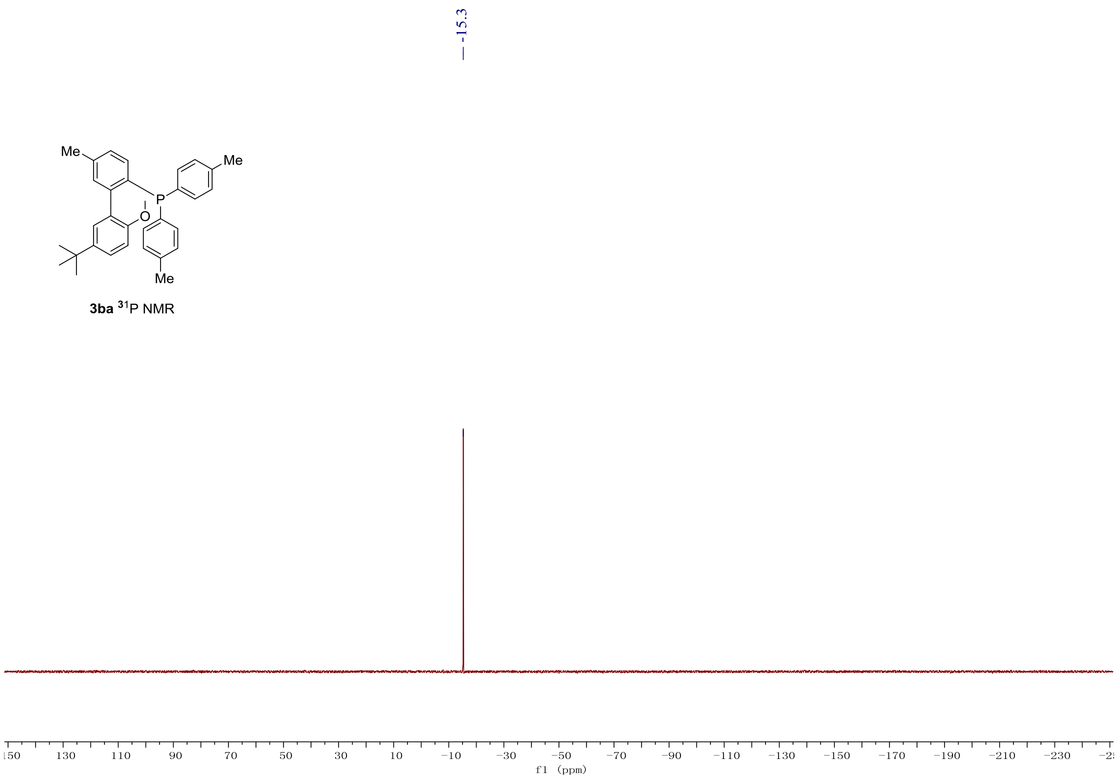

Supplementary Figure 42. ^{31}P NMR spectrum of **3ba**

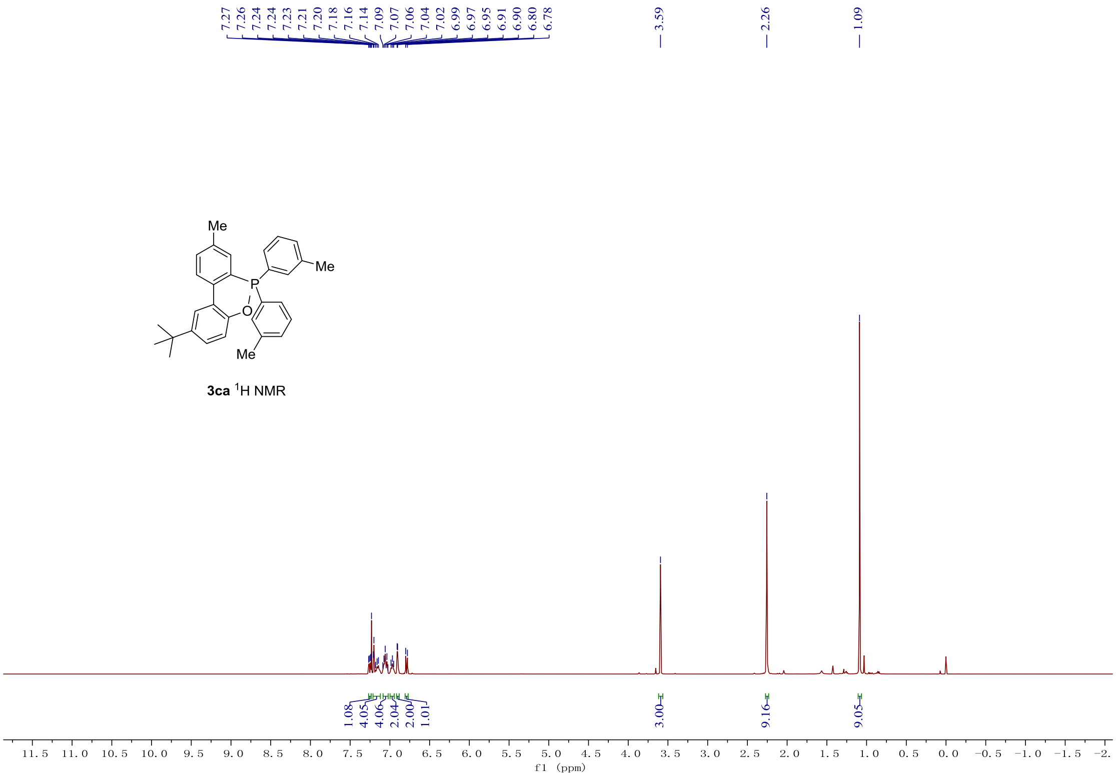

Supplementary Figure 43. ^{1}H NMR spectrum of **3ca**

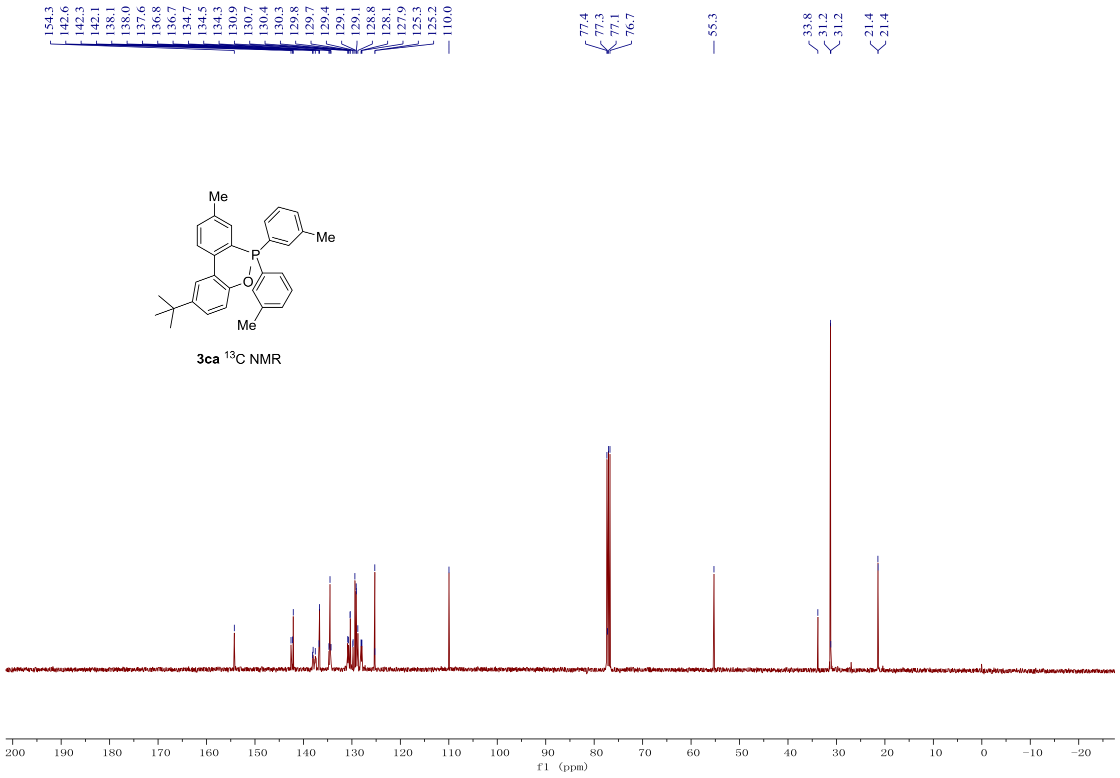

Supplementary Figure 44. ^{13}C NMR spectrum of **3ca**

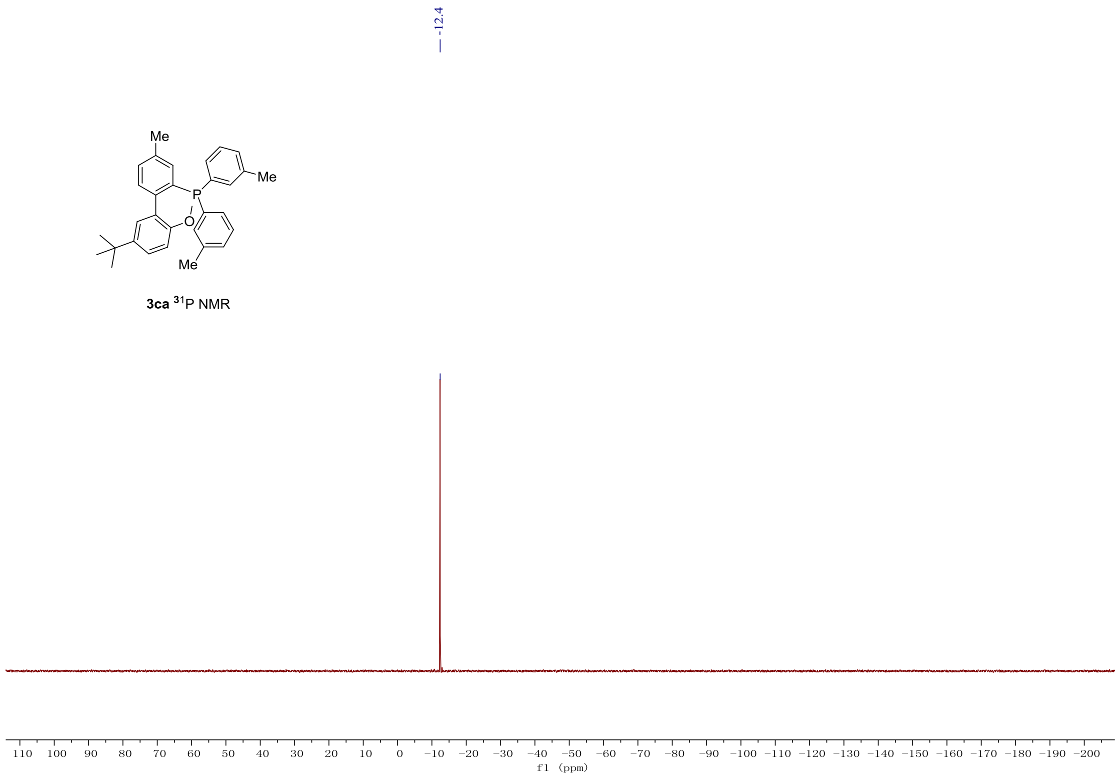

Supplementary Figure 45. ^{31}P NMR spectrum of **3ca**

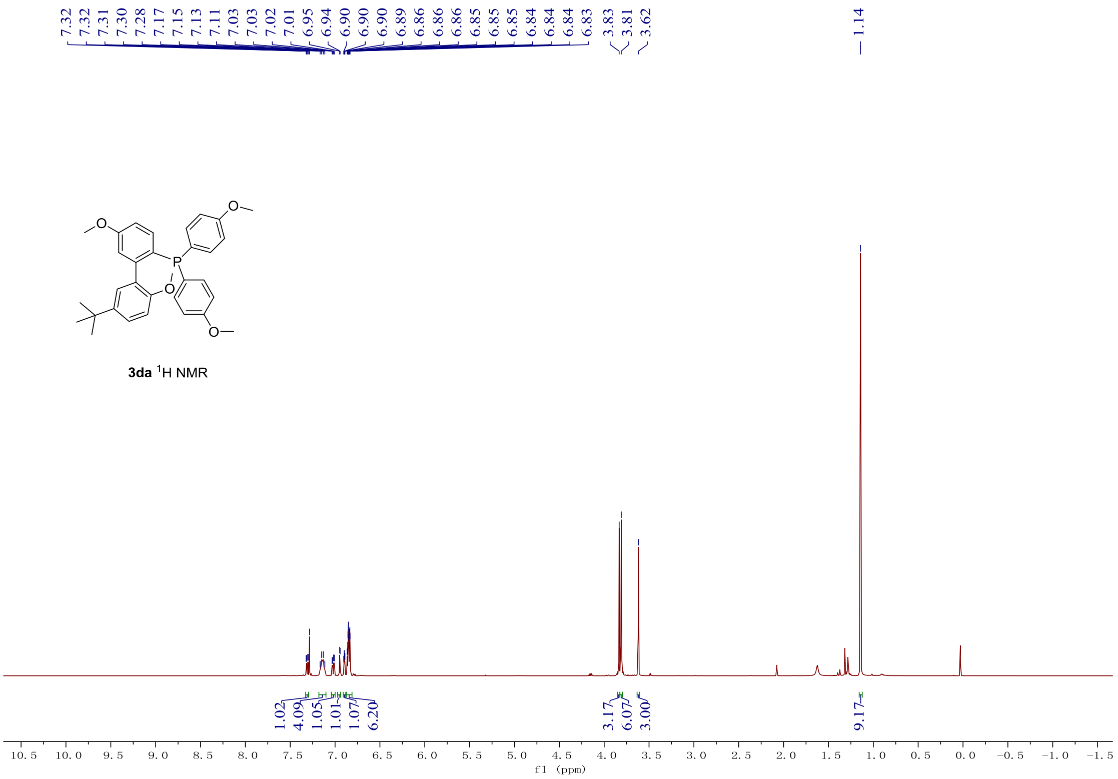

Supplementary Figure 46. ^{1}H NMR spectrum of **3da**

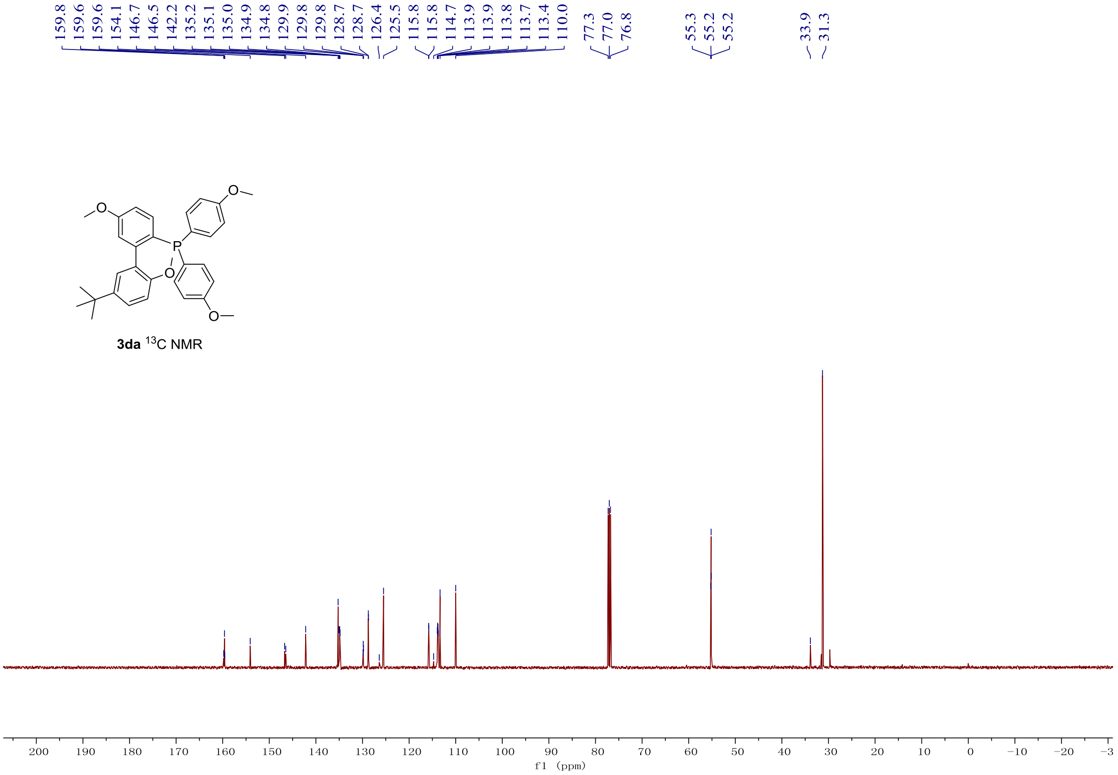

Supplementary Figure 47. ^{13}C NMR spectrum of **3da**

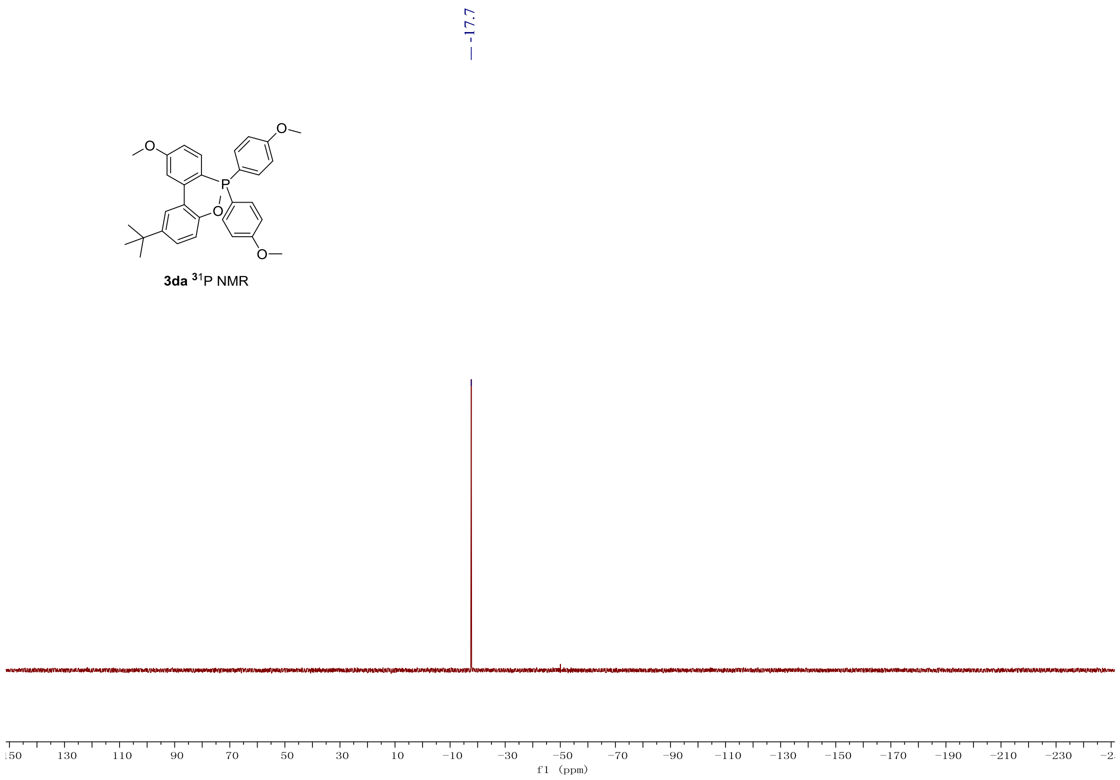

Supplementary Figure 48. ^{31}P NMR spectrum of **3da**

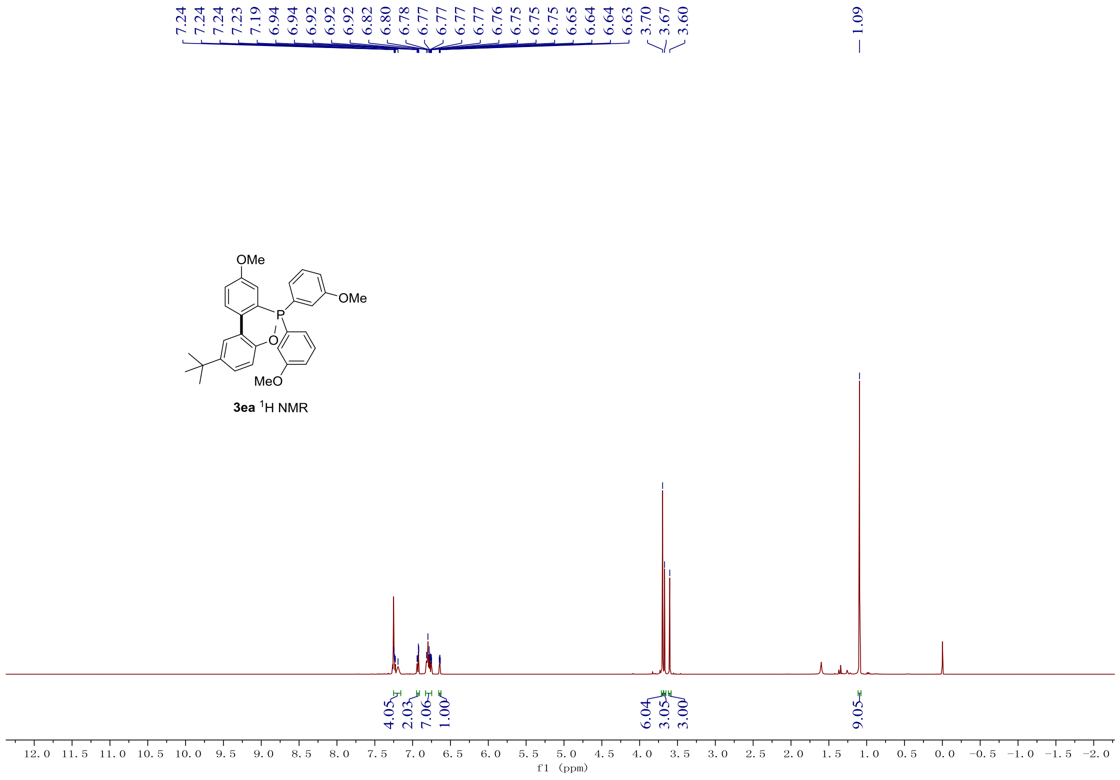

Supplementary Figure 49. ^{1}H NMR spectrum of **3ea**

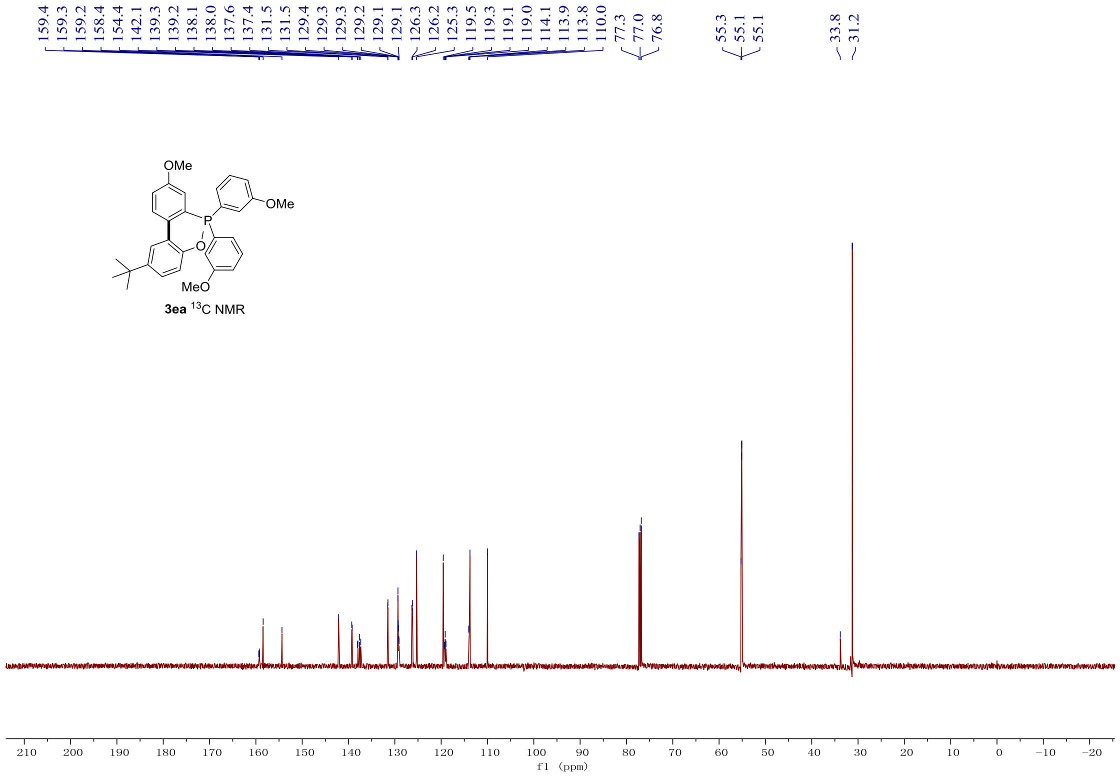

Supplementary Figure 50. ^{13}C NMR spectrum of **3ea**

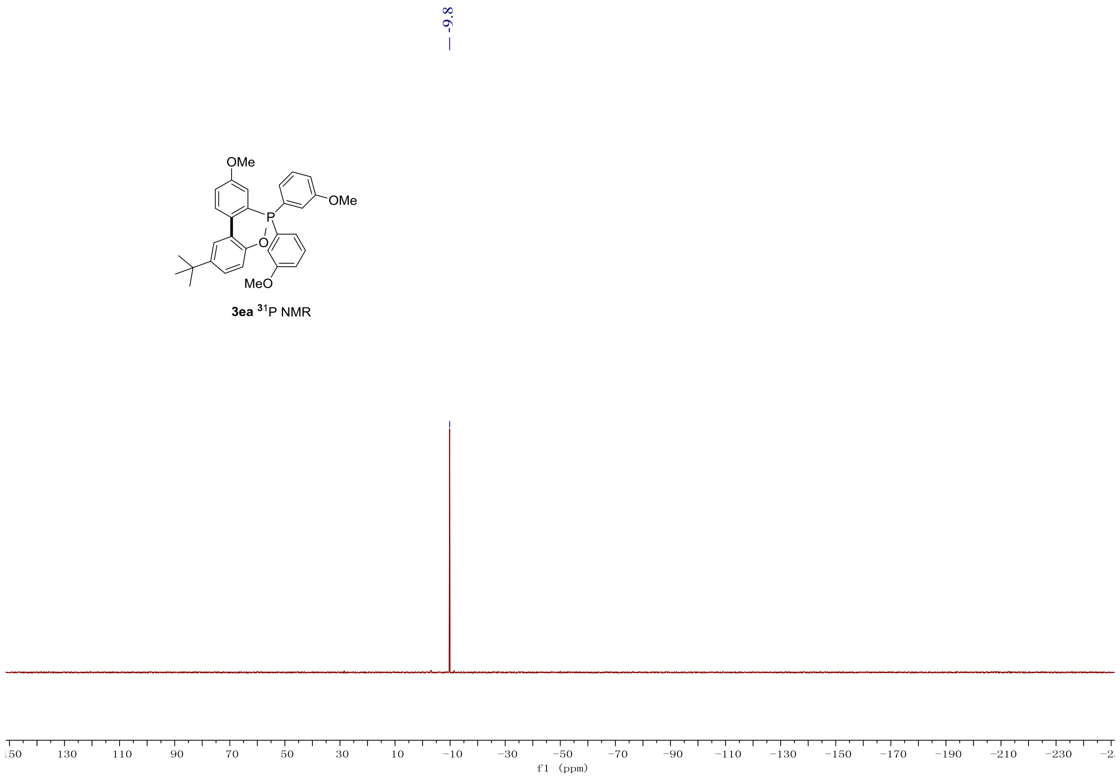

Supplementary Figure 51. ^{31}P NMR spectrum of **3ea**

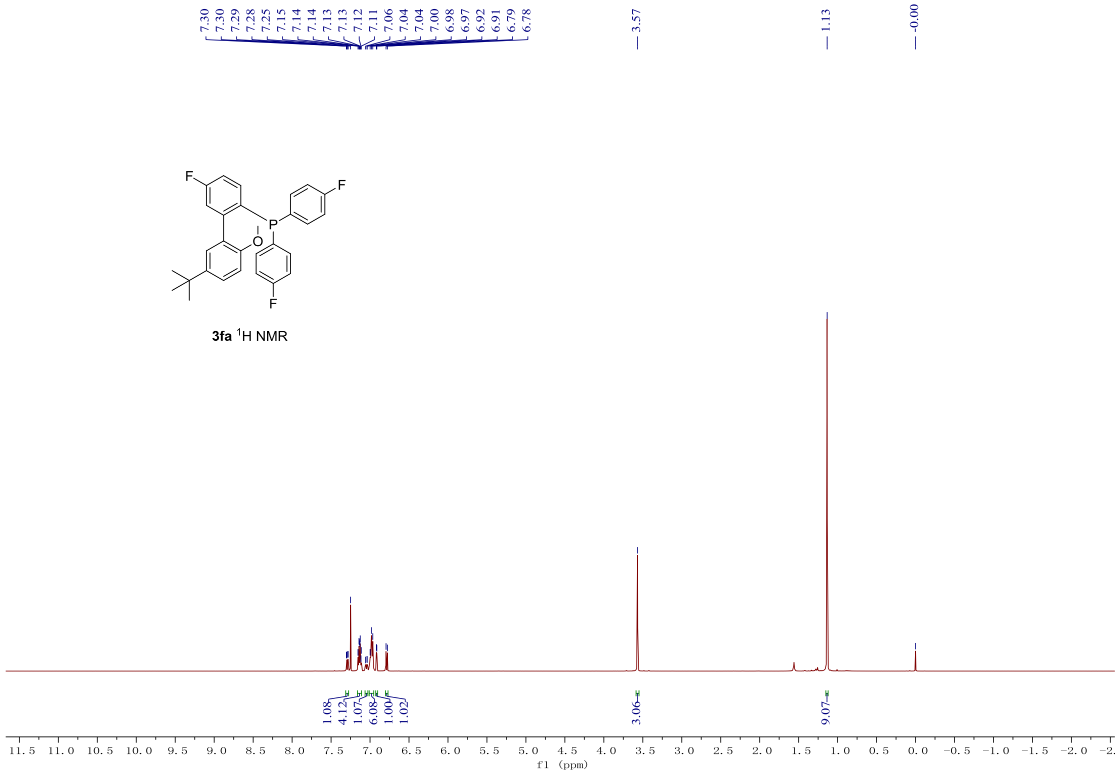

Supplementary Figure 52. ^{1}H NMR spectrum of **3fa**

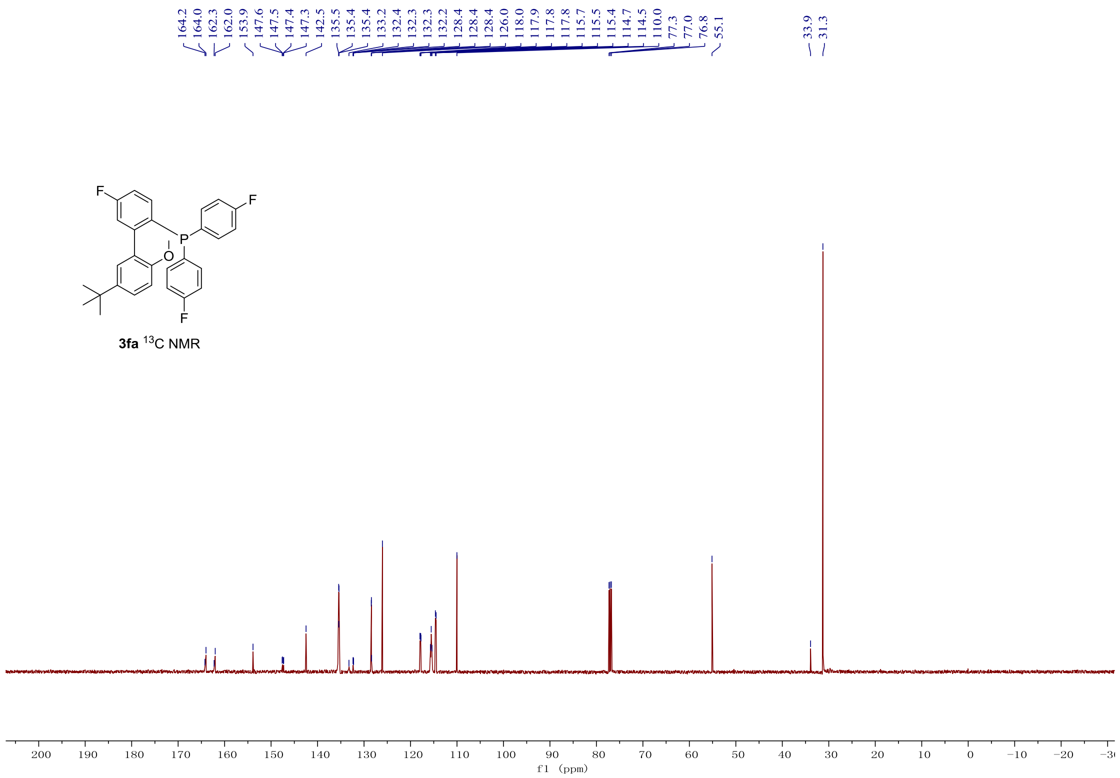

Supplementary Figure 53. ^{13}C NMR spectrum of **3fa**

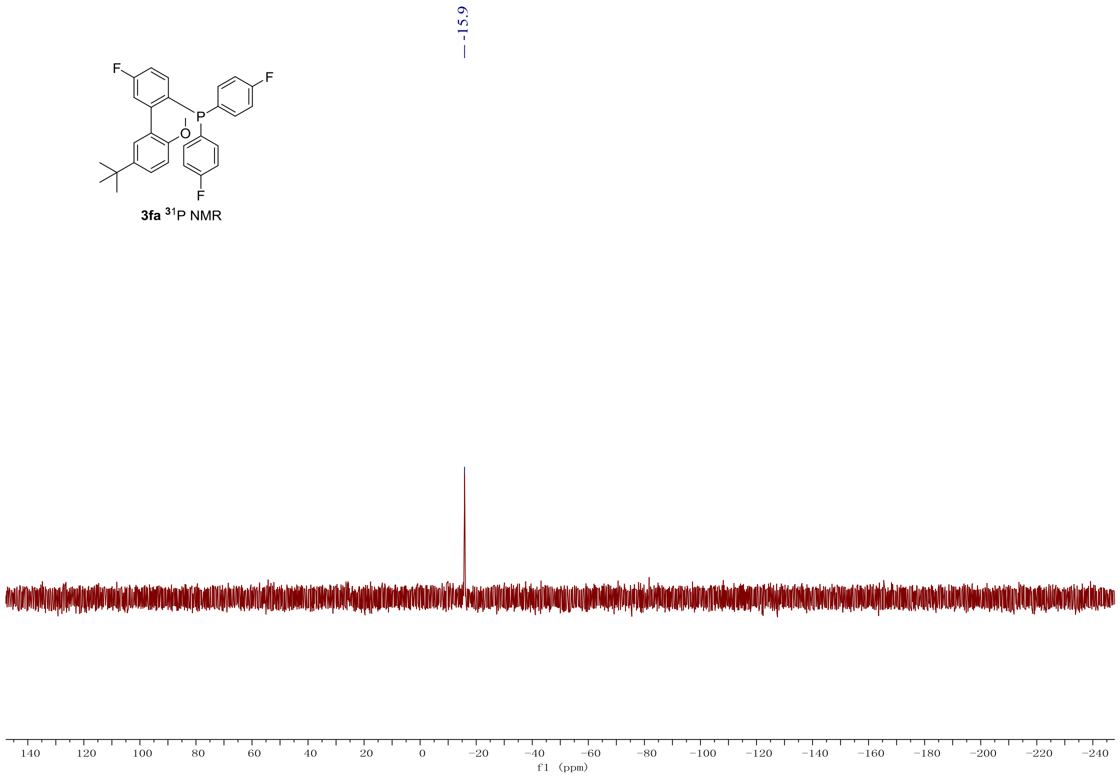

Supplementary Figure 54. ^{31}P NMR spectrum of **3fa**

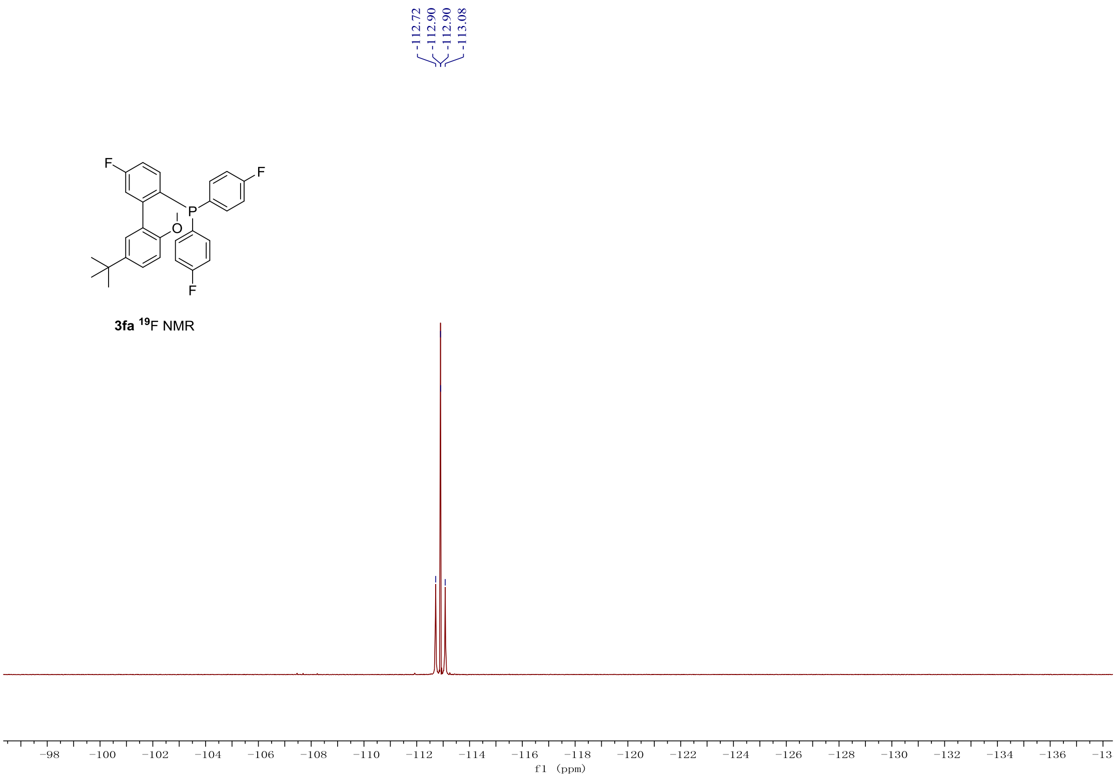

Supplementary Figure 55. ^{19}F NMR spectrum of **3fa**

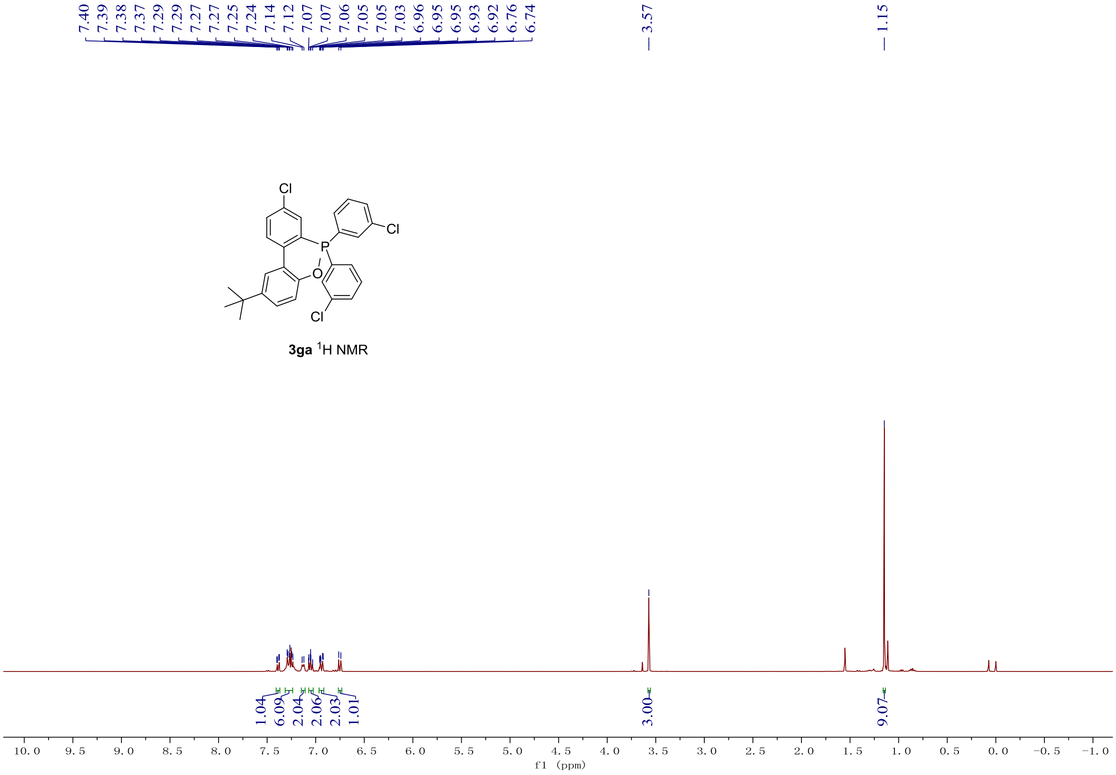

Supplementary Figure 56. ^{1}H NMR spectrum of **3ga**

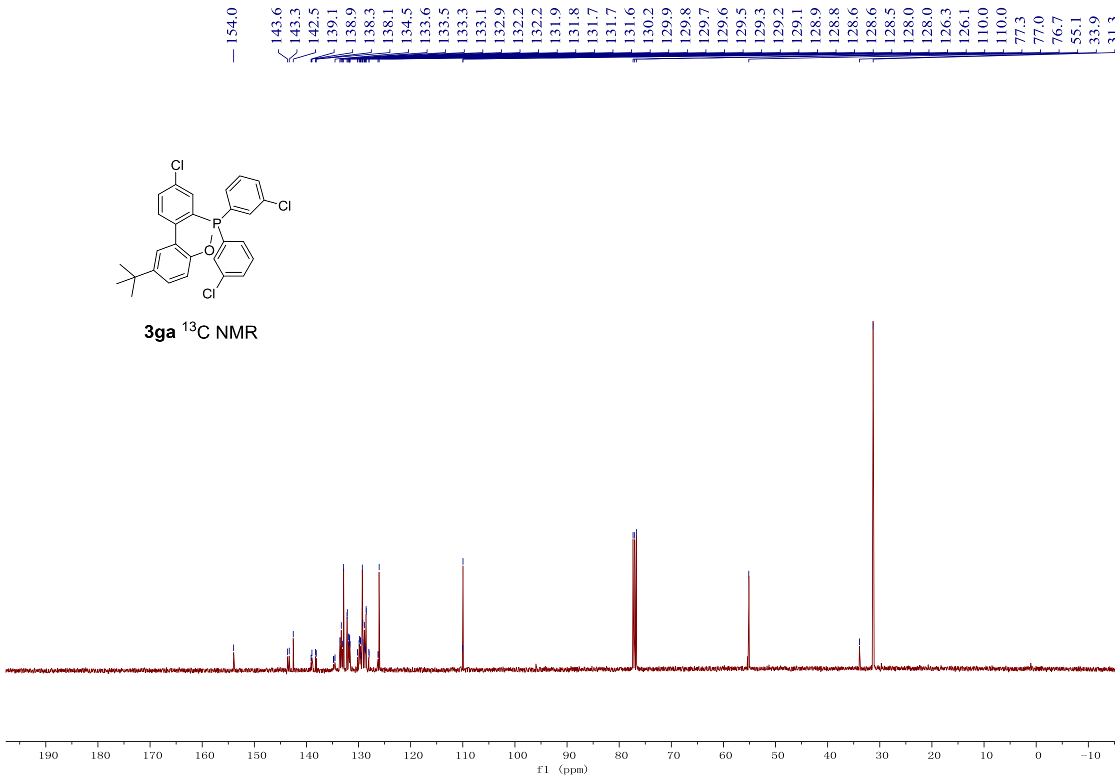

Supplementary Figure 57. ^{13}C NMR spectrum of **3ga**

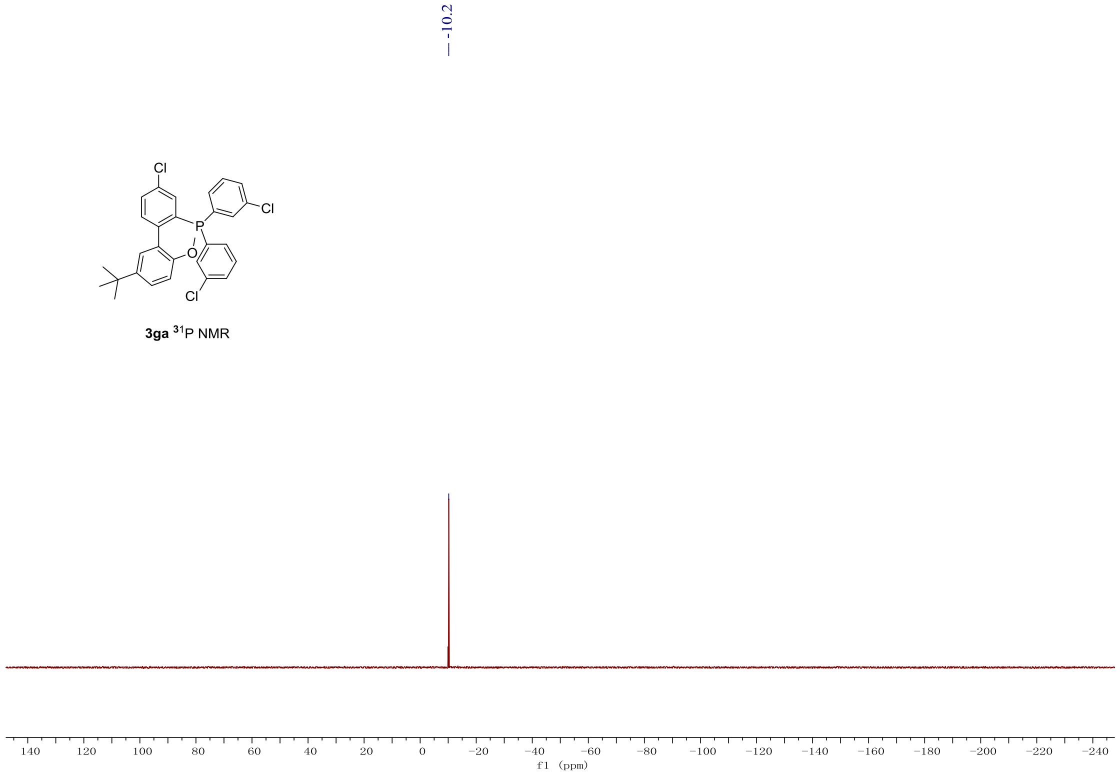

Supplementary Figure 58. ^{31}P NMR spectrum of **3ga**

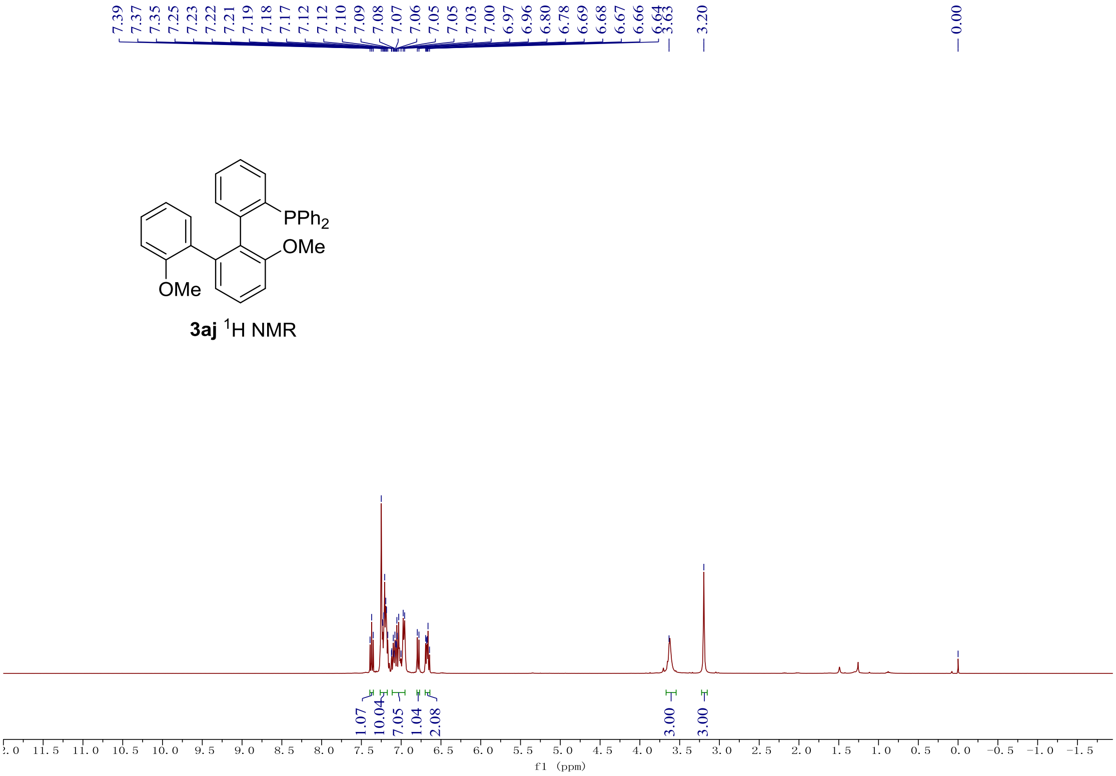

Supplementary Figure 59. ^{1}H NMR spectrum of **3aj**

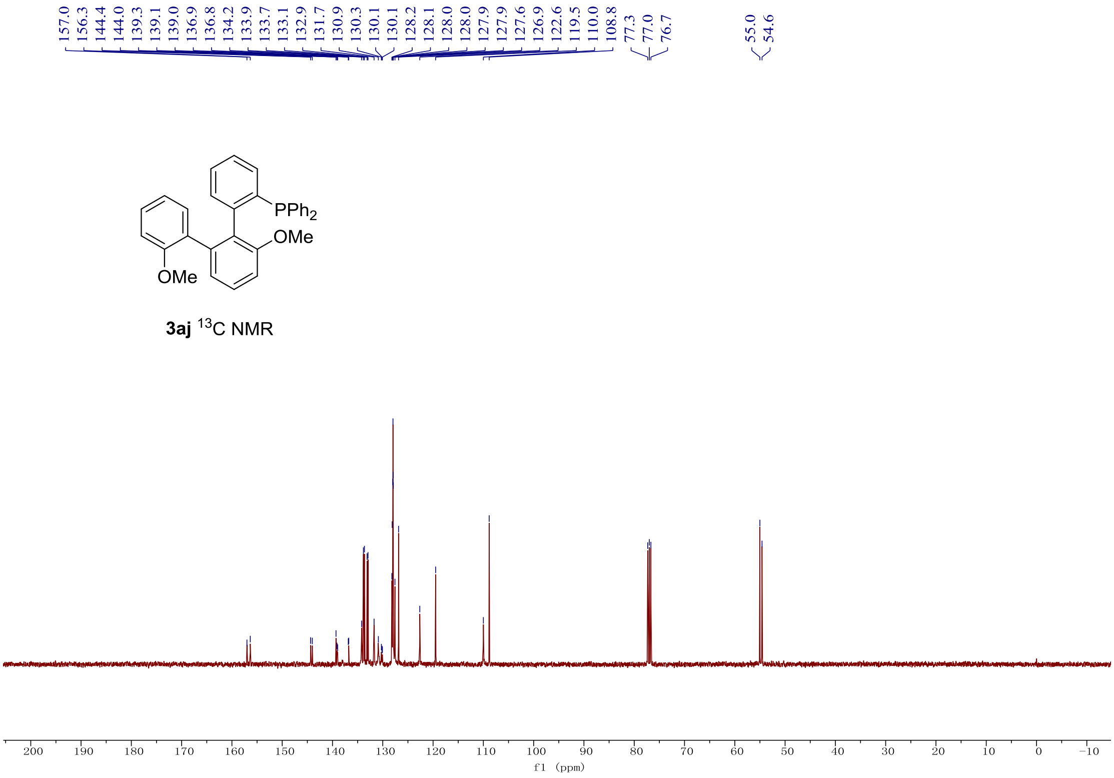

Supplementary Figure 60. ^{13}C NMR spectrum of **3aj**

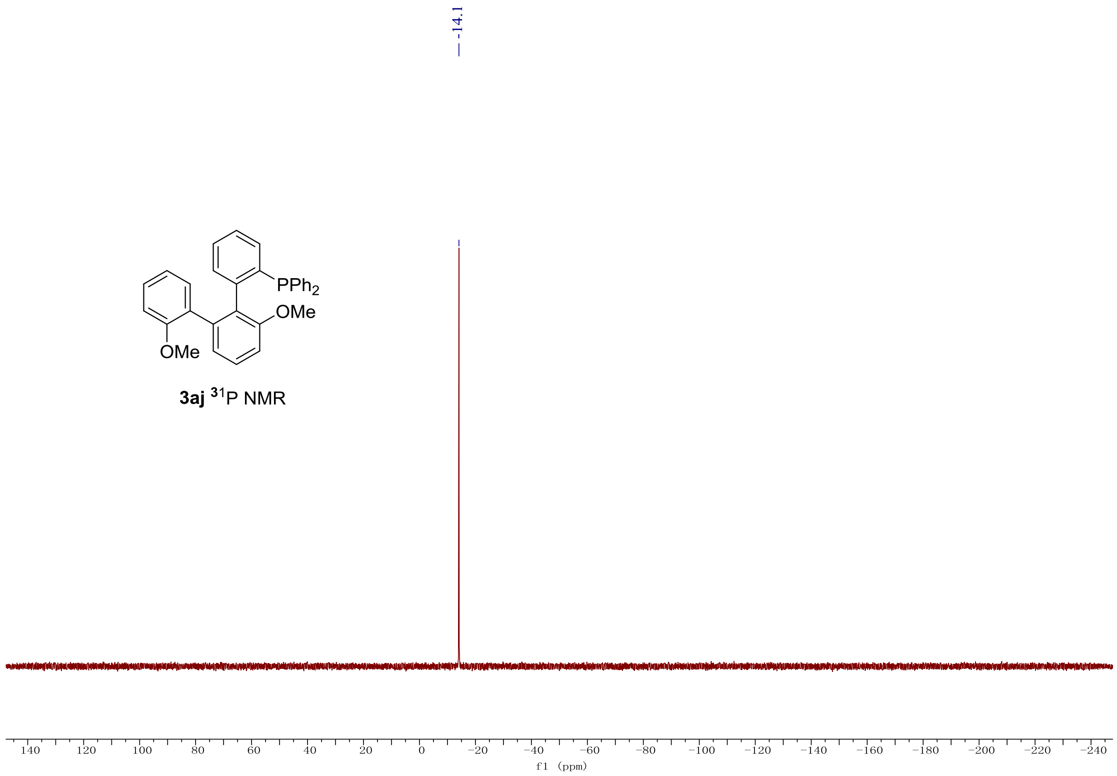

Supplementary Figure 61. ^{31}P NMR spectrum of **3aj**

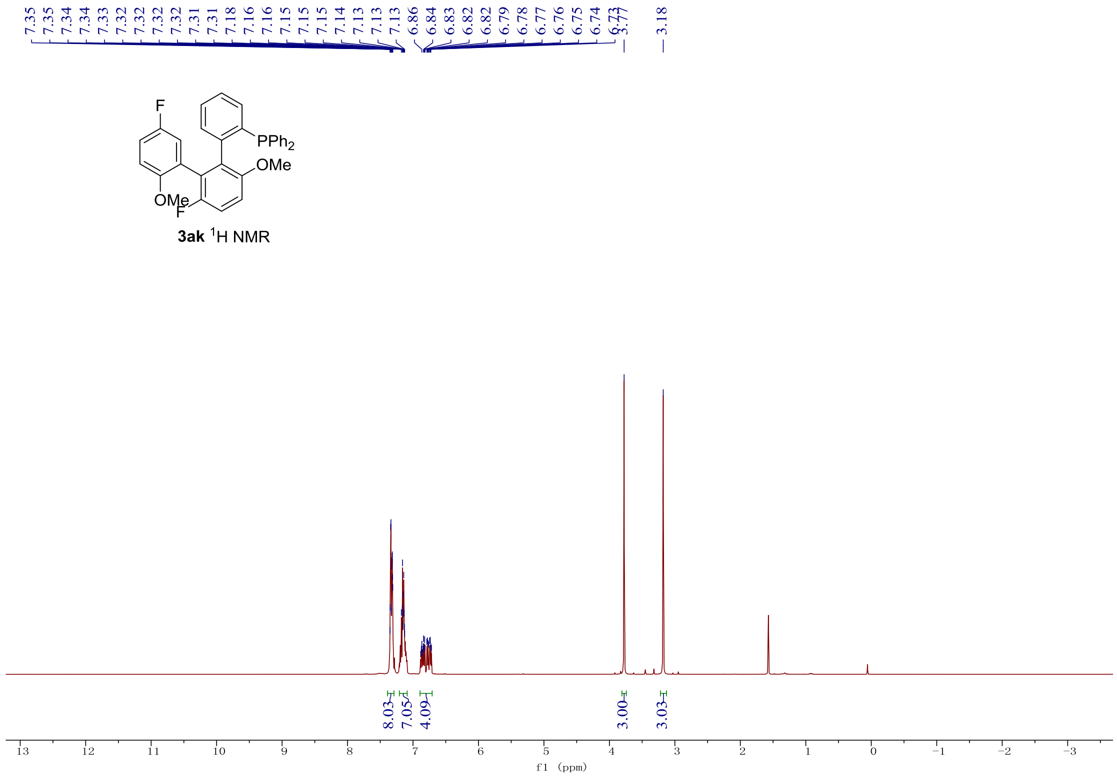

Supplementary Figure 62. ^{1}H NMR spectrum of **3ak**

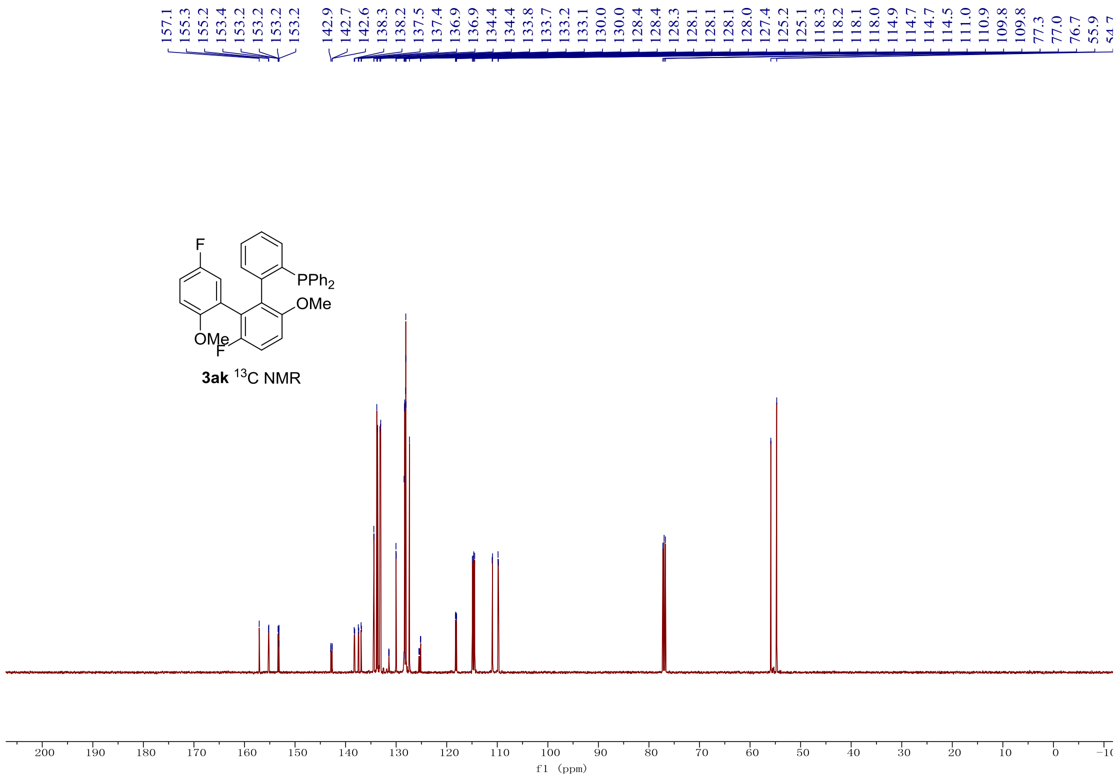

Supplementary Figure 63. ^{13}C NMR spectrum of **3ak**

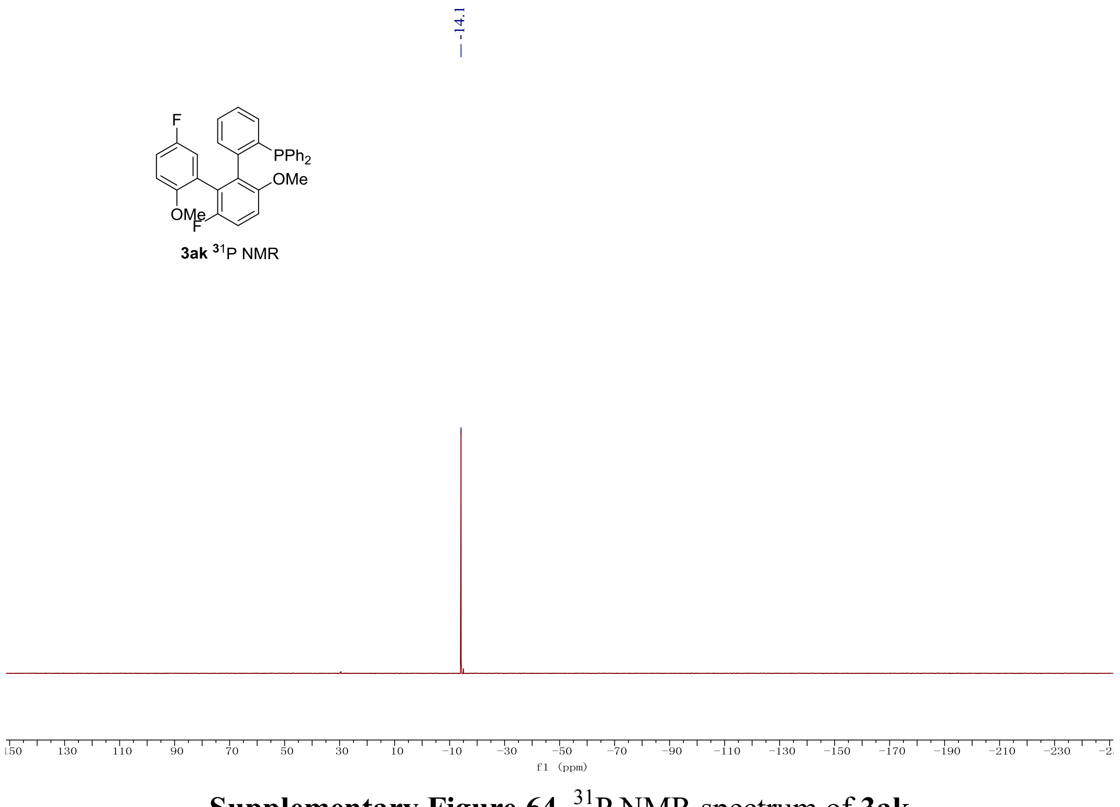

Supplementary Figure 64. ^{31}P NMR spectrum of **3ak**

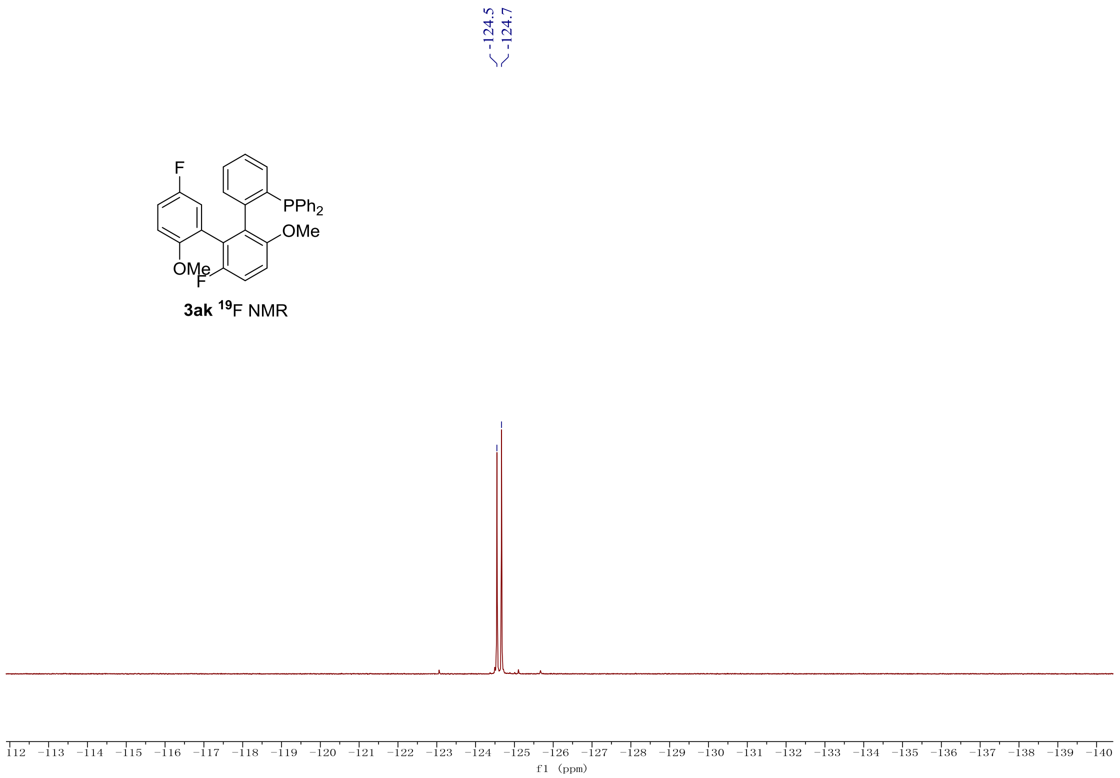

Supplementary Figure 65. ^{19}F NMR spectrum of **3ak**

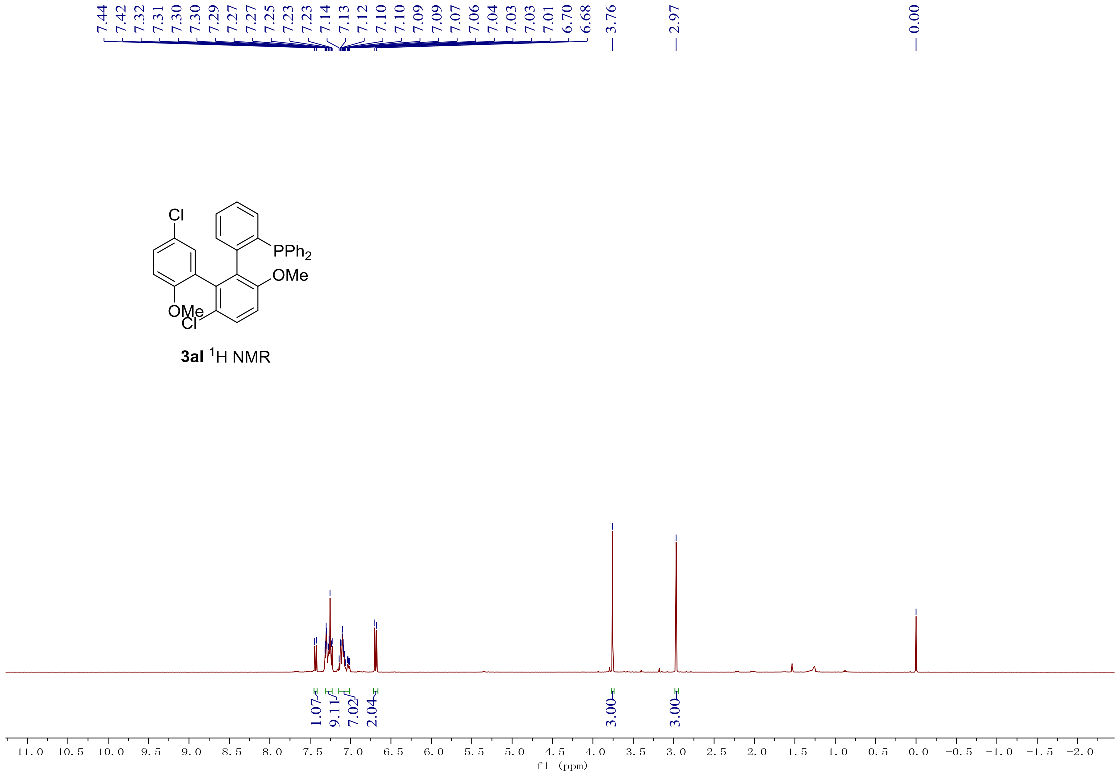

Supplementary Figure 66. ^{1}H NMR spectrum of **3al**

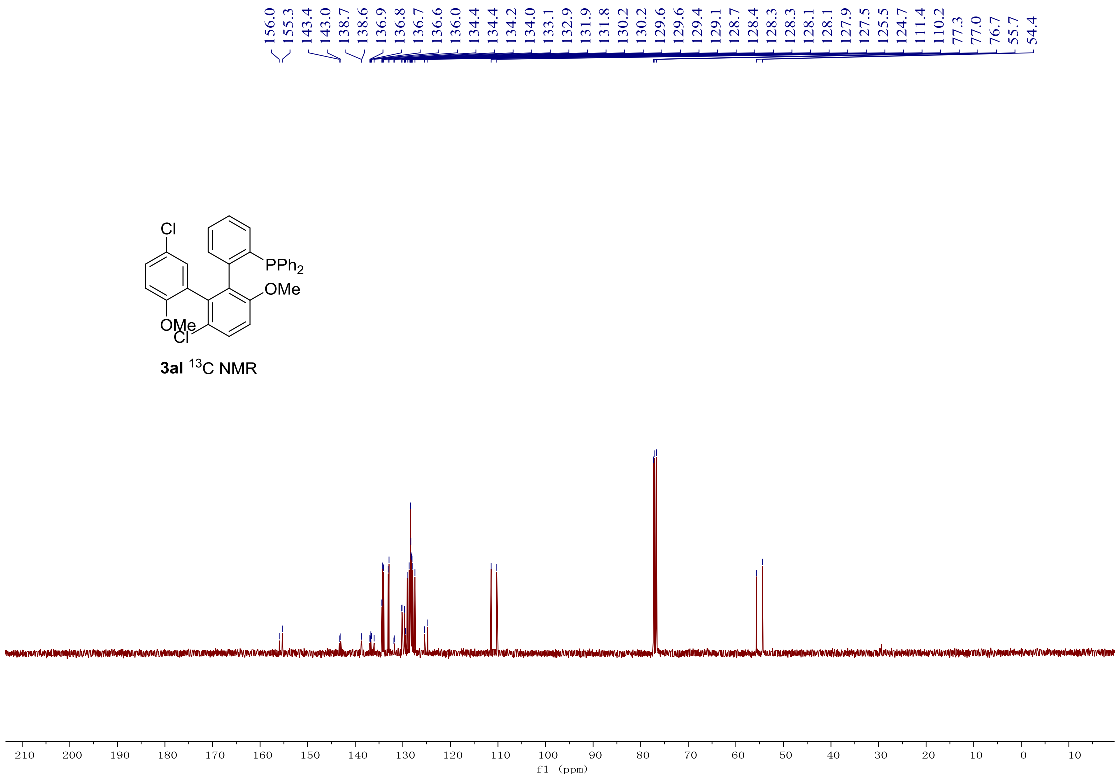

Supplementary Figure 67. ^{13}C NMR spectrum of **3al**

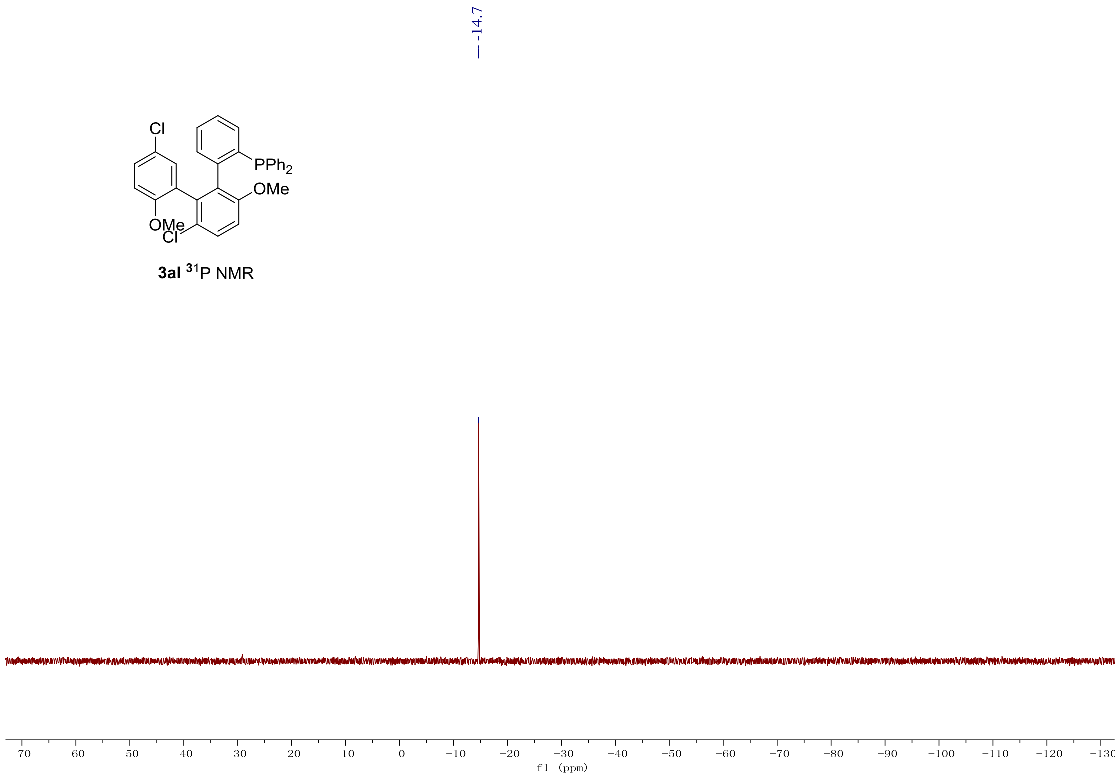

Supplementary Figure 68. ^{31}P NMR spectrum of **3al**

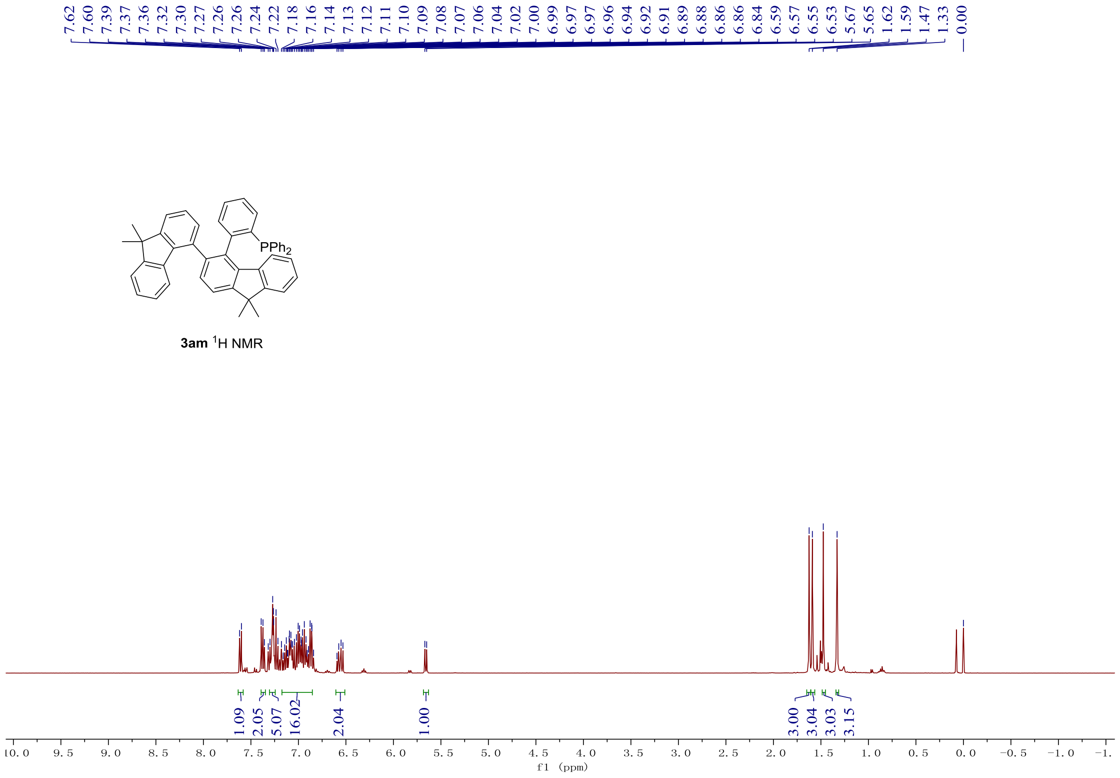

Supplementary Figure 69. ^{1}H NMR spectrum of **3am**

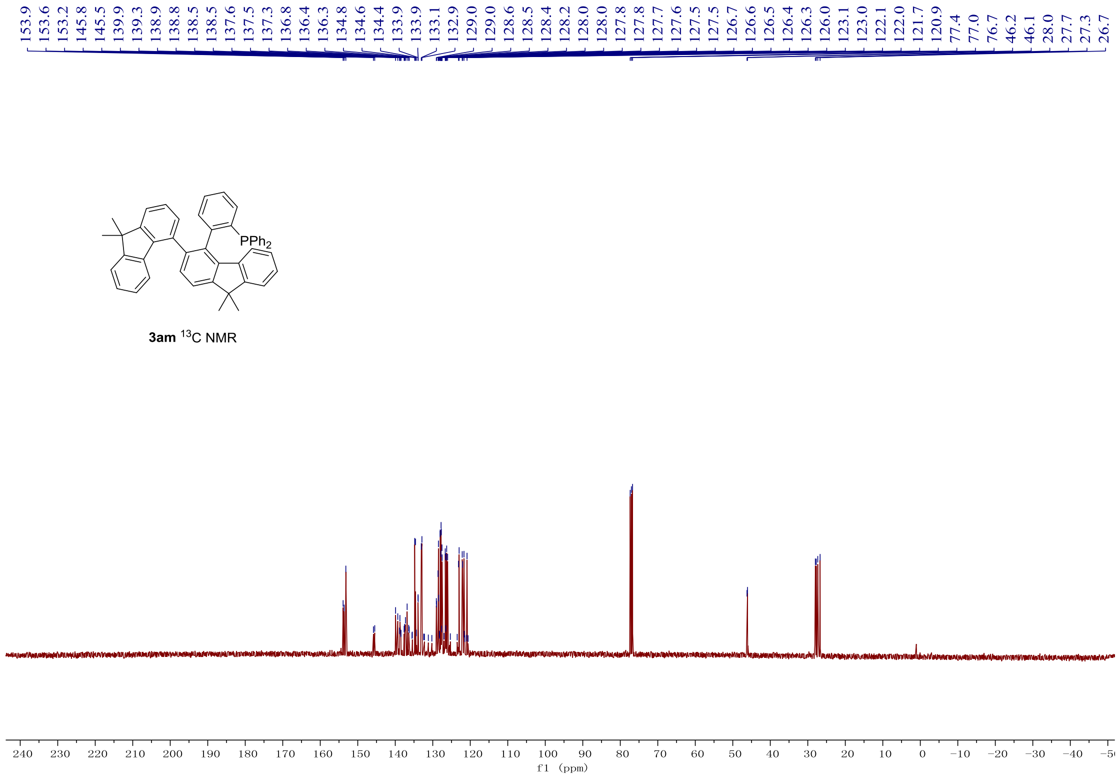

Supplementary Figure 70. ^{13}C NMR spectrum of **3am**

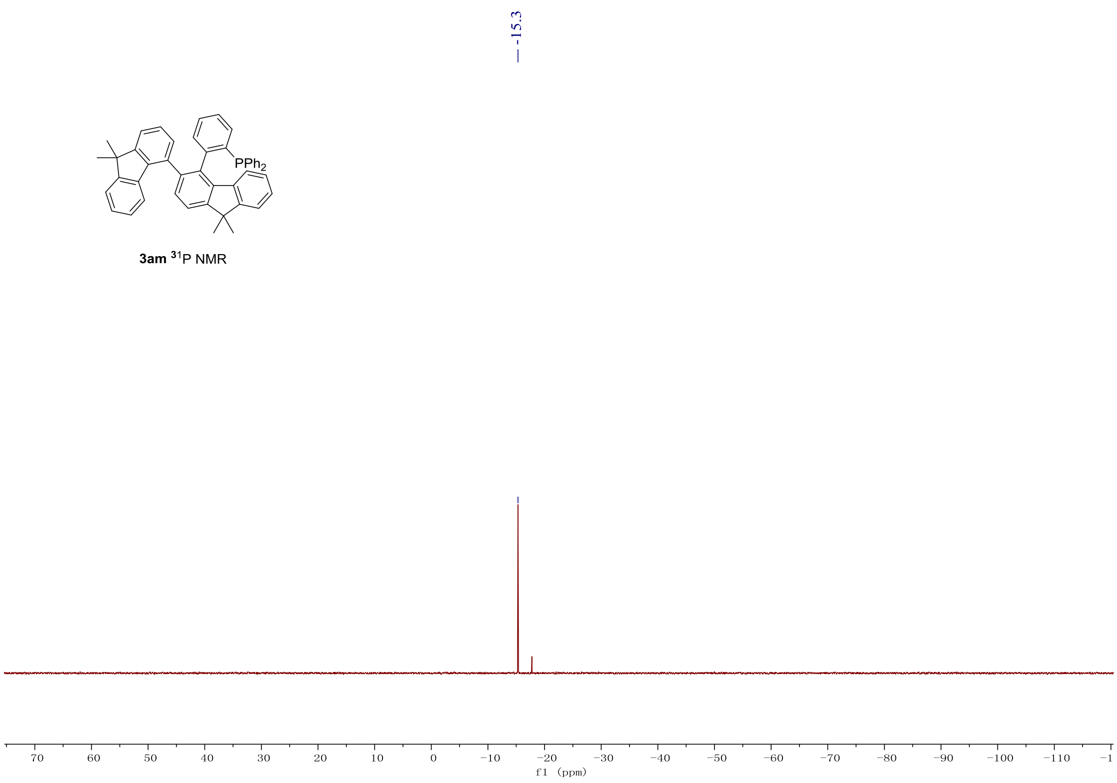

Supplementary Figure 71. ^{31}P NMR spectrum of **3am**

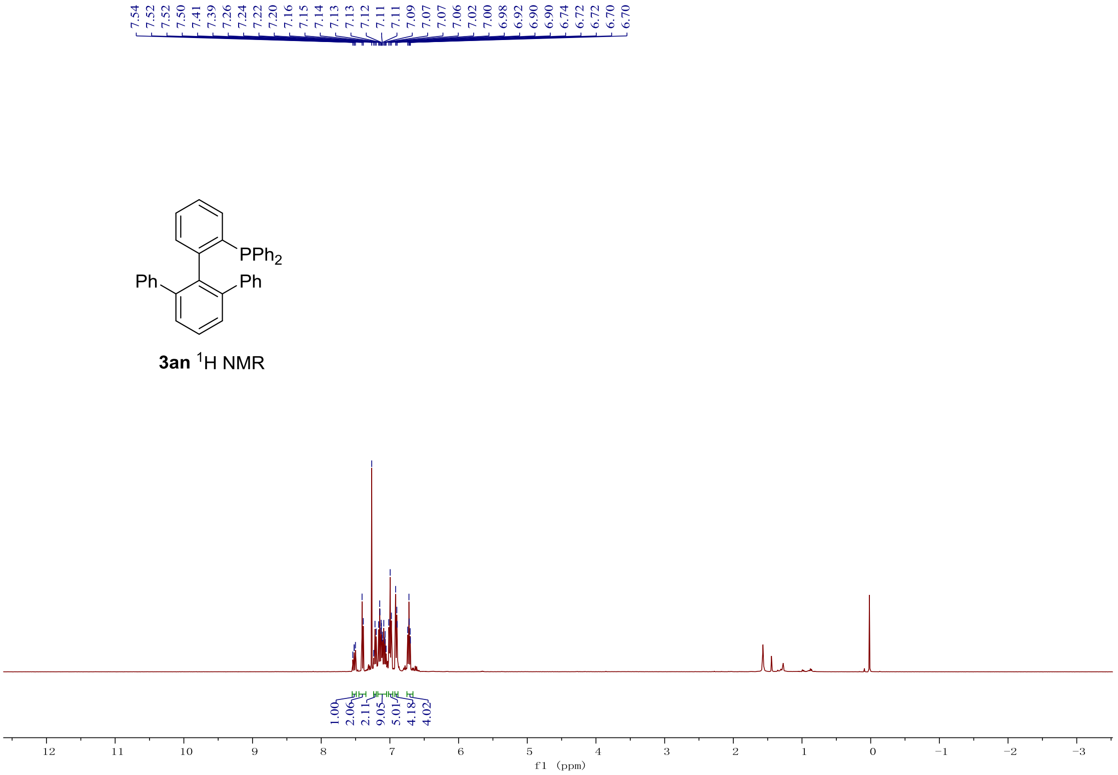

Supplementary Figure 72. ^{1}H NMR spectrum of **3an**

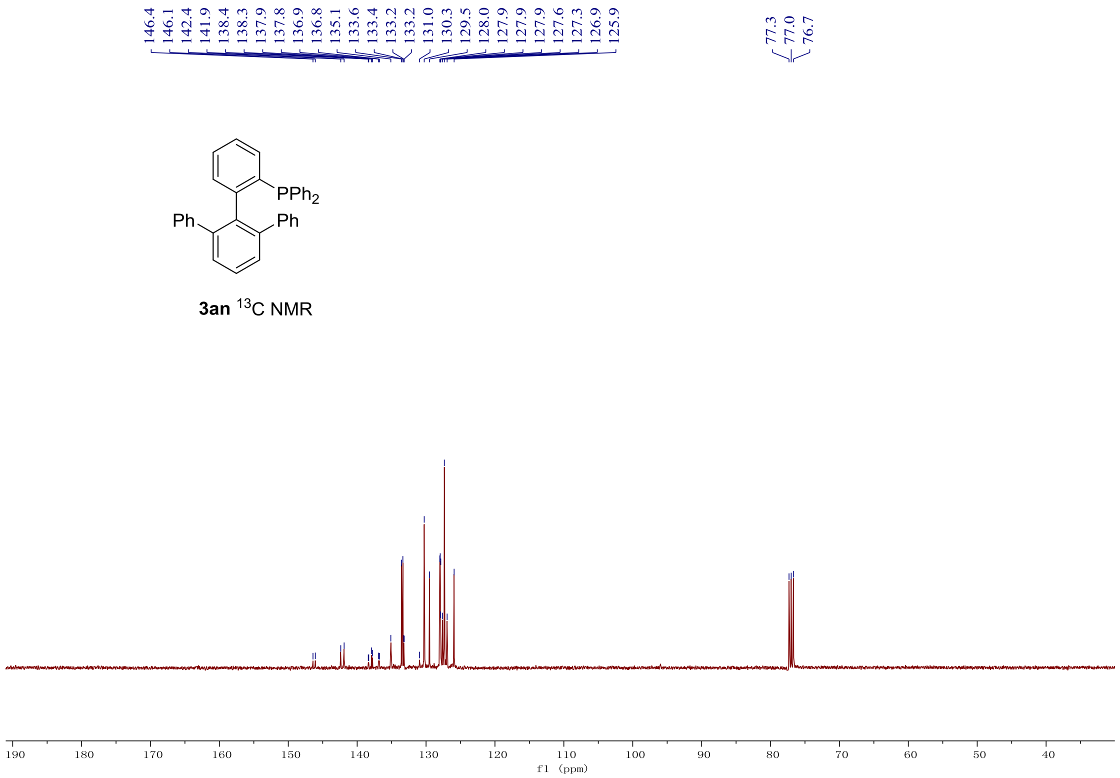

Supplementary Figure 73. ^{13}C NMR spectrum of **3an**

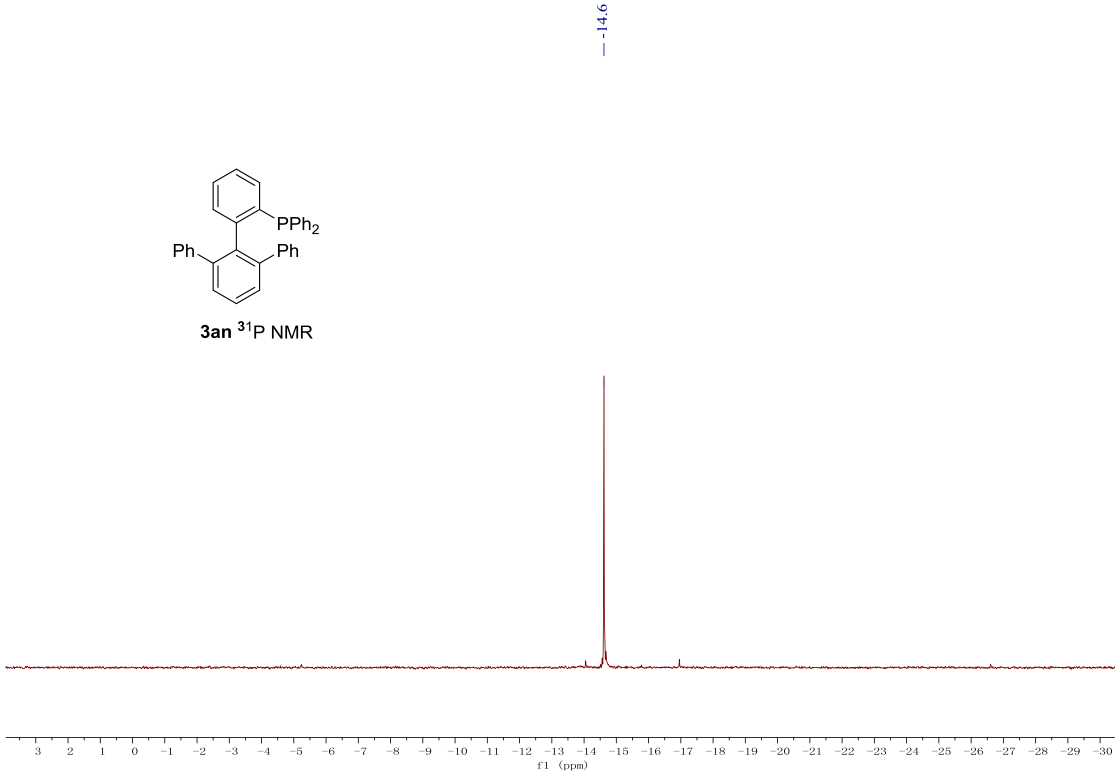

Supplementary Figure 74. ^{31}P NMR spectrum of **3an**

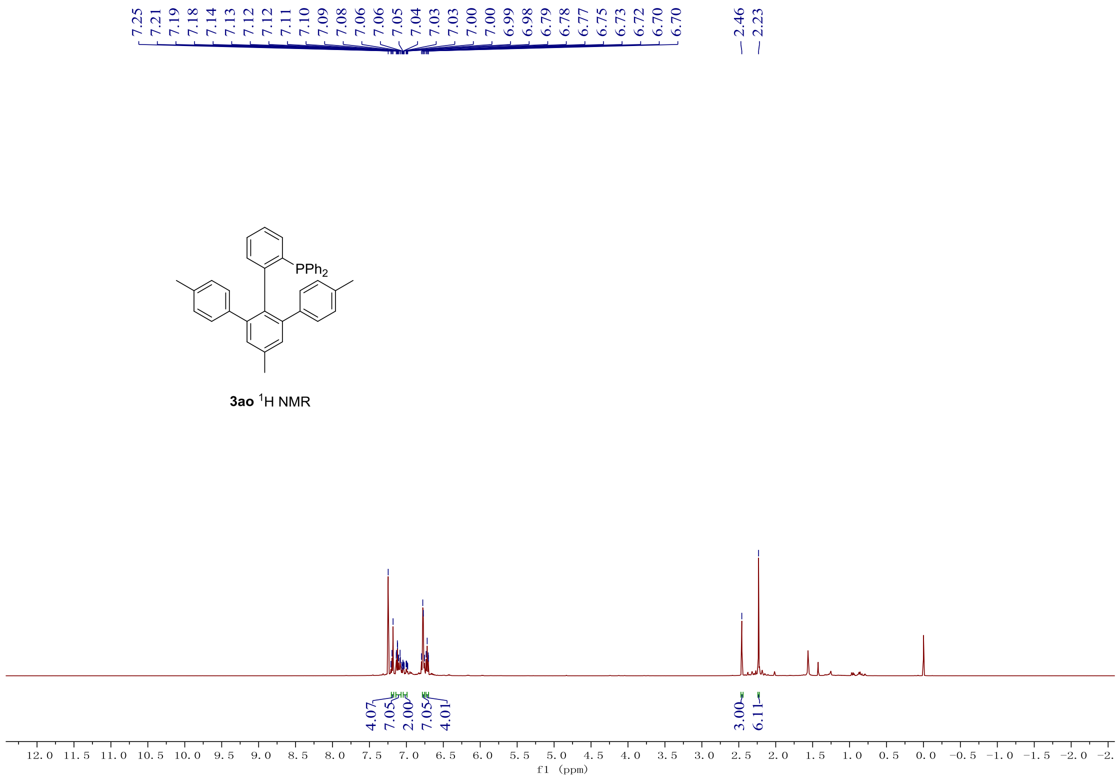

Supplementary Figure 75. ^{1}H NMR spectrum of **3ao**

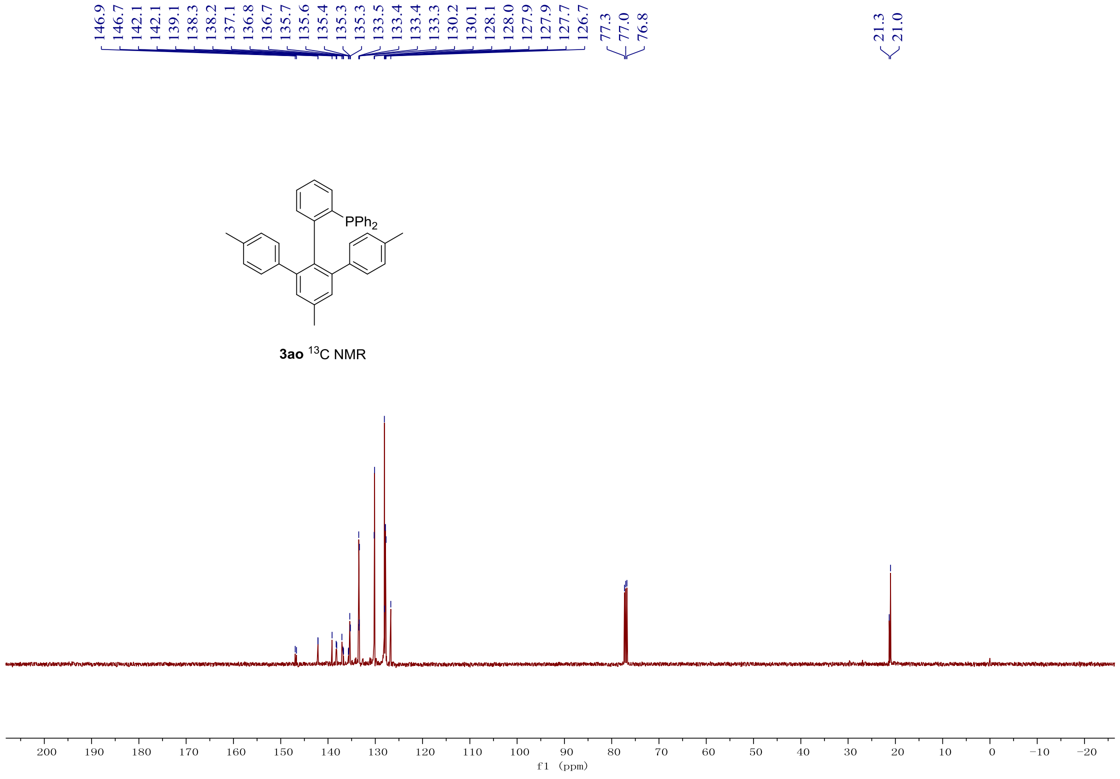

Supplementary Figure 76. ^{13}C NMR spectrum of **3ao**

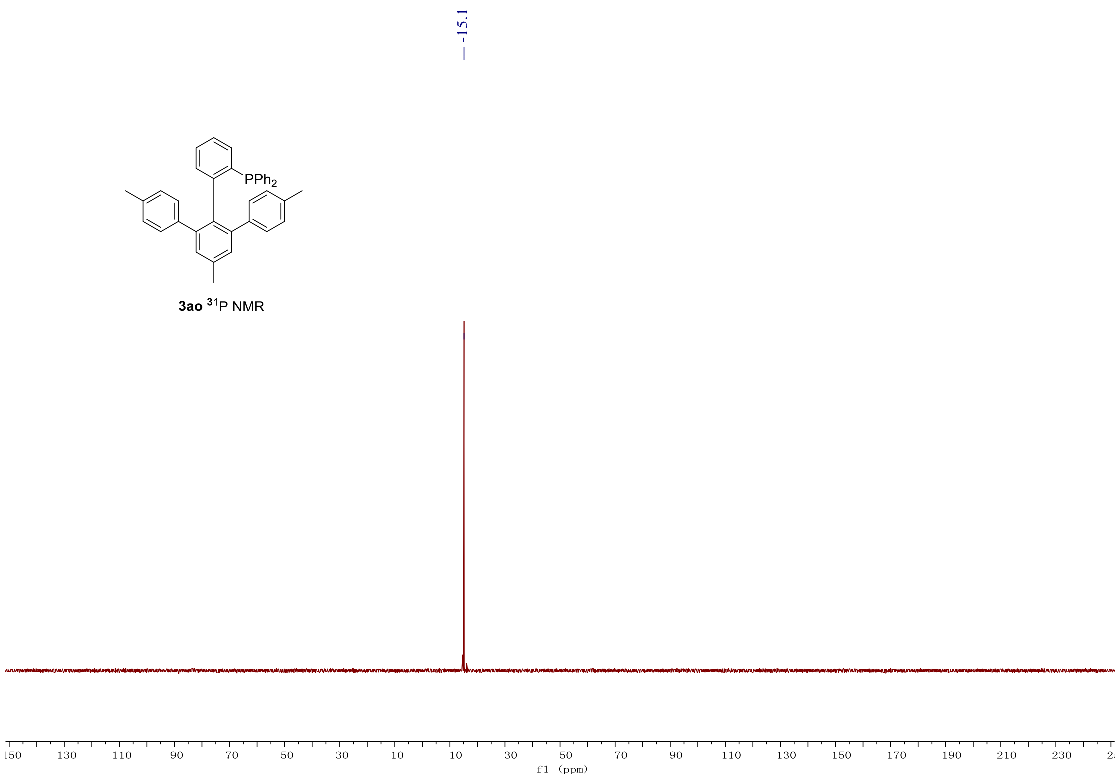

Supplementary Figure 77. ^{31}P NMR spectrum of **3ao**

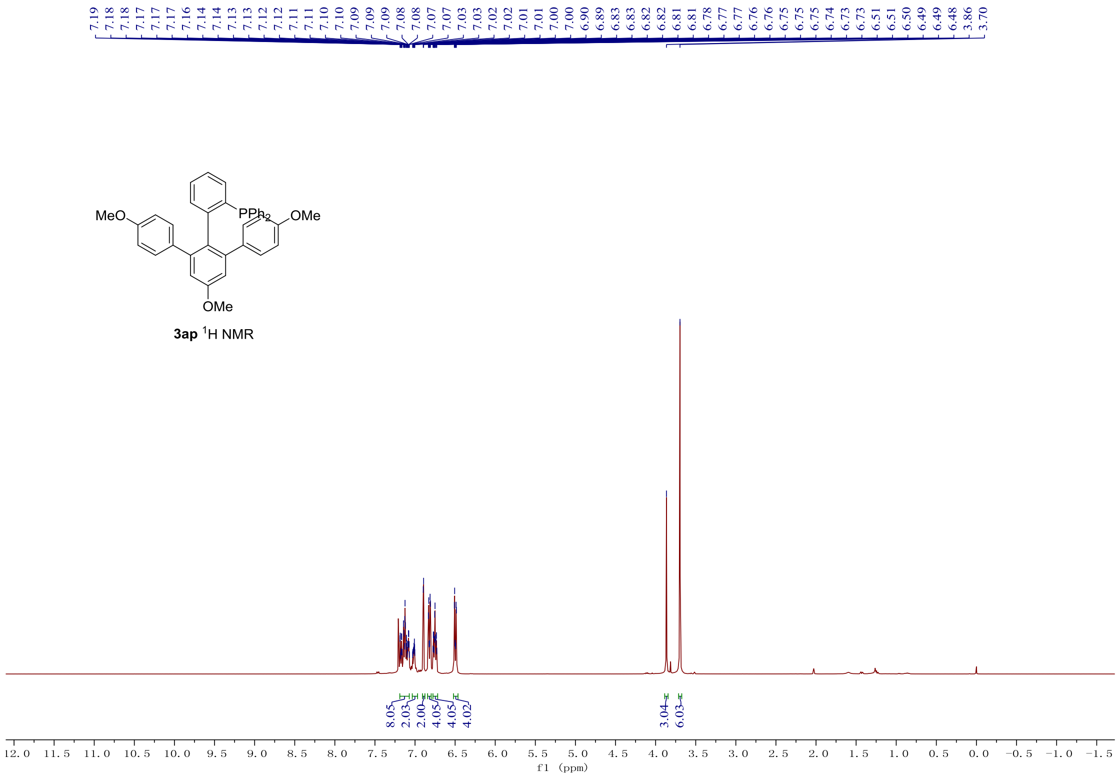

Supplementary Figure 78. ^{1}H NMR spectrum of **3ap**

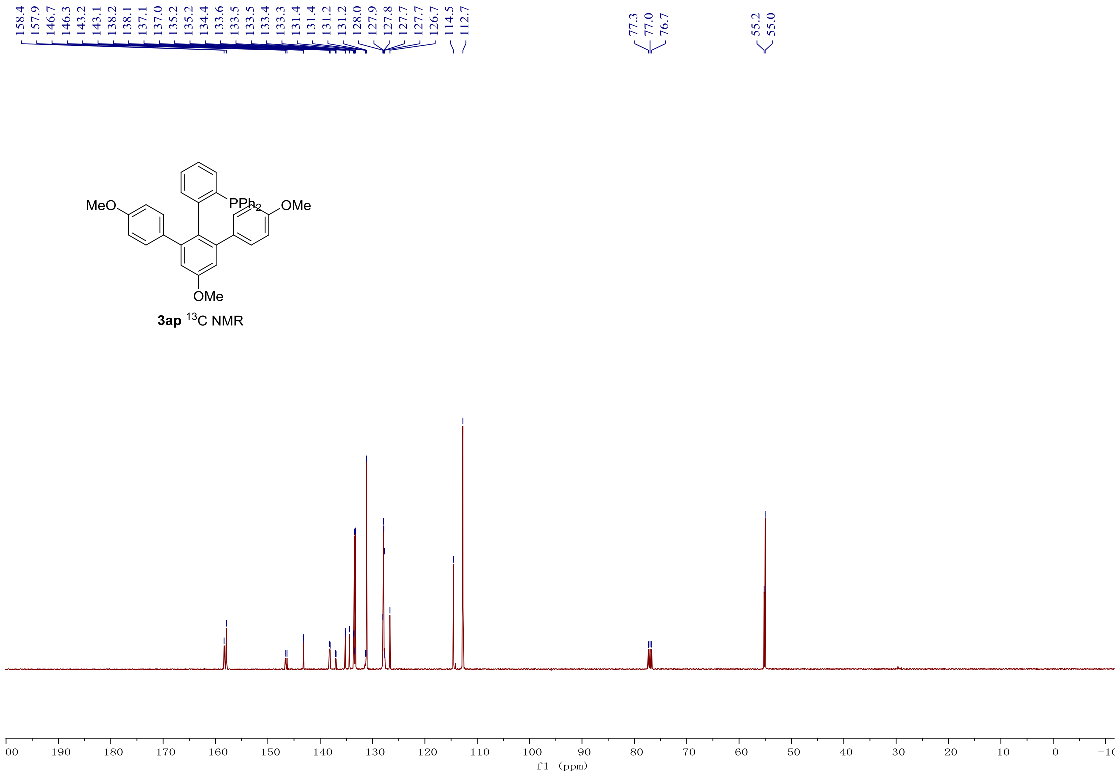

Supplementary Figure 79. ^{13}C NMR spectrum of **3ap**

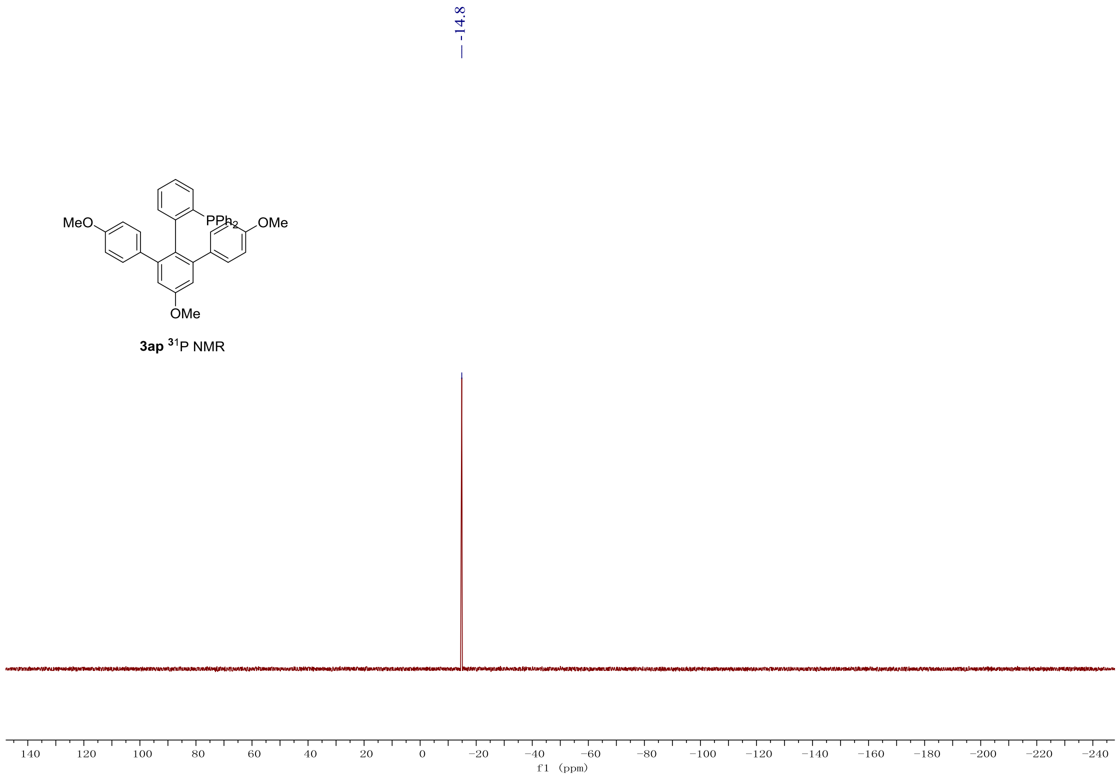

Supplementary Figure 80. ^{31}P NMR spectrum of **3ap**

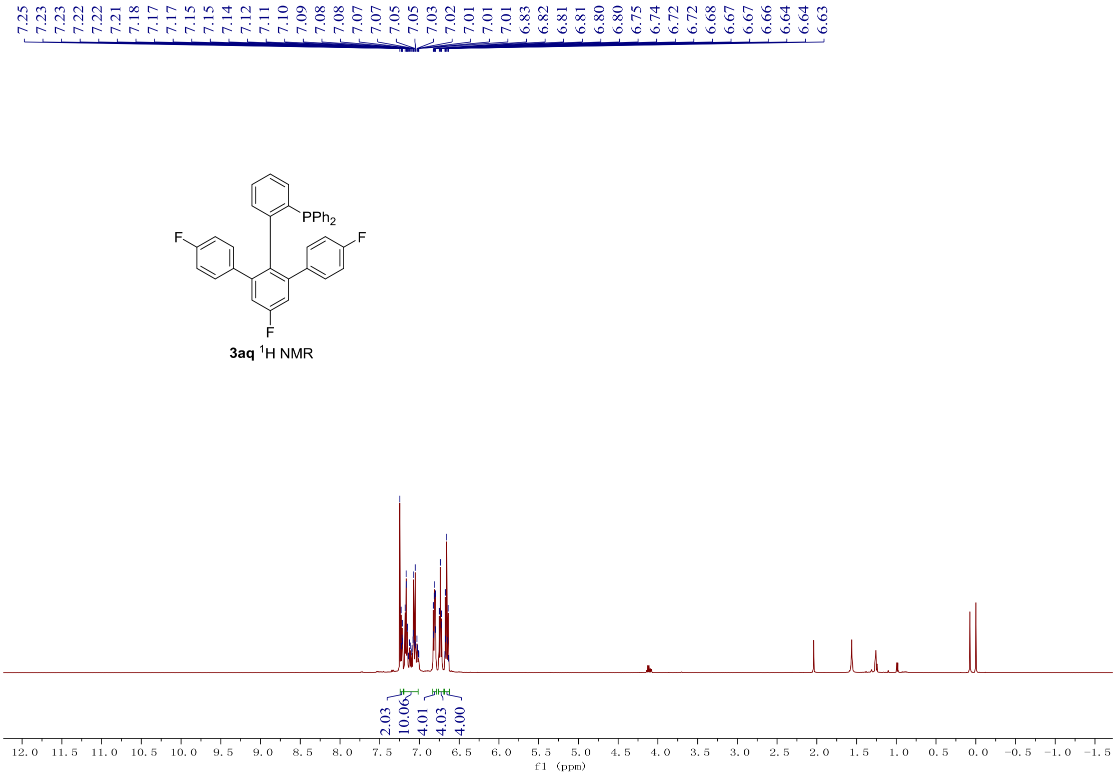

Supplementary Figure 81. ^{1}H NMR spectrum of **3aq**

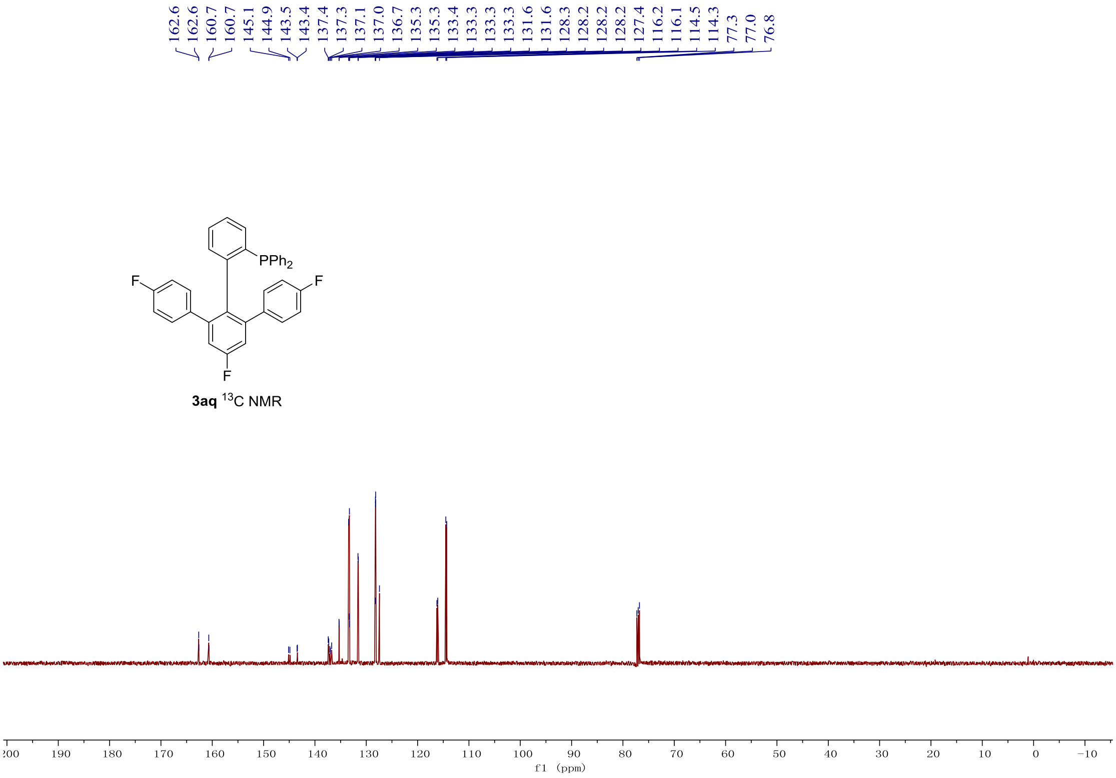

Supplementary Figure 82. ^{13}C NMR spectrum of **3aq**

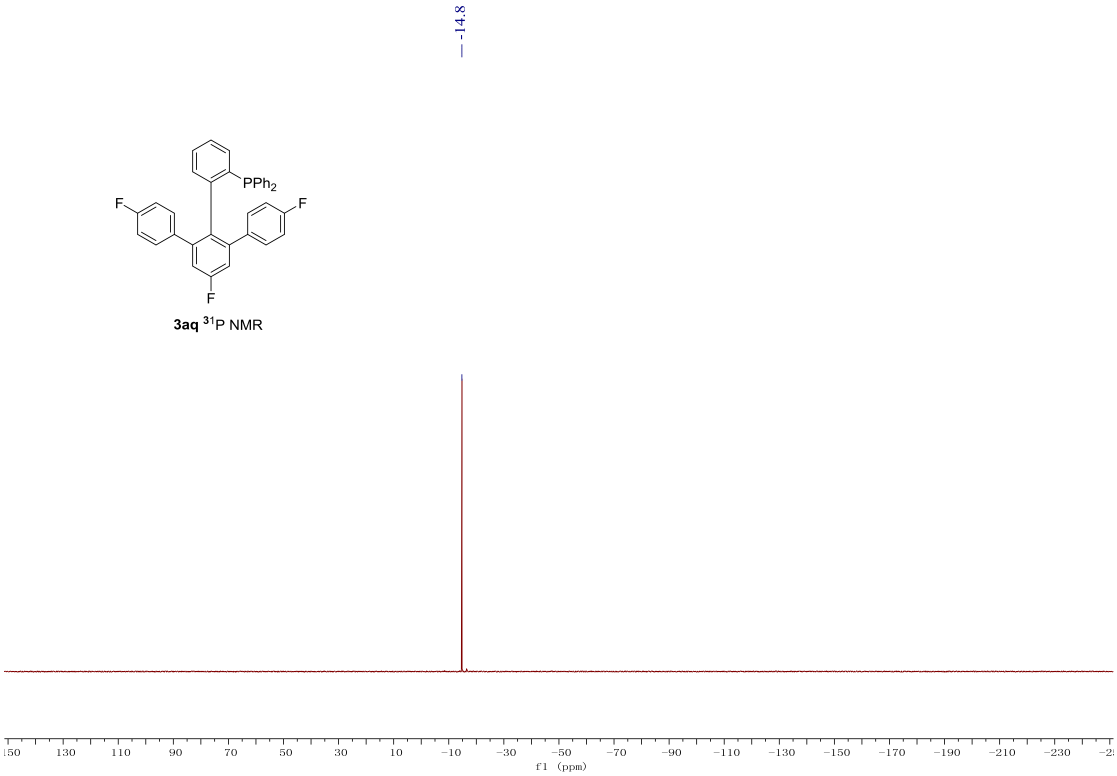

Supplementary Figure 83. ^{31}P NMR spectrum of **3aq**

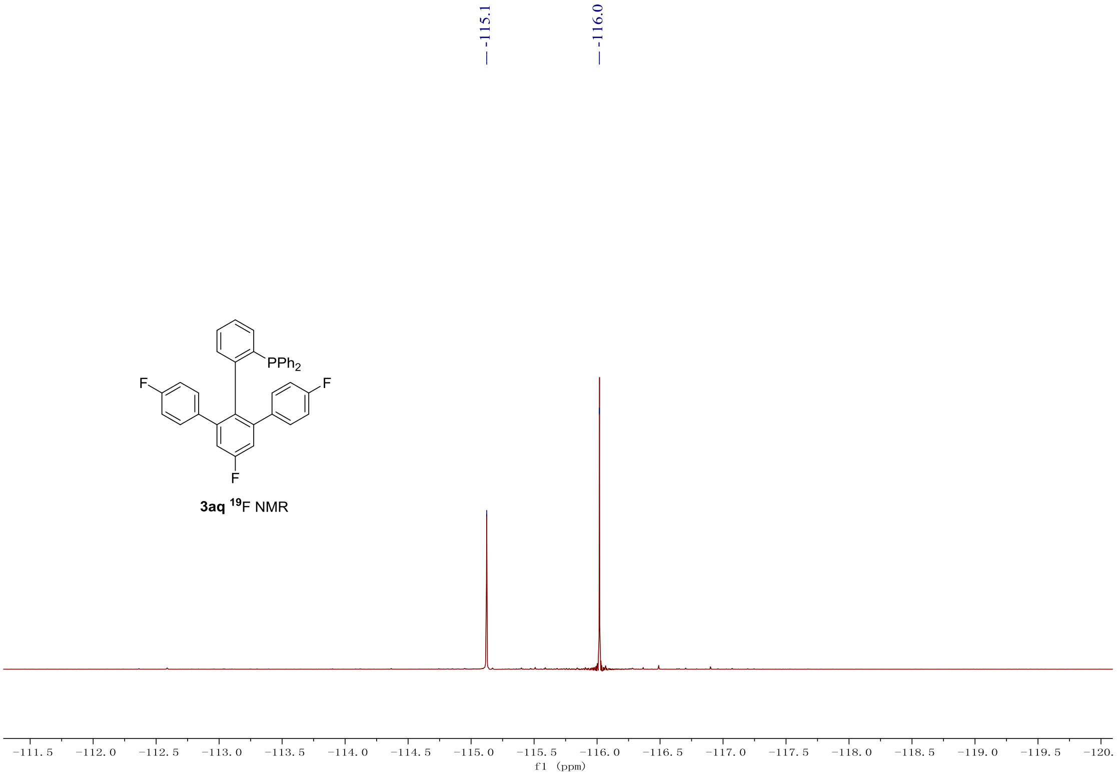

Supplementary Figure 84. ^{19}P NMR spectrum of **3aq**

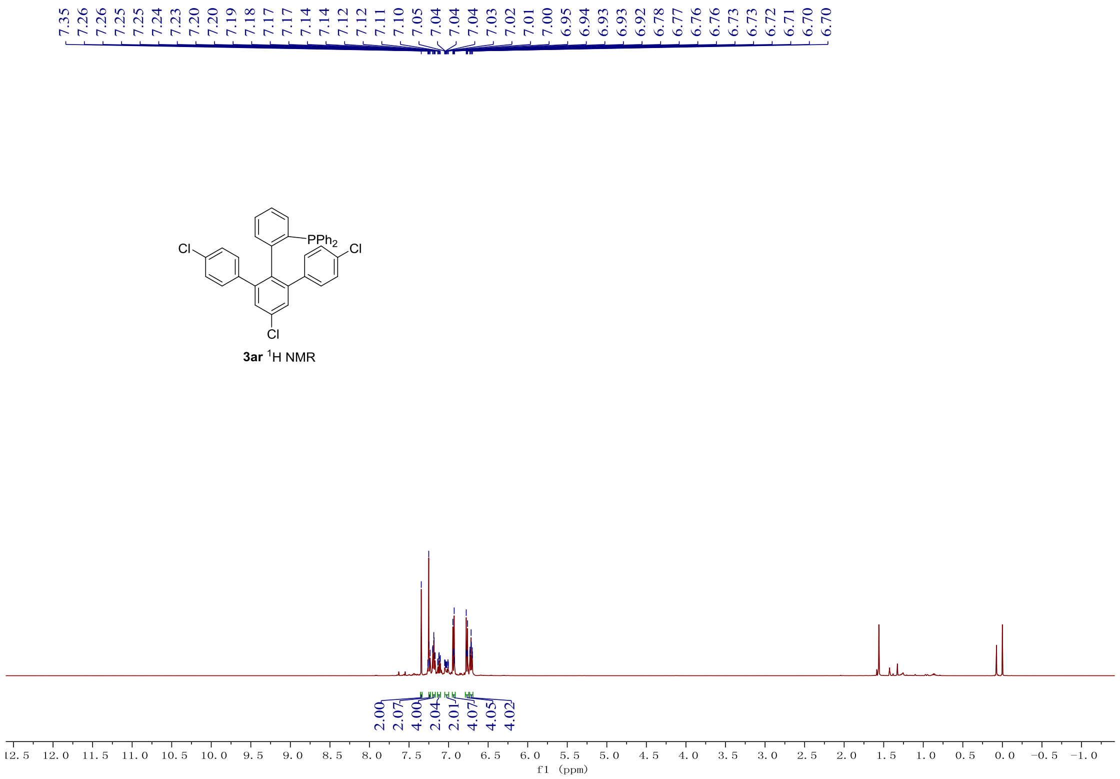

Supplementary Figure 85. ^{1}H NMR spectrum of **3af**

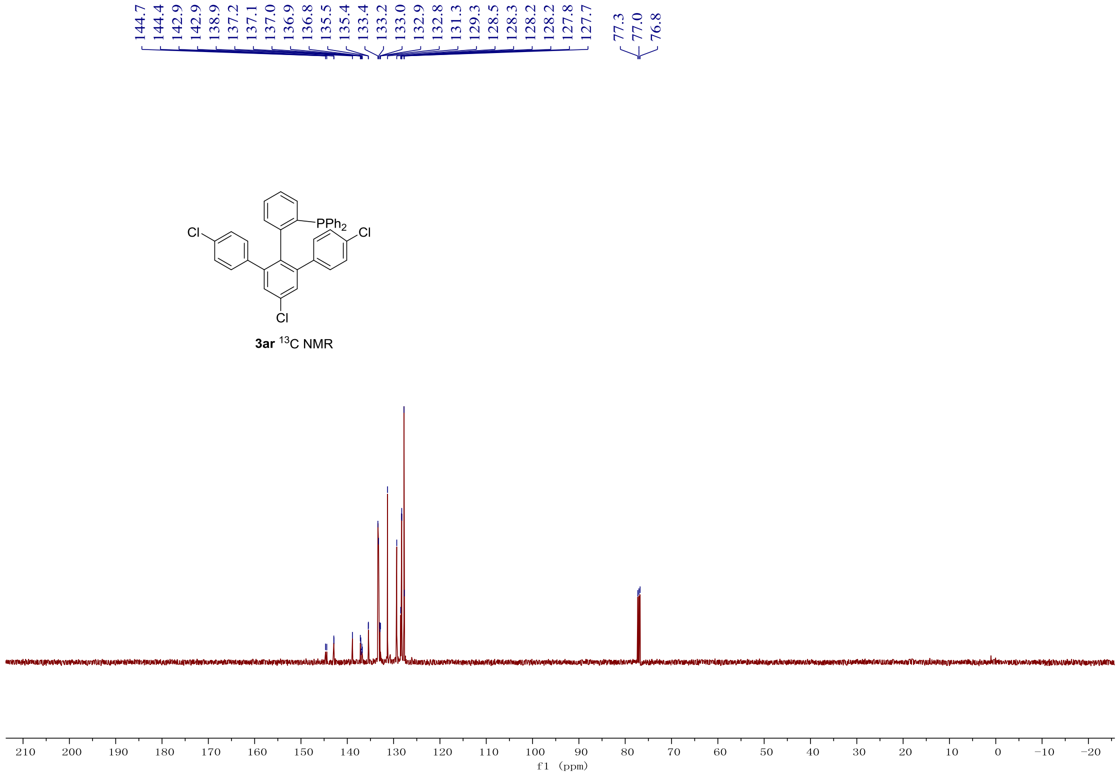

Supplementary Figure 86. ^{13}C NMR spectrum of **3af**

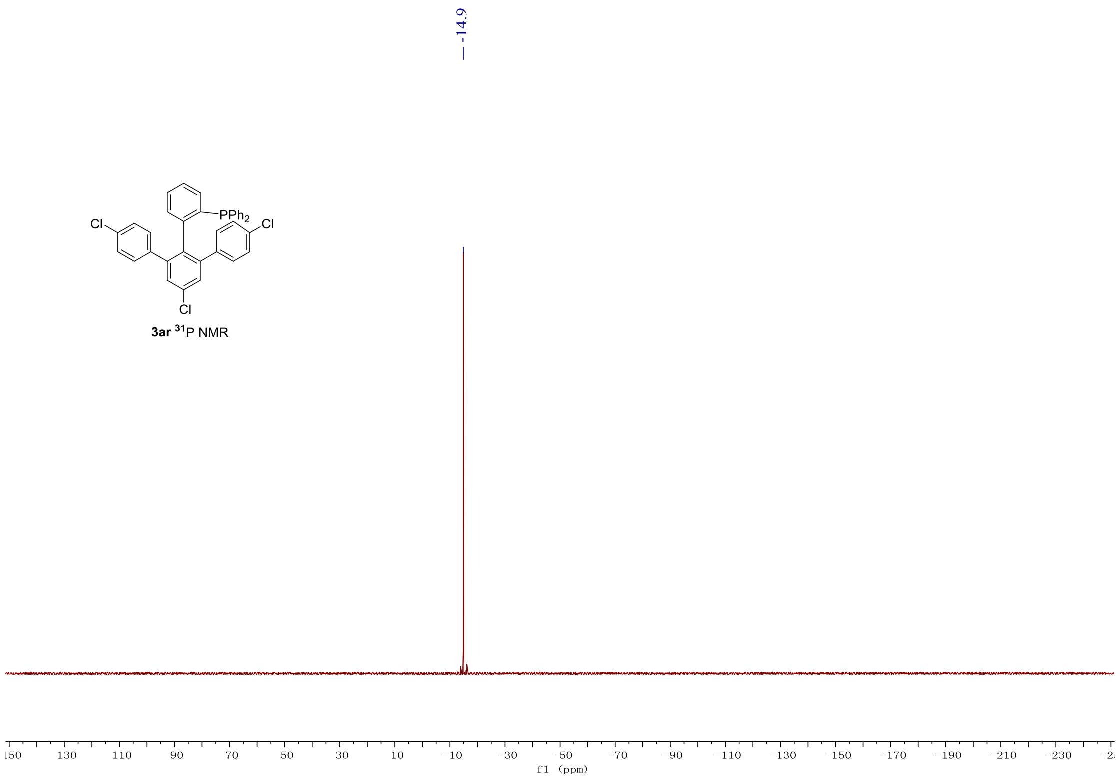

Supplementary Figure 87. ^{31}P NMR spectrum of **3af**

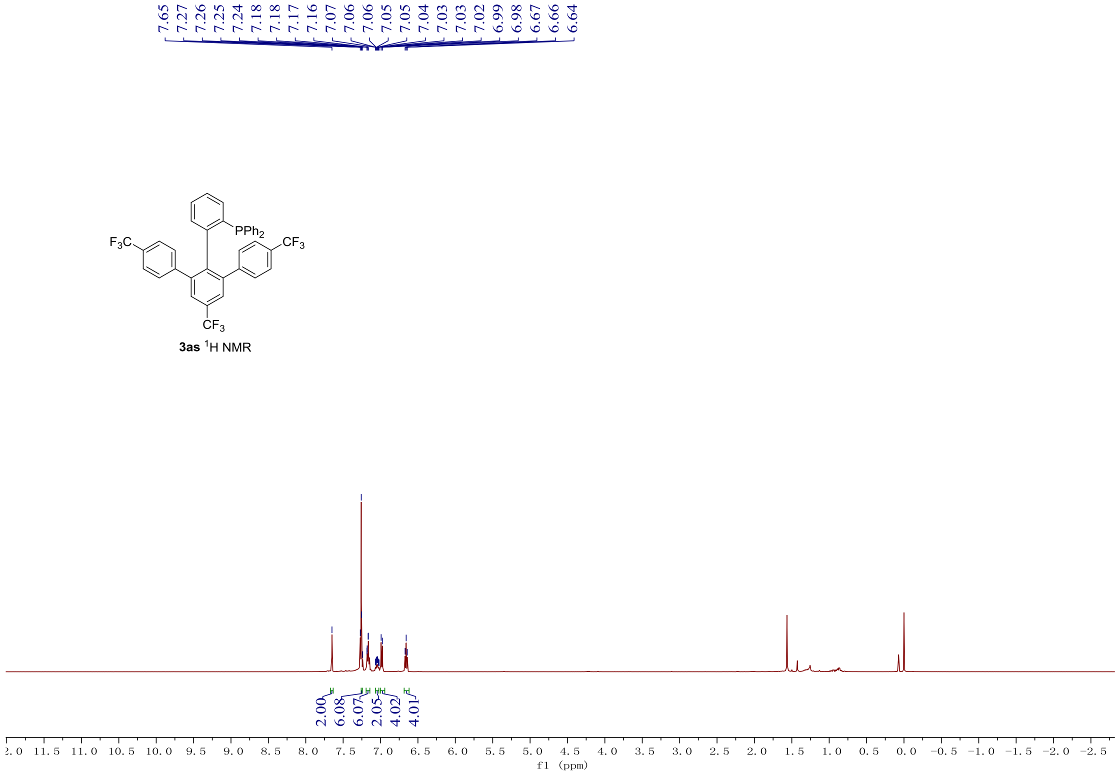

Supplementary Figure 88. ^{1}H NMR spectrum of **3as**

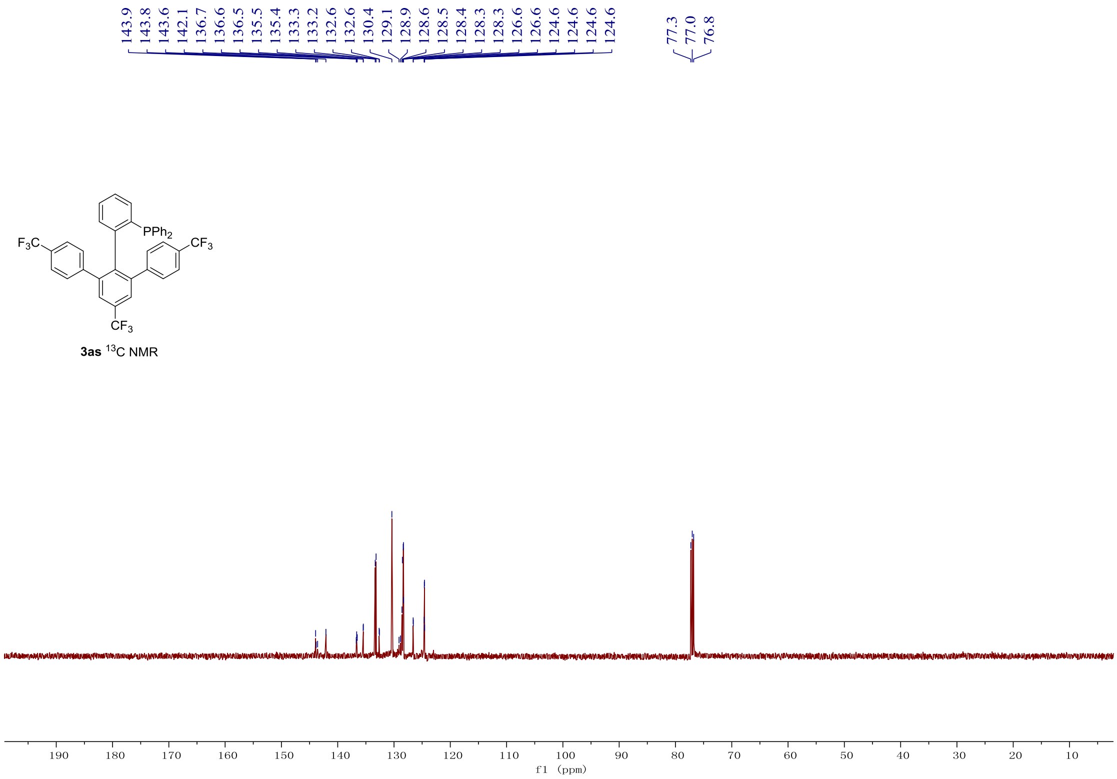

Supplementary Figure 89. ^{13}C NMR spectrum of **3as**

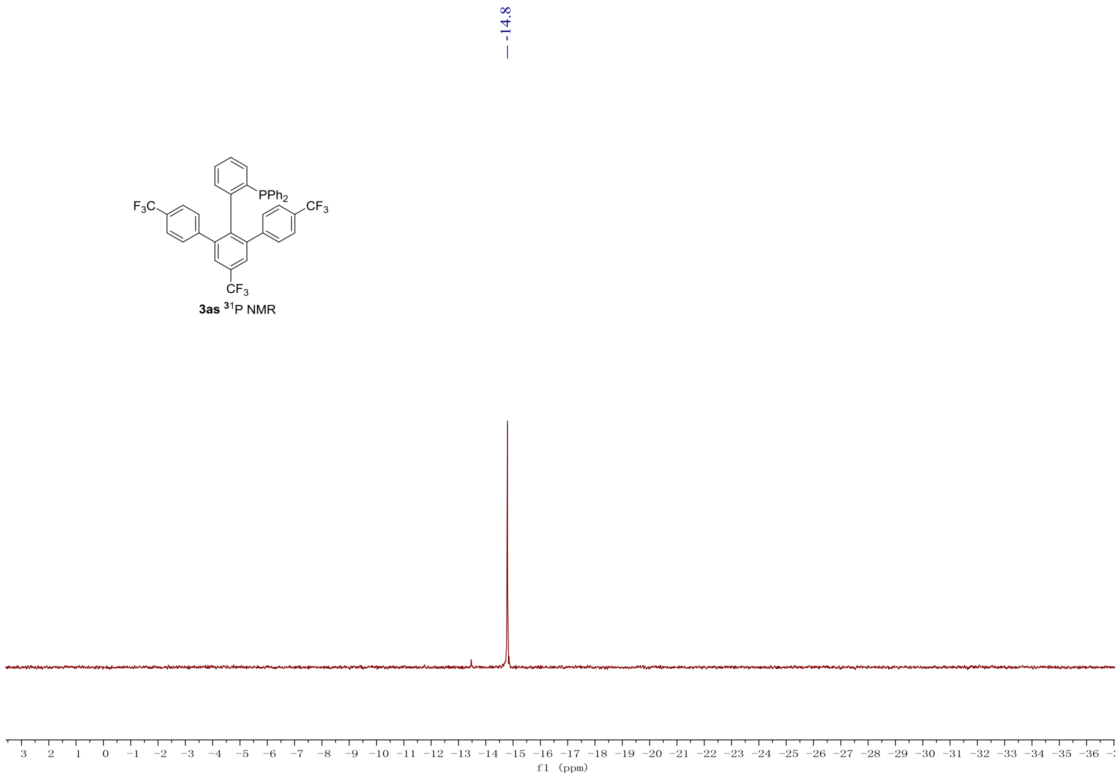

Supplementary Figure 90. ^{31}P NMR spectrum of **3as**

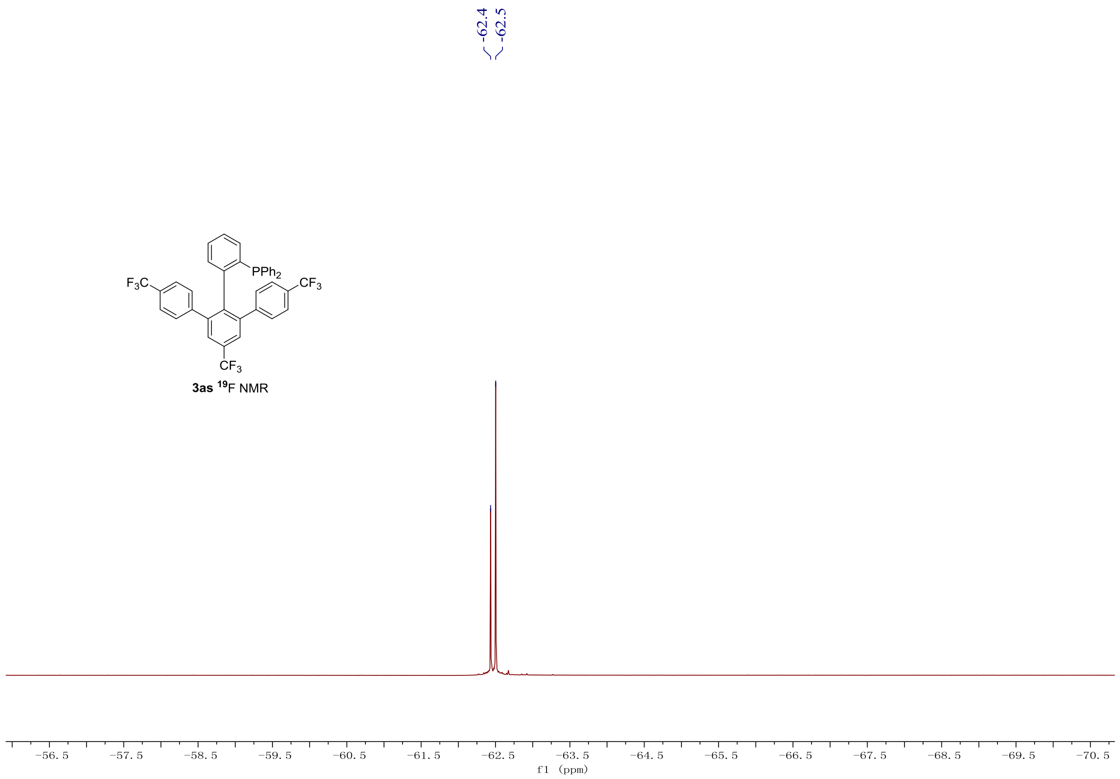

Supplementary Figure 91. ^{19}P NMR spectrum of **3as**

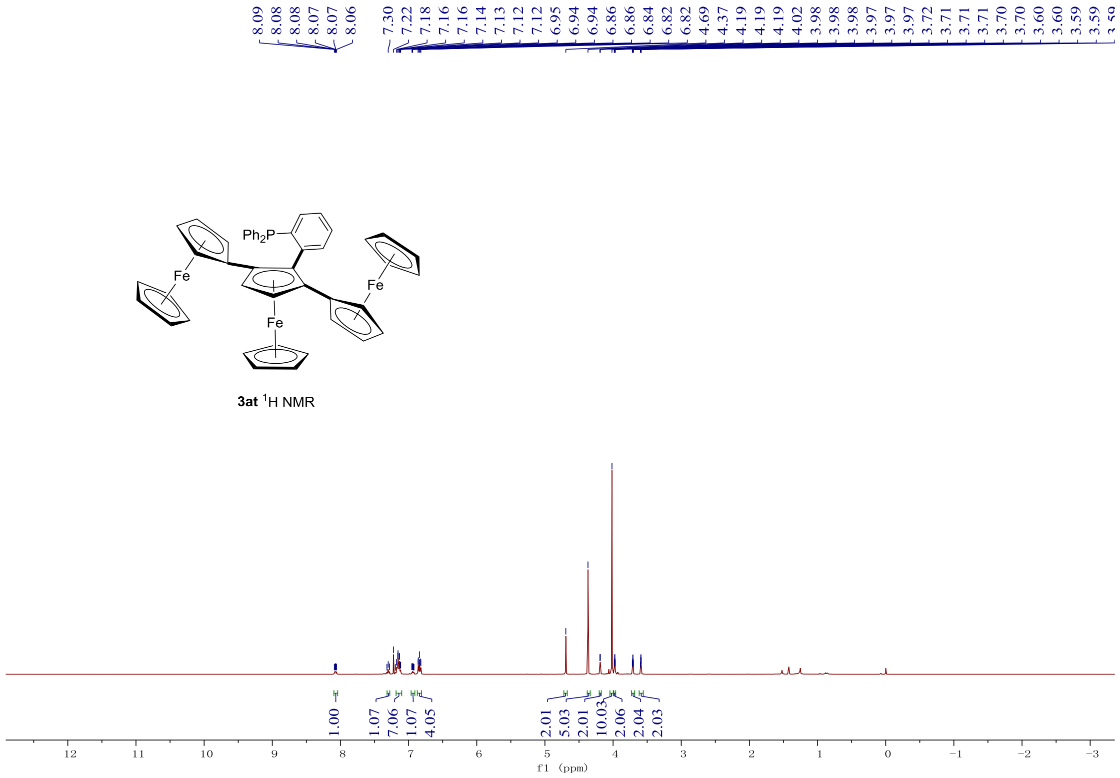

Supplementary Figure 92. ^{1}H NMR spectrum of **3af**

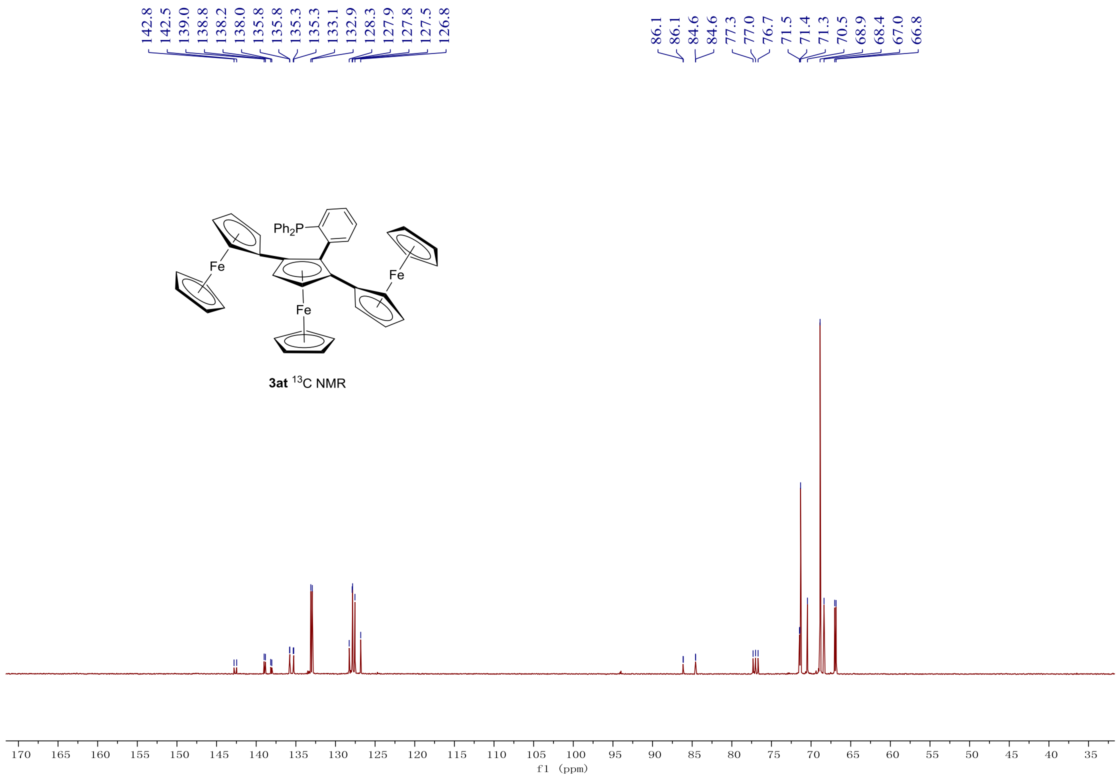

Supplementary Figure 93. ^{13}C NMR spectrum of **3af**

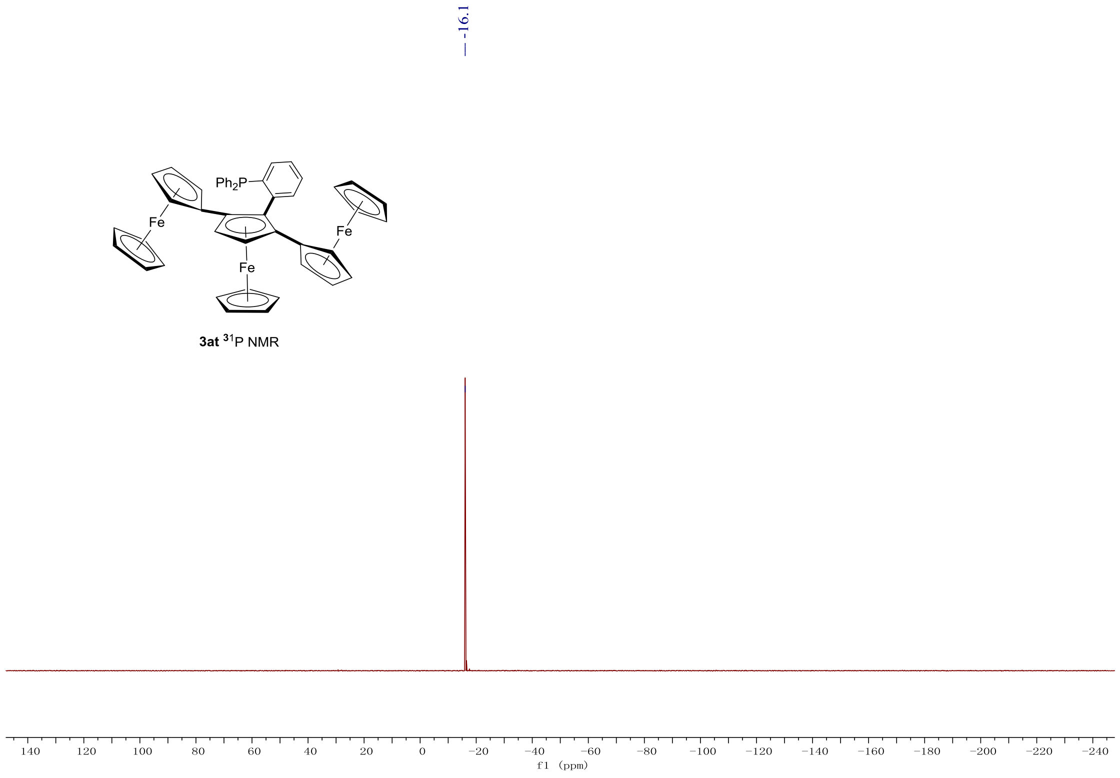

Supplementary Figure 94. ^{31}P NMR spectrum of **3af**

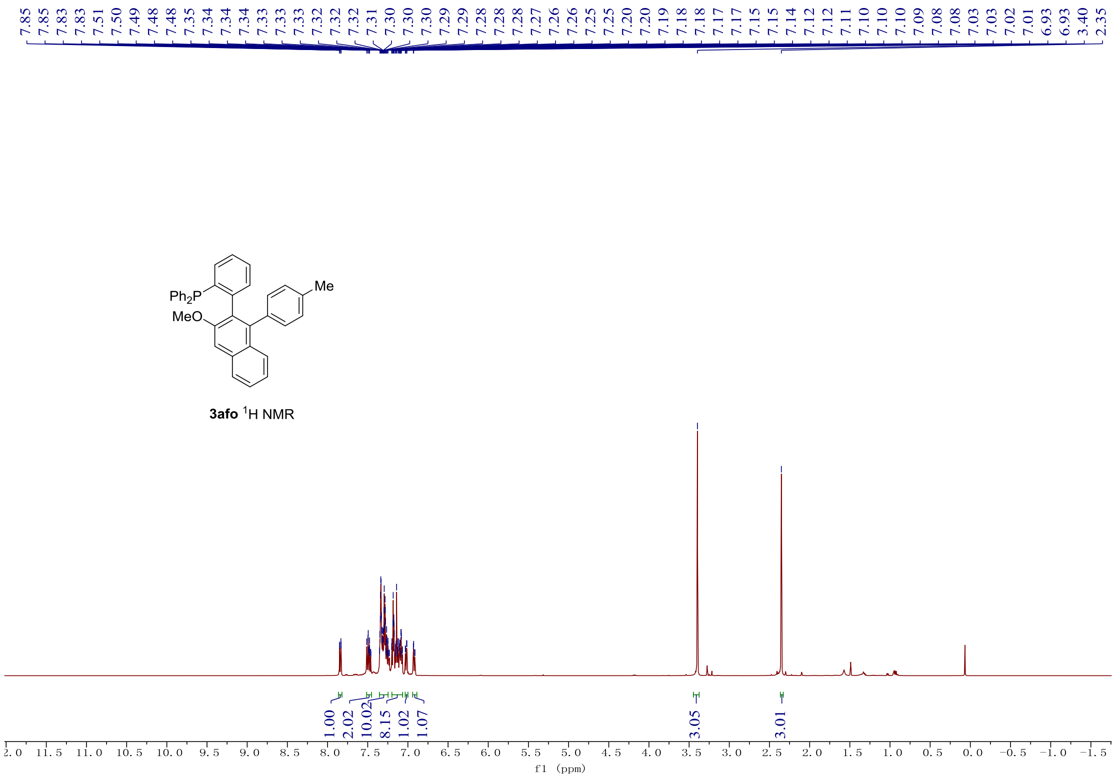

Supplementary Figure 95. ^{1}H NMR spectrum of **3afo**

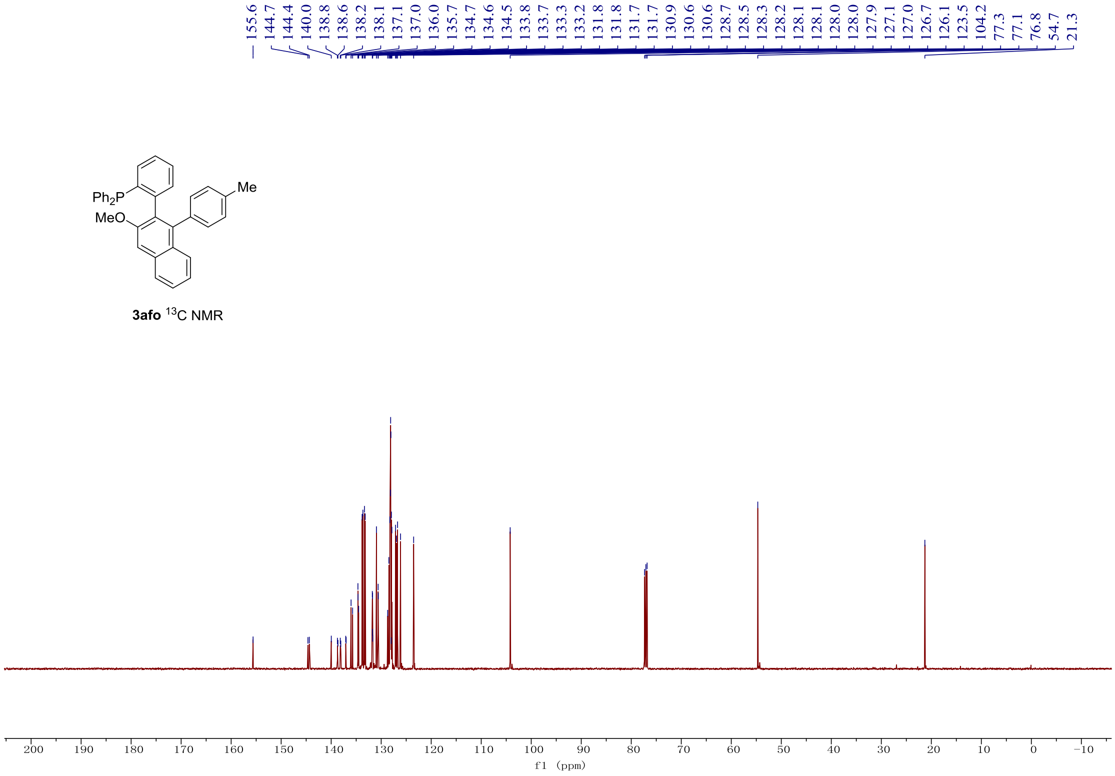

Supplementary Figure 96. ^{13}C NMR spectrum of **3afo**

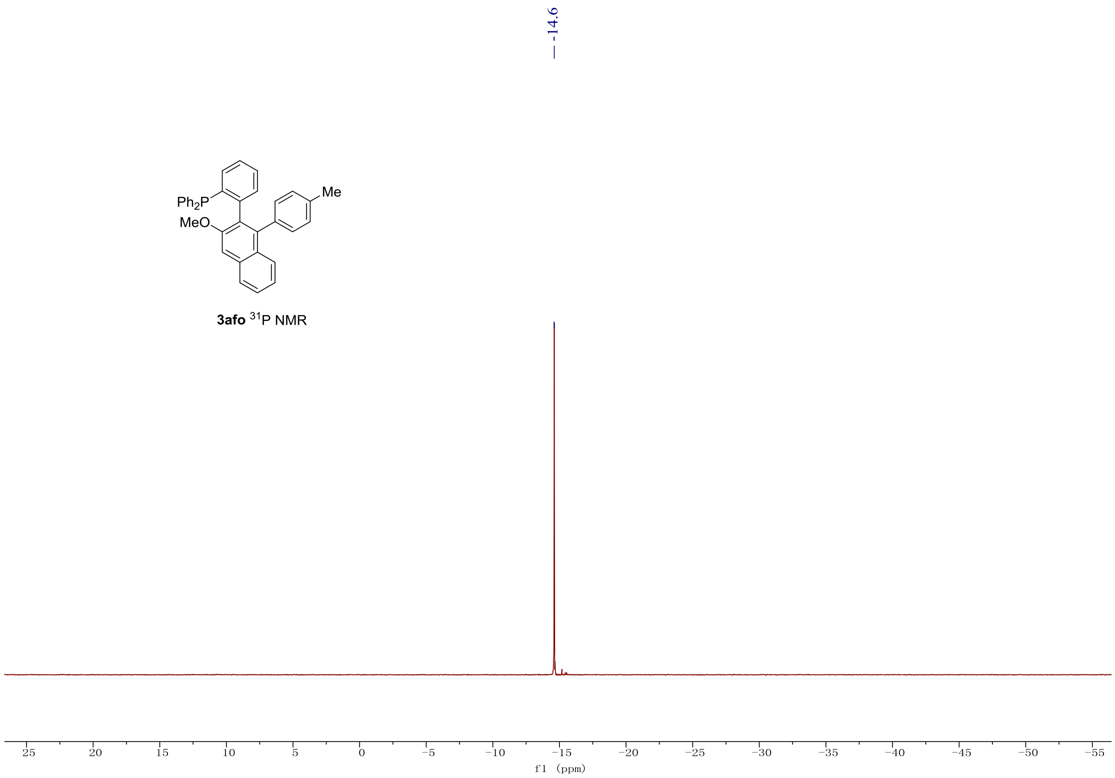

Supplementary Figure 97. ^{31}P NMR spectrum of **3afo**

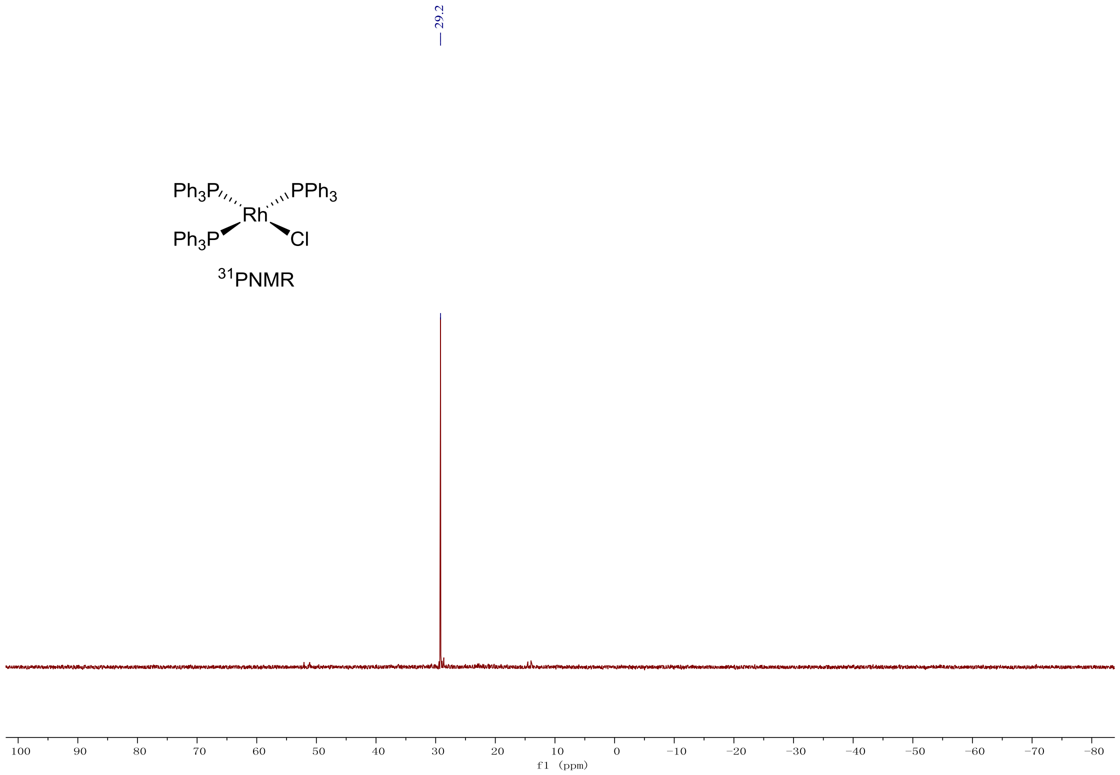

Supplementary Figure 98. ^{31}P NMR spectrum of Wilkinson’s catalyst

3 Supplementary References

1 vanderEnt, A.; Onderdelindent, A. L. *Inorg. Synth.***1990**, *86*, 90.

2 Katsuaki, B.; Mamoru, T.; Naoto, C. Qiu, X.; Wang, M.; Zhao, Y.; Shi, Z. *Angew.Chem. Int. Ed*. **2017**, *56*, 7233.

3 Gaussian 09, Revision E.01, Frisch, M. J.; Trucks, G. W.; Schlegel, H. B.; Scuseria, G. E.; Robb, M. A.; Cheeseman, J. R.; Scalmani, G.; Barone, V.; Mennucci, B.; Petersson, G. A.; Nakatsuji, H.; Caricato, M.; Li, X.; Hratchian, H. P.; Izmaylov, A. F.; Bloino, J.; Zheng, G.; Sonnenberg, J. L.; Hada, M.; Ehara, M.; Toyota, K.; Fukuda, R.; Hasegawa, J.; Ishida, M.; Nakajima, T.; Honda, Y.; Kitao, O.; Nakai, H.; Vreven, T.; Montgomery, J. A.; Peralta, Jr., J. E.; Ogliaro, F.; Bearpark, M.; Heyd, J. J.; Brothers, E.; Kudin, K. N.; Staroverov, V. N.; Keith, T.; Kobayashi, R.; Normand, J.; Raghavachari, K.; Rendell, A.; Burant, J. C.; Iyengar, S. S.; Tomasi, J.; Cossi, M.; Rega, N.; Millam, J. M.; Klene, M.; Knox, J. E.; Cross, J. B.; Bakken, V.; Adamo, C.; Jaramillo, J.; Gomperts, R.; Stratmann, R. E.; Yazyev, O.; Austin, A. J.; Cammi, R.; Pomelli, C.; Ochterski, J. W.; Martin, R. L.; Morokuma, K.; Zakrzewski, V. G.; Voth, G. A.; Salvador, P.; Dannenberg, J. J.; Dapprich, S.; Daniels, A. D.; Farkas, O.; Foresman, J. B.; Ortiz, J. V.; Cioslowski, J.; and Fox, D. J. Gaussian, Inc., Wallingford CT, **2013**.

4 a) Becke, A. D. *J. Chem. Phys.* **1993**, 98, 5648. b) Becke, A. D. *J. Chem. Phys.* **1993**, *98*, 1372. c) Stephens, P. J.; Devlin, F. J.; Chabalowski, C. F.; Frisch, M. J. *J. Phys. Chem.* **1994**, *98*, 11623. d) Lee, C.; Yang, W.; Parr, R. G. *Phys. Rev. A* **1988**, *37*, 785.

5 Hay, P. J.; Wadt, W. R. *J. Chem. Phys.*, **1985**, *82*, 299.

6 a) Ditchfield, R.; Hehre, W. J.; Pople, J. A. *J. Chem. Phys.* **1971**, *54*, 724. b) Hehre, W. J.; Ditchfield, R.; Pople, J. A. *J. Chem. Phys.* **1972**, *56*, 2257.

7 Zhao, Y.; Truhlar, D. G. *Theor. Chem. Acc.* **2008**, *120*, 215.

8 a) Dolg, M.; Wedig, U.; Stoll, H.; Preuss, H. *J. Chem. Phys.* **1987**, *86*, 866. b) Nicklass, A.; Dolg, M.; Stoll, H.; Preuss, H. *J. Chem. Phys.* **1995**, *102*, 8942.

9 a) Clark, T.; Chandrasekhar, J.; Spitznagel, G. W.; Schleyer, P. Von R. *J. Comput. Chem.* **1983**, *4*, 294. b) Krishnan, R.; Binkley, J. S.; Seeger, R.; Pople, J. A. *J. Chem. Phys.* **1980**, *72*, 650.

10 Marenich, A. V.; Cramer, C. J.; Truhlar, D. G. *J. Phys. Chem. B*. **2009**, *113*, 6378.

11 Legault, C. Y. CYL View, version 1.0 b; Universite de Sherbrooke, Sherbrooke, Quebec, Canada, **2009**; http://www.cylview.org.
