# Supplementary material for: Rhodium-catalyzed selective direct arylation of phosphines with aryl bromides
Source: Nat Commun. 2022 May 25;13:2934. doi: 10.1038/s41467-022-30697-7 (PMC9132997; doi:10.1038/s41467-022-30697-7)
Supplement: Supplementary file 3 — Description of Additional Supplementary Files [file 41467_2022_30697_MOESM3_ESM.docx]

File Name: Supplementary Data 1

Description: Crystallographic Data

File Name: Supplementary Data 2

Description: Table S1. The calculated energies of stationary points

File Name: Supplementary Data 3

Description: Cartesian coordinates of the optimized structures
