# Supplementary material for: Rhodium-catalyzed selective direct arylation of phosphines with aryl bromides
Source: Nat Commun. 2022 May 25;13:2934. doi: 10.1038/s41467-022-30697-7 (PMC9132997; doi:10.1038/s41467-022-30697-7)
Supplement: Supplementary file 4 — Supplementary Data 1 [file 41467_2022_30697_MOESM4_ESM.docx]

**Supplementary Data 1**

**Crystallographic Data**

P(1)-C(17) 1.839(5)

P(1)-C(12) 1.828(5)

P(1)-C(6) 1.840(5)

P(2)-C(62) 1.847(5)

P(2)-C(51) 1.828(6)

P(2)-C(57) 1.839(6)

O(2)-C(19) 1.367(6)

O(2)-C(24) 1.446(6)

O(3)-C(43) 1.376(6)

O(3)-C(63) 1.434(6)

O(1)-C(26) 1.368(6)

O(1)-C(31) 1.420(6)

O(4)-C(33) 1.360(6)

O(4)-C(32) 1.441(6)

C(39)-C(44) 1.383(7)

C(39)-C(38) 1.485(7)

C(39)-C(40) 1.405(7)

C(23)-C(18) 1.399(7)

C(23)-C(25) 1.490(7)

C(23)-C(22) 1.407(6)

C(43)-C(44) 1.401(6)

C(43)-C(42) 1.386(7)

C(61)-C(44) 1.505(6)

C(61)-C(62) 1.390(7)

C(61)-C(00O) 1.405(7)

C(19)-C(18) 1.397(6)

C(19)-C(20) 1.391(6)

C(17)-C(00D) 1.407(7)

C(17)-C(13) 1.403(6)

C(00D)-C(18) 1.499(6)

C(00D)-C(16) 1.379(7)

C(25)-C(26) 1.396(7)

C(25)-C(30) 1.379(7)

C(62)-C(58) 1.398(7)

C(22)-H(22) 0.9500

C(22)-C(21) 1.374(7)

C(16)-H(16) 0.9500

C(16)-C(15) 1.384(7)

C(38)-C(33) 1.391(7)

C(38)-C(37) 1.391(7)

C(42)-H(42) 0.9500

C(42)-C(41) 1.361(7)

C(40)-H(40) 0.9500

C(40)-C(41) 1.384(7)

C(20)-H(20) 0.9500

C(20)-C(21) 1.385(7)

C(00O)-H(00O) 0.9500

C(00O)-C(60) 1.372(7)

C(21)-H(21) 0.9500

C(26)-C(27) 1.385(7)

C(51)-C(46) 1.380(7)

C(51)-C(50) 1.385(8)

C(12)-C(11) 1.404(7)

C(12)-C(7) 1.383(7)

C(6)-C(5) 1.386(7)

C(6)-C(1) 1.377(7)

C(33)-C(34) 1.387(7)

C(5)-H(5) 0.9500

C(5)-C(4) 1.417(8)

C(13)-H(13) 0.9500

C(13)-C(14) 1.378(8)

C(41)-H(41) 0.9500

C(30)-H(30) 0.9500

C(30)-C(29) 1.381(8)

C(11)-H(11) 0.9500

C(11)-C(10) 1.382(8)

C(57)-C(52) 1.384(8)

C(57)-C(56) 1.398(8)

C(7)-H(7) 0.9500

C(7)-C(8) 1.379(8)

C(60)-H(60) 0.9500

C(60)-C(59) 1.371(8)

C(1)-H(1) 0.9500

C(1)-C(2) 1.395(8)

C(58)-H(58) 0.9500

C(58)-C(59) 1.382(8)

C(15)-H(15) 0.9500

C(15)-C(14) 1.374(8)

C(9)-H(9) 0.9500

C(9)-C(10) 1.363(8)

C(9)-C(8) 1.370(8)

C(37)-H(37) 0.9500

C(37)-C(36) 1.370(8)

C(27)-H(27) 0.9500

C(27)-C(28) 1.393(9)

C(46)-H(46) 0.9500

C(46)-C(47) 1.394(9)

C(34)-H(34) 0.9500

C(34)-C(35) 1.383(8)

C(50)-H(50) 0.9500

C(50)-C(49) 1.398(8)

C(10)-H(10) 0.9500

C(14)-H(14) 0.9500

C(52)-H(52) 0.9500

C(52)-C(53) 1.374(9)

C(48)-H(48) 0.9500

C(48)-C(49) 1.369(8)

C(48)-C(47) 1.364(9)

C(24)-H(24A) 0.9800

C(24)-H(24B) 0.9800

C(24)-H(24C) 0.9800

C(56)-H(56) 0.9500

C(56)-C(55) 1.393(9)

C(49)-H(49) 0.9500

C(8)-H(8) 0.9500

C(31)-H(31A) 0.9800

C(31)-H(31B) 0.9800

C(31)-H(31C) 0.9800

C(4)-H(4) 0.9500

C(4)-C(3) 1.365(9)

C(63)-H(63A) 0.9800

C(63)-H(63B) 0.9800

C(63)-H(63C) 0.9800

C(59)-H(59) 0.9500

C(36)-H(36) 0.9500

C(36)-C(35) 1.395(9)

C(29)-H(29) 0.9500

C(29)-C(28) 1.374(9)

C(35)-H(35) 0.9500

C(28)-H(28) 0.9500

C(47)-H(47) 0.9500

C(53)-H(53) 0.9500

C(53)-C(54) 1.389(11)

C(54)-H(54) 0.9500

C(54)-C(55) 1.363(11)

C(55)-H(55) 0.9500

C(3)-H(3) 0.9500

C(3)-C(2) 1.374(10)

C(32)-H(32A) 0.9800

C(32)-H(32B) 0.9800

C(32)-H(32C) 0.9800

C(2)-H(2) 0.9500

C(17)-P(1)-C(6) 101.4(2)

C(12)-P(1)-C(17) 102.3(2)

C(12)-P(1)-C(6) 101.9(2)

C(51)-P(2)-C(62) 103.4(2)

C(51)-P(2)-C(57) 101.4(3)

C(57)-P(2)-C(62) 101.5(2)

C(19)-O(2)-C(24) 117.7(4)

C(43)-O(3)-C(63) 117.2(4)

C(26)-O(1)-C(31) 118.6(4)

C(33)-O(4)-C(32) 118.5(4)

C(44)-C(39)-C(38) 120.2(4)

C(44)-C(39)-C(40) 119.7(4)

C(40)-C(39)-C(38) 120.0(5)

C(18)-C(23)-C(25) 120.0(4)

C(18)-C(23)-C(22) 119.6(4)

C(22)-C(23)-C(25) 120.4(4)

O(3)-C(43)-C(44) 115.4(4)

O(3)-C(43)-C(42) 124.4(4)

C(42)-C(43)-C(44) 120.1(5)

C(62)-C(61)-C(44) 122.6(4)

C(62)-C(61)-C(00O) 120.2(4)

C(00O)-C(61)-C(44) 117.1(4)

O(2)-C(19)-C(18) 115.5(4)

O(2)-C(19)-C(20) 124.0(4)

C(20)-C(19)-C(18) 120.5(5)

C(00D)-C(17)-P(1) 118.6(3)

C(13)-C(17)-P(1) 123.9(4)

C(13)-C(17)-C(00D) 117.5(5)

C(17)-C(00D)-C(18) 121.9(4)

C(16)-C(00D)-C(17) 120.0(4)

C(16)-C(00D)-C(18) 118.1(4)

C(23)-C(18)-C(00D) 120.3(4)

C(19)-C(18)-C(23) 119.6(4)

C(19)-C(18)-C(00D) 120.1(4)

C(26)-C(25)-C(23) 120.5(4)

C(30)-C(25)-C(23) 120.0(5)

C(30)-C(25)-C(26) 119.4(5)

C(39)-C(44)-C(43) 119.7(4)

C(39)-C(44)-C(61) 119.9(4)

C(43)-C(44)-C(61) 120.4(4)

C(61)-C(62)-P(2) 118.6(3)

C(61)-C(62)-C(58) 118.0(5)

C(58)-C(62)-P(2) 123.3(4)

C(23)-C(22)-H(22) 120.2

C(21)-C(22)-C(23) 119.6(5)

C(21)-C(22)-H(22) 120.2

C(00D)-C(16)-H(16) 119.2

C(00D)-C(16)-C(15) 121.6(5)

C(15)-C(16)-H(16) 119.2

C(33)-C(38)-C(39) 121.9(4)

C(37)-C(38)-C(39) 119.9(5)

C(37)-C(38)-C(33) 118.2(5)

C(43)-C(42)-H(42) 120.1

C(41)-C(42)-C(43) 119.7(5)

C(41)-C(42)-H(42) 120.1

C(39)-C(40)-H(40) 120.4

C(41)-C(40)-C(39) 119.2(5)

C(41)-C(40)-H(40) 120.4

C(19)-C(20)-H(20) 120.4

C(21)-C(20)-C(19) 119.2(5)

C(21)-C(20)-H(20) 120.4

C(61)-C(00O)-H(00O) 119.9

C(60)-C(00O)-C(61) 120.3(5)

C(60)-C(00O)-H(00O) 119.9

C(22)-C(21)-C(20) 121.6(4)

C(22)-C(21)-H(21) 119.2

C(20)-C(21)-H(21) 119.2

O(1)-C(26)-C(25) 115.2(4)

O(1)-C(26)-C(27) 124.2(5)

C(27)-C(26)-C(25) 120.7(5)

C(46)-C(51)-P(2) 117.9(5)

C(46)-C(51)-C(50) 117.7(5)

C(50)-C(51)-P(2) 124.5(4)

C(11)-C(12)-P(1) 117.8(4)

C(7)-C(12)-P(1) 124.5(4)

C(7)-C(12)-C(11) 117.6(5)

C(5)-C(6)-P(1) 116.0(4)

C(1)-C(6)-P(1) 125.8(4)

C(1)-C(6)-C(5) 118.2(5)

O(4)-C(33)-C(38) 114.5(4)

O(4)-C(33)-C(34) 124.7(5)

C(34)-C(33)-C(38) 120.8(5)

C(6)-C(5)-H(5) 119.8

C(6)-C(5)-C(4) 120.5(6)

C(4)-C(5)-H(5) 119.8

C(17)-C(13)-H(13) 119.4

C(14)-C(13)-C(17) 121.2(5)

C(14)-C(13)-H(13) 119.4

C(42)-C(41)-C(40) 121.5(5)

C(42)-C(41)-H(41) 119.2

C(40)-C(41)-H(41) 119.2

C(25)-C(30)-H(30) 120.0

C(25)-C(30)-C(29) 120.1(6)

C(29)-C(30)-H(30) 120.0

C(12)-C(11)-H(11) 119.3

C(10)-C(11)-C(12) 121.3(5)

C(10)-C(11)-H(11) 119.3

C(52)-C(57)-P(2) 125.2(5)

C(52)-C(57)-C(56) 118.0(6)

C(56)-C(57)-P(2) 116.8(5)

C(12)-C(7)-H(7) 119.8

C(8)-C(7)-C(12) 120.5(5)

C(8)-C(7)-H(7) 119.8

C(00O)-C(60)-H(60) 120.0

C(59)-C(60)-C(00O) 119.9(5)

C(59)-C(60)-H(60) 120.0

C(6)-C(1)-H(1) 119.4

C(6)-C(1)-C(2) 121.3(6)

C(2)-C(1)-H(1) 119.4

C(62)-C(58)-H(58) 119.5

C(59)-C(58)-C(62) 121.1(5)

C(59)-C(58)-H(58) 119.5

C(16)-C(15)-H(15) 120.6

C(14)-C(15)-C(16) 118.7(5)

C(14)-C(15)-H(15) 120.6

C(10)-C(9)-H(9) 119.8

C(10)-C(9)-C(8) 120.3(6)

C(8)-C(9)-H(9) 119.8

C(38)-C(37)-H(37) 119.0

C(36)-C(37)-C(38) 122.1(6)

C(36)-C(37)-H(37) 119.0

C(26)-C(27)-H(27) 120.6

C(26)-C(27)-C(28) 118.9(6)

C(28)-C(27)-H(27) 120.6

C(51)-C(46)-H(46) 119.7

C(51)-C(46)-C(47) 120.6(6)

C(47)-C(46)-H(46) 119.7

C(33)-C(34)-H(34) 120.2

C(35)-C(34)-C(33) 119.6(5)

C(35)-C(34)-H(34) 120.2

C(51)-C(50)-H(50) 119.4

C(51)-C(50)-C(49) 121.1(5)

C(49)-C(50)-H(50) 119.4

C(11)-C(10)-H(10) 120.3

C(9)-C(10)-C(11) 119.5(5)

C(9)-C(10)-H(10) 120.3

C(13)-C(14)-H(14) 119.6

C(15)-C(14)-C(13) 120.8(5)

C(15)-C(14)-H(14) 119.6

C(57)-C(52)-H(52) 119.1

C(53)-C(52)-C(57) 121.8(7)

C(53)-C(52)-H(52) 119.1

C(49)-C(48)-H(48) 120.7

C(47)-C(48)-H(48) 120.7

C(47)-C(48)-C(49) 118.6(6)

O(2)-C(24)-H(24A) 109.5

O(2)-C(24)-H(24B) 109.5

O(2)-C(24)-H(24C) 109.5

H(24A)-C(24)-H(24B) 109.5

H(24A)-C(24)-H(24C) 109.5

H(24B)-C(24)-H(24C) 109.5

C(57)-C(56)-H(56) 119.7

C(55)-C(56)-C(57) 120.5(7)

C(55)-C(56)-H(56) 119.7

C(50)-C(49)-H(49) 119.8

C(48)-C(49)-C(50) 120.5(7)

C(48)-C(49)-H(49) 119.8

C(7)-C(8)-H(8) 119.6

C(9)-C(8)-C(7) 120.8(6)

C(9)-C(8)-H(8) 119.6

O(1)-C(31)-H(31A) 109.5

O(1)-C(31)-H(31B) 109.5

O(1)-C(31)-H(31C) 109.5

H(31A)-C(31)-H(31B) 109.5

H(31A)-C(31)-H(31C) 109.5

H(31B)-C(31)-H(31C) 109.5

C(5)-C(4)-H(4) 120.1

C(3)-C(4)-C(5) 119.8(6)

C(3)-C(4)-H(4) 120.1

O(3)-C(63)-H(63A) 109.5

O(3)-C(63)-H(63B) 109.5

O(3)-C(63)-H(63C) 109.5

H(63A)-C(63)-H(63B) 109.5

H(63A)-C(63)-H(63C) 109.5

H(63B)-C(63)-H(63C) 109.5

C(60)-C(59)-C(58) 120.4(5)

C(60)-C(59)-H(59) 119.8

C(58)-C(59)-H(59) 119.8

C(37)-C(36)-H(36) 120.6

C(37)-C(36)-C(35) 118.8(6)

C(35)-C(36)-H(36) 120.6

C(30)-C(29)-H(29) 119.7

C(28)-C(29)-C(30) 120.5(6)

C(28)-C(29)-H(29) 119.7

C(34)-C(35)-C(36) 120.6(5)

C(34)-C(35)-H(35) 119.7

C(36)-C(35)-H(35) 119.7

C(27)-C(28)-H(28) 119.8

C(29)-C(28)-C(27) 120.4(6)

C(29)-C(28)-H(28) 119.8

C(46)-C(47)-H(47) 119.2

C(48)-C(47)-C(46) 121.5(6)

C(48)-C(47)-H(47) 119.2

C(52)-C(53)-H(53) 120.5

C(52)-C(53)-C(54) 118.9(8)

C(54)-C(53)-H(53) 120.5

C(53)-C(54)-H(54) 119.5

C(55)-C(54)-C(53) 121.0(7)

C(55)-C(54)-H(54) 119.5

C(56)-C(55)-H(55) 120.2

C(54)-C(55)-C(56) 119.6(8)

C(54)-C(55)-H(55) 120.2

C(4)-C(3)-H(3) 120.0

C(4)-C(3)-C(2) 120.1(6)

C(2)-C(3)-H(3) 120.0

O(4)-C(32)-H(32A) 109.5

O(4)-C(32)-H(32B) 109.5

O(4)-C(32)-H(32C) 109.5

H(32A)-C(32)-H(32B) 109.5

H(32A)-C(32)-H(32C) 109.5

H(32B)-C(32)-H(32C) 109.5

C(1)-C(2)-H(2) 120.0

C(3)-C(2)-C(1) 120.1(7)

C(3)-C(2)-H(2) 120.0

C(1)-C(2) 1.389(3)

C(1)-C(6) 1.391(3)

C(1)-H(1) 0.9500

C(2)-C(3) 1.378(3)

C(2)-H(2) 0.9500

C(3)-C(4) 1.381(3)

C(3)-H(3) 0.9500

C(4)-C(5) 1.382(3)

C(4)-H(4) 0.9500

C(5)-C(6) 1.398(3)

C(5)-H(5) 0.9500

C(6)-P(1) 1.8373(19)

C(7)-C(8) 1.375(3)

C(7)-C(12) 1.397(2)

C(7)-H(7) 0.9500

C(8)-C(9) 1.387(3)

C(8)-H(8) 0.9500

C(9)-C(10) 1.377(3)

C(9)-H(9) 0.9500

C(10)-C(11) 1.385(3)

C(10)-H(10) 0.9500

C(11)-C(12) 1.393(3)

C(11)-H(11) 0.9500

C(12)-P(1) 1.8360(19)

C(13)-C(14) 1.397(2)

C(13)-C(18) 1.405(2)

C(13)-P(1) 1.8518(17)

C(14)-C(15) 1.385(3)

C(14)-H(14) 0.9500

C(15)-C(16) 1.380(3)

C(15)-H(15) 0.9500

C(16)-C(17) 1.386(3)

C(16)-H(16) 0.9500

C(17)-C(18) 1.397(2)

C(17)-H(17) 0.9500

C(18)-C(19) 1.499(2)

C(19)-C(24) 1.410(3)

C(19)-C(20) 1.410(2)

C(20)-C(21) 1.399(2)

C(20)-C(36) 1.487(3)

C(21)-C(22) 1.381(3)

C(21)-H(21) 0.9500

C(22)-O(2) 1.377(2)

C(22)-C(23) 1.388(2)

C(23)-C(24) 1.393(2)

C(23)-H(23) 0.9500

C(24)-C(30) 1.497(2)

C(25)-C(26) 1.376(3)

C(25)-C(30) 1.393(2)

C(25)-H(25) 0.9500

C(26)-C(27) 1.382(3)

C(26)-H(26) 0.9500

C(27)-O(1) 1.372(2)

C(27)-C(28) 1.388(3)

C(28)-C(29) 1.392(2)

C(28)-H(28) 0.9500

C(29)-C(30) 1.390(3)

C(29)-H(29) 0.9500

C(31)-C(32) 1.377(3)

C(31)-C(36) 1.398(2)

C(31)-H(31) 0.9500

C(32)-C(33) 1.390(3)

C(32)-H(32) 0.9500

C(33)-O(3) 1.371(2)

C(33)-C(34) 1.385(3)

C(34)-C(35) 1.389(3)

C(34)-H(34) 0.9500

C(35)-C(36) 1.393(2)

C(35)-H(35) 0.9500

C(37)-O(3) 1.425(2)

C(37)-H(37A) 0.9800

C(37)-H(37B) 0.9800

C(37)-H(37C) 0.9800

C(38)-O(2) 1.431(2)

C(38)-H(38A) 0.9800

C(38)-H(38B) 0.9800

C(38)-H(38C) 0.9800

C(39)-O(1) 1.414(3)

C(39)-H(39A) 0.9800

C(39)-H(39B) 0.9800

C(39)-H(39C) 0.9800

C(2)-C(1)-C(6) 120.94(19)

C(2)-C(1)-H(1) 119.5

C(6)-C(1)-H(1) 119.5

C(3)-C(2)-C(1) 120.37(19)

C(3)-C(2)-H(2) 119.8

C(1)-C(2)-H(2) 119.8

C(2)-C(3)-C(4) 119.54(19)

C(2)-C(3)-H(3) 120.2

C(4)-C(3)-H(3) 120.2

C(3)-C(4)-C(5) 120.2(2)

C(3)-C(4)-H(4) 119.9

C(5)-C(4)-H(4) 119.9

C(4)-C(5)-C(6) 121.14(18)

C(4)-C(5)-H(5) 119.4

C(6)-C(5)-H(5) 119.4

C(1)-C(6)-C(5) 117.76(18)

C(1)-C(6)-P(1) 123.55(15)

C(5)-C(6)-P(1) 118.18(14)

C(8)-C(7)-C(12) 120.83(17)

C(8)-C(7)-H(7) 119.6

C(12)-C(7)-H(7) 119.6

C(7)-C(8)-C(9) 120.19(18)

C(7)-C(8)-H(8) 119.9

C(9)-C(8)-H(8) 119.9

C(10)-C(9)-C(8) 119.86(19)

C(10)-C(9)-H(9) 120.1

C(8)-C(9)-H(9) 120.1

C(9)-C(10)-C(11) 120.04(18)

C(9)-C(10)-H(10) 120.0

C(11)-C(10)-H(10) 120.0

C(10)-C(11)-C(12) 120.85(17)

C(10)-C(11)-H(11) 119.6

C(12)-C(11)-H(11) 119.6

C(11)-C(12)-C(7) 118.23(17)

C(11)-C(12)-P(1) 117.09(13)

C(7)-C(12)-P(1) 124.59(14)

C(14)-C(13)-C(18) 118.92(16)

C(14)-C(13)-P(1) 121.95(13)

C(18)-C(13)-P(1) 119.13(13)

C(15)-C(14)-C(13) 121.29(17)

C(15)-C(14)-H(14) 119.4

C(13)-C(14)-H(14) 119.4

C(16)-C(15)-C(14) 119.79(17)

C(16)-C(15)-H(15) 120.1

C(14)-C(15)-H(15) 120.1

C(15)-C(16)-C(17) 119.77(17)

C(15)-C(16)-H(16) 120.1

C(17)-C(16)-H(16) 120.1

C(16)-C(17)-C(18) 121.24(17)

C(16)-C(17)-H(17) 119.4

C(18)-C(17)-H(17) 119.4

C(17)-C(18)-C(13) 118.96(16)

C(17)-C(18)-C(19) 118.21(15)

C(13)-C(18)-C(19) 122.82(15)

C(24)-C(19)-C(20) 119.04(16)

C(24)-C(19)-C(18) 120.29(15)

C(20)-C(19)-C(18) 120.61(16)

C(21)-C(20)-C(19) 119.70(17)

C(21)-C(20)-C(36) 117.47(15)

C(19)-C(20)-C(36) 122.82(16)

C(22)-C(21)-C(20) 120.70(16)

C(22)-C(21)-H(21) 119.7

C(20)-C(21)-H(21) 119.7

O(2)-C(22)-C(21) 124.21(16)

O(2)-C(22)-C(23) 115.82(16)

C(21)-C(22)-C(23) 119.97(16)

C(22)-C(23)-C(24) 120.63(17)

C(22)-C(23)-H(23) 119.7

C(24)-C(23)-H(23) 119.7

C(23)-C(24)-C(19) 119.89(16)

C(23)-C(24)-C(30) 117.29(16)

C(19)-C(24)-C(30) 122.72(15)

C(26)-C(25)-C(30) 121.38(17)

C(26)-C(25)-H(25) 119.3

C(30)-C(25)-H(25) 119.3

C(25)-C(26)-C(27) 120.37(17)

C(25)-C(26)-H(26) 119.8

C(27)-C(26)-H(26) 119.8

O(1)-C(27)-C(26) 115.71(16)

O(1)-C(27)-C(28) 124.51(17)

C(26)-C(27)-C(28) 119.76(17)

C(27)-C(28)-C(29) 119.19(17)

C(27)-C(28)-H(28) 120.4

C(29)-C(28)-H(28) 120.4

C(30)-C(29)-C(28) 121.73(16)

C(30)-C(29)-H(29) 119.1

C(28)-C(29)-H(29) 119.1

C(29)-C(30)-C(25) 117.54(17)

C(29)-C(30)-C(24) 121.58(15)

C(25)-C(30)-C(24) 120.70(16)

C(32)-C(31)-C(36) 121.19(16)

C(32)-C(31)-H(31) 119.4

C(36)-C(31)-H(31) 119.4

C(31)-C(32)-C(33) 120.47(17)

C(31)-C(32)-H(32) 119.8

C(33)-C(32)-H(32) 119.8

O(3)-C(33)-C(34) 124.56(16)

O(3)-C(33)-C(32) 115.73(16)

C(34)-C(33)-C(32) 119.71(18)

C(33)-C(34)-C(35) 119.05(17)

C(33)-C(34)-H(34) 120.5

C(35)-C(34)-H(34) 120.5

C(34)-C(35)-C(36) 122.31(17)

C(34)-C(35)-H(35) 118.8

C(36)-C(35)-H(35) 118.8

C(35)-C(36)-C(31) 117.19(17)

C(35)-C(36)-C(20) 119.73(16)

C(31)-C(36)-C(20) 123.08(15)

O(3)-C(37)-H(37A) 109.5

O(3)-C(37)-H(37B) 109.5

H(37A)-C(37)-H(37B) 109.5

O(3)-C(37)-H(37C) 109.5

H(37A)-C(37)-H(37C) 109.5

H(37B)-C(37)-H(37C) 109.5

O(2)-C(38)-H(38A) 109.5

O(2)-C(38)-H(38B) 109.5

H(38A)-C(38)-H(38B) 109.5

O(2)-C(38)-H(38C) 109.5

H(38A)-C(38)-H(38C) 109.5

H(38B)-C(38)-H(38C) 109.5

O(1)-C(39)-H(39A) 109.5

O(1)-C(39)-H(39B) 109.5

H(39A)-C(39)-H(39B) 109.5

O(1)-C(39)-H(39C) 109.5

H(39A)-C(39)-H(39C) 109.5

H(39B)-C(39)-H(39C) 109.5

C(27)-O(1)-C(39) 117.37(15)

C(22)-O(2)-C(38) 116.15(14)

C(33)-O(3)-C(37) 117.27(15)

C(12)-P(1)-C(6) 103.42(8)

C(12)-P(1)-C(13) 100.35(8)

C(6)-P(1)-C(13) 101.73(8)

| C1 | | | C2 | | 1.394(3) | | |  | C28 | | Fe3 | | 2.030(2) | | |
| --- | --- | --- | --- | --- | --- | --- | --- | --- | --- | --- | --- | --- | --- | --- | --- |
| C1 | | | C6 | | 1.391(3) | | |  | C29 | | C30 | | 1.442(3) | | |
| C2 | | | C3 | | 1.377(4) | | |  | C29 | | C33 | | 1.426(3) | | |
| C3 | | | C4 | | 1.381(4) | | |  | C29 | | Fe2 | | 2.076(2) | | |
| C4 | | | C5 | | 1.379(3) | | |  | C30 | | C31 | | 1.439(3) | | |
| C5 | | | C6 | | 1.395(3) | | |  | C30 | | Fe2 | | 2.068(2) | | |
| C6 | | | P1 | | 1.829(2) | | |  | C31 | | C32 | | 1.429(3) | | |
| C7 | | | C8 | | 1.392(3) | | |  | C31 | | C47 | | 1.470(3) | | |
| C7 | | | C12 | | 1.389(3) | | |  | C31 | | Fe2 | | 2.078(2) | | |
| C8 | | | C9 | | 1.374(4) | | |  | C32 | | C33 | | 1.415(3) | | |
| C9 | | | C10 | | 1.376(4) | | |  | C32 | | Fe2 | | 2.060(2) | | |
| C10 | | | C11 | | 1.389(3) | | |  | C33 | | Fe2 | | 2.053(2) | | |
| C11 | | | C12 | | 1.392(3) | | |  | C34 | | C35 | | 1.406(3) | | |
| C12 | | | P1 | | 1.845(2) | | |  | C34 | | C38 | | 1.412(4) | | |
| C13 | | | C14 | | 1.382(3) | | |  | C34 | | Fe2 | | 2.063(2) | | |
| C13 | | | C18 | | 1.397(3) | | |  | C35 | | C36 | | 1.404(4) | | |
| C14 | | | C15 | | 1.375(3) | | |  | C35 | | Fe2 | | 2.064(2) | | |
| C15 | | | C16 | | 1.384(3) | | |  | C36 | | C37 | | 1.402(4) | | |
| C16 | | | C17 | | 1.397(3) | | |  | C36 | | Fe2 | | 2.057(2) | | |
| C17 | | | C18 | | 1.405(3) | | |  | C37 | | C38 | | 1.418(4) | | |
| C17 | | | C30 | | 1.493(3) | | |  | C37 | | Fe2 | | 2.046(2) | | |
| C18 | | | P1 | | 1.843(2) | | |  | C38 | | Fe2 | | 2.048(2) | | |
| C19 | | | C20 | | 1.398(4) | | |  | C39 | | C40 | | 1.414(3) | | |
| C19 | | | C23 | | 1.413(5) | | |  | C39 | | C43 | | 1.418(3) | | |
| C19 | | | Fe3 | | 2.026(3) | | |  | C39 | | Fe1 | | 2.036(2) | | |
| C20 | | | C21 | | 1.416(4) | | |  | C40 | | C41 | | 1.418(3) | | |
| C20 | | | Fe3 | | 2.035(3) | | |  | C40 | | Fe1 | | 2.028(2) | | |
| C21 | | | C22 | | 1.406(4) | | |  | C41 | | C42 | | 1.411(3) | | |
| C21 | | | Fe3 | | 2.042(3) | | |  | C41 | | Fe1 | | 2.045(2) | | |
| C22 | | | C23 | | 1.400(4) | | |  | C42 | | C43 | | 1.424(3) | |  |
| C22 | | | Fe3 | | 2.040(3) | | |  | C42 | | Fe1 | | 2.050(2) | |  |
| C23 | | | Fe3 | | 2.037(3) | | |  | C43 | | Fe1 | | 2.046(2) | |  |
| C24 | | | C25 | | 1.418(3) | | |  | C44 | | C45 | | 1.409(4) | |  |
| C24 | | | C28 | | 1.405(4) | | |  | C44 | | C48 | | 1.426(3) | |  |
| C24 | | | Fe3 | | 2.044(3) | | |  | C44 | | Fe1 | | 2.032(2) | |  |
| C25 | | | C26 | | 1.418(3) | | |  | C45 | | C46 | | 1.425(3) | |  |
| C25 | | | Fe3 | | 2.055(2) | | |  | C45 | | Fe1 | | 2.027(2) | |  |
| C26 | | | C27 | | 1.431(3) | | |  | C46 | | C47 | | 1.425(3) | |  |
| C26 | | | C29 | | 1.468(3) | | |  | C46 | | Fe1 | | 2.030(2) | |  |
| C26 | | | Fe3 | | 2.060(2) | | |  | C47 | | C48 | | 1.423(3) | |  |
| C27 | | | C28 | | 1.419(3) | | |  | C47 | | Fe1 | | 2.053(2) | |  |
| C27 | | | Fe3 | | 2.030(2) | | |  | C48 | | Fe1 | | 2.047(2) | |  |
| **Atom** | **Atom** | **Atom** | | **Angle/˚** | |  | **Atom** | | | **Atom** | | **Atom** | | **Angle/˚** | |
| C6 | C1 | C2 | | 120.1(2) | |  | C39 | | | Fe1 | | C48 | | 107.59(10) | |
| C3 | C2 | C1 | | 120.5(2) | |  | C40 | | | Fe1 | | C39 | | 40.73(9) | |
| C2 | C3 | C4 | | 119.7(2) | |  | C40 | | | Fe1 | | C41 | | 40.74(9) | |
| C5 | C4 | C3 | | 120.3(2) | |  | C40 | | | Fe1 | | C42 | | 68.27(9) | |
| C4 | C5 | C6 | | 120.8(2) | |  | C40 | | | Fe1 | | C43 | | 68.37(10) | |
| C1 | C6 | C5 | | 118.6(2) | |  | C40 | | | Fe1 | | C44 | | 156.60(11) | |
| C1 | C6 | P1 | | 124.25(17) | |  | C40 | | | Fe1 | | C46 | | 124.31(9) | |
| C5 | C6 | P1 | | 116.99(17) | |  | C40 | | | Fe1 | | C47 | | 107.39(9) | |
| C12 | C7 | C8 | | 120.5(2) | |  | C40 | | | Fe1 | | C48 | | 121.12(9) | |
| C9 | C8 | C7 | | 120.4(3) | |  | C41 | | | Fe1 | | C42 | | 40.33(9) | |
| C8 | C9 | C10 | | 119.8(2) | |  | C41 | | | Fe1 | | C43 | | 68.23(10) | |
| C9 | C10 | C11 | | 120.2(2) | |  | C41 | | | Fe1 | | C47 | | 121.15(9) | |
| C10 | C11 | C12 | | 120.8(2) | |  | C41 | | | Fe1 | | C48 | | 156.48(9) | |
| C7 | C12 | C11 | | 118.3(2) | |  | C42 | | | Fe1 | | C47 | | 156.27(10) | |
| C7 | C12 | P1 | | 125.67(18) | |  | C43 | | | Fe1 | | C42 | | 40.70(10) | |
| C11 | C12 | P1 | | 116.00(17) | |  | C43 | | | Fe1 | | C47 | | 161.47(10) | |
| C14 | C13 | C18 | | 121.7(2) | |  | C43 | | | Fe1 | | C48 | | 124.83(10) | |
| C15 | C14 | C13 | | 119.7(2) | |  | C44 | | | Fe1 | | C39 | | 121.15(11) | |
| C14 | C15 | C16 | | 119.4(2) | |  | C44 | | | Fe1 | | C41 | | 161.18(10) | |
| C15 | C16 | C17 | | 122.1(2) | |  | C44 | | | Fe1 | | C42 | | 124.66(10) | |
| C16 | C17 | C18 | | 118.16(19) | |  | C44 | | | Fe1 | | C43 | | 107.58(10) | |
| C16 | C17 | C30 | | 120.82(19) | |  | C44 | | | Fe1 | | C47 | | 68.71(9) | |
| C18 | C17 | C30 | | 121.02(18) | |  | C44 | | | Fe1 | | C48 | | 40.93(9) | |
| C13 | C18 | C17 | | 118.88(19) | |  | C45 | | | Fe1 | | C39 | | 156.26(11) | |
| C13 | C18 | P1 | | 120.47(16) | |  | C45 | | | Fe1 | | C40 | | 161.47(11) | |
| C17 | C18 | P1 | | 120.36(15) | |  | C45 | | | Fe1 | | C41 | | 124.43(11) | |
| C20 | C19 | C23 | | 108.1(3) | |  | C45 | | | Fe1 | | C42 | | 107.41(10) | |
| C20 | C19 | Fe3 | | 70.22(16) | |  | C45 | | | Fe1 | | C43 | | 120.93(10) | |
| C23 | C19 | Fe3 | | 70.08(17) | |  | C45 | | | Fe1 | | C44 | | 40.64(11) | |
| C19 | C20 | C21 | | 107.9(3) | |  | C45 | | | Fe1 | | C46 | | 41.13(10) | |
| C19 | C20 | Fe3 | | 69.52(17) | |  | C45 | | | Fe1 | | C47 | | 68.89(9) | |
| C21 | C20 | Fe3 | | 69.92(16) | |  | C45 | | | Fe1 | | C48 | | 68.66(10) | |
| C20 | C21 | Fe3 | | 69.42(15) | |  | C46 | | | Fe1 | | C39 | | 161.30(9) | |
| C22 | C21 | C20 | | 107.9(3) | |  | C46 | | | Fe1 | | C41 | | 107.30(10) | |
| C22 | C21 | Fe3 | | 69.81(16) | |  | C46 | | | Fe1 | | C42 | | 120.93(10) | |
| C21 | C22 | Fe3 | | 69.91(15) | |  | C46 | | | Fe1 | | C43 | | 156.44(10) | |
| C23 | C22 | C21 | | 108.1(3) | |  | C46 | | | Fe1 | | C44 | | 68.77(11) | |
| C23 | C22 | Fe3 | | 69.80(15) | |  | C46 | | | Fe1 | | C47 | | 40.84(9) | |
| C19 | C23 | Fe3 | | 69.22(16) | |  | C46 | | | Fe1 | | C48 | | 68.59(10) | |
| C22 | C23 | C19 | | 108.0(3) | |  | C48 | | | Fe1 | | C42 | | 161.79(10) | |
| C22 | C23 | Fe3 | | 70.04(16) | |  | C48 | | | Fe1 | | C47 | | 40.62(9) | |
| C25 | C24 | Fe3 | | 70.16(14) | |  | C29 | | | Fe2 | | C31 | | 68.20(8) | |
| C28 | C24 | C25 | | 108.1(2) | |  | C30 | | | Fe2 | | C29 | | 40.71(8) | |
| C28 | C24 | Fe3 | | 69.29(14) | |  | C30 | | | Fe2 | | C31 | | 40.63(8) | |
| C24 | C25 | Fe3 | | 69.35(13) | |  | C32 | | | Fe2 | | C29 | | 67.84(8) | |
| C26 | C25 | C24 | | 108.6(2) | |  | C32 | | | Fe2 | | C30 | | 68.10(8) | |
| C26 | C25 | Fe3 | | 70.07(12) | |  | C32 | | | Fe2 | | C31 | | 40.42(8) | |
| C25 | C26 | C27 | | 106.91(19) | |  | C32 | | | Fe2 | | C34 | | 149.32(10) | |
| C25 | C26 | C29 | | 128.2(2) | |  | C32 | | | Fe2 | | C35 | | 115.78(9) | |
| C25 | C26 | Fe3 | | 69.63(12) | |  | C33 | | | Fe2 | | C29 | | 40.39(8) | |
| C27 | C26 | C29 | | 124.40(19) | |  | C33 | | | Fe2 | | C30 | | 68.23(8) | |
| C27 | C26 | Fe3 | | 68.39(12) | |  | C33 | | | Fe2 | | C31 | | 68.02(8) | |
| C29 | C26 | Fe3 | | 133.37(15) | |  | C33 | | | Fe2 | | C32 | | 40.24(8) | |
| C26 | C27 | Fe3 | | 70.66(12) | |  | C33 | | | Fe2 | | C34 | | 170.42(9) | |
| C28 | C27 | C26 | | 108.2(2) | |  | C33 | | | Fe2 | | C35 | | 146.79(9) | |
| C28 | C27 | Fe3 | | 69.53(13) | |  | C33 | | | Fe2 | | C36 | | 114.38(10) | |
| C24 | C28 | C27 | | 108.2(2) | |  | C34 | | | Fe2 | | C29 | | 133.85(9) | |
| C24 | C28 | Fe3 | | 70.36(15) | |  | C34 | | | Fe2 | | C30 | | 112.31(9) | |
| C27 | C28 | Fe3 | | 69.55(13) | |  | C34 | | | Fe2 | | C31 | | 119.06(9) | |
| C26 | C29 | Fe2 | | 136.25(15) | |  | C34 | | | Fe2 | | C35 | | 39.82(10) | |
| C30 | C29 | C26 | | 124.50(18) | |  | C35 | | | Fe2 | | C29 | | 171.99(9) | |
| C30 | C29 | Fe2 | | 69.33(11) | |  | C35 | | | Fe2 | | C30 | | 132.66(9) | |
| C33 | C29 | C26 | | 126.99(19) | |  | C35 | | | Fe2 | | C31 | | 109.48(9) | |
| C33 | C29 | C30 | | 107.42(18) | |  | C36 | | | Fe2 | | C29 | | 147.58(10) | |
| C33 | C29 | Fe2 | | 68.93(12) | |  | C36 | | | Fe2 | | C30 | | 168.99(10) | |
| C17 | C30 | Fe2 | | 131.09(14) | |  | C36 | | | Fe2 | | C31 | | 129.19(10) | |
| C29 | C30 | C17 | | 126.07(18) | |  | C36 | | | Fe2 | | C32 | | 106.50(10) | |
| C29 | C30 | Fe2 | | 69.95(11) | |  | C36 | | | Fe2 | | C34 | | 67.06(10) | |
| C31 | C30 | C17 | | 125.67(18) | |  | C36 | | | Fe2 | | C35 | | 39.84(10) | |
| C31 | C30 | C29 | | 107.87(17) | |  | C37 | | | Fe2 | | C29 | | 116.96(10) | |
| C31 | C30 | Fe2 | | 70.05(11) | |  | C37 | | | Fe2 | | C30 | | 150.87(11) | |
| C30 | C31 | C47 | | 125.87(18) | |  | C37 | | | Fe2 | | C31 | | 166.53(11) | |
| C30 | C31 | Fe2 | | 69.32(11) | |  | C37 | | | Fe2 | | C32 | | 128.00(11) | |
| C32 | C31 | C30 | | 107.35(18) | |  | C37 | | | Fe2 | | C33 | | 107.20(10) | |
| C32 | C31 | C47 | | 125.88(19) | |  | C37 | | | Fe2 | | C34 | | 67.39(11) | |
| C32 | C31 | Fe2 | | 69.13(12) | |  | C37 | | | Fe2 | | C35 | | 67.13(10) | |
| C47 | C31 | Fe2 | | 135.19(15) | |  | C37 | | | Fe2 | | C36 | | 39.95(11) | |
| C31 | C32 | Fe2 | | 70.45(12) | |  | C37 | | | Fe2 | | C38 | | 40.54(12) | |
| C33 | C32 | C31 | | 108.63(18) | |  | C38 | | | Fe2 | | C29 | | 110.71(9) | |
| C33 | C32 | Fe2 | | 69.61(12) | |  | C38 | | | Fe2 | | C30 | | 119.57(10) | |
| C29 | C33 | Fe2 | | 70.68(12) | |  | C38 | | | Fe2 | | C31 | | 151.75(11) | |
| C32 | C33 | C29 | | 108.70(18) | |  | C38 | | | Fe2 | | C32 | | 167.45(11) | |
| C32 | C33 | Fe2 | | 70.15(12) | |  | C38 | | | Fe2 | | C33 | | 130.66(10) | |
| C35 | C34 | C38 | | 108.2(2) | |  | C38 | | | Fe2 | | C34 | | 40.18(11) | |
| C35 | C34 | Fe2 | | 70.14(13) | |  | C38 | | | Fe2 | | C35 | | 67.42(10) | |
| C38 | C34 | Fe2 | | 69.32(14) | |  | C38 | | | Fe2 | | C36 | | 67.62(11) | |
| C34 | C35 | Fe2 | | 70.04(14) | |  | C19 | | | Fe3 | | C20 | | 40.26(12) | |
| C36 | C35 | C34 | | 108.2(2) | |  | C19 | | | Fe3 | | C21 | | 67.99(12) | |
| C36 | C35 | Fe2 | | 69.78(14) | |  | C19 | | | Fe3 | | C22 | | 68.07(12) | |
| C35 | C36 | Fe2 | | 70.37(14) | |  | C19 | | | Fe3 | | C23 | | 40.70(13) | |
| C37 | C36 | C35 | | 108.2(2) | |  | C19 | | | Fe3 | | C24 | | 127.16(12) | |
| C37 | C36 | Fe2 | | 69.63(14) | |  | C19 | | | Fe3 | | C25 | | 108.48(11) | |
| C36 | C37 | C38 | | 108.2(2) | |  | C19 | | | Fe3 | | C26 | | 119.35(11) | |
| C36 | C37 | Fe2 | | 70.42(14) | |  | C19 | | | Fe3 | | C27 | | 153.68(12) | |
| C38 | C37 | Fe2 | | 69.78(14) | |  | C19 | | | Fe3 | | C28 | | 164.23(12) | |
| C34 | C38 | C37 | | 107.3(2) | |  | C20 | | | Fe3 | | C21 | | 40.66(12) | |
| C34 | C38 | Fe2 | | 70.49(14) | |  | C20 | | | Fe3 | | C22 | | 68.07(12) | |
| C37 | C38 | Fe2 | | 69.69(15) | |  | C20 | | | Fe3 | | C23 | | 67.96(14) | |
| C40 | C39 | C43 | | 107.8(2) | |  | C20 | | | Fe3 | | C24 | | 164.98(12) | |
| C40 | C39 | Fe1 | | 69.34(13) | |  | C20 | | | Fe3 | | C25 | | 127.91(11) | |
| C43 | C39 | Fe1 | | 70.04(14) | |  | C20 | | | Fe3 | | C26 | | 108.79(10) | |
| C39 | C40 | C41 | | 108.3(2) | |  | C21 | | | Fe3 | | C24 | | 152.56(12) | |
| C39 | C40 | Fe1 | | 69.93(13) | |  | C21 | | | Fe3 | | C25 | | 165.83(12) | |
| C41 | C40 | Fe1 | | 70.29(13) | |  | C21 | | | Fe3 | | C26 | | 128.27(12) | |
| C40 | C41 | Fe1 | | 68.98(12) | |  | C22 | | | Fe3 | | C21 | | 40.29(13) | |
| C42 | C41 | C40 | | 107.9(2) | |  | C22 | | | Fe3 | | C24 | | 118.50(12) | |
| C42 | C41 | Fe1 | | 70.00(13) | |  | C22 | | | Fe3 | | C25 | | 152.65(12) | |
| C41 | C42 | C43 | | 108.0(2) | |  | C22 | | | Fe3 | | C26 | | 165.55(11) | |
| C41 | C42 | Fe1 | | 69.67(13) | |  | C23 | | | Fe3 | | C21 | | 67.70(13) | |
| C43 | C42 | Fe1 | | 69.50(13) | |  | C23 | | | Fe3 | | C22 | | 40.16(12) | |
| C39 | C43 | C42 | | 107.9(2) | |  | C23 | | | Fe3 | | C24 | | 107.59(12) | |
| C39 | C43 | Fe1 | | 69.29(13) | |  | C23 | | | Fe3 | | C25 | | 119.28(11) | |
| C42 | C43 | Fe1 | | 69.80(13) | |  | C23 | | | Fe3 | | C26 | | 153.21(11) | |
| C45 | C44 | C48 | | 108.3(2) | |  | C24 | | | Fe3 | | C25 | | 40.49(9) | |
| C45 | C44 | Fe1 | | 69.52(14) | |  | C24 | | | Fe3 | | C26 | | 68.28(9) | |
| C48 | C44 | Fe1 | | 70.10(13) | |  | C25 | | | Fe3 | | C26 | | 40.30(8) | |
| C44 | C45 | C46 | | 108.0(2) | |  | C27 | | | Fe3 | | C20 | | 119.86(11) | |
| C44 | C45 | Fe1 | | 69.85(14) | |  | C27 | | | Fe3 | | C21 | | 108.51(11) | |
| C46 | C45 | Fe1 | | 69.55(13) | |  | C27 | | | Fe3 | | C22 | | 127.22(11) | |
| C45 | C46 | Fe1 | | 69.32(14) | |  | C27 | | | Fe3 | | C23 | | 164.31(12) | |
| C47 | C46 | C45 | | 108.1(2) | |  | C27 | | | Fe3 | | C24 | | 68.30(10) | |
| C47 | C46 | Fe1 | | 70.42(13) | |  | C27 | | | Fe3 | | C25 | | 68.15(9) | |
| C31 | C47 | Fe1 | | 131.00(15) | |  | C27 | | | Fe3 | | C26 | | 40.95(8) | |
| C46 | C47 | C31 | | 122.6(2) | |  | C28 | | | Fe3 | | C20 | | 153.79(12) | |
| C46 | C47 | Fe1 | | 68.74(12) | |  | C28 | | | Fe3 | | C21 | | 119.13(11) | |
| C48 | C47 | C31 | | 129.6(2) | |  | C28 | | | Fe3 | | C22 | | 107.48(11) | |
| C48 | C47 | C46 | | 107.6(2) | |  | C28 | | | Fe3 | | C23 | | 126.31(13) | |
| C48 | C47 | Fe1 | | 69.47(13) | |  | C28 | | | Fe3 | | C24 | | 40.36(10) | |
| C44 | C48 | Fe1 | | 68.97(13) | |  | C28 | | | Fe3 | | C25 | | 68.07(10) | |
| C47 | C48 | C44 | | 108.0(2) | |  | C28 | | | Fe3 | | C26 | | 68.71(9) | |
| C47 | C48 | Fe1 | | 69.92(13) | |  | C28 | | | Fe3 | | C27 | | 40.91(9) | |
| C39 | Fe1 | C41 | | 68.47(9) | |  | C6 | | | P1 | | C12 | | 101.44(10) | |
| C39 | Fe1 | C42 | | 68.44(10) | |  | C6 | | | P1 | | C18 | | 104.42(10) | |
| C39 | Fe1 | C43 | | 40.67(10) | |  | C18 | | | P1 | | C12 | | 98.92(9) | |
| C39 | Fe1 | C47 | | 124.47(9) | |  |  | | |  | |  | |  | |
