# Supplementary material for: Rhodium-catalyzed selective direct arylation of phosphines with aryl bromides
Source: Nat Commun. 2022 May 25;13:2934. doi: 10.1038/s41467-022-30697-7 (PMC9132997; doi:10.1038/s41467-022-30697-7)
Supplement: Supplementary file 5 — Supplementary Data 2 [file 41467_2022_30697_MOESM5_ESM.docx]

**Table S1. The calculated energies of stationary points (in Hartree/Particle)**

| **Structure** | **E_ele_** | **E_0_** | **E** | **H** | **G** |
| --- | --- | --- | --- | --- | --- |
| **1a** | -1035.909820 | -1035.635511 | -1035.619569 | -1035.618625 | -1035.682375 |
| **2a** | -3077.199256 | -3076.962550 | -3076.948694 | -3076.947749 | -3077.003529 |
| **INT1A** | -2446.345026 | -2445.776843 | -2445.737884 | -2445.736940 | -2445.853268 |
| **TS2A** | -5523.512636 | -5522.708757 | -5522.653856 | -5522.652911 | -5522.806740 |
| **INT2A** | -4487.584984 | -4487.055943 | -4487.018271 | -4487.017327 | -4487.131488 |
| **TS3A** | -4487.578173 | -4487.050142 | -4487.013039 | -4487.012095 | -4487.122731 |
| **INT3A** | -4487.631882 | -4487.101716 | -4487.064630 | -4487.063686 | -4487.173108 |
| **TS2B** | -2446.272462 | -2445.710237 | -2445.671478 | -2445.670534 | -2445.786761 |
| **INT2B** | -2446.300912 | -2445.736274 | -2445.697648 | -2445.696704 | -2445.811698 |
| **TS3B** | -2446.262839 | -2445.699884 | -2445.661517 | -2445.660573 | -2445.772897 |
| **INT3B** | -2446.322737 | -2445.755860 | -2445.716161 | -2445.715217 | -2445.834382 |
| **TS4A** | -4487.573479 | -4487.049782 | -4487.012490 | -4487.011546 | -4487.122880 |
| **INT4A** | -4487.624623 | -4487.094607 | -4487.057312 | -4487.056368 | -4487.166128 |
| **INT3A-D** | -4487.631880 | -4487.111786 | -4487.074288 | -4487.073344 | -4487.183470 |
| **TS4A-D** | -4487.573479 | -4487.058374 | -4487.020735 | -4487.019791 | -4487.131707 |
| K_2_CO_3_ | -1463.713050 | -1463.696403 | -1463.689705 | -1463.688761 | -1463.729454 |
| **INT5A** | -5951.386312 | -5950.839541 | -5950.793217 | -5950.792273 | -5950.925233 |
| **TS6A** | -5951.381491 | -5950.834077 | -5950.789075 | -5950.788131 | -5950.917216 |
| **INT6A** | -5951.406714 | -5950.859262 | -5950.813163 | -5950.812218 | -5950.945785 |
| **TS7A** | -5951.401838 | -5950.855180 | -5950.809502 | -5950.808558 | -5950.939782 |
| **INT7A** | -5951.427087 | -5950.877988 | -5950.832069 | -5950.831125 | -5950.963480 |
| **INT8A** | -9028.652095 | -9027.865002 | -9027.803442 | -9027.802498 | -9027.968906 |
| **INT9A** | -4990.180611 | -4989.424702 | -4989.374316 | -4989.373372 | -4989.512984 |
| KBr-KHCO_3_ | -4038.425134 | -4038.395685 | -4038.386164 | -4038.385220 | -4038.433848 |
| **TS10A** | -4990.168613 | -4989.413774 | -4989.364012 | -4989.363068 | -4989.499379 |
| **INT10A** | -4990.225643 | -4989.468675 | -4989.418902 | -4989.417958 | -4989.553570 |
| **TS11** | -6026.129483 | -6025.097460 | -6025.030485 | -6025.029541 | -6025.206868 |
| **3aa** | -1538.502636 | -1538.001705 | -1537.972828 | -1537.971884 | -1538.063834 |
| **TS11A** | -4990.174764 | -4989.423246 | -4989.373925 | -4989.372981 | -4989.506760 |
| **INT11A** | -4990.219266 | -4989.462090 | -4989.412643 | -4989.411699 | -4989.545091 |
| **TS11B** | -4990.147965 | -4989.396470 | -4989.346846 | -4989.345902 | -4989.481870 |
| **INT11B** | -4990.217431 | -4989.461157 | -4989.410984 | -4989.410040 | -4989.547140 |
| **TS11C** | -4990.150264 | -4989.398865 | -4989.349027 | -4989.348083 | -4989.484835 |
| **INT11C** | -4990.219153 | -4989.462521 | -4989.412216 | -4989.411271 | -4989.549323 |
| **INT12A** | -6453.976099 | -6453.201015 | -6453.142979 | -6453.142035 | -6453.295673 |
| **TS13A** | -6453.978991 | -6453.203960 | -6453.147122 | -6453.146178 | -6453.296278 |
| **INT13A** | -6454.014924 | -6453.239562 | -6453.181516 | -6453.180572 | -6453.335956 |
| **TS14A** | -6453.955123 | -6453.181348 | -6453.123535 | -6453.122591 | -6453.279164 |
| **INT14A** | -6454.011260 | -6453.234557 | -6453.176566 | -6453.175622 | -6453.333014 |
| **2j** | -2920.029510 | -2919.905672 | -2919.897455 | -2919.896511 | -2919.939885 |
| **TS2A-2j** | -5366.340505 | -5365.649886 | -5365.600419 | -5365.599475 | -5365.743712 |
| **INT2A-2j** | -4330.416663 | -4330.000600 | -4329.968564 | -4329.967619 | -4330.069760 |
| **TS3A-2j** | -4330.408441 | -4329.993605 | -4329.962014 | -4329.961070 | -4330.060864 |
| **INT3A-2j** | -4330.461091 | -4330.044046 | -4330.012526 | -4330.011581 | -4330.109388 |
| **TS4A-2j** | -4330.402447 | -4329.991841 | -4329.960147 | -4329.959203 | -4330.058587 |
| **INT4A-2j** | -4330.453376 | -4330.036699 | -4330.004876 | -4330.003932 | -4330.102739 |
| **INT5A-2j** | -5794.215464 | -5793.781339 | -5793.740862 | -5793.739918 | -5793.859256 |
| **TS6A-2j** | -5794.211462 | -5793.777185 | -5793.737705 | -5793.736761 | -5793.854493 |
| **INT6A-2j** | -5794.233636 | -5793.799340 | -5793.758707 | -5793.757763 | -5793.878674 |
| **TS7A-2j** | -5794.229386 | -5793.795653 | -5793.755636 | -5793.754691 | -5793.874148 |
| **INT7A-2j** | -5794.251006 | -5793.815231 | -5793.774601 | -5793.773657 | -5793.897661 |
| **INT8A-2j** | -8714.308719 | -8713.747831 | -8713.697065 | -8713.696121 | -8713.842574 |
| **INT9A-2j** | -4675.840967 | -4675.311171 | -4675.272016 | -4675.271072 | -4675.386762 |
| **TS10A-2j** | -4675.821130 | -4675.292660 | -4675.253879 | -4675.252935 | -4675.368326 |
| **INT10A-2j** | -4675.880979 | -4675.350191 | -4675.311591 | -4675.310646 | -4675.422702 |
| **TS11A-2j** | -4675.826546 | -4675.299233 | -4675.261033 | -4675.260089 | -4675.371385 |
| **INT11A-2j** | -4675.888939 | -4675.358436 | -4675.319505 | -4675.318561 | -4675.432515 |
| **TS11B-2j** | -4675.808334 | -4675.282613 | -4675.244333 | -4675.243389 | -4675.354515 |
| **INT11B-2j** | -4675.876997 | -4675.346807 | -4675.307806 | -4675.306862 | -4675.420395 |
| **TS11C-2j** | -4675.806572 | -4675.281268 | -4675.242622 | -4675.241677 | -4675.354981 |
| **INT11C-2j** | -4675.878422 | -4675.347997 | -4675.308798 | -4675.307853 | -4675.423345 |
| **INT12A-2j** | -6139.645997 | -6139.096962 | -6139.049640 | -6139.048696 | -6139.182853 |
| **TS13A-2j** | -6139.641265 | -6139.092491 | -6139.046295 | -6139.045351 | -6139.175877 |
| **INT13A-2j** | -6139.681106 | -6139.132315 | -6139.084619 | -6139.083675 | -6139.220128 |
| **TS14A-2j** | -6139.645942 | -6139.097902 | -6139.050838 | -6139.049894 | -6139.184348 |
| **INT14A-2j** | -6139.663080 | -6139.113274 | -6139.065332 | -6139.064388 | -6139.205280 |
| **INT15A-2j** | -9059.734155 | -9059.060072 | -9059.001860 | -9059.000916 | -9059.163834 |
| **INT16A-2j** | -5021.258021 | -5020.614598 | -5020.568149 | -5020.567205 | -5020.699606 |
| **TS17A-2j** | -5021.244241 | -5020.602139 | -5020.556079 | -5020.555135 | -5020.685899 |
| **INT17A-2j** | -5021.301532 | -5020.656854 | -5020.611108 | -5020.610163 | -5020.737454 |
| **TS18-2j** | -6057.218821 | -6056.299138 | -6056.236113 | -6056.235169 | -6056.403750 |
| **3aj** | -1726.752452 | -1726.250987 | -1726.220345 | -1726.219401 | -1726.316063 |
| **2n** | -2805.538296 | -2805.447385 | -2805.441696 | -2805.440752 | -2805.478255 |
| **INT1A-2n** | -2415.423611 | -2414.745201 | -2414.702521 | -2414.701577 | -2414.825823 |
| **TS2A-2n** | -5220.929322 | -5220.160670 | -5220.110337 | -5220.109393 | -5220.256668 |
| **INT2A-2n** | -4185.006660 | -4184.513881 | -4184.480778 | -4184.479834 | -4184.585063 |
| **TS3A-2n** | -4185.002346 | -4184.509335 | -4184.477020 | -4184.476075 | -4184.577401 |
| **INT3A-2n** | -4185.041817 | -4184.547747 | -4184.514995 | -4184.514051 | -4184.615553 |
| **TS4A-2n** | -4184.986517 | -4184.497315 | -4184.465087 | -4184.464143 | -4184.563922 |
| **INT4A-2n** | -3951.449383 | -3951.094111 | -3951.069078 | -3951.068134 | -3951.153918 |
| *^t^*BuOH | -233.581863 | -233.445703 | -233.438966 | -233.438022 | -233.474719 |
| **TS2B-2n** | -2415.392615 | -2414.720198 | -2414.678579 | -2414.677635 | -2414.798716 |
| **INT2B-2n** | -2415.394617 | -2414.720578 | -2414.677868 | -2414.676924 | -2414.800911 |
| **TS3B-2n** | -2415.366106 | -2414.693711 | -2414.651303 | -2414.650359 | -2414.773970 |
| **INT3B-2n** | -2181.814317 | -2181.275020 | -2181.239937 | -2181.238993 | -2181.348830 |
| **INT4B-2n** | -4987.374776 | -4986.743743 | -4986.700972 | -4986.700028 | -4986.829282 |
| **TS5B-2n** | -4987.364148 | -4986.733796 | -4986.691826 | -4986.690882 | -4986.814192 |
| **INT5B-2n** | -4987.407492 | -4986.775458 | -4986.733086 | -4986.732142 | -4986.856146 |
| **TS6B-2n** | -4987.388073 | -4986.756714 | -4986.714960 | -4986.714015 | -4986.836241 |
| **INT6B-2n** | -4987.448199 | -4986.814283 | -4986.772571 | -4986.771627 | -4986.894590 |
| LiO*^t^*Bu | -240.546052 | -240.419608 | -240.411963 | -240.411019 | -240.450225 |
| **INT7B-2n** | -5228.040853 | -5227.278110 | -5227.227465 | -5227.226521 | -5227.368245 |
| **TS8B-2n** | -5228.029167 | -5227.267282 | -5227.217025 | -5227.216081 | -5227.356025 |
| **INT8B-2n** | -5228.043705 | -5227.281343 | -5227.230362 | -5227.229417 | -5227.372356 |
| **TS9A-2n** | -8033.546321 | -8032.693091 | -8032.634667 | -8032.633723 | -8032.797352 |
| **INT9A-2n** | -6997.624827 | -6997.047411 | -6997.006124 | -6997.005180 | -6997.129232 |
| **TS10A-2n** | -6997.613224 | -6997.035983 | -6996.995573 | -6996.994629 | -6997.113189 |
| **INT10A-2n** | -6997.651261 | -6997.073228 | -6997.032133 | -6997.031188 | -6997.152622 |
| **TS9B-2n** | -5228.016395 | -5227.257895 | -5227.207835 | -5227.206891 | -5227.346356 |
| **INT9B-2n** | -5228.027261 | -5227.267357 | -5227.216974 | -5227.216030 | -5227.357599 |
| **TS10B-2n** | -5227.994745 | -5227.237572 | -5227.186957 | -5227.186012 | -5227.328266 |
| **INT10B-2n** | -2412.764373 | -2412.143637 | -2412.104184 | -2412.103240 | -2412.221878 |
| LiBr-*^t^*BuOH | -2815.245217 | -2815.105717 | -2815.095328 | -2815.094384 | -2815.144626 |
| **TS9C-2n** | -5228.007723 | -5227.250521 | -5227.199989 | -5227.199045 | -5227.339959 |
| **INT9C-2n** | -5228.011165 | -5227.252017 | -5227.201302 | -5227.200358 | -5227.341766 |
| **TS10C-2n** | -5227.977762 | -5227.220963 | -5227.170270 | -5227.169326 | -5227.311239 |
| **INT10C-2n** | -2412.745682 | -2412.125537 | -2412.085752 | -2412.084808 | -2412.204763 |
| **INT11B-2n** | -5218.315147 | -5217.602810 | -5217.555410 | -5217.554465 | -5217.693653 |
| **TS12B-2n** | -5218.303920 | -5217.591892 | -5217.545556 | -5217.544612 | -5217.676292 |
| **INT12B-2n** | -5218.338854 | -5217.625237 | -5217.578554 | -5217.577610 | -5217.709829 |
| **TS13B-2n** | -5218.325949 | -5217.612288 | -5217.566698 | -5217.565754 | -5217.693275 |
| **INT13B-2n** | -5218.382934 | -5217.668122 | -5217.621731 | -5217.620787 | -5217.753730 |
| **INT14B-2n** | -5458.967915 | -5458.124066 | -5458.068872 | -5458.067928 | -5458.218333 |
| **TS15B-2n** | -5458.955999 | -5458.113201 | -5458.058309 | -5458.057365 | -5458.207238 |
| **INT15B-2n** | -5458.973428 | -5458.130231 | -5458.074583 | -5458.073639 | -5458.227222 |
| **TS16B-2n** | -5458.940610 | -5458.101423 | -5458.046525 | -5458.045581 | -5458.196283 |
| **INT16B-2n** | -5458.948571 | -5458.107718 | -5458.052541 | -5458.051596 | -5458.204000 |
| **TS17B-2n** | -5458.921295 | -5458.083595 | -5458.028150 | -5458.027206 | -5458.179890 |
| **INT17B-2n** | -2643.689814 | -2642.988325 | -2642.944109 | -2642.943165 | -2643.072181 |
| **INT18B-2n** | -5449.241114 | -5448.448165 | -5448.395973 | -5448.395028 | -5448.544994 |
| **TS19B-2n** | -5449.230152 | -5448.437244 | -5448.386202 | -5448.385258 | -5448.527103 |
| **INT19B-2n** | -5449.263403 | -5448.469112 | -5448.417625 | -5448.416680 | -5448.560168 |
| **TS20B-2n** | -5449.253523 | -5448.459157 | -5448.408725 | -5448.407781 | -5448.546347 |
| **INT20B-2n** | -5449.308163 | -5448.512317 | -5448.461290 | -5448.460346 | -5448.600558 |
| **INT21B-2n** | -5689.889713 | -5688.964452 | -5688.904868 | -5688.903924 | -5689.062858 |
| **TS22B-2n** | -5689.877452 | -5688.953678 | -5688.894194 | -5688.893249 | -5689.053017 |
| **INT22B-2n** | -5689.904215 | -5688.979669 | -5688.919458 | -5688.918514 | -5689.080796 |
| **TS23B-2n** | -6725.805568 | -6724.604425 | -6724.527586 | -6724.526641 | -6724.725056 |
| **INT23B-2n** | -4997.117822 | -4996.436268 | -4996.389888 | -4996.388944 | -4996.522840 |
| **3an** | -1728.699814 | -1728.182843 | -1728.152824 | -1728.151880 | -1728.246226 |
| **TS2A-1** | -5251.853991 | -5251.195900 | -5251.149163 | -5251.148219 | -5251.285945 |
| **INT2A-1** | -4215.928571 | -4215.545012 | -4215.515636 | -4215.514692 | -4215.612068 |
| **TS3A-1** | -4215.919828 | -4215.537465 | -4215.508590 | -4215.507646 | -4215.601792 |
| **INT3A-1** | -4215.973716 | -4215.589324 | -4215.560218 | -4215.559274 | -4215.652854 |
| **TS4A-1** | -4215.908067 | -4215.529856 | -4215.501097 | -4215.500153 | -4215.592796 |
| **INT4A-1** | -4215.970103 | -4215.586729 | -4215.557243 | -4215.556299 | -4215.651896 |
