# Supplementary material for: Rhodium-catalyzed selective direct arylation of phosphines with aryl bromides
Source: Nat Commun. 2022 May 25;13:2934. doi: 10.1038/s41467-022-30697-7 (PMC9132997; doi:10.1038/s41467-022-30697-7)
Supplement: Supplementary file 6 — Supplementary Data 3 [file 41467_2022_30697_MOESM6_ESM.docx]

**Cartesian coordinates of the optimized structures**

**1a**

C -0.890789 3.242932 1.276217

C -0.264782 2.108063 0.754903

C -0.758753 1.487819 -0.403202

C -1.889104 2.040317 -1.029862

C -2.521254 3.166806 -0.503204

C -2.021299 3.772931 0.651727

H -0.493543 3.712389 2.172743

H 0.614162 1.706146 1.249808

H -2.272823 1.585462 -1.940341

H -3.396599 3.577261 -0.999993

H -2.506133 4.656574 1.058400

P 0.000322 0.000118 -1.207231

C 1.668363 -0.086986 -0.403603

C 1.956837 -0.821261 0.757435

C 2.714063 0.609927 -1.033287

C 3.252630 -0.848181 1.278685

H 1.168250 -1.378116 1.254607

C 4.005767 0.592413 -0.506721

H 2.513701 1.166345 -1.946157

C 4.278789 -0.139536 0.651209

H 3.458999 -1.424128 2.177368

H 4.800493 1.140771 -1.005931

H 5.286470 -0.162675 1.057791

C -0.908826 -1.400347 -0.402785

C -0.836083 -2.652528 -1.037030

C -1.679851 -1.284207 0.764321

C -1.496824 -3.762062 -0.509354

H -0.260440 -2.755802 -1.954097

C -2.350851 -2.392431 1.286781

H -1.759602 -0.323915 1.264779

C -2.258605 -3.633672 0.654377

H -1.425796 -4.723168 -1.012070

H -2.946039 -2.284505 2.190127

H -2.782491 -4.494389 1.061784

**2a**

Br -2.169267 -1.610614 0.000304

C -0.822588 -0.257689 0.000059

C -1.199974 1.097692 -0.000176

C 0.513382 -0.630597 0.000116

C -0.179703 2.052831 -0.000373

C 1.547846 0.322938 -0.000051

H 0.742793 -1.690421 0.000284

C 1.164619 1.666859 -0.000318

H -0.423131 3.109360 -0.000582

H 1.914141 2.450255 -0.000487

O -2.528919 1.379242 -0.000230

C -2.926824 2.740850 -0.000284

H -2.568046 3.265623 -0.895496

H -4.018020 2.730255 -0.000185

H -2.567877 3.265741 0.894788

C 3.015222 -0.141671 -0.000035

C 3.288724 -0.993554 1.262460

C 3.288329 -0.994754 -1.261805

C 3.997579 1.044785 -0.000743

H 3.116131 -0.409933 2.173986

H 2.644367 -1.877997 1.305933

H 4.329654 -1.339842 1.271621

H 3.115436 -0.412005 -2.173833

H 4.329256 -1.341051 -1.270972

H 2.643948 -1.879234 -1.304216

H 5.027511 0.670696 -0.000679

H 3.874827 1.674533 -0.889377

H 3.875028 1.675414 0.887294

**INT1A**

P -1.842302 -0.095412 -0.037095

C -1.934240 1.160622 1.339071

C -1.976153 2.896703 3.560237

C -1.473068 0.733299 2.597274

C -2.408151 2.473427 1.213373

C -2.428031 3.335304 2.315261

C -1.499063 1.589161 3.698301

H -1.074371 -0.273700 2.693792

H -2.754881 2.834224 0.250365

H -2.794315 4.352771 2.195112

H -1.137173 1.238335 4.661818

H -1.989983 3.568015 4.416021

C -2.298416 0.900603 -1.552460

C -2.862905 2.340964 -3.908201

C -3.583237 1.418059 -1.789413

C -1.310379 1.100299 -2.526981

C -1.586998 1.818345 -3.692637

C -3.862537 2.135350 -2.954129

H -4.375808 1.245264 -1.067203

H -0.328546 0.665357 -2.365145

H -0.805212 1.961702 -4.434775

H -4.864484 2.525298 -3.120602

H -3.081904 2.895239 -4.818268

C -3.446783 -1.004825 0.242998

C -3.609513 -2.260392 -0.367055

C -4.503351 -0.485915 1.010202

C -4.809652 -2.960611 -0.227752

H -2.774683 -2.710951 -0.893047

C -5.700816 -1.191937 1.148874

H -4.394803 0.472430 1.508976

C -5.859414 -2.430294 0.524648

H -4.912947 -3.936135 -0.696772

H -6.506257 -0.773503 1.749169

H -6.789672 -2.983631 0.635592

Rh 0.020710 -1.401795 -0.093782

P 1.756945 0.082115 -0.020152

C 2.724772 -0.047886 1.573103

C 3.408634 1.011776 2.187957

C 2.782739 -1.324634 2.162793

C 4.122295 0.809011 3.373125

H 3.382210 2.005263 1.750995

C 3.505965 -1.523237 3.339835

H 2.270698 -2.155339 1.679070

C 4.172142 -0.458703 3.953233

H 4.639560 1.645293 3.839380

H 3.544226 -2.517058 3.779751

H 4.727309 -0.616745 4.875545

C 3.097618 -0.353813 -1.244091

C 2.834477 -1.323078 -2.221694

C 4.369217 0.242413 -1.208486

C 3.814882 -1.671449 -3.155696

H 1.872480 -1.824891 -2.214884

C 5.345990 -0.103252 -2.142062

H 4.599480 0.979528 -0.443900

C 5.068459 -1.060852 -3.122351

H 3.596906 -2.432385 -3.901128

H 6.325282 0.369084 -2.101034

H 5.831267 -1.335341 -3.847985

C 1.637472 1.924956 -0.274384

C 1.993497 2.530186 -1.491610

C 1.072737 2.738566 0.723857

C 1.808311 3.898842 -1.697977

H 2.426720 1.929647 -2.284819

C 0.897546 4.108506 0.522109

H 0.766386 2.301128 1.668750

C 1.265338 4.696145 -0.689742

H 2.093966 4.340944 -2.649989

H 0.460387 4.711142 1.314078

H 1.124298 5.762816 -0.848512

C 0.294414 -3.908495 -0.119193

O -0.878684 -3.273148 -0.121519

O 1.311783 -3.036319 -0.096911

O 0.428083 -5.125850 -0.138253

**TS2A**

P 0.230478 1.918880 -0.252346

C 1.108797 2.104817 1.375849

C 2.391390 2.156930 3.890718

C 0.437819 1.688343 2.539881

C 2.440316 2.530490 1.495799

C 3.075612 2.556106 2.741557

C 1.067421 1.723019 3.783941

H -0.585942 1.332689 2.474037

H 2.986962 2.848343 0.614080

H 4.106230 2.897803 2.810580

H 0.519719 1.401521 4.666208

H 2.885288 2.180621 4.859558

C 1.306101 2.913377 -1.408637

C 2.909241 4.331231 -3.232543

C 1.576672 4.278262 -1.214127

C 1.834086 2.274523 -2.538504

C 2.630811 2.980680 -3.444598

C 2.378122 4.981053 -2.114199

H 1.153664 4.796826 -0.357562

H 1.584031 1.230331 -2.705513

H 3.024134 2.472968 -4.322071

H 2.581523 6.036970 -1.948156

H 3.528091 4.880632 -3.938937

C -1.197065 3.106524 -0.082116

C -2.163952 3.051241 -1.102011

C -1.370157 4.025319 0.963016

C -3.273778 3.895734 -1.071700

H -2.037366 2.337371 -1.912491

C -2.487442 4.865870 0.994085

H -0.639886 4.081055 1.764765

C -3.441808 4.803833 -0.022012

H -4.015562 3.833141 -1.864074

H -2.609602 5.568045 1.816407

H -4.312831 5.455015 0.004797

C -0.931531 -0.832210 -3.378913

O -0.910940 0.429917 -2.928771

O -0.632391 -1.689556 -2.409631

Br 1.204384 -1.585936 0.801125

O -1.189326 -1.143114 -4.538863

C 2.979303 -1.857876 0.118074

C 4.068006 -1.335393 0.799523

C 3.136076 -2.564490 -1.089980

C 5.379035 -1.483455 0.307736

H 3.880123 -0.784910 1.715445

C 4.435133 -2.703269 -1.590190

C 5.529947 -2.173041 -0.900029

H 4.593432 -3.239552 -2.520572

H 6.515840 -2.313410 -1.330351

O 2.028395 -3.064417 -1.666230

C 1.990369 -3.235884 -3.082839

H 0.934045 -3.178084 -3.346159

H 2.527817 -2.422864 -3.584407

H 2.429863 -4.201007 -3.372623

C 6.558735 -0.879658 1.091113

C 6.396098 0.657176 1.167384

C 6.585141 -1.460912 2.524783

C 7.915482 -1.186458 0.428680

H 6.396191 1.098981 0.164333

H 5.456799 0.940746 1.652756

H 7.220935 1.104053 1.738602

H 6.708665 -2.550055 2.502335

H 7.418747 -1.033991 3.097794

H 5.660664 -1.242282 3.068918

H 8.725778 -0.748119 1.023696

H 8.099577 -2.265048 0.358970

H 7.979999 -0.763152 -0.580180

C -2.810930 -2.817822 0.585971

C -3.133775 -5.615560 0.597138

C -3.555543 -3.451241 1.595319

C -2.230974 -3.607584 -0.423983

C -2.400456 -4.994414 -0.415597

C -3.710779 -4.838612 1.604367

H -4.013710 -2.862274 2.384620

H -1.656519 -3.129888 -1.217067

H -1.948045 -5.589899 -1.205172

H -4.288367 -5.311048 2.396703

H -3.255617 -6.696751 0.603307

C -2.883842 -0.366964 2.150693

C -3.047187 0.780340 4.738433

C -2.275271 -0.982656 3.264436

C -3.551779 0.854992 2.367207

C -3.632395 1.417779 3.641971

C -2.365022 -0.424211 4.539220

H -1.727944 -1.912084 3.131647

H -4.009500 1.372882 1.530000

H -4.155572 2.362070 3.774732

H -1.897672 -0.932398 5.380179

H -3.114460 1.217816 5.731647

C -4.106820 -0.512095 -0.461398

C -3.999579 -0.002092 -1.763179

C -5.386540 -0.708098 0.086328

C -5.150462 0.304107 -2.498100

H -3.019018 0.156161 -2.205295

C -6.531337 -0.391437 -0.643552

H -5.488711 -1.111513 1.090007

C -6.414984 0.115385 -1.941843

H -5.047100 0.687234 -3.510212

H -7.514124 -0.546828 -0.202990

H -7.307938 0.355613 -2.515272

P -2.543020 -0.979953 0.439410

Rh -0.279149 -0.144061 -1.023410

**INT2A**

P -1.864349 -0.548599 0.046323

C -1.004709 -2.133419 -0.421861

C 0.490613 -4.417974 -1.144844

C -1.365673 -2.910029 -1.532546

C 0.136920 -2.512201 0.307241

C 0.867155 -3.648690 -0.039487

C -0.623274 -4.038688 -1.893535

H -2.236440 -2.635216 -2.119488

H 0.450717 -1.910497 1.156314

H 1.730094 -3.936892 0.556672

H -0.923741 -4.624239 -2.759830

H 1.062872 -5.301258 -1.419282

C -2.380145 -0.909170 1.803867

C -3.199763 -1.278803 4.471564

C -2.466671 -2.197368 2.355779

C -2.709376 0.196963 2.608301

C -3.121507 0.006399 3.928783

C -2.869511 -2.380912 3.681630

H -2.204583 -3.065044 1.757071

H -2.638367 1.199550 2.187345

H -3.374687 0.871486 4.537439

H -2.922890 -3.386298 4.095002

H -3.512493 -1.420508 5.504149

C -3.501010 -0.717333 -0.836718

C -3.826829 0.265071 -1.782487

C -4.423646 -1.746554 -0.587775

C -5.039321 0.211621 -2.475452

H -3.120950 1.076925 -1.943353

C -5.633029 -1.802793 -1.281790

H -4.201687 -2.503695 0.159449

C -5.942635 -0.823258 -2.230088

H -5.279478 0.986026 -3.200188

H -6.337683 -2.606298 -1.077295

H -6.888407 -0.863668 -2.766483

Rh -0.865067 1.438035 -0.240013

C -1.652214 3.715496 0.454446

O -2.339809 2.563013 0.595568

O -0.503969 3.498957 -0.167751

Br 1.160673 0.615463 -1.622638

O -2.058040 4.797945 0.862719

C 2.842657 0.724345 -0.666569

C 3.611349 -0.413404 -0.480433

C 3.245055 1.976853 -0.165257

C 4.825636 -0.367276 0.233266

H 3.240237 -1.351230 -0.881817

C 4.439726 2.026327 0.561831

C 5.209720 0.874478 0.751029

H 4.777559 2.974224 0.969245

H 6.130781 0.969696 1.316445

O 2.460936 3.030747 -0.441135

C 2.421904 4.146738 0.447919

H 1.429941 4.576254 0.299316

H 2.523344 3.815488 1.488322

H 3.221650 4.862723 0.210366

C 5.653169 -1.652086 0.420000

C 4.819479 -2.700091 1.194586

C 6.037641 -2.229928 -0.962827

C 6.952128 -1.398843 1.208865

H 4.540587 -2.321828 2.184683

H 3.896389 -2.951870 0.663059

H 5.392726 -3.626868 1.330263

H 6.641555 -1.512863 -1.531084

H 6.621690 -3.152456 -0.846290

H 5.152792 -2.467642 -1.562067

H 7.510481 -2.336857 1.314814

H 7.604121 -0.679923 0.699035

H 6.747596 -1.019107 2.216421

**TS3A**

P -1.557629 0.198198 -0.020159

C -1.569389 1.487099 -1.358774

C -1.345080 3.305005 -3.511274

C -1.193533 2.822441 -1.141879

C -1.813418 1.078515 -2.683047

C -1.713174 1.977201 -3.744292

C -1.080721 3.721764 -2.206617

H -0.994690 3.169156 -0.132954

H -2.084495 0.045537 -2.882065

H -1.913549 1.636566 -4.757487

H -0.790087 4.751352 -2.009951

H -1.259424 4.004338 -4.339584

C -3.274271 -0.523843 -0.140394

C -5.814741 -1.733052 -0.171047

C -4.350138 0.088428 -0.800486

C -3.487397 -1.752248 0.510660

C -4.751040 -2.344785 0.497794

C -5.610755 -0.515055 -0.820805

H -4.206514 1.034767 -1.313802

H -2.656940 -2.228995 1.028427

H -4.900545 -3.293299 1.008163

H -6.431831 -0.030599 -1.345584

H -6.795583 -2.203553 -0.187078

C -1.747989 1.200497 1.538509

C -0.914297 0.891759 2.623452

C -2.723482 2.199856 1.694605

C -1.043215 1.585549 3.830917

H -0.202409 0.077659 2.522925

C -2.842573 2.898090 2.896257

H -3.396905 2.433909 0.874180

C -1.998245 2.593111 3.968123

H -0.397005 1.328079 4.666539

H -3.598662 3.673664 2.999250

H -2.094231 3.133001 4.907791

C 0.351748 -3.186127 1.797507

O -0.501272 -2.142205 1.867666

O 0.996928 -3.181163 0.644823

Rh 0.031687 -1.427855 0.031390

O 0.491874 -4.003887 2.699982

Br 1.100102 -1.044657 -2.341253

C 2.223862 -0.462432 -0.763211

C 3.286176 -1.325261 -0.405245

C 2.273936 0.897437 -0.445863

C 4.271566 -0.811708 0.437689

C 3.290165 1.421278 0.371156

H 1.479067 1.533978 -0.816897

C 4.272931 0.533163 0.824669

H 5.071548 -1.462714 0.776549

H 5.076536 0.877036 1.466441

O 3.315238 -2.571116 -0.938918

C 3.847963 -3.626840 -0.145735

H 3.643437 -4.542285 -0.704698

H 4.935779 -3.526791 -0.012563

H 3.327137 -3.671551 0.813483

C 3.280304 2.917156 0.740753

C 3.194716 3.778543 -0.541641

C 2.060186 3.221521 1.640251

C 4.553134 3.335224 1.502983

H 4.048988 3.583211 -1.200770

H 2.281759 3.576329 -1.110455

H 3.197363 4.846837 -0.286092

H 2.106075 2.644204 2.570175

H 2.024055 4.288722 1.899604

H 1.120918 2.959573 1.144906

H 4.518432 4.409876 1.721133

H 4.646226 2.808055 2.459106

H 5.458958 3.140711 0.916501

**INT3A**

P -1.218790 0.607655 0.105910

C -0.303172 2.092756 -0.521411

C 1.023666 4.350344 -1.546227

C -0.104463 3.229002 0.278893

C 0.177284 2.097169 -1.841978

C 0.830504 3.221780 -2.347368

C 0.557280 4.349447 -0.231147

H -0.463605 3.244280 1.302086

H 0.053249 1.209568 -2.457880

H 1.197464 3.208706 -3.370567

H 0.705659 5.219858 0.403578

H 1.538848 5.222213 -1.942821

C -2.885089 0.788719 -0.671564

C -5.455768 1.041520 -1.763962

C -3.320184 2.020118 -1.187575

C -3.747203 -0.319633 -0.707567

C -5.026053 -0.183776 -1.251048

C -4.600188 2.144079 -1.730936

H -2.662105 2.882908 -1.173465

H -3.403821 -1.274737 -0.312718

H -5.684241 -1.048485 -1.279012

H -4.923589 3.101744 -2.132489

H -6.451579 1.137276 -2.191434

C -1.492041 1.014018 1.884610

C -0.437558 0.804245 2.788935

C -2.699804 1.547657 2.354150

C -0.587512 1.146583 4.131748

H 0.487639 0.359543 2.440883

C -2.849247 1.877789 3.703578

H -3.527893 1.701841 1.670387

C -1.793072 1.681296 4.592996

H 0.233406 0.973906 4.822776

H -3.794562 2.283161 4.056248

H -1.910868 1.933629 5.644335

C -1.585562 -2.595705 1.668946

O -0.412489 -1.926187 1.798523

O -2.034444 -2.518260 0.429282

Rh -0.264435 -1.394225 -0.164521

O -2.107007 -3.188460 2.605120

Br -0.450232 -1.303148 -2.741135

C 1.716120 -0.904035 -0.143063

C 2.300924 -2.176864 -0.137984

C 2.575114 0.186494 -0.030115

C 3.665126 -2.385634 -0.020960

C 3.976284 0.032233 0.096249

H 2.161930 1.190169 -0.035219

C 4.500489 -1.264790 0.100115

H 4.086774 -3.387834 -0.029638

H 5.568903 -1.429645 0.192156

O 1.357070 -3.201950 -0.308125

C 1.349812 -4.240678 0.679843

H 0.529858 -4.907093 0.405900

H 2.295492 -4.796149 0.660235

H 1.163900 -3.816407 1.670477

C 4.867282 1.284700 0.216604

C 4.740976 2.134479 -1.070481

C 4.420800 2.133553 1.431219

C 6.355562 0.933951 0.409714

H 5.073746 1.563135 -1.945195

H 3.706575 2.442715 -1.249917

H 5.357881 3.041330 -0.998874

H 4.502079 1.555966 2.359730

H 5.051144 3.028147 1.528702

H 3.382562 2.465599 1.334452

H 6.946045 1.854180 0.502732

H 6.518125 0.340244 1.316955

H 6.753645 0.369710 -0.441581

**TS2B**

P -2.534257 0.148182 -0.066899

C -3.522469 1.276939 -1.131620

C -4.952106 2.936595 -2.890653

C -3.503075 1.032999 -2.516224

C -4.251871 2.370970 -0.642798

C -4.960555 3.197282 -1.519437

C -4.220727 1.852620 -3.386369

H -2.903740 0.212354 -2.903416

H -4.254967 2.583238 0.422844

H -5.519031 4.045138 -1.128640

H -4.197855 1.653468 -4.455036

H -5.504269 3.580011 -3.571859

C -3.680951 -1.242578 0.302836

C -5.331145 -3.407820 0.962989

C -3.111304 -2.494670 0.594538

C -5.076842 -1.088352 0.328435

C -5.897724 -2.166313 0.661603

C -3.944793 -3.567116 0.924461

H -2.025606 -2.624539 0.535672

H -5.523966 -0.129571 0.079585

H -6.978042 -2.038091 0.679949

H -3.500451 -4.535993 1.138341

H -5.971916 -4.250011 1.216433

C -2.169855 0.997360 1.513626

C -0.760774 1.191077 1.476850

C -2.922728 1.260400 2.654881

C -0.147950 1.756967 2.608429

C -2.287506 1.814346 3.774427

H -3.984204 1.022109 2.685850

C -0.910836 2.063990 3.738848

H 0.924889 1.930462 2.619382

H -2.861858 2.046596 4.668330

H -0.421596 2.485944 4.615206

P 2.170997 0.102197 -0.170756

C 3.180625 -1.045880 -1.216098

C 4.391381 -1.597827 -0.764401

C 2.736081 -1.341586 -2.514693

C 5.160991 -2.403107 -1.605159

H 4.730245 -1.407748 0.249885

C 3.509229 -2.153570 -3.348049

H 1.750509 -1.006665 -2.823854

C 4.722928 -2.677681 -2.902748

H 6.095864 -2.824409 -1.241479

H 3.141894 -2.395947 -4.341863

H 5.316651 -3.314828 -3.554957

C 3.021515 1.733205 -0.494416

C 4.411001 1.841550 -0.680278

C 2.249539 2.901102 -0.578879

C 5.007892 3.079916 -0.921394

H 5.030176 0.950477 -0.650529

C 2.845369 4.142145 -0.816382

H 1.169837 2.824345 -0.484351

C 4.226951 4.236361 -0.986349

H 6.084431 3.139295 -1.065802

H 2.224229 5.032549 -0.879678

H 4.691943 5.200527 -1.179544

C 2.723512 -0.352628 1.540010

C 2.173983 -1.538754 2.065389

C 3.599545 0.406038 2.328083

C 2.521606 -1.953600 3.350885

H 1.465107 -2.109323 1.461590

C 3.932382 -0.012151 3.621193

H 4.025735 1.326121 1.939776

C 3.397612 -1.194112 4.133176

H 2.094665 -2.871779 3.747409

H 4.610125 0.589059 4.223885

H 3.656618 -1.519871 5.138494

Rh -0.173880 0.011529 -0.253311

H -0.312325 1.588984 0.289522

C -0.088045 -2.485584 -1.291117

O -0.079458 -3.523059 -1.964566

O -0.076809 -2.410926 0.019482

O -0.119348 -1.274464 -1.893527

**INT2B**

P -2.516799 0.158212 0.011554

C -3.447380 1.266332 -1.120069

C -4.816724 2.879083 -2.964899

C -3.439259 0.945689 -2.488099

C -4.131422 2.411714 -0.690567

C -4.812885 3.214199 -1.610000

C -4.126245 1.744632 -3.401711

H -2.872904 0.082229 -2.829065

H -4.117722 2.685536 0.360626

H -5.337328 4.102906 -1.265868

H -4.111458 1.488670 -4.458356

H -5.346150 3.504707 -3.679960

C -3.708610 -1.190857 0.400407

C -5.439806 -3.283899 1.093440

C -3.186234 -2.440256 0.778159

C -5.099761 -1.004445 0.359768

C -5.960650 -2.045881 0.708690

C -4.057240 -3.476492 1.123305

H -2.105850 -2.594659 0.773189

H -5.512005 -0.048350 0.048743

H -7.037104 -1.891218 0.674882

H -3.646663 -4.443485 1.402905

H -6.111485 -4.097566 1.359266

C -2.058762 1.014387 1.548256

C -0.642722 0.938587 1.499517

C -2.777691 1.555017 2.616368

C 0.038482 1.485937 2.602360

C -2.063726 2.097608 3.688431

H -3.866575 1.543183 2.625222

C -0.664401 2.059473 3.669253

H 1.124280 1.457444 2.653973

H -2.589622 2.533937 4.535122

H -0.110074 2.473861 4.511004

P 2.137547 0.060396 -0.179059

C 3.147582 -0.964674 -1.346369

C 4.441583 -1.406634 -1.020445

C 2.611094 -1.287249 -2.603175

C 5.199204 -2.127342 -1.944527

H 4.857062 -1.193144 -0.039987

C 3.374581 -2.013164 -3.521379

H 1.573638 -1.044602 -2.815331

C 4.667921 -2.426545 -3.201403

H 6.199055 -2.463002 -1.677846

H 2.940651 -2.274565 -4.483145

H 5.254125 -2.996712 -3.919040

C 2.841927 1.765730 -0.425646

C 4.122486 1.993888 -0.954013

C 2.061102 2.874027 -0.059112

C 4.613469 3.293552 -1.102292

H 4.737689 1.154640 -1.261595

C 2.556261 4.171350 -0.199694

H 1.058851 2.710871 0.322106

C 3.833204 4.386446 -0.722411

H 5.605359 3.448610 -1.521048

H 1.935181 5.015505 0.090330

H 4.214173 5.398473 -0.840548

C 2.820399 -0.453003 1.465539

C 2.289207 -1.626641 2.031338

C 3.814982 0.255221 2.155503

C 2.767227 -2.080647 3.261288

H 1.494134 -2.157090 1.505119

C 4.282063 -0.203655 3.391027

H 4.225203 1.167889 1.733394

C 3.761163 -1.373982 3.944276

H 2.351157 -2.988271 3.692019

H 5.050743 0.357072 3.918646

H 4.123381 -1.730571 4.906240

Rh -0.200190 -0.097863 -0.262567

H -0.252535 1.318784 -0.975404

C -0.194936 -2.567524 -1.118383

O -0.220090 -3.720576 -1.549140

O -0.127163 -2.257860 0.182903

O -0.243093 -1.481097 -1.892999

**TS3B**

P -2.466658 0.352919 0.035102

C -3.667187 -0.011326 -1.312384

C -5.499674 -0.601738 -3.349735

C -3.902682 -1.349550 -1.677513

C -4.332805 1.021001 -1.989874

C -5.247188 0.726413 -3.003487

C -4.823859 -1.629681 -2.688469

H -3.357948 -2.167130 -1.201802

H -4.128995 2.056815 -1.738336

H -5.756911 1.536159 -3.521390

H -4.996781 -2.665430 -2.970222

H -6.210811 -0.833714 -4.140116

C -3.305244 -0.158759 1.592614

C -4.517059 -1.020725 3.968036

C -3.266850 -1.521021 1.945476

C -3.931928 0.763344 2.444028

C -4.535605 0.333471 3.627482

C -3.883742 -1.939054 3.127035

H -2.754171 -2.245719 1.303959

H -3.938206 1.819762 2.191659

H -5.017785 1.056903 4.281639

H -3.855483 -2.992866 3.394192

H -4.986449 -1.356575 4.890523

C -2.026569 2.119231 0.043633

C -0.615107 2.059092 -0.128873

C -2.745612 3.316766 0.139739

C 0.043297 3.297766 -0.253792

C -2.048094 4.522555 0.039931

H -3.825193 3.318681 0.279175

C -0.662802 4.501985 -0.166165

H 1.115523 3.336024 -0.418544

H -2.578418 5.470063 0.110115

H -0.125681 5.445550 -0.259330

P 2.227457 -0.091877 -0.040482

C 3.026134 -1.166645 -1.318271

C 4.427057 -1.232462 -1.428765

C 2.227183 -1.964885 -2.149779

C 5.024896 -2.066249 -2.371479

H 5.055250 -0.629791 -0.778362

C 2.839912 -2.801261 -3.091396

H 1.134066 -1.967857 -2.060616

C 4.227741 -2.853112 -3.209574

H 6.109387 -2.105362 -2.449128

H 2.210752 -3.418500 -3.727851

H 4.692750 -3.506982 -3.944787

C 3.153886 1.506383 -0.193728

C 3.252032 2.376266 0.906277

C 3.642056 1.948361 -1.433550

C 3.850931 3.629806 0.779132

H 2.854233 2.072709 1.870150

C 4.231081 3.208097 -1.564098

H 3.563939 1.303176 -2.302809

C 4.344904 4.051116 -0.457677

H 3.921094 4.281767 1.646558

H 4.601654 3.528179 -2.535043

H 4.806812 5.030284 -0.558941

C 2.884332 -0.811080 1.545995

C 4.131071 -0.465930 2.094230

C 2.099791 -1.783639 2.192557

C 4.581537 -1.065113 3.271800

H 4.753558 0.280402 1.610350

C 2.560463 -2.379298 3.370100

H 1.149522 -2.087028 1.750526

C 3.794500 -2.022240 3.915519

H 5.547740 -0.782368 3.684338

H 1.944367 -3.129746 3.859539

H 4.144202 -2.487922 4.834466

Rh -0.127110 0.034475 -0.027864

H -0.248801 -0.222414 -1.532604

C -0.972280 -2.585159 -0.682354

O -1.890610 -3.357077 -0.278975

O -0.141479 -2.051125 0.252989

O -0.801203 -2.231851 -1.902570

**INT3B**

P 2.486196 -0.214924 -0.041358

C 3.834095 0.839815 -0.747954

C 5.781403 2.482721 -1.932535

C 5.173035 0.420282 -0.825156

C 3.481817 2.098618 -1.260989

C 4.455465 2.910688 -1.851192

C 6.140042 1.234230 -1.415542

H 5.461577 -0.543285 -0.412958

H 2.452188 2.447803 -1.183239

H 4.171036 3.885483 -2.240676

H 7.172736 0.895682 -1.471485

H 6.535673 3.119330 -2.390756

C 3.337680 -1.029309 1.382223

C 4.518353 -2.220167 3.647612

C 3.939634 -0.241187 2.378835

C 3.330122 -2.422902 1.545820

C 3.916703 -3.012349 2.667993

C 4.526533 -0.830528 3.498121

H 3.948629 0.841001 2.275639

H 2.862367 -3.043661 0.787220

H 3.903383 -4.094992 2.774996

H 4.987103 -0.202869 4.257917

H 4.973277 -2.679746 4.522211

C 2.047505 -1.493169 -1.259234

C 0.633650 -1.345695 -1.329677

C 2.794145 -2.362113 -2.058798

C -0.006504 -2.162189 -2.290804

C 2.116847 -3.159755 -2.984451

H 3.878653 -2.411506 -1.971345

C 0.723759 -3.044092 -3.093114

H -1.080755 -2.106432 -2.435418

H 2.663310 -3.852785 -3.621367

H 0.197343 -3.653016 -3.828427

P -2.126027 -0.047607 0.020419

C -3.120376 1.339159 -0.729651

C -4.503313 1.496659 -0.536169

C -2.432356 2.291049 -1.496796

C -5.187683 2.563588 -1.118663

H -5.047169 0.785690 0.080021

C -3.118781 3.364124 -2.073878

H -1.353383 2.213042 -1.605691

C -4.495212 3.499441 -1.893421

H -6.259085 2.670124 -0.961175

H -2.565865 4.099139 -2.653374

H -5.027689 4.335753 -2.341805

C -2.946536 -1.566535 -0.670545

C -2.548299 -2.810707 -0.148372

C -3.872017 -1.555118 -1.723233

C -3.081888 -3.999627 -0.640987

H -1.800777 -2.840646 0.639728

C -4.396883 -2.749326 -2.229135

H -4.187387 -0.610834 -2.154892

C -4.010262 -3.974210 -1.686379

H -2.760464 -4.949171 -0.219432

H -5.110774 -2.716341 -3.049408

H -4.419632 -4.902329 -2.078879

C -2.819338 -0.063920 1.758453

C -3.938565 -0.824987 2.135191

C -2.199229 0.745165 2.727290

C -4.426762 -0.783219 3.443963

H -4.430820 -1.464364 1.409211

C -2.695863 0.788269 4.031672

H -1.332963 1.341048 2.449344

C -3.806687 0.024649 4.397671

H -5.291848 -1.385491 3.714735

H -2.203090 1.420911 4.766503

H -4.184477 0.056680 5.417618

Rh 0.151248 0.191259 -0.105497

C 0.286333 3.174315 0.592409

O 0.558428 3.466219 -0.587089

O 0.053730 2.049335 1.126912

O 0.218557 4.227382 1.506202

H 0.411079 5.003613 0.954977

**TS4A**

P -1.076739 0.939948 0.161471

C -0.264423 2.404782 -0.611983

C 0.896919 4.651443 -1.836790

C 0.019236 3.559983 0.136664

C 0.046599 2.385681 -1.982412

C 0.621158 3.504911 -2.585791

C 0.595101 4.675746 -0.474474

H -0.207738 3.589967 1.196737

H -0.141539 1.485846 -2.563034

H 0.860218 3.473566 -3.645734

H 0.810019 5.561354 0.118709

H 1.349845 5.519047 -2.311110

C -2.798542 0.816432 -0.384900

C -5.317416 0.239995 -1.337814

C -3.593825 1.864243 -0.872577

C -3.202108 -0.536528 -0.365228

C -4.495778 -0.784793 -0.861675

C -4.871717 1.568065 -1.343780

H -3.218975 2.885751 -0.899618

H -2.720276 -1.695697 0.371829

H -4.869626 -1.808199 -0.872300

H -5.512517 2.361572 -1.722643

H -6.313645 0.009463 -1.713883

C -1.051891 1.323506 1.957231

C 0.077902 1.010063 2.727679

C -2.148491 1.951727 2.563089

C 0.115014 1.347279 4.079827

H 0.909205 0.481089 2.275049

C -2.107739 2.283291 3.919376

H -3.037032 2.169799 1.977926

C -0.975032 1.985014 4.677750

H 0.989527 1.091016 4.672017

H -2.965429 2.765472 4.382079

H -0.945990 2.236053 5.735475

C -1.587863 -2.549456 1.891080

O -0.633832 -1.643316 1.603172

O -2.560819 -2.683744 1.007641

Rh -0.507263 -1.151915 -0.370622

O -1.493849 -3.213566 2.922214

Br -0.392251 -1.040723 -2.918350

C 1.474932 -1.148935 -0.190730

C 1.923656 -2.473988 -0.122548

C 2.408304 -0.121249 -0.107624

C 3.270544 -2.764594 0.058568

C 3.786063 -0.379086 0.070425

H 2.072094 0.908099 -0.188907

C 4.192926 -1.714575 0.160248

H 3.611109 -3.795890 0.105943

H 5.239608 -1.964307 0.299037

O 0.944469 -3.434782 -0.322473

C 0.822737 -4.448649 0.683597

H -0.023962 -5.066968 0.379855

H 1.727674 -5.069438 0.726707

H 0.611531 -4.004002 1.660214

C 4.771571 0.802909 0.148823

C 4.724905 1.603495 -1.175055

C 4.381295 1.735048 1.320653

C 6.224043 0.341193 0.375715

H 5.007593 0.967444 -2.021932

H 3.722936 1.995367 -1.376403

H 5.419511 2.453997 -1.137800

H 4.412773 1.194657 2.274231

H 5.074771 2.584553 1.386582

H 3.371104 2.138526 1.199129

H 6.887490 1.213288 0.431266

H 6.329999 -0.218094 1.312670

H 6.580214 -0.295510 -0.442394

**INT4A**

P -1.059077 1.167227 0.292931

C -2.700760 1.869322 -0.154368

C -5.236590 2.922886 -0.717210

C -2.846024 3.222467 -0.501155

C -3.835199 1.041771 -0.097149

C -5.093797 1.575702 -0.377961

C -4.110277 3.745252 -0.778637

H -1.977565 3.870533 -0.558363

H -3.712506 -0.011664 0.150854

H -5.965068 0.927078 -0.336804

H -4.210883 4.794325 -1.047465

H -6.220825 3.330256 -0.937839

C -0.943803 0.857891 2.076802

C -0.636556 -0.514928 4.436497

C -1.175799 1.544287 3.271190

C -0.593067 -0.506286 2.026429

C -0.433802 -1.197105 3.231856

C -1.005837 0.838631 4.464827

H -1.488534 2.586636 3.279784

H -0.168826 -2.251014 3.236547

H -1.168954 1.335331 5.418586

H -0.517644 -1.048788 5.378154

C 0.133207 2.429972 -0.286671

C 0.504112 2.418347 -1.641596

C 0.669480 3.398883 0.572460

C 1.382566 3.386963 -2.128879

H 0.131930 1.630144 -2.293404

C 1.553678 4.361943 0.079193

H 0.407278 3.391975 1.626640

C 1.905076 4.360949 -1.272520

H 1.669668 3.371428 -3.177030

H 1.968788 5.108985 0.751338

H 2.593838 5.110170 -1.655482

Br -0.468194 -1.247679 -2.631360

C 1.514171 -0.904522 0.128752

C 1.819997 -2.265867 0.242729

C 2.585290 -0.018474 0.084542

C 3.111194 -2.761639 0.279511

C 3.927985 -0.464869 0.131736

H 2.393085 1.045300 -0.006784

C 4.168590 -1.839944 0.222512

H 3.310054 -3.827521 0.356675

H 5.183218 -2.222526 0.254543

O 0.654082 -3.042840 0.349053

C 0.556586 -4.199097 -0.499812

H -0.493141 -4.492359 -0.489163

H 0.852550 -3.939306 -1.520612

H 1.189095 -5.004797 -0.106905

C 5.069052 0.570229 0.068922

C 5.006979 1.327755 -1.279025

C 4.921283 1.583210 1.229296

C 6.462392 -0.078204 0.184777

H 5.122278 0.632778 -2.118987

H 4.051854 1.846102 -1.406563

H 5.809782 2.075849 -1.340189

H 4.967326 1.073334 2.198893

H 5.728235 2.328156 1.195700

H 3.968288 2.118716 1.179508

H 7.237408 0.697787 0.149600

H 6.579101 -0.623083 1.128974

H 6.656095 -0.776916 -0.637338

Rh -0.514791 -1.007747 0.065018

O -2.612071 -1.647454 0.262585

C -3.097843 -2.813887 -0.044866

O -3.984828 -3.408570 0.553390

O -2.552249 -3.420621 -1.162608

H -1.990523 -2.761880 -1.627539

**INT3A-D**

P -1.218898 0.607604 0.105879

C -0.303364 2.092758 -0.521422

C 1.023536 4.350335 -1.546178

C -0.104787 3.229042 0.278856

C 0.177182 2.097158 -1.841958

C 0.830439 3.221755 -2.347317

C 0.556996 4.349481 -0.231154

H -0.464093 3.244397 1.301989

D 0.053105 1.209545 -2.457847

H 1.197496 3.208662 -3.370483

H 0.705253 5.219931 0.403544

H 1.538797 5.222179 -1.942728

C -2.885197 0.788543 -0.671582

C -5.455902 1.041354 -1.763891

C -3.320237 2.019897 -1.187750

C -3.747353 -0.319777 -0.707425

C -5.026225 -0.183905 -1.250860

C -4.600261 2.143871 -1.731062

D -2.662075 2.882633 -1.173798

H -3.404006 -1.274870 -0.312521

H -5.684470 -1.048576 -1.278688

H -4.923622 3.101498 -2.132730

H -6.451738 1.137106 -2.191311

C -1.492207 1.013892 1.884593

C -0.437681 0.804211 2.788893

C -2.700031 1.547336 2.354188

C -0.587649 1.146459 4.131723

D 0.487586 0.359685 2.440799

C -2.849491 1.877385 3.703638

H -3.528149 1.701470 1.670446

C -1.793276 1.680979 4.593026

H 0.233306 0.973858 4.822725

H -3.794851 2.282618 4.056346

H -1.911092 1.933233 5.644382

C -1.585681 -2.595760 1.668798

O -0.412623 -1.926218 1.798476

O -2.034483 -2.518350 0.429095

Rh -0.264384 -1.394231 -0.164535

O -2.107247 -3.188418 2.604960

Br -0.449657 -1.302834 -2.741220

C 1.716169 -0.904071 -0.143121

C 2.301030 -2.176859 -0.137911

C 2.575125 0.186493 -0.030239

C 3.665250 -2.385564 -0.020975

C 3.976308 0.032294 0.096193

H 2.161939 1.190172 -0.035452

C 4.500572 -1.264711 0.100064

H 4.086902 -3.387763 -0.029662

H 5.569007 -1.429499 0.192032

O 1.357253 -3.202071 -0.307918

C 1.350009 -4.240470 0.680419

H 0.530042 -4.906963 0.406709

H 2.295691 -4.795949 0.660978

H 1.164088 -3.815866 1.670904

C 4.867244 1.284809 0.216611

C 4.740605 2.134927 -1.070209

C 4.420900 2.133343 1.431471

C 6.355558 0.934156 0.409337

H 5.073090 1.563798 -1.945152

H 3.706189 2.443262 -1.249284

H 5.357580 3.041712 -0.998531

H 4.502885 1.555705 2.359871

H 5.050881 3.028211 1.528757

H 3.382471 2.464887 1.335186

H 6.946052 1.854376 0.502260

H 6.518350 0.340410 1.316498

H 6.753449 0.369961 -0.442063

**TS4A-D**

P -1.076739 0.939948 0.161471

C -0.264423 2.404782 -0.611983

C 0.896919 4.651443 -1.836790

C 0.019236 3.559983 0.136664

C 0.046599 2.385681 -1.982412

C 0.621158 3.504911 -2.585791

C 0.595101 4.675746 -0.474474

H -0.207738 3.589967 1.196737

D -0.141539 1.485846 -2.563034

H 0.860218 3.473566 -3.645734

H 0.810019 5.561354 0.118709

H 1.349845 5.519047 -2.311110

C -2.798542 0.816432 -0.384900

C -5.317416 0.239995 -1.337814

C -3.593825 1.864243 -0.872577

C -3.202108 -0.536528 -0.365228

C -4.495778 -0.784793 -0.861675

C -4.871717 1.568065 -1.343780

H -3.218975 2.885751 -0.899618

D -2.720276 -1.695697 0.371829

H -4.869626 -1.808199 -0.872300

H -5.512517 2.361572 -1.722643

H -6.313645 0.009463 -1.713883

C -1.051891 1.323506 1.957231

C 0.077902 1.010063 2.727679

C -2.148491 1.951727 2.563089

C 0.115014 1.347279 4.079827

H 0.909205 0.481089 2.275049

C -2.107739 2.283291 3.919376

D -3.037032 2.169799 1.977926

C -0.975032 1.985014 4.677750

H 0.989527 1.091016 4.672017

H -2.965429 2.765472 4.382079

H -0.945990 2.236053 5.735475

C -1.587863 -2.549456 1.891080

O -0.633832 -1.643316 1.603172

O -2.560819 -2.683744 1.007641

Rh -0.507263 -1.151915 -0.370622

O -1.493849 -3.213566 2.922214

Br -0.392251 -1.040723 -2.918350

C 1.474932 -1.148935 -0.190730

C 1.923656 -2.473988 -0.122548

C 2.408304 -0.121249 -0.107624

C 3.270544 -2.764594 0.058568

C 3.786063 -0.379086 0.070425

H 2.072094 0.908099 -0.188907

C 4.192926 -1.714575 0.160248

H 3.611109 -3.795890 0.105943

H 5.239608 -1.964307 0.299037

O 0.944469 -3.434782 -0.322473

C 0.822737 -4.448649 0.683597

H -0.023962 -5.066968 0.379855

H 1.727674 -5.069438 0.726707

H 0.611531 -4.004002 1.660214

C 4.771571 0.802909 0.148823

C 4.724905 1.603495 -1.175055

C 4.381295 1.735048 1.320653

C 6.224043 0.341193 0.375715

H 5.007593 0.967444 -2.021932

H 3.722936 1.995367 -1.376403

H 5.419511 2.453997 -1.137800

H 4.412773 1.194657 2.274231

H 5.074771 2.584553 1.386582

H 3.371104 2.138526 1.199129

H 6.887490 1.213288 0.431266

H 6.329999 -0.218094 1.312670

H 6.580214 -0.295510 -0.442394

K_2_CO_3_

K 2.483194 -0.605887 -0.000028

O 1.128093 1.412817 0.000200

C 0.000128 0.801775 0.000032

O -1.127375 1.413581 -0.000215

K -2.483429 -0.605766 0.000052

O -0.000256 -0.550054 -0.000066

**INT5A**

P -2.437175 -0.497724 0.199748

C -3.558006 -1.369758 -0.965715

C -5.213593 -2.721103 -2.778661

C -4.847329 -1.776484 -0.582863

C -3.104581 -1.643217 -2.265028

C -3.936246 -2.315340 -3.165981

C -5.669221 -2.451024 -1.484403

H -5.210332 -1.564857 0.419325

H -2.107175 -1.327505 -2.566378

H -3.577523 -2.521609 -4.170990

H -6.663572 -2.766666 -1.178204

H -5.855479 -3.247429 -3.481077

C -2.260936 -1.417080 1.756026

C -1.141855 -2.861920 3.812490

C -3.101512 -1.873039 2.777880

C -0.873989 -1.659337 1.740093

C -0.310819 -2.393297 2.792037

C -2.522953 -2.604499 3.815397

H -4.168808 -1.661798 2.775935

H 0.755548 -2.588846 2.811388

H -3.140733 -2.974825 4.630311

H -0.711104 -3.436864 4.630640

C -3.282079 1.099155 0.541636

C -4.106812 1.707042 -0.417134

C -3.063539 1.749189 1.766616

C -4.713942 2.935536 -0.149207

H -4.281205 1.218484 -1.371095

C -3.674277 2.974828 2.031643

H -2.414857 1.294897 2.508854

C -4.501770 3.570526 1.076043

H -5.352815 3.394378 -0.899495

H -3.500212 3.465917 2.985489

H -4.976140 4.526094 1.284789

C 0.408668 0.841877 0.731174

C 1.373853 0.962886 1.754692

C 0.007501 2.003047 0.052433

C 1.929983 2.227417 2.017476

C 0.550631 3.275261 0.304840

H -0.709554 1.889979 -0.748537

C 1.529526 3.355466 1.305863

H 2.708336 2.324586 2.765584

H 2.002888 4.305766 1.539988

O 1.740490 -0.145006 2.452464

C 3.027234 -0.148125 3.078831

H 3.169485 -1.176567 3.426017

H 3.810275 0.116519 2.353067

H 3.046918 0.513299 3.958066

C 0.127974 4.536400 -0.473291

C 1.362331 5.161140 -1.168162

C -0.924161 4.235824 -1.558437

C -0.473895 5.567561 0.511151

H 2.150812 5.415694 -0.452343

H 1.789954 4.467879 -1.902989

H 1.082098 6.080131 -1.700287

H -1.849290 3.836654 -1.129056

H -1.176614 5.161218 -2.091686

H -0.553370 3.515209 -2.295924

H -0.775142 6.480449 -0.020255

H -1.358324 5.154150 1.009355

H 0.245442 5.852890 1.286865

Rh -0.154648 -0.951046 -0.008080

C 1.590020 -3.097801 -0.412691

O 2.447497 -3.946379 -0.669030

Br 0.432751 -0.267880 -2.587818

K 3.037226 1.291167 -1.146616

O 5.595288 1.230669 -1.284382

C 5.610014 0.224387 -0.482875

O 6.446108 -0.751556 -0.583696

O 4.646445 0.138428 0.446262

K 4.615986 -2.360361 0.142647

O 1.732959 -1.962252 0.152312

O 0.256769 -3.346057 -0.738124

H 0.238179 -4.109713 -1.338659

**TS6A**

P 1.140869 1.792917 0.298460

C 0.391702 3.388913 -0.243260

C -0.706311 5.839402 -1.049788

C 1.185346 4.549136 -0.275508

C -0.957071 3.463084 -0.621037

C -1.494664 4.688585 -1.024184

C 0.636387 5.768663 -0.670032

H 2.236744 4.501508 -0.006696

H -1.575798 2.573057 -0.557044

H -2.540435 4.736637 -1.317682

H 1.259704 6.659620 -0.689003

H -1.133039 6.788974 -1.365179

C 1.227881 1.629691 2.103336

C 0.840732 0.569290 4.609916

C 1.657343 2.373684 3.205162

C 0.581028 0.383519 2.223733

C 0.392906 -0.155783 3.499977

C 1.462749 1.820125 4.473069

H 2.117785 3.352827 3.089513

H -0.108784 -1.110537 3.628343

H 1.787316 2.362195 5.358316

H 0.692679 0.160478 5.608096

C 2.802583 1.882417 -0.468225

C 2.905476 1.644907 -1.848970

C 3.947665 2.222772 0.264045

C 4.138212 1.777942 -2.487434

H 2.026730 1.330964 -2.407707

C 5.179931 2.355674 -0.382109

H 3.877512 2.373655 1.337604

C 5.274641 2.141401 -1.758118

H 4.212672 1.589224 -3.555234

H 6.064168 2.621776 0.191820

H 6.233806 2.243794 -2.259724

C 1.562425 -1.492744 0.265195

C 0.895043 -2.683599 0.571564

C 2.924082 -1.569434 -0.022037

C 1.516295 -3.924857 0.558682

C 3.610377 -2.804834 -0.026841

H 3.464872 -0.664487 -0.267354

C 2.882301 -3.967674 0.258964

H 0.969011 -4.837354 0.776005

H 3.377571 -4.935693 0.252053

O -0.437407 -2.446800 0.894650

C -1.407819 -3.433997 0.501016

H -2.398835 -2.992647 0.606742

H -1.234460 -3.726864 -0.540547

H -1.320825 -4.312302 1.152565

C 5.115568 -2.920287 -0.345388

C 5.313642 -3.795803 -1.606037

C 5.770863 -1.552921 -0.612881

C 5.852840 -3.573156 0.848471

H 4.908784 -4.803830 -1.466631

H 4.804632 -3.351021 -2.468796

H 6.380915 -3.892425 -1.849397

H 5.683274 -0.883999 0.250135

H 6.839465 -1.689030 -0.824075

H 5.322453 -1.047074 -1.474039

H 6.926500 -3.669910 0.635960

H 5.737276 -2.965835 1.753825

H 5.464112 -4.573102 1.068794

Rh 0.024208 -0.170467 0.367392

O -1.866890 0.966620 0.650193

C -3.000835 0.385884 0.677995

O -4.026105 0.803490 0.076623

O -3.053062 -0.753279 1.407415

H -3.865540 -1.283165 1.189543

Br -0.557671 -0.509275 -2.299948

K -3.704321 -1.137992 -2.040700

O -6.154553 -1.509146 -1.129446

C -5.592655 -2.010131 -0.049670

O -6.079120 -1.744251 1.118630

O -4.469041 -2.655148 -0.155620

K -6.593402 0.546169 0.320766

**INT6A**

P -0.624920 1.700343 0.722292

C 1.034688 2.422910 1.100513

C 3.594451 3.509326 1.531585

C 1.347782 3.046029 2.319202

C 2.025244 2.341437 0.107544

C 3.296593 2.884614 0.315396

C 2.619090 3.585693 2.532439

H 0.601037 3.100272 3.106233

H 1.818434 1.818507 -0.822937

H 4.055478 2.769224 -0.453582

H 2.847407 4.067980 3.480225

H 4.583216 3.930024 1.699129

C -1.651531 2.953859 -0.100253

C -3.019529 4.331284 -2.046091

C -2.072699 4.258435 0.183858

C -1.880376 2.309315 -1.339061

C -2.585278 3.031829 -2.317616

C -2.775249 4.945518 -0.806432

H -1.855408 4.730036 1.140481

H -2.781021 2.582455 -3.286656

H -3.124962 5.959352 -0.624451

H -3.556718 4.888292 -2.812770

C -1.285875 1.228722 2.365737

C -0.647874 0.198059 3.079415

C -2.429664 1.834152 2.904370

C -1.139770 -0.196840 4.323892

H 0.214017 -0.309980 2.650997

C -2.919163 1.430480 4.149210

H -2.940604 2.613728 2.347037

C -2.273495 0.419394 4.862371

H -0.642975 -0.997848 4.865167

H -3.807441 1.905975 4.557997

H -2.656936 0.104545 5.829858

C -2.275460 -0.656542 -0.806886

C -3.532817 -0.671217 -1.439908

C -1.968729 -1.672883 0.102519

C -4.449203 -1.679565 -1.112503

C -2.865941 -2.707567 0.426044

H -0.984645 -1.673157 0.552409

C -4.117739 -2.676679 -0.195830

H -5.426881 -1.702900 -1.582567

H -4.857208 -3.444522 0.018888

O -3.807846 0.304723 -2.360580

C -5.011087 0.222062 -3.088029

H -4.994891 1.058477 -3.791196

H -5.085435 -0.719633 -3.650575

H -5.896215 0.319008 -2.440690

C -2.513004 -3.847801 1.400672

C -2.632645 -5.205742 0.667545

C -1.081037 -3.736877 1.957034

C -3.496753 -3.825645 2.595416

H -3.643807 -5.374242 0.279741

H -1.937258 -5.249294 -0.178182

H -2.391994 -6.032332 1.349379

H -0.938900 -2.809975 2.522839

H -0.888055 -4.572965 2.640939

H -0.318709 -3.773399 1.171989

H -3.270615 -4.641083 3.295692

H -3.421408 -2.877461 3.140557

H -4.536831 -3.942191 2.269682

Rh -0.788684 0.581070 -1.337332

O -0.650010 -0.218976 -3.252050

C 0.343963 -1.031039 -2.942471

O 0.936285 -1.774654 -3.737531

Br 5.328611 -0.192118 -1.174517

K 3.916853 -0.039312 1.592970

O 3.082493 -2.438865 2.232584

C 2.400530 -2.517752 1.188539

O 2.304039 -3.409291 0.319385

O 1.655729 -1.316874 0.932836

K 2.983429 -2.280643 -2.028095

O 0.687042 -0.938874 -1.649979

H 1.210768 -1.396204 0.058777

**TS7A**

P 1.767560 1.438454 0.041176

C 1.328797 3.225355 0.111277

C 0.502259 5.903288 0.220016

C 2.266839 4.261206 -0.017209

C -0.026568 3.545858 0.295595

C -0.437131 4.877556 0.352663

C 1.851910 5.593778 0.037617

H 3.318537 4.034097 -0.162001

H -0.773951 2.766169 0.404801

H -1.494185 5.088557 0.484970

H 2.586790 6.389252 -0.065802

H 0.183569 6.942721 0.255440

C 1.433784 0.726013 1.682092

C 0.131189 -0.407036 3.844874

C 1.631846 1.254810 2.958245

C 0.551218 -0.373218 1.435971

C -0.102421 -0.917249 2.566545

C 0.998734 0.670388 4.057070

H 2.243296 2.145495 3.087538

H -0.766501 -1.766797 2.450451

H 1.156439 1.062349 5.058272

H -0.381185 -0.859120 4.692122

C 3.576187 1.451744 -0.315865

C 3.984285 1.550805 -1.656327

C 4.553181 1.364097 0.687077

C 5.340553 1.581642 -1.983120

H 3.234081 1.597976 -2.442033

C 5.910977 1.385046 0.357208

H 4.251517 1.270125 1.726412

C 6.306940 1.498894 -0.976790

H 5.642569 1.660077 -3.024403

H 6.657950 1.312595 1.143962

H 7.363461 1.514815 -1.232579

C 0.990550 -1.731544 0.206979

C 0.157278 -2.892401 0.218788

C 2.375085 -1.953454 0.084175

C 0.718612 -4.152716 0.016576

H 3.027003 -1.089783 0.131451

C 2.097278 -4.314531 -0.148168

H 0.081753 -5.029670 -0.009716

H 2.479582 -5.319249 -0.293042

O -1.177704 -2.719166 0.447817

C -2.070004 -3.784336 0.122145

H -3.080942 -3.385001 0.233762

H -1.912659 -4.111743 -0.912310

H -1.934807 -4.637709 0.801526

Rh 0.133147 -0.020036 -0.663140

O -0.559896 0.880841 -2.346391

C -1.676409 0.172586 -2.422203

O -1.812006 -0.668343 -1.403682

C 2.959343 -3.216603 -0.105740

O -2.509469 0.288065 -3.334188

Br -5.471991 -2.084358 0.531192

K -2.918947 -0.147610 1.074543

O -3.022203 2.503313 1.320687

C -3.728717 2.694473 0.310656

O -4.291788 1.839689 -0.434815

O -3.915739 4.027021 -0.050474

K -4.645424 -0.228614 -1.907443

H -4.434162 3.980324 -0.870465

C 4.486383 -3.343910 -0.261638

C 4.951115 -2.549682 -1.505206

C 5.189268 -2.783016 0.997131

C 4.931468 -4.808626 -0.439545

H 4.474898 -2.939306 -2.412671

H 4.699063 -1.487983 -1.427582

H 6.039733 -2.628156 -1.629077

H 4.880678 -3.334446 1.893261

H 6.279915 -2.869612 0.900466

H 4.952937 -1.726271 1.155634

H 6.022376 -4.854634 -0.544301

H 4.654165 -5.425896 0.423137

H 4.493259 -5.260210 -1.337128

**INT7A**

P 0.900350 -1.424558 -0.433134

C 0.891149 -1.313850 1.438762

C 0.652010 -1.029764 4.241111

C 0.154871 -2.233135 2.208293

C 1.537070 -0.241921 2.102389

C 1.400569 -0.121606 3.493514

C 0.032398 -2.100126 3.594523

H -0.332765 -3.068304 1.715671

H 1.889718 0.712035 3.989544

H -0.531549 -2.838474 4.162293

H 0.559818 -0.904150 5.317191

C -0.352364 -2.759978 -0.725388

C -2.377654 -4.675280 -1.102860

C -0.036398 -4.125293 -0.806112

C -1.695564 -2.367549 -0.856353

C -2.704516 -3.318359 -1.025728

C -1.043080 -5.074702 -1.003102

H 0.995212 -4.452399 -0.716661

H -1.950641 -1.312021 -0.853296

H -3.736045 -2.979932 -1.077211

H -0.780870 -6.128337 -1.073849

H -3.160183 -5.416933 -1.245964

C 2.502882 -2.287675 -0.804855

C 2.988662 -2.182543 -2.119632

C 3.238241 -3.036360 0.125917

C 4.175228 -2.814137 -2.496320

H 2.417447 -1.606655 -2.846802

C 4.421652 -3.675248 -0.252980

H 2.889422 -3.113598 1.150805

C 4.893629 -3.565201 -1.563244

H 4.535408 -2.721365 -3.518008

H 4.976407 -4.259365 0.478067

H 5.816097 -4.062040 -1.854923

C 3.746265 0.645028 1.262372

C 1.756272 1.993055 0.967340

C 4.495967 1.652204 0.645363

C 2.494145 3.039394 0.389357

H 0.686682 2.086105 1.112588

C 3.868452 2.824438 0.220659

H 5.566278 1.543549 0.508498

H 4.482728 3.590677 -0.245058

O 4.286184 -0.509315 1.775878

C 5.670060 -0.732197 1.598000

H 5.882333 -1.697122 2.063934

H 5.940727 -0.782261 0.535039

H 6.273756 0.045274 2.088031

C 2.356450 0.801945 1.403072

C 1.855760 4.374636 -0.040912

C 2.605701 5.541045 0.648770

C 1.962410 4.533741 -1.576955

C 0.370934 4.469735 0.357244

H 2.547161 5.455423 1.740834

H 3.665694 5.575378 0.371420

H 2.156960 6.499860 0.360020

H 1.387782 3.758248 -2.094601

H 1.563813 5.510686 -1.881356

H 3.004811 4.479622 -1.915555

H -0.026640 5.445787 0.054853

H -0.246707 3.709298 -0.126263

H 0.231876 4.378173 1.440825

Rh 0.623388 0.417708 -1.699742

O -0.014074 1.938968 -2.991258

C -1.263860 1.752677 -2.675902

O -1.396359 0.797894 -1.718574

O -2.253843 2.333899 -3.137464

Br -5.311564 -0.887723 0.323882

K -2.949348 -0.560175 2.395115

O -2.880631 2.050285 2.724443

C -2.560054 2.230102 1.530807

O -2.953257 3.053437 0.676460

O -1.615221 1.256069 1.069113

K -4.024659 1.625745 -1.255434

H -1.457711 1.360487 0.103846

**INT8A**

P -0.208094 -1.406696 1.114281

C 0.769501 -0.537493 2.437481

C 2.175280 0.914779 4.399224

C 1.705703 -1.172497 3.265293

C 0.553324 0.843375 2.598903

C 1.238467 1.556778 3.583091

C 2.411636 -0.449789 4.232529

H 1.880516 -2.238899 3.163884

H -0.162307 1.356645 1.960120

H 1.043255 2.618476 3.706264

H 3.138037 -0.959787 4.861309

H 2.715164 1.475542 5.158769

C -1.901534 -1.450043 1.902438

C -4.493383 -1.679133 2.993529

C -2.152656 -1.088417 3.236641

C -2.974422 -1.907919 1.115215

C -4.257954 -2.022634 1.656701

C -3.438898 -1.202744 3.778943

H -1.342938 -0.731836 3.864447

H -2.803451 -2.145209 0.069244

H -5.083546 -2.334761 1.025524

H -3.608803 -0.933832 4.820004

H -5.493875 -1.770531 3.409754

C 0.294544 -3.208151 1.381626

C 1.336971 -3.840298 0.660531

C -0.393949 -3.971696 2.344004

C 1.625865 -5.191631 0.921926

C -0.094278 -5.310208 2.589711

H -1.188419 -3.512463 2.919357

C 0.922314 -5.930654 1.868360

H 2.424461 -5.661629 0.354291

H -0.658177 -5.859527 3.339875

H 1.168822 -6.975958 2.038963

Rh -0.141797 -0.615437 -1.000081

Br 2.157642 0.640956 -0.784787

C 1.895412 2.555091 -0.770044

C 2.513541 3.316779 0.209180

C 1.115634 3.137475 -1.787531

C 2.373151 4.717975 0.236780

H 3.103065 2.805813 0.963300

C 0.938621 4.525591 -1.738082

C 1.560879 5.290203 -0.748474

H 0.314744 5.011763 -2.479567

H 1.397188 6.362156 -0.765286

O 0.630145 2.319260 -2.728998

C -0.392711 2.781664 -3.615662

H -0.694267 1.888385 -4.161553

H -1.242509 3.166996 -3.044404

H 0.005529 3.540500 -4.304498

C 2.226754 -3.190941 -0.356121

C 1.916195 -3.257208 -1.729363

C 3.466665 -2.673212 0.035917

C 2.840385 -2.766774 -2.660484

C 4.415558 -2.192299 -0.879448

H 3.681314 -2.656772 1.098791

C 4.062912 -2.250374 -2.234629

H 2.608454 -2.779612 -3.718895

H 4.752307 -1.885967 -2.992002

O 0.740875 -3.850090 -2.059449

C 0.255897 -3.683062 -3.390888

H -0.757850 -4.085756 -3.386903

H 0.225064 -2.622602 -3.659679

H 0.869894 -4.247421 -4.107890

C 5.796356 -1.658425 -0.453872

C 6.018503 -0.237739 -1.023178

C 5.946978 -1.584596 1.078089

C 6.895952 -2.599992 -1.002023

H 5.982729 -0.226890 -2.117562

H 5.248218 0.450726 -0.662054

H 7.000455 0.147823 -0.718492

H 5.871397 -2.574121 1.543989

H 6.931898 -1.174890 1.333554

H 5.187159 -0.936849 1.529466

H 7.893621 -2.236873 -0.720943

H 6.777150 -3.614399 -0.602959

H 6.858550 -2.666086 -2.094999

C 3.104857 5.542824 1.311563

C 2.646399 5.101785 2.720931

C 4.630937 5.319902 1.182853

C 2.830397 7.052634 1.172275

H 1.567581 5.253173 2.843237

H 2.857887 4.043255 2.901616

H 3.163966 5.684111 3.494770

H 4.990308 5.637085 0.196952

H 5.171138 5.896177 1.945634

H 4.897994 4.265358 1.309908

H 3.373486 7.600310 1.951908

H 3.163274 7.439173 0.202030

H 1.765163 7.284322 1.284548

O -0.445936 -0.379871 -3.086699

C -1.670916 -0.820574 -2.987735

O -1.963057 -1.242006 -1.746349

O -2.525361 -0.815954 -3.891744

Br -6.636866 0.173843 -0.198357

K -4.451285 1.327553 1.736681

O -3.586275 3.448889 0.444314

C -2.911934 2.729059 -0.319057

O -2.876516 2.656669 -1.570574

O -2.127526 1.768393 0.386835

K -4.217670 0.444506 -2.267689

H -1.596918 1.226117 -0.246736

**INT9A**

P 1.495034 -0.750060 1.030891

C 0.602595 0.300389 2.291133

C -0.881843 1.767101 4.192319

C 1.217910 1.277349 3.087726

C -0.772900 0.071816 2.466106

C -1.505311 0.789360 3.412271

C 0.482189 2.009771 4.025217

H 2.282458 1.462754 2.983958

H -1.265771 -0.679368 1.855046

H -2.564631 0.581834 3.542817

H 0.980824 2.763579 4.630856

H -1.453824 2.331608 4.925285

C 1.734644 -2.307554 2.042943

C 2.059429 -4.734446 3.437468

C 1.598529 -2.357999 3.440109

C 2.036549 -3.494836 1.350551

C 2.203795 -4.692867 2.048279

C 1.754622 -3.562754 4.131559

H 1.366419 -1.455710 3.997546

H 2.131643 -3.467267 0.266091

H 2.439180 -5.600498 1.497050

H 1.637346 -3.581793 5.213382

H 2.180456 -5.672990 3.974766

C 3.219486 0.001417 1.084972

C 3.562672 1.138181 0.310969

C 4.218422 -0.580847 1.884007

C 4.870852 1.642353 0.380291

C 5.516117 -0.071592 1.935076

H 3.979376 -1.455902 2.477136

C 5.846751 1.050602 1.179765

H 5.118375 2.513456 -0.221021

H 6.261148 -0.555573 2.562719

H 6.853835 1.460896 1.205323

Rh 0.654822 -1.196761 -1.004872

C 1.536012 -2.780162 -2.752549

O 2.105912 -2.518928 -1.564045

O 0.450691 -2.040314 -2.927769

Br -1.320791 0.498421 -1.062168

O 1.989352 -3.589407 -3.557582

C -3.061177 -0.334692 -0.887465

C -3.979412 0.154283 0.028242

C -3.363674 -1.432921 -1.716604

C -5.247697 -0.441249 0.184617

H -3.691455 1.006476 0.636007

C -4.607912 -2.049104 -1.545583

C -5.526415 -1.555474 -0.613522

H -4.869087 -2.907447 -2.156323

H -6.481086 -2.063512 -0.528050

O -2.435981 -1.783743 -2.617873

C -2.426733 -3.093837 -3.174820

H -1.423750 -3.195836 -3.590655

H -2.586541 -3.847953 -2.393932

H -3.199514 -3.198901 -3.950062

C 2.613068 1.875526 -0.585156

C 2.556392 1.566654 -1.960752

C 1.857216 2.945651 -0.097443

C 1.707946 2.310769 -2.790625

C 1.008645 3.712000 -0.911931

H 1.935736 3.166588 0.960740

C 0.951612 3.358469 -2.266910

H 1.628954 2.068868 -3.844369

H 0.304284 3.908774 -2.945889

O 3.376959 0.582229 -2.395530

C 3.103053 -0.024352 -3.658264

H 3.730578 -0.914510 -3.701450

H 2.054968 -0.333212 -3.721281

H 3.361355 0.655180 -4.484448

C 0.172197 4.889737 -0.378097

C -1.330198 4.615400 -0.624875

C 0.374408 5.114298 1.132862

C 0.578667 6.189776 -1.112753

H -1.550492 4.505010 -1.692075

H -1.640835 3.690231 -0.128159

H -1.943365 5.441121 -0.238668

H 1.419369 5.346244 1.370811

H -0.237340 5.962168 1.465870

H 0.078738 4.237659 1.719177

H -0.016946 7.040720 -0.755196

H 1.637288 6.419255 -0.942039

H 0.426987 6.106762 -2.194414

C -6.252054 0.145273 1.193324

C -5.638225 0.141380 2.612909

C -6.594398 1.600339 0.792585

C -7.565612 -0.659043 1.241162

H -5.382575 -0.877515 2.925657

H -4.725137 0.743483 2.658878

H -6.347402 0.555892 3.341342

H -7.049671 1.632900 -0.204127

H -7.301995 2.042340 1.506537

H -5.701582 2.234028 0.770047

H -8.249692 -0.208871 1.970561

H -8.074077 -0.665646 0.270158

H -7.394142 -1.698542 1.543828

KBr-KHCO_3_

Br 2.072350 0.008534 -0.253282

K -0.111500 2.078131 0.373131

O -1.586933 0.045940 1.031768

C -2.169458 -0.114392 -0.073392

O -2.403708 -1.163884 -0.702961

K -0.128018 -2.094646 0.355425

O -2.515282 1.100412 -0.702347

H -2.917291 0.841709 -1.549051

**TS10A**

P -0.254508 1.067254 -0.194855

C 0.651911 2.658240 -0.633559

C 2.025059 4.965023 -1.527447

C 0.481379 3.896947 0.007012

C 1.518305 2.606482 -1.739999

C 2.196202 3.743852 -2.182885

C 1.163574 5.035387 -0.432205

H -0.184519 3.978668 0.859303

H 1.664689 1.663536 -2.257535

H 2.859776 3.672038 -3.041612

H 1.015778 5.980567 0.085938

H 2.556810 5.850977 -1.866665

C -1.641612 1.276973 -1.430451

C -3.592130 1.521797 -3.443908

C -2.280784 2.505128 -1.662486

C -1.994226 0.175446 -2.222316

C -2.962916 0.296521 -3.221637

C -3.249471 2.626452 -2.659180

H -2.017307 3.374958 -1.069039

H -1.500706 -0.775676 -2.038925

H -3.224323 -0.569869 -3.824537

H -3.729651 3.587809 -2.830680

H -4.342849 1.618386 -4.225402

C -0.958108 1.479307 1.479647

C -2.279802 1.245796 1.916989

C -0.022063 1.966017 2.412584

C -2.631022 1.612975 3.229745

C -0.381570 2.302952 3.713096

H 1.015116 2.077067 2.112129

C -1.706937 2.146063 4.120509

H -3.654181 1.440159 3.552036

H 0.371112 2.676692 4.403108

H -2.010983 2.406538 5.131713

C 0.128458 -2.837921 1.512353

O -0.208614 -1.539178 1.614378

O 0.816606 -3.054563 0.408518

Rh 0.677644 -1.026599 -0.140319

O -0.192992 -3.685027 2.343210

Br 2.011705 -1.049433 -2.444858

C 3.091665 -1.111961 -0.720931

C 3.632385 -2.378218 -0.396065

C 3.674912 0.059287 -0.234965

C 4.627706 -2.414821 0.580258

C 4.699971 0.019080 0.725580

H 3.282434 1.008926 -0.576271

C 5.146902 -1.241130 1.137859

H 5.024794 -3.374101 0.896611

H 5.927519 -1.334675 1.884539

O 3.194007 -3.463521 -1.079089

C 3.167905 -4.714789 -0.400413

H 2.649956 -5.400460 -1.074430

H 4.182635 -5.097811 -0.214960

H 2.597704 -4.622047 0.526944

C -3.335147 0.517213 1.141060

C -4.501327 1.172276 0.696490

C -3.233639 -0.866566 0.979431

C -5.524659 0.434105 0.104143

C -4.257617 -1.636250 0.394419

H -2.315628 -1.334809 1.327985

C -5.398089 -0.953566 -0.035159

H -6.425884 0.920065 -0.254166

H -6.222599 -1.491047 -0.492530

O -4.544189 2.534069 0.885183

C -5.659671 3.234657 0.380109

H -5.486960 4.288596 0.613609

H -5.753723 3.115134 -0.708227

H -6.597838 2.912059 0.855511

C -4.085624 -3.159777 0.249405

C -2.965982 -3.454160 -0.777689

C -3.700859 -3.787222 1.611786

C -5.380251 -3.839195 -0.240333

H -3.229008 -3.050226 -1.763999

H -2.008748 -3.019087 -0.475622

H -2.818392 -4.537013 -0.881087

H -4.442261 -3.533461 2.380374

H -3.668956 -4.880860 1.520627

H -2.712841 -3.470977 1.959932

H -5.223092 -4.922265 -0.307117

H -6.217313 -3.665820 0.447911

H -5.679722 -3.490177 -1.236473

C 5.276833 1.338514 1.272485

C 5.874367 2.165802 0.109150

C 4.156962 2.159767 1.953123

C 6.391168 1.099310 2.309689

H 6.675868 1.611114 -0.393484

H 5.115457 2.411893 -0.640492

H 6.293622 3.110210 0.481973

H 3.705051 1.592332 2.774533

H 4.558814 3.097485 2.360571

H 3.362005 2.416238 1.246250

H 6.775538 2.061979 2.668819

H 6.021842 0.543391 3.179004

H 7.233406 0.542886 1.881658

**INT10A**

P -0.017550 0.683867 -0.214341

C 1.115151 2.123790 -0.522162

C 2.736417 4.334914 -1.154947

C 1.116812 3.266486 0.294165

C 1.932683 2.104443 -1.664238

C 2.734148 3.204058 -1.975072

C 1.924988 4.362537 -0.020048

H 0.487058 3.307330 1.175807

H 1.942028 1.223067 -2.300814

H 3.362534 3.169819 -2.861500

H 1.915437 5.237450 0.625775

H 3.366344 5.187555 -1.398046

C -1.385351 1.069433 -1.395530

C -3.342928 1.633783 -3.324587

C -1.627744 2.393019 -1.797924

C -2.129828 0.029217 -1.971362

C -3.104974 0.317845 -2.927148

C -2.602935 2.672102 -2.756005

H -1.051743 3.208742 -1.376055

H -1.944006 -0.992391 -1.660207

H -3.671666 -0.497560 -3.368538

H -2.770317 3.700662 -3.068536

H -4.095941 1.850011 -4.079401

C -0.539700 0.955961 1.543225

C -1.844341 1.119989 2.052442

C 0.549436 0.974276 2.438022

C -1.993295 1.389756 3.426671

C 0.378076 1.231300 3.792130

H 1.546361 0.762904 2.063666

C -0.906025 1.459836 4.289087

H -3.000327 1.519820 3.813120

H 1.239819 1.235423 4.454287

H -1.062465 1.661641 5.346175

C -0.910895 -2.444664 1.063230

O 0.286238 -1.956668 1.435377

O -1.124722 -2.282758 -0.225674

Rh 0.863612 -1.371427 -0.439361

O -1.690692 -2.960961 1.869565

Br 1.241371 -1.175602 -2.979353

C 2.833182 -1.078146 -0.014481

C 3.286457 -2.402093 0.034895

C 3.749954 -0.082679 0.313431

C 4.576391 -2.751095 0.397424

C 5.080436 -0.381092 0.693420

H 3.441002 0.957398 0.279041

C 5.472069 -1.723445 0.730650

H 4.897692 -3.789598 0.418499

H 6.482724 -1.997058 1.014695

O 2.299124 -3.318032 -0.364520

C 2.007903 -4.387068 0.544960

H 1.177296 -4.938085 0.100063

H 2.875411 -5.050514 0.646406

H 1.705051 -3.985539 1.515841

C -3.124085 0.924267 1.295009

C -3.870272 2.018027 0.817399

C -3.661736 -0.360437 1.194345

C -5.122774 1.798802 0.245062

C -4.923933 -0.609293 0.623360

H -3.069758 -1.186972 1.575109

C -5.635626 0.499171 0.156868

H -5.711688 2.625328 -0.137437

H -6.615781 0.374244 -0.292215

O -3.289829 3.255529 0.960803

C -3.981634 4.372535 0.442871

H -3.346210 5.239712 0.639910

H -4.144041 4.280102 -0.639412

H -4.952391 4.518706 0.938995

C -5.447219 -2.052993 0.504252

C -4.694096 -2.766579 -0.645347

C -5.191387 -2.834370 1.815419

C -6.959618 -2.089574 0.205377

H -4.873290 -2.258565 -1.601685

H -3.615223 -2.786327 -0.461570

H -5.045270 -3.803217 -0.741924

H -5.635956 -2.319507 2.676955

H -5.648677 -3.830451 1.745654

H -4.121919 -2.975383 1.998304

H -7.303769 -3.130420 0.179142

H -7.539712 -1.563426 0.974403

H -7.201532 -1.644809 -0.767554

C 6.042371 0.771463 1.043025

C 6.225055 1.686186 -0.191572

C 5.460044 1.604602 2.210121

C 7.434946 0.269200 1.472241

H 6.646226 1.122160 -1.032142

H 5.272914 2.112584 -0.521989

H 6.906290 2.517225 0.038853

H 5.317492 0.979684 3.099758

H 6.138893 2.426881 2.474880

H 4.491092 2.042470 1.950721

H 8.079190 1.123349 1.715725

H 7.380101 -0.370726 2.360782

H 7.925769 -0.299119 0.673668

**TS11**

P 2.927213 1.281492 0.139504

C 2.434133 2.873692 0.974705

C 1.495043 5.111242 2.408548

C 3.251260 4.001802 1.161437

C 1.141962 2.888092 1.522959

C 0.676870 3.993659 2.240075

C 2.783545 5.112249 1.865531

H 4.254425 4.019812 0.744336

H 0.492121 2.024502 1.409209

H -0.325320 3.964958 2.659550

H 3.428726 5.978986 1.994985

H 1.134215 5.977310 2.958953

C 3.917233 0.551571 1.531183

C 5.143947 -0.582007 3.790583

C 5.124868 1.071152 2.022386

C 3.331530 -0.543807 2.184321

C 3.943196 -1.104310 3.309785

C 5.735435 0.506620 3.142239

H 5.598196 1.910234 1.520422

H 2.393745 -0.947695 1.808554

H 3.477669 -1.951264 3.808164

H 6.670838 0.920112 3.514611

H 5.619035 -1.018459 4.666641

C 4.143668 1.830381 -1.152640

C 5.212667 1.038548 -1.639089

C 3.834855 3.020046 -1.845932

C 5.952276 1.498771 -2.744014

C 4.570532 3.451460 -2.945320

H 2.995184 3.621775 -1.512699

C 5.648784 2.689302 -3.395448

H 6.779243 0.888153 -3.096229

H 4.298456 4.377407 -3.446760

H 6.237254 3.009229 -4.252190

C 0.363729 -1.197551 -0.840999

O -0.627342 -0.645970 -1.587968

O 0.114485 -1.092916 0.443272

O 1.343292 -1.723300 -1.366616

Br -2.263312 1.024335 2.308158

C -2.952769 1.284201 -1.040573

C -3.568676 0.986021 -2.269700

C -3.019834 2.619639 -0.612318

C -4.219103 1.978844 -3.004702

C -3.643000 3.647650 -1.344882

H -2.568660 2.863349 0.342947

C -4.253394 3.298917 -2.554174

H -4.709586 1.707555 -3.937177

H -4.768761 4.041367 -3.155239

O -3.589052 -0.314436 -2.757113

C -2.803461 -0.515479 -3.932126

H -2.963480 -1.553708 -4.235832

H -3.125000 0.147965 -4.747045

H -1.742586 -0.355423 -3.714286

C 5.619410 -0.298442 -1.093628

C 6.890127 -0.446798 -0.496138

C 4.822200 -1.435314 -1.265604

C 7.332570 -1.709022 -0.108320

C 5.254738 -2.726957 -0.899365

H 3.832230 -1.303704 -1.692343

C 6.523167 -2.828337 -0.321874

H 8.303024 -1.840854 0.357785

H 6.911903 -3.794492 -0.018508

O 7.623400 0.704336 -0.327168

C 8.858112 0.608943 0.349660

H 9.251842 1.627011 0.404727

H 8.733099 0.212590 1.367064

H 9.576621 -0.024238 -0.191477

C 4.356706 -3.962761 -1.105229

C 3.293154 -4.009206 0.019104

C 3.636751 -3.896943 -2.472930

C 5.174385 -5.271530 -1.062654

H 3.771598 -4.022381 1.006509

H 2.622952 -3.147433 -0.045390

H 2.683205 -4.918433 -0.076136

H 4.356665 -3.789546 -3.294707

H 3.073865 -4.825269 -2.637004

H 2.918964 -3.074647 -2.512381

H 4.515908 -6.119398 -1.285598

H 5.985381 -5.271349 -1.802154

H 5.612779 -5.456913 -0.074848

C -3.639723 5.083786 -0.787915

C -4.336007 5.107188 0.593986

C -2.181365 5.576889 -0.632925

C -4.380512 6.070817 -1.711060

H -5.380515 4.784206 0.506942

H -3.841906 4.440662 1.307431

H -4.326112 6.122462 1.013877

H -1.669990 5.586198 -1.602762

H -2.160170 6.596979 -0.225664

H -1.603161 4.935019 0.038644

H -4.353514 7.078146 -1.277147

H -3.917373 6.124197 -2.703448

H -5.433301 5.793293 -1.841804

P -3.259797 -1.579224 0.231196

C -5.016885 -1.037297 0.097148

C -7.678505 -0.159665 -0.050343

C -5.490489 -0.053512 0.981921

C -5.881630 -1.555956 -0.876061

C -7.206022 -1.118466 -0.946323

C -6.815886 0.372852 0.911050

H -4.810374 0.389757 1.704308

H -5.521973 -2.286959 -1.590050

H -7.864295 -1.525720 -1.709850

H -7.169078 1.134484 1.601520

H -8.709848 0.180592 -0.107844

C -3.137179 -3.095145 -0.826797

C -2.968262 -5.465920 -2.324108

C -3.939839 -4.205176 -0.504215

C -2.241046 -3.190691 -1.898451

C -2.158574 -4.375414 -2.637374

C -3.862418 -5.377553 -1.253586

H -4.621667 -4.158882 0.339364

H -1.599616 -2.348793 -2.135639

H -1.446941 -4.439192 -3.456365

H -4.492022 -6.224914 -0.992842

H -2.898611 -6.384690 -2.901866

C -3.119975 -2.341599 1.911309

C -1.838272 -2.677548 2.379106

C -4.243254 -2.659644 2.687361

C -1.696176 -3.330111 3.603167

H -0.960678 -2.402540 1.798108

C -4.089960 -3.309165 3.914845

H -5.238226 -2.393062 2.346293

C -2.817299 -3.647662 4.374048

H -0.700224 -3.579262 3.960082

H -4.968621 -3.543742 4.511245

H -2.698173 -4.150183 5.331188

Rh -1.716821 0.015896 -0.013540

**3aa**

P -1.356252 0.017089 -0.624773

C -0.707470 1.690305 -0.153071

C 0.360302 4.240386 0.387861

C -0.618158 2.165204 1.165201

C -0.247767 2.514155 -1.193882

C 0.275472 3.780961 -0.928602

C -0.086709 3.429122 1.433515

H -0.961193 1.542255 1.986197

H -0.292515 2.154406 -2.219087

H 0.623194 4.405103 -1.747846

H -0.024009 3.780651 2.460448

H 0.774274 5.223168 0.597914

C -3.187916 0.301188 -0.701321

C -5.976015 0.586856 -0.997059

C -3.959490 -0.714396 -1.293969

C -3.838922 1.467559 -0.271097

C -5.220950 1.609591 -0.421221

C -5.340696 -0.579406 -1.431095

H -3.470163 -1.617098 -1.653119

H -3.265602 2.270785 0.181301

H -5.707120 2.522022 -0.084744

H -5.919360 -1.378889 -1.886858

H -7.050852 0.699306 -1.112903

C -1.169956 -0.934546 0.961089

C 0.076047 -1.551391 1.223357

C -2.214848 -1.098720 1.883364

C 0.238510 -2.294576 2.399700

C -2.040546 -1.849949 3.047186

H -3.176660 -0.633669 1.691197

C -0.809154 -2.448820 3.308244

H 1.200068 -2.762394 2.593712

H -2.865474 -1.962006 3.746277

H -0.662690 -3.035578 4.211410

C 1.242493 -1.406217 0.294534

C 2.219434 -0.434879 0.537385

C 1.394698 -2.261658 -0.813769

C 3.341673 -0.261510 -0.285917

H 2.071092 0.209377 1.396382

C 2.506669 -2.114154 -1.648716

C 3.456003 -1.126931 -1.381368

H 2.643245 -2.759655 -2.509159

H 4.303200 -1.039854 -2.056582

O 0.415835 -3.197054 -0.991073

C 0.493814 -4.045285 -2.122908

H -0.387865 -4.687541 -2.076579

H 0.476765 -3.472301 -3.059452

H 1.396800 -4.670595 -2.099776

C 4.399694 0.829120 -0.038172

C 4.410710 1.808694 -1.236282

C 4.114909 1.641603 1.239889

C 5.796119 0.179420 0.106773

H 4.647664 1.296949 -2.175480

H 3.432616 2.288963 -1.353100

H 5.162075 2.594194 -1.084601

H 4.104201 1.005614 2.132835

H 4.899755 2.394092 1.380054

H 3.156832 2.170234 1.183067

H 6.560407 0.947526 0.279138

H 5.817760 -0.517176 0.952911

H 6.082958 -0.378143 -0.791286

**TS11A**

P -0.701704 1.342017 0.282990

C 0.359203 2.780944 0.748506

C 1.974913 4.943704 1.514200

C 0.160687 4.050287 0.185563

C 1.378925 2.598979 1.699723

C 2.175222 3.681670 2.077340

C 0.965719 5.125574 0.567167

H -0.617940 4.203771 -0.553488

H 1.535701 1.611476 2.127895

H 2.960530 3.529781 2.813300

H 0.801642 6.104113 0.121497

H 2.602272 5.781413 1.810846

C -1.863693 2.013912 -0.997036

C -3.614947 3.148949 -2.883258

C -3.003408 2.725083 -0.581058

C -1.618921 1.869403 -2.370092

C -2.492493 2.433853 -3.302794

C -3.867948 3.292828 -1.518139

H -3.221852 2.835013 0.475423

H -0.768439 1.287932 -2.702321

H -2.295347 2.298299 -4.363427

H -4.743656 3.839426 -1.176355

H -4.292830 3.584507 -3.614160

C -1.825587 1.104218 1.718153

C -2.751205 0.040170 1.685293

C -1.819824 2.000655 2.798371

C -3.676925 -0.058924 2.738032

C -2.736891 1.870264 3.838361

H -1.093811 2.805081 2.825724

C -3.674757 0.837938 3.801477

H -4.389944 -0.876915 2.722365

H -2.712678 2.567829 4.671643

H -4.393069 0.719961 4.609422

Rh 0.365448 -0.595781 -0.059239

Br 0.007839 -1.158512 -2.566612

C 2.188179 0.170399 -0.577120

C 2.500865 1.188244 -1.492491

C 3.250144 -0.520467 0.016982

C 3.836228 1.457712 -1.815284

C 4.601626 -0.277731 -0.288891

H 3.005935 -1.260463 0.768576

C 4.868104 0.726377 -1.224671

H 4.087429 2.236479 -2.528141

H 5.892704 0.964549 -1.502181

O 1.454164 1.903747 -2.024750

C 1.741089 2.935288 -2.941774

H 2.354422 3.727391 -2.488611

H 2.253204 2.552876 -3.836207

H 0.775311 3.354540 -3.234229

C -2.758238 -1.036855 0.646151

C -3.951558 -1.310922 -0.054155

C -4.036271 -2.462808 -0.833079

C -1.748268 -3.082183 -0.229143

C -2.957444 -3.349882 -0.876626

H -4.939409 -2.698545 -1.386113

H -3.086870 -4.266065 -1.446137

O -4.979870 -0.401158 0.080086

C -6.140714 -0.595831 -0.696635

H -6.796092 0.252182 -0.480863

H -5.914314 -0.612112 -1.772153

H -6.663213 -1.527776 -0.432767

C -0.653192 -4.176312 -0.217904

C -0.531491 -4.876711 -1.590771

C 0.742277 -3.633906 0.152153

C -1.050827 -5.218022 0.859298

H -1.437379 -5.430230 -1.864172

H -0.321740 -4.141383 -2.374010

H 0.291144 -5.603464 -1.564599

H 0.751684 -3.191408 1.150537

H 1.472917 -4.454375 0.147472

H 1.090553 -2.909486 -0.597743

H -0.316316 -6.034669 0.896836

H -1.092731 -4.749308 1.848540

H -2.033721 -5.655691 0.644731

C 5.755450 -1.052551 0.375085

C 6.670071 -0.065694 1.139904

C 5.254317 -2.109369 1.378385

C 6.586303 -1.776561 -0.710816

H 7.092272 0.694977 0.473645

H 6.107737 0.452783 1.924736

H 7.505953 -0.598445 1.613746

H 4.626660 -2.864170 0.891374

H 6.110404 -2.628158 1.828073

H 4.670817 -1.660172 2.189143

H 7.421636 -2.327477 -0.257138

H 5.964224 -2.491811 -1.261228

H 7.005749 -1.072638 -1.438514

C -1.625024 -1.872456 0.506483

H -0.853263 -1.864103 1.621712

O -0.364101 -1.967992 2.769461

C 0.591543 -1.094942 2.950379

O 0.992482 -0.365698 1.879671

O 1.172614 -0.894353 4.017785

**INT11A**

P -1.335816 -0.832304 0.637398

C -2.284078 -2.334149 0.092639

C -3.787655 -4.553636 -0.753154

C -2.157722 -3.570111 0.741911

C -3.181525 -2.219577 -0.983584

C -3.921158 -3.324250 -1.402272

C -2.907142 -4.671274 0.321492

H -1.473818 -3.686739 1.574045

H -3.281221 -1.276723 -1.507388

H -4.592784 -3.217226 -2.249620

H -2.791363 -5.623041 0.834899

H -4.363650 -5.414463 -1.085443

C -0.663786 -1.353754 2.274887

C 0.348797 -2.252296 4.733422

C -1.277575 -0.974422 3.476017

C 0.468566 -2.182970 2.314107

C 0.965216 -2.631644 3.539265

C -0.772555 -1.421237 4.699069

H -2.144902 -0.322375 3.458754

H 0.951055 -2.473875 1.385247

H 1.843903 -3.271178 3.556379

H -1.257170 -1.117328 5.624148

H 0.742334 -2.599414 5.686121

C -2.687683 0.362820 0.921801

C -2.605557 1.706401 0.493773

C -3.906990 -0.154602 1.404387

C -3.826249 2.420460 0.439295

C -5.067718 0.606582 1.426791

H -3.948026 -1.188713 1.730926

C -5.027204 1.896491 0.894254

H -3.818224 3.418301 0.019952

H -5.995088 0.182525 1.803094

H -5.933944 2.492943 0.823714

Rh 0.094672 -0.085391 -0.912741

Br 0.891519 -2.635396 -1.442132

C 1.679435 0.162255 0.425196

C 1.658701 0.893752 1.633074

C 2.924838 -0.380182 0.060858

C 2.821045 1.072208 2.394910

C 4.112374 -0.221437 0.798104

H 2.954828 -0.984710 -0.835511

C 4.032872 0.524109 1.978299

H 2.790492 1.632355 3.323627

H 4.913217 0.679334 2.598573

O 0.447656 1.395071 2.043427

C 0.388396 2.211663 3.191325

H 0.630332 1.651356 4.106425

H 1.065241 3.073826 3.109302

H -0.640611 2.573581 3.246134

C -1.375639 2.447224 0.068909

C -1.280674 3.802700 0.491973

C -0.314861 4.651675 -0.034342

C 0.561545 2.807472 -1.379661

C 0.544279 4.154954 -1.007201

H -0.234586 5.685531 0.279989

H 1.245196 4.846927 -1.466770

O -2.137562 4.216822 1.498409

C -2.084879 5.562934 1.913990

H -2.854962 5.670823 2.682694

H -1.107770 5.823761 2.346420

H -2.298861 6.258554 1.089533

C 1.557041 2.442112 -2.508905

C 3.015433 2.684095 -2.052334

C 1.419809 0.990137 -2.980604

C 1.261145 3.322977 -3.750632

H 3.179943 3.728850 -1.764432

H 3.266301 2.052898 -1.194952

H 3.713360 2.450680 -2.868398

H 0.432342 0.774049 -3.388990

H 2.156606 0.759938 -3.761593

H 1.683688 0.266371 -2.184394

H 1.932300 3.048237 -4.575153

H 0.229202 3.178617 -4.089146

H 1.405589 4.389897 -3.549903

C 5.449339 -0.854177 0.365919

C 5.891550 -1.890889 1.426892

C 5.348638 -1.574280 -0.992896

C 6.536238 0.240436 0.246418

H 6.009292 -1.430835 2.414600

H 5.147881 -2.690853 1.518318

H 6.852083 -2.347354 1.150439

H 5.061043 -0.884205 -1.794094

H 6.322870 -2.005698 -1.256427

H 4.614309 -2.386072 -0.973281

H 7.496018 -0.199195 -0.057714

H 6.251861 0.988928 -0.502241

H 6.695142 0.763531 1.195856

C -0.335189 1.909232 -0.729231

C -1.836744 -0.627216 -3.484869

O -1.578078 -0.095588 -2.324475

O -2.848745 -0.412106 -4.142975

O -0.893854 -1.483237 -4.004105

H -0.286982 -1.757052 -3.284702

**TS11B**

P 0.328489 -0.210620 0.671915

C 1.612561 -1.169224 1.579767

C 3.352606 -2.475093 3.355170

C 3.006520 -1.098615 1.365414

C 1.112052 -1.938342 2.643896

C 1.974139 -2.580425 3.530323

C 3.853972 -1.751263 2.275997

H 0.038631 -2.039391 2.767817

H 1.561837 -3.173334 4.342239

H 4.926079 -1.703909 2.104217

H 4.036552 -2.972829 4.038783

C 0.663256 0.253363 -1.064122

C 0.429620 1.000183 -3.738371

C 1.346918 1.383491 -1.531130

C -0.253753 -0.120117 -3.267582

C 1.239343 1.745889 -2.873068

H 1.958119 1.974179 -0.856285

H -0.858956 -0.718275 -3.944234

H 1.785675 2.609955 -3.243710

H 0.342106 1.293485 -4.782688

C 0.219562 1.367478 1.623411

C 0.564740 1.419730 2.983232

C -0.256666 2.537210 1.007425

C 0.443054 2.609164 3.703228

H 0.929690 0.530095 3.483838

C -0.368969 3.726980 1.727485

H -0.538456 2.519125 -0.039073

C -0.020467 3.767507 3.078913

H 0.713844 2.626250 4.755959

H -0.734033 4.621644 1.229287

H -0.111759 4.694126 3.640332

Rh -1.521418 -1.297840 0.036680

Br -2.548720 -1.637034 2.271765

C -3.039578 0.067466 -0.347363

C -3.927790 -0.720286 -1.094933

C -3.408094 1.389722 -0.127519

C -5.095231 -0.228784 -1.658475

C -4.593639 1.946666 -0.667367

H -2.767618 2.020563 0.482342

C -5.416360 1.120490 -1.439352

H -5.757856 -0.866163 -2.239103

H -6.331792 1.505198 -1.877233

O -3.498276 -2.041354 -1.159396

C -3.680311 -2.791468 -2.362392

H -2.827852 -3.470735 -2.417347

H -4.632759 -3.336275 -2.327987

H -3.674361 -2.122951 -3.232391

C 3.666023 -0.442810 0.194175

C 3.670979 -1.111323 -1.051941

C 4.384387 0.747176 0.333988

C 4.380420 -0.550477 -2.121512

C 5.098795 1.330407 -0.725911

H 4.370213 1.220132 1.310379

C 5.073397 0.648107 -1.950333

H 4.390043 -1.043972 -3.086887

H 5.611085 1.051553 -2.805472

O 2.986655 -2.270745 -1.096169

C 2.867485 -2.996001 -2.320644

H 3.855922 -3.327916 -2.670009

H 2.225379 -3.847036 -2.079636

H 2.388579 -2.379871 -3.090748

C -0.141036 -0.522605 -1.927223

H -0.455505 -1.687797 -1.804240

O -0.589818 -3.147638 -2.032436

C -0.092201 -3.681829 -0.940419

O -0.240685 -2.974627 0.190961

O 0.515071 -4.765786 -0.912520

C -4.929644 3.425221 -0.391166

C -5.034220 3.660897 1.134691

C -3.814622 4.329276 -0.969200

C -6.264078 3.858102 -1.029279

H -5.825472 3.039035 1.569013

H -4.100422 3.410957 1.647715

H -5.267846 4.713159 1.348918

H -3.725752 4.192246 -2.053422

H -4.031038 5.388910 -0.774142

H -2.841403 4.096853 -0.525288

H -6.460691 4.913617 -0.802220

H -6.246420 3.750708 -2.120158

H -7.106228 3.271920 -0.643731

C 5.892007 2.642712 -0.583240

C 5.358552 3.689801 -1.590059

C 5.780924 3.243972 0.831159

C 7.389025 2.379190 -0.874386

H 5.445966 3.339183 -2.624050

H 4.301189 3.908492 -1.400979

H 5.921118 4.629140 -1.506357

H 6.178231 2.564667 1.593945

H 6.356738 4.175923 0.884457

H 4.743141 3.478680 1.093396

H 7.969994 3.306999 -0.786805

H 7.801160 1.650719 -0.166489

H 7.540937 1.983179 -1.884294

**INT11B**

P 0.342570 -0.050670 0.514114

C 1.483299 -0.979915 1.631724

C 2.937810 -2.168958 3.720455

C 2.893571 -1.013788 1.566558

C 0.825570 -1.590776 2.712139

C 1.545023 -2.179728 3.750253

C 3.595881 -1.601678 2.631134

H -0.261073 -1.629969 2.726576

H 1.010663 -2.652222 4.570170

H 4.680895 -1.634156 2.574929

H 3.512423 -2.620782 4.525793

C 0.757478 0.220287 -1.233811

C 0.423820 0.216360 -3.968065

C 1.723161 0.856932 -2.017517

C -0.541175 -0.408897 -3.172112

C 1.540339 0.850436 -3.402690

H 2.598671 1.327228 -1.580357

H -1.405850 -0.885221 -3.626335

H 2.273495 1.332449 -4.045145

H 0.305892 0.216734 -5.050758

C 0.146116 1.548462 1.401209

C -0.764153 1.619860 2.468499

C 0.892674 2.682908 1.054674

C -0.903318 2.807189 3.188623

H -1.380293 0.756780 2.710631

C 0.744113 3.870866 1.774623

H 1.581810 2.644175 0.216740

C -0.149331 3.933177 2.845648

H -1.611967 2.854535 4.011468

H 1.324790 4.746782 1.495353

H -0.265257 4.858229 3.405314

Rh -1.491911 -1.101994 -0.254155

Br -2.838134 -1.874430 1.980401

C -2.813894 0.405030 -0.568848

C -3.799987 -0.267637 -1.299660

C -3.028779 1.753392 -0.305422

C -4.974703 0.325733 -1.727072

C -4.204273 2.419021 -0.728614

H -2.282634 2.309980 0.253346

C -5.166985 1.684702 -1.430014

H -5.726659 -0.229757 -2.281919

H -6.084740 2.156114 -1.765377

O -3.414647 -1.591643 -1.572986

C -4.377280 -2.627142 -1.321509

H -3.821374 -3.564561 -1.354807

H -4.816913 -2.496121 -0.328079

H -5.155387 -2.613184 -2.094956

C 3.710530 -0.542533 0.406630

C 3.753995 -1.332741 -0.764867

C 4.522172 0.591515 0.496552

C 4.607857 -0.952824 -1.809210

C 5.375600 0.998229 -0.543312

H 4.468605 1.165575 1.415618

C 5.396584 0.191314 -1.690612

H 4.651749 -1.545596 -2.716192

H 6.043475 0.453094 -2.525132

O 2.959970 -2.419382 -0.769416

C 2.685284 -3.104581 -1.993405

H 3.573828 -3.654880 -2.336360

H 1.861131 -3.788049 -1.769798

H 2.372286 -2.392093 -2.765807

C -0.379455 -0.404189 -1.782869

C -0.639848 -4.061821 -0.585907

O -0.218220 -2.884872 -0.265372

O -0.053215 -4.881889 -1.292194

C -4.384739 3.913917 -0.398044

C -4.396315 4.107619 1.137216

C -3.215680 4.726129 -1.005027

C -5.700470 4.488510 -0.957929

H -5.223092 3.549011 1.591480

H -3.466991 3.755426 1.595263

H -4.518237 5.169389 1.393191

H -3.190280 4.614075 -2.095461

H -3.325156 5.794545 -0.773237

H -2.248283 4.395463 -0.614713

H -5.778312 5.553637 -0.706373

H -5.750259 4.399697 -2.049551

H -6.576447 3.983079 -0.534917

C 6.266210 2.251459 -0.454603

C 5.914552 3.226724 -1.603368

C 6.086840 3.003599 0.878096

C 7.753378 1.840796 -0.576330

H 6.050968 2.762123 -2.585663

H 4.870313 3.552600 -1.532470

H 6.553024 4.119290 -1.563609

H 6.360075 2.380597 1.737397

H 6.730975 3.891177 0.893659

H 5.053435 3.340139 1.018669

H 8.405737 2.722347 -0.519089

H 8.034837 1.155213 0.231385

H 7.956620 1.335327 -1.526599

O -1.870255 -4.430865 -0.067292

H -2.132266 -3.745915 0.585760

**TS11C**

P 0.401208 -0.706416 0.386186

C 0.654593 0.587012 1.674930

C 0.976168 2.510131 3.700443

C 1.920456 1.135067 1.945853

C -0.441832 0.994241 2.450902

C -0.279199 1.953972 3.453366

C 2.077101 2.092845 2.948621

H 2.789371 0.810833 1.385857

H -1.409906 0.535819 2.288687

H -1.138849 2.252760 4.047321

H 3.063533 2.507244 3.140662

H 1.100971 3.253403 4.484854

C 1.745817 -1.919068 0.660779

C 3.698553 -3.891172 1.069866

C 2.322544 -2.094605 1.926336

C 2.131317 -2.764930 -0.391526

C 3.109005 -3.737223 -0.187786

C 3.300495 -3.071870 2.126784

H 2.006040 -1.478556 2.760999

H 1.653965 -2.679596 -1.359809

H 3.385394 -4.394515 -1.007336

H 3.737646 -3.198867 3.114133

H 4.452287 -4.658799 1.228425

C 0.474580 -0.036635 -1.335703

C 1.230730 1.022086 -1.883662

C 0.838028 1.542020 -3.126808

C -0.969237 -0.049924 -3.291363

C -0.258454 1.025924 -3.817467

H 1.416290 2.356355 -3.555102

H -1.792153 -0.498775 -3.840568

H -0.538641 1.453825 -4.777774

Rh -1.634824 -1.508757 0.002514

Br -2.203230 -2.100774 2.345983

C -2.895902 0.147462 0.007234

C -4.117822 -0.388168 -0.431640

C -2.830368 1.532023 0.132166

C -5.227476 0.394475 -0.720005

C -3.929251 2.380254 -0.147743

H -1.889185 1.990241 0.426352

C -5.122456 1.786428 -0.567810

H -6.160598 -0.051463 -1.055926

H -5.993781 2.392962 -0.793451

O -4.066968 -1.767992 -0.555873

C -4.742079 -2.394948 -1.645231

H -5.758691 -2.686273 -1.348109

H -4.793170 -1.719225 -2.507440

H -4.140748 -3.264666 -1.910327

C 2.477432 1.560258 -1.263763

C 2.553523 2.884068 -0.786501

C 3.622517 0.755976 -1.201131

C 3.756997 3.354138 -0.250264

C 4.842847 1.202785 -0.670791

H 3.533138 -0.254288 -1.583146

C 4.874195 2.519804 -0.193020

H 3.831998 4.364818 0.135290

H 5.787866 2.920868 0.238094

O 1.411666 3.629025 -0.866527

C 1.388451 4.894286 -0.229729

H 0.371883 5.271156 -0.354799

H 1.614598 4.804721 0.840772

H 2.094901 5.597632 -0.693231

C -0.609818 -0.597887 -2.054053

H -1.114253 -1.710499 -1.980291

O -1.726014 -3.037942 -2.294348

C -1.062798 -3.872555 -1.516206

O -0.681415 -3.362400 -0.328942

O -0.771918 -5.034989 -1.814863

C 6.096162 0.311753 -0.585807

C 6.404384 0.009270 0.900465

C 5.913265 -1.029113 -1.323266

C 7.305201 1.039789 -1.220270

H 6.561957 0.929876 1.474477

H 5.578749 -0.542232 1.364224

H 7.312203 -0.601842 0.988772

H 5.684511 -0.878464 -2.384931

H 6.841551 -1.609860 -1.262793

H 5.117118 -1.638522 -0.884123

H 8.199351 0.405699 -1.169169

H 7.113272 1.271430 -2.274495

H 7.539016 1.979753 -0.708631

C -3.762291 3.905376 -0.002641

C -3.336421 4.251693 1.444287

C -2.674187 4.401343 -0.985875

C -5.062420 4.674754 -0.306885

H -4.096625 3.919245 2.161116

H -2.392373 3.768821 1.714980

H -3.207215 5.336842 1.562240

H -2.969825 4.203255 -2.022853

H -2.517954 5.484527 -0.877594

H -1.716791 3.897456 -0.818613

H -4.896278 5.752540 -0.183540

H -5.402224 4.506692 -1.335334

H -5.873276 4.382633 0.370689

**INT11C**

P 0.320273 -0.263797 0.511066

C -0.021878 1.194591 1.564506

C -0.578012 3.332132 3.286809

C 0.413389 2.486705 1.243298

C -0.763001 0.979962 2.739794

C -1.030950 2.046176 3.598094

C 0.134379 3.551075 2.106433

H 0.947106 2.670514 0.316795

H -1.157647 -0.011393 2.953250

H -1.607398 1.874109 4.503332

H 0.473321 4.552575 1.851959

H -0.792583 4.162262 3.955685

C 1.649392 -1.155865 1.422997

C 3.645450 -2.557296 2.808033

C 2.372976 -0.529988 2.450656

C 1.934282 -2.490660 1.088687

C 2.931552 -3.180326 1.781463

C 3.365524 -1.229671 3.139498

H 2.161736 0.498739 2.721926

H 1.352630 -2.975658 0.305911

H 3.136245 -4.216286 1.523685

H 3.911479 -0.738014 3.941387

H 4.412161 -3.104434 3.352064

C 0.751187 0.168186 -1.206261

C 1.781353 0.796011 -1.929271

C 1.603436 0.879546 -3.318791

C -0.517997 -0.321610 -3.211168

C 0.474069 0.335586 -3.942386

H 2.371109 1.363145 -3.917649

H -1.374103 -0.765009 -3.711886

H 0.382863 0.413945 -5.024646

Rh -1.456433 -1.319857 -0.381139

Br -2.619867 -2.376093 1.823367

C -2.834330 0.174828 -0.452278

C -3.808818 -0.435366 -1.251195

C -3.104372 1.463080 -0.001968

C -5.012236 0.161389 -1.583564

C -4.313717 2.128335 -0.316915

H -2.378130 1.970249 0.624068

C -5.255378 1.458179 -1.105129

H -5.749140 -0.347983 -2.199325

H -6.196056 1.931715 -1.365445

O -3.391800 -1.703413 -1.692059

C -4.321843 -2.787152 -1.524464

H -4.743389 -2.762829 -0.515041

H -5.116499 -2.717179 -2.277587

H -3.745475 -3.702699 -1.656412

C 3.055841 1.300471 -1.329881

C 3.309343 2.678557 -1.187820

C 4.070947 0.398148 -0.987919

C 4.546929 3.113911 -0.704429

C 5.329734 0.805729 -0.520750

H 3.850739 -0.655165 -1.116357

C 5.536150 2.184079 -0.383078

H 4.753573 4.171336 -0.580174

H 6.489902 2.558555 -0.020035

O 2.284352 3.522875 -1.533673

C 2.505425 4.917461 -1.457137

H 1.580420 5.385943 -1.799604

H 2.716034 5.243453 -0.428663

H 3.334298 5.232862 -2.106244

C -0.377846 -0.411057 -1.824377

C -0.709771 -4.288253 -1.136163

O -0.249387 -3.112541 -0.821648

O -0.213780 -5.067598 -1.939272

C 6.460848 -0.184476 -0.185329

C 6.833960 -0.055275 1.310568

C 6.059227 -1.646611 -0.458177

C 7.703071 0.138914 -1.050122

H 7.165273 0.959463 1.559649

H 5.974206 -0.295647 1.944836

H 7.649881 -0.745089 1.563312

H 5.803283 -1.807095 -1.511727

H 6.899678 -2.309047 -0.217756

H 5.205843 -1.959471 0.151580

H 8.520071 -0.557127 -0.820100

H 7.470352 0.048443 -2.117550

H 8.072548 1.155310 -0.873826

C -4.559051 3.549806 0.227942

C -4.578126 3.513638 1.774739

C -3.429795 4.495464 -0.246454

C -5.899779 4.141955 -0.248604

H -5.380255 2.859531 2.136133

H -3.634607 3.136741 2.180645

H -4.746210 4.519773 2.183988

H -3.395104 4.539345 -1.341547

H -3.595150 5.513858 0.131831

H -2.448696 4.161969 0.104823

H -6.025391 5.153150 0.158663

H -5.946238 4.214635 -1.341626

H -6.752314 3.541223 0.088790

O -1.864449 -4.680995 -0.483297

H -2.048479 -4.037433 0.236575

**INT12A**

P -0.012975 0.745405 1.455121

C -1.690627 0.313273 2.087565

C -4.274229 -0.466768 2.885051

C -1.870542 -0.515054 3.203734

C -2.830649 0.782925 1.402119

C -4.110154 0.389742 1.787411

C -3.157225 -0.892131 3.602244

H -1.017155 -0.888626 3.756724

H -2.707512 1.433882 0.545522

H -4.997961 0.670970 1.214892

H -3.273936 -1.550162 4.460821

H -5.274573 -0.825302 3.100667

C 1.092526 0.316281 2.864544

C 2.670507 -0.403082 5.070114

C 1.564041 1.296146 3.748112

C 1.419546 -1.030136 3.091989

C 2.201679 -1.383284 4.192951

C 2.350622 0.937198 4.844884

H 1.322610 2.340810 3.579517

H 1.056154 -1.794038 2.410838

H 2.448346 -2.428687 4.358393

H 2.710665 1.707335 5.522996

H 3.282225 -0.682274 5.924726

C -0.094004 2.565713 1.309812

C 0.457058 3.267632 0.213549

C -0.901693 3.238169 2.248993

C 0.039376 4.610615 0.056257

C -1.223769 4.581977 2.109086

H -1.316021 2.679170 3.081328

C -0.775398 5.259336 0.974069

H 0.374075 5.153954 -0.818458

H -1.851682 5.074579 2.846407

H -1.064742 6.292931 0.798494

Rh 0.394820 -0.170070 -0.566027

C 2.251343 -0.912878 -0.036503

C 3.329728 -0.180409 0.506679

C 2.505983 -2.262153 -0.336000

C 4.578382 -0.784128 0.707771

C 3.746196 -2.897948 -0.147482

H 1.679695 -2.850053 -0.709759

C 4.782872 -2.123004 0.381125

H 5.400127 -0.216204 1.130805

H 5.765598 -2.554869 0.557097

O 3.099979 1.127077 0.847309

C 4.153698 1.903038 1.375149

H 4.489205 1.523960 2.350833

H 5.011181 1.942585 0.688811

H 3.747632 2.909799 1.493885

C 1.403490 2.736268 -0.819867

C 2.435013 3.618487 -1.247167

C 3.208678 3.324826 -2.364393

C 2.002452 1.215397 -2.641834

C 2.934019 2.163992 -3.077047

H 3.996091 3.989192 -2.700033

H 3.494644 1.985754 -3.990853

O 2.679922 4.734511 -0.467775

C 3.702068 5.622443 -0.864935

H 3.710871 6.422051 -0.119689

H 4.687213 5.134670 -0.880385

H 3.509909 6.056761 -1.856652

C 1.788819 0.005154 -3.587006

C 3.080692 -0.838703 -3.698887

C 0.634152 -0.906288 -3.151576

C 1.407262 0.526617 -4.997583

H 3.918193 -0.241909 -4.077676

H 3.368865 -1.247285 -2.726395

H 2.928861 -1.676835 -4.392925

H -0.325062 -0.387657 -3.127219

H 0.530423 -1.755861 -3.839344

H 0.824857 -1.405298 -2.182966

H 1.206641 -0.318451 -5.668988

H 0.504288 1.146160 -4.951608

H 2.203483 1.125224 -5.452023

C 3.973670 -4.385888 -0.476207

C 4.340388 -5.147551 0.820326

C 2.724726 -5.052685 -1.084761

C 5.133560 -4.526941 -1.490541

H 5.244820 -4.740410 1.286113

H 3.527480 -5.078636 1.552216

H 4.520024 -6.210436 0.609230

H 2.432705 -4.577231 -2.027952

H 2.935416 -6.108866 -1.294667

H 1.864631 -5.007906 -0.408665

H 5.312691 -5.584263 -1.727915

H 4.898703 -4.003484 -2.424658

H 6.068906 -4.109396 -1.101659

C 1.318952 1.451837 -1.413386

O -1.449186 0.860662 -1.406798

C -2.572986 0.580440 -1.916767

O -3.143325 -0.492651 -2.139676

O -3.303711 1.730957 -2.284299

H -2.745136 2.481862 -2.022189

Br -0.785653 -2.515318 0.215258

K -4.008528 -2.382332 -0.236921

O -6.342473 -1.694539 -1.188582

C -6.483619 -1.090998 -0.020720

O -6.767574 0.182642 0.000244

O -6.220997 -1.743363 1.057411

K -5.800869 0.466198 -2.324403

**TS13A**

P 0.361461 -1.335864 -1.222104

C -1.178323 -1.481543 -2.249836

C -3.575624 -1.641835 -3.710334

C -1.192696 -1.203611 -3.624645

C -2.374707 -1.869464 -1.622441

C -3.570263 -1.931997 -2.341575

C -2.383977 -1.292601 -4.349371

H -0.285907 -0.906006 -4.137262

H -2.389931 -2.088743 -0.562794

H -4.492931 -2.149893 -1.810462

H -2.376930 -1.072717 -5.414723

H -4.506132 -1.687063 -4.271801

C 1.684020 -1.185258 -2.497415

C 3.614743 -0.932929 -4.517973

C 2.466129 -2.285920 -2.871869

C 1.878025 0.047330 -3.141335

C 2.837149 0.166601 -4.148697

C 3.427473 -2.159184 -3.877046

H 2.330601 -3.240887 -2.374209

H 1.274753 0.903557 -2.853755

H 2.979736 1.126570 -4.638056

H 4.029266 -3.020677 -4.157023

H 4.363867 -0.834477 -5.300201

C 0.473304 -2.989689 -0.459542

C 0.835174 -3.166973 0.894164

C -0.006947 -4.077824 -1.215468

C 0.557642 -4.435210 1.455104

C -0.185397 -5.334969 -0.653796

H -0.281659 -3.919593 -2.253213

C 0.064253 -5.496493 0.709984

H 0.733109 -4.578253 2.513645

H -0.561097 -6.156858 -1.257321

H -0.135914 -6.447448 1.197908

Rh 0.179255 0.251539 0.364594

C 2.008705 1.136206 -0.083477

C 3.265801 0.495519 -0.143461

C 2.023134 2.533384 -0.240085

C 4.444407 1.231551 -0.326156

C 3.185252 3.304213 -0.422983

H 1.068004 3.040089 -0.241473

C 4.403069 2.618029 -0.459175

H 5.404171 0.728035 -0.375347

H 5.339379 3.153425 -0.601501

O 3.282765 -0.872540 -0.039974

C 4.518872 -1.552520 -0.026640

H 5.046881 -1.459911 -0.986627

H 5.171655 -1.189408 0.779441

H 4.278557 -2.601647 0.157890

C 1.451562 -2.151303 1.807068

C 2.462396 -2.627008 2.687835

C 2.923477 -1.842544 3.738796

C 1.435830 -0.033710 3.034316

C 2.355515 -0.588867 3.930148

H 3.691246 -2.196000 4.416900

H 2.669207 -0.017365 4.799812

O 3.011374 -3.866295 2.411728

C 3.980125 -4.385320 3.295865

H 4.244382 -5.371940 2.906184

H 4.883991 -3.759949 3.330870

H 3.589182 -4.494085 4.317785

C 0.868073 1.352477 3.429420

C 1.992618 2.414440 3.460484

C -0.249832 1.833954 2.497059

C 0.230216 1.250944 4.839945

H 2.780934 2.145701 4.172960

H 2.451734 2.526894 2.474240

H 1.587714 3.389630 3.764915

H -1.121546 1.178859 2.488992

H -0.596716 2.833261 2.794852

H 0.105247 2.009675 1.463495

H -0.214689 2.214956 5.121755

H -0.560266 0.492024 4.852433

H 0.961138 0.988685 5.612077

C 3.148569 4.834808 -0.597257

C 3.727826 5.209192 -1.982671

C 1.719397 5.404663 -0.509898

C 4.000562 5.507002 0.505744

H 4.760593 4.861297 -2.096841

H 3.133034 4.757152 -2.784661

H 3.722189 6.298440 -2.125790

H 1.263342 5.203809 0.466093

H 1.746815 6.492922 -0.648276

H 1.063129 4.982229 -1.277879

H 3.994970 6.599244 0.388589

H 3.605677 5.269358 1.500426

H 5.043516 5.172803 0.474445

C 1.079013 -0.785986 1.878397

O -1.643302 -0.783797 1.103003

C -2.850498 -0.382320 1.227632

O -3.233715 0.811979 1.331106

O -3.745901 -1.396883 1.240900

H -4.668899 -1.063381 1.065985

Br -1.027073 1.920015 -1.498959

K -4.167320 1.217572 -1.311731

O -6.308501 1.636065 0.141834

C -6.278250 0.352578 0.437269

O -6.528518 -0.037274 1.646720

O -5.857151 -0.499326 -0.445891

K -5.294656 1.957352 2.461414

**INT13A**

P -1.655648 -1.504876 -0.431288

C -0.956165 -3.140384 -0.908784

C 0.058639 -5.654797 -1.624993

C -1.808568 -4.131292 -1.427242

C 0.405805 -3.424609 -0.739190

C 0.905208 -4.680506 -1.095044

C -1.300427 -5.377643 -1.792394

H -2.870807 -3.934020 -1.537997

H 1.062472 -2.663600 -0.332552

H 1.962698 -4.890446 -0.957936

H -1.968505 -6.133896 -2.197235

H 0.453764 -6.629369 -1.901570

C -3.242454 -2.003700 0.381060

C -5.619943 -2.905292 1.569209

C -4.470406 -1.849132 -0.280191

C -3.213898 -2.609495 1.648629

C -4.400351 -3.054381 2.233482

C -5.652056 -2.300251 0.312268

H -4.510608 -1.370065 -1.253258

H -2.266876 -2.698311 2.173043

H -4.367467 -3.516105 3.217143

H -6.596885 -2.172470 -0.210821

H -6.540787 -3.254936 2.030486

C -2.142325 -0.610208 -1.934869

C -2.619250 0.704250 -1.738992

C -1.907299 -1.113996 -3.219506

C -2.805190 1.494929 -2.887631

C -2.126443 -0.317073 -4.340377

H -1.531257 -2.124944 -3.337516

C -2.559765 0.998676 -4.164967

H -3.141098 2.518100 -2.769743

H -1.940078 -0.711607 -5.335962

H -2.702802 1.645953 -5.027113

C 0.399731 1.083705 -0.716727

C 1.118296 0.553496 -1.813979

C 0.458527 2.474592 -0.542673

C 1.877458 1.391748 -2.645258

C 1.219974 3.346487 -1.346775

H -0.118879 2.898863 0.268335

C 1.930785 2.769800 -2.405838

H 2.429909 0.981689 -3.484343

H 2.523509 3.387553 -3.078103

O 1.029275 -0.793490 -2.027055

C 1.769647 -1.369239 -3.095898

H 1.477412 -0.933424 -4.061863

H 2.848588 -1.266142 -2.928213

H 1.513536 -2.430125 -3.089888

C -2.933800 1.254656 -0.380620

C -4.166510 1.947949 -0.249304

C -4.498232 2.570189 0.945491

C -2.402900 1.785987 1.963626

C -3.602343 2.505766 2.008421

H -5.432513 3.105940 1.066228

H -3.879402 3.034200 2.913193

O -5.018651 1.934624 -1.333583

C -6.279272 2.555824 -1.202758

H -6.789903 2.402600 -2.157028

H -6.876036 2.105369 -0.397136

H -6.189258 3.635191 -1.013233

C -2.085188 1.089028 0.752305

C -1.542416 1.787011 3.260679

C -1.920160 0.571617 4.141876

C -0.025265 1.774221 2.985073

C -1.784262 3.069396 4.099868

H -2.997209 0.574179 4.351783

H -1.649679 -0.369695 3.659117

H -1.387099 0.621569 5.101553

H 0.299401 2.626331 2.383398

H 0.529493 1.803073 3.930587

H 0.335947 0.852177 2.506786

H -1.058658 3.104783 4.920707

H -1.659855 3.979397 3.500476

H -2.778511 3.094195 4.559187

C 1.285207 4.867342 -1.106851

C 0.767647 5.614595 -2.359310

C 0.434779 5.312450 0.098306

C 2.750289 5.286675 -0.833080

H 1.363888 5.379753 -3.247974

H -0.271248 5.338031 -2.573449

H 0.807841 6.701529 -2.206802

H 0.776929 4.846276 1.028730

H 0.511427 6.399597 0.223369

H -0.624268 5.066007 -0.037052

H 2.821042 6.370013 -0.667387

H 3.138463 4.783525 0.060736

H 3.406104 5.038447 -1.676026

Rh -0.402184 -0.102034 0.757272

O -0.466785 -1.652180 2.351430

C 0.808864 -1.942700 2.214276

O 1.412473 -2.832315 2.835215

O 1.420998 -1.180218 1.296475

Br 5.148556 -1.597560 -1.199288

K 4.305080 1.391222 -0.505888

O 5.228258 1.710477 1.995175

C 4.394277 0.864282 2.378213

O 4.507016 -0.145717 3.109206

O 3.102778 1.074054 1.799277

K 3.865472 -2.257936 1.713775

H 2.535932 0.289915 1.956311

**TS14A**

P 0.072675 0.642763 1.225303

C -0.797019 2.263334 1.402237

C -2.026584 4.780110 1.611841

C -1.535148 2.603195 2.547045

C -0.685482 3.199336 0.364065

C -1.294937 4.450355 0.470802

C -2.145027 3.854431 2.650927

H -1.636074 1.888874 3.359641

H -0.152544 2.924673 -0.540722

H -1.215434 5.157652 -0.350266

H -2.718074 4.104173 3.541142

H -2.514735 5.748400 1.686624

C -0.927729 -0.486430 2.296461

C -2.559654 -2.191318 3.824488

C -0.656949 -0.699113 3.655830

C -2.015259 -1.152004 1.707465

C -2.830142 -1.997700 2.465270

C -1.470482 -1.545950 4.414804

H 0.196922 -0.212815 4.119217

H -2.220745 -1.028822 0.647780

H -3.654053 -2.507866 1.970054

H -1.245942 -1.706349 5.467006

H -3.184071 -2.858185 4.414829

C 1.650556 0.907372 2.128361

C 2.634709 -0.087021 1.950994

C 1.961895 2.090592 2.814563

C 3.923420 0.149903 2.452461

C 3.244957 2.299574 3.321596

H 1.201493 2.853980 2.944290

C 4.230874 1.330008 3.126301

H 4.686221 -0.608977 2.312600

H 3.473928 3.218985 3.855068

H 5.238993 1.491319 3.501051

C 2.757415 -0.312369 -1.044292

C 3.715603 -1.077377 -1.768814

C 4.206347 1.690111 -1.426604

C 5.073884 0.902125 -2.183064

H 5.951777 1.334026 -2.656791

C 2.278513 -1.397471 1.328551

C 2.450197 -2.518846 2.166896

C 2.097967 -3.781382 1.721209

C 1.320054 -2.849165 -0.442232

C 1.536772 -3.900677 0.452458

H 2.219181 -4.663980 2.337989

H 1.237062 -4.899693 0.170059

O 2.944428 -2.282292 3.433352

C 3.025130 -3.373340 4.324686

H 3.397887 -2.964587 5.267594

H 2.042639 -3.836128 4.493339

H 3.722251 -4.146122 3.969253

C 1.724303 -1.519062 0.006896

C 0.510158 -3.252965 -1.731097

C -0.978683 -2.872822 -1.514610

C 0.995810 -2.664574 -3.076639

C 0.514052 -4.789602 -1.966927

H -1.364376 -3.302903 -0.583448

H -1.114601 -1.789849 -1.465023

H -1.588532 -3.259844 -2.341924

H 1.898874 -3.173657 -3.423348

H 0.212328 -2.834802 -3.826742

H 1.174750 -1.593796 -3.057506

H 0.043887 -4.986798 -2.936899

H 1.530855 -5.199871 -2.003206

H -0.061458 -5.348306 -1.220234

Rh 0.544929 0.045044 -0.919326

O -1.247108 0.766407 -1.702229

C -0.830173 0.633482 -2.985780

O 0.425211 0.310054 -3.067602

C 3.101530 1.048732 -0.843300

H 2.450654 1.656831 -0.237509

C 4.843366 -0.461161 -2.324991

H 5.558472 -1.054628 -2.882601

O 3.561913 -2.433073 -1.848809

C 4.548486 -3.211978 -2.485490

H 4.221717 -4.248783 -2.375376

H 4.635994 -2.975283 -3.555418

H 5.533476 -3.092417 -2.011715

C 4.476929 3.198605 -1.267382

C 5.820539 3.403916 -0.527504

C 4.556229 3.855908 -2.666297

C 3.376125 3.923644 -0.470325

H 5.781877 2.956991 0.472649

H 6.654530 2.942446 -1.068501

H 6.043200 4.473891 -0.416941

H 3.613555 3.724806 -3.209242

H 4.753478 4.932400 -2.574630

H 5.354809 3.421975 -3.277877

H 3.606136 4.995264 -0.417739

H 2.395924 3.817096 -0.947705

H 3.298576 3.550208 0.555840

O -1.619551 0.775259 -3.931678

Br -5.262793 -1.940047 -0.611682

K -5.049123 0.484441 1.337855

O -5.653149 2.596901 -0.062655

C -4.737179 2.421000 -0.895555

O -4.691303 2.574090 -2.131423

O -3.561955 1.854524 -0.289850

K -3.889743 0.096977 -2.763420

H -2.816663 1.811350 -0.924607

**INT14A**

P 0.913717 1.749565 -0.440100

C 2.044524 0.991795 -1.734136

C 3.323152 -0.147174 -3.989167

C 1.464864 1.033729 -3.020258

C 3.275258 0.312013 -1.584463

C 3.897332 -0.227780 -2.725645

C 2.086291 0.481151 -4.135224

H 0.500105 1.517475 -3.145531

H 4.847458 -0.736727 -2.592482

H 1.605664 0.543051 -5.108524

H 3.830069 -0.581359 -4.847715

C 0.628909 3.438084 -1.177646

C 0.091794 6.016226 -2.165026

C 1.548305 4.088137 -2.017070

C -0.562197 4.098592 -0.832667

C -0.822763 5.380562 -1.322324

C 1.279093 5.367264 -2.510359

H 2.474628 3.591998 -2.292193

H -1.273424 3.603662 -0.175496

H -1.748977 5.878971 -1.046709

H 1.997929 5.854879 -3.165579

H -0.119158 7.010938 -2.551483

C 1.868662 2.218544 1.088148

C 1.691964 1.421325 2.228852

C 2.612849 3.402533 1.206955

C 2.277463 1.773616 3.445981

H 1.051058 0.546755 2.158107

C 3.187702 3.762466 2.427303

H 2.727297 4.059165 0.351479

C 3.028387 2.945175 3.548714

H 2.122469 1.145089 4.319420

H 3.744275 4.694200 2.506766

H 3.468139 3.232089 4.501270

C 4.866955 1.137931 0.150601

C 3.941861 -1.108918 0.423460

C 5.604492 0.970139 1.315480

C 4.644659 -1.270288 1.655123

C 5.462353 -0.205895 2.050241

H 6.267767 1.745997 1.679744

H 6.027593 -0.272991 2.970476

O 4.929862 2.250563 -0.646434

C 5.843759 3.270348 -0.289860

H 5.755435 4.036092 -1.063909

H 5.598156 3.710396 0.684868

H 6.877155 2.896092 -0.267023

C 4.005966 0.112607 -0.285126

C 4.500691 -2.497629 2.600183

C 5.370640 -2.347831 3.870992

C 3.034630 -2.622159 3.083468

C 4.937349 -3.825219 1.934748

H 6.440945 -2.294749 3.638012

H 5.100557 -1.464033 4.459653

H 5.216822 -3.224697 4.510024

H 2.347769 -2.823435 2.259760

H 2.944023 -3.445666 3.803860

H 2.709989 -1.700149 3.579566

H 4.956619 -4.623033 2.688616

H 4.252547 -4.137262 1.146081

H 5.940364 -3.737334 1.503368

Rh -0.990189 0.652116 0.055748

O -1.768545 2.022075 1.359159

C -2.874274 1.329143 1.567472

O -2.884960 0.186919 0.879273

C 3.141084 -2.199873 -0.237191

C 3.775504 -3.143155 -1.075303

C 1.750375 -2.226482 -0.159166

C 3.015685 -4.101226 -1.745465

C 0.951310 -3.146098 -0.864711

H 1.265843 -1.469993 0.450953

C 1.619177 -4.094483 -1.639722

H 3.491684 -4.843796 -2.376578

H 1.060883 -4.840412 -2.195462

C -0.578745 -3.011468 -0.807287

C -1.076264 -3.056629 0.656359

C -0.961352 -1.654685 -1.454495

C -1.300644 -4.123180 -1.593945

H -0.772829 -3.998770 1.131054

H -0.672815 -2.226011 1.244718

H -2.168563 -2.980924 0.694531

H -0.688998 -1.628115 -2.516050

H -2.029999 -1.453184 -1.361317

H -0.332711 -0.849603 -0.995581

H -2.383806 -3.980878 -1.521738

H -1.031070 -4.118469 -2.657455

H -1.068780 -5.114724 -1.185934

O 5.142374 -3.037510 -1.183165

C 5.822637 -3.988414 -1.976983

H 5.501542 -3.949045 -3.027420

H 6.882226 -3.729136 -1.915801

H 5.678625 -5.011000 -1.600711

O -3.792420 1.678360 2.322740

Br -8.311219 0.259891 0.777384

K -7.059916 -0.802404 -1.819477

O -5.724545 -2.953150 -1.273878

C -4.988853 -2.362381 -0.452926

O -4.614409 -2.677188 0.697251

O -4.584284 -1.066263 -0.915306

K -5.408916 -0.420194 2.020661

H -3.949384 -0.659817 -0.274316

**2j**

Br -1.963163 -0.039762 0.000001

C -0.120074 -0.529085 -0.000001

C 0.866452 0.475548 -0.000081

C 0.229246 -1.874634 0.000072

C 2.213181 0.087416 -0.000111

C 1.575298 -2.250147 0.000074

H -0.555526 -2.623472 0.000138

C 2.560278 -1.265726 -0.000025

H 2.994336 0.839051 -0.000233

H 1.842513 -3.302464 0.000142

H 3.610891 -1.542434 -0.000046

O 0.438053 1.763725 -0.000233

C 1.407368 2.799983 0.000211

H 2.041194 2.759455 -0.895229

H 0.841293 3.732840 0.000522

H 2.041086 2.758771 0.895690

**INT1A-2j**

P -1.842302 -0.095412 -0.037095

C -1.934240 1.160622 1.339071

C -1.976153 2.896703 3.560237

C -1.473068 0.733299 2.597274

C -2.408151 2.473427 1.213373

C -2.428031 3.335304 2.315261

C -1.499063 1.589161 3.698301

H -1.074371 -0.273700 2.693792

H -2.754881 2.834224 0.250365

H -2.794315 4.352771 2.195112

H -1.137173 1.238335 4.661818

H -1.989983 3.568015 4.416021

C -2.298416 0.900603 -1.552460

C -2.862905 2.340964 -3.908201

C -3.583237 1.418059 -1.789413

C -1.310379 1.100299 -2.526981

C -1.586998 1.818345 -3.692637

C -3.862537 2.135350 -2.954129

H -4.375808 1.245264 -1.067203

H -0.328546 0.665357 -2.365145

H -0.805212 1.961702 -4.434775

H -4.864484 2.525298 -3.120602

H -3.081904 2.895239 -4.818268

C -3.446783 -1.004825 0.242998

C -3.609513 -2.260392 -0.367055

C -4.503351 -0.485915 1.010202

C -4.809652 -2.960611 -0.227752

H -2.774683 -2.710951 -0.893047

C -5.700816 -1.191937 1.148874

H -4.394803 0.472430 1.508976

C -5.859414 -2.430294 0.524648

H -4.912947 -3.936135 -0.696772

H -6.506257 -0.773503 1.749169

H -6.789672 -2.983631 0.635592

Rh 0.020710 -1.401795 -0.093782

P 1.756945 0.082115 -0.020152

C 2.724772 -0.047886 1.573103

C 3.408634 1.011776 2.187957

C 2.782739 -1.324634 2.162793

C 4.122295 0.809011 3.373125

H 3.382210 2.005263 1.750995

C 3.505965 -1.523237 3.339835

H 2.270698 -2.155339 1.679070

C 4.172142 -0.458703 3.953233

H 4.639560 1.645293 3.839380

H 3.544226 -2.517058 3.779751

H 4.727309 -0.616745 4.875545

C 3.097618 -0.353813 -1.244091

C 2.834477 -1.323078 -2.221694

C 4.369217 0.242413 -1.208486

C 3.814882 -1.671449 -3.155696

H 1.872480 -1.824891 -2.214884

C 5.345990 -0.103252 -2.142062

H 4.599480 0.979528 -0.443900

C 5.068459 -1.060852 -3.122351

H 3.596906 -2.432385 -3.901128

H 6.325282 0.369084 -2.101034

H 5.831267 -1.335341 -3.847985

C 1.637472 1.924956 -0.274384

C 1.993497 2.530186 -1.491610

C 1.072737 2.738566 0.723857

C 1.808311 3.898842 -1.697977

H 2.426720 1.929647 -2.284819

C 0.897546 4.108506 0.522109

H 0.766386 2.301128 1.668750

C 1.265338 4.696145 -0.689742

H 2.093966 4.340944 -2.649989

H 0.460387 4.711142 1.314078

H 1.124298 5.762816 -0.848512

C 0.294414 -3.908495 -0.119193

O -0.878684 -3.273148 -0.121519

O 1.311783 -3.036319 -0.096911

O 0.428083 -5.125850 -0.138253

**TS2A-2j**

P 1.364060 1.590647 -0.062064

C 1.644384 1.743013 1.772260

C 1.921334 1.788529 4.580629

C 0.519359 1.853742 2.608462

C 2.908332 1.639333 2.372875

C 3.045108 1.658786 3.764087

C 0.655796 1.887391 3.996270

H -0.473603 1.909951 2.172105

H 3.793832 1.542141 1.753452

H 4.035521 1.575285 4.206900

H -0.232572 1.976349 4.615821

H 2.028475 1.805546 5.663013

C 3.097559 1.777037 -0.732521

C 5.696308 1.944567 -1.795996

C 3.840976 2.963527 -0.623005

C 3.670112 0.682274 -1.393709

C 4.962235 0.764197 -1.919582

C 5.131506 3.046630 -1.147470

H 3.405627 3.830295 -0.132878

H 3.072499 -0.219896 -1.503701

H 5.389558 -0.093528 -2.433624

H 5.693865 3.973661 -1.056727

H 6.700113 2.011238 -2.210302

C 0.676907 3.263073 -0.510090

C 0.192027 3.407210 -1.823426

C 0.623116 4.365236 0.355868

C -0.324334 4.630341 -2.252242

H 0.218362 2.552792 -2.497039

C 0.094416 5.585679 -0.076101

H 0.986821 4.273574 1.374940

C -0.378563 5.722524 -1.381305

H -0.694214 4.726841 -3.270424

H 0.054419 6.428383 0.611254

H -0.790149 6.671862 -1.717392

C -0.096807 -0.826440 -3.324153

O 0.321739 0.370751 -2.893494

O -0.345673 -1.637267 -2.300729

Br 0.813488 -1.931562 1.304764

O -0.218765 -1.127278 -4.508189

C 2.477637 -2.836301 1.011154

C 3.506738 -2.715994 1.936800

C 2.639519 -3.586465 -0.172065

H 3.351232 -2.115180 2.827086

C 3.880114 -4.200006 -0.402802

C 4.914654 -4.077572 0.528151

H 4.026582 -4.779843 -1.308198

H 5.866894 -4.563668 0.329793

O 1.579176 -3.666263 -0.993382

C 1.770406 -3.943202 -2.379930

H 0.878208 -3.549833 -2.867901

H 2.652663 -3.416632 -2.762773

H 1.884575 -5.022931 -2.551654

C -3.199714 -2.030808 0.242935

C -4.297115 -4.623228 0.144222

C -4.225232 -2.410826 1.124671

C -2.727579 -2.971488 -0.692268

C -3.281055 -4.252605 -0.739568

C -4.766189 -3.697804 1.078459

H -4.603419 -1.703901 1.857359

H -1.930547 -2.692636 -1.380997

H -2.907198 -4.966101 -1.470325

H -5.559039 -3.973358 1.771335

H -4.719194 -5.625391 0.107329

C -2.964808 0.415025 1.776709

C -3.499521 1.654508 4.268632

C -2.672998 -0.233379 2.994726

C -3.506009 1.712497 1.843858

C -3.766091 2.323799 3.072977

C -2.949686 0.368900 4.220755

H -2.227555 -1.225144 2.980450

H -3.731946 2.247935 0.927194

H -4.186314 3.327086 3.092015

H -2.728048 -0.164892 5.142352

H -3.711409 2.127638 5.224555

C -3.489768 0.487934 -1.061767

C -2.897722 1.075572 -2.187666

C -4.890172 0.514412 -0.938915

C -3.692026 1.686578 -3.164267

H -1.818637 1.033596 -2.312392

C -5.678807 1.131282 -1.908845

H -5.366767 0.044354 -0.082851

C -5.078684 1.721236 -3.026153

H -3.216271 2.125195 -4.037563

H -6.761228 1.144234 -1.798408

H -5.693761 2.195743 -3.787957

P -2.394292 -0.351547 0.188168

Rh 0.283085 -0.201453 -0.896108

C 4.734713 -3.342677 1.699144

H 5.541186 -3.242976 2.420298

**INT2A-2j**

P 1.394620 0.223888 0.058429

C 1.154668 2.054755 -0.190054

C 0.561008 4.787160 -0.590920

C 1.838700 2.807390 -1.155826

C 0.151614 2.695964 0.559459

C -0.134545 4.047793 0.370855

C 1.543088 4.159343 -1.356648

H 2.611098 2.337539 -1.756230

H -0.410841 2.126430 1.294871

H -0.906401 4.522875 0.972449

H 2.087451 4.721035 -2.112885

H 0.334583 5.840043 -0.743429

C 1.974474 0.165057 1.831791

C 2.832062 -0.096143 4.499607

C 2.490930 1.268121 2.530163

C 1.889488 -1.075865 2.488856

C 2.322792 -1.200117 3.810454

C 2.913290 1.139785 3.856449

H 2.553503 2.237988 2.044672

H 1.481651 -1.932953 1.953621

H 2.253490 -2.166344 4.305015

H 3.304029 2.007339 4.384815

H 3.159886 -0.197481 5.532267

C 3.001328 -0.087872 -0.840321

C 2.968360 -0.965987 -1.933053

C 4.230877 0.479911 -0.468805

C 4.133049 -1.256878 -2.648425

H 2.018694 -1.429137 -2.191979

C 5.394083 0.192586 -1.184593

H 4.283414 1.142345 0.391014

C 5.346959 -0.676214 -2.278664

H 4.090540 -1.946150 -3.488516

H 6.339205 0.639920 -0.883563

H 6.255178 -0.905573 -2.832165

Rh -0.238718 -1.231356 -0.435137

C -0.333335 -3.704820 -0.039816

O 0.718132 -2.900069 0.220147

O -1.312972 -3.021551 -0.612457

Br -1.864094 0.405940 -1.601078

O -0.353663 -4.898906 0.237929

C -3.318052 0.910319 -0.426045

C -3.565468 2.249126 -0.154350

C -4.084896 -0.113439 0.164640

C -4.583913 2.600043 0.739818

H -2.945520 3.009752 -0.618009

C -5.089115 0.250619 1.074689

C -5.333084 1.597524 1.354900

H -4.775010 3.647171 0.958980

H -5.682959 -0.524192 1.549380

H -6.119263 1.859084 2.059316

O -3.802629 -1.371695 -0.206466

C -4.061328 -2.461596 0.676797

H -3.371045 -3.240285 0.348206

H -3.835234 -2.180508 1.712442

H -5.109282 -2.785728 0.603220

**TS3A-2j**

P -1.318257 -0.006615 0.008513

C -1.856299 1.751836 -0.268813

C -2.453278 4.458121 -0.809952

C -2.137541 2.650321 0.772044

C -1.863000 2.242935 -1.587459

C -2.168918 3.576003 -1.856527

C -2.430409 3.991242 0.504000

H -2.133897 2.301722 1.799954

H -1.625528 1.570500 -2.407289

H -2.174684 3.929939 -2.884881

H -2.644817 4.668466 1.327921

H -2.683648 5.500308 -1.018527

C -2.658026 -0.968092 -0.861701

C -4.578226 -2.574689 -2.145798

C -3.880471 -0.423324 -1.283074

C -2.406980 -2.333562 -1.091131

C -3.365797 -3.126885 -1.722552

C -4.832548 -1.220720 -1.925652

H -4.091413 0.629994 -1.122641

H -1.457057 -2.759477 -0.771004

H -3.158153 -4.180868 -1.891699

H -5.771919 -0.779816 -2.253465

H -5.317992 -3.195345 -2.647114

C -1.789302 -0.301315 1.785193

C -0.772096 -0.646228 2.686129

C -3.111988 -0.225885 2.251781

C -1.070420 -0.891558 4.029544

H 0.243596 -0.742797 2.315050

C -3.408609 -0.467318 3.593449

H -3.915897 0.012891 1.560465

C -2.385397 -0.799240 4.486545

H -0.271693 -1.165574 4.714364

H -4.438021 -0.405755 3.940246

H -2.616758 -0.995206 5.531432

C 1.702940 -3.025488 -0.203117

O 0.518758 -2.580664 0.263845

O 2.310818 -2.082076 -0.903408

Rh 0.755732 -0.730146 -0.558815

O 2.137688 -4.150524 0.017572

Br 1.727599 1.412690 -1.752462

C 2.353528 1.102848 0.132882

C 3.648652 0.556744 0.301922

C 1.758279 1.844969 1.160720

C 4.218184 0.597151 1.578852

C 2.357373 1.893651 2.419837

H 0.808055 2.334315 0.979607

C 3.576076 1.248865 2.637038

H 5.184900 0.130326 1.739840

H 1.860876 2.427039 3.226783

H 4.045446 1.270447 3.617385

O 4.282559 0.063801 -0.791346

C 5.127504 -1.071081 -0.624940

H 5.405767 -1.376338 -1.635600

H 6.040720 -0.819320 -0.064895

H 4.574125 -1.883911 -0.149402

**INT3A-2j**

P -1.026136 0.142102 0.160344

C -1.183066 1.983331 0.308642

C -1.454350 4.778211 0.453319

C -1.774305 2.586359 1.429916

C -0.724335 2.798551 -0.740241

C -0.864934 4.184265 -0.665242

C -1.906794 3.975788 1.501048

H -2.130253 1.975810 2.252436

H -0.241519 2.340696 -1.600216

H -0.500094 4.800697 -1.483015

H -2.363175 4.426623 2.379210

H -1.555254 5.859753 0.510286

C -2.394115 -0.302022 -0.998899

C -4.496050 -1.051954 -2.697782

C -3.458545 0.579991 -1.244905

C -2.387410 -1.565715 -1.612359

C -3.439436 -1.931443 -2.454213

C -4.503863 0.205194 -2.091139

H -3.473403 1.562092 -0.783620

H -1.555989 -2.244014 -1.425889

H -3.422922 -2.909527 -2.928352

H -5.319556 0.899849 -2.278491

H -5.308409 -1.342105 -3.360689

C -1.608523 -0.454407 1.806446

C -0.707994 -0.434181 2.884319

C -2.922631 -0.892223 2.020721

C -1.127768 -0.823735 4.155274

H 0.319393 -0.132208 2.718675

C -3.335145 -1.292445 3.294135

H -3.624964 -0.926800 1.194430

C -2.440066 -1.255390 4.363298

H -0.419933 -0.810092 4.979922

H -4.355265 -1.637328 3.445041

H -2.759621 -1.571716 5.353622

C 0.585206 -3.035566 0.534571

O 1.090019 -1.960604 1.191621

O 0.244011 -2.712487 -0.699378

Rh 0.975667 -0.681455 -0.392094

O 0.494882 -4.138555 1.059058

Br 0.906252 0.349553 -2.763966

C 2.221777 0.755679 0.344398

C 3.470818 0.159155 0.129594

C 2.215951 1.978392 1.019209

C 4.678081 0.711267 0.538551

C 3.417939 2.563526 1.451939

H 1.282449 2.494982 1.222892

C 4.641729 1.938433 1.214906

H 5.622476 0.213282 0.333029

H 3.391376 3.516694 1.977763

H 5.569373 2.399040 1.547943

O 3.369985 -1.044071 -0.589747

C 3.955500 -2.203229 0.018029

H 3.734595 -3.036028 -0.652182

H 5.042215 -2.082732 0.106613

H 3.499486 -2.386605 0.994824

**TS4A-2j**

P -1.188732 0.004937 0.210327

C -2.018363 1.649213 0.133984

C -3.339072 4.121864 -0.056849

C -2.680431 2.192681 1.247309

C -2.018307 2.364267 -1.076578

C -2.679267 3.588881 -1.166974

C -3.335000 3.422598 1.150463

H -2.686825 1.657913 2.190778

H -1.472148 1.973600 -1.930692

H -2.666857 4.132100 -2.108428

H -3.840949 3.830984 2.022059

H -3.848165 5.080124 -0.131001

C -1.978952 -1.134998 -0.953134

C -2.788148 -2.914732 -2.894023

C -3.283325 -1.037217 -1.460173

C -1.033965 -2.088941 -1.389810

C -1.489832 -2.977445 -2.381701

C -3.690749 -1.947041 -2.434520

H -3.959973 -0.257051 -1.116661

H 0.195100 -2.586870 -0.803233

H -0.809483 -3.739727 -2.760127

H -4.698500 -1.897449 -2.841738

H -3.105657 -3.619308 -3.662000

C -1.512204 -0.574485 1.922053

C -0.625444 -0.247658 2.958666

C -2.661303 -1.325526 2.206400

C -0.904789 -0.646309 4.265706

H 0.289105 0.290233 2.736185

C -2.932700 -1.727204 3.515827

H -3.335261 -1.607764 1.402743

C -2.057009 -1.384365 4.547205

H -0.207240 -0.398613 5.061432

H -3.822964 -2.315172 3.725569

H -2.263978 -1.703521 5.566030

C 1.534175 -2.788559 0.780369

O 1.274012 -1.501060 1.079667

O 1.117063 -3.219553 -0.395324

Rh 0.892789 -0.308222 -0.529890

O 2.161236 -3.475425 1.586241

Br 0.816195 0.971394 -2.738056

C 1.926648 1.134599 0.369275

C 3.289701 0.830628 0.267101

C 1.547475 2.288573 1.052802

C 4.267082 1.631060 0.854728

C 2.520052 3.106651 1.649000

H 0.499629 2.567386 1.125559

C 3.871559 2.775987 1.556787

H 5.319336 1.376589 0.757143

H 2.213628 4.003608 2.183592

H 4.624449 3.410539 2.019142

O 3.574345 -0.277742 -0.518462

C 4.386727 -1.297442 0.078241

H 4.452829 -2.095328 -0.663692

H 5.394694 -0.918689 0.293541

H 3.917247 -1.686717 0.986011

**INT4A-2j**

P -1.332012 0.287783 0.285181

C -2.823444 -0.677218 -0.194808

C -5.111858 -2.181659 -0.794348

C -3.984539 -0.045172 -0.667706

C -2.810740 -2.074108 -0.032296

C -3.955224 -2.813893 -0.331970

C -5.123550 -0.796509 -0.963816

H -4.005456 1.030658 -0.807059

H -1.895345 -2.565276 0.292434

H -3.934090 -3.893841 -0.210409

H -6.016999 -0.295995 -1.330089

H -5.998880 -2.766544 -1.027748

C -1.030989 0.148752 2.069246

C 0.250210 -0.452416 4.424088

C -1.742257 0.301168 3.261903

C 0.295003 -0.325101 2.017196

C 0.941982 -0.625141 3.220353

C -1.076820 0.003035 4.453452

H -2.779972 0.628916 3.270699

H 1.963172 -0.996962 3.224314

H -1.589896 0.114551 5.405993

H 0.748161 -0.687119 5.363707

C -1.702787 1.999155 -0.248047

C -1.398268 2.362761 -1.570216

C -2.277433 2.940535 0.617045

C -1.691265 3.650708 -2.020097

H -0.899881 1.647421 -2.221728

C -2.561795 4.230023 0.161730

H -2.487751 2.671393 1.648289

C -2.274422 4.583815 -1.158498

H -1.447232 3.929371 -3.041929

H -3.002021 4.957450 0.839486

H -2.493767 5.588680 -1.511811

Br 1.081389 -0.449921 -2.635334

C 1.824338 1.277522 0.216324

C 3.125974 0.771360 0.320261

C 1.685600 2.664694 0.239524

C 4.268942 1.553791 0.402713

H 0.705968 3.126562 0.167804

C 4.099655 2.946206 0.407067

H 5.258202 1.108174 0.467182

H 4.970771 3.594492 0.471903

O 3.115042 -0.633754 0.364281

C 4.046613 -1.328317 -0.481288

H 3.694053 -2.358505 -0.532994

H 4.043401 -0.888921 -1.483488

H 5.051911 -1.284143 -0.043865

Rh 0.782765 -0.459632 0.055591

O 0.106803 -2.549666 0.129452

C 0.808057 -3.598036 -0.182821

O 0.769591 -4.687684 0.374121

O 1.667997 -3.449615 -1.257486

H 1.457498 -2.598477 -1.701321

C 2.818574 3.491533 0.333693

H 2.692478 4.572893 0.342945

**INT5A-2j**

P 0.606008 1.437528 0.463250

C -1.132525 1.984977 0.456271

C -3.867300 2.587924 0.416428

C -1.785510 2.349693 1.640459

C -1.853847 1.944359 -0.751169

C -3.218434 2.225608 -0.772888

C -3.148114 2.669398 1.611527

H -1.247491 2.356967 2.583408

H -1.354362 1.635318 -1.667335

H -3.812684 2.027825 -1.665304

H -3.648598 2.955293 2.535494

H -4.940786 2.738117 0.387967

C 1.183770 1.136828 2.155564

C 2.025204 -0.276758 4.362223

C 1.363772 1.830888 3.355284

C 1.410574 -0.248662 2.031303

C 1.838687 -0.961710 3.156334

C 1.795741 1.103218 4.467383

H 1.174767 2.899268 3.434778

H 2.017079 -2.031298 3.095857

H 1.951687 1.606979 5.418312

H 2.355776 -0.825390 5.242735

C 1.546487 2.719831 -0.443752

C 1.736318 2.571426 -1.826661

C 2.039721 3.859912 0.207655

C 2.399056 3.567491 -2.546362

H 1.389399 1.668793 -2.325608

C 2.708910 4.846774 -0.517054

H 1.908756 3.975858 1.279816

C 2.884669 4.703663 -1.895596

H 2.544899 3.446139 -3.616388

H 3.092962 5.725533 -0.004944

H 3.405287 5.473894 -2.459397

C 3.006166 -0.707495 -0.257280

C 3.319339 -2.070584 -0.215547

C 4.041276 0.180503 -0.541945

C 4.580075 -2.586625 -0.480054

H 3.861315 1.250015 -0.585174

C 5.601773 -1.674545 -0.781894

H 4.775182 -3.655282 -0.447529

H 6.603786 -2.040625 -0.993078

O 2.205577 -2.827263 0.183143

C 1.877082 -3.994668 -0.579455

H 0.946790 -4.368310 -0.149391

H 1.730173 -3.731592 -1.631785

H 2.663078 -4.752793 -0.476329

Rh 1.023978 -0.754495 0.109477

C -1.479028 -2.007626 1.522661

O -2.516047 -1.960629 2.209402

Br 0.553769 -1.082259 -2.553865

K -2.601451 -1.263048 -1.957662

O -4.694453 0.237549 -2.397329

C -5.205102 -0.215142 -1.295756

O -5.891560 0.539050 -0.497815

O -4.922883 -1.453997 -0.922350

K -4.708381 -0.659881 1.451443

O -1.140587 -1.252220 0.557951

O -0.572693 -3.015591 1.815064

H -0.967838 -3.476031 2.574051

C 5.333695 -0.306257 -0.803285

H 6.133003 0.395667 -1.032791

**TS6A-2j**

P 2.044318 0.923404 0.292677

C 1.870100 2.713849 -0.111587

C 1.645559 5.448823 -0.687238

C 3.019384 3.517585 -0.211043

C 0.604719 3.288584 -0.306461

C 0.503568 4.652205 -0.594369

C 2.905823 4.879087 -0.491319

H 4.005141 3.080670 -0.081597

H -0.283111 2.669758 -0.203642

H -0.480247 5.088991 -0.747614

H 3.802064 5.490658 -0.564981

H 1.557029 6.509064 -0.913809

C 2.093771 0.600559 2.076967

C 1.411411 -0.462173 4.517898

C 2.759026 1.076413 3.209649

C 1.073977 -0.370427 2.135445

C 0.735180 -0.913286 3.378825

C 2.409211 0.522307 4.443429

H 3.515362 1.855950 3.142775

H -0.052155 -1.656829 3.462094

H 2.905846 0.858655 5.350685

H 1.149603 -0.874845 5.490816

C 3.624039 0.488379 -0.526133

C 3.623347 0.327495 -1.921773

C 4.814583 0.315321 0.191600

C 4.808995 0.016935 -2.586414

H 2.688951 0.407378 -2.472502

C 5.999787 0.004814 -0.480861

H 4.811075 0.405237 1.274106

C 5.998819 -0.140426 -1.869100

H 4.800278 -0.116940 -3.664934

H 6.919678 -0.131307 0.082520

H 6.920333 -0.387614 -2.390677

C 1.363590 -2.285974 -0.007991

C 0.358553 -3.237363 0.230302

C 2.623314 -2.763059 -0.358010

C 0.546734 -4.607730 0.095587

H 3.441147 -2.080545 -0.560010

C 1.824882 -5.059351 -0.265991

H -0.264553 -5.308900 0.266813

H 2.005350 -6.126636 -0.374652

O -0.822692 -2.621168 0.622449

C -2.067052 -3.255230 0.283835

H -2.865160 -2.524634 0.418942

H -2.039317 -3.592479 -0.758754

H -2.232363 -4.115408 0.945006

Rh 0.338590 -0.555932 0.266984

O -1.088270 1.092701 0.737085

C -2.345386 0.889293 0.777417

O -3.206343 1.657024 0.271879

O -2.728972 -0.244738 1.410486

H -3.669966 -0.477151 1.191537

Br -0.349519 -0.439720 -2.390966

K -3.524765 -0.102263 -2.017393

O -5.958092 0.214604 -1.034217

C -5.561986 -0.530760 -0.024468

O -5.925262 -0.237480 1.181398

O -4.693999 -1.476085 -0.231746

K -5.724538 2.164527 0.596536

C 2.852661 -4.145659 -0.483478

H 3.843010 -4.500137 -0.763477

**INT6A-2j**

P 2.125249 -0.550726 -0.055045

C 2.295214 -2.026511 -1.144729

C 2.488456 -4.282483 -2.800994

C 3.440962 -2.236496 -1.927418

C 1.235330 -2.947065 -1.212215

C 1.342480 -4.073101 -2.031073

C 3.535095 -3.359044 -2.751778

H 4.253725 -1.516991 -1.903205

H 0.322704 -2.799994 -0.634946

H 0.519919 -4.783057 -2.062842

H 4.426772 -3.510086 -3.355672

H 2.564189 -5.157797 -3.441921

C 3.268428 0.746540 -0.609891

C 4.220825 3.075417 -1.732255

C 4.654470 0.926371 -0.695198

C 2.337369 1.702567 -1.077721

C 2.838329 2.886133 -1.640776

C 5.126316 2.111333 -1.264174

H 5.350140 0.174552 -0.327503

H 2.154361 3.653169 -1.988816

H 6.196582 2.288839 -1.342017

H 4.607769 3.993948 -2.170848

C 2.523964 -1.158963 1.634219

C 1.704748 -2.138106 2.224857

C 3.593910 -0.623726 2.365946

C 1.977536 -2.587049 3.517499

H 0.837291 -2.535258 1.700128

C 3.859850 -1.078743 3.659308

H 4.212412 0.154101 1.929351

C 3.056283 -2.063974 4.235416

H 1.335574 -3.342571 3.962720

H 4.694011 -0.658485 4.215988

H 3.263450 -2.416430 5.242985

C 0.184221 1.943456 0.862156

C 0.001016 3.343041 0.881230

C 0.028966 1.251937 2.075712

C -0.356779 3.991997 2.072972

H 0.190745 0.178931 2.102722

C -0.536545 3.283383 3.259352

H -0.475267 5.072510 2.055583

H -0.809604 3.807223 4.172136

O 0.230945 4.104635 -0.240638

C -0.909088 4.767742 -0.791287

H -0.535553 5.363685 -1.627923

H -1.627996 4.034733 -1.165361

H -1.382053 5.436251 -0.058304

Rh 0.464249 0.936331 -0.839248

O -0.870023 1.985318 -2.054273

C -1.848781 1.113070 -1.902122

O -2.925579 1.141579 -2.529829

Br -1.973369 -3.040564 0.837102

K -2.806192 0.050891 1.493362

O -5.497917 -0.094073 1.275092

C -5.338231 0.134459 0.059198

O -5.651783 -0.542312 -0.953673

O -4.586176 1.313621 -0.192849

K -3.428031 -1.616261 -1.730900

O -1.562809 0.162406 -1.016893

H -4.339784 1.324281 -1.142814

C -0.335240 1.902649 3.262269

H -0.439038 1.327830 4.180509

**TS7A-2j**

P 2.449689 0.368874 0.003727

C 2.631115 2.194208 0.154909

C 2.745689 4.985832 0.393690

C 3.862673 2.861405 0.069419

C 1.458693 2.940043 0.361856

C 1.516234 4.328206 0.484096

C 3.916316 4.251939 0.188574

H 4.778625 2.301235 -0.091411

H 0.493068 2.449990 0.439214

H 0.589227 4.873858 0.633590

H 4.875584 4.760202 0.117806

H 2.792295 6.069202 0.479651

C 1.912310 -0.268575 1.622001

C 0.326294 -1.014904 3.763702

C 2.285045 0.101290 2.914766

C 0.713783 -1.001107 1.349853

C -0.073550 -1.356312 2.470255

C 1.502460 -0.294626 4.001668

H 3.158799 0.731276 3.068138

H -0.981564 -1.933493 2.331918

H 1.789033 -0.026436 5.015030

H -0.299010 -1.314488 4.602768

C 4.153963 -0.212995 -0.394728

C 4.577706 -0.159118 -1.733202

C 5.028434 -0.727363 0.573985

C 5.854571 -0.592959 -2.089724

H 3.900218 0.217889 -2.495556

C 6.303566 -1.170144 0.213439

H 4.706497 -0.793521 1.609308

C 6.720615 -1.099932 -1.116929

H 6.169474 -0.544766 -3.129093

H 6.969888 -1.570538 0.973789

H 7.712873 -1.445415 -1.396538

C 0.684351 -2.366035 0.049302

C -0.470896 -3.207465 0.024873

C 1.929055 -3.006421 -0.114143

C -0.350882 -4.574213 -0.249500

H 2.834389 -2.416671 -0.042099

C 0.901782 -5.157391 -0.455448

H -1.239857 -5.192424 -0.300400

H 0.970288 -6.222973 -0.660718

O -1.676888 -2.627934 0.295362

C -2.870736 -3.336605 -0.032503

H -3.696023 -2.639551 0.131530

H -2.853676 -3.650866 -1.082905

H -2.999603 -4.217859 0.611679

Rh 0.412848 -0.428673 -0.715654

O 0.029485 0.735545 -2.335987

C -1.257776 0.433129 -2.409065

O -1.646906 -0.364334 -1.420440

C 2.047995 -4.371248 -0.374105

H 3.033494 -4.808859 -0.513552

O -2.020102 0.853594 -3.292730

Br -5.519891 -0.629118 0.572064

K -2.476721 0.379029 1.102891

O -1.708026 2.908978 1.422774

C -2.326565 3.353148 0.434886

O -3.160104 2.758329 -0.310070

O -2.056876 4.678571 0.101227

K -4.190451 0.967707 -1.827163

H -2.573226 4.829833 -0.707361

**INT7A-2j**

P -1.081031 0.457205 -0.492280

C -1.115575 0.590947 1.366212

C -0.595223 0.782456 4.137953

C -0.066783 1.391249 1.865371

C -1.912068 -0.127873 2.285299

C -1.635069 -0.012533 3.659632

C 0.197410 1.488304 3.231009

H 0.595600 1.925963 1.186400

H -2.259175 -0.564963 4.357720

H 1.033634 2.099154 3.559709

H -0.403394 0.843240 5.206323

C -1.163486 2.241855 -0.967438

C -1.268798 4.909247 -1.829272

C -1.969163 3.174673 -0.293859

C -0.405061 2.661454 -2.068355

C -0.458566 3.989810 -2.497057

C -2.024117 4.500248 -0.726509

H -2.545340 2.862168 0.573297

H 0.231350 1.937365 -2.571293

H 0.142570 4.306956 -3.345354

H -2.648628 5.216755 -0.197821

H -1.304970 5.944838 -2.159035

C -2.629941 -0.243143 -1.224214

C -2.558382 -1.617547 -1.528940

C -3.710036 0.500961 -1.723348

C -3.547525 -2.234133 -2.297871

H -1.725701 -2.212338 -1.132950

C -4.706626 -0.121478 -2.476871

H -3.760739 1.568850 -1.537013

C -4.630006 -1.487811 -2.765021

H -3.468764 -3.293122 -2.528317

H -5.540037 0.466005 -2.856357

H -5.404991 -1.963500 -3.361048

C -4.308879 -0.575263 1.580416

C -2.832744 -2.439339 1.983928

C -5.352940 -1.468454 1.317628

H -1.845557 -2.810524 2.246018

C -5.127232 -2.846175 1.386086

H -6.339102 -1.103128 1.055535

H -5.946911 -3.528596 1.175384

O -4.447522 0.786267 1.549635

C -5.720443 1.324147 1.241387

H -5.604439 2.409187 1.282411

H -6.047850 1.031880 0.235368

H -6.478315 1.013709 1.974040

C -3.024079 -1.056565 1.910662

Rh 0.260150 -0.981634 -1.574941

C -3.869291 -3.339130 1.719809

H -3.689531 -4.408959 1.774764

O 1.595967 -2.202361 -2.642536

C 2.615126 -1.578765 -2.135853

O 3.820567 -1.799012 -2.354950

Br 3.505780 2.285334 1.067741

K 2.372337 -0.757190 1.467875

O 4.659224 -1.897094 2.373848

C 4.984419 -2.053739 1.180296

O 5.960084 -1.614203 0.521411

O 4.015588 -2.781526 0.432106

K 4.933411 0.286239 -0.956079

O 2.239510 -0.598586 -1.285969

H 4.270636 -2.732381 -0.511136

**INT8A-2j**

P 2.482496 -0.345087 0.796137

C 3.585019 1.071346 0.298957

C 5.165542 3.315853 -0.341400

C 4.906052 0.915626 -0.148256

C 3.071500 2.371828 0.421869

C 3.854422 3.484431 0.110196

C 5.688508 2.028180 -0.470006

H 5.330220 -0.079478 -0.239570

H 2.050930 2.509496 0.767061

H 3.432548 4.480971 0.211194

H 6.709415 1.885485 -0.817146

H 5.774931 4.181571 -0.590233

C 2.761537 -0.353136 2.643263

C 3.056006 -0.400495 5.443743

C 3.823447 0.323139 3.264979

C 1.845004 -1.055825 3.446504

C 1.999759 -1.081706 4.833727

C 3.966978 0.303067 4.655036

H 4.544475 0.870326 2.666046

H 1.009767 -1.571150 2.979045

H 1.281732 -1.629588 5.439292

H 4.792437 0.839310 5.118251

H 3.165964 -0.414816 6.525820

C 3.494168 -1.823928 0.246200

C 3.467063 -2.288382 -1.091271

C 4.295971 -2.510367 1.174586

C 4.243656 -3.404424 -1.440127

C 5.055841 -3.622090 0.811386

H 4.327043 -2.171570 2.203699

C 5.032300 -4.072730 -0.506218

H 4.210616 -3.752427 -2.469148

H 5.660031 -4.129892 1.559449

H 5.617023 -4.938916 -0.806866

Rh 0.253863 -0.300365 0.391096

Br 0.027026 1.475545 -1.458529

C -0.134827 3.376180 -1.082388

C 0.786673 4.239433 -1.662907

C -1.162456 3.852175 -0.246562

C 0.715275 5.612775 -1.410166

H 1.567397 3.831532 -2.296376

C -1.210379 5.231554 0.014605

C -0.281304 6.097677 -0.565283

H 1.436182 6.286338 -1.864730

H -1.980426 5.629341 0.664855

H -0.346099 7.161263 -0.350353

O -2.045020 2.953281 0.244342

C -2.939733 3.373464 1.283511

H -3.534749 2.497299 1.546287

H -2.370847 3.735316 2.148541

H -3.614733 4.159906 0.924327

C 2.662391 -1.667295 -2.192480

C 1.397842 -2.210461 -2.521748

C 3.179146 -0.639910 -2.983905

C 0.675251 -1.695450 -3.606953

C 2.465282 -0.127766 -4.072658

H 4.155038 -0.231182 -2.737202

C 1.213954 -0.659294 -4.374820

H -0.300089 -2.098958 -3.853898

H 2.885715 0.675590 -4.671530

H 0.642415 -0.272639 -5.215509

O 0.983400 -3.242560 -1.751252

C -0.360144 -3.699386 -1.885161

H -0.519078 -4.389963 -1.056378

H -1.075041 -2.876132 -1.811220

H -0.503902 -4.226002 -2.839339

O -1.812927 -0.723775 0.515419

C -1.561737 -1.868453 1.115108

O -2.445367 -2.705311 1.405254

Br -5.701563 0.922119 1.344965

K -3.606468 0.285555 -1.094951

O -5.212337 -1.579492 -2.384913

C -4.829831 -2.404875 -1.536009

O -5.460865 -3.104049 -0.703348

O -3.407610 -2.499299 -1.452889

K -4.991196 -2.109925 1.640981

O -0.277232 -2.050608 1.352217

H -3.191167 -2.973510 -0.626337

**INT9A-2j**

P 1.141770 0.862532 0.249076

C 0.863562 2.061983 -1.155406

C 0.278033 3.910391 -3.207589

C 1.829669 2.406236 -2.112321

C -0.402916 2.664405 -1.246112

C -0.691083 3.584080 -2.254781

C 1.538592 3.317068 -3.133120

H 2.820989 1.966727 -2.057576

H -1.164816 2.407524 -0.515459

H -1.676145 4.043543 -2.297282

H 2.303166 3.566104 -3.866093

H 0.051984 4.621266 -3.999284

C 1.439630 2.076619 1.641831

C 1.815290 3.811385 3.830219

C 1.744870 3.432922 1.440481

C 1.322689 1.599083 2.960318

C 1.517232 2.462532 4.040507

C 1.926929 4.294703 2.525788

H 1.840489 3.824943 0.432496

H 1.072763 0.552288 3.126834

H 1.425400 2.075939 5.053017

H 2.156061 5.343693 2.347997

H 1.956419 4.481432 4.675979

C 2.892425 0.272387 -0.111437

C 3.145734 -0.822993 -0.974512

C 3.990752 0.892932 0.509016

C 4.470479 -1.238850 -1.181116

C 5.300861 0.464032 0.296233

H 3.819054 1.727053 1.179663

C 5.545160 -0.610028 -0.556157

H 4.647144 -2.082662 -1.843200

H 6.122374 0.968849 0.799814

H 6.559785 -0.960768 -0.731511

Rh -0.351604 -0.723157 0.792115

C -0.368217 -2.266389 2.780476

O 0.574515 -1.351576 2.498134

O -1.289210 -2.305315 1.827504

Br -1.913683 -0.584481 -1.273091

O -0.347449 -2.972039 3.785638

C -3.534287 0.376199 -0.839794

C -3.912630 1.479257 -1.594419

C -4.302023 -0.055611 0.260280

C -5.061505 2.200492 -1.248351

H -3.296848 1.785172 -2.434329

C -5.439907 0.687365 0.609643

C -5.812087 1.804892 -0.141977

H -5.353812 3.067816 -1.834209

H -6.037031 0.377794 1.461548

H -6.700322 2.363979 0.142747

O -3.890271 -1.169839 0.884027

C -4.212321 -1.390350 2.255121

H -3.455403 -2.097490 2.596845

H -4.130346 -0.457680 2.826272

H -5.227850 -1.798592 2.360246

C 2.087578 -1.578431 -1.720563

C 1.594323 -2.801927 -1.208265

C 1.638988 -1.145038 -2.969731

C 0.652247 -3.536238 -1.941070

C 0.698574 -1.875562 -3.703845

H 2.019234 -0.206960 -3.364107

C 0.207069 -3.069013 -3.179761

H 0.254429 -4.460611 -1.538184

H 0.353912 -1.508918 -4.667053

H -0.531233 -3.647100 -3.730927

O 2.112161 -3.208586 -0.024911

C 1.388173 -4.175149 0.737076

H 1.826954 -4.155992 1.734676

H 0.332304 -3.898146 0.813833

H 1.496587 -5.179721 0.300999

**TS10A-2j**

P -0.511653 0.682099 0.020587

C -0.464324 2.558840 0.141258

C -0.348069 5.383677 0.094213

C -1.233305 3.329935 1.028809

C 0.359552 3.236624 -0.773941

C 0.417642 4.631396 -0.798710

C -1.173372 4.726021 1.007407

H -1.881930 2.840248 1.747363

H 0.970273 2.663583 -1.464669

H 1.067735 5.128457 -1.515086

H -1.777402 5.298914 1.707995

H -0.300234 6.470187 0.080022

C -1.806667 0.591243 -1.326088

C -3.574119 0.483787 -3.513415

C -2.916561 1.448507 -1.392575

C -1.595234 -0.316800 -2.373157

C -2.473325 -0.371904 -3.458023

C -3.794365 1.393884 -2.476010

H -3.094114 2.170447 -0.601660

H -0.730015 -0.974403 -2.318994

H -2.293701 -1.084491 -4.259535

H -4.645145 2.071443 -2.515765

H -4.255205 0.446370 -4.361082

C -1.345010 0.246132 1.629988

C -2.458834 -0.599479 1.819647

C -0.699095 0.766224 2.769257

C -2.940134 -0.800279 3.127488

C -1.175828 0.540617 4.055708

H 0.204035 1.355709 2.639982

C -2.323141 -0.233875 4.236402

H -3.804974 -1.444548 3.260699

H -0.648798 0.959380 4.909652

H -2.714900 -0.420456 5.233688

C 1.537244 -3.058434 0.519277

O 0.596705 -2.181335 0.915194

O 2.315283 -2.515488 -0.402548

Rh 1.278980 -0.708335 -0.320423

O 1.642548 -4.194587 0.969332

Br 2.846377 0.686169 -1.949758

C 3.450476 0.513843 -0.026019

C 4.500615 -0.402799 0.217981

C 3.208196 1.569712 0.859994

C 5.143086 -0.349070 1.459778

C 3.880599 1.617181 2.081933

H 2.461749 2.314273 0.611781

C 4.830761 0.643688 2.392898

H 5.910843 -1.081695 1.687564

H 3.648975 2.411261 2.787682

H 5.350622 0.664766 3.347382

O 4.858614 -1.250929 -0.777809

C 5.304958 -2.555824 -0.420382

H 5.383501 -3.103667 -1.361653

H 6.295168 -2.528719 0.058829

H 4.563579 -3.045290 0.215262

C -3.111797 -1.418276 0.748850

C -4.442161 -1.178365 0.346981

C -2.436985 -2.523450 0.218905

C -5.074585 -2.032374 -0.561709

C -3.067316 -3.380112 -0.689307

H -1.403569 -2.691274 0.521132

C -4.382360 -3.135207 -1.072804

H -6.094178 -1.845599 -0.880519

H -2.523422 -4.232055 -1.087891

H -4.883969 -3.794764 -1.777904

O -5.044987 -0.070741 0.895216

C -6.334451 0.278157 0.440636

H -6.603228 1.196052 0.969870

H -6.344013 0.467549 -0.641779

H -7.078064 -0.498661 0.672678

**INT10A-2j**

P -0.223212 0.520900 0.048437

C 0.092038 2.333777 0.292985

C 0.483815 5.110047 0.510039

C -0.576720 3.074933 1.281396

C 0.953443 3.002156 -0.591945

C 1.144770 4.380370 -0.479994

C -0.379259 4.453656 1.388977

H -1.256137 2.579458 1.966334

H 1.471795 2.436811 -1.362694

H 1.820959 4.880487 -1.168797

H -0.902119 5.011504 2.162480

H 0.640181 6.183018 0.596987

C -1.523871 0.559884 -1.266047

C -3.382650 0.657085 -3.367167

C -2.281580 1.720364 -1.493968

C -1.701675 -0.551692 -2.104715

C -2.632311 -0.497592 -3.143055

C -3.205996 1.766468 -2.538635

H -2.146602 2.596331 -0.870184

H -1.115131 -1.448868 -1.929312

H -2.760383 -1.365595 -3.784304

H -3.774288 2.677864 -2.712766

H -4.096881 0.696194 -4.186975

C -0.922792 0.021293 1.694657

C -2.157728 -0.597963 1.973557

C -0.038940 0.284988 2.760329

C -2.483203 -0.865974 3.317230

C -0.378493 0.009791 4.078537

H 0.942054 0.697076 2.546513

C -1.619497 -0.561636 4.362230

H -3.437335 -1.342677 3.524348

H 0.330345 0.223830 4.874122

H -1.903026 -0.790340 5.386904

C 0.370878 -2.912435 0.373563

O 1.142770 -2.080899 1.102205

O 0.254847 -2.493194 -0.870307

Rh 1.540778 -0.783157 -0.427092

O -0.152052 -3.918363 0.855590

Br 1.982226 0.299893 -2.727816

C 3.086329 0.213780 0.455293

C 4.120476 -0.718502 0.302844

C 3.387911 1.372690 1.174226

C 5.400096 -0.555397 0.817786

C 4.669976 1.568961 1.715130

H 2.636298 2.142327 1.325586

C 5.671275 0.614508 1.540681

H 6.170484 -1.305911 0.658386

H 4.882415 2.478602 2.274701

H 6.663458 0.774230 1.957307

O 3.729811 -1.823034 -0.474540

C 3.903256 -3.115888 0.121058

H 3.502826 -3.832620 -0.598444

H 4.967683 -3.321390 0.288275

H 3.339676 -3.178342 1.056024

C -3.156264 -1.083433 0.966031

C -4.262877 -0.289039 0.604120

C -3.074158 -2.392403 0.479237

C -5.256875 -0.799010 -0.236324

C -4.067263 -2.904477 -0.361945

H -2.218822 -3.009973 0.747956

C -5.152922 -2.108992 -0.714444

H -6.103730 -0.188018 -0.527726

H -3.978184 -3.919355 -0.738804

H -5.929991 -2.495903 -1.370156

O -4.284173 0.979216 1.130212

C -5.334866 1.841597 0.744473

H -5.146552 2.791247 1.251326

H -5.343322 2.005056 -0.341266

H -6.315985 1.455957 1.058177

**TS11A-2j**

P -1.158827 0.497720 0.339049

C -2.938204 0.035760 0.206524

C -5.626212 -0.685334 -0.109591

C -3.747665 -0.182799 1.329629

C -3.481113 -0.108301 -1.082133

C -4.821041 -0.464967 -1.229910

C -5.087663 -0.542482 1.169375

H -3.340964 -0.077708 2.329376

H -2.855446 0.056693 -1.958886

H -5.228131 -0.584114 -2.230373

H -5.705451 -0.714257 2.047871

H -6.668563 -0.971263 -0.233111

C -0.938134 0.986809 2.119758

C -0.623104 1.675194 4.833738

C -0.862355 2.332318 2.515111

C -0.834365 -0.010486 3.105304

C -0.689286 0.334498 4.449167

C -0.703829 2.671590 3.861155

H -0.920953 3.123812 1.777632

H -0.847179 -1.055429 2.809827

H -0.611644 -0.453183 5.194407

H -0.643166 3.719923 4.144151

H -0.500287 1.940575 5.881360

C -1.083583 2.129876 -0.526686

C 0.102815 2.614585 -1.122021

C -2.220659 2.955088 -0.480092

C 0.108878 3.935311 -1.612441

C -2.196492 4.252176 -0.983835

H -3.133530 2.574977 -0.034355

C -1.018184 4.745798 -1.546074

H 1.013815 4.310895 -2.076360

H -3.090115 4.869294 -0.936672

H -0.981546 5.755035 -1.949375

Rh 0.224298 -1.054884 -0.488555

Br -0.701201 -2.873181 1.027940

C 1.863447 -0.861407 0.771522

C 2.725510 -1.922208 0.447244

C 2.313882 0.021472 1.758265

C 3.944453 -2.146493 1.084117

C 3.542438 -0.170011 2.411200

H 1.707240 0.875824 2.044578

C 4.351498 -1.257910 2.086124

H 4.576116 -2.984324 0.798109

H 3.856465 0.529513 3.184747

H 5.299560 -1.415187 2.596805

O 2.296736 -2.689795 -0.637565

C 2.289564 -4.107731 -0.488242

H 3.311952 -4.507464 -0.438202

H 1.722890 -4.396677 0.402164

H 1.789894 -4.499052 -1.376651

C 1.291747 1.763464 -1.387954

C 2.606952 2.220106 -1.173049

C 3.694725 1.459494 -1.619148

C 2.198485 -0.230638 -2.472092

C 3.489331 0.247696 -2.284193

H 4.708154 1.805407 -1.447963

H 2.023472 -1.178607 -2.969909

H 4.348170 -0.320890 -2.631582

O 2.755473 3.415905 -0.508576

C 4.037544 3.745575 -0.009761

H 3.902171 4.646878 0.593422

H 4.440393 2.941272 0.619674

H 4.752416 3.963358 -0.816814

C 1.089524 0.500423 -1.999361

H 0.047590 0.307504 -2.499142

O -1.224519 -0.008023 -3.323281

C -1.283114 -1.309637 -3.208450

O -1.648726 -2.075419 -4.114117

O -0.943591 -1.824981 -2.014283

**INT11A-2j**

P -0.377840 1.161136 0.544685

C -1.316504 2.671828 0.031709

C -2.689626 4.943673 -0.887873

C -2.225677 3.340701 0.865885

C -1.090149 3.160792 -1.269419

C -1.776220 4.290078 -1.718262

C -2.908868 4.469124 0.405967

H -2.405307 2.987389 1.875426

H -0.355253 2.662757 -1.901225

H -1.590577 4.658654 -2.723914

H -3.612314 4.975158 1.063285

H -3.224799 5.820617 -1.245256

C -1.056510 0.753205 2.210204

C -2.102437 0.139538 4.747232

C -0.226723 0.742250 3.340462

C -2.417611 0.440381 2.365068

C -2.935876 0.142432 3.625675

C -0.746476 0.434902 4.599806

H 0.828024 0.974180 3.234924

H -3.072181 0.420680 1.500012

H -3.991195 -0.097880 3.726304

H -0.088227 0.430337 5.465321

H -2.508151 -0.094742 5.728796

C 1.329805 1.729374 0.828696

C 2.382006 0.795400 0.696834

C 1.599364 3.091446 1.018929

C 3.690731 1.311306 0.668211

C 2.907779 3.564352 1.044507

H 0.775392 3.791401 1.115972

C 3.953386 2.666443 0.836914

H 4.514830 0.631312 0.495529

H 3.102956 4.624882 1.181877

H 4.979874 3.021477 0.789014

Rh -0.306392 -0.340010 -1.184946

Br -0.404501 -1.718108 -3.394798

C -1.936993 -1.382094 -0.477981

C -2.101184 -2.216586 0.650914

C -3.087905 -1.151165 -1.248164

C -3.340099 -2.797656 0.960138

H -3.003937 -0.538135 -2.143826

C -4.461143 -2.536172 0.166530

H -3.443907 -3.442197 1.827122

H -5.416999 -2.990559 0.419917

O -0.996453 -2.400997 1.445003

C -1.073636 -3.283482 2.538879

H -1.780217 -2.930030 3.304328

H -1.364783 -4.298313 2.228497

H -0.068245 -3.315812 2.964869

C 2.174277 -0.682477 0.569650

C 3.060378 -1.546542 1.283870

C 3.052006 -2.925931 1.097468

C 2.132285 -3.483150 0.204849

H 3.746355 -3.565733 1.629391

H 2.134629 -4.557291 0.030614

O 3.902587 -0.943283 2.197841

C 4.791801 -1.753457 2.931229

H 5.351345 -1.074369 3.580276

H 4.260871 -2.489624 3.552894

H 5.496684 -2.289155 2.278942

C 1.179284 -1.292010 -0.229025

C 2.157436 1.099777 -2.720730

O 0.992291 1.127059 -2.128180

O 2.930999 2.045039 -2.802225

O 2.512078 -0.091988 -3.296937

H 1.723555 -0.673968 -3.292656

C 1.195386 -2.684393 -0.430268

H 0.458592 -3.131297 -1.083868

C -4.340171 -1.700222 -0.938578

H -5.198942 -1.489178 -1.572315

**TS11B-2j**

P -0.461508 0.714473 -0.075794

C -1.784513 0.693928 -1.355702

C -3.612259 1.065902 -3.454564

C -3.163348 0.502096 -1.119686

C -1.339541 1.012462 -2.650027

C -2.245798 1.205130 -3.690617

C -4.056395 0.706352 -2.184301

H -0.274498 1.098455 -2.840995

H -1.876225 1.445715 -4.683730

H -5.116499 0.550279 -2.002636

H -4.329982 1.212048 -4.258531

C -0.702860 -0.293451 1.429163

C -0.329670 -1.801260 3.741828

C -1.351444 0.081352 2.613087

C 0.320562 -2.164537 2.563315

C -1.174350 -0.684791 3.764367

H -1.988391 0.959580 2.638775

H 0.955357 -3.046696 2.535456

H -1.693017 -0.409924 4.679736

H -0.188046 -2.389278 4.646561

C -0.427626 2.475157 0.476404

C -0.901145 3.507116 -0.349041

C 0.111821 2.812047 1.729407

C -0.843743 4.836977 0.071068

H -1.315621 3.273976 -1.323255

C 0.160936 4.141249 2.149485

H 0.494594 2.033414 2.378898

C -0.316656 5.159303 1.321864

H -1.214318 5.620849 -0.584799

H 0.578370 4.379242 3.124700

H -0.275102 6.195488 1.648785

Rh 1.427136 -0.400363 -0.526864

Br 2.344987 1.094535 -2.283079

C 2.947466 0.274093 0.716271

C 3.872159 -0.776011 0.606889

C 3.302112 1.331179 1.552773

C 5.061253 -0.848661 1.323985

C 4.499362 1.299618 2.290235

H 2.657234 2.200811 1.643544

C 5.367539 0.213997 2.186811

H 5.740457 -1.689643 1.207660

H 4.750968 2.132069 2.945670

H 6.291112 0.191196 2.761663

O 3.459060 -1.709635 -0.339445

C 3.690584 -3.102230 -0.114815

H 2.849535 -3.619665 -0.579395

H 4.648468 -3.402567 -0.559202

H 3.704363 -3.317884 0.960871

C -3.756103 0.023001 0.167030

C -3.691934 -1.359572 0.478105

C -4.475357 0.871274 1.009316

C -4.328262 -1.845656 1.627972

C -5.107674 0.389721 2.162185

H -4.534197 1.927276 0.755872

C -5.027099 -0.968534 2.462252

H -4.273900 -2.900383 1.872536

H -5.656454 1.068531 2.809618

H -5.513432 -1.360245 3.353080

O -3.008975 -2.115807 -0.403106

C -2.816526 -3.511323 -0.167335

H -3.782526 -4.036629 -0.157511

H -2.187291 -3.849684 -0.994390

H -2.293217 -3.675497 0.781698

C 0.137454 -1.427429 1.382568

H 0.438828 -2.091370 0.413091

O 0.593113 -3.220714 -0.534430

C 0.068338 -2.768189 -1.650233

O 0.164949 -1.446980 -1.863342

O -0.519807 -3.483212 -2.479751

**INT11B-2j**

P 0.564546 0.704139 0.138930

C 1.720738 0.291431 1.519962

C 3.217311 0.035943 3.883863

C 3.124600 0.160225 1.444437

C 1.087324 0.239917 2.772780

C 1.827670 0.114429 3.946646

C 3.849321 0.047778 2.642164

H 0.001446 0.270145 2.826946

H 1.311431 0.068031 4.901859

H 4.928969 -0.062096 2.580914

H 3.808185 -0.058374 4.792085

C 0.919629 0.175064 -1.563056

C 0.488087 -1.004839 -4.013662

C 1.869893 0.374103 -2.567458

C -0.461730 -1.187075 -3.003428

C 1.637580 -0.227813 -3.806748

H 2.770024 0.957806 -2.400515

H -1.352605 -1.781746 -3.187077

H 2.357370 -0.099040 -4.611705

H 0.331232 -1.470793 -4.985530

C 0.453897 2.534605 0.265102

C -0.491630 3.093839 1.139677

C 1.310566 3.376686 -0.457788

C -0.562777 4.479031 1.295135

H -1.181255 2.443633 1.673692

C 1.230694 4.761964 -0.301579

H 2.035732 2.953614 -1.146428

C 0.296883 5.314416 0.577595

H -1.300953 4.904726 1.970004

H 1.895990 5.408144 -0.869336

H 0.234394 6.393531 0.697429

Rh -1.324799 -0.501922 -0.055317

Br -2.617201 -0.164248 2.317914

C -2.617241 0.763007 -0.976246

C -3.653675 -0.119589 -1.303977

C -2.786226 2.098374 -1.339997

C -4.839528 0.257840 -1.918309

H -2.015567 2.832656 -1.123622

C -4.990591 1.611590 -2.254432

H -5.620602 -0.465245 -2.139151

H -5.904980 1.946886 -2.739053

O -3.310923 -1.441319 -0.968095

C -4.292202 -2.230737 -0.279476

H -3.753104 -3.087088 0.127231

H -4.734278 -1.651992 0.537202

H -5.067537 -2.562871 -0.981500

C 3.909827 0.051221 0.177085

C 3.882597 -1.170605 -0.542628

C 4.760159 1.070287 -0.255057

C 4.698298 -1.332539 -1.671403

H 4.783782 2.000439 0.308063

C 5.531564 -0.290887 -2.087355

H 4.675478 -2.264803 -2.224374

H 6.155915 -0.430622 -2.967233

O 3.053665 -2.109482 -0.051144

C 2.711219 -3.252910 -0.837299

H 3.571470 -3.932649 -0.926414

H 1.881757 -3.733831 -0.310814

H 2.381915 -2.942823 -1.836207

C -0.250371 -0.584879 -1.758806

C -0.561855 -3.333834 0.944392

O -0.100832 -2.153546 0.699434

O -0.021437 -4.403697 0.662836

O -1.780215 -3.388813 1.600883

H -2.003951 -2.475702 1.885308

C 5.566698 0.915643 -1.389793

H 6.215853 1.724622 -1.714193

C -3.968367 2.517957 -1.974488

H -4.088539 3.565506 -2.245689

**TS11C-2j**

P -0.423971 0.609961 0.256957

C -1.346971 -0.254007 1.599214

C -2.678848 -1.563509 3.700785

C -2.729762 -0.081439 1.787088

C -0.636807 -1.066221 2.496422

C -1.302824 -1.720347 3.535847

C -3.389483 -0.736211 2.827626

H -3.294018 0.573226 1.134192

H 0.437663 -1.158944 2.396330

H -0.734745 -2.343209 4.221805

H -4.459642 -0.592644 2.954311

H -3.194470 -2.070098 4.513802

C -1.019078 2.340430 0.317320

C -1.815285 5.027852 0.397054

C -1.468431 2.911272 1.515850

C -0.933810 3.140118 -0.833699

C -1.343427 4.471121 -0.794322

C -1.869468 4.248456 1.552440

H -1.500836 2.319614 2.424420

H -0.529509 2.730318 -1.751462

H -1.263720 5.080191 -1.690420

H -2.215962 4.678942 2.488841

H -2.123015 6.070522 0.427287

C -0.736877 -0.126908 -1.409621

C -1.897237 -0.716926 -1.957715

C -1.757627 -1.499272 -3.115015

C 0.614026 -1.058119 -3.203262

C -0.516157 -1.684668 -3.722664

H -2.645473 -1.954957 -3.544777

H 1.577853 -1.144771 -3.697015

H -0.442272 -2.299843 -4.617054

Rh 1.761913 0.276794 0.023743

Br 2.405108 0.814140 2.360350

C 2.096762 -1.762313 0.310552

C 3.447100 -1.893056 -0.055145

C 1.411204 -2.947487 0.580884

C 4.114203 -3.110869 -0.148462

C 2.051733 -4.197447 0.500615

H 0.354099 -2.925886 0.837190

C 3.395134 -4.279551 0.141694

H 5.162091 -3.157286 -0.434341

H 1.490468 -5.106229 0.713060

H 3.891191 -5.246140 0.079518

O 4.026578 -0.660230 -0.322351

C 4.977935 -0.549553 -1.379820

H 4.759160 -1.275967 -2.171851

H 4.864922 0.457684 -1.780236

H 5.993879 -0.718993 -0.997942

C -3.275096 -0.481842 -1.435741

C -4.075652 -1.543659 -0.953780

C -3.829368 0.802191 -1.467836

C -5.383118 -1.302579 -0.516533

C -5.133967 1.050583 -1.029698

H -3.217335 1.620233 -1.834718

C -5.906443 -0.006432 -0.555868

H -5.994770 -2.113713 -0.137820

H -5.535144 2.059460 -1.062130

H -6.923495 0.166344 -0.212051

O -3.487712 -2.775412 -0.936438

C -4.171624 -3.844045 -0.309826

H -3.492274 -4.697581 -0.352928

H -4.399458 -3.613491 0.739196

H -5.103527 -4.097690 -0.835313

C 0.517542 -0.267450 -2.051999

H 1.497772 0.458931 -2.012317

O 2.679539 1.303819 -2.385850

C 2.465979 2.433370 -1.740309

O 1.829995 2.305167 -0.558266

O 2.787817 3.553281 -2.150231

**INT11C-2j**

P -0.449901 0.377407 0.463613

C -0.961504 -0.727209 1.830650

C -1.656216 -2.365199 3.992951

C -2.023172 -1.633817 1.713389

C -0.229957 -0.661791 3.029318

C -0.584641 -1.474463 4.105643

C -2.367925 -2.448965 2.795057

H -2.564913 -1.721994 0.777472

H 0.636199 -0.006401 3.095709

H -0.010717 -1.423882 5.027306

H -3.191052 -3.152765 2.696946

H -1.925882 -3.002247 4.832171

C -1.108998 2.027150 0.946635

C -2.159443 4.547625 1.584354

C -2.036598 2.165879 1.991163

C -0.701367 3.163134 0.225943

C -1.232747 4.413336 0.547713

C -2.557988 3.421643 2.306800

H -2.358050 1.298509 2.557498

H 0.040937 3.053111 -0.563400

H -0.907926 5.286824 -0.011889

H -3.275219 3.516328 3.118813

H -2.564767 5.526137 1.832651

C -1.032780 -0.195877 -1.165276

C -2.240355 -0.392907 -1.860842

C -2.129920 -0.918512 -3.157662

C 0.308080 -0.963715 -3.028696

C -0.880978 -1.196627 -3.724802

H -3.035032 -1.096521 -3.732067

H 1.269438 -1.167822 -3.491872

H -0.841810 -1.591456 -4.738877

Rh 1.619969 0.102223 -0.368538

Br 3.120091 0.938042 1.726124

C 1.978675 -1.846427 0.083141

C 3.154717 -2.057584 -0.647095

C 1.491176 -2.929852 0.814528

C 3.866772 -3.248940 -0.661953

H 0.584408 -2.840322 1.403616

C 3.359946 -4.316415 0.092616

H 4.780184 -3.354191 -1.241744

H 3.888443 -5.267153 0.105357

O 3.503496 -0.918480 -1.395946

C 4.854856 -0.444416 -1.261221

H 5.123912 -0.389298 -0.202074

H 5.540137 -1.107607 -1.803606

H 4.865905 0.556911 -1.691643

C -3.579554 -0.020634 -1.316725

C -4.589961 -0.988178 -1.104753

C -3.885912 1.316550 -1.042398

C -5.855115 -0.609802 -0.643132

H -3.117845 2.065104 -1.207306

C -6.128290 0.736651 -0.381798

H -6.627747 -1.352465 -0.478208

H -7.115240 1.018184 -0.022353

O -4.234365 -2.288720 -1.353819

C -5.201665 -3.302306 -1.167750

H -4.704952 -4.239951 -1.425835

H -5.547648 -3.351794 -0.125555

H -6.072042 -3.160639 -1.823998

C 0.234462 -0.454999 -1.729926

C 2.583033 2.739403 -1.813839

O 1.566690 2.076470 -1.345320

O 2.587480 3.441443 -2.816734

O 3.758712 2.636442 -1.091762

H 3.559533 2.191749 -0.238495

C 2.179861 -4.154792 0.817751

H 1.787218 -4.987005 1.399746

C -5.145880 1.702341 -0.575615

H -5.348519 2.749104 -0.369050

**INT12A-2j**

P 1.365184 1.194868 0.118307

C 1.042191 2.806591 -0.750471

C 0.662999 5.203902 -2.169755

C 0.999898 4.034840 -0.077119

C 0.888963 2.791894 -2.146698

C 0.703336 3.982446 -2.846809

C 0.810165 5.225719 -0.783779

H 1.108732 4.071886 1.001160

H 0.882753 1.848943 -2.681997

H 0.577674 3.952582 -3.926264

H 0.774098 6.168920 -0.243731

H 0.511741 6.130152 -2.718946

C 1.387440 1.705074 1.889834

C 1.316451 2.562849 4.561454

C 2.575130 1.859413 2.616589

C 0.160161 1.979856 2.513623

C 0.129823 2.410069 3.841026

C 2.538853 2.285496 3.946215

H 3.529419 1.641673 2.147330

H -0.761235 1.866306 1.948335

H -0.825834 2.622220 4.314484

H 3.467887 2.400259 4.499746

H 1.288713 2.894955 5.596484

C 3.093261 0.880996 -0.421804

C 3.518196 -0.376889 -0.917279

C 3.935975 2.004860 -0.537414

C 4.734946 -0.386088 -1.640852

C 5.168821 1.938210 -1.172692

H 3.594954 2.960963 -0.153574

C 5.546608 0.732413 -1.765733

H 5.045864 -1.308685 -2.111910

H 5.797899 2.821629 -1.244895

H 6.471656 0.662878 -2.333612

Rh -0.061693 -0.374795 -0.646016

C -0.496528 -1.160741 1.180158

C 0.393029 -1.491272 2.224599

C -1.834096 -1.538933 1.339044

C -0.067702 -2.112448 3.394886

H -2.557460 -1.349031 0.554213

C -1.416074 -2.454931 3.531183

H 0.623821 -2.342564 4.198947

H -1.754445 -2.948857 4.439750

O 1.719978 -1.184932 2.036978

C 2.674288 -1.668173 2.956365

H 2.592062 -1.172226 3.934383

H 2.583943 -2.754073 3.094763

H 3.650072 -1.443762 2.519029

C 2.785911 -1.672162 -0.767386

C 3.561597 -2.858262 -0.571889

C 2.986323 -4.126759 -0.598103

C 1.608314 -4.240979 -0.780593

H 3.590977 -5.016578 -0.469046

H 1.148588 -5.226340 -0.821228

O 4.907069 -2.691958 -0.295030

C 5.689851 -3.833495 -0.023827

H 6.695236 -3.462500 0.192541

H 5.315410 -4.390779 0.846696

H 5.737517 -4.517178 -0.883780

C 1.378328 -1.808923 -0.803217

O -0.119288 -0.125277 -3.019876

C -1.080212 -0.932121 -2.992414

O -1.474771 -1.559741 -1.968288

O -1.804561 -1.146477 -4.136558

H -1.419382 -0.551999 -4.802972

Br -2.158444 1.429399 -0.502142

K -4.337447 0.572204 1.852345

O -5.009548 -1.399901 0.456202

C -6.127240 -0.741039 0.128941

O -6.595297 -0.870947 -1.062951

O -6.638782 0.064781 1.000729

K -4.231143 -1.202014 -1.942103

C -2.304088 -2.181106 2.494197

H -3.353852 -2.462467 2.534821

C 0.819589 -3.101943 -0.853165

H -0.255094 -3.210712 -0.926579

**TS13A-2j**

P 0.910154 -0.812470 0.934761

C -0.537515 -1.532406 1.841504

C -2.834324 -2.546672 3.100194

C -0.656550 -2.906299 2.097446

C -1.569078 -0.668649 2.248660

C -2.720384 -1.173091 2.856892

C -1.796178 -3.405913 2.733569

H 0.123615 -3.594201 1.792820

H -1.503776 0.394146 2.049541

H -3.540276 -0.494947 3.078781

H -1.874799 -4.473785 2.925515

H -3.732602 -2.941673 3.569251

C 2.068058 -2.242317 0.792200

C 3.736779 -4.488190 0.585034

C 3.155857 -2.399782 1.661462

C 1.823138 -3.217027 -0.188282

C 2.654710 -4.334378 -0.284181

C 3.986003 -3.517752 1.557410

H 3.362011 -1.646238 2.414344

H 0.982144 -3.097826 -0.866608

H 2.456183 -5.081648 -1.048105

H 4.828437 -3.626819 2.236489

H 4.384101 -5.358298 0.503533

C 1.597777 0.369140 2.155442

C 2.010835 1.690417 1.840210

C 1.534387 -0.052748 3.498523

C 2.226867 2.550396 2.943642

C 1.837700 0.797695 4.553364

H 1.202877 -1.062572 3.718483

C 2.153091 2.124408 4.262522

H 2.453508 3.588683 2.751567

H 1.779171 0.443391 5.578955

H 2.326750 2.838411 5.064216

Rh 0.231008 0.207630 -0.906641

C 1.687790 -0.542747 -2.140422

C 3.087144 -0.542341 -1.934922

C 1.245262 -0.882601 -3.426988

C 3.978478 -0.878287 -2.961811

H 0.177686 -0.918666 -3.619551

C 3.496534 -1.205752 -4.232579

H 5.048631 -0.881738 -2.782592

H 4.198757 -1.463620 -5.022707

O 3.525852 -0.190350 -0.681658

C 4.908989 -0.045995 -0.449326

H 5.440820 -1.005359 -0.526624

H 5.362037 0.673322 -1.145722

H 5.003282 0.336955 0.569353

C 2.157182 2.256413 0.463934

C 3.089896 3.314343 0.222313

C 3.101772 4.024101 -0.978610

C 2.215318 3.668945 -1.996349

H 3.793358 4.843627 -1.131893

H 2.200421 4.235842 -2.924710

O 4.019537 3.576745 1.209946

C 5.006477 4.557297 0.974455

H 5.641897 4.561250 1.863993

H 5.619012 4.318484 0.093459

H 4.571873 5.558189 0.840312

C 1.396039 1.822603 -0.638899

O -1.235254 1.472684 0.124830

C -2.507885 1.554724 0.017771

O -3.191115 1.305434 -1.005747

O -3.102739 1.951859 1.168623

H -4.082733 1.775553 1.152119

Br -1.408907 -1.884804 -1.608684

K -4.213001 -1.228364 -0.200928

O -6.390314 0.182512 -0.559033

C -6.024152 0.881818 0.495960

O -6.156161 2.170000 0.493087

O -5.401008 0.293448 1.470648

K -5.481978 2.157980 -1.899773

C 1.396124 2.561421 -1.832463

H 0.763514 2.246608 -2.661581

C 2.127075 -1.200728 -4.469774

H 1.733100 -1.456905 -5.450918

**INT13A-2j**

P -1.201511 -0.046158 0.944440

C -0.065618 -1.004060 2.031477

C 1.835617 -2.264490 3.660713

C -0.150816 -0.904106 3.431275

C 0.978736 -1.745521 1.456035

C 1.935565 -2.359745 2.271183

C 0.785906 -1.547454 4.240604

H -0.935955 -0.312827 3.892922

H 1.046543 -1.836362 0.375313

H 2.778406 -2.878449 1.826359

H 0.706138 -1.468806 5.322350

H 2.588436 -2.736029 4.286898

C -1.040550 1.662163 1.650382

C -0.699465 4.197689 2.811942

C -1.836374 2.062427 2.733906

C -0.084638 2.554349 1.138182

C 0.083344 3.812599 1.720749

C -1.663888 3.321971 3.312477

H -2.606215 1.399225 3.117887

H 0.503002 2.266780 0.271608

H 0.820961 4.497121 1.308713

H -2.291431 3.619402 4.149274

H -0.567524 5.179491 3.260151

C -2.935735 -0.504363 1.254194

C -3.911046 0.121520 0.436096

C -3.288027 -1.518360 2.151174

C -5.235337 -0.337658 0.556366

C -4.612753 -1.936146 2.263991

H -2.518632 -1.995922 2.748947

C -5.581716 -1.346663 1.452079

H -6.000027 0.096166 -0.073793

H -4.878860 -2.724457 2.963555

H -6.615145 -1.681346 1.504469

C -2.159313 -1.648661 -1.721440

C -2.192692 -2.940014 -1.152958

C -2.947963 -1.440026 -2.859929

C -2.968405 -3.964642 -1.706670

H -2.960860 -0.455343 -3.321945

C -3.731358 -3.721304 -2.854368

H -2.983336 -4.953247 -1.259196

H -4.328656 -4.524864 -3.280143

O -1.423980 -3.122757 -0.025434

C -1.325895 -4.416758 0.528280

H -2.293627 -4.773119 0.912147

H -0.944650 -5.142334 -0.203618

H -0.616629 -4.335665 1.355057

C -3.531852 1.194857 -0.530550

C -4.401510 2.299173 -0.762392

C -4.073371 3.305208 -1.672346

C -2.850215 3.254562 -2.344477

H -4.747861 4.134719 -1.848001

H -2.591841 4.042588 -3.048678

O -5.563452 2.344497 -0.024814

C -6.393910 3.481724 -0.132944

H -7.211938 3.322931 0.574247

H -5.858824 4.403788 0.132860

H -6.811049 3.592908 -1.143914

C -2.286358 1.177016 -1.207467

Rh -0.797571 -0.220782 -1.205111

O 1.152481 0.795879 -1.087113

C 1.745215 -0.249381 -1.662413

O 2.975479 -0.262251 -1.881776

O 0.937139 -1.249256 -1.918854

Br 5.860181 2.655327 -0.802855

K 3.441698 1.078531 0.503988

O 4.841465 -1.058121 0.894602

C 5.416542 -2.156262 0.705159

O 6.138781 -2.519132 -0.255746

O 5.206245 -3.111893 1.707217

K 5.549424 -0.386082 -1.735395

H 5.700783 -3.884661 1.386839

C -1.960052 2.217792 -2.095659

H -0.989565 2.212827 -2.587736

C -3.721678 -2.459017 -3.435931

H -4.311541 -2.256179 -4.327525

**TS14A-2j**

P 1.516069 1.149550 0.342052

C 2.174621 2.597257 -0.607078

C 3.127904 4.809021 -2.053893

C 1.568037 2.965702 -1.820661

C 3.269862 3.341059 -0.135486

C 3.741174 4.439531 -0.854963

C 2.045272 4.069784 -2.531555

H 0.730922 2.380769 -2.193640

H 3.765367 3.061997 0.788316

H 4.590416 5.004282 -0.477243

H 1.562961 4.346925 -3.465437

H 3.495360 5.666750 -2.612739

C 0.269915 1.839711 1.512597

C -1.849833 2.675647 3.155348

C -0.555707 2.905631 1.124080

C 0.006101 1.179453 2.726491

C -1.035440 1.608398 3.550766

C -1.617459 3.315681 1.934376

H -0.406446 3.395935 0.167908

H 0.603214 0.323067 3.023737

H -1.217261 1.097667 4.494383

H -2.313986 4.066863 1.580264

H -2.682126 2.995153 3.777501

C 2.927008 0.615739 1.386399

C 3.735165 -0.473406 0.979199

C 3.194535 1.273982 2.600105

C 4.771418 -0.870935 1.853300

C 4.234894 0.872322 3.432513

H 2.565428 2.106056 2.900108

C 5.021000 -0.217932 3.054312

H 5.380765 -1.723299 1.577367

H 4.419900 1.395466 4.367129

H 5.824348 -0.566543 3.699121

Rh 0.554450 -0.553711 -0.686561

C 1.028374 -2.290508 0.509072

C -0.089491 -3.066627 0.092653

C 1.518629 -2.559677 1.798516

C -0.683394 -4.032765 0.898637

C 0.951069 -3.544543 2.611098

H 2.364759 -2.004251 2.179813

C -0.146399 -4.279803 2.166140

H -1.572862 -4.557364 0.568449

H 1.367485 -3.726854 3.598855

H -0.600264 -5.036268 2.800912

O -0.558304 -2.715093 -1.150352

C -1.884045 -3.107876 -1.534788

H -2.075239 -2.583435 -2.471413

H -1.913989 -4.191171 -1.707464

H -2.625679 -2.816040 -0.785371

C 3.543719 -1.245929 -0.283699

C 4.693600 -1.505864 -1.076764

C 2.271558 -1.726433 -0.743134

C 4.640961 -2.285425 -2.231822

C 2.258819 -2.553153 -1.904185

C 3.408886 -2.818134 -2.621882

H 5.528425 -2.475332 -2.822543

H 1.316307 -2.960318 -2.243657

H 3.351631 -3.434599 -3.516514

O 5.863603 -0.906737 -0.662877

C 6.997579 -0.984978 -1.500532

H 7.769202 -0.382078 -1.015234

H 7.363206 -2.016652 -1.606135

H 6.793943 -0.577092 -2.499904

O -0.406802 0.763187 -1.953036

C -1.726180 0.675410 -1.788335

O -2.478421 1.286184 -2.607414

Br -5.202972 -2.291364 0.359546

K -3.707284 0.454686 1.292467

O -4.475210 3.336002 1.038480

C -4.399451 2.866112 -0.116563

O -5.044910 1.872007 -0.580217

O -3.454834 3.436696 -0.953330

K -4.636249 -0.179427 -1.990240

O -2.150839 -0.053901 -0.816015

H -3.276427 2.786458 -1.671987

**INT14A-2j**

C -1.057680 -0.359280 1.675031

C -1.504666 -1.882109 3.994409

C -2.055170 0.007160 2.589536

C -0.264921 -1.486035 1.950164

C -0.495357 -2.244785 3.099601

C -2.282330 -0.751137 3.739080

H -2.638566 0.906756 2.420357

H 0.536676 -1.752133 1.267112

H 0.129227 -3.111330 3.302523

H -3.060385 -0.452691 4.437580

H -1.675462 -2.469794 4.893309

C -0.899276 2.387052 0.865793

C -1.167124 4.982445 1.928322

C -2.065977 3.134377 0.643307

C 0.131846 2.965276 1.626558

C 0.000964 4.250542 2.153631

C -2.198380 4.420471 1.173458

H -2.871923 2.715688 0.048923

H 1.040843 2.392495 1.803755

H 0.813271 4.681597 2.733575

H -3.109610 4.985225 0.990098

H -1.270048 5.986075 2.333558

C -1.886011 0.573862 -1.150871

C -3.055694 -0.210749 -1.283487

C -1.543004 1.441677 -2.207673

C -3.830115 -0.069482 -2.453628

C -2.327655 1.578092 -3.347257

H -0.620141 2.011693 -2.134226

C -3.487871 0.814159 -3.470265

H -4.713249 -0.692784 -2.556893

H -2.022960 2.261100 -4.136060

H -4.111442 0.890224 -4.357840

C -3.537973 -1.267671 -0.335575

C -4.813695 -1.213662 0.257934

C -2.771054 -2.451280 -0.188661

C -3.257866 -3.533198 0.551259

C -4.515056 -3.448286 1.150652

H -2.666746 -4.434975 0.656672

H -4.888217 -4.288789 1.730853

O -1.574010 -2.449258 -0.826377

C -0.749952 -3.610619 -0.780335

H 0.185371 -3.317561 -1.255067

H -0.541598 -3.907005 0.254392

H -1.226462 -4.445743 -1.312025

C -5.699799 -0.009374 0.146238

C -6.852181 -0.025128 -0.671502

C -5.447310 1.141157 0.896870

C -7.705564 1.082761 -0.722561

H -4.572528 1.152181 1.537804

C -7.421511 2.219165 0.040266

H -8.586202 1.072123 -1.354665

H -8.091907 3.073612 -0.008872

O -7.051322 -1.167072 -1.401675

C -8.190222 -1.245240 -2.237862

H -8.146574 -2.228701 -2.710275

H -9.123095 -1.160881 -1.663252

H -8.178989 -0.469524 -3.015873

P -0.596383 0.670892 0.206888

Rh 1.544510 0.423020 -0.455549

C -6.291699 2.254363 0.851570

H -6.065954 3.132675 1.449723

C -5.288537 -2.303861 1.006546

H -6.269863 -2.241076 1.467413

O 1.709692 -1.567746 -0.764812

C 2.998435 -1.514074 -1.072184

O 3.671286 -2.501948 -1.411440

Br 6.500788 2.085482 -0.923483

K 4.889119 0.487207 1.302233

O 6.753811 -1.317249 2.052582

C 6.342938 -2.033521 1.118765

O 6.915582 -2.459695 0.084789

O 4.950880 -2.325167 1.212704

K 6.019412 -0.937753 -1.854703

O 3.480117 -0.288622 -0.971829

H 4.688174 -2.763599 0.378294

**INT15A-2j**

P 0.582088 -0.253023 1.326111

C 1.544154 1.020735 2.295444

C 2.884115 3.120401 3.622223

C 2.738995 0.783926 2.991726

C 1.046596 2.336537 2.258114

C 1.700646 3.373194 2.924375

C 3.403652 1.825843 3.646125

H 3.164071 -0.213034 3.019418

H 0.150351 2.541055 1.677652

H 1.298435 4.381707 2.874220

H 4.331060 1.619446 4.175855

H 3.402927 3.929161 4.131487

C -0.828343 -0.671211 2.472937

C -3.093039 -1.348465 4.012614

C -1.247723 0.151665 3.529118

C -1.564279 -1.839379 2.198541

C -2.684236 -2.177492 2.960694

C -2.372082 -0.184702 4.292586

H -0.699865 1.058957 3.763063

H -1.270551 -2.475861 1.370244

H -3.268324 -3.053853 2.696143

H -2.681767 0.467213 5.106514

H -3.973940 -1.603695 4.595978

C 1.607089 -1.808708 1.589211

C 2.259054 -2.515077 0.553918

C 1.726229 -2.294009 2.906061

C 3.018621 -3.653443 0.881256

C 2.480373 -3.423221 3.214128

H 1.212791 -1.773365 3.708940

C 3.137517 -4.106648 2.190850

H 3.515901 -4.184632 0.074436

H 2.548422 -3.766428 4.243653

H 3.730239 -4.991945 2.409004

Rh -0.208235 0.498256 -0.647026

Br 1.756818 1.963808 -1.430460

C 1.720858 3.823573 -0.893076

C 2.934169 4.479691 -0.734845

C 0.493824 4.493956 -0.708923

C 2.959659 5.833679 -0.386001

H 3.858223 3.929177 -0.881445

C 0.534533 5.852785 -0.354351

C 1.756683 6.511049 -0.198044

H 3.910750 6.343836 -0.263331

H -0.392646 6.395506 -0.209901

H 1.758673 7.564062 0.072469

O -0.631774 3.779368 -0.879415

C -1.901731 4.399228 -0.684319

H -2.646150 3.615096 -0.824659

H -1.986107 4.807754 0.331594

H -2.057797 5.203764 -1.414719

C 2.180221 -2.172625 -0.902080

C 1.014963 -2.525785 -1.625894

C 3.284860 -1.648660 -1.595525

C 0.939665 -2.292296 -3.002169

C 3.198947 -1.424354 -2.980103

C 2.030627 -1.731627 -3.667088

H 0.035205 -2.524609 -3.550581

H 4.054208 -1.008324 -3.504216

H 1.960823 -1.539634 -4.734807

O 0.036873 -3.141118 -0.907366

C -1.187663 -3.474138 -1.560320

H -1.819813 -3.926364 -0.794604

H -1.686690 -2.578762 -1.937043

H -1.014153 -4.198907 -2.368107

C 4.562405 -1.310570 -0.890216

C 5.722166 -2.095610 -1.080180

C 4.653078 -0.200749 -0.046309

C 6.924688 -1.756248 -0.449859

C 5.848343 0.142689 0.592731

H 3.769565 0.411386 0.099307

C 6.982528 -0.635847 0.384647

H 7.814217 -2.359061 -0.595342

H 5.881221 1.010989 1.244751

H 7.922246 -0.383174 0.870208

O 5.576534 -3.193305 -1.889035

C 6.705745 -4.008368 -2.128323

H 6.365644 -4.803276 -2.795697

H 7.516625 -3.449288 -2.616469

H 7.091627 -4.455822 -1.201322

O -1.415524 0.915325 -2.337400

C -2.336734 0.113093 -1.904871

O -3.438644 -0.096259 -2.465236

Br -5.561853 -2.819219 0.392918

K -4.009148 0.010548 1.006372

O -6.045804 1.855852 0.641380

C -5.796274 1.768982 -0.574349

O -6.490868 1.358087 -1.539012

O -4.452632 2.122195 -0.900867

K -5.809510 -1.051261 -2.170358

O -2.018463 -0.483595 -0.751019

H -4.280426 1.774570 -1.797455

**INT16A-2j**

P 0.016036 0.771842 1.254218

C 0.344877 -0.703571 2.352760

C 1.045863 -2.957384 3.906365

C -0.640353 -1.552328 2.876723

C 1.692548 -1.019161 2.607414

C 2.039950 -2.122403 3.386159

C -0.293029 -2.671808 3.641169

H -1.687879 -1.346604 2.688113

H 2.472064 -0.390887 2.184385

H 3.089340 -2.335336 3.576490

H -1.077746 -3.316388 4.031330

H 1.314977 -3.823154 4.507592

C 0.600455 2.162559 2.365866

C 1.492728 4.345832 3.912431

C 0.865960 2.014437 3.737456

C 0.801702 3.423588 1.775431

C 1.235947 4.502400 2.547930

C 1.310795 3.095555 4.503873

H 0.736203 1.048282 4.215725

H 0.649950 3.542898 0.705423

H 1.387774 5.469008 2.072673

H 1.515715 2.955429 5.563545

H 1.839818 5.188339 4.507232

C -1.856445 0.964094 1.467144

C -2.801521 0.815278 0.423139

C -2.339158 1.255451 2.757463

C -4.171026 0.939030 0.722878

C -3.697782 1.376889 3.037243

H -1.630659 1.395467 3.566910

C -4.624926 1.210219 2.009404

H -4.881093 0.817141 -0.089786

H -4.024987 1.602976 4.049821

H -5.691505 1.300912 2.204254

Rh 1.055489 0.815051 -0.743298

C 1.343936 2.693945 -2.383652

O 0.702048 2.763420 -1.204596

O 1.894523 1.511084 -2.558097

Br 1.644135 -1.690915 -0.976915

O 1.362464 3.635846 -3.179512

C 3.510610 -2.076304 -0.625155

C 3.866677 -3.008367 0.339712

C 4.483357 -1.375018 -1.364358

C 5.220011 -3.246652 0.608610

H 3.090348 -3.523091 0.895832

C 5.835796 -1.607907 -1.071530

C 6.194941 -2.537967 -0.092643

H 5.501350 -3.971616 1.367688

H 6.600532 -1.070636 -1.623499

H 7.248554 -2.708945 0.115496

O 4.033401 -0.544855 -2.317150

C 4.817415 0.569053 -2.736373

H 4.088453 1.264906 -3.154054

H 5.321778 1.032017 -1.879385

H 5.565067 0.264949 -3.482908

C -2.470165 0.582513 -1.021132

C -2.178788 1.706573 -1.830890

C -2.577616 -0.681603 -1.623834

C -1.879794 1.543866 -3.187392

C -2.299246 -0.831670 -2.991661

C -1.928627 0.270909 -3.753679

H -1.568859 2.391451 -3.785825

H -2.369006 -1.817545 -3.442798

H -1.676703 0.146546 -4.803949

O -2.259628 2.911300 -1.211263

C -1.941265 4.082636 -1.958393

H -2.061270 4.911063 -1.255962

H -0.910257 4.057649 -2.322703

H -2.647726 4.214402 -2.792162

C -3.001472 -1.894370 -0.851105

C -4.352260 -2.311188 -0.853507

C -2.077458 -2.676651 -0.155869

C -4.744250 -3.471920 -0.177704

C -2.459120 -3.838041 0.525232

H -1.037592 -2.367154 -0.151721

C -3.792770 -4.231928 0.510469

H -5.781230 -3.789445 -0.178298

H -1.713847 -4.418148 1.061755

H -4.108680 -5.132020 1.033192

O -5.224749 -1.511714 -1.547565

C -6.585191 -1.885859 -1.594701

H -7.085346 -1.124686 -2.197884

H -6.722735 -2.868793 -2.068048

H -7.039388 -1.908924 -0.593596

**TS17A-2j**

P 0.273116 -0.062197 0.519781

C -0.019056 -1.697199 1.376676

C -0.210344 -4.168984 2.735489

C -0.629378 -2.807925 0.773340

C 0.506222 -1.857032 2.673145

C 0.405267 -3.072827 3.346987

C -0.722797 -4.031639 1.445913

H -1.038600 -2.722059 -0.227304

H 0.997593 -1.017823 3.156260

H 0.816148 -3.167087 4.349525

H -1.199844 -4.876569 0.953840

H -0.282932 -5.119937 3.258151

C -0.553197 1.096515 1.722078

C -1.542904 2.904559 3.641214

C -1.571418 0.717255 2.608883

C -0.031408 2.398585 1.815339

C -0.527668 3.294167 2.764201

C -2.064139 1.612920 3.560673

H -1.973754 -0.289783 2.576789

H 0.761938 2.693645 1.134214

H -0.108269 4.295881 2.824731

H -2.853530 1.297194 4.239018

H -1.921246 3.601043 4.386504

C -0.774474 -0.263558 -1.027943

C -2.097465 0.144352 -1.331469

C -0.064893 -0.985436 -2.006963

C -2.658948 -0.249587 -2.561565

C -0.639126 -1.368017 -3.216790

H 0.974040 -1.233934 -1.815177

C -1.956535 -1.008609 -3.491969

H -3.669230 0.080164 -2.788222

H -0.048794 -1.925931 -3.939443

H -2.426650 -1.288670 -4.432051

C 2.941529 2.826807 -1.091900

O 1.721685 2.264891 -0.993556

O 3.852524 2.077612 -0.500629

Rh 2.390246 0.679930 0.088768

O 3.131774 3.899451 -1.660969

Br 3.854722 -0.937662 1.573022

C 3.848203 -1.339450 -0.397514

C 4.963053 -0.898965 -1.151998

C 3.081376 -2.424338 -0.841177

C 5.142425 -1.425995 -2.435147

C 3.297181 -2.956384 -2.113854

H 2.291888 -2.810983 -0.206179

C 4.310827 -2.440263 -2.922319

H 5.956001 -1.053259 -3.049531

H 2.665000 -3.767405 -2.467651

H 4.479186 -2.839061 -3.919490

O 5.817712 -0.023892 -0.568887

C 6.465712 0.936609 -1.395255

H 6.950031 1.632948 -0.708886

H 7.230069 0.470300 -2.035354

H 5.728466 1.483385 -1.987135

C -2.951813 1.084238 -0.533030

C -2.556714 2.447994 -0.481655

C -4.219393 0.727768 -0.037150

C -3.390629 3.412020 0.092519

C -5.044932 1.707091 0.545579

C -4.628703 3.029679 0.612299

H -3.081545 4.450059 0.128157

H -6.015250 1.416268 0.936276

H -5.272554 3.780003 1.065808

O -1.359433 2.718749 -1.046718

C -0.866718 4.053110 -1.089474

H 0.150798 3.966684 -1.472277

H -1.493665 4.676574 -1.743217

H -0.837801 4.495113 -0.085860

C -4.724883 -0.679907 -0.089826

C -5.892320 -1.010930 -0.817453

C -4.079749 -1.706889 0.604153

C -6.386721 -2.320039 -0.817803

C -4.562150 -3.018242 0.606400

H -3.175076 -1.470081 1.152233

C -5.720773 -3.319659 -0.102224

H -7.280366 -2.571837 -1.377793

H -4.028836 -3.787065 1.157945

H -6.114800 -4.333162 -0.112028

O -6.472956 0.012320 -1.522172

C -7.622731 -0.266396 -2.294102

H -7.899758 0.677080 -2.769731

H -7.421757 -1.017058 -3.071504

H -8.459316 -0.615828 -1.672043

**INT17A-2j**

P -0.441107 0.117615 0.371629

C -0.300185 1.791727 1.168654

C -0.121750 4.287617 2.458833

C 0.597033 2.769726 0.713551

C -1.111919 2.082152 2.279793

C -1.017729 3.318517 2.917598

C 0.683624 4.009469 1.355004

H 1.232116 2.574649 -0.141960

H -1.835883 1.346952 2.622135

H -1.659723 3.526666 3.769889

H 1.382837 4.754285 0.982333

H -0.056924 5.253597 2.954760

C 0.253294 -0.985213 1.679608

C 1.170841 -2.650269 3.743093

C 1.128655 -0.491596 2.658385

C -0.175465 -2.320014 1.752588

C 0.288655 -3.142679 2.779986

C 1.590805 -1.321198 3.680517

H 1.433015 0.549201 2.642324

H -0.866745 -2.698209 1.005403

H -0.058881 -4.171485 2.833828

H 2.270564 -0.923626 4.430262

H 1.522404 -3.295560 4.545250

C 0.693914 0.297326 -1.095704

C 2.025461 -0.148780 -1.284884

C 0.090401 1.072931 -2.105069

C 2.689789 0.228516 -2.468322

C 0.772200 1.440765 -3.260072

H -0.939826 1.387674 -1.983034

C 2.086084 1.016340 -3.442246

H 3.703626 -0.130349 -2.616845

H 0.266966 2.036327 -4.015552

H 2.635204 1.280471 -4.343220

C -1.817114 -2.273540 -1.945680

O -2.003075 -0.957534 -2.176845

O -1.987386 -2.567163 -0.673372

Rh -2.520095 -0.494955 -0.253102

O -1.536049 -3.068120 -2.844090

Br -3.442315 -0.607923 2.155792

C -3.393681 1.313304 -0.612873

C -4.624206 0.912292 -1.149238

C -3.141263 2.686496 -0.576435

C -5.584300 1.789862 -1.636912

C -4.087830 3.602474 -1.065662

H -2.208189 3.067884 -0.172725

C -5.300970 3.161773 -1.593271

H -6.530037 1.425836 -2.030863

H -3.869695 4.668661 -1.030209

H -6.031611 3.876282 -1.966415

O -4.802416 -0.480496 -1.092520

C -5.100281 -1.139566 -2.330194

H -5.146637 -2.204915 -2.096891

H -6.070576 -0.806684 -2.718797

H -4.303377 -0.957291 -3.056473

C 2.817661 -1.062412 -0.394586

C 2.455047 -2.433490 -0.343944

C 4.022143 -0.662599 0.213714

C 3.247494 -3.357843 0.345689

C 4.807973 -1.600196 0.905947

C 4.417128 -2.931186 0.974406

H 2.954890 -4.400059 0.389072

H 5.727353 -1.270322 1.380953

H 5.027254 -3.651071 1.515254

O 1.332488 -2.759920 -1.021416

C 0.942506 -4.127565 -1.133128

H 0.045308 -4.113621 -1.755233

H 1.742395 -4.715430 -1.605505

H 0.712523 -4.549535 -0.147019

C 4.521978 0.750207 0.165501

C 5.596088 1.111221 -0.679525

C 3.992656 1.733102 1.003597

C 6.102120 2.415666 -0.673413

C 4.489741 3.040222 1.017408

H 3.174642 1.461076 1.661239

C 5.546186 3.376141 0.177229

H 6.921523 2.692784 -1.327159

H 4.048622 3.779404 1.679836

H 5.947436 4.386753 0.172257

O 6.080232 0.117282 -1.489587

C 7.145034 0.421961 -2.368559

H 7.361556 -0.502775 -2.907556

H 6.868138 1.204139 -3.088983

H 8.044385 0.742028 -1.823603

**TS18-2j**

P -1.346732 0.484528 -0.514887

C -2.086487 1.984282 -1.324667

C -3.163830 4.228827 -2.638239

C -3.023256 2.805923 -0.682847

C -1.692271 2.304739 -2.636998

C -2.229665 3.415513 -3.285450

C -3.556135 3.921504 -1.336143

H -3.343128 2.583946 0.327943

H -0.932717 1.706732 -3.129387

H -1.905507 3.651109 -4.295878

H -4.277075 4.548378 -0.817211

H -3.576846 5.098334 -3.144334

C -1.749553 -0.811297 -1.776680

C -2.287235 -2.705459 -3.782888

C -2.872905 -0.675380 -2.610016

C -0.896475 -1.909055 -1.961370

C -1.168909 -2.845477 -2.961297

C -3.143280 -1.618462 -3.600754

H -3.534855 0.175550 -2.503106

H -0.035729 -2.029026 -1.311563

H -0.490122 -3.683528 -3.099122

H -4.020608 -1.497050 -4.231049

H -2.490976 -3.435675 -4.563027

C -2.366392 0.326710 1.037560

C -3.491113 -0.491615 1.306591

C -1.938670 1.233384 2.027707

C -4.132857 -0.350322 2.552435

C -2.602483 1.367182 3.242481

H -1.061293 1.842639 1.841949

C -3.709113 0.564717 3.509211

H -4.982211 -0.994120 2.759808

H -2.237417 2.078842 3.978066

H -4.233588 0.639143 4.459012

C 0.820054 -1.511914 1.522285

O 0.533722 -0.242704 1.845101

O 1.055767 -1.644175 0.236453

O 0.871184 -2.414995 2.365600

Br 1.604165 1.014729 -2.437725

C 1.037407 2.532985 0.463533

C 1.914618 2.808820 1.533745

C 0.476550 3.654760 -0.168145

C 2.156500 4.112658 1.985167

C 0.705232 4.962403 0.272416

H -0.143611 3.516265 -1.043396

C 1.537585 5.197152 1.366578

H 2.855012 4.265778 2.805048

H 1.728078 6.209623 1.716983

O 2.658293 1.772169 2.083564

C 2.417501 1.466865 3.456552

H 3.134224 0.687364 3.723197

H 2.580018 2.346023 4.097051

H 1.404649 1.076362 3.584448

C -4.075309 -1.558725 0.427480

C -3.363450 -2.775142 0.264790

C -5.389603 -1.474047 -0.070319

C -3.932465 -3.846935 -0.431839

C -5.219882 -3.725424 -0.954751

H -3.376835 -4.767552 -0.562993

H -5.655751 -4.558568 -1.501363

O -2.143396 -2.817235 0.846536

C -1.396633 -4.033407 0.832756

H -0.481545 -3.808434 1.383896

H -1.964691 -4.836558 1.322966

H -1.154938 -4.330875 -0.194888

P 4.286954 -0.543900 0.150296

C 5.573050 0.686367 -0.382854

C 7.396195 2.701214 -1.132162

C 6.575136 0.431133 -1.331251

C 5.493246 1.974434 0.178032

C 6.401589 2.967914 -0.187574

C 7.477127 1.431172 -1.704609

H 6.650774 -0.552787 -1.783699

H 4.711450 2.192412 0.900436

H 6.320977 3.957583 0.256101

H 8.244227 1.214091 -2.445151

H 8.096869 3.480007 -1.425340

C 5.004527 -1.160920 1.752865

C 5.930838 -2.117471 4.238639

C 6.287189 -0.829402 2.219253

C 4.188173 -1.978604 2.557090

C 4.653331 -2.454308 3.784747

C 6.745703 -1.301712 3.451948

H 6.932465 -0.195760 1.618615

H 3.181311 -2.243916 2.243624

H 4.001363 -3.079502 4.389746

H 7.742302 -1.030563 3.794888

H 6.286141 -2.482478 5.200078

C 4.626059 -1.976842 -0.981887

C 3.888401 -2.038585 -2.176696

C 5.549642 -2.998268 -0.707257

C 4.087169 -3.085814 -3.078368

H 3.156326 -1.264917 -2.395280

C 5.738841 -4.050265 -1.606626

H 6.120745 -2.975463 0.216193

C 5.010085 -4.095491 -2.796760

H 3.508539 -3.115427 -3.998825

H 6.456173 -4.835009 -1.374937

H 5.156540 -4.915729 -3.496530

Rh 0.869507 0.533156 -0.011724

H 0.238162 5.794480 -0.251324

C -5.947884 -2.557160 -0.770809

H -6.958486 -2.470536 -1.159090

C -6.248049 -0.259400 0.120644

C -7.323924 -0.277312 1.038049

C -6.054709 0.899185 -0.633948

C -8.164154 0.832718 1.176234

C -6.886408 2.015400 -0.501873

H -5.232484 0.926961 -1.340208

C -7.941936 1.976300 0.403036

H -8.984892 0.818542 1.884527

H -6.700746 2.900555 -1.103190

H -8.600292 2.833682 0.520239

O -7.463528 -1.425793 1.772849

C -8.512808 -1.499594 2.717855

H -8.430474 -2.485891 3.179360

H -8.417750 -0.728030 3.494475

H -9.498454 -1.404834 2.240646

**3aj**

P -1.729558 -0.085377 -0.561014

C -1.624659 1.759500 -0.747360

C -1.503800 4.529727 -1.253192

C -1.292225 2.661894 0.275755

C -1.881739 2.271723 -2.031223

C -1.831156 3.644308 -2.281935

C -1.230686 4.034232 0.024030

H -1.069583 2.291451 1.271179

H -2.117932 1.585644 -2.841388

H -2.036907 4.019569 -3.281130

H -0.969789 4.717106 0.828823

H -1.455421 5.598248 -1.446610

C -3.451839 -0.321389 0.088634

C -6.083748 -0.888957 0.926522

C -3.827715 -1.626704 0.456768

C -4.422024 0.690045 0.139923

C -5.727492 0.406498 0.552964

C -5.125703 -1.906179 0.879425

H -3.089982 -2.424702 0.419027

H -4.161199 1.705809 -0.140400

H -6.464228 1.205453 0.586906

H -5.392540 -2.919863 1.168303

H -7.098257 -1.107009 1.249793

C -0.722740 -0.421429 0.965930

C 0.571205 -0.976212 0.832477

C -1.216919 -0.154795 2.253886

C 1.330196 -1.221984 1.987403

C -0.452923 -0.407871 3.392533

H -2.219065 0.248673 2.365074

C 0.829348 -0.939810 3.256722

H 2.324362 -1.644289 1.874473

H -0.858819 -0.192093 4.377643

H 1.435605 -1.143425 4.135804

C 1.171062 -1.346835 -0.491157

C 2.274176 -0.656406 -1.025526

C 0.681640 -2.481741 -1.182018

C 2.850067 -1.081044 -2.234497

C 1.255732 -2.891125 -2.387347

C 2.340703 -2.182959 -2.908332

H 3.696702 -0.532959 -2.636329

H 0.873534 -3.754507 -2.919024

H 2.786489 -2.503319 -3.846406

O -0.360454 -3.136621 -0.584546

C -0.899878 -4.275911 -1.232710

H -1.704505 -4.634164 -0.587636

H -1.311391 -4.023529 -2.218489

H -0.148415 -5.068728 -1.346119

C 2.847560 0.555360 -0.356321

C 4.026784 0.465671 0.414962

C 2.259067 1.810628 -0.525370

C 4.585255 1.611271 0.992223

C 2.808350 2.959592 0.051158

H 1.355309 1.885464 -1.122208

C 3.972065 2.853890 0.806815

H 5.489248 1.546055 1.587013

H 2.325723 3.921463 -0.094008

H 4.415071 3.736490 1.261127

O 4.551229 -0.791909 0.558258

C 5.757850 -0.940115 1.286181

H 5.998879 -2.004385 1.251650

H 6.577453 -0.367861 0.831297

H 5.641959 -0.628935 2.333211

**2n**

Br 1.810945 0.000000 0.000002

C -0.101721 -0.000055 -0.000006

C -0.785235 1.215500 -0.000027

C -0.785245 -1.215507 0.000014

C -2.181558 1.207870 0.000023

H -0.234841 2.149919 -0.000024

C -2.181657 -1.207813 -0.000019

H -0.235007 -2.150017 0.000024

C -2.882432 0.000005 0.000001

H -2.718838 2.152037 0.000031

H -2.718863 -2.152023 -0.000015

H -3.968452 0.000103 0.000015

**INT1A-2n**

P -1.908264 0.107847 -0.018157

C -2.474353 -1.012960 -1.378414

C -3.207033 -2.632740 -3.556407

C -1.947034 -0.772282 -2.659798

C -3.366474 -2.079508 -1.203211

C -3.727524 -2.885604 -2.286469

C -2.316507 -1.571088 -3.741954

H -1.232242 0.036717 -2.793840

H -3.776095 -2.290572 -0.220912

H -4.416361 -3.712683 -2.134465

H -1.902443 -1.370450 -4.726569

H -3.490121 -3.260638 -4.397235

C -2.595675 -0.655121 1.524365

C -3.587798 -1.811763 3.888454

C -3.964873 -0.586313 1.836009

C -1.733380 -1.300392 2.420869

C -2.225847 -1.878828 3.592656

C -4.456594 -1.162358 3.007809

H -4.647575 -0.072471 1.165437

H -0.673451 -1.350462 2.200895

H -1.542120 -2.377508 4.274520

H -5.517466 -1.098551 3.235661

H -3.971040 -2.256927 4.802911

C -2.999003 1.597111 -0.210015

C -2.797949 2.660755 0.689045

C -3.961150 1.740157 -1.220677

C -3.541760 3.835243 0.579715

H -2.053036 2.566472 1.475423

C -4.703505 2.918723 -1.328844

H -4.133558 0.933239 -1.925531

C -4.496074 3.967766 -0.431848

H -3.372883 4.647138 1.282067

H -5.445609 3.014784 -2.117253

H -5.073644 4.884014 -0.520678

Rh 0.122704 1.111141 -0.230087

P 1.579428 -0.556789 0.002668

C 2.724232 -0.685293 -1.450977

C 3.401272 -1.877157 -1.763823

C 2.928247 0.448931 -2.254569

C 4.270064 -1.931843 -2.855223

H 3.248159 -2.767731 -1.162195

C 3.800686 0.385731 -3.343775

H 2.406996 1.374068 -2.012591

C 4.471169 -0.800229 -3.648296

H 4.785191 -2.860788 -3.086595

H 3.950406 1.269321 -3.959107

H 5.143981 -0.845470 -4.501040

C 2.704807 -0.288019 1.454020

C 2.155812 0.250862 2.629088

C 4.070089 -0.609328 1.430843

C 2.949525 0.449257 3.759649

H 1.105454 0.530932 2.640445

C 4.866003 -0.398197 2.559087

H 4.518062 -1.014362 0.529192

C 4.308294 0.127337 3.725944

H 2.509358 0.868030 4.660874

H 5.924471 -0.642660 2.522832

H 4.930515 0.292805 4.601548

C 1.070642 -2.328255 0.227220

C 1.363352 -3.058803 1.389087

C 0.356022 -2.962707 -0.803551

C 0.941001 -4.384420 1.521848

H 1.926724 -2.596646 2.192829

C -0.060554 -4.286743 -0.671204

H 0.128376 -2.423189 -1.717160

C 0.227029 -5.001321 0.494585

H 1.177912 -4.934269 2.429158

H -0.613979 -4.756752 -1.479492

H -0.100285 -6.032449 0.598508

O 1.247409 2.657843 -0.817700

C 1.885160 3.688547 -0.094542

C 1.831496 4.940299 -0.995922

C 1.163240 3.987231 1.235173

C 3.356042 3.318376 0.183410

H 2.333018 4.738791 -1.948733

H 0.789758 5.203902 -1.210317

H 2.319224 5.800715 -0.519792

H 1.175224 3.102553 1.881782

H 1.636974 4.816467 1.777382

H 0.118200 4.258636 1.042014

H 3.883329 4.129502 0.701852

H 3.414439 2.420675 0.806673

H 3.881119 3.115515 -0.756752

**TS2A-2n**

P 2.731927 -0.201326 0.289244

C 3.192272 1.537983 0.739562

C 3.773996 4.177874 1.513014

C 4.342645 2.182053 0.263529

C 2.333563 2.236919 1.605184

C 2.623969 3.543905 1.992014

C 4.629227 3.495586 0.647974

H 5.020431 1.662477 -0.405877

H 1.431188 1.752882 1.969646

H 1.947538 4.070734 2.659597

H 5.525412 3.981485 0.270434

H 3.998910 5.198572 1.811249

C 3.053150 -1.089691 1.880916

C 3.510735 -2.466988 4.284724

C 4.052648 -0.659133 2.771303

C 2.276440 -2.210753 2.212825

C 2.511614 -2.893972 3.408731

C 4.280850 -1.346578 3.964066

H 4.649074 0.218174 2.538702

H 1.492141 -2.529300 1.529473

H 1.903852 -3.760129 3.658313

H 5.055561 -1.002346 4.644539

H 3.685600 -2.999090 5.216446

C 4.119287 -0.724221 -0.825395

C 5.282561 -1.358763 -0.364794

C 6.296880 -1.714199 -1.257365

H 5.396666 -1.586629 0.689896

C 5.012059 -0.804472 -3.088067

C 6.167117 -1.434840 -2.618437

H 7.188020 -2.213435 -0.885737

H 4.897743 -0.592212 -4.147873

H 6.956650 -1.714370 -3.311051

Br 0.549835 1.919270 -1.661525

C -0.192837 3.478773 -0.753431

C 0.320192 4.730860 -1.078749

C -0.223959 5.854871 -0.452323

H 1.127089 4.829524 -1.797267

C -1.753396 4.444101 0.783053

C -1.258336 5.713516 0.475733

H -2.566750 4.325141 1.493016

H -1.679529 6.592064 0.956479

H 0.165348 6.840481 -0.693077

O 0.360370 -2.346856 -0.236414

C 0.592038 -3.327254 -1.239417

C -0.408076 -4.465129 -0.947873

C 0.328326 -2.773239 -2.654005

C 2.028901 -3.876612 -1.152306

H -0.247868 -4.864094 0.060148

H -1.436791 -4.095140 -1.009233

H -0.289693 -5.286062 -1.665955

H 1.030317 -1.962227 -2.881719

H 0.444041 -3.553578 -3.417874

H -0.692131 -2.377742 -2.723262

H 2.193403 -4.679535 -1.882165

H 2.759013 -3.086343 -1.349551

H 2.223968 -4.280734 -0.152588

C -1.219973 3.308394 0.167519

H -1.599001 2.317944 0.394524

Rh 0.685496 -0.402055 -0.540787

P -3.020135 -0.375469 0.178547

C -4.243251 0.927257 0.682923

C -5.236473 1.437966 -0.165491

C -4.098775 1.499969 1.961138

C -6.064341 2.484209 0.252024

H -5.369245 1.016200 -1.156768

C -4.933068 2.535463 2.381918

H -3.335975 1.121023 2.637975

C -5.919210 3.034788 1.525289

H -6.829211 2.863352 -0.421142

H -4.815226 2.950714 3.379955

H -6.567756 3.844160 1.849978

C -3.722108 -0.996486 -1.418981

C -4.664994 -2.031069 -1.520768

C -3.248606 -0.399142 -2.600195

C -5.128761 -2.448870 -2.769762

H -5.032939 -2.517022 -0.622329

C -3.722710 -0.808002 -3.848206

H -2.499844 0.387911 -2.542006

C -4.663196 -1.836446 -3.935271

H -5.855330 -3.255156 -2.831486

H -3.347860 -0.331876 -4.750514

H -5.025431 -2.164120 -4.906201

C -3.392545 -1.727587 1.384809

C -2.356515 -2.634386 1.666230

C -4.629697 -1.881302 2.033634

C -2.565036 -3.678577 2.570865

H -1.394840 -2.526920 1.168171

C -4.829366 -2.922825 2.941191

H -5.436047 -1.180795 1.837094

C -3.796883 -3.824521 3.210907

H -1.757586 -4.376243 2.778582

H -5.790923 -3.029269 3.437412

H -3.952601 -4.634351 3.919364

C 3.993446 -0.460845 -2.199616

H 3.080383 -0.000824 -2.567859

**INT2A-2n**

P -0.972081 -0.670696 0.063834

C -0.076124 -2.204581 -0.448553

C 1.400239 -4.462372 -1.247115

C -0.545027 -3.037780 -1.474044

C 1.149096 -2.515144 0.166230

C 1.877439 -3.637395 -0.224800

C 0.190571 -4.157694 -1.871032

H -1.487155 -2.816138 -1.964406

H 1.535002 -1.877265 0.956398

H 2.820168 -3.864772 0.265920

H -0.188231 -4.792930 -2.667509

H 1.969408 -5.335649 -1.554754

C -1.298292 -0.940828 1.866484

C -1.867205 -1.237989 4.599755

C -1.250599 -2.208016 2.469502

C -1.636215 0.178466 2.646145

C -1.921373 0.024387 4.003387

C -1.532035 -2.353073 3.830364

H -0.986699 -3.083143 1.884001

H -1.676755 1.155630 2.170034

H -2.184114 0.895839 4.597896

H -1.487620 -3.338854 4.286543

H -2.084565 -1.352336 5.658761

C -2.640482 -0.856014 -0.709443

C -2.933264 -0.121269 -1.867136

C -3.614083 -1.721110 -0.185993

C -4.170589 -0.259173 -2.498772

H -2.186782 0.566735 -2.253406

C -4.851669 -1.854704 -0.816442

H -3.411067 -2.284832 0.719592

C -5.131511 -1.125609 -1.974781

H -4.386201 0.318274 -3.393945

H -5.599222 -2.524586 -0.399431

H -6.097708 -1.227890 -2.462205

Rh 0.016234 1.267618 -0.419999

Br 2.157458 0.445350 -1.564525

C 3.621487 0.327175 -0.283729

C 3.377994 0.638180 1.050003

C 4.436038 0.543535 1.958150

H 2.381202 0.943659 1.359100

C 5.921229 -0.157299 0.181918

C 5.704559 0.147670 1.527738

H 6.906412 -0.465500 -0.157480

H 6.522743 0.077412 2.238810

H 4.262717 0.782017 3.004039

O -1.553736 2.249695 0.315937

C -1.372932 3.631610 0.072029

C -1.612969 4.389050 1.390269

C 0.071128 3.898485 -0.423967

C -2.380734 4.094765 -0.998013

H -2.620959 4.173402 1.761127

H -0.893598 4.063239 2.150240

H -1.517436 5.475065 1.261645

H 0.281379 3.352673 -1.367374

H 0.230768 4.955734 -0.668149

H 0.812576 3.626261 0.341317

H -2.317147 5.175057 -1.184098

H -2.199518 3.568233 -1.941998

H -3.398503 3.858750 -0.670107

C 4.874648 -0.070413 -0.739926

H 5.034490 -0.308415 -1.786619

**TS3A-2n**

P 1.230787 0.021539 0.053029

C 1.901799 1.752992 0.028961

C 2.815260 4.414893 0.085999

C 2.708073 2.253382 -1.002830

C 1.558532 2.608169 1.091331

C 2.014699 3.924756 1.122323

C 3.158375 3.576915 -0.974481

H 2.989415 1.612859 -1.832020

H 0.929575 2.236523 1.895898

H 1.743465 4.569625 1.954208

H 3.781604 3.948266 -1.783978

H 3.167388 5.442848 0.107191

C 2.072928 -0.690174 1.537410

C 3.281588 -1.796395 3.815812

C 3.365112 -0.292475 1.922719

C 1.386202 -1.638689 2.311675

C 1.995212 -2.189242 3.442409

C 3.965677 -0.845181 3.054678

H 3.900237 0.456488 1.345763

H 0.380825 -1.931201 2.015463

H 1.456564 -2.923042 4.036692

H 4.964400 -0.528460 3.344511

H 3.748755 -2.223247 4.699799

C 2.076966 -0.763676 -1.397328

C 1.474042 -0.620027 -2.658587

C 3.275696 -1.485299 -1.301515

C 2.064338 -1.167159 -3.797663

H 0.529556 -0.086651 -2.734789

C 3.858219 -2.045793 -2.441128

H 3.753024 -1.622141 -0.336733

C 3.258117 -1.885204 -3.690705

H 1.585445 -1.044474 -4.765651

H 4.782139 -2.610871 -2.348805

H 3.712758 -2.323509 -4.575239

Rh -1.003125 -0.026697 0.058980

Br -1.716521 2.560464 -0.399623

O -1.265443 -1.904614 0.636928

C -1.402546 -3.028181 -0.215632

C -2.009976 -2.662986 -1.583383

C -0.050331 -3.740094 -0.423824

C -2.365976 -3.973528 0.534651

H -2.984788 -2.180854 -1.451774

H -1.352166 -1.969780 -2.119621

H -2.148941 -3.556822 -2.205953

H 0.394929 -4.003767 0.541842

H -0.170872 -4.662108 -1.007043

H 0.651514 -3.094224 -0.959434

H -2.518564 -4.908670 -0.019040

H -1.960250 -4.217472 1.522524

H -3.336147 -3.485293 0.676365

C -3.122840 1.286752 0.003839

C -3.878230 0.756051 -1.049303

C -3.430215 1.026204 1.346729

C -4.941358 -0.093329 -0.739939

H -3.632247 0.999036 -2.076472

C -4.495632 0.166763 1.626444

H -2.852431 1.482208 2.141462

C -5.249268 -0.389659 0.591465

H -5.529596 -0.521884 -1.546294

H -4.732077 -0.062359 2.661270

H -6.079687 -1.050798 0.820965

**INT3A-2n**

P 0.974733 -0.099785 0.332206

C 1.942726 1.452974 0.215020

C 3.437823 3.821405 0.168945

C 3.345368 1.404348 0.179535

C 1.294440 2.696822 0.224216

C 2.042853 3.873388 0.205733

C 4.087356 2.586088 0.155353

H 3.859896 0.448886 0.165602

H 0.211677 2.746012 0.230791

H 1.531524 4.831804 0.206688

H 5.172295 2.538080 0.121380

H 4.016703 4.740758 0.145410

C 0.842424 -0.446845 2.129405

C 0.667899 -1.032699 4.865526

C 1.443373 0.398999 3.075283

C 0.140760 -1.586570 2.565708

C 0.061427 -1.873285 3.928784

C 1.354572 0.103907 4.436617

H 1.978786 1.285724 2.755393

H -0.371424 -2.205929 1.837048

H -0.484003 -2.753336 4.258750

H 1.823149 0.765551 5.159949

H 0.600907 -1.260250 5.926134

C 2.052847 -1.416987 -0.366065

C 2.297334 -1.418289 -1.751940

C 2.631644 -2.412386 0.436816

C 3.108136 -2.402719 -2.316628

H 1.852329 -0.652831 -2.382617

C 3.440888 -3.393667 -0.138111

H 2.454479 -2.425424 1.506307

C 3.678742 -3.392737 -1.513499

H 3.289644 -2.394682 -3.387738

H 3.884387 -4.159175 0.492746

H 4.306609 -4.160446 -1.957708

Rh -0.932023 -0.199750 -0.862080

Br -0.097454 1.216023 -2.809315

O -1.610533 -1.790374 0.163071

C -2.768934 -2.379751 -0.413955

C -3.020252 -1.848304 -1.842870

C -2.515912 -3.897596 -0.470803

C -3.990868 -2.077090 0.474236

H -3.178900 -0.756370 -1.853962

H -2.192211 -2.095559 -2.519908

H -3.932143 -2.284261 -2.269158

H -2.320612 -4.282826 0.536116

H -3.379905 -4.434570 -0.882098

H -1.640861 -4.113069 -1.093938

H -4.896242 -2.569127 0.096752

H -3.805382 -2.433300 1.493567

H -4.174799 -0.999013 0.521692

C -1.933663 1.194707 0.176755

C -2.401323 2.345020 -0.480105

C -2.291641 0.972287 1.514617

C -3.223012 3.252077 0.200190

H -2.118960 2.538835 -1.507811

C -3.106906 1.890194 2.184400

H -1.963200 0.075885 2.025047

C -3.574784 3.032517 1.532277

H -3.579328 4.137175 -0.321827

H -3.380876 1.701173 3.219847

H -4.208247 3.743157 2.056895

**TS4A-2n**

P -1.017594 0.498377 0.381749

C -0.964461 2.157640 -0.357758

C -0.537739 4.505133 -1.740205

C -1.997668 3.104164 -0.415377

C 0.312702 2.348010 -0.943040

C 0.479978 3.551670 -1.653014

C -1.770751 4.290644 -1.111784

H -2.955299 2.929340 0.069284

H 1.562418 1.779415 -0.198888

H 1.431128 3.762408 -2.141819

H -2.551241 5.045018 -1.168221

H -0.372701 5.428875 -2.291068

C -2.543461 -0.432576 0.015322

C -4.831724 -1.943042 -0.547280

C -3.533934 0.079520 -0.833485

C -2.694285 -1.718421 0.561485

C -3.836034 -2.466438 0.281648

C -4.677067 -0.673416 -1.105069

H -3.406213 1.050094 -1.298989

H -1.915173 -2.140328 1.189867

H -3.942888 -3.461655 0.703763

H -5.440610 -0.269169 -1.763672

H -5.720039 -2.529172 -0.766363

C -0.965464 0.641893 2.215561

C 0.209662 1.086966 2.847278

C -2.117537 0.416370 2.987985

C 0.225989 1.292016 4.226605

H 1.104480 1.259337 2.257924

C -2.091925 0.629890 4.367152

H -3.034581 0.077477 2.519971

C -0.921428 1.064350 4.989827

H 1.140724 1.630537 4.705607

H -2.990208 0.453453 4.952488

H -0.903395 1.225798 6.064303

Rh 0.822510 -0.146988 -0.756340

Br -0.458141 -0.982817 -2.694661

O 2.184000 1.077986 0.433292

C 3.597858 1.077121 0.090447

C 3.806106 0.559011 -1.342344

C 4.094194 2.523938 0.223134

C 4.295982 0.165175 1.103388

H 3.460635 -0.479087 -1.438016

H 3.275770 1.178906 -2.073905

H 4.870763 0.566962 -1.604418

H 3.901713 2.901750 1.232746

H 5.172071 2.581954 0.032275

H 3.586206 3.181341 -0.491546

H 5.372971 0.120694 0.903058

H 4.150785 0.544523 2.120709

H 3.890596 -0.849765 1.054918

C 1.187313 -1.983580 0.005973

C 1.454286 -3.079092 -0.833270

C 1.320718 -2.157065 1.393470

C 1.866019 -4.303194 -0.294404

H 1.331912 -2.986354 -1.906546

C 1.716601 -3.388187 1.928290

H 1.133962 -1.329683 2.071208

C 1.994020 -4.465913 1.085740

H 2.075289 -5.135197 -0.963280

H 1.813024 -3.497683 3.006431

H 2.304088 -5.421787 1.500407

**INT4A-2n**

P 1.153484 0.105660 0.037851

C 2.534550 1.171944 -0.530225

C 4.569587 2.913917 -1.357660

C 3.848417 1.000897 -0.062674

C 2.254424 2.220315 -1.419008

C 3.267900 3.088429 -1.829457

C 4.858485 1.869168 -0.475145

H 4.083854 0.185055 0.614661

H 1.241380 2.357469 -1.789831

H 3.039034 3.896381 -2.518616

H 5.871747 1.730221 -0.108109

H 5.359161 3.588244 -1.677780

C 0.583018 0.656464 1.676244

C -1.026614 1.666281 3.677109

C 1.133779 0.821862 2.950627

C -0.748891 0.997753 1.392945

C -1.574612 1.508812 2.399200

C 0.306710 1.328913 3.955147

H 2.168954 0.563003 3.157952

H -2.603364 1.782992 2.192635

H 0.698019 1.466795 4.959455

H -1.649670 2.065170 4.474183

C 1.841226 -1.586750 0.138771

C 2.646122 -2.079591 -0.901697

C 1.501518 -2.434191 1.203961

C 3.115672 -3.392244 -0.866218

H 2.916409 -1.436317 -1.734608

C 1.973809 -3.746825 1.233954

H 0.868799 -2.069482 2.006796

C 2.781462 -4.227836 0.201953

H 3.741868 -3.761565 -1.673767

H 1.707925 -4.393622 2.065409

H 3.147373 -5.250569 0.228039

Rh -1.075218 0.689023 -0.541035

Br -3.255137 1.768453 -0.920668

C -1.656628 -1.215874 -0.465206

C -1.320496 -2.024433 -1.557319

C -2.475639 -1.718574 0.551035

C -1.820077 -3.328663 -1.640496

H -0.671588 -1.651604 -2.346769

C -2.955967 -3.029438 0.466303

H -2.753022 -1.095104 1.393623

C -2.634832 -3.835698 -0.627541

H -1.559848 -3.946875 -2.496360

H -3.595722 -3.413468 1.257538

H -3.016525 -4.851305 -0.689893

*^t^*BuOH

C -0.691467 1.265003 -0.510327

C 0.005416 0.000005 0.014456

H -1.749266 1.277080 -0.214974

H -0.212982 2.159633 -0.098279

H -0.653351 1.321938 -1.604564

C 1.490700 0.001157 -0.356801

C -0.689429 -1.266201 -0.510128

H 1.984814 -0.884871 0.055378

H 1.622356 0.001082 -1.444140

H 1.983379 0.888119 0.055084

H -0.651268 -1.323221 -1.604360

H -0.209452 -2.159984 -0.097977

H -1.747184 -1.279973 -0.214708

O -0.014229 0.000094 1.451998

H -0.944533 -0.000340 1.729348

**TS2B-2n**

P -2.527009 0.319257 -0.010708

C -3.373768 1.086400 -1.454440

C -4.554045 2.309387 -3.685690

C -2.726022 1.070841 -2.697727

C -4.622095 1.721301 -1.339871

C -5.206773 2.330247 -2.449214

C -3.315224 1.679752 -3.808783

H -1.758236 0.584724 -2.783792

H -5.139324 1.734883 -0.384451

H -6.171238 2.821408 -2.350194

H -2.805389 1.659910 -4.768268

H -5.012681 2.783337 -4.549564

C -3.833873 -0.678979 0.813656

C -5.715929 -2.340840 2.078676

C -3.797458 -0.869579 2.205280

C -4.817202 -1.344521 0.064218

C -5.752178 -2.167182 0.694256

C -4.735378 -1.690591 2.831286

H -3.036252 -0.373833 2.800130

H -4.859051 -1.216898 -1.013228

H -6.508607 -2.672747 0.099880

H -4.697647 -1.822832 3.909312

H -6.444568 -2.981429 2.568105

C -2.053078 1.657415 1.135334

C -0.643426 1.709150 1.012599

C -2.749193 2.435642 2.058747

C 0.058451 2.620889 1.817417

C -2.030666 3.326007 2.863137

H -3.827810 2.345636 2.162929

C -0.639459 3.410834 2.736171

H 1.138216 2.706900 1.748064

H -2.551229 3.953115 3.581782

H -0.087120 4.101559 3.369372

P 2.250473 0.122065 -0.022165

C 3.095952 -1.057893 -1.160731

C 4.170238 -1.862968 -0.762944

C 2.676199 -1.092639 -2.499030

C 4.818090 -2.685082 -1.689572

H 4.498733 -1.860580 0.271704

C 3.328541 -1.906210 -3.423371

H 1.825584 -0.492464 -2.808323

C 4.401654 -2.706639 -3.020242

H 5.647074 -3.309521 -1.366404

H 2.992657 -1.924876 -4.456933

H 4.905606 -3.346473 -3.739915

C 3.164025 1.688230 -0.413357

C 4.570764 1.711933 -0.436893

C 2.471109 2.857357 -0.756424

C 5.259228 2.877436 -0.769447

H 5.130497 0.810705 -0.205382

C 3.161015 4.025196 -1.093924

H 1.386948 2.855721 -0.770570

C 4.555011 4.039495 -1.097120

H 6.345994 2.876115 -0.780828

H 2.603720 4.920020 -1.357922

H 5.092159 4.946638 -1.361128

C 2.818116 -0.367143 1.663978

C 2.325851 -1.587863 2.162769

C 3.631117 0.427911 2.483301

C 2.668867 -2.009770 3.446075

H 1.664306 -2.179844 1.535116

C 3.961630 0.003624 3.773851

H 4.010857 1.378371 2.123334

C 3.487359 -1.216064 4.255561

H 2.287953 -2.957087 3.818954

H 4.591890 0.630577 4.399447

H 3.747754 -1.544971 5.258307

Rh -0.109989 0.029830 -0.106495

H -0.216269 1.619175 -0.408345

O 0.326334 -1.926042 -0.417750

C -0.462001 -3.042347 -0.766753

C -1.362760 -2.754182 -1.983412

C 0.531204 -4.165327 -1.143826

C -1.313624 -3.506736 0.431116

H -2.103782 -1.983033 -1.748752

H -0.753355 -2.394158 -2.820374

H -1.902076 -3.653262 -2.308625

H 1.193460 -4.380196 -0.297682

H 0.010467 -5.092270 -1.417954

H 1.155260 -3.851400 -1.986911

H -1.903161 -4.401904 0.192247

H -0.662921 -3.743394 1.281342

H -2.003181 -2.718412 0.744970

**INT2B-2n**

P -2.509947 0.366897 0.002227

C -3.370097 1.182864 -1.402953

C -4.604518 2.464159 -3.571781

C -2.751520 1.203809 -2.660779

C -4.619008 1.807049 -1.243603

C -5.229712 2.446372 -2.321306

C -3.367510 1.841545 -3.740548

H -1.786662 0.721206 -2.785681

H -5.117433 1.787548 -0.278377

H -6.193626 2.930034 -2.186764

H -2.879775 1.849596 -4.711602

H -5.083552 2.961102 -4.411287

C -3.798550 -0.700680 0.766173

C -5.659156 -2.450504 1.939498

C -3.794796 -0.920885 2.153533

C -4.739599 -1.378355 -0.025811

C -5.664405 -2.244519 0.558738

C -4.721616 -1.787169 2.733988

H -3.070111 -0.410162 2.780424

H -4.757252 -1.224561 -1.100652

H -6.388602 -2.758800 -0.067454

H -4.710055 -1.942704 3.809512

H -6.379750 -3.125346 2.393572

C -2.012104 1.610430 1.232644

C -0.603964 1.526549 1.196845

C -2.704825 2.443900 2.114331

C 0.117958 2.333766 2.089092

C -1.964408 3.239661 2.991094

H -3.792040 2.470871 2.126688

C -0.565603 3.173625 2.975920

H 1.203253 2.306264 2.120748

H -2.470869 3.903993 3.686335

H 0.003336 3.787028 3.671718

P 2.237567 0.138959 -0.046870

C 3.048358 -0.999969 -1.247408

C 4.099506 -1.855031 -0.895213

C 2.612175 -0.966450 -2.580953

C 4.706519 -2.662094 -1.861797

H 4.441194 -1.901236 0.133857

C 3.223079 -1.766668 -3.544267

H 1.782978 -0.322033 -2.858764

C 4.271885 -2.619101 -3.186131

H 5.517862 -3.325911 -1.574626

H 2.874560 -1.733182 -4.573170

H 4.743654 -3.248270 -3.936290

C 3.070273 1.744059 -0.436063

C 4.453252 1.787788 -0.690493

C 2.333133 2.934471 -0.512246

C 5.082083 2.994324 -0.995410

H 5.038415 0.873322 -0.662597

C 2.965077 4.142119 -0.820347

H 1.264210 2.916325 -0.333495

C 4.338606 4.175804 -1.059816

H 6.151107 3.010044 -1.190629

H 2.377649 5.054670 -0.876808

H 4.828221 5.115225 -1.302775

C 2.884237 -0.366747 1.602956

C 2.382362 -1.566134 2.141387

C 3.789244 0.398080 2.352216

C 2.800960 -1.995063 3.400246

H 1.661991 -2.139092 1.561711

C 4.195405 -0.033812 3.617994

H 4.180347 1.328942 1.954602

C 3.706115 -1.230392 4.142149

H 2.412374 -2.925252 3.806657

H 4.895525 0.568219 4.191476

H 4.024349 -1.564384 5.126343

Rh -0.114918 0.014865 -0.092535

H -0.129727 1.293600 -0.975573

O 0.300118 -1.987166 -0.334759

C -0.430960 -3.132803 -0.706045

C -1.350591 -2.845154 -1.908522

C 0.596926 -4.214040 -1.109522

C -1.263051 -3.643551 0.487272

H -2.099959 -2.090133 -1.648310

H -0.757510 -2.461071 -2.746717

H -1.878321 -3.748119 -2.242067

H 1.263328 -4.431070 -0.266853

H 0.109333 -5.150318 -1.412307

H 1.213357 -3.855960 -1.940900

H -1.807395 -4.565197 0.242275

H -0.604409 -3.853029 1.338615

H -1.990891 -2.889125 0.800606

**TS3B-2n**

P -2.487918 -0.270366 0.004251

C -3.535342 -0.041874 1.504279

C -5.023031 0.171835 3.877787

C -2.915151 0.342427 2.703036

C -4.912216 -0.318299 1.509858

C -5.649806 -0.209861 2.689562

C -3.654072 0.445273 3.882378

H -1.851249 0.561625 2.705836

H -5.411581 -0.612499 0.591728

H -6.715206 -0.424888 2.679337

H -3.160346 0.746714 4.802378

H -5.599930 0.257946 4.794792

C -3.636446 -0.037132 -1.415168

C -5.253400 0.412713 -3.671901

C -3.626851 -0.938100 -2.491726

C -4.460420 1.099141 -1.490820

C -5.265482 1.318381 -2.608647

C -4.431555 -0.713817 -3.610413

H -2.993544 -1.819225 -2.451455

H -4.480291 1.812501 -0.671640

H -5.900119 2.199662 -2.649252

H -4.416902 -1.424092 -4.432990

H -5.879657 0.585437 -4.543025

C -1.914578 -1.991630 0.020587

C -0.508976 -1.852367 0.066183

C -2.580871 -3.221141 0.051513

C 0.232363 -3.048069 0.141160

C -1.812459 -4.382618 0.122415

H -3.667181 -3.273006 0.027504

C -0.414663 -4.286542 0.166631

H 1.317301 -3.032338 0.176090

H -2.293249 -5.357311 0.147405

H 0.178856 -5.197025 0.223687

P 2.236236 -0.047131 -0.018572

C 3.289800 1.428653 -0.378289

C 4.230381 1.450929 -1.418312

C 3.160234 2.558958 0.446450

C 5.030726 2.577928 -1.626508

H 4.347299 0.590755 -2.068697

C 3.967730 3.676350 0.241231

H 2.403884 2.573304 1.221218

C 4.905048 3.690259 -0.795183

H 5.754480 2.579159 -2.437495

H 3.855149 4.544086 0.885756

H 5.529943 4.565239 -0.954709

C 2.943154 -0.629670 1.588754

C 4.302767 -0.457507 1.901821

C 2.104524 -1.246546 2.529502

C 4.812463 -0.908463 3.119926

H 4.962224 0.041493 1.198122

C 2.617539 -1.696621 3.748074

H 1.050401 -1.367321 2.302723

C 3.970909 -1.530509 4.045257

H 5.865575 -0.766702 3.348364

H 1.954852 -2.171393 4.466818

H 4.367896 -1.876313 4.996154

C 2.809971 -1.272234 -1.278187

C 2.179157 -1.248806 -2.533258

C 3.830591 -2.206552 -1.051533

C 2.568262 -2.130553 -3.541345

H 1.376508 -0.538637 -2.717337

C 4.211401 -3.097141 -2.058579

H 4.326392 -2.247861 -0.086835

C 3.584135 -3.059956 -3.304637

H 2.071461 -2.097934 -4.507287

H 4.999647 -3.820291 -1.865957

H 3.881506 -3.753956 -4.086276

Rh -0.109721 0.168718 -0.065945

H -0.068163 1.176095 1.146661

O 0.251334 2.324239 0.083758

C -0.651455 3.396760 -0.121324

C -1.754680 3.439149 0.951214

C 0.190592 4.686876 -0.014472

C -1.269641 3.312807 -1.529901

H -2.388818 2.549244 0.904997

H -1.304269 3.479883 1.950403

H -2.397215 4.320516 0.830243

H 1.006285 4.665648 -0.744084

H -0.420153 5.580403 -0.197542

H 0.630724 4.770258 0.985944

H -1.933778 4.162976 -1.735259

H -0.473484 3.309023 -2.283006

H -1.847153 2.390440 -1.645547

**INT3B-2n**

P 2.521350 0.011601 0.112044

C 3.674085 -1.341208 0.595703

C 3.809440 -2.500106 -0.183356

C 4.362254 -1.264340 1.818924

C 4.623135 -3.551030 0.245465

H 3.278534 -2.578599 -1.127086

C 5.176059 -2.314121 2.243611

H 4.269355 -0.375592 2.438197

C 5.309249 -3.461535 1.457489

H 4.721435 -4.440021 -0.372319

H 5.705020 -2.236603 3.190034

H 5.942353 -4.280160 1.789359

C 2.033715 -0.230585 -1.631372

C 0.623021 -0.254391 -1.561322

C 2.735153 -0.351832 -2.834383

C -0.091458 -0.438303 -2.758845

C 2.004723 -0.523160 -4.012875

H 3.822100 -0.313644 -2.855724

C 0.604821 -0.570416 -3.964848

H -1.176578 -0.485623 -2.763806

H 2.519755 -0.621618 -4.964944

H 0.045511 -0.711247 -4.887730

C 3.549700 1.538912 0.218544

C 2.895753 2.748994 0.495676

C 4.935477 1.550436 -0.007200

C 3.609449 3.947923 0.537043

H 1.822583 2.739915 0.672463

C 5.648167 2.749303 0.038669

H 5.459896 0.621116 -0.209786

C 4.986981 3.949853 0.309920

H 3.090321 4.878297 0.752320

H 6.720772 2.745882 -0.137934

H 5.544776 4.882009 0.346995

Rh 0.140430 -0.005227 0.333251

P -2.152999 0.005342 0.200103

C -2.992909 1.499449 -0.498983

C -4.251951 1.936892 -0.054862

C -2.336463 2.229453 -1.502079

C -4.846172 3.070207 -0.612971

H -4.765276 1.398003 0.736586

C -2.935826 3.358415 -2.063705

H -1.350489 1.914869 -1.830785

C -4.190750 3.781000 -1.620951

H -5.818914 3.399701 -0.256832

H -2.415740 3.913159 -2.840249

H -4.652907 4.665002 -2.052632

C -2.999692 -1.421116 -0.618128

C -2.336849 -2.659813 -0.595160

C -4.247573 -1.330761 -1.253276

C -2.919519 -3.786675 -1.175544

H -1.355264 -2.724468 -0.131894

C -4.824031 -2.457977 -1.843273

H -4.768429 -0.379446 -1.295971

C -4.164090 -3.687568 -1.802123

H -2.395253 -4.738301 -1.149746

H -5.788541 -2.372832 -2.337274

H -4.613724 -4.562873 -2.263744

C -2.783318 -0.036792 1.940067

C -3.690963 -0.990342 2.423063

C -2.257881 0.912314 2.836298

C -4.066261 -0.992097 3.769010

H -4.105593 -1.733774 1.749620

C -2.636710 0.910737 4.178333

H -1.553478 1.659504 2.475251

C -3.541808 -0.044170 4.648894

H -4.771121 -1.737521 4.128367

H -2.223073 1.652471 4.856575

H -3.834392 -0.049817 5.695482

**INT4B-2n**

P 2.426250 -0.821411 0.053236

C 3.439708 -1.450679 -1.352920

C 4.850640 -2.512040 -3.537667

C 2.785374 -1.710245 -2.566911

C 4.813026 -1.723612 -1.249083

C 5.512582 -2.250909 -2.335441

C 3.485542 -2.241690 -3.651162

H 1.722676 -1.494673 -2.649699

H 5.338627 -1.515999 -0.321497

H 6.575609 -2.458533 -2.242489

H 2.965959 -2.438238 -4.585461

H 5.398038 -2.921357 -4.382814

C 3.625908 0.128639 1.078375

C 5.341066 1.720302 2.641190

C 3.765238 -0.109890 2.454191

C 4.351916 1.183967 0.498693

C 5.205294 1.969469 1.272863

C 4.618242 0.680494 3.228045

H 3.207349 -0.917360 2.918657

H 4.256022 1.387406 -0.564904

H 5.762670 2.778332 0.807467

H 4.719497 0.478404 4.291417

H 6.005247 2.333543 3.244554

C 1.807982 -2.234125 1.013968

C 0.407714 -2.024522 0.981214

C 2.417764 -3.324072 1.642066

C -0.383421 -2.979557 1.650773

C 1.602159 -4.251564 2.292286

H 3.498374 -3.449604 1.623847

C 0.212708 -4.068862 2.293551

H -1.465080 -2.889165 1.680464

H 2.040256 -5.110834 2.793871

H -0.417905 -4.794744 2.803722

Rh 0.098798 -0.348352 -0.105015

P -2.223141 -0.392764 0.027698

C -2.897344 -0.562399 1.749545

C -2.126325 -0.031728 2.796278

C -4.128634 -1.160986 2.055934

C -2.583844 -0.079249 4.114083

H -1.155448 0.399499 2.566066

C -4.579594 -1.220336 3.376189

H -4.735491 -1.594976 1.268033

C -3.811888 -0.675426 4.407330

H -1.974020 0.336682 4.911914

H -5.532207 -1.694749 3.597910

H -4.165074 -0.723008 5.434201

C -3.127645 1.108614 -0.589291

C -3.206539 1.339839 -1.973613

C -3.670125 2.065909 0.279987

C -3.825958 2.484915 -2.472484

H -2.792254 0.613676 -2.667638

C -4.283991 3.217126 -0.220514

H -3.622321 1.913020 1.353300

C -4.367320 3.429303 -1.596536

H -3.884110 2.639879 -3.546888

H -4.702228 3.944610 0.470503

H -4.849462 4.322595 -1.984949

C -3.060734 -1.740823 -0.934497

C -2.304866 -2.856896 -1.324663

C -4.417873 -1.684977 -1.298609

C -2.894953 -3.900142 -2.042027

H -1.251500 -2.896163 -1.066087

C -5.005452 -2.727020 -2.017226

H -5.014923 -0.817028 -1.034806

C -4.245478 -3.839112 -2.387942

H -2.294289 -4.757349 -2.334882

H -6.055586 -2.667173 -2.291747

H -4.703177 -4.648930 -2.950332

Br 0.302389 1.842365 -1.724528

C 0.528461 3.417274 -0.632044

C 0.442053 4.668676 -1.236669

C 0.785831 3.266409 0.727699

C 0.620256 5.806203 -0.445793

H 0.237614 4.755888 -2.298536

C 0.961099 4.415534 1.502653

H 0.843981 2.271446 1.159597

C 0.879720 5.682643 0.920890

H 0.554032 6.789256 -0.904116

H 1.165422 4.313249 2.564920

H 1.017786 6.571345 1.530265

**TS5B-2n**

P -2.465615 0.411506 0.154587

C -2.965208 2.030420 -0.573244

C -3.588925 4.554830 -1.635139

C -2.018127 2.741170 -1.325046

C -4.233139 2.598293 -0.363887

C -4.541315 3.852042 -0.892038

C -2.327359 3.997942 -1.849648

H -1.038033 2.301622 -1.490157

H -4.984016 2.057193 0.204625

H -5.525759 4.280892 -0.722922

H -1.583152 4.537287 -2.429738

H -3.832225 5.530830 -2.047046

C -4.023731 -0.569395 0.152499

C -6.296020 -2.226272 0.046654

C -4.419939 -1.312291 1.276501

C -4.776096 -0.680930 -1.030504

C -5.904551 -1.499311 -1.080456

C -5.549827 -2.131203 1.222647

H -3.845666 -1.246305 2.195440

H -4.483432 -0.119187 -1.913589

H -6.477233 -1.570047 -2.001494

H -5.847633 -2.692977 2.104305

H -7.175129 -2.863878 0.006831

C -1.951082 0.689105 1.877040

C -0.587440 0.313871 1.864149

C -2.612686 1.147323 3.019751

C 0.109944 0.407088 3.081747

C -1.894788 1.223981 4.214811

H -3.661566 1.433344 2.982695

C -0.545848 0.848773 4.236438

H 1.156741 0.133251 3.148398

H -2.379260 1.572606 5.123348

H 0.007889 0.906269 5.171540

Rh -0.161894 -0.290128 -0.038092

P 2.132899 0.304342 0.049676

C 3.177316 -0.244538 1.485698

C 2.906300 -1.493879 2.067835

C 4.235475 0.522303 1.997563

C 3.686659 -1.971369 3.122763

H 2.073925 -2.086006 1.700610

C 5.008822 0.047218 3.058555

H 4.455098 1.496772 1.573955

C 4.739494 -1.201820 3.621409

H 3.461818 -2.940167 3.561106

H 5.820918 0.656799 3.446411

H 5.341601 -1.569501 4.448207

C 3.168990 -0.222895 -1.397914

C 2.844320 0.263837 -2.676382

C 4.230539 -1.132146 -1.277672

C 3.569902 -0.135667 -3.797117

H 2.022480 0.963428 -2.794125

C 4.948915 -1.542397 -2.404765

H 4.506580 -1.518333 -0.302054

C 4.623447 -1.044434 -3.665822

H 3.306586 0.256911 -4.775734

H 5.768450 -2.247190 -2.290252

H 5.184453 -1.361005 -4.541066

C 2.384525 2.146341 0.036652

C 1.433830 2.970982 0.658742

C 3.506894 2.747079 -0.559663

C 1.604377 4.356261 0.690768

H 0.557025 2.525500 1.115748

C 3.672165 4.133339 -0.532528

H 4.253013 2.133276 -1.053945

C 2.721769 4.941998 0.093499

H 0.855934 4.976151 1.177197

H 4.544772 4.579317 -1.002990

H 2.849881 6.021196 0.111917

Br -0.305359 -1.301610 -2.506023

C 0.997498 -3.146253 -0.547870

C -1.429927 -3.048160 -0.331291

C 1.003521 -4.325691 0.198464

H 1.914392 -2.757422 -0.974702

C -1.397310 -4.223167 0.420666

H -2.374428 -2.587076 -0.588022

C -0.185973 -4.862019 0.700567

H 1.948924 -4.834759 0.371202

H -2.335068 -4.647061 0.771048

H -0.172566 -5.786463 1.270839

C -0.217376 -2.463839 -0.737194

**INT5B-2n**

P -2.124765 0.390355 -0.214992

C -2.323236 2.172500 -0.631454

C -2.603086 4.850603 -1.428681

C -3.456052 2.911791 -0.255062

C -1.333912 2.792189 -1.411449

C -1.473212 4.123258 -1.807783

C -3.592814 4.242489 -0.652973

H -4.228388 2.451090 0.352905

H -0.448300 2.236358 -1.708230

H -0.695458 4.588304 -2.406943

H -4.474425 4.804271 -0.355777

H -2.711871 5.887554 -1.734874

C -2.839622 -0.608358 -1.548209

C -3.152861 -2.395179 -3.614131

C -4.088249 -0.679364 -2.176997

C -1.736731 -1.388490 -1.938536

C -1.906447 -2.301133 -2.989091

C -4.236464 -1.594399 -3.218968

H -4.917206 -0.047770 -1.866143

H -1.073477 -2.910439 -3.326237

H -5.191618 -1.686621 -3.729651

H -3.286784 -3.098442 -4.433559

C -3.092443 0.142500 1.323224

C -2.849074 0.946319 2.449754

C -4.037769 -0.890039 1.412504

C -3.549724 0.728535 3.635440

H -2.110202 1.740732 2.405537

C -4.735867 -1.104531 2.602226

H -4.226281 -1.526935 0.554430

C -4.495699 -0.296457 3.714295

H -3.352392 1.357953 4.499075

H -5.466783 -1.906594 2.657599

H -5.039792 -0.465971 4.639577

Br 1.521882 -2.026096 -2.306917

C -0.404967 -2.159934 0.573987

C -0.418651 -1.825083 1.933296

C -0.514353 -3.507532 0.201848

C -0.519597 -2.824211 2.908536

H -0.349335 -0.789968 2.249442

C -0.615117 -4.499477 1.183535

H -0.497637 -3.789260 -0.843293

C -0.617200 -4.165373 2.539017

H -0.524754 -2.541276 3.958753

H -0.689171 -5.540564 0.877092

H -0.696615 -4.940707 3.296721

Rh -0.127342 -0.753735 -0.848254

P 1.857267 0.437402 0.268480

C 2.472839 1.796365 -0.835950

C 2.939007 3.031627 -0.355408

C 2.476710 1.567919 -2.223975

C 3.386714 4.015945 -1.239024

H 2.950034 3.232245 0.710821

C 2.926650 2.554884 -3.103400

H 2.150020 0.607348 -2.613607

C 3.378126 3.782558 -2.615434

H 3.743881 4.965292 -0.848050

H 2.924643 2.358086 -4.172203

H 3.725606 4.550338 -3.301835

C 1.543122 1.315119 1.871361

C 2.091094 0.863621 3.081569

C 0.671143 2.419120 1.902518

C 1.782531 1.500589 4.286671

H 2.764739 0.013358 3.085626

C 0.377368 3.063881 3.104630

H 0.230815 2.791797 0.981969

C 0.930062 2.604182 4.303256

H 2.218963 1.134003 5.212083

H -0.282198 3.928218 3.102695

H 0.699434 3.104840 5.239806

C 3.377651 -0.545513 0.636261

C 4.648716 0.048421 0.685949

C 3.253959 -1.917251 0.903668

C 5.773053 -0.713475 1.004023

H 4.764693 1.105756 0.468429

C 4.380927 -2.674561 1.230077

H 2.282833 -2.394702 0.845697

C 5.640685 -2.076434 1.279439

H 6.752160 -0.242361 1.032847

H 4.271135 -3.736458 1.432340

H 6.517497 -2.670138 1.524823

**TS6B-2n**

P -1.881881 0.573082 -0.238897

C -1.744974 2.202739 -1.086538

C -1.420205 4.637808 -2.446750

C -2.361124 3.369782 -0.610663

C -0.961857 2.270201 -2.252010

C -0.803080 3.479613 -2.927269

C -2.198561 4.579616 -1.289633

H -2.967244 3.337030 0.288633

H -0.477566 1.372539 -2.627045

H -0.192693 3.517217 -3.825199

H -2.682556 5.476527 -0.911909

H -1.293584 5.580727 -2.971954

C -2.670391 -0.579895 -1.407885

C -3.084713 -2.531474 -3.329455

C -3.718407 -0.421642 -2.321848

C -1.798224 -1.693334 -1.465150

C -2.015817 -2.671497 -2.445057

C -3.938556 -1.420216 -3.270697

H -4.334011 0.474833 -2.312453

H -1.351207 -3.525118 -2.521191

H -4.761718 -1.329986 -3.974362

H -3.251148 -3.293212 -4.087416

C -3.012402 0.903101 1.178536

C -2.473831 1.291267 2.415530

C -4.404352 0.768798 1.049726

C -3.315339 1.556588 3.496821

H -1.399562 1.377351 2.538930

C -5.241627 1.029832 2.135703

H -4.834008 0.443991 0.107219

C -4.699049 1.427522 3.359574

H -2.885034 1.856718 4.448293

H -6.316967 0.918366 2.025271

H -5.352019 1.628154 4.204781

Br 1.602961 -2.179790 -2.017704

C -1.158231 -2.541174 0.262444

C -2.112368 -2.413763 1.287806

C -0.500501 -3.782029 0.127436

C -2.344993 -3.454803 2.189031

H -2.692084 -1.507922 1.396739

C -0.740297 -4.817776 1.032280

H 0.220629 -3.925675 -0.666647

C -1.656643 -4.662652 2.073942

H -3.078584 -3.313997 2.979467

H -0.199831 -5.754152 0.913429

H -1.843223 -5.473841 2.772731

Rh -0.080507 -0.893305 -0.524769

P 1.810038 0.363226 0.351731

C 2.810074 1.436628 -0.778679

C 3.585265 2.503646 -0.289320

C 2.805921 1.182178 -2.159044

C 4.336990 3.294075 -1.159352

H 3.597078 2.727175 0.772790

C 3.559825 1.976432 -3.026002

H 2.239334 0.341732 -2.547100

C 4.324303 3.033502 -2.531305

H 4.930357 4.114211 -0.763485

H 3.549018 1.761409 -4.091278

H 4.908712 3.650755 -3.208784

C 1.374119 1.545493 1.715190

C 1.488745 1.184161 3.066161

C 0.846514 2.810846 1.400796

C 1.093008 2.066203 4.075642

H 1.894308 0.214378 3.335319

C 0.457783 3.691959 2.410156

H 0.748324 3.116738 0.364101

C 0.579849 3.323363 3.752531

H 1.196149 1.769028 5.116062

H 0.058772 4.667214 2.144359

H 0.280354 4.011576 4.538511

C 3.025478 -0.769322 1.159382

C 4.395367 -0.480769 1.237319

C 2.542818 -1.952864 1.739866

C 5.263609 -1.355029 1.893359

H 4.791778 0.417648 0.776007

C 3.412181 -2.818485 2.404918

H 1.490488 -2.205428 1.652045

C 4.774214 -2.522129 2.482443

H 6.324748 -1.124176 1.938448

H 3.023807 -3.731971 2.847039

H 5.452895 -3.202083 2.990759

**INT6B-2n**

P 2.448813 0.053090 -0.022474

C 2.904876 1.623150 -0.859327

C 3.436281 3.999224 -2.231377

C 4.127534 1.805689 -1.520068

C 1.921393 2.634769 -0.887270

C 2.218810 3.825211 -1.575109

C 4.397772 2.987904 -2.208152

H 4.860416 1.003570 -1.517076

H 1.491455 4.631006 -1.577216

H 5.345248 3.114876 -2.724487

H 3.634028 4.929248 -2.757955

C 3.468862 -0.020202 1.516241

C 4.909603 -0.172135 3.924011

C 4.489953 0.901554 1.795325

C 3.168147 -1.014579 2.465405

C 3.891386 -1.090883 3.655661

C 5.204000 0.825166 2.994011

H 4.729732 1.683995 1.082550

H 2.362798 -1.716365 2.266564

H 3.651240 -1.866175 4.378421

H 5.989841 1.547947 3.197767

H 5.466516 -0.230836 4.855570

C 3.166113 -1.228232 -1.133860

C 2.590104 -1.392925 -2.404116

C 4.266430 -2.016954 -0.776838

C 3.109758 -2.324072 -3.300132

H 1.729810 -0.791323 -2.685484

C 4.781263 -2.955954 -1.675368

H 4.719910 -1.906583 0.202651

C 4.206823 -3.110769 -2.936369

H 2.655329 -2.441489 -4.280440

H 5.632195 -3.566236 -1.384342

H 4.608243 -3.842001 -3.632972

Br 0.224536 -2.331931 0.611980

C -0.517707 3.219663 -0.705417

C 0.566652 2.167625 1.222904

C -1.559125 3.631226 0.094840

C -0.532284 2.598939 2.025531

H 1.491384 1.946806 1.748253

C -1.564856 3.326805 1.477679

H -2.389928 4.179551 -0.336479

H -0.504752 2.393336 3.092620

H -2.386104 3.670756 2.099615

C 0.609418 2.513942 -0.171371

H -0.517870 3.468541 -1.763419

Rh 0.170452 0.182572 0.208210

P -2.273469 -0.174676 -0.018339

C -3.568381 1.144104 -0.261630

C -4.435609 1.556535 0.759078

C -3.671572 1.766962 -1.517581

C -5.382837 2.559582 0.530058

H -4.385979 1.087167 1.736032

C -4.621705 2.760153 -1.749598

H -3.012018 1.462911 -2.325464

C -5.481481 3.163239 -0.723437

H -6.051066 2.858462 1.333789

H -4.691968 3.219075 -2.732629

H -6.224318 3.936010 -0.903138

C -2.669793 -1.215988 -1.505943

C -1.641479 -1.755715 -2.291042

C -4.002297 -1.445463 -1.896056

C -1.935899 -2.508996 -3.430394

H -0.610260 -1.604470 -1.995476

C -4.295199 -2.206531 -3.027544

H -4.816704 -1.017963 -1.319330

C -3.260545 -2.739792 -3.800001

H -1.123478 -2.920015 -4.023895

H -5.331454 -2.376810 -3.308086

H -3.487893 -3.328920 -4.684793

C -2.942281 -1.104790 1.437267

C -3.745079 -2.248382 1.335803

C -2.605823 -0.629100 2.715328

C -4.214071 -2.889718 2.484474

H -3.993919 -2.656644 0.362695

C -3.085768 -1.262610 3.861680

H -1.951979 0.232815 2.810295

C -3.892467 -2.396848 3.749229

H -4.827517 -3.781709 2.386597

H -2.815757 -0.878939 4.842257

H -4.257943 -2.898772 4.641300

*^t^*BuOLi

C 0.632412 -1.109220 -0.942677

C 0.115528 0.000064 -0.000090

H 0.260405 -2.085251 -0.608299

H 0.261401 -0.936396 -1.960202

H 1.728930 -1.150849 -0.977489

C 0.630485 1.371803 -0.489058

C 0.630850 -0.261653 1.432300

H 0.257589 2.165577 0.169525

H 1.726891 1.424876 -0.507589

H 0.258592 1.569648 -1.501661

H 1.727324 -0.272201 1.487342

H 0.259095 0.516536 2.109835

H 0.258075 -1.228783 1.790828

O -1.271441 -0.000758 -0.000391

Li -2.874143 -0.001018 -0.000671

**INT7B-2n**

P 2.385682 0.432786 0.080612

C 2.854917 1.338077 -1.471703

C 3.479122 2.617233 -3.900339

C 2.661176 0.678090 -2.699240

C 3.357754 2.647978 -1.481060

C 3.665790 3.282374 -2.687773

C 2.979189 1.312658 -3.901071

H 2.273538 -0.337112 -2.706034

H 3.506242 3.181950 -0.548927

H 4.054004 4.297854 -2.675709

H 2.832263 0.784059 -4.839391

H 3.721131 3.111463 -4.837770

C 2.664317 1.692133 1.423050

C 3.092541 3.571339 3.478907

C 3.967343 2.084535 1.781401

C 1.586501 2.251339 2.120505

C 1.796580 3.185217 3.138283

C 4.178718 3.017337 2.796595

H 4.821860 1.651324 1.270629

H 0.576266 1.956901 1.869576

H 0.943531 3.606100 3.663960

H 5.193330 3.305547 3.059207

H 3.257985 4.294079 4.273675

C 3.840421 -0.671408 0.401839

C 3.832284 -1.424902 1.587698

C 4.926619 -0.796562 -0.472345

C 4.887825 -2.283032 1.890749

H 2.993864 -1.341645 2.274349

C 5.983241 -1.658941 -0.167577

H 4.950282 -0.228271 -1.396037

C 5.967294 -2.403622 1.011343

H 4.865266 -2.860872 2.811034

H 6.817903 -1.748309 -0.858083

H 6.789066 -3.075484 1.244607

P -1.295976 0.411746 0.117922

C -2.230112 -0.176472 -1.385244

C -2.095599 0.455220 -2.633322

C -2.974735 -1.372268 -1.326635

C -2.654262 -0.099934 -3.786024

H -1.557556 1.391334 -2.718200

C -3.529590 -1.927149 -2.483983

H -3.108504 -1.872009 -0.377355

C -3.364738 -1.299247 -3.721716

H -2.532000 0.411970 -4.736909

H -4.111488 -2.842694 -2.409220

H -3.798789 -1.731103 -4.619286

C -2.437102 0.287148 1.591959

C -1.712305 0.108488 2.785881

C -3.854385 0.366842 1.666771

C -2.328760 0.027347 4.031675

H -0.633225 0.004241 2.715415

C -4.456608 0.257422 2.937441

C -3.718137 0.097860 4.106029

H -1.728609 -0.107458 4.927271

H -5.539145 0.323321 2.996561

H -4.227543 0.028860 5.063532

C -1.145360 2.250328 -0.108666

C -1.793351 3.170110 0.734140

C -0.282108 2.753861 -1.097009

C -1.607292 4.544940 0.572035

H -2.440205 2.816538 1.529794

C -0.107149 4.127908 -1.267250

H 0.279162 2.070972 -1.724722

C -0.771438 5.029799 -0.434541

H -2.118835 5.235534 1.237558

H 0.566624 4.485918 -2.040414

H -0.628852 6.099708 -0.560290

O -0.926691 -2.519640 0.309507

C -0.820316 -3.549276 1.289595

C 0.324911 -3.281527 2.283381

C -2.159210 -3.622809 2.050707

C -0.563652 -4.895833 0.579146

H 1.289185 -3.255957 1.764536

H 0.175911 -2.323262 2.792693

H 0.370497 -4.069924 3.046123

H -2.980807 -3.825359 1.352340

H -2.151100 -4.423317 2.801741

H -2.367414 -2.675370 2.556885

H -0.522731 -5.729409 1.291925

H -1.375477 -5.110383 -0.131095

H 0.382305 -4.862383 0.028900

C -4.809600 0.532942 0.525987

C -4.767568 1.618646 -0.361042

C -5.857500 -0.395161 0.387002

C -5.732381 1.763996 -1.359028

H -3.987202 2.364772 -0.266694

C -6.819134 -0.253101 -0.612300

H -5.903919 -1.245669 1.061975

C -6.760411 0.829936 -1.491377

H -5.680154 2.616028 -2.031715

H -7.613273 -0.989629 -0.703764

H -7.509650 0.945524 -2.269995

Rh 0.529814 -0.953093 0.112210

Br 1.738538 -2.760690 -1.635820

Li -0.642889 -2.688342 -1.456572

**TS8B-2n**

P -2.291331 -0.456038 0.162433

C -3.061733 -1.706692 -0.966578

C -4.240905 -3.525907 -2.757267

C -2.805681 -1.620731 -2.343358

C -3.918365 -2.718326 -0.498593

C -4.502320 -3.621245 -1.388576

C -3.394110 -2.522908 -3.231962

H -2.152508 -0.835943 -2.711366

H -4.126234 -2.808396 0.563086

H -5.160862 -4.399163 -1.010922

H -3.188817 -2.438692 -4.295868

H -4.695813 -4.230230 -3.448966

C -2.101516 -1.403599 1.748791

C -1.723615 -2.805833 4.165200

C -2.416561 -0.809696 2.983168

C -1.594031 -2.713893 1.747946

C -1.410123 -3.407286 2.945540

C -2.227789 -1.504297 4.179041

H -2.822985 0.195523 3.014560

H -1.351820 -3.204527 0.811716

H -1.019896 -4.421039 2.919147

H -2.485937 -1.027693 5.121210

H -1.581366 -3.348255 5.096115

C -3.670803 0.712461 0.576688

C -3.332961 1.955489 1.137681

C -5.022664 0.405874 0.372591

C -4.327943 2.861374 1.505940

H -2.285662 2.216110 1.267382

C -6.016313 1.320741 0.729457

H -5.308039 -0.539704 -0.075714

C -5.673403 2.546828 1.300071

H -4.049674 3.816822 1.943272

H -7.060242 1.072466 0.556425

H -6.448820 3.256515 1.576247

P 1.227842 -0.436576 -0.046675

C 2.292501 0.117882 -1.447782

C 2.973040 -0.765062 -2.302135

C 2.310076 1.497800 -1.727773

C 3.663620 -0.277562 -3.410509

H 2.979668 -1.829639 -2.097718

C 3.002735 1.974036 -2.844278

H 1.782755 2.207550 -1.086266

C 3.677942 1.092184 -3.687624

H 4.195914 -0.970536 -4.056887

H 3.008326 3.041541 -3.049624

H 4.214129 1.466192 -4.556165

C 2.108566 -0.133470 1.560732

C 1.242862 0.143350 2.633768

C 3.505768 -0.153384 1.797009

C 1.720770 0.387882 3.919297

H 0.174975 0.175488 2.447166

C 3.967404 0.120940 3.096215

C 3.094673 0.384391 4.150248

H 1.022009 0.592680 4.725387

H 5.039686 0.117882 3.271561

H 3.489466 0.588227 5.142084

C 1.179293 -2.275453 -0.191664

C 1.664985 -3.133440 0.804616

C 0.575493 -2.835975 -1.330831

C 1.564344 -4.519971 0.659909

H 2.108694 -2.721933 1.704699

C 0.478432 -4.219464 -1.476295

H 0.173049 -2.182916 -2.099274

C 0.973404 -5.066472 -0.479892

H 1.945086 -5.170520 1.443114

H 0.007746 -4.634771 -2.363195

H 0.893402 -6.144616 -0.590845

C 4.564978 -0.437392 0.773284

C 4.962354 -1.751310 0.487956

C 5.264301 0.621975 0.175556

C 6.019298 -2.001382 -0.389879

H 4.447271 -2.582621 0.958871

C 6.317671 0.372333 -0.703978

H 4.969957 1.644031 0.395657

C 6.698554 -0.940567 -0.989972

H 6.314578 -3.026721 -0.597722

H 6.841603 1.205290 -1.164995

H 7.522054 -1.134576 -1.672203

Rh -0.594486 0.774638 -0.630488

C 0.881905 4.212815 0.894093

C 0.060871 3.923263 2.169384

C 2.367340 3.883408 1.163231

C 0.764770 5.718520 0.555780

H -0.991328 4.191220 2.007113

H 0.109372 2.855364 2.406766

H 0.424709 4.488096 3.038137

H 2.964724 4.082279 0.264914

H 2.778180 4.481975 1.986750

H 2.480683 2.825298 1.422076

H 1.139566 6.363495 1.361806

H 1.334707 5.943244 -0.354486

H -0.286859 5.984944 0.377056

O 0.396190 3.458880 -0.179817

Br -2.116430 2.012829 -2.423388

Li -0.826129 3.652451 -1.360944

**INT8B-2n**

P -1.920679 0.906996 0.332797

C -1.411062 1.120320 2.098351

C -0.645918 1.325879 4.797017

C -0.992806 -0.016780 2.812119

C -1.429161 2.362909 2.753057

C -1.047288 2.462671 4.092638

C -0.622510 0.087290 4.153919

H -0.957923 -0.991861 2.333680

H -1.730684 3.258171 2.221337

H -1.065508 3.432198 4.584037

H -0.308454 -0.805745 4.685976

H -0.350406 1.406198 5.839849

C -2.195998 2.622665 -0.302570

C -2.567106 5.225401 -1.299568

C -3.241471 3.431607 0.178871

C -1.349901 3.134915 -1.294946

C -1.531754 4.428751 -1.788871

C -3.423091 4.723305 -0.315552

H -3.922776 3.048405 0.932632

H -0.553503 2.507005 -1.680531

H -0.864780 4.809513 -2.557481

H -4.236923 5.335236 0.064341

H -2.712420 6.230820 -1.685769

C -3.651468 0.246731 0.447969

C -4.587670 0.567427 -0.549651

C -4.023790 -0.655462 1.456821

C -5.867369 0.014892 -0.527922

H -4.317631 1.254011 -1.345286

C -5.303451 -1.219794 1.467027

H -3.326642 -0.934221 2.238864

C -6.229210 -0.883842 0.479699

H -6.581098 0.282773 -1.302712

H -5.566376 -1.921889 2.252798

H -7.224822 -1.318956 0.492458

P 1.475170 0.317088 -0.337509

C 2.124972 -0.592592 1.125829

C 3.072994 -0.082084 2.025184

C 1.580073 -1.865572 1.361582

C 3.467603 -0.834496 3.131733

H 3.512695 0.895034 1.863280

C 1.977798 -2.617984 2.466648

H 0.809296 -2.264408 0.707499

C 2.923289 -2.101832 3.354495

H 4.204302 -0.427826 3.819482

H 1.513391 -3.584630 2.636391

H 3.229817 -2.680491 4.222259

C 2.404359 -0.270459 -1.845338

C 1.612519 -0.268454 -3.012869

C 3.720417 -0.791143 -1.923908

C 2.070538 -0.789448 -4.222786

H 0.609810 0.162895 -2.967137

C 4.152447 -1.336438 -3.145617

C 3.346217 -1.344503 -4.282787

H 1.429794 -0.769504 -5.099671

H 5.160366 -1.738463 -3.198928

H 3.720978 -1.769667 -5.209833

C 1.992817 2.077869 -0.122795

C 2.548593 2.803741 -1.189087

C 1.728059 2.750279 1.082429

C 2.843111 4.161779 -1.050372

H 2.758060 2.308166 -2.132263

C 2.027711 4.106397 1.219097

H 1.290429 2.217943 1.919896

C 2.586187 4.817003 0.154972

H 3.278451 4.704354 -1.885551

H 1.820646 4.606401 2.161463

H 2.818402 5.872914 0.264008

O -2.160472 -1.665570 -1.584998

C -2.502249 -1.959355 -2.933034

C -3.817018 -2.762535 -2.909004

C -2.706658 -0.660674 -3.733774

C -1.391555 -2.812345 -3.575905

H -3.682679 -3.697987 -2.350741

H -4.606507 -2.181593 -2.420495

H -4.149793 -3.022447 -3.921604

H -1.796565 -0.049124 -3.711909

H -2.956165 -0.872192 -4.780945

H -3.518509 -0.069197 -3.298597

H -1.638231 -3.091857 -4.608181

H -0.441637 -2.265253 -3.589795

H -1.241385 -3.731438 -2.997695

C 4.732668 -0.764382 -0.823358

C 5.332662 0.443168 -0.435778

C 5.193261 -1.960665 -0.253375

C 6.359452 0.454527 0.509803

H 5.000847 1.374928 -0.883876

C 6.211346 -1.947392 0.699951

H 4.739237 -2.901890 -0.550485

C 6.799621 -0.739903 1.082647

H 6.818947 1.397996 0.793139

H 6.546736 -2.881914 1.141428

H 7.598802 -0.731153 1.819045

Rh -0.636430 -0.396548 -0.948392

Br -1.748239 -3.840201 1.779168

Li -2.312238 -2.578204 -0.044966

**TS9A-2n**

P -2.085737 0.037374 0.676185

C -2.843700 -1.461252 1.450969

C -3.873376 -3.684031 2.831753

C -4.226793 -1.618648 1.618048

C -1.983753 -2.423480 2.003000

C -2.493282 -3.527085 2.685759

C -4.736474 -2.725128 2.301116

H -4.910753 -0.876193 1.223832

H -0.909479 -2.302743 1.913399

H -1.810030 -4.261744 3.103033

H -5.811499 -2.833100 2.418140

H -4.272660 -4.544731 3.361653

C -1.627678 0.977681 2.212289

C -0.894141 2.392346 4.527204

C -2.528855 1.110647 3.283031

C -0.353106 1.550851 2.326559

C 0.010001 2.260525 3.475267

C -2.163177 1.812533 4.429735

H -3.516990 0.664525 3.221398

H 0.360572 1.426940 1.516101

H 0.992939 2.718478 3.532371

H -2.870856 1.910185 5.248700

H -0.616939 2.948211 5.418533

C -3.493452 1.030234 -0.035883

C -3.569682 2.377557 0.364765

C -4.416899 0.562722 -1.012849

C -4.506626 3.263294 -0.166873

H -2.902360 2.761202 1.129391

C -5.370325 1.469643 -1.512266

C -5.419493 2.802510 -1.110493

H -4.502359 4.298200 0.161044

H -6.069635 1.113327 -2.263044

H -6.159845 3.471666 -1.540185

Br -0.100630 -2.680374 -0.959345

C 0.777261 -3.975445 0.206475

C 0.459456 -5.318198 0.017916

C 1.070426 -6.266482 0.841411

H -0.249492 -5.618976 -0.746465

C 2.269082 -4.512125 1.998437

C 1.972866 -5.865929 1.829998

H 2.975217 -4.190729 2.757837

H 2.443859 -6.608776 2.467399

H 0.834753 -7.318755 0.708075

C -0.369354 2.128675 -2.566552

C 0.265729 3.519898 -2.754926

C 0.486474 1.079579 -3.298278

C -1.796355 2.135413 -3.139263

H -0.303375 4.298169 -2.230898

H 1.292753 3.536144 -2.376309

H 0.288628 3.788931 -3.817796

H 0.060163 0.072132 -3.177779

H 0.521315 1.288906 -4.374278

H 1.515000 1.075235 -2.924608

H -1.788541 2.387323 -4.206870

H -2.269536 1.156307 -3.024660

H -2.416589 2.874977 -2.621269

C 1.670003 -3.548252 1.181287

H 1.904024 -2.497105 1.301870

C -4.492125 -0.824824 -1.567670

C -5.730876 -1.491980 -1.577572

C -3.394919 -1.460327 -2.166857

C -5.865653 -2.753510 -2.154764

H -6.593165 -1.016992 -1.116743

C -3.531289 -2.720255 -2.753251

H -2.430406 -0.960542 -2.176583

C -4.764073 -3.373407 -2.748467

H -6.831800 -3.251242 -2.141585

H -2.669218 -3.187313 -3.222277

H -4.867363 -4.354221 -3.204925

Rh -0.262494 -0.125367 -0.641480

P 3.164543 0.225562 -0.287956

C 4.146483 -0.803336 0.907082

C 5.158988 -1.696839 0.529256

C 3.812709 -0.706434 2.271272

C 5.826531 -2.464223 1.488337

H 5.436091 -1.790762 -0.515817

C 4.484969 -1.466063 3.228400

H 3.031056 -0.020019 2.588992

C 5.496705 -2.349943 2.839134

H 6.613076 -3.146301 1.175574

H 4.221905 -1.364308 4.278239

H 6.024445 -2.939884 3.583927

C 4.066378 -0.025300 -1.885032

C 5.023491 0.860421 -2.402314

C 3.719187 -1.158816 -2.642676

C 5.626838 0.611385 -3.637343

H 5.295219 1.750147 -1.843508

C 4.334419 -1.415073 -3.868218

H 2.958365 -1.842167 -2.271363

C 5.289563 -0.527705 -4.370088

H 6.363026 1.310539 -4.025506

H 4.058264 -2.299656 -4.436229

H 5.761310 -0.719220 -5.330187

C 3.637530 1.923848 0.267988

C 2.652727 2.922097 0.215479

C 4.906117 2.250560 0.779540

C 2.918135 4.220454 0.661566

H 1.671522 2.674433 -0.178172

C 5.176430 3.548018 1.215583

H 5.677359 1.488484 0.848365

C 4.183711 4.531838 1.160702

H 2.124716 4.963703 0.635979

H 6.161234 3.789234 1.608115

H 4.395433 5.537411 1.514585

O -0.403390 1.859890 -1.148615

Li -0.851648 3.280378 -0.075106

Br -0.950160 5.381690 0.783390

**INT9A-2n**

P -0.127167 0.720164 0.827996

C 1.070518 1.098216 2.183791

C 2.918759 1.793749 4.185817

C 0.734369 0.973463 3.540135

C 2.341249 1.590418 1.844548

C 3.257107 1.936063 2.837632

C 1.655366 1.315314 4.534296

H -0.250873 0.616957 3.824379

H 2.612773 1.716237 0.800993

H 4.235672 2.316382 2.557223

H 1.379302 1.211528 5.580189

H 3.634123 2.060129 4.959135

C -0.624167 2.426099 0.296997

C -1.442239 4.977288 -0.553877

C -0.494890 3.537813 1.147146

C -1.159899 2.612852 -0.988032

C -1.573421 3.879073 -1.405328

C -0.899448 4.804011 0.720904

H -0.080021 3.420391 2.142963

H -1.252335 1.762576 -1.655844

H -2.001230 4.001783 -2.396172

H -0.791688 5.654547 1.388879

H -1.761870 5.962990 -0.880901

C -1.634861 0.045705 1.685271

C -2.703403 0.918759 1.964597

C -1.784364 -1.337685 1.988952

C -3.896831 0.464400 2.526575

H -2.615230 1.970232 1.717228

C -2.996311 -1.767284 2.555190

C -4.043245 -0.886560 2.823572

H -4.708740 1.162826 2.700079

H -3.107884 -2.821732 2.790893

H -4.970424 -1.260499 3.247827

Rh 0.434803 -0.603442 -0.881188

Br 2.843034 -1.159556 -0.283125

C 4.072503 0.012150 -1.235254

C 3.562263 1.047441 -2.011953

C 4.463841 1.878307 -2.683454

H 2.487194 1.191001 -2.084108

C 6.323375 0.615529 -1.788567

C 5.839520 1.664582 -2.573930

H 7.392857 0.445018 -1.701836

H 6.533785 2.312989 -3.100419

H 4.083246 2.692628 -3.293722

C -1.458966 -1.222465 -2.886647

C -1.612574 -0.156332 -3.984239

C -0.115161 -1.967266 -3.050993

C -2.621113 -2.226053 -2.947242

H -2.547134 0.396687 -3.840370

H -0.778902 0.555083 -3.946034

H -1.633594 -0.609167 -4.983167

H -0.007054 -2.772879 -2.313328

H -0.025933 -2.418324 -4.046192

H 0.748148 -1.267728 -2.988030

H -2.648216 -2.741144 -3.915573

H -2.514401 -2.980336 -2.158939

H -3.581563 -1.714735 -2.810739

C 5.438410 -0.224119 -1.107660

H 5.807071 -1.040698 -0.495240

C -0.758077 -2.399951 1.746115

C 0.473802 -2.421932 2.417638

C -1.081719 -3.484270 0.911713

C 1.366179 -3.481635 2.238319

H 0.731715 -1.618263 3.098494

C -0.189946 -4.541082 0.730123

H -2.037109 -3.485540 0.394743

C 1.040857 -4.542349 1.390969

H 2.310327 -3.483291 2.776869

H -0.460004 -5.366966 0.077098

H 1.733524 -5.368894 1.256916

O -1.471946 -0.580603 -1.602086

Li -3.075138 -0.069419 -0.848935

Br -5.225265 0.562763 -1.214733

**TS10A-2n**

P 0.291575 0.777732 -0.317687

C 0.426364 2.576358 -0.713978

C 0.493695 5.308442 -1.391592

C 0.937841 2.995508 -1.952688

C -0.067073 3.548510 0.172760

C -0.025776 4.902375 -0.160491

C 0.970800 4.350615 -2.287373

H 1.315466 2.264471 -2.660036

H -0.479332 3.250844 1.131200

H -0.402445 5.639658 0.543361

H 1.374006 4.654834 -3.249472

H 0.523978 6.363158 -1.650746

C 0.798635 0.594303 1.446227

C 1.437491 0.194315 4.152422

C 1.508584 1.571209 2.162159

C 0.408645 -0.584507 2.106728

C 0.740054 -0.786974 3.446543

C 1.817031 1.373242 3.508036

H 1.842210 2.476937 1.671359

H -0.161307 -1.339399 1.574550

H 0.450095 -1.713349 3.933703

H 2.369657 2.137931 4.046804

H 1.688326 0.039724 5.198521

C 1.553847 -0.019551 -1.430583

C 1.006126 -0.601331 -2.594842

C 2.959534 -0.066061 -1.239524

C 1.806385 -1.235262 -3.544179

H -0.070074 -0.563860 -2.733018

C 3.739637 -0.731710 -2.200225

C 3.182515 -1.311644 -3.336688

H 1.352241 -1.673701 -4.428303

H 4.811530 -0.786633 -2.037603

H 3.821218 -1.821822 -4.052087

Rh -1.776335 -0.139016 -0.596800

Br -3.125238 2.105047 -0.588284

C -1.821531 -3.102049 -0.854384

C -2.892520 -2.763050 -1.909411

C -0.996555 -4.301107 -1.359802

C -2.458520 -3.451310 0.501223

H -3.493045 -1.893796 -1.613211

H -2.421530 -2.540355 -2.872993

H -3.585579 -3.601776 -2.049246

H -0.208427 -4.578101 -0.648904

H -1.638439 -5.179265 -1.498534

H -0.529245 -4.072085 -2.325919

H -3.161337 -4.288956 0.411632

H -1.677193 -3.736951 1.214487

H -2.999497 -2.593719 0.911933

C -3.666523 0.632643 0.638979

C -4.704355 -0.193694 0.179793

C -3.329513 0.699790 2.002233

C -5.342943 -1.038744 1.096503

H -5.020899 -0.153837 -0.856383

C -3.978490 -0.153106 2.888090

H -2.563427 1.384926 2.345429

C -4.984772 -1.023659 2.442296

H -6.137178 -1.692302 0.746833

H -3.699172 -0.135348 3.937762

H -5.492112 -1.673340 3.148879

C 3.716452 0.551880 -0.104593

C 4.016912 1.922194 -0.111623

C 4.253595 -0.264702 0.902590

C 4.828076 2.473047 0.882936

H 3.625163 2.555756 -0.902399

C 5.057970 0.292743 1.897432

H 4.022737 -1.327800 0.903008

C 5.348584 1.659253 1.891118

H 5.060258 3.534955 0.862082

H 5.462671 -0.347318 2.676867

H 5.984162 2.086368 2.662801

O -0.916268 -1.999194 -0.692715

Li 0.875729 -2.481213 -0.591290

Br 2.549801 -3.726667 0.355083

**INT10A-2n**

P 1.022352 -0.339566 -0.085694

C 1.868533 -1.280814 -1.408879

C 3.121267 -2.777577 -3.420685

C 3.097957 -1.909118 -1.163049

C 1.257909 -1.430742 -2.664987

C 1.888266 -2.168500 -3.665599

C 3.721148 -2.650695 -2.167382

H 3.562105 -1.836703 -0.185223

H 0.285182 -0.990708 -2.858682

H 1.407515 -2.276834 -4.633588

H 4.671331 -3.136142 -1.963637

H 3.605854 -3.358547 -4.200356

C 0.751811 1.365748 -0.686726

C 0.160211 3.954721 -1.581480

C 1.179094 1.789551 -1.955418

C 0.040100 2.257904 0.136616

C -0.257762 3.543161 -0.313784

C 0.885651 3.081458 -2.393209

H 1.743803 1.124281 -2.597618

H -0.324035 1.925837 1.101742

H -0.846363 4.206992 0.310689

H 1.220278 3.400168 -3.376388

H -0.089283 4.949979 -1.937509

C 2.151363 -0.269966 1.378265

C 1.946186 -1.264948 2.353506

C 3.134123 0.731006 1.608104

C 2.647394 -1.264117 3.557288

H 1.245993 -2.071118 2.157170

C 3.805521 0.721901 2.842888

C 3.566948 -0.249876 3.812489

H 2.466057 -2.048827 4.285729

H 4.554661 1.487733 3.021391

H 4.113066 -0.222546 4.751348

Rh -0.906353 -1.348666 0.607298

Br -0.006077 -3.674558 0.338741

C -2.485831 0.040822 2.646702

C -1.452842 -0.585133 3.601675

C -2.946354 1.396884 3.206301

C -3.690650 -0.903396 2.473596

H -1.134081 -1.582288 3.263371

H -0.566599 0.052770 3.680386

H -1.876285 -0.714897 4.604426

H -3.648733 1.892206 2.526169

H -3.443983 1.263483 4.174427

H -2.087188 2.061748 3.349832

H -4.176647 -1.112337 3.433862

H -4.437246 -0.461212 1.803887

H -3.379229 -1.866355 2.044429

C -1.976967 -1.235653 -1.073314

C -2.773977 -2.361209 -1.333702

C -2.136563 -0.079159 -1.846877

C -3.741760 -2.304757 -2.343142

H -2.639129 -3.278138 -0.771326

C -3.110451 -0.036368 -2.854974

H -1.514744 0.797859 -1.691976

C -3.916039 -1.146711 -3.102910

H -4.355952 -3.181380 -2.533467

H -3.241546 0.880834 -3.421326

H -4.674727 -1.109258 -3.879161

C 3.583381 1.773427 0.632401

C 4.311727 1.420884 -0.513745

C 3.403088 3.134781 0.922738

C 4.831233 2.404461 -1.356842

H 4.486801 0.374289 -0.739892

C 3.913631 4.116904 0.074415

H 2.847355 3.421050 1.811255

C 4.631215 3.754842 -1.067758

H 5.399794 2.112291 -2.235739

H 3.753366 5.165729 0.308658

H 5.035726 4.520699 -1.723841

O -1.882673 0.303979 1.362005

Li -2.887528 1.296460 0.136992

Br -4.200777 3.093224 -0.316575

**TS9B-2n**

P -2.258167 -0.283705 -0.080035

C -2.781074 0.079462 1.656010

C -3.469043 0.632380 4.327459

C -3.489509 -0.859854 2.420268

C -2.418503 1.299333 2.252363

C -2.767769 1.576229 3.573689

C -3.825856 -0.585238 3.747865

H -3.783719 -1.807065 1.981909

H -1.870975 2.044340 1.683940

H -2.484027 2.527846 4.014777

H -4.372157 -1.326026 4.325509

H -3.734477 0.845306 5.359440

C -3.065156 1.028865 -1.101076

C -4.299041 2.889496 -2.810440

C -4.031283 1.916907 -0.607818

C -2.727146 1.083814 -2.464455

C -3.341770 2.003661 -3.313389

C -4.639676 2.844243 -1.458068

H -4.315540 1.886612 0.438641

H -1.983295 0.397294 -2.859777

H -3.073503 2.027924 -4.366277

H -5.385219 3.527313 -1.059895

H -4.779408 3.606735 -3.470688

C -3.236993 -1.779077 -0.530586

C -4.502801 -1.651212 -1.123760

C -2.705374 -3.065986 -0.280586

C -5.243937 -2.771186 -1.494879

H -4.916059 -0.664091 -1.298855

C -3.465406 -4.182069 -0.681976

C -4.714237 -4.043335 -1.280364

H -6.219438 -2.647506 -1.956742

H -3.048742 -5.174358 -0.542631

H -5.266461 -4.927984 -1.585975

C -1.399807 -3.298357 0.390725

C -0.243072 -2.517071 0.151008

C -1.301097 -4.390674 1.278494

C 0.978462 -2.927619 0.711771

C -0.097579 -4.749226 1.874643

H -2.199120 -4.956570 1.509570

C 1.053890 -4.021620 1.572120

H 1.894770 -2.393071 0.501189

H -0.058210 -5.594143 2.556639

H 2.018457 -4.286445 1.995978

P 0.997062 1.553856 0.231998

C 0.089194 3.166235 0.111039

C -1.281316 5.602773 -0.235617

C -0.537643 3.473595 -1.108504

C -0.002940 4.092364 1.162365

C -0.685336 5.298552 0.989161

C -1.206062 4.685816 -1.285092

H -0.514386 2.757540 -1.924242

H 0.453705 3.874877 2.121537

H -0.746034 6.002350 1.815174

H -1.682719 4.900669 -2.236947

H -1.808179 6.543822 -0.368373

C 1.435744 1.373335 2.014071

C 2.092716 1.017234 4.721886

C 2.323318 2.256701 2.657374

C 0.895495 0.305145 2.743926

C 1.222278 0.129638 4.090971

C 2.644121 2.081473 4.002343

H 2.779560 3.070245 2.101408

H 0.232824 -0.399133 2.252566

H 0.803129 -0.709502 4.638472

H 3.335008 2.767374 4.484977

H 2.353723 0.875321 5.767115

C 2.634042 1.954146 -0.542971

C 3.795194 1.314421 -0.075305

C 2.738544 2.801386 -1.655553

C 5.025111 1.507639 -0.710630

H 3.765810 0.663603 0.795340

C 3.970755 3.003520 -2.281317

H 1.865178 3.321770 -2.034097

C 5.116784 2.355815 -1.815846

H 5.896480 0.980194 -0.334457

H 4.031367 3.672244 -3.136142

H 6.072441 2.511664 -2.308621

C 1.902537 -1.404757 -2.981377

C 1.011059 -0.541279 -3.891642

C 3.375716 -1.235017 -3.420220

C 1.517302 -2.890588 -3.112199

H -0.038102 -0.641901 -3.589035

H 1.298904 0.513506 -3.812098

H 1.093198 -0.844048 -4.943176

H 4.046006 -1.880620 -2.834365

H 3.511442 -1.514171 -4.471741

H 3.695074 -0.192709 -3.300937

H 1.688636 -3.252854 -4.133433

H 2.114414 -3.499897 -2.425200

H 0.460093 -3.049395 -2.873814

Rh -0.053543 -0.627067 -0.670326

H -0.477654 -1.998835 -1.360856

O 1.816589 -0.954228 -1.628733

Li 3.411358 -1.121385 -0.759495

Br 4.909983 -2.187274 0.598929

**INT9B-2n**

P -2.021349 0.371366 -0.601629

C -2.423780 1.835411 0.451554

C -2.927938 4.043274 2.119246

C -3.404072 1.764011 1.453551

C -1.700119 3.030096 0.295431

C -1.953697 4.124840 1.122016

C -3.651423 2.860838 2.282030

H -3.981455 0.855900 1.585787

H -0.944149 3.113912 -0.477702

H -1.383974 5.039377 0.983669

H -4.416925 2.789517 3.050172

H -3.124310 4.896918 2.762497

C -1.923374 1.057129 -2.304001

C -1.735549 2.010412 -4.936564

C -2.696530 2.156114 -2.719400

C -1.056427 0.445948 -3.221680

C -0.959566 0.924643 -4.530096

C -2.604550 2.624915 -4.029661

H -3.363556 2.649650 -2.018972

H -0.427995 -0.389516 -2.926239

H -0.261751 0.448840 -5.212473

H -3.207672 3.473981 -4.340502

H -1.661026 2.383571 -5.954700

C -3.543274 -0.665227 -0.565715

C -4.505299 -0.564650 -1.578572

C -3.689020 -1.633575 0.457770

C -5.607933 -1.417758 -1.612000

H -4.384859 0.175879 -2.361747

C -4.799907 -2.494201 0.390224

C -5.746645 -2.393680 -0.626865

H -6.338025 -1.329780 -2.411629

H -4.905127 -3.274937 1.136895

H -6.584040 -3.085863 -0.652691

C -2.749460 -1.749922 1.609330

C -1.350515 -1.550501 1.523451

C -3.303607 -2.066417 2.869754

C -0.570170 -1.726254 2.676352

C -2.519140 -2.224210 4.006691

H -4.381861 -2.166989 2.955510

C -1.137736 -2.061955 3.905942

H 0.505433 -1.609544 2.611047

H -2.982848 -2.464778 4.959456

H -0.499168 -2.189218 4.776503

P 1.676864 0.722251 0.734868

C 1.769114 2.339458 -0.160090

C 1.820039 4.751138 -1.603830

C 1.681344 2.332181 -1.563866

C 1.888296 3.568023 0.508429

C 1.911179 4.765337 -0.210518

C 1.713073 3.532064 -2.277223

H 1.620247 1.393320 -2.109743

H 1.958814 3.594540 1.590484

H 2.004354 5.708810 0.321492

H 1.648526 3.506027 -3.361286

H 1.836998 5.683912 -2.161525

C 1.650119 1.230885 2.513686

C 1.500161 2.077038 5.191186

C 2.817869 1.404546 3.274600

C 0.406493 1.483995 3.115506

C 0.333859 1.910558 4.442383

C 2.741459 1.821251 4.604756

H 3.789669 1.216873 2.829619

H -0.508906 1.346538 2.550335

H -0.637499 2.103009 4.889353

H 3.654281 1.949131 5.180733

H 1.443005 2.401788 6.226803

C 3.387467 0.032286 0.540266

C 3.684815 -1.192896 1.170133

C 4.388181 0.655761 -0.223229

C 4.952500 -1.766772 1.046629

H 2.919761 -1.702821 1.744885

C 5.651604 0.074507 -0.350004

H 4.180485 1.592006 -0.728342

C 5.938531 -1.135052 0.284158

H 5.165744 -2.708663 1.545075

H 6.408644 0.568561 -0.952225

H 6.921761 -1.585972 0.182254

C 1.122754 -3.964146 -0.172014

C -0.091462 -4.451891 -0.979118

C 2.409623 -4.460342 -0.872992

C 1.069491 -4.540184 1.254698

H -1.026578 -4.182916 -0.478083

H -0.091933 -4.001324 -1.977625

H -0.067498 -5.543121 -1.092003

H 3.302640 -4.120213 -0.331005

H 2.448851 -5.554949 -0.913103

H 2.459453 -4.097327 -1.909184

H 1.063962 -5.637368 1.238712

H 1.942133 -4.213291 1.833116

H 0.169778 -4.198702 1.774063

Rh -0.287789 -1.070995 -0.109318

H -1.221027 -2.068644 -0.820849

O 1.210641 -2.537322 -0.109175

Li 2.491119 -1.848038 -1.226479

Br 2.422751 -0.969002 -3.357064

**TS10B-2n**

P 2.171252 -0.487952 -0.021068

C 2.970400 -0.721068 -1.663673

C 4.126995 -1.030159 -4.200342

C 4.131944 -1.491189 -1.823959

C 2.392511 -0.110343 -2.787980

C 2.970671 -0.261686 -4.048476

C 4.705803 -1.643567 -3.087725

H 4.583770 -1.977442 -0.964571

H 1.489111 0.483475 -2.676891

H 2.512313 0.213449 -4.911333

H 5.602160 -2.247105 -3.202049

H 4.572129 -1.155195 -5.183686

C 2.988725 1.001296 0.694816

C 4.189049 3.247193 1.876429

C 4.140303 1.569788 0.129831

C 2.447823 1.567684 1.860486

C 3.045855 2.682637 2.448344

C 4.734746 2.688606 0.718723

H 4.572696 1.142341 -0.768967

H 1.551194 1.143221 2.301032

H 2.608195 3.115647 3.343031

H 5.625331 3.121699 0.271056

H 4.651513 4.119439 2.330483

C 2.794411 -1.858493 1.033563

C 3.836488 -1.640925 1.947843

C 2.192626 -3.137015 0.937358

C 4.285763 -2.661777 2.784644

H 4.303850 -0.664605 2.010923

C 2.648873 -4.142428 1.809367

C 3.677889 -3.914676 2.719520

H 5.092360 -2.471883 3.487059

H 2.165671 -5.113724 1.788438

H 3.995968 -4.714475 3.382982

C 1.127329 -3.440997 -0.051663

C 0.188218 -2.476868 -0.474867

C 1.050314 -4.742663 -0.590177

C -0.804567 -2.856226 -1.390551

C 0.057203 -5.101172 -1.497322

H 1.798343 -5.479333 -0.310883

C -0.887454 -4.155239 -1.895905

H -1.575277 -2.148227 -1.698839

H 0.023261 -6.113388 -1.890889

H -1.693042 -4.417846 -2.575248

P -0.847104 1.909056 -0.148632

C -0.071371 3.007649 -1.426212

C 1.196727 4.557996 -3.410006

C 1.258362 3.437273 -1.264440

C -0.750965 3.365690 -2.601542

C -0.119577 4.130886 -3.585235

C 1.882182 4.210653 -2.243738

H 1.809155 3.178666 -0.367336

H -1.778130 3.052594 -2.753049

H -0.665148 4.397202 -4.486642

H 2.907598 4.537191 -2.092606

H 1.684808 5.156628 -4.174365

C -2.636722 1.970206 -0.604288

C -5.337303 2.014688 -1.386488

C -3.506295 2.954315 -0.107393

C -3.142157 1.011404 -1.497280

C -4.484551 1.030029 -1.887534

C -4.845732 2.974143 -0.498370

H -3.140217 3.702777 0.587410

H -2.487023 0.241771 -1.903578

H -4.859386 0.249195 -2.539155

H -5.507854 3.739241 -0.101374

H -6.383772 2.024006 -1.677500

C -0.809160 2.891354 1.414379

C -1.253098 2.232304 2.574218

C -0.404238 4.230372 1.500367

C -1.279139 2.902347 3.797341

H -1.589423 1.200830 2.499201

C -0.426312 4.894864 2.730118

H -0.070425 4.759825 0.614079

C -0.859852 4.233515 3.880124

H -1.628892 2.384264 4.686497

H -0.108553 5.932940 2.784325

H -0.878053 4.753544 4.834304

C -2.222183 -1.799892 2.558977

C -1.109950 -1.594548 3.602875

C -3.586548 -1.460175 3.198117

C -2.222108 -3.267204 2.091739

H -0.129492 -1.838674 3.175774

H -1.085727 -0.556521 3.954700

H -1.263722 -2.242852 4.473930

H -4.407323 -1.625662 2.487853

H -3.782539 -2.091386 4.073686

H -3.612938 -0.410802 3.513193

H -2.410108 -3.944167 2.934998

H -3.000601 -3.443868 1.339800

H -1.258705 -3.529258 1.646082

Rh -0.069092 -0.502066 -0.093168

H -0.385409 -0.650622 1.407197

O -2.054175 -0.896645 1.450377

Li -3.392025 -1.202839 0.214990

Br -4.790938 -2.444181 -1.110375

**INT10B-2n**

P 1.629126 -0.645533 0.048703

C 2.610077 -0.738012 1.615980

C 4.018195 -0.893182 4.041599

C 3.928154 -1.218350 1.644412

C 2.009519 -0.329915 2.816416

C 2.707050 -0.412218 4.022241

C 4.627809 -1.293158 2.850764

H 4.413009 -1.525694 0.722342

H 0.998036 0.067155 2.789527

H 2.230074 -0.090582 4.944468

H 5.651048 -1.659887 2.858001

H 4.565214 -0.948684 4.979235

C 1.008113 -2.378732 -0.160250

C -0.058622 -4.954541 -0.546852

C 1.335194 -3.425724 0.714839

C 0.135788 -2.642336 -1.230604

C -0.387406 -3.919464 -1.426796

C 0.800922 -4.703582 0.523077

H 2.005872 -3.248291 1.549016

H -0.140921 -1.837924 -1.905119

H -1.060676 -4.101541 -2.260021

H 1.061567 -5.502461 1.212736

H -0.473295 -5.948477 -0.693053

C 2.904150 -0.474697 -1.271831

C 3.323517 -1.586282 -2.017071

C 3.424188 0.812956 -1.567361

C 4.229869 -1.455455 -3.069610

H 2.932331 -2.569572 -1.778651

C 4.316537 0.918427 -2.650247

C 4.716556 -0.190630 -3.392579

H 4.536643 -2.330861 -3.635655

H 4.683644 1.898736 -2.935346

H 5.400952 -0.060811 -4.227146

C 3.069694 2.027160 -0.778606

C 1.831025 2.156049 -0.102254

C 4.002978 3.082521 -0.703408

C 1.583336 3.351168 0.602194

C 3.726052 4.259389 -0.014064

H 4.978590 2.971109 -1.168169

C 2.498270 4.403969 0.634843

H 0.640486 3.466273 1.140127

H 4.470333 5.050442 0.029022

H 2.265748 5.316831 1.179023

P -2.103789 0.270995 -0.004705

C -2.892347 -0.744699 1.327338

C -4.004885 -2.352308 3.347996

C -2.171525 -1.825795 1.858227

C -4.176717 -0.475945 1.828436

C -4.727746 -1.274183 2.832649

C -2.726984 -2.626979 2.857268

H -1.173052 -2.040133 1.490509

H -4.745639 0.362629 1.437767

H -5.721311 -1.051481 3.213290

H -2.155966 -3.461544 3.255385

H -4.433999 -2.971428 4.131519

C -2.971409 1.902034 0.169975

C -4.101616 4.456760 0.513183

C -3.930047 2.385376 -0.732629

C -2.586012 2.721190 1.247942

C -3.149101 3.985563 1.420172

C -4.489133 3.654441 -0.561129

H -4.241184 1.771925 -1.572159

H -1.843022 2.360443 1.956413

H -2.841226 4.603444 2.259613

H -5.230037 4.014535 -1.270316

H -4.536951 5.444030 0.642403

C -2.818279 -0.423574 -1.565545

C -2.194307 -0.056429 -2.770524

C -3.917966 -1.292296 -1.610747

C -2.668946 -0.534464 -3.992337

H -1.323971 0.596063 -2.737680

C -4.385692 -1.779112 -2.834239

H -4.408541 -1.595329 -0.691056

C -3.765503 -1.400093 -4.026180

H -2.176606 -0.239255 -4.915215

H -5.236186 -2.455862 -2.853798

H -4.131199 -1.780577 -4.976382

Rh 0.179193 0.996128 -0.042194

*^t^*BuOH-LiBr

C -2.340820 0.005544 0.041231

C -3.343021 1.000213 -0.549288

C -2.957484 -1.381527 0.239265

C -1.713193 0.534780 1.330687

H -2.866106 1.968953 -0.728695

H -3.750092 0.632443 -1.499599

H -4.184769 1.149501 0.135081

H -2.209894 -2.088103 0.614009

H -3.781618 -1.336922 0.959004

H -3.361969 -1.771758 -0.703243

H -2.482548 0.670374 2.096971

H -0.968550 -0.165681 1.727485

H -1.230426 1.504544 1.163841

H -1.546922 -0.465432 -1.744876

O -1.217073 -0.121110 -0.899351

Li 0.599566 -0.000867 -0.480851

Br 2.755654 -0.002300 0.007029

**TS9C-2n**

P -2.031657 0.259053 0.352358

C -3.098625 1.690544 -0.083462

C -4.545664 3.998732 -0.757826

C -2.872463 2.342377 -1.304974

C -4.043278 2.222978 0.807456

C -4.764179 3.366974 0.469224

C -3.597344 3.486245 -1.643585

H -2.118465 1.960952 -1.986164

H -4.217098 1.747044 1.766714

H -5.496089 3.766633 1.165644

H -3.412614 3.980087 -2.593925

H -5.107111 4.892513 -1.016838

C -2.891625 -1.378834 0.358518

C -3.740163 -4.050334 0.644056

C -1.978217 -2.417507 0.627364

C -4.262144 -1.695630 0.203533

C -4.654851 -3.037706 0.366233

C -2.386214 -3.740175 0.767161

H -0.918662 -2.199964 0.730216

H -5.707521 -3.279082 0.246150

H -1.635354 -4.501915 0.954203

H -4.083869 -5.076032 0.748309

C -1.474771 0.559790 2.059477

C -0.116753 0.905084 1.910094

C 0.610613 1.266600 3.051869

C -1.330915 0.771543 4.444534

C -0.003324 1.192643 4.306402

H 1.646882 1.577427 2.983766

H -1.780627 0.706785 5.431645

H 0.569243 1.455623 5.192616

P 2.729487 0.718459 0.006231

C 3.668434 -0.273436 -1.247963

C 4.104151 -1.578574 -0.970329

C 3.877394 0.247806 -2.536084

C 4.732682 -2.342945 -1.959464

H 3.962144 -2.016032 0.011617

C 4.520923 -0.510083 -3.513894

H 3.546150 1.252909 -2.775974

C 4.948141 -1.810434 -3.230306

H 5.053338 -3.353203 -1.722621

H 4.684202 -0.084887 -4.500749

H 5.444678 -2.401987 -3.994596

C 3.346867 2.431672 -0.311151

C 4.711762 2.682928 -0.546672

C 2.453866 3.512743 -0.322869

C 5.167302 3.981525 -0.770792

H 5.418890 1.859121 -0.564037

C 2.911444 4.813780 -0.548382

H 1.394567 3.332502 -0.170733

C 4.267607 5.051086 -0.769905

H 6.224523 4.157313 -0.950496

H 2.203147 5.637708 -0.556711

H 4.623562 6.062261 -0.948013

C 3.490791 0.219374 1.617190

C 3.071488 -0.994120 2.191393

C 4.438629 0.999297 2.297888

C 3.616638 -1.430454 3.399083

H 2.321515 -1.614325 1.706189

C 4.965546 0.567264 3.517602

H 4.764635 1.949379 1.889280

C 4.562206 -0.650238 4.067424

H 3.289438 -2.379169 3.814524

H 5.694527 1.186011 4.034499

H 4.977931 -0.986115 5.013731

Rh 0.352504 0.436587 -0.067941

H 0.199288 1.848119 0.619478

C 0.027248 -0.808916 -3.032856

C 0.259059 0.563650 -3.690206

C 0.716379 -1.904161 -3.877544

C -1.479699 -1.116111 -2.975727

H -0.146041 1.357648 -3.051857

H 1.330189 0.749750 -3.816521

H -0.220783 0.627781 -4.674882

H 0.532115 -2.900511 -3.449997

H 0.334381 -1.921535 -4.905421

H 1.797887 -1.733650 -3.917407

H -1.901792 -1.199114 -3.985232

H -1.660750 -2.057439 -2.447081

H -2.022965 -0.323289 -2.452781

O 0.631206 -0.835405 -1.744352

Li 1.371431 -2.371175 -1.139391

Br 1.519162 -4.105641 0.349890

C -2.080682 0.447577 3.311646

H -3.109287 0.108391 3.406426

C -5.352297 -0.732083 -0.139828

C -5.433077 -0.145545 -1.411458

C -6.383646 -0.485208 0.779657

C -6.504842 0.681954 -1.746708

H -4.657952 -0.352661 -2.143808

C -7.455327 0.344483 0.446110

H -6.339604 -0.947701 1.762495

C -7.518117 0.931833 -0.818654

H -6.550799 1.127409 -2.736812

H -8.242619 0.527548 1.172989

H -8.353696 1.574874 -1.082109

**INT9C-2n**

P 2.080327 0.053278 0.423896

C 2.705221 1.244345 -0.833022

C 3.258697 3.184325 -2.792018

C 1.868273 2.359288 -1.017539

C 3.858289 1.103982 -1.646599

C 4.101735 2.085168 -2.621702

C 2.134803 3.329398 -1.982437

H 0.975663 2.462017 -0.410449

H 4.981506 1.978074 -3.250577

H 1.437127 4.153259 -2.101851

H 3.481473 3.919404 -3.560912

C 3.153610 0.197169 1.906290

C 4.666692 0.439874 4.261287

C 3.273609 -0.886848 2.791049

C 3.792015 1.407835 2.221779

C 4.546350 1.524872 3.390367

C 4.027525 -0.764574 3.958774

H 2.791394 -1.832083 2.558723

H 3.706496 2.257067 1.549697

H 5.038639 2.466206 3.619039

H 4.119195 -1.614386 4.629986

H 5.254476 0.532748 5.170385

C 2.027202 -1.682297 -0.116738

C 0.637644 -1.923252 -0.225503

C 2.993091 -2.674603 -0.316101

C 0.234847 -3.218525 -0.592385

C 2.559444 -3.952967 -0.669131

H 4.050396 -2.462191 -0.199083

C 1.191111 -4.215728 -0.808342

H -0.816068 -3.471406 -0.697169

H 3.285315 -4.744107 -0.837859

H 0.864933 -5.216575 -1.083852

P -2.533572 -0.926900 -0.170334

C -3.856191 0.293717 0.224811

C -4.751241 0.124739 1.288298

C -3.918598 1.472086 -0.539819

C -5.690177 1.118470 1.581989

H -4.725470 -0.779559 1.887999

C -4.848118 2.467285 -0.237149

H -3.250782 1.623093 -1.385949

C -5.738397 2.290330 0.826324

H -6.383461 0.971834 2.406133

H -4.858583 3.377432 -0.829105

H -6.464373 3.063198 1.063050

C -2.841672 -1.255907 -1.962246

C -4.155957 -1.280886 -2.465000

C -1.776490 -1.479079 -2.846932

C -4.395209 -1.539393 -3.814266

H -4.993977 -1.081208 -1.804203

C -2.019834 -1.734207 -4.198471

H -0.756301 -1.450812 -2.483064

C -3.326664 -1.767608 -4.684493

H -5.415947 -1.550771 -4.186606

H -1.182151 -1.897698 -4.870719

H -3.513266 -1.959984 -5.737536

C -3.043639 -2.471615 0.707442

C -2.583451 -2.662341 2.021193

C -3.854337 -3.458677 0.126700

C -2.941402 -3.800560 2.744283

H -1.939873 -1.918760 2.482086

C -4.203653 -4.601955 0.848470

H -4.208327 -3.343812 -0.892186

C -3.752278 -4.774403 2.158001

H -2.579295 -3.928639 3.760680

H -4.828813 -5.359208 0.383030

H -4.025737 -5.665362 2.716625

Rh -0.331354 -0.236851 0.418515

H -0.216299 0.256312 -1.017752

C -0.925058 1.916709 2.702495

C -0.762999 0.645392 3.559216

C 0.328397 2.802987 2.838281

C -2.154478 2.704878 3.201367

H -1.680224 0.045930 3.522035

H 0.076131 0.036750 3.191884

H -0.557131 0.880791 4.610690

H 0.226440 3.698333 2.212848

H 0.484657 3.129393 3.874438

H 1.223735 2.262506 2.519357

H -2.053951 2.987362 4.257093

H -2.278859 3.629728 2.622801

H -3.063660 2.105989 3.084342

C 4.833342 -0.026380 -1.567961

C 4.984074 -0.888742 -2.666757

C 5.673245 -0.204863 -0.457830

C 5.931587 -1.912263 -2.649145

H 4.342376 -0.757637 -3.533697

C 6.624299 -1.227888 -0.440957

H 5.593425 0.468009 0.389201

C 6.754783 -2.086865 -1.534104

H 6.026375 -2.573394 -3.506518

H 7.269718 -1.345382 0.425598

H 7.496290 -2.881251 -1.520354

O -1.146934 1.569647 1.345026

Li -1.649316 2.780703 0.121042

Br -1.566299 4.430559 -1.451967

**TS10C-2n**

P -2.067809 0.279485 0.192487

C -3.129614 -0.247651 1.620577

C -4.424285 -1.048542 3.998877

C -2.494716 -0.164974 2.871809

C -4.448802 -0.771351 1.563028

C -5.062781 -1.163799 2.765415

C -3.126574 -0.548174 4.053795

H -1.470970 0.187562 2.907393

H -6.072043 -1.563137 2.718687

H -2.602536 -0.462796 5.001674

H -4.938190 -1.356605 4.905510

C -2.835120 1.770545 -0.548257

C -3.788770 4.157680 -1.677269

C -2.607585 2.056726 -1.903192

C -3.531723 2.701473 0.238683

C -4.009211 3.885517 -0.325374

C -3.085925 3.241657 -2.462665

H -2.053053 1.356196 -2.520704

H -3.711598 2.498022 1.290803

H -4.549953 4.596309 0.293973

H -2.900445 3.451318 -3.512266

H -4.155170 5.082528 -2.114159

C -1.933422 -1.075751 -1.008843

C -0.603736 -1.513031 -0.812405

C -2.802144 -1.666475 -1.934223

C -0.186907 -2.655563 -1.517110

C -2.347465 -2.777758 -2.642296

H -3.801340 -1.277443 -2.095793

C -1.057969 -3.277061 -2.414906

H 0.814478 -3.055936 -1.402135

H -2.998657 -3.263907 -3.364097

H -0.720384 -4.153835 -2.963496

P 2.494304 -0.951573 -0.027224

C 3.893483 0.114549 0.564511

C 4.594683 0.960637 -0.309115

C 4.197582 0.158363 1.937961

C 5.579598 1.827080 0.180095

H 4.382040 0.955792 -1.372205

C 5.191560 1.008452 2.417641

H 3.661071 -0.480116 2.632752

C 5.884266 1.849382 1.539831

H 6.101653 2.480284 -0.512643

H 5.422793 1.019185 3.479632

H 6.654087 2.517142 1.916816

C 2.766663 -2.509263 0.927342

C 4.055018 -3.037570 1.127811

C 1.665676 -3.181473 1.478617

C 4.232353 -4.218600 1.848265

H 4.922786 -2.518374 0.731721

C 1.846708 -4.363805 2.200793

H 0.670626 -2.769884 1.341930

C 3.127435 -4.885359 2.384637

H 5.233641 -4.614713 1.994991

H 0.983943 -4.871986 2.623056

H 3.267456 -5.803536 2.948977

C 2.945932 -1.336957 -1.777611

C 2.589245 -0.399453 -2.762854

C 3.608304 -2.512059 -2.163952

C 2.912231 -0.627135 -4.101538

H 2.077945 0.522968 -2.495177

C 3.913391 -2.741962 -3.507044

H 3.879289 -3.258025 -1.424487

C 3.569948 -1.799688 -4.477882

H 2.642418 0.116815 -4.845645

H 4.420979 -3.659965 -3.791783

H 3.811402 -1.979626 -5.522147

Rh 0.326018 -0.030433 0.219822

H 0.727476 0.241823 1.756686

C 0.889821 2.543488 2.550013

C 0.921779 1.873414 3.932723

C 1.933171 3.679017 2.507324

C -0.497175 3.116817 2.236453

H 0.218707 1.033478 3.979098

H 1.924632 1.485879 4.141755

H 0.651611 2.581881 4.724683

H 1.915530 4.196591 1.538932

H 1.725386 4.429618 3.279359

H 2.940457 3.282400 2.676586

H -0.762567 3.907846 2.948693

H -0.514449 3.540092 1.226873

H -1.266167 2.342350 2.294090

C -5.245507 -1.007178 0.318440

C -5.737950 0.037923 -0.476769

C -5.594942 -2.326492 -0.018227

C -6.534444 -0.231604 -1.592242

H -5.509434 1.065189 -0.219200

C -6.390116 -2.595646 -1.131813

H -5.222775 -3.144639 0.592235

C -6.859849 -1.547366 -1.926605

H -6.906670 0.592791 -2.194514

H -6.639864 -3.623934 -1.379525

H -7.479682 -1.753981 -2.794986

O 1.306281 1.590792 1.555355

Li 2.146316 2.266725 0.052008

Br 2.153584 3.415623 -1.915040

**INT10C-2n**

P -2.073880 -0.760741 0.006718

C -3.251241 -0.883823 -1.407523

C -2.749837 -0.660811 -2.698881

C -4.602776 -1.226910 -1.251666

C -3.579331 -0.791356 -3.813318

H -1.705346 -0.383832 -2.820201

C -5.432416 -1.351945 -2.367353

H -5.009660 -1.393759 -0.258390

C -4.922661 -1.136262 -3.649586

H -3.178298 -0.615858 -4.808239

H -6.477856 -1.618914 -2.234317

H -5.570850 -1.232710 -4.516784

C -1.291956 -2.398075 0.223277

C 0.084826 -2.076156 0.183563

C -1.758166 -3.712855 0.311951

C 1.009019 -3.131944 0.275760

C -0.821319 -4.745365 0.406882

H -2.823674 -3.931967 0.296206

C 0.547655 -4.447562 0.392807

H 2.078554 -2.945969 0.237699

H -1.152630 -5.777876 0.481538

H 1.269443 -5.259260 0.460636

C -3.162401 -0.406025 1.457936

C -3.432585 -1.407388 2.402559

C -3.738839 0.877514 1.630305

C -4.274439 -1.169413 3.490015

H -2.977832 -2.385292 2.283673

C -4.597068 1.091580 2.719575

C -4.867482 0.082434 3.643678

H -4.467325 -1.962296 4.207839

H -5.036980 2.076856 2.849204

H -5.528275 0.279655 4.483690

Rh 0.211766 -0.125104 -0.088792

P 2.487479 0.200011 -0.006991

C 3.350737 0.055973 1.623805

C 4.484970 0.817745 1.951121

C 2.841842 -0.844565 2.572573

C 5.103698 0.670523 3.193764

H 4.879200 1.536465 1.238138

C 3.466216 -0.994755 3.812311

H 1.948757 -1.416500 2.338864

C 4.597407 -0.238739 4.125384

H 5.978227 1.269364 3.435071

H 3.060456 -1.695635 4.537111

H 5.077889 -0.350367 5.093919

C 3.560635 -0.752791 -1.175999

C 2.964580 -1.220209 -2.359087

C 4.918292 -1.025504 -0.947701

C 3.714172 -1.925112 -3.301773

H 1.905088 -1.038003 -2.521792

C 5.664268 -1.739581 -1.887578

H 5.394759 -0.690101 -0.031885

C 5.065719 -2.186896 -3.067302

H 3.238716 -2.280478 -4.212170

H 6.713519 -1.948582 -1.695305

H 5.648133 -2.744457 -3.796237

C 2.793035 1.969725 -0.465510

C 3.526646 2.364146 -1.593467

C 2.183525 2.962039 0.324813

C 3.649990 3.717032 -1.921810

H 4.005146 1.615295 -2.216574

C 2.314066 4.312047 -0.000526

H 1.614327 2.673608 1.205700

C 3.046275 4.693312 -1.128598

H 4.223330 4.005793 -2.799001

H 1.846627 5.065866 0.627927

H 3.145597 5.744579 -1.385397

C -3.471957 2.023486 0.708566

C -4.524724 2.626856 0.003957

C -2.179581 2.558097 0.571438

C -4.291856 3.723583 -0.827634

H -5.528418 2.221336 0.099330

C -1.946413 3.654837 -0.259091

H -1.359320 2.112618 1.128871

C -3.001760 4.239148 -0.963517

H -5.118985 4.171772 -1.372013

H -0.938776 4.051413 -0.353573

H -2.819559 5.091664 -1.612504

**INT11B-2n**

P 2.229678 -0.362613 0.325311

C 3.620206 -0.241262 -0.891266

C 5.691008 0.027775 -2.770689

C 4.849470 -0.888027 -0.693770

C 3.440250 0.538048 -2.044200

C 4.470978 0.676570 -2.974475

C 5.877220 -0.755547 -1.630143

H 5.002458 -1.502822 0.188233

H 2.483584 1.025345 -2.211268

H 4.316775 1.283074 -3.863177

H 6.821800 -1.268864 -1.469011

H 6.490632 0.126949 -3.500275

C 2.637232 0.958833 1.559657

C 3.118579 2.961939 3.475955

C 3.835626 1.688820 1.542716

C 1.685675 1.246079 2.552789

C 1.924845 2.235900 3.505546

C 4.071429 2.685735 2.494055

H 4.585781 1.483231 0.786161

H 0.748937 0.697273 2.567048

H 1.172400 2.447562 4.260319

H 5.002929 3.245488 2.465224

H 3.303100 3.740176 4.211916

C 2.586434 -1.914756 1.262564

C 3.188555 -1.880344 2.528486

C 2.193802 -3.157058 0.703360

C 3.390103 -3.047232 3.266999

H 3.502569 -0.930992 2.948373

C 2.389500 -4.315631 1.475969

C 2.976257 -4.268555 2.739013

H 3.854764 -2.995402 4.248019

H 2.048303 -5.269813 1.086937

H 3.100977 -5.185262 3.309883

C 1.611452 -3.260670 -0.661950

C 0.826825 -2.225495 -1.223287

C 1.869165 -4.423913 -1.418442

C 0.326936 -2.418223 -2.526034

C 1.350837 -4.592910 -2.697524

H 2.514138 -5.196149 -1.007994

C 0.563634 -3.582661 -3.255584

H -0.278909 -1.635713 -2.984544

H 1.571608 -5.496625 -3.259749

H 0.150317 -3.695441 -4.255662

P -0.881823 1.741515 -0.309013

C -0.032771 3.231762 -1.023180

C 1.360723 5.452510 -2.056000

C 1.234721 3.580662 -0.526537

C -0.590413 4.016763 -2.044364

C 0.103980 5.115119 -2.558755

C 1.921899 4.683061 -1.034078

H 1.686525 2.991959 0.263741

H -1.569443 3.775276 -2.444075

H -0.343592 5.707554 -3.352763

H 2.899872 4.934401 -0.632147

H 1.899330 6.307105 -2.457087

C -2.394099 1.620912 -1.380577

C -4.604748 1.338800 -3.102325

C -3.645230 2.147839 -1.028917

C -2.269427 0.946803 -2.608430

C -3.362843 0.809252 -3.464415

C -4.741825 2.006026 -1.883600

H -3.766913 2.670633 -0.085772

H -1.304139 0.535471 -2.896766

H -3.245454 0.287149 -4.410351

H -5.704089 2.420809 -1.594566

H -5.458302 1.232050 -3.766631

C -1.566498 2.395815 1.285520

C -2.021784 1.460360 2.230063

C -1.644888 3.761979 1.595135

C -2.551140 1.882752 3.450204

H -1.958176 0.399137 2.004474

C -2.166928 4.182606 2.820854

H -1.293523 4.500823 0.881761

C -2.621698 3.245511 3.750342

H -2.902300 1.146207 4.168234

H -2.217023 5.244576 3.047703

H -3.027186 3.574673 4.703601

Rh 0.181830 -0.437684 -0.491889

Br -2.024946 -2.141866 0.518418

C -3.953997 -2.251684 0.585509

C -4.715453 -1.602481 -0.383998

C -4.548147 -2.988545 1.609294

C -6.107670 -1.699014 -0.320799

H -4.234292 -1.033590 -1.171743

C -5.941274 -3.076239 1.657518

H -3.934273 -3.486613 2.352556

C -6.723005 -2.433369 0.695163

H -6.708374 -1.194441 -1.072817

H -6.412586 -3.650627 2.450716

H -7.806282 -2.504728 0.737143

**TS12B-2n**

P -1.602798 0.960745 -0.192818

C -2.007787 2.173365 1.144862

C -2.531664 4.032453 3.185904

C -3.296782 2.700675 1.314482

C -0.985842 2.588933 2.011884

C -1.244343 3.516477 3.021415

C -3.556353 3.622287 2.331283

H -4.100706 2.387081 0.655644

H 0.015145 2.185280 1.893623

H -0.440725 3.830113 3.682436

H -4.561687 4.016139 2.456358

H -2.735993 4.747664 3.978416

C -1.167659 2.039253 -1.631718

C -0.505368 3.572716 -3.894394

C -1.334688 3.431275 -1.622422

C -0.664969 1.423194 -2.790759

C -0.339777 2.184677 -3.913814

C -1.004715 4.192202 -2.747563

H -1.720665 3.926611 -0.737711

H -0.531291 0.345941 -2.812955

H 0.044187 1.692930 -4.803827

H -1.140111 5.270592 -2.724669

H -0.249995 4.165808 -4.768726

C -3.242290 0.283225 -0.692541

C -3.909804 0.736561 -1.839406

C -3.812353 -0.746728 0.091891

C -5.124242 0.177035 -2.237547

H -3.480096 1.535564 -2.433095

C -5.029865 -1.305333 -0.337898

C -5.680675 -0.855580 -1.484524

H -5.622196 0.542078 -3.131733

H -5.458723 -2.125704 0.229264

H -6.616250 -1.317185 -1.790240

C -3.183676 -1.222065 1.353125

C -1.779459 -1.301813 1.530008

C -4.045854 -1.593756 2.408249

C -1.319086 -1.794474 2.769927

C -3.561083 -2.069085 3.620245

H -5.118303 -1.473414 2.280502

C -2.181344 -2.178831 3.796804

H -0.251361 -1.896551 2.936808

H -4.249026 -2.340923 4.416567

H -1.773432 -2.558794 4.731159

P 2.210839 0.172262 0.104743

C 2.501953 2.002993 0.261451

C 2.889441 4.792335 0.420902

C 2.073822 2.851495 -0.774286

C 3.125793 2.580378 1.379777

C 3.311563 3.962593 1.459887

C 2.274032 4.230641 -0.699186

H 1.588009 2.435656 -1.650188

H 3.473006 1.953489 2.193119

H 3.795406 4.387096 2.335890

H 1.935821 4.862825 -1.515147

H 3.037554 5.867224 0.483241

C 2.968295 -0.447627 1.684403

C 4.004428 -1.317558 4.155319

C 4.289867 -0.905888 1.784582

C 2.178164 -0.421572 2.846409

C 2.691833 -0.847492 4.071729

C 4.800063 -1.344324 3.009450

H 4.926780 -0.925635 0.906938

H 1.151613 -0.069734 2.787413

H 2.062724 -0.818840 4.957542

H 5.824296 -1.704064 3.064833

H 4.403582 -1.659467 5.106582

C 3.497452 -0.315616 -1.155458

C 3.680749 -1.686060 -1.422803

C 4.267710 0.609597 -1.876070

C 4.603928 -2.112373 -2.378031

H 3.101697 -2.424901 -0.877256

C 5.185305 0.180285 -2.838974

H 4.161689 1.671869 -1.687468

C 5.357930 -1.179780 -3.094176

H 4.730049 -3.176007 -2.562700

H 5.770373 0.916606 -3.384243

H 6.073667 -1.511345 -3.841727

Rh -0.218499 -0.743373 0.314017

Br 0.686623 -3.253791 0.297355

C -0.445106 -2.515632 -1.308064

C -1.757522 -3.006010 -1.395092

C 0.280788 -2.209215 -2.469431

C -2.379666 -3.055633 -2.640713

H -2.285521 -3.307623 -0.499220

C -0.363479 -2.272171 -3.708105

H 1.321948 -1.919117 -2.409231

C -1.694244 -2.685035 -3.802801

H -3.412159 -3.389576 -2.697837

H 0.194846 -2.010895 -4.604334

H -2.184647 -2.741400 -4.770374

**INT12B-2n**

P -1.646521 1.013469 -0.043018

C -1.968088 1.683603 1.644784

C -2.360834 2.653853 4.251000

C -3.262606 1.820384 2.163474

C -0.870683 2.042840 2.446093

C -1.067267 2.529984 3.738406

C -3.455191 2.299969 3.460913

H -4.120653 1.548189 1.557982

H 0.140335 1.963996 2.055457

H -0.209100 2.809480 4.343570

H -4.464248 2.394876 3.852816

H -2.514162 3.025305 5.260601

C -1.160136 2.506623 -1.020805

C -0.575891 4.752451 -2.612488

C -1.198484 3.798350 -0.475319

C -0.822555 2.355188 -2.377424

C -0.534922 3.469715 -3.165450

C -0.906460 4.911707 -1.266685

H -1.461734 3.943882 0.566134

H -0.809221 1.367490 -2.825314

H -0.286202 3.334024 -4.214678

H -0.943968 5.904576 -0.826576

H -0.357930 5.620738 -3.228849

C -3.284891 0.572724 -0.750591

C -3.902848 1.368438 -1.726067

C -3.892705 -0.638025 -0.343392

C -5.095433 0.970902 -2.329482

H -3.450947 2.306786 -2.024589

C -5.083966 -1.027038 -0.979995

C -5.678063 -0.241461 -1.964345

H -5.554775 1.600753 -3.086099

H -5.536701 -1.974792 -0.705518

H -6.592770 -0.578504 -2.444830

C -3.364379 -1.444921 0.789758

C -1.989105 -1.658747 1.045052

C -4.320140 -1.949747 1.698213

C -1.638987 -2.355331 2.218306

C -3.952783 -2.646045 2.843689

H -5.372216 -1.748405 1.516129

C -2.598986 -2.840172 3.108597

H -0.595671 -2.557717 2.427588

H -4.714032 -3.016941 3.524923

H -2.280635 -3.381239 3.996491

P 2.274750 0.061617 0.092915

C 2.623882 1.875750 0.304078

C 3.093229 4.647106 0.526553

C 2.400145 2.735353 -0.785642

C 3.079090 2.433231 1.509706

C 3.305935 3.808280 1.620023

C 2.641563 4.104088 -0.679008

H 2.045535 2.332907 -1.729607

H 3.272291 1.793547 2.364084

H 3.662654 4.217839 2.561712

H 2.467058 4.746306 -1.537241

H 3.277940 5.714791 0.610748

C 2.970452 -0.637173 1.658153

C 3.930541 -1.653345 4.097889

C 4.278461 -1.128101 1.759044

C 2.148646 -0.667078 2.796408

C 2.625764 -1.162920 4.009249

C 4.752479 -1.636437 2.970673

H 4.927770 -1.124735 0.889784

H 1.123715 -0.306637 2.740416

H 1.973967 -1.179582 4.878720

H 5.766736 -2.022532 3.029683

H 4.300941 -2.052082 5.038529

C 3.511356 -0.409114 -1.206709

C 3.315635 -1.601317 -1.923926

C 4.640246 0.377945 -1.494601

C 4.228051 -1.991917 -2.905775

H 2.461969 -2.231263 -1.696037

C 5.547389 -0.016317 -2.479592

H 4.814059 1.302204 -0.953669

C 5.342472 -1.201157 -3.189515

H 4.060730 -2.917659 -3.449692

H 6.414748 0.604432 -2.689559

H 6.048708 -1.505786 -3.957672

Rh -0.338071 -0.923656 0.088068

Br 0.878090 -3.187435 0.245607

C -0.791992 -1.474206 -1.803284

C -1.780409 -2.414383 -2.115355

C 0.005212 -0.962827 -2.833703

C -1.989301 -2.799468 -3.443408

H -2.374421 -2.866001 -1.331807

C -0.208180 -1.351898 -4.162573

H 0.815970 -0.275703 -2.612541

C -1.212740 -2.267047 -4.473864

H -2.761105 -3.532921 -3.665734

H 0.425548 -0.941884 -4.945967

H -1.378714 -2.573028 -5.503528

**TS13B-2n**

P -1.240427 1.200221 -0.163006

C -1.199079 2.214689 1.384911

C -1.189847 3.672761 3.788820

C -2.007877 3.355449 1.534429

C -0.413796 1.801419 2.467531

C -0.406747 2.526045 3.661054

C -1.994264 4.084273 2.723193

H -2.671806 3.661702 0.731684

H 0.173811 0.895076 2.379560

H 0.206162 2.187024 4.491723

H -2.623984 4.964505 2.822127

H -1.186572 4.236104 4.718191

C -0.603700 2.265546 -1.531656

C 0.307378 3.740544 -3.745807

C -0.506241 3.663220 -1.465010

C -0.228351 1.616540 -2.720085

C 0.222105 2.348262 -3.819751

C -0.056684 4.394296 -2.566893

H -0.763135 4.185658 -0.550266

H -0.274190 0.532491 -2.773581

H 0.513781 1.828532 -4.728111

H 0.015067 5.476711 -2.499093

H 0.660441 4.312026 -4.600076

C -3.053756 1.170476 -0.481619

C -3.675697 1.994196 -1.429453

C -3.822634 0.270419 0.286942

C -5.056899 1.941849 -1.621879

H -3.081082 2.681132 -2.022672

C -5.210422 0.230044 0.073352

C -5.824128 1.053995 -0.867786

H -5.525581 2.586041 -2.360840

H -5.806248 -0.482425 0.635735

H -6.898397 0.992278 -1.019871

C -3.211543 -0.613713 1.310637

C -2.025399 -1.365166 1.093744

C -3.875090 -0.723406 2.548413

C -1.599889 -2.239560 2.120819

C -3.414123 -1.552620 3.564029

H -4.761645 -0.117066 2.712984

C -2.269158 -2.319665 3.339158

H -0.729735 -2.863044 1.949152

H -3.943607 -1.604778 4.511278

H -1.893169 -2.988510 4.109836

P 2.066383 -0.149853 0.050762

C 2.571224 1.600668 0.464369

C 3.321077 4.251056 1.078398

C 2.577027 2.597106 -0.526494

C 2.947718 1.961123 1.768878

C 3.316756 3.272645 2.072572

C 2.950945 3.906738 -0.222310

H 2.309429 2.351216 -1.547973

H 2.972321 1.213049 2.553460

H 3.609972 3.523936 3.088533

H 2.952696 4.655977 -1.009263

H 3.615294 5.270603 1.313301

C 2.984670 -1.073116 1.369423

C 4.328495 -2.326026 3.497308

C 4.341730 -1.400639 1.259350

C 2.304979 -1.393980 2.554679

C 2.974132 -2.006247 3.614155

C 5.006488 -2.028337 2.315085

H 4.883528 -1.188255 0.344463

H 1.240469 -1.194755 2.635163

H 2.431163 -2.250013 4.523587

H 6.056449 -2.287896 2.207640

H 4.848056 -2.813653 4.317896

C 2.995109 -0.408533 -1.533492

C 2.425398 -1.174755 -2.561822

C 4.260319 0.168337 -1.745554

C 3.106824 -1.365654 -3.765720

H 1.461141 -1.645927 -2.408734

C 4.942222 -0.030619 -2.946565

H 4.711968 0.788824 -0.978478

C 4.366342 -0.798224 -3.960848

H 2.649970 -1.966576 -4.547422

H 5.921472 0.418977 -3.088980

H 4.896538 -0.950756 -4.897386

Rh -0.319245 -0.923180 -0.076353

Br 0.955942 -3.210343 -0.167030

C -1.996978 -2.099361 -0.797579

C -2.198212 -3.481404 -0.610358

C -2.511927 -1.528449 -1.975905

C -2.863217 -4.251928 -1.559087

H -1.835125 -3.965264 0.286125

C -3.160538 -2.311064 -2.940318

H -2.405266 -0.473614 -2.181258

C -3.345611 -3.674473 -2.737361

H -3.000109 -5.314628 -1.374264

H -3.522332 -1.834639 -3.848710

H -3.863846 -4.279068 -3.477239

**INT13B-2n**

P -2.558270 -0.111809 -0.055356

C -2.868916 1.641562 -0.491372

C -3.208150 4.328042 -1.164762

C -4.087132 2.289059 -0.237066

C -1.791195 2.343800 -1.066562

C -1.994391 3.691674 -1.413496

C -4.260605 3.631255 -0.566766

H -4.894372 1.740129 0.240154

H -1.190026 4.246616 -1.883647

H -5.203698 4.128468 -0.357345

H -3.330303 5.373055 -1.437264

C -3.598777 -1.115599 -1.205614

C -5.068691 -2.731132 -2.975150

C -4.495214 -0.540107 -2.119417

C -3.437716 -2.513661 -1.196579

C -4.175021 -3.311632 -2.070875

C -5.223848 -1.345226 -2.999461

H -4.628529 0.536388 -2.150952

H -2.725358 -2.965056 -0.511556

H -4.043010 -4.390217 -2.050113

H -5.912329 -0.884105 -3.702909

H -5.636673 -3.356139 -3.659366

C -3.366448 -0.286022 1.589942

C -2.733070 0.292958 2.700985

C -4.598867 -0.927641 1.766807

C -3.325203 0.239239 3.960453

H -1.768867 0.777698 2.575594

C -5.187801 -0.986554 3.033176

H -5.099117 -1.389299 0.921689

C -4.555097 -0.403167 4.130227

H -2.823379 0.690203 4.812516

H -6.141602 -1.492426 3.157993

H -5.013650 -0.451493 5.114328

Br -0.575897 -2.465193 1.159400

C 0.738872 2.458386 -1.388621

C -0.537412 0.552936 -2.314149

C 1.732858 2.187238 -2.313301

C 0.534204 0.297280 -3.221198

H -1.508163 0.158720 -2.602174

C 1.630824 1.124259 -3.241464

H 2.645552 2.772198 -2.291904

H 0.431965 -0.514986 -3.936262

H 2.439661 0.956358 -3.946046

C -0.486180 1.676169 -1.416998

Rh -0.286492 -0.368410 -0.266546

P 2.138661 -0.842322 0.057150

C 3.583809 0.051066 -0.709605

C 4.309582 -0.474009 -1.788270

C 3.959457 1.305912 -0.199400

C 5.384401 0.231380 -2.337526

H 4.048681 -1.444384 -2.197209

C 5.036635 2.005965 -0.742043

H 3.411483 1.739737 0.631648

C 5.753767 1.471390 -1.815888

H 5.938570 -0.198750 -3.168050

H 5.312855 2.971151 -0.325435

H 6.595230 2.014925 -2.237656

C 2.646310 -0.700746 1.839981

C 1.692786 -0.418811 2.828226

C 3.993289 -0.829673 2.226238

C 2.071768 -0.278045 4.165424

H 0.649258 -0.332716 2.550570

C 4.369377 -0.699007 3.562954

H 4.756247 -1.019749 1.477953

C 3.408084 -0.421153 4.537490

H 1.316072 -0.061503 4.915959

H 5.414603 -0.807701 3.840758

H 3.701554 -0.314381 5.578662

C 2.486853 -2.594723 -0.436300

C 3.245433 -3.488379 0.330716

C 1.960358 -3.036655 -1.661109

C 3.482603 -4.787943 -0.122483

H 3.639194 -3.183439 1.293765

C 2.209091 -4.330597 -2.119211

H 1.338152 -2.368277 -2.248997

C 2.971765 -5.211514 -1.349624

H 4.064239 -5.471105 0.491163

H 1.793436 -4.654346 -3.070020

H 3.157326 -6.223669 -1.699515

C 0.952313 3.537814 -0.387961

C 1.583272 4.743106 -0.744601

C 0.567099 3.363898 0.953971

C 1.821464 5.737691 0.203852

H 1.868150 4.909085 -1.780068

C 0.806872 4.357843 1.901843

H 0.098565 2.428953 1.248162

C 1.434776 5.549820 1.532264

H 2.303413 6.664079 -0.098282

H 0.508318 4.197505 2.934613

H 1.620101 6.324148 2.271858

**INT14B-2n**

P 2.962250 0.141248 0.237320

C 3.637672 1.229592 -1.107756

C 4.579336 2.795387 -3.251273

C 3.479126 0.795608 -2.437053

C 4.264568 2.462580 -0.872397

C 4.730845 3.238881 -1.937006

C 3.953999 1.570447 -3.496515

H 2.993221 -0.155568 -2.636169

H 4.383411 2.830123 0.140518

H 5.214110 4.191442 -1.733981

H 3.830407 1.214015 -4.515847

H 4.944414 3.399390 -4.077979

C 3.279453 1.086624 1.806815

C 3.758017 2.436100 4.231553

C 4.592280 1.377513 2.222105

C 2.218876 1.463190 2.638439

C 2.453923 2.135558 3.840791

C 4.828584 2.052177 3.419027

H 5.434390 1.061586 1.613467

H 1.205191 1.218956 2.347851

H 1.614628 2.420021 4.469978

H 5.849565 2.268429 3.722747

H 3.942978 2.956832 5.167502

C 4.269111 -1.163398 0.431552

C 4.099085 -2.102742 1.462135

C 5.402839 -1.252245 -0.384534

C 5.041404 -3.108427 1.668632

H 3.222872 -2.049279 2.102691

C 6.346900 -2.261462 -0.175883

H 5.551848 -0.541191 -1.189847

C 6.169362 -3.191463 0.847752

H 4.893062 -3.829454 2.468302

H 7.220327 -2.319171 -0.820320

H 6.903260 -3.977253 1.006074

P -0.652084 0.671320 0.059402

C -1.463215 0.570829 -1.617042

C -1.234718 1.495114 -2.647764

C -2.250076 -0.562399 -1.908000

C -1.742527 1.278736 -3.931087

H -0.670068 2.399865 -2.459702

C -2.758409 -0.775034 -3.192178

H -2.434233 -1.289383 -1.126438

C -2.497867 0.141352 -4.215293

H -1.547381 2.011301 -4.710016

H -3.378093 -1.648090 -3.384243

H -2.893019 -0.022104 -5.214040

C -2.009283 0.490553 1.339340

C -1.510379 -0.068313 2.532263

C -3.375681 0.890686 1.300213

C -2.287159 -0.202960 3.679782

H -0.481765 -0.417540 2.539510

C -4.140283 0.740158 2.474438

C -3.616101 0.214114 3.650409

H -1.858183 -0.636672 4.578984

H -5.178792 1.054179 2.451842

H -4.246201 0.125526 4.531301

C -0.165524 2.452935 0.273093

C -0.651726 3.236927 1.334474

C 0.793365 3.022476 -0.581491

C -0.217795 4.551835 1.513221

H -1.374399 2.822491 2.028916

C 1.214954 4.342546 -0.411722

H 1.233627 2.429850 -1.374841

C 0.709292 5.114202 0.634918

H -0.610043 5.135738 2.341832

H 1.954295 4.755025 -1.092093

H 1.042771 6.139313 0.771882

O -0.690683 -2.362808 -0.233175

C -0.736788 -3.555046 0.549917

C 0.381738 -3.596478 1.607468

C -2.108594 -3.614083 1.248529

C -0.592078 -4.777358 -0.382573

H 1.366256 -3.574817 1.128801

H 0.303559 -2.740304 2.287326

H 0.315490 -4.513780 2.207279

H -2.909420 -3.619589 0.499000

H -2.211296 -4.521090 1.858337

H -2.251928 -2.742770 1.894538

H -0.689130 -5.721561 0.168337

H -1.380658 -4.761513 -1.149080

H 0.382927 -4.766736 -0.880552

C -4.098751 1.499231 0.132181

C -5.317840 0.961084 -0.364557

C -3.639142 2.711958 -0.403925

C -5.982570 1.646399 -1.397475

C -4.320967 3.380903 -1.417871

H -2.736812 3.154380 -0.001023

C -5.499382 2.840095 -1.925981

H -6.893984 1.213872 -1.800072

H -3.930461 4.319460 -1.801849

H -6.040519 3.339331 -2.725329

Rh 0.946885 -0.964322 -0.067948

Br 2.121988 -2.627743 -1.967410

Li -0.230634 -2.287029 -1.975536

C -5.969031 -0.283243 0.145386

C -7.346454 -0.269413 0.430873

C -5.274126 -1.491388 0.315888

C -8.005456 -1.419122 0.866880

H -7.900645 0.659355 0.325175

C -5.932095 -2.642442 0.747995

H -4.211655 -1.532384 0.107844

C -7.300469 -2.613054 1.025184

H -9.069166 -1.378599 1.087139

H -5.370358 -3.564687 0.868661

H -7.810399 -3.510730 1.364515

**TS15B-2n**

P -2.556113 -0.889110 0.166525

C -3.164795 -2.097246 -1.101832

C -4.101951 -3.871060 -3.073771

C -3.036958 -1.784090 -2.463167

C -3.767246 -3.315998 -0.742382

C -4.232810 -4.194852 -1.721464

C -3.504654 -2.664347 -3.441151

H -2.579059 -0.843234 -2.749766

H -3.866707 -3.586290 0.304286

H -4.695338 -5.133101 -1.426149

H -3.401010 -2.402890 -4.490933

H -4.463278 -4.556858 -3.835732

C -2.017066 -2.020697 1.538635

C -1.108684 -3.690792 3.620051

C -2.347126 -1.747076 2.877110

C -1.223716 -3.147651 1.262348

C -0.776631 -3.974014 2.294224

C -1.896395 -2.574760 3.907329

H -2.967147 -0.891048 3.120011

H -0.960791 -3.392277 0.239272

H -0.164967 -4.839585 2.055264

H -2.171783 -2.349209 4.934414

H -0.760497 -4.336621 4.421793

C -4.092956 -0.132849 0.882855

C -3.952594 1.044427 1.637867

C -5.370443 -0.684016 0.720037

C -5.063573 1.642064 2.234093

H -2.969586 1.499618 1.737202

C -6.483245 -0.075483 1.306192

H -5.507695 -1.580090 0.124437

C -6.333617 1.083765 2.067708

H -4.936961 2.548563 2.820668

H -7.469109 -0.509950 1.162381

H -7.201100 1.553928 2.523338

P 0.845246 0.053525 -0.216638

C 1.599863 1.050029 -1.571103

C 2.459749 0.500872 -2.536045

C 1.163134 2.381176 -1.714446

C 2.878041 1.268464 -3.622322

H 2.803543 -0.523561 -2.439552

C 1.580969 3.137435 -2.812504

H 0.501246 2.839257 -0.974499

C 2.434768 2.585896 -3.767503

H 3.550548 0.835609 -4.358604

H 1.232479 4.161844 -2.914585

H 2.755876 3.176606 -4.621731

C 1.773110 0.277943 1.385490

C 0.991890 -0.084430 2.499899

C 3.084137 0.768743 1.607747

C 1.464216 0.019717 3.804093

H -0.019814 -0.437349 2.337346

C 3.520300 0.914830 2.938937

C 2.737577 0.539826 4.027221

H 0.829023 -0.278711 4.633150

H 4.507120 1.337746 3.108946

H 3.117752 0.664406 5.037720

C 1.187988 -1.695151 -0.692189

C 1.990572 -2.558113 0.063693

C 0.558838 -2.190398 -1.848829

C 2.156360 -3.891224 -0.324614

H 2.489033 -2.199925 0.957002

C 0.730689 -3.517234 -2.238276

H -0.079159 -1.533870 -2.432517

C 1.527526 -4.374338 -1.471711

H 2.787677 -4.543663 0.271298

H 0.231512 -3.884391 -3.130934

H 1.655819 -5.411695 -1.770070

C 4.047805 1.264345 0.562545

C 3.984348 2.632517 0.254081

C 5.063823 0.476052 -0.030653

C 4.874012 3.230042 -0.634636

H 3.211000 3.232840 0.723844

C 5.953797 1.100712 -0.925935

C 5.867577 2.455270 -1.230873

H 4.789914 4.290530 -0.854495

H 6.746727 0.503205 -1.366751

H 6.579134 2.902691 -1.919733

Rh -1.273931 0.821482 -0.516799

C -0.656577 4.336609 1.338278

C -0.211003 3.458338 2.524392

C 0.508224 5.264577 0.923283

C -1.848817 5.215474 1.790344

H -1.032469 2.803343 2.838635

H 0.625765 2.819176 2.227783

H 0.100804 4.058595 3.389550

H 0.198127 5.907391 0.090039

H 0.845584 5.907516 1.747048

H 1.361714 4.667491 0.581898

H -1.601415 5.865609 2.640028

H -2.177127 5.862204 0.963496

H -2.693721 4.579956 2.089305

O -1.038031 3.543872 0.249302

Br -3.238745 1.793559 -2.032625

Li -2.488517 3.529062 -0.668635

C 5.278024 -0.982288 0.215232

C 5.461604 -1.842643 -0.882896

C 5.401077 -1.528482 1.504188

C 5.755664 -3.193643 -0.703527

H 5.362448 -1.443548 -1.888520

C 5.692737 -2.881564 1.685496

H 5.290366 -0.889119 2.371806

C 5.873952 -3.720376 0.584012

H 5.888588 -3.834920 -1.570893

H 5.793734 -3.276140 2.693376

H 6.109911 -4.771580 0.727850

**INT15B-2n**

P -2.650391 0.422293 0.107520

C -2.450053 1.097253 1.818239

C -2.102704 2.021705 4.451896

C -1.758948 0.319475 2.764263

C -2.951855 2.350096 2.207365

C -2.778101 2.806898 3.515535

C -1.595955 0.777376 4.072716

H -1.347116 -0.648750 2.492619

H -3.469330 2.979699 1.492586

H -3.172410 3.779204 3.800009

H -1.062868 0.157305 4.787324

H -1.969314 2.379252 5.469559

C -3.436877 1.791706 -0.856863

C -4.599612 3.874842 -2.344913

C -4.746384 2.225225 -0.580343

C -2.727021 2.410803 -1.894036

C -3.303094 3.447532 -2.631992

C -5.320986 3.260356 -1.317733

H -5.322765 1.745713 0.205215

H -1.722678 2.069320 -2.121403

H -2.737241 3.916691 -3.432241

H -6.334422 3.582559 -1.093658

H -5.050178 4.678655 -2.921102

C -4.046457 -0.791061 0.265507

C -4.897092 -1.016995 -0.830402

C -4.213753 -1.572186 1.419438

C -5.898551 -1.984017 -0.766169

H -4.779721 -0.433352 -1.737637

C -5.210347 -2.552534 1.475245

H -3.572937 -1.433173 2.282661

C -6.057256 -2.758447 0.386925

H -6.552609 -2.136810 -1.620752

H -5.315148 -3.150333 2.375897

H -6.834340 -3.516492 0.433874

P 0.800262 0.998187 -0.267870

C 1.571438 0.609402 1.359852

C 2.270112 1.532817 2.153307

C 1.389303 -0.696713 1.843787

C 2.786227 1.148953 3.390963

H 2.417469 2.549388 1.809650

C 1.906369 -1.080504 3.081719

H 0.806589 -1.416997 1.275021

C 2.609615 -0.156990 3.856256

H 3.328919 1.874139 3.991508

H 1.723689 -2.091654 3.432848

H 3.010793 -0.448728 4.823470

C 2.006983 0.597523 -1.639149

C 1.341896 0.189314 -2.816900

C 3.425403 0.565493 -1.622431

C 2.027075 -0.259147 -3.944546

H 0.250549 0.237523 -2.845309

C 4.094651 0.092242 -2.765899

C 3.418164 -0.319010 -3.911124

H 1.475901 -0.563764 -4.829644

H 5.179787 0.065989 -2.749245

H 3.977975 -0.672323 -4.772698

C 0.624880 2.838411 -0.313837

C 0.925222 3.562865 -1.479284

C 0.077733 3.525610 0.782779

C 0.696597 4.938891 -1.542493

H 1.347295 3.053253 -2.340368

C -0.147150 4.901619 0.716918

H -0.169808 2.992168 1.693986

C 0.161202 5.613240 -0.443756

H 0.943258 5.482686 -2.450708

H -0.566064 5.415328 1.578049

H -0.014055 6.684574 -0.491669

O -1.809136 -2.312467 -1.290777

C -1.794448 -2.946319 -2.563172

C -2.699106 -4.189776 -2.462268

C -2.345965 -1.997351 -3.642962

C -0.359550 -3.383185 -2.916841

H -2.318061 -4.879016 -1.697683

H -3.716832 -3.896104 -2.183604

H -2.741735 -4.735870 -3.412853

H -1.738940 -1.086007 -3.703734

H -2.350182 -2.474508 -4.630837

H -3.371333 -1.701932 -3.398229

H -0.317582 -3.903701 -3.882170

H 0.308958 -2.515758 -2.972650

H 0.029112 -4.058948 -2.146092

C 4.298648 1.099743 -0.528212

C 4.281097 2.483518 -0.291987

C 5.258802 0.300599 0.140955

C 5.165893 3.084371 0.601536

H 3.572328 3.099364 -0.836558

C 6.134331 0.924642 1.046166

C 6.094730 2.297265 1.280964

H 5.131602 4.159297 0.757873

H 6.850770 0.307653 1.580993

H 6.788222 2.746820 1.986713

Rh -0.854895 -0.517901 -0.836474

Br -1.042048 -3.637735 2.428548

Li -1.831859 -2.956521 0.387816

C 5.407317 -1.167692 -0.080299

C 4.319550 -2.049910 0.017016

C 6.677045 -1.703344 -0.357238

C 4.494958 -3.422351 -0.158269

H 3.337493 -1.662359 0.263081

C 6.852167 -3.075587 -0.539658

H 7.529104 -1.034060 -0.445639

C 5.760888 -3.940483 -0.440740

H 3.640793 -4.087286 -0.062084

H 7.841737 -3.467426 -0.760620

H 5.895688 -5.009950 -0.578573

**TS16B-2n**

P -1.303799 1.481239 -0.366402

C -1.899614 1.960972 1.319351

C -2.760775 2.743379 3.880369

C -3.261770 2.184217 1.578681

C -0.976740 2.143251 2.360015

C -1.403406 2.534417 3.629761

C -3.687995 2.569250 2.851610

H -3.991158 2.061072 0.784657

H 0.081025 1.988863 2.182663

H -0.673108 2.668846 4.422851

H -4.746112 2.734293 3.036480

H -3.093915 3.040260 4.871142

C -0.849714 3.098203 -1.139947

C -0.187791 5.494784 -2.451316

C -1.069857 4.333994 -0.516919

C -0.289328 3.077587 -2.429135

C 0.036349 4.266510 -3.080966

C -0.738733 5.524485 -1.169845

H -1.502221 4.372944 0.477259

H -0.118067 2.125519 -2.923733

H 0.461938 4.233902 -4.080417

H -0.915762 6.474988 -0.673704

H 0.062848 6.422054 -2.959768

C -2.844051 1.023277 -1.266056

C -3.526506 1.968939 -2.048365

C -3.303450 -0.313786 -1.215195

C -4.635407 1.608479 -2.811218

H -3.183094 2.997414 -2.069030

C -4.404672 -0.658525 -2.021101

C -5.062678 0.281214 -2.808875

H -5.146818 2.355217 -3.412294

H -4.741360 -1.688449 -2.033263

H -5.905431 -0.026983 -3.421628

C -2.641999 -1.383188 -0.411028

C -1.232563 -1.542621 -0.428858

C -3.428277 -2.344285 0.291181

C -0.657968 -2.713790 0.087295

C -2.812191 -3.475422 0.842341

C -1.441291 -3.676040 0.716997

H 0.406967 -2.888873 0.022608

H -3.422394 -4.191591 1.384921

H -0.963168 -4.563372 1.122274

P 2.272855 0.633591 0.584744

C 2.593847 2.435189 0.885835

C 3.107459 5.188220 1.191441

C 2.535249 3.306074 -0.215530

C 2.892140 2.973111 2.147926

C 3.142610 4.338677 2.297851

C 2.802787 4.667565 -0.067115

H 2.265630 2.924920 -1.195559

H 2.929232 2.329902 3.019908

H 3.370372 4.735619 3.283674

H 2.752056 5.319932 -0.933783

H 3.307728 6.249613 1.310505

C 2.197337 -0.128919 2.264036

C 2.019983 -1.337581 4.794307

C 3.312697 -0.159119 3.122715

C 1.000573 -0.723481 2.688659

C 0.912827 -1.323314 3.947423

C 3.221304 -0.755128 4.379236

H 4.258335 0.266832 2.801138

H 0.142106 -0.732845 2.025381

H -0.018574 -1.790561 4.253677

H 4.091658 -0.776595 5.029380

H 1.955164 -1.812594 5.769398

C 3.906305 0.026400 -0.055173

C 4.350364 -1.264535 0.279465

C 4.671757 0.785518 -0.952238

C 5.521716 -1.785909 -0.277380

H 3.801802 -1.882009 0.986524

C 5.848456 0.267874 -1.497880

H 4.367457 1.791764 -1.219805

C 6.276244 -1.019613 -1.167368

H 5.825513 -2.793425 -0.008848

H 6.431835 0.877465 -2.183140

H 7.190054 -1.420375 -1.597244

C 1.669071 -1.718248 -3.142880

C 1.731945 -0.366932 -3.876166

C 2.960024 -2.513507 -3.447764

C 0.459954 -2.536806 -3.633192

H 0.816300 0.204207 -3.682801

H 2.588728 0.215437 -3.518297

H 1.828483 -0.497985 -4.961349

H 2.935779 -3.510408 -2.984087

H 3.083021 -2.671725 -4.525574

H 3.841895 -1.974433 -3.081525

H 0.554846 -2.779682 -4.698865

H 0.382891 -3.473910 -3.071256

H -0.475547 -1.983024 -3.498972

Rh 0.254602 -0.161286 -0.829621

H -0.795833 -0.809489 -1.832883

O 1.629828 -1.513421 -1.729938

Li 2.580770 -2.781274 -0.833288

Br 2.820659 -4.698236 0.381563

C -4.896079 -2.198458 0.533569

C -5.773505 -3.250888 0.222528

C -5.425728 -1.052388 1.148574

C -7.137909 -3.154098 0.499849

H -5.380424 -4.145595 -0.253305

C -6.788059 -0.956586 1.429807

H -4.757591 -0.242450 1.425014

C -7.651551 -2.005043 1.103349

H -7.798970 -3.977817 0.242869

H -7.176017 -0.063519 1.913483

H -8.713366 -1.928763 1.321843

**INT16B-2n**

P -1.025646 1.538540 -0.229172

C -1.528604 1.927044 1.505458

C -2.227788 2.584994 4.148182

C -2.866302 2.171443 1.849590

C -0.544307 2.030085 2.500667

C -0.891374 2.357809 3.811278

C -3.212977 2.494404 3.164086

H -3.636859 2.125798 1.087143

H 0.499547 1.872671 2.252494

H -0.113560 2.435773 4.565877

H -4.254043 2.680040 3.414777

H -2.498550 2.837298 5.169940

C -0.217076 3.092232 -0.790326

C 1.054367 5.414452 -1.716285

C -0.517118 4.341142 -0.219733

C 0.728828 3.020230 -1.823801

C 1.364676 4.176310 -2.280501

C 0.112559 5.495189 -0.686230

H -1.237218 4.412612 0.589644

H 1.002919 2.065332 -2.263190

H 2.112093 4.090041 -3.063391

H -0.127651 6.456583 -0.239712

H 1.549229 6.314869 -2.071060

C -2.604951 1.423817 -1.164455

C -3.064242 2.507234 -1.924490

C -3.270867 0.173892 -1.226169

C -4.156376 2.371192 -2.781222

H -2.548596 3.460067 -1.866157

C -4.344160 0.055366 -2.125509

C -4.782362 1.131647 -2.894811

H -4.494459 3.219782 -3.369380

H -4.840555 -0.902392 -2.228312

H -5.611306 0.995155 -3.584001

C -2.857393 -1.010697 -0.409509

C -1.492889 -1.366363 -0.254298

C -3.848133 -1.848025 0.189764

C -1.150972 -2.559017 0.397283

C -3.465862 -3.032161 0.834549

C -2.127526 -3.397463 0.927658

H -0.107485 -2.840223 0.476928

H -4.233560 -3.649421 1.291757

H -1.841207 -4.320221 1.426064

P 2.152847 -0.598800 1.116190

C 2.883207 0.971155 1.770794

C 3.927501 3.422940 2.663416

C 3.184283 1.994112 0.853831

C 3.113567 1.192006 3.138713

C 3.629091 2.412809 3.579992

C 3.710455 3.207362 1.300973

H 3.042493 1.837268 -0.212913

H 2.889818 0.414325 3.861663

H 3.801162 2.570163 4.641767

H 3.941824 3.983490 0.577099

H 4.328078 4.372177 3.009447

C 1.723875 -1.541352 2.649650

C 0.991661 -2.885344 5.008712

C 2.657018 -2.332767 3.340997

C 0.418575 -1.437710 3.156253

C 0.057532 -2.100784 4.330848

C 2.291598 -3.000898 4.510415

H 3.671327 -2.427746 2.966338

H -0.321173 -0.844025 2.630194

H -0.957899 -2.010328 4.706604

H 3.024305 -3.610732 5.032447

H 0.708367 -3.407844 5.918599

C 3.632743 -1.526677 0.490618

C 3.446077 -2.845912 0.030244

C 4.919039 -0.966836 0.419303

C 4.520878 -3.583183 -0.471857

H 2.458391 -3.293139 0.055470

C 5.989484 -1.705843 -0.089691

H 5.087193 0.051243 0.750848

C 5.795787 -3.013989 -0.534414

H 4.358208 -4.601173 -0.815709

H 6.974082 -1.250398 -0.143523

H 6.629862 -3.585760 -0.931637

C 0.833352 -2.687463 -2.998624

C -0.242130 -2.053318 -3.894609

C 2.113345 -2.901934 -3.840206

C 0.339035 -4.049183 -2.474851

H -1.181864 -1.932587 -3.346962

H 0.084255 -1.068030 -4.244498

H -0.435586 -2.684427 -4.771142

H 2.906253 -3.360633 -3.232671

H 1.928727 -3.570582 -4.689016

H 2.480953 -1.950450 -4.247932

H 0.110794 -4.733728 -3.301537

H 1.106062 -4.519213 -1.847499

H -0.565183 -3.924301 -1.872638

Rh 0.125449 -0.359541 -0.885008

H -0.693933 -0.123682 -2.161828

O 1.194324 -1.856319 -1.891310

Li 2.873012 -1.134103 -2.051784

Br 3.634916 0.909211 -2.803586

C -5.309440 -1.527411 0.235187

C -6.251219 -2.458367 -0.233885

C -5.783527 -0.343172 0.821306

C -7.620741 -2.205331 -0.138343

H -5.901781 -3.381574 -0.689035

C -7.151014 -0.090986 0.922119

H -5.069758 0.372570 1.215943

C -8.076518 -1.019029 0.438682

H -8.330762 -2.936633 -0.516088

H -7.495630 0.830442 1.384747

H -9.142246 -0.820663 0.514956

**TS17B-2n**

P -0.887764 -1.660838 0.086031

C -1.567700 -2.278912 -1.508491

C -2.538851 -3.211018 -3.969215

C -2.798191 -2.950199 -1.576911

C -0.828394 -2.081398 -2.684555

C -1.311510 -2.547695 -3.907853

C -3.280074 -3.412902 -2.802991

H -3.379644 -3.108328 -0.673050

H 0.126222 -1.563600 -2.642799

H -0.731359 -2.386138 -4.812064

H -4.236033 -3.927780 -2.846560

H -2.918446 -3.566541 -4.923232

C 0.018540 -3.099369 0.796023

C 1.407936 -5.236894 1.973647

C -0.092262 -4.394666 0.268347

C 0.830076 -2.888482 1.922646

C 1.518927 -3.950606 2.508672

C 0.601120 -5.456112 0.855274

H -0.715033 -4.577948 -0.600986

H 0.929922 -1.889580 2.334888

H 2.151147 -3.767383 3.372524

H 0.508697 -6.454632 0.436310

H 1.948463 -6.063920 2.426132

C -2.323710 -1.458317 1.213912

C -2.570323 -2.392268 2.232161

C -3.125784 -0.295739 1.106876

C -3.573770 -2.177887 3.175702

H -1.968531 -3.291551 2.298664

C -4.103331 -0.085432 2.095504

C -4.326027 -1.006039 3.116491

H -3.748606 -2.911413 3.957790

H -4.694785 0.821709 2.063555

H -5.087317 -0.802896 3.864772

C -2.928931 0.706367 0.019821

C -1.622164 1.056077 -0.388110

C -4.038575 1.366644 -0.585351

C -1.436762 2.100469 -1.303058

C -3.807885 2.414608 -1.489608

C -2.517653 2.800796 -1.836416

H -0.435219 2.426685 -1.587691

H -4.662023 2.907940 -1.944403

H -2.345405 3.633958 -2.511398

P 2.678419 -0.300661 -0.239495

C 3.111205 -1.577082 -1.513876

C 3.598451 -3.514780 -3.503108

C 2.898146 -2.943298 -1.255962

C 3.568543 -1.200900 -2.787439

C 3.805989 -2.161842 -3.773003

C 3.146791 -3.901335 -2.239615

H 2.549141 -3.267767 -0.282271

H 3.749140 -0.155430 -3.013620

H 4.162286 -1.847710 -4.750504

H 2.983251 -4.951681 -2.013855

H 3.788760 -4.261829 -4.268950

C 3.638058 1.172988 -0.809838

C 5.040956 3.420520 -1.750330

C 4.988724 1.361910 -0.473773

C 3.004915 2.127437 -1.621316

C 3.698671 3.247733 -2.089110

C 5.682578 2.476982 -0.943757

H 5.497135 0.642006 0.159162

H 1.960254 2.001395 -1.902017

H 3.170824 3.994119 -2.671863

H 6.726252 2.612089 -0.671941

H 5.580183 4.295432 -2.102194

C 3.603018 -0.792453 1.281954

C 3.317188 -0.057875 2.446230

C 4.577593 -1.799081 1.330317

C 3.986911 -0.339254 3.637282

H 2.580526 0.741300 2.400809

C 5.240653 -2.080747 2.528252

H 4.823958 -2.366593 0.439033

C 4.945346 -1.355681 3.683684

H 3.760601 0.238270 4.529828

H 5.992781 -2.865083 2.553150

H 5.464086 -1.575556 4.613097

C 0.292031 2.755165 2.632231

C -0.064647 1.669083 3.662810

C 1.291712 3.747253 3.266000

C -0.985467 3.506704 2.213539

H -0.787549 0.959342 3.242379

H 0.828023 1.112222 3.971615

H -0.514846 2.109552 4.560593

H 1.548902 4.550758 2.563269

H 0.868344 4.220214 4.160700

H 2.216769 3.232585 3.549748

H -1.461576 3.981182 3.081234

H -0.761260 4.293374 1.482664

H -1.703364 2.819706 1.757154

Rh 0.224532 0.281356 -0.056132

H 0.342243 0.605653 1.444631

O 0.954670 2.177141 1.492889

Li 1.310625 3.496202 0.257559

Br 0.913474 5.309189 -1.080662

C -5.464983 0.969487 -0.380996

C -6.423233 1.926221 -0.006851

C -5.898652 -0.342993 -0.631291

C -7.768224 1.578821 0.131250

H -6.104492 2.946547 0.189953

C -7.242508 -0.689463 -0.498536

H -5.175306 -1.088989 -0.947588

C -8.183222 0.268649 -0.112378

H -8.490926 2.333814 0.429932

H -7.558055 -1.709515 -0.703263

H -9.230205 -0.003062 -0.007363

**INT17B-2n**

P -0.668915 -1.137515 -0.112223

C -1.554004 -1.625135 -1.662960

C -2.842965 -2.363910 -4.046801

C -2.683798 -2.458367 -1.633413

C -1.082174 -1.163960 -2.899828

C -1.720532 -1.534412 -4.084959

C -3.324450 -2.824297 -2.819028

H -3.067140 -2.817058 -0.682050

H -0.222199 -0.499982 -2.920533

H -1.346398 -1.166481 -5.036790

H -4.200998 -3.466117 -2.782731

H -3.345080 -2.645421 -4.968731

C 0.323145 -2.648169 0.295264

C 1.953130 -4.845122 0.961185

C 0.313105 -3.810078 -0.491464

C 1.166740 -2.603749 1.419254

C 1.968308 -3.693967 1.753860

C 1.125940 -4.898668 -0.160805

H -0.325848 -3.869888 -1.365963

H 1.200044 -1.705768 2.028216

H 2.613188 -3.637872 2.626632

H 1.107956 -5.789336 -0.783798

H 2.585037 -5.692033 1.215444

C -1.991796 -1.093091 1.167429

C -2.105436 -2.103884 2.132568

C -2.832305 0.049155 1.231724

C -3.002823 -1.991267 3.194828

H -1.474951 -2.983951 2.064784

C -3.696850 0.154019 2.335233

C -3.784110 -0.843222 3.304880

H -3.072250 -2.784592 3.934394

H -4.309324 1.041679 2.438859

H -4.463329 -0.717315 4.144049

C -2.781358 1.143442 0.212382

C -1.534270 1.528571 -0.342880

C -3.961009 1.848976 -0.168167

C -1.481622 2.663553 -1.171601

C -3.851429 2.990465 -0.976744

C -2.616709 3.419512 -1.456100

H -0.526332 2.967527 -1.604560

H -4.758210 3.513548 -1.267242

H -2.550227 4.306651 -2.082153

P 2.729031 0.649099 0.030863

C 3.850576 -0.263415 -1.124838

C 5.482881 -1.736530 -2.877203

C 3.448474 -1.527425 -1.583376

C 5.079987 0.255978 -1.562785

C 5.889313 -0.476049 -2.433712

C 4.261869 -2.260683 -2.448823

H 2.495758 -1.937403 -1.264234

H 5.403191 1.237640 -1.228833

H 6.836345 -0.059396 -2.767148

H 3.935821 -3.238951 -2.791959

H 6.112748 -2.304181 -3.557200

C 3.207251 2.418688 -0.258679

C 3.736669 5.131043 -0.792567

C 3.919027 3.201643 0.662216

C 2.762082 3.018114 -1.452160

C 3.028438 4.361064 -1.718627

C 4.179680 4.548300 0.395703

H 4.270462 2.761765 1.590124

H 2.207252 2.424648 -2.175922

H 2.679683 4.806419 -2.646670

H 4.731667 5.141412 1.120306

H 3.939772 6.179077 -0.995737

C 3.436754 0.291895 1.704160

C 2.629090 0.591945 2.814874

C 4.706949 -0.260996 1.921028

C 3.087712 0.359103 4.111930

H 1.631807 0.995429 2.649539

C 5.161204 -0.503129 3.220101

H 5.342397 -0.508767 1.076647

C 4.355725 -0.191416 4.316986

H 2.451735 0.598401 4.960150

H 6.146436 -0.936263 3.373152

H 4.711580 -0.380844 5.326363

Rh 0.348186 0.806852 -0.151525

C -5.350954 1.403973 0.154409

C -5.786928 0.105752 -0.161420

C -6.282496 2.300454 0.704072

C -7.104082 -0.283806 0.075924

H -5.085833 -0.593338 -0.608119

C -7.600891 1.909921 0.945974

H -5.961900 3.308233 0.955752

C -8.017194 0.614867 0.633958

H -7.421254 -1.291454 -0.181371

H -8.301549 2.618918 1.380122

H -9.043354 0.308816 0.820221

**INT18B-2n**

P -1.238045 -1.432076 0.442839

C -2.320223 -2.386220 -0.716703

C -3.898663 -3.864474 -2.509190

C -3.595952 -2.835467 -0.339557

C -1.848966 -2.681366 -2.003362

C -2.631808 -3.420096 -2.892675

C -4.380363 -3.569726 -1.231564

H -3.978539 -2.606363 0.651471

H -0.871887 -2.313312 -2.304278

H -2.255214 -3.639270 -3.888405

H -5.368147 -3.908279 -0.929401

H -4.511603 -4.431961 -3.204613

C -0.567989 -2.744971 1.568614

C 0.565696 -4.641841 3.311193

C -0.987899 -4.083920 1.537620

C 0.428496 -2.372548 2.486566

C 0.987005 -3.310217 3.354239

C -0.420877 -5.025029 2.401756

H -1.755546 -4.397752 0.838141

H 0.771679 -1.343023 2.509715

H 1.760157 -3.001389 4.052398

H -0.753967 -6.059055 2.361653

H 1.006827 -5.376299 3.979998

C -2.423717 -0.511483 1.514510

C -2.647570 -0.885663 2.847054

C -3.026428 0.663306 0.997272

C -3.425966 -0.097744 3.695861

H -2.199484 -1.794628 3.233475

C -3.777527 1.454936 1.882197

C -3.975387 1.087686 3.212138

H -3.584662 -0.403495 4.726663

H -4.207077 2.382234 1.521414

H -4.557906 1.731665 3.865921

C -2.836402 1.086576 -0.423197

C -1.567338 0.927198 -1.032205

C -3.899939 1.689227 -1.158949

C -1.370368 1.448902 -2.323044

C -3.645105 2.212372 -2.433937

C -2.380984 2.114294 -3.011066

H -0.394797 1.343863 -2.800087

H -4.464217 2.658891 -2.990708

H -2.198549 2.524479 -4.002050

P 2.426149 -0.846289 -0.419661

C 2.780126 -2.469600 -1.250178

C 3.242160 -4.972009 -2.456419

C 1.930715 -3.557399 -0.995031

C 3.865747 -2.656211 -2.121820

C 4.092098 -3.896881 -2.721868

C 2.162703 -4.799110 -1.588129

H 1.085221 -3.435345 -0.327936

H 4.536960 -1.831068 -2.337532

H 4.934673 -4.020782 -3.397447

H 1.493477 -5.628234 -1.374166

H 3.418653 -5.936643 -2.925119

C 3.389148 0.324791 -1.493341

C 4.710992 2.111576 -3.222198

C 4.646291 0.846166 -1.155919

C 2.802515 0.715842 -2.710415

C 3.457222 1.599175 -3.568978

C 5.301172 1.733483 -2.015148

H 5.118336 0.560471 -0.221524

H 1.829089 0.316544 -2.987638

H 2.989142 1.887352 -4.506587

H 6.275730 2.127133 -1.738019

H 5.223197 2.799413 -3.889888

C 3.461067 -0.933484 1.117793

C 3.238293 0.037852 2.109262

C 4.430518 -1.920865 1.346576

C 3.977694 0.028009 3.292601

H 2.479809 0.800127 1.952154

C 5.163044 -1.934978 2.536270

H 4.614061 -2.684293 0.597520

C 4.940577 -0.960896 3.510886

H 3.795482 0.789290 4.046678

H 5.907970 -2.709749 2.699280

H 5.511368 -0.973036 4.435805

Rh 0.166606 0.034953 -0.425971

Br 0.683542 2.736984 0.638478

C 2.006880 4.147646 0.661593

C 2.902076 4.273606 -0.398486

C 2.032090 5.021904 1.747517

C 3.844814 5.304041 -0.362950

H 2.867343 3.583314 -1.233905

C 2.979510 6.047975 1.766808

H 1.323401 4.905174 2.560836

C 3.886424 6.191405 0.714430

H 4.547540 5.407915 -1.185363

H 3.004526 6.735447 2.608206

H 4.621487 6.991172 0.734116

C -5.322853 1.731302 -0.702938

C -6.039801 2.939374 -0.709572

C -6.004676 0.558101 -0.338246

C -7.388294 2.978042 -0.350028

H -5.526999 3.856634 -0.987613

C -7.352216 0.594864 0.016891

H -5.471764 -0.388113 -0.350147

C -8.050497 1.805286 0.016034

H -7.920046 3.926386 -0.355169

H -7.861510 -0.326210 0.289830

H -9.100612 1.832517 0.295217

**TS19B-2n**

P 0.817052 1.170939 0.463432

C 1.427049 2.253122 -0.906608

C 2.281946 3.914558 -3.004924

C 2.595335 3.021703 -0.777263

C 0.695184 2.328264 -2.099537

C 1.117832 3.156425 -3.140858

C 3.019849 3.846409 -1.820898

H 3.174806 2.972101 0.140433

H -0.207199 1.734939 -2.210607

H 0.539095 3.204308 -4.059376

H 3.928204 4.432895 -1.709683

H 2.615455 4.553481 -3.818373

C -0.008625 2.362596 1.614836

C -1.283036 4.075260 3.448327

C 0.031207 3.753042 1.438699

C -0.696126 1.840332 2.724225

C -1.325621 2.690361 3.634016

C -0.601378 4.602671 2.350700

H 0.551579 4.179184 0.587941

H -0.733419 0.766785 2.878144

H -1.849695 2.269526 4.488162

H -0.559606 5.678352 2.199301

H -1.774777 4.737187 4.156442

C 2.341503 0.711573 1.389495

C 2.657673 1.296713 2.624333

C 3.150641 -0.326941 0.872312

C 3.742840 0.848667 3.377045

H 2.046923 2.104399 3.011492

C 4.216971 -0.784128 1.666729

C 4.513725 -0.210273 2.900717

H 3.970181 1.315469 4.331745

H 4.822474 -1.608100 1.307011

H 5.346253 -0.592602 3.485754

C 2.883600 -0.957050 -0.454729

C 1.555111 -1.272939 -0.845650

C 3.977826 -1.288166 -1.311807

C 1.379998 -2.006270 -2.037320

C 3.743605 -2.017249 -2.483639

C 2.451912 -2.394015 -2.836322

H 0.381143 -2.296840 -2.345192

H 4.581178 -2.252115 -3.134290

H 2.277202 -2.969799 -3.742734

P -2.739564 -0.029265 -0.504574

C -3.088681 1.730239 -0.998306

C -3.584187 4.425323 -1.669579

C -2.982664 2.746041 -0.032670

C -3.441528 2.093201 -2.308671

C -3.680968 3.428630 -2.640717

C -3.237481 4.078000 -0.362835

H -2.706337 2.498896 0.986469

H -3.535557 1.334743 -3.077287

H -3.951467 3.685218 -3.661843

H -3.151790 4.841973 0.404501

H -3.775202 5.463459 -1.928379

C -3.127180 -0.922718 -2.087889

C -3.605213 -2.191569 -4.557203

C -4.399428 -1.424457 -2.400264

C -2.102083 -1.058050 -3.038807

C -2.338197 -1.682768 -4.264133

C -4.633125 -2.059651 -3.622486

H -5.213253 -1.323574 -1.690337

H -1.110353 -0.675774 -2.812939

H -1.529718 -1.776834 -4.984399

H -5.623775 -2.448932 -3.842926

H -3.789823 -2.686649 -5.506914

C -4.233350 -0.448989 0.531679

C -4.397838 -1.786960 0.938522

C -5.185070 0.492984 0.950064

C -5.476668 -2.165712 1.737525

H -3.680738 -2.538372 0.623104

C -6.260612 0.112634 1.757758

H -5.099725 1.528983 0.642642

C -6.411418 -1.215445 2.155176

H -5.583424 -3.205829 2.034297

H -6.985133 0.861175 2.068443

H -7.249741 -1.509298 2.781189

Rh -0.277874 -0.729028 -0.067018

Br -0.997927 -3.290639 0.135681

C -0.297217 -2.229316 1.813753

C 0.992372 -2.575300 2.246648

C -1.284903 -1.856178 2.738262

C 1.319850 -2.412343 3.591111

H 1.727610 -2.931369 1.536056

C -0.933357 -1.704503 4.082365

H -2.302970 -1.679473 2.415443

C 0.367020 -1.971813 4.515963

H 2.333358 -2.633656 3.913866

H -1.695383 -1.391693 4.792754

H 0.630244 -1.862469 5.564123

C 5.386503 -0.834858 -1.093529

C 6.444097 -1.759452 -1.121133

C 5.700969 0.525966 -0.942928

C 7.769284 -1.341639 -0.985772

H 6.219232 -2.816701 -1.236865

C 7.024191 0.944998 -0.812474

H 4.898122 1.256886 -0.944345

C 8.065333 0.013283 -0.829382

H 8.569960 -2.076996 -1.001772

H 7.244307 2.004178 -0.703201

H 9.096395 0.341016 -0.725271

**INT19B-2n**

P 0.882893 1.233455 0.251444

C 1.440932 1.904130 -1.373586

C 2.226158 2.956326 -3.855282

C 2.637792 2.626851 -1.493320

C 0.638939 1.723964 -2.509137

C 1.028667 2.247891 -3.743017

C 3.028196 3.147647 -2.727904

H 3.262485 2.782498 -0.618559

H -0.298203 1.180817 -2.430373

H 0.397748 2.098889 -4.614959

H 3.959614 3.701498 -2.808999

H 2.533354 3.358565 -4.816784

C 0.072883 2.697105 1.045162

C -1.065410 4.928274 2.332524

C -0.045957 3.925423 0.377844

C -0.402315 2.599505 2.365827

C -0.963748 3.706370 3.002573

C -0.609019 5.031271 1.018793

H 0.303497 4.028869 -0.642770

H -0.311134 1.665096 2.908072

H -1.313242 3.613443 4.027458

H -0.687052 5.974538 0.484977

H -1.494991 5.792392 2.832606

C 2.413193 0.981405 1.238517

C 2.741315 1.814638 2.317435

C 3.205650 -0.157724 0.960632

C 3.811894 1.512614 3.158514

H 2.151062 2.701556 2.514843

C 4.253585 -0.460130 1.844812

C 4.553275 0.354797 2.933504

H 4.047850 2.169774 3.990923

H 4.842714 -1.353355 1.672226

H 5.368295 0.084847 3.599554

C 2.969913 -1.011568 -0.241841

C 1.666865 -1.429989 -0.613423

C 4.084225 -1.401060 -1.045250

C 1.513869 -2.239970 -1.754060

C 3.884281 -2.222815 -2.161762

C 2.608019 -2.637595 -2.518710

H 0.531584 -2.603840 -2.028209

H 4.742158 -2.503084 -2.766209

H 2.459241 -3.275934 -3.386363

P -2.865480 -0.170220 -0.369039

C -3.337474 1.577290 -0.791533

C -4.009706 4.256542 -1.357757

C -3.452233 2.525627 0.240090

C -3.549749 2.002004 -2.112849

C -3.876821 3.331430 -2.392622

C -3.797543 3.847199 -0.039068

H -3.286129 2.230397 1.271929

H -3.478423 1.291177 -2.929198

H -4.042072 3.636411 -3.422861

H -3.892423 4.558344 0.776308

H -4.276883 5.287404 -1.574920

C -3.310789 -1.055136 -1.934304

C -3.907802 -2.314422 -4.378096

C -4.612727 -1.495296 -2.210723

C -2.312466 -1.264729 -2.897470

C -2.607341 -1.880668 -4.113872

C -4.906937 -2.123653 -3.422416

H -5.399511 -1.357642 -1.476179

H -1.288429 -0.963114 -2.693393

H -1.818478 -2.036456 -4.844914

H -5.919686 -2.467121 -3.616476

H -4.138550 -2.806049 -5.319398

C -4.213252 -0.658635 0.809787

C -4.001469 -1.763396 1.651741

C -5.443317 0.019208 0.879066

C -4.995964 -2.174194 2.541443

H -3.068013 -2.313532 1.593717

C -6.433083 -0.394902 1.772120

H -5.632294 0.874035 0.238406

C -6.211197 -1.491327 2.607603

H -4.813794 -3.031425 3.183929

H -7.377987 0.141116 1.812126

H -6.981994 -1.811404 3.304033

Rh -0.172613 -0.852354 0.075550

Br -1.114098 -3.249404 -0.019083

C 0.059136 -1.220766 2.048611

C 1.070171 -2.037440 2.565788

C -0.920337 -0.722227 2.913305

C 1.117723 -2.310733 3.936209

H 1.807049 -2.480565 1.908680

C -0.868713 -0.999272 4.285893

H -1.744672 -0.131678 2.525812

C 0.156197 -1.789533 4.803954

H 1.910313 -2.948660 4.320648

H -1.642433 -0.601911 4.939265

H 0.197409 -2.009381 5.867600

C 5.492749 -0.940794 -0.827368

C 6.520521 -1.883941 -0.662161

C 5.841273 0.418309 -0.874263

C 7.850632 -1.481783 -0.527868

H 6.267993 -2.940740 -0.628533

C 7.169848 0.821396 -0.745587

H 5.062613 1.158494 -1.028407

C 8.180711 -0.126282 -0.567417

H 8.628167 -2.229429 -0.392936

H 7.417943 1.879029 -0.790112

H 9.215634 0.189279 -0.464957

**TS20B-2n**

P 0.653184 0.974637 0.795015

C 1.124396 2.197796 -0.511741

C 1.897341 3.990755 -2.536231

C 2.007450 3.257248 -0.233031

C 0.659840 2.038328 -1.822517

C 1.043486 2.927860 -2.828433

C 2.380798 4.153057 -1.235239

H 2.424930 3.370443 0.763192

H 0.011111 1.202778 -2.058393

H 0.677032 2.783793 -3.841040

H 3.060351 4.968756 -1.002728

H 2.196842 4.682676 -3.318785

C -0.261130 1.916177 2.097782

C -1.651368 3.180645 4.192241

C -0.263494 3.314174 2.210636

C -0.975383 1.161521 3.044326

C -1.663546 1.788042 4.084027

C -0.950963 3.939954 3.253310

H 0.252842 3.922996 1.477374

H -1.002114 0.079451 2.956170

H -2.214376 1.187256 4.802295

H -0.943741 5.024500 3.324697

H -2.188804 3.670620 4.999746

C 2.283399 0.655936 1.585087

C 2.642544 1.214048 2.820778

C 3.153116 -0.246203 0.935578

C 3.850342 0.876778 3.430525

H 1.970815 1.905656 3.318280

C 4.352139 -0.590845 1.581065

C 4.699579 -0.040993 2.812215

H 4.115918 1.317645 4.387587

H 5.013897 -1.308787 1.109685

H 5.631254 -0.333945 3.288449

C 2.814898 -0.858172 -0.380996

C 1.557346 -1.488518 -0.595942

C 3.788313 -0.876695 -1.420755

C 1.340646 -2.183913 -1.805892

C 3.516559 -1.545859 -2.620380

C 2.303259 -2.202342 -2.807368

H 0.405900 -2.713567 -1.948753

H 4.261099 -1.531528 -3.410728

H 2.101138 -2.726614 -3.738404

P -2.546678 0.070045 -0.524283

C -2.770331 1.905097 -0.791275

C -3.114961 4.679229 -1.175966

C -2.976738 2.764649 0.300815

C -2.737910 2.465212 -2.079170

C -2.906624 3.837902 -2.268984

C -3.149894 4.136003 0.109167

H -3.027327 2.363703 1.306864

H -2.599231 1.827843 -2.945466

H -2.884446 4.245168 -3.276433

H -3.315427 4.777265 0.970637

H -3.254334 5.746864 -1.324118

C -3.107974 -0.569913 -2.169831

C -3.860400 -1.390543 -4.750796

C -4.459439 -0.734531 -2.497835

C -2.136715 -0.833473 -3.147749

C -2.510347 -1.230771 -4.431406

C -4.831335 -1.148236 -3.778775

H -5.228235 -0.562293 -1.752856

H -1.083850 -0.761710 -2.891902

H -1.744494 -1.433569 -5.175482

H -5.883927 -1.284821 -4.012707

H -4.152291 -1.711716 -5.747162

C -3.915196 -0.262863 0.682802

C -3.733249 -1.205702 1.705772

C -5.137658 0.429925 0.615466

C -4.749357 -1.454052 2.630892

H -2.807319 -1.766298 1.759496

C -6.154460 0.173198 1.535855

H -5.295571 1.185243 -0.147570

C -5.962329 -0.769919 2.547401

H -4.589685 -2.191506 3.412944

H -7.093900 0.714927 1.463113

H -6.753200 -0.967860 3.266297

Rh -0.369435 -0.992723 0.133770

Br -1.815267 -3.102655 -0.458202

C 0.927998 -2.451959 1.090999

C 1.056979 -3.811968 0.746589

C 1.122515 -2.111980 2.442215

C 1.347341 -4.779318 1.703911

H 0.930301 -4.126126 -0.280241

C 1.389921 -3.090767 3.408541

H 1.053580 -1.089061 2.780503

C 1.509834 -4.428342 3.047117

H 1.440336 -5.817281 1.393723

H 1.509210 -2.786826 4.446041

H 1.734602 -5.185988 3.793420

C 5.107799 -0.176720 -1.335715

C 6.293083 -0.885008 -1.595508

C 5.199781 1.200265 -1.077187

C 7.532094 -0.242304 -1.583252

H 6.239082 -1.952156 -1.795001

C 6.436730 1.843840 -1.069853

H 4.293950 1.769657 -0.895943

C 7.608964 1.126081 -1.319454

H 8.436590 -0.812754 -1.778715

H 6.484452 2.912191 -0.873927

H 8.572239 1.629341 -1.310676

**INT20B-2n**

P 2.182446 -0.081362 0.595035

C 2.930663 1.019438 -0.661285

C 3.864907 2.832883 -2.567773

C 4.288226 1.372422 -0.698091

C 2.036191 1.532922 -1.613090

C 2.523104 2.461014 -2.552506

C 4.758588 2.275393 -1.647963

H 4.974204 0.939520 0.024272

H 1.842637 2.870634 -3.292930

H 5.810161 2.548279 -1.668248

H 4.217681 3.545441 -3.308768

C 2.211668 0.932955 2.151471

C 2.244579 2.398021 4.556836

C 2.728703 2.238023 2.188913

C 1.704415 0.373406 3.338217

C 1.727289 1.100157 4.528602

C 2.742372 2.964506 3.383301

H 3.123918 2.696074 1.289135

H 1.279031 -0.625198 3.321842

H 1.334417 0.648286 5.435597

H 3.150318 3.972166 3.391590

H 2.259723 2.961760 5.486104

C 3.464280 -1.363591 0.933411

C 3.432537 -2.561876 0.206722

C 4.494545 -1.156491 1.864489

C 4.422472 -3.526324 0.394621

H 2.627620 -2.743103 -0.495121

C 5.482298 -2.125400 2.051733

H 4.521951 -0.245069 2.453323

C 5.449103 -3.311222 1.316079

H 4.383370 -4.450538 -0.175515

H 6.273038 -1.954116 2.777622

H 6.215761 -4.067047 1.466252

Br -0.019549 -2.329979 1.680581

C 0.345180 -0.241009 -2.358676

C -0.399145 2.102612 -1.910321

C -0.868965 -0.396804 -3.116734

C -1.513756 1.889362 -2.698807

C -1.734315 0.646591 -3.325362

H -1.036042 -1.345771 -3.616024

H -2.254298 2.675911 -2.797928

H -2.606526 0.512324 -3.957127

C 0.606918 1.059267 -1.759776

Rh 0.002138 -0.473632 -0.083235

P -2.470887 -0.561956 0.180890

C -3.652049 0.675596 -0.565871

C -3.754133 1.967347 -0.023829

C -4.412105 0.377528 -1.708978

C -4.594643 2.922625 -0.596156

H -3.183804 2.234124 0.859144

C -5.259604 1.330746 -2.278274

H -4.353771 -0.608528 -2.156537

C -5.354274 2.608237 -1.725011

H -4.658178 3.912794 -0.152757

H -5.846297 1.070171 -3.155791

H -6.015608 3.349702 -2.165625

C -3.331695 -2.162760 -0.188282

C -2.562816 -3.275635 -0.557420

C -4.729843 -2.307196 -0.122389

C -3.170156 -4.498055 -0.852752

H -1.483000 -3.187536 -0.573859

C -5.336251 -3.529402 -0.414924

H -5.352057 -1.458327 0.143140

C -4.557134 -4.628702 -0.782678

H -2.554512 -5.349050 -1.131834

H -6.417871 -3.620337 -0.357299

H -5.029987 -5.580113 -1.012110

C -2.774442 -0.246468 1.987339

C -3.755353 -0.906640 2.738673

C -1.984809 0.726119 2.620794

C -3.952228 -0.588848 4.084352

H -4.358470 -1.686514 2.288027

C -2.190249 1.052775 3.961305

H -1.186619 1.210856 2.065963

C -3.177348 0.395568 4.698070

H -4.711710 -1.118982 4.653399

H -1.564018 1.806580 4.430769

H -3.333478 0.640671 5.745461

C 1.375267 -1.286991 -2.714440

C 2.601418 -0.982500 -3.327668

C 1.030865 -2.646633 -2.583120

C 3.461586 -1.997016 -3.756519

H 2.886931 0.048930 -3.490477

C 1.883336 -3.657922 -3.020062

H 0.085410 -2.905846 -2.118850

C 3.111736 -3.337715 -3.604872

H 4.405806 -1.729249 -4.223716

H 1.588893 -4.697142 -2.897857

H 3.781297 -4.123342 -3.944617

C -0.237364 3.434747 -1.263622

C 0.147563 3.555759 0.081859

C -0.510156 4.613242 -1.980948

C 0.255134 4.806934 0.688282

H 0.346204 2.658357 0.657811

C -0.402662 5.865153 -1.375729

H -0.791045 4.545303 -3.028550

C -0.019541 5.968353 -0.036956

H 0.551031 4.868454 1.732259

H -0.611985 6.761405 -1.954262

H 0.065033 6.943532 0.435336

**INT21B-2n**

P -3.087977 0.405808 -0.151764

C -3.585461 1.338390 1.375450

C -4.275914 2.631785 3.779491

C -3.432267 0.684079 2.611398

C -4.079182 2.651468 1.364880

C -4.421523 3.292207 2.558767

C -3.782011 1.325367 3.800815

H -3.054078 -0.334039 2.634129

H -4.188487 3.186357 0.428295

H -4.802509 4.310160 2.530395

H -3.665038 0.800104 4.745155

H -4.544175 3.131408 4.706882

C -3.300159 1.627618 -1.539330

C -3.637872 3.386085 -3.714088

C -4.571089 2.139993 -1.861746

C -2.212818 1.996967 -2.337997

C -2.377281 2.871577 -3.415575

C -4.736681 3.016221 -2.933374

H -5.438981 1.837960 -1.283185

H -1.232518 1.596856 -2.117915

H -1.516468 3.146555 -4.019491

H -5.726355 3.400699 -3.166175

H -3.768367 4.063681 -4.553920

C -4.573766 -0.649986 -0.510982

C -4.567611 -1.399678 -1.698645

C -5.691139 -0.724313 0.329240

C -5.652810 -2.206746 -2.035107

H -3.708801 -1.355412 -2.362890

C -6.778863 -1.533012 -0.010270

H -5.715542 -0.158458 1.254242

C -6.763201 -2.276678 -1.189769

H -5.629735 -2.783238 -2.956275

H -7.637336 -1.582308 0.654655

H -7.608975 -2.907684 -1.449977

P 0.592200 0.324512 0.045170

C 1.376352 -0.032953 1.697483

C 1.532130 0.932832 2.701723

C 1.700343 -1.369593 2.005539

C 1.946403 0.570076 3.987473

H 1.326277 1.975610 2.493006

C 2.112023 -1.727462 3.290293

H 1.637640 -2.115405 1.216574

C 2.222581 -0.760312 4.294239

H 2.049702 1.337889 4.750044

H 2.361081 -2.764404 3.507774

H 2.534396 -1.041285 5.296438

C 1.895256 0.144445 -1.298416

C 1.270678 0.014629 -2.555377

C 3.313975 0.246097 -1.261399

C 1.973124 0.050038 -3.756276

H 0.193508 -0.128232 -2.573342

C 3.998917 0.280534 -2.492043

C 3.356664 0.200304 -3.724050

H 1.443196 -0.047500 -4.699806

H 5.081365 0.374561 -2.467584

H 3.935930 0.237589 -4.642798

C 0.327368 2.162244 0.054922

C 0.837536 3.006283 -0.943664

C -0.526471 2.719877 1.022908

C 0.496827 4.359793 -0.977536

H 1.500514 2.615419 -1.705490

C -0.842781 4.078535 1.004143

H -0.961805 2.090650 1.791368

C -0.338987 4.903171 -0.002901

H 0.902774 4.989790 -1.763329

H -1.504517 4.480413 1.765849

H -0.599330 5.958108 -0.028976

O 0.092419 -2.704403 -0.125886

C -0.043016 -3.712499 -1.131882

C -1.371541 -3.587380 -1.900693

C 1.130734 -3.601868 -2.118604

C 0.008353 -5.084150 -0.425296

H -2.229707 -3.710023 -1.233229

H -1.436991 -2.598825 -2.374058

H -1.434882 -4.346118 -2.691953

H 2.084143 -3.642392 -1.585634

H 1.109515 -4.417774 -2.853037

H 1.086662 -2.653525 -2.661127

H -0.042548 -5.912232 -1.143383

H 0.947465 -5.184881 0.134284

H -0.836817 -5.192220 0.266877

C 4.201783 0.278665 -0.035943

C 4.554037 1.503643 0.592265

C 4.776422 -0.929358 0.453754

C 5.374576 1.486799 1.733853

C 5.592597 -0.890547 1.597304

C 5.878283 0.301216 2.248715

H 5.644437 2.432870 2.192759

H 6.028915 -1.818654 1.953484

H 6.519609 0.309676 3.125856

Rh -1.255363 -1.036205 -0.070617

Br -2.797896 -2.809254 1.587047

Li -0.478079 -2.923149 1.565973

C 4.178796 2.873555 0.117966

C 3.669645 3.806477 1.039290

C 4.484718 3.329564 -1.175045

C 3.477120 5.141148 0.687095

H 3.425428 3.474832 2.044321

C 4.291524 4.665472 -1.530276

H 4.908439 2.645336 -1.900153

C 3.792391 5.578796 -0.600427

H 3.078657 5.838348 1.419199

H 4.552239 4.995270 -2.532858

H 3.652598 6.620939 -0.875514

C 4.630091 -2.295314 -0.152706

C 5.041886 -2.593143 -1.461883

C 4.244556 -3.371611 0.667613

C 5.051401 -3.906124 -1.935157

H 5.389623 -1.799601 -2.110405

C 4.251386 -4.684868 0.197260

H 3.946689 -3.170321 1.691340

C 4.655037 -4.959045 -1.110189

H 5.382506 -4.104772 -2.951108

H 3.947251 -5.493998 0.856432

H 4.664180 -5.980664 -1.479970

**TS22B-2n**

P 2.926378 0.556900 0.316909

C 3.720757 1.889142 -0.698324

C 4.963304 3.830152 -2.312125

C 3.586282 1.850904 -2.093704

C 4.486235 2.918545 -0.121844

C 5.101428 3.881375 -0.923277

C 4.206944 2.811980 -2.894129

H 3.004974 1.055584 -2.547130

H 4.599333 2.975223 0.956294

H 5.688608 4.670659 -0.460924

H 4.097648 2.761413 -3.974318

H 5.443381 4.580107 -2.935523

C 2.542138 1.436322 1.905920

C 1.844224 2.722190 4.314828

C 2.721473 0.793136 3.142865

C 2.004825 2.733864 1.898132

C 1.660875 3.369786 3.092479

C 2.376224 1.431006 4.335279

H 3.142618 -0.205922 3.179638

H 1.862463 3.257648 0.960048

H 1.248661 4.374897 3.061888

H 2.533651 0.919296 5.281323

H 1.579293 3.220227 5.243776

C 4.341220 -0.535767 0.824718

C 4.038108 -1.826285 1.290407

C 5.681223 -0.128134 0.784068

C 5.052460 -2.679792 1.727112

H 3.005280 -2.167815 1.283975

C 6.695559 -0.989299 1.209993

H 5.944296 0.854891 0.408954

C 6.385313 -2.263132 1.687115

H 4.798044 -3.672484 2.089578

H 7.730811 -0.661204 1.164118

H 7.176960 -2.929992 2.018686

P -0.577313 0.294626 -0.278795

C -1.388287 -0.433730 -1.762851

C -2.091057 0.321338 -2.716009

C -1.136504 -1.796707 -2.014976

C -2.535936 -0.271940 -3.895996

H -2.290451 1.371810 -2.535618

C -1.569478 -2.377307 -3.211237

H -0.597903 -2.413601 -1.291908

C -2.268801 -1.620539 -4.151283

H -3.089330 0.320574 -4.619798

H -1.354104 -3.425580 -3.401433

H -2.603468 -2.075141 -5.080191

C -1.695375 0.164240 1.215484

C -0.939690 0.107951 2.402167

C -3.115465 0.183235 1.311943

C -1.526262 0.107369 3.664619

H 0.141078 0.061158 2.325382

C -3.678723 0.173728 2.601593

C -2.913465 0.145787 3.764849

H -0.900117 0.073037 4.551369

H -4.762335 0.189573 2.684313

H -3.402641 0.143075 4.735372

C -0.491140 2.111486 -0.595243

C -0.906545 3.068042 0.339690

C 0.153561 2.546674 -1.768071

C -0.677033 4.428264 0.111421

H -1.404920 2.760317 1.251180

C 0.363952 3.903500 -2.002234

H 0.508075 1.814452 -2.486769

C -0.044467 4.849940 -1.056836

H -1.008555 5.154095 0.847739

H 0.867715 4.220035 -2.911359

H 0.133494 5.907856 -1.232113

C -4.105803 0.170644 0.167097

C -4.610280 -1.071818 -0.312073

C -4.614408 1.375768 -0.394808

C -5.507344 -1.083238 -1.393663

C -5.521483 1.306267 -1.468474

C -5.949787 0.091951 -1.984015

H -5.884530 -2.039285 -1.742568

H -5.920456 2.231716 -1.871518

H -6.656730 0.062380 -2.808761

Rh 1.396496 -0.798797 -0.612490

C 0.483370 -4.324193 0.864559

C -0.549926 -3.582640 1.733260

C -0.189202 -5.532981 0.174396

C 1.613122 -4.850838 1.781814

H -0.076188 -2.726991 2.226624

H -1.370956 -3.204429 1.116650

H -0.980011 -4.232501 2.506107

H 0.553500 -6.093210 -0.410488

H -0.649499 -6.225843 0.891491

H -0.967116 -5.184030 -0.512153

H 1.242106 -5.561024 2.532763

H 2.380122 -5.365291 1.185338

H 2.093259 -4.020545 2.313013

O 1.009111 -3.471507 -0.116333

Br 3.166834 -1.726252 -2.399072

Li 2.329570 -3.539169 -1.204634

C -4.328375 2.767984 0.080282

C -4.062792 3.774154 -0.867584

C -4.478317 3.161644 1.420923

C -3.960065 5.113599 -0.497768

H -3.932326 3.498320 -1.910130

C -4.366177 4.501651 1.795606

H -4.714018 2.422956 2.175946

C -4.113210 5.485826 0.838889

H -3.753428 5.865097 -1.255051

H -4.499551 4.776795 2.838816

H -4.040384 6.530205 1.131049

C -4.321865 -2.427320 0.262322

C -3.929250 -3.467184 -0.599905

C -4.617554 -2.759839 1.594561

C -3.831842 -4.782832 -0.148076

H -3.697823 -3.235602 -1.634317

C -4.513906 -4.074931 2.049509

H -4.966168 -1.994689 2.276302

C -4.122848 -5.093908 1.180867

H -3.527033 -5.565762 -0.837028

H -4.756407 -4.302835 3.084279

H -4.047629 -6.118788 1.533900

**INT22B-2n**

P -2.751205 0.857762 0.175981

C -2.562832 1.296308 1.965709

C -2.276493 1.861355 4.708148

C -2.193078 0.285674 2.870588

C -2.767742 2.597627 2.453804

C -2.624384 2.875840 3.814753

C -2.062091 0.567316 4.231319

H -2.006923 -0.728071 2.525847

H -3.030469 3.402055 1.776671

H -2.786774 3.888966 4.173929

H -1.780252 -0.230748 4.911859

H -2.167979 2.079343 5.767345

C -3.164002 2.458760 -0.658268

C -3.755425 4.880214 -1.959126

C -4.354241 3.152756 -0.373396

C -2.284662 2.992272 -1.608804

C -2.575958 4.196537 -2.254019

C -4.645131 4.354966 -1.017714

H -5.060205 2.747673 0.345516

H -1.372937 2.452075 -1.840427

H -1.880048 4.596095 -2.986577

H -5.569656 4.878367 -0.788733

H -3.985613 5.815430 -2.462693

C -4.382225 -0.034256 0.111304

C -5.201351 0.096110 -1.023589

C -4.778123 -0.921713 1.124377

C -6.389049 -0.625277 -1.133077

H -4.913064 0.766634 -1.826107

C -5.962695 -1.656154 1.004657

H -4.173930 -1.058134 2.013275

C -6.773420 -1.508048 -0.119950

H -7.012003 -0.501507 -2.015175

H -6.242241 -2.341882 1.799272

H -7.696052 -2.075140 -0.208688

P 0.778949 0.708069 0.007616

C 1.341884 0.142064 1.663769

C 2.233563 0.869639 2.467674

C 0.794886 -1.050264 2.162243

C 2.579967 0.401674 3.734688

H 2.650289 1.803437 2.107142

C 1.152732 -1.524085 3.425044

H 0.064482 -1.608154 1.582400

C 2.045282 -0.797814 4.213570

H 3.271444 0.973548 4.348056

H 0.710212 -2.449739 3.781547

H 2.319706 -1.158279 5.201760

C 1.901579 0.078395 -1.353380

C 1.265467 0.200733 -2.612287

C 3.153306 -0.588195 -1.301109

C 1.812217 -0.318158 -3.784237

H 0.308343 0.718684 -2.667022

C 3.661180 -1.142335 -2.491390

C 3.015738 -1.014838 -3.718134

H 1.287870 -0.195454 -4.727617

H 4.605028 -1.677285 -2.439530

H 3.453482 -1.453967 -4.610290

C 1.002096 2.542599 0.034011

C 1.577971 3.242240 -1.036051

C 0.459012 3.273647 1.105348

C 1.602927 4.638762 -1.038579

H 2.028212 2.703266 -1.862128

C 0.491376 4.667720 1.101754

H 0.010810 2.755990 1.946591

C 1.058237 5.355905 0.026763

H 2.065168 5.162167 -1.870903

H 0.067973 5.214646 1.940091

H 1.079658 6.442505 0.023280

O -2.412505 -1.756488 -1.594463

C -2.480250 -2.150253 -2.957918

C -3.673129 -3.115673 -3.096848

C -2.704838 -0.922056 -3.858997

C -1.183230 -2.875689 -3.363965

H -3.521942 -4.002212 -2.467554

H -4.598089 -2.623113 -2.778855

H -3.796815 -3.457403 -4.132042

H -1.893959 -0.196669 -3.722878

H -2.743686 -1.203347 -4.918782

H -3.646144 -0.426903 -3.599471

H -1.222268 -3.225958 -4.403449

H -0.316982 -2.212050 -3.263305

H -1.017513 -3.744458 -2.716672

C 4.035793 -0.761920 -0.094532

C 4.847252 0.302378 0.380055

C 4.181253 -2.053554 0.474798

C 5.710774 0.070498 1.464344

C 5.053182 -2.234904 1.559750

C 5.802657 -1.179024 2.064588

H 6.344605 0.881830 1.809206

H 5.132168 -3.222671 2.003609

H 6.475876 -1.335630 2.903132

Rh -1.118720 -0.318654 -0.807826

Br -2.093677 -3.618680 1.942483

Li -2.668111 -2.583012 -0.025095

C 3.508481 -3.280734 -0.056543

C 4.307964 -4.375663 -0.430260

C 2.115694 -3.419461 -0.140565

C 3.733269 -5.565409 -0.878281

H 5.390070 -4.285914 -0.375341

C 1.537801 -4.614457 -0.570439

H 1.474089 -2.603470 0.165571

C 2.344522 -5.690389 -0.945921

H 4.372252 -6.395743 -1.168252

H 0.455399 -4.704799 -0.584966

H 1.893690 -6.620844 -1.280416

C 4.920471 1.664564 -0.235907

C 4.925991 2.803727 0.589992

C 5.121465 1.856480 -1.614126

C 5.116549 4.080960 0.064333

H 4.780674 2.683864 1.659709

C 5.315222 3.133769 -2.141647

H 5.154396 0.999555 -2.276603

C 5.314074 4.252524 -1.306472

H 5.109005 4.941569 0.727803

H 5.483680 3.250624 -3.209265

H 5.471330 5.245762 -1.718661

**TS23B-2n**

P 2.522310 2.099215 0.067604

C 2.783425 1.925909 1.906782

C 3.236410 1.612653 4.672113

C 2.027235 2.647236 2.844920

C 3.756953 1.028632 2.380554

C 3.984289 0.879122 3.748945

C 2.252978 2.491793 4.215045

H 1.280848 3.359938 2.510640

H 4.351180 0.450479 1.681724

H 4.745640 0.182237 4.087824

H 1.666927 3.071812 4.923466

H 3.418943 1.500949 5.737611

C 4.271564 2.001916 -0.525572

C 6.912254 1.828914 -1.472965

C 5.299310 2.756501 0.072918

C 4.594870 1.135818 -1.577400

C 5.907177 1.057043 -2.053146

C 6.607461 2.673726 -0.400700

H 5.078722 3.402561 0.917847

H 3.835541 0.507562 -2.031575

H 6.128853 0.384205 -2.876838

H 7.389219 3.264929 0.069261

H 7.932255 1.768047 -1.843736

C 2.048701 3.888962 -0.100299

C 0.716655 4.233146 0.192657

C 2.923646 4.903487 -0.515217

C 0.285434 5.557942 0.118765

H 0.007872 3.449159 0.447289

C 2.485308 6.227161 -0.608173

H 3.947769 4.667828 -0.780953

C 1.171020 6.561276 -0.281426

H -0.747934 5.798291 0.354156

H 3.177550 6.996995 -0.939147

H 0.835113 7.592540 -0.350588

Rh 0.700726 0.729371 -0.530852

P 2.012027 -2.229505 1.074450

C 1.648615 -2.634806 2.849186

C 1.615598 -3.940276 3.368839

C 1.434388 -1.561873 3.728232

C 1.362830 -4.162854 4.723519

H 1.782748 -4.787498 2.711262

C 1.193577 -1.782144 5.086674

H 1.461985 -0.544018 3.350813

C 1.150969 -3.084692 5.586914

H 1.337875 -5.180225 5.105867

H 1.037655 -0.934391 5.748668

H 0.957024 -3.259957 6.642024

C 1.427898 -3.752569 0.178282

C 2.137299 -4.304793 -0.900522

C 0.176499 -4.306811 0.503963

C 1.620851 -5.388027 -1.615444

H 3.090387 -3.892312 -1.205720

C -0.332553 -5.394311 -0.206834

H -0.402825 -3.899450 1.324474

C 0.387812 -5.941726 -1.270199

H 2.190835 -5.791567 -2.447733

H -1.297940 -5.808062 0.073111

H -0.009716 -6.787276 -1.825701

C 3.862527 -2.401116 1.026998

C 4.531982 -2.101332 -0.172665

C 4.626981 -2.771437 2.146561

C 5.922507 -2.202995 -0.254330

H 3.975931 -1.806870 -1.059709

C 6.018569 -2.853213 2.064522

H 4.140032 -3.003151 3.087156

C 6.671505 -2.574390 0.862752

H 6.415027 -1.979483 -1.196100

H 6.590597 -3.143915 2.942446

H 7.754389 -2.643485 0.798800

O 0.381600 1.169476 -2.572120

C 0.759485 2.133764 -3.574992

C 2.272638 2.376957 -3.643130

C 0.303670 1.506261 -4.913566

C 0.037481 3.481989 -3.399520

H 2.639735 2.870563 -2.740237

H 2.812860 1.436393 -3.780924

H 2.511497 3.029907 -4.491889

H -0.763250 1.263680 -4.877547

H 0.475459 2.201938 -5.744293

H 0.865582 0.589670 -5.134251

H 0.397362 4.200460 -4.145762

H -1.042833 3.375094 -3.542525

H 0.224697 3.906912 -2.410109

Br 2.879948 -1.700118 -3.681245

Li 1.008845 -0.538942 -2.954169

P -1.595588 -0.133619 -0.622009

C -2.097943 -1.395707 0.625644

C -2.941689 -2.471984 0.310771

C -1.597324 -1.294713 1.932132

C -3.300303 -3.401638 1.286543

H -3.319571 -2.581849 -0.699381

C -1.964697 -2.217791 2.912774

H -0.907166 -0.497252 2.188870

C -2.821159 -3.271819 2.592070

H -3.959246 -4.226213 1.026987

H -1.561369 -2.123394 3.916011

H -3.103721 -3.995231 3.352264

C -2.825480 1.284099 -0.530089

C -2.320614 2.395395 -1.233310

C -4.052573 1.439191 0.168500

C -2.969139 3.625859 -1.264842

H -1.382495 2.270394 -1.758627

C -4.663468 2.710138 0.163275

C -4.147362 3.792183 -0.540202

H -2.539073 4.446730 -1.831810

H -5.582407 2.831745 0.730193

H -4.661450 4.749609 -0.519420

C -1.900682 -0.970198 -2.238964

C -2.809816 -0.506805 -3.195907

C -1.068145 -2.060791 -2.560372

C -2.882305 -1.117227 -4.451943

H -3.465039 0.326711 -2.969190

C -1.142050 -2.665948 -3.816194

H -0.363347 -2.451336 -1.828596

C -2.049227 -2.190205 -4.768059

H -3.597064 -0.747070 -5.181394

H -0.480131 -3.494994 -4.045749

H -2.101331 -2.653384 -5.749559

C -4.816163 0.407175 0.957106

C -5.685856 -0.511434 0.312565

C -4.812589 0.476947 2.376462

C -6.449606 -1.396501 1.092643

C -5.579847 -0.441523 3.112472

C -6.383416 -1.381543 2.479882

H -7.126499 -2.082156 0.591878

H -5.537161 -0.407193 4.196827

H -6.975389 -2.080162 3.065067

C -4.102149 1.534470 3.162588

C -4.812562 2.203107 4.177435

C -2.755979 1.884530 2.971186

C -4.203615 3.174512 4.971658

H -5.862336 1.968727 4.330828

C -2.142739 2.850206 3.769306

H -2.178315 1.396940 2.197613

C -2.861430 3.500820 4.773662

H -4.781602 3.678345 5.742110

H -1.094740 3.087648 3.605953

H -2.382542 4.255888 5.391427

C -5.917603 -0.581267 -1.164891

C -5.939199 -1.833907 -1.805239

C -6.251587 0.549525 -1.930426

C -6.271296 -1.954365 -3.154170

H -5.691373 -2.724677 -1.235208

C -6.586075 0.430721 -3.280223

H -6.273458 1.527169 -1.464465

C -6.598265 -0.820625 -3.899460

H -6.272404 -2.935266 -3.621903

H -6.851996 1.320892 -3.844564

H -6.864984 -0.911406 -4.949082

**INT23B-2n**

P -1.218453 1.145323 0.194782

C -0.771021 1.405559 1.970472

C -0.097907 1.692766 4.685599

C -0.650530 0.276328 2.800124

C -0.542003 2.678027 2.518138

C -0.207771 2.818241 3.866788

C -0.323370 0.423486 4.149198

H -0.820839 -0.722500 2.406285

H -0.609572 3.562943 1.895851

H -0.031734 3.810396 4.274578

H -0.240858 -0.461476 4.773455

H 0.163239 1.804296 5.734681

C -1.064731 2.810388 -0.595013

C -0.787871 5.322825 -1.824919

C -1.932710 3.867350 -0.266475

C -0.067273 3.029730 -1.554222

C 0.072573 4.278385 -2.164087

C -1.792243 5.114284 -0.875845

H -2.727099 3.713310 0.457766

H 0.590028 2.209055 -1.822727

H 0.851967 4.431238 -2.905666

H -2.471615 5.920924 -0.613546

H -0.682567 6.293894 -2.301347

C -3.054829 0.885360 0.226463

C -3.831045 1.280490 -0.876396

C -3.679479 0.199272 1.279540

C -5.198523 1.014794 -0.915050

H -3.365167 1.797765 -1.708442

C -5.049463 -0.079231 1.230666

H -3.112184 -0.129757 2.142549

C -5.812873 0.330714 0.138062

H -5.783957 1.336469 -1.772465

H -5.510192 -0.618141 2.053412

H -6.877905 0.117847 0.104270

P 1.981179 -0.262298 -0.154857

C 2.462793 -1.275740 1.307768

C 3.694151 -1.119207 1.967179

C 1.577692 -2.274655 1.740427

C 4.028093 -1.949038 3.038293

H 4.389438 -0.348066 1.649005

C 1.913429 -3.101116 2.814434

H 0.611246 -2.405662 1.260168

C 3.138363 -2.940124 3.463659

H 4.982116 -1.818648 3.542693

H 1.201097 -3.854685 3.137573

H 3.400157 -3.580501 4.302096

C 2.856271 -1.069895 -1.574726

C 2.500296 -0.681023 -2.880453

C 3.095785 -1.281625 -3.991481

H 1.749761 0.093094 -3.023387

C 4.399653 -2.689006 -2.523657

C 4.046602 -2.288222 -3.814243

H 2.809762 -0.968431 -4.991950

H 5.135685 -3.475546 -2.380398

H 4.506486 -2.761882 -4.677279

C 2.899193 1.325258 0.104914

C 3.717148 1.888941 -0.886067

C 2.724066 2.022169 1.312949

C 4.338433 3.122596 -0.677608

H 3.883089 1.362528 -1.820373

C 3.348029 3.252525 1.518492

H 2.108242 1.601172 2.100653

C 4.154680 3.808716 0.523181

H 4.973426 3.540878 -1.454305

H 3.201935 3.774951 2.460064

H 4.640462 4.767201 0.685252

O -1.934392 -1.382335 -1.593461

C -2.087080 -1.757138 -2.955683

C -3.441572 -2.479960 -3.079807

C -2.082280 -0.505027 -3.851163

C -0.950683 -2.711670 -3.371581

H -3.456587 -3.378081 -2.449086

H -4.253611 -1.818601 -2.757538

H -3.638227 -2.793037 -4.112636

H -1.139709 0.042631 -3.730619

H -2.199716 -0.766654 -4.910102

H -2.900791 0.164643 -3.567924

H -1.060843 -3.051622 -4.409148

H 0.024189 -2.212973 -3.288093

H -0.939429 -3.593264 -2.720096

Rh -0.186229 -0.518634 -0.872883

Br -2.115116 -3.340936 1.913269

Li -2.386828 -2.147754 -0.028424

C 3.810194 -2.086780 -1.411089

H 4.091240 -2.410305 -0.414511

**3an**

P -1.018098 0.769379 -0.618975

C -0.021467 2.320638 -0.857308

C 1.476953 4.636189 -1.430806

C 0.414662 3.167819 0.175967

C 0.312094 2.654420 -2.179916

C 1.051544 3.804669 -2.466988

C 1.157914 4.314387 -0.108222

H 0.180950 2.925833 1.208244

H -0.006941 2.002255 -2.989149

H 1.299865 4.044924 -3.497452

H 1.487398 4.958556 0.703311

H 2.057607 5.528523 -1.649697

C -2.660777 1.432428 -0.070449

C -5.272388 2.272523 0.583344

C -3.617162 0.514227 0.403310

C -3.043221 2.773847 -0.222590

C -4.338563 3.188685 0.100242

C -4.904780 0.932505 0.734902

H -3.353149 -0.533539 0.517369

H -2.329423 3.502965 -0.592438

H -4.612780 4.233544 -0.023277

H -5.624606 0.207801 1.107206

H -6.278298 2.596887 0.836880

C -0.331221 0.091804 0.977782

C 0.625625 -0.951847 0.947346

C -0.729213 0.605392 2.224772

C 1.173583 -1.405381 2.159584

C -0.188343 0.131322 3.418568

H -1.484316 1.384693 2.259613

C 0.776589 -0.875402 3.384485

H 1.918115 -2.196025 2.130985

H -0.516322 0.549184 4.366876

H 1.213655 -1.252318 4.305403

C 1.081750 -1.629223 -0.315692

C 0.204323 -2.479130 -1.038462

C 0.666812 -3.122239 -2.196829

C 2.851306 -2.168925 -1.905341

C 1.977911 -2.967505 -2.634572

H -0.011482 -3.777077 -2.736056

H 3.878809 -2.042096 -2.234007

H 2.319672 -3.477850 -3.531098

C -1.199256 -2.775099 -0.620094

C -1.507894 -3.258550 0.662166

C -3.550809 -3.013581 -1.220403

C -2.815371 -3.612475 0.997567

H -0.717943 -3.370295 1.397115

C -3.842467 -3.493211 0.058186

H -4.341772 -2.908527 -1.958317

H -3.029034 -3.991313 1.993735

H -4.859714 -3.772122 0.320325

C 2.425121 -1.494714 -0.749807

C -2.243183 -2.659776 -1.554526

H -2.024349 -2.272206 -2.545188

C 3.446578 -0.676725 -0.025914

C 4.645363 -1.281034 0.389009

C 3.278617 0.696558 0.206169

C 5.639797 -0.539304 1.028181

H 4.789658 -2.344861 0.217923

C 4.274318 1.439555 0.839906

H 2.373185 1.191046 -0.126250

C 5.457341 0.825654 1.256689

H 6.556043 -1.029392 1.347677

H 4.122975 2.503741 1.000861

H 6.231133 1.405975 1.752491

**TS2A-1**

P -1.523934 -1.417893 -0.031282

C -2.058792 -1.481892 1.748719

C -2.736738 -1.397630 4.486833

C -1.062087 -1.409954 2.739074

C -3.400573 -1.492877 2.158157

C -3.735875 -1.448403 3.514397

C -1.396257 -1.379527 4.093073

H -0.015620 -1.378249 2.451300

H -4.190864 -1.534724 1.416031

H -4.783437 -1.454200 3.808023

H -0.603290 -1.333109 4.835219

H -2.999354 -1.365450 5.541919

C -3.110771 -1.778186 -0.942711

C -5.491869 -2.227146 -2.368279

C -3.788065 -3.004236 -0.836544

C -3.634082 -0.787578 -1.784268

C -4.818686 -1.011153 -2.491460

C -4.972945 -3.226149 -1.539194

H -3.382881 -3.791675 -0.206327

H -3.083554 0.143741 -1.890304

H -5.206696 -0.235712 -3.147548

H -5.486928 -4.180624 -1.446397

H -6.411565 -2.402380 -2.922510

C -0.658236 -3.050476 -0.261838

C -0.034448 -3.246653 -1.507611

C -0.581350 -4.068126 0.699671

C 0.645866 -4.433520 -1.778328

H -0.083916 -2.458640 -2.255400

C 0.107935 -5.254216 0.426935

H -1.051767 -3.936909 1.669395

C 0.722719 -5.440688 -0.811388

H 1.128193 -4.565768 -2.743693

H 0.162469 -6.031215 1.186793

H 1.261290 -6.362050 -1.022090

C -0.086534 1.091924 -3.231914

O -0.467865 -0.124644 -2.828257

O 0.124117 1.896447 -2.186954

Br -1.047244 2.085122 1.303548

O 0.056234 1.415191 -4.405571

C -2.010898 3.593635 0.577110

C -2.783214 4.365301 1.443608

H -2.841733 4.113794 2.497897

C -2.612243 4.979176 -1.276733

C -3.397664 5.767770 -0.432226

H -2.540108 5.215760 -2.335277

H -3.942465 6.620012 -0.831272

C 3.126337 1.830637 0.241736

C 4.454710 4.315299 0.245634

C 4.218222 2.060943 1.095669

C 2.710225 2.865480 -0.616424

C 3.377019 4.093146 -0.613789

C 4.872934 3.294187 1.101872

H 4.559008 1.277668 1.766520

H 1.867749 2.700995 -1.287652

H 3.043836 4.880329 -1.286166

H 5.714785 3.453594 1.773065

H 4.965765 5.275837 0.249456

C 2.612221 -0.634857 1.689980

C 2.878603 -2.027333 4.145700

C 2.489548 0.032211 2.927223

C 2.843534 -2.024697 1.720630

C 2.975551 -2.708536 2.930244

C 2.632048 -0.650633 4.134965

H 2.283567 1.099140 2.943071

H 2.919501 -2.576770 0.788997

H 3.154899 -3.781327 2.918782

H 2.548329 -0.103966 5.072025

H 2.986251 -2.560511 5.087240

C 3.248553 -0.659650 -1.125617

C 2.691014 -0.994664 -2.367640

C 4.602779 -0.953639 -0.888911

C 3.474351 -1.612294 -3.349277

H 1.647238 -0.767651 -2.571488

C 5.378051 -1.578827 -1.864352

H 5.054154 -0.691329 0.063991

C 4.813605 -1.909232 -3.100673

H 3.027890 -1.852945 -4.310851

H 6.424146 -1.801419 -1.663721

H 5.420061 -2.389990 -3.865517

P 2.177887 0.231221 0.113536

Rh -0.438281 0.408273 -0.808807

C -1.906132 3.877610 -0.782841

H -1.285514 3.258169 -1.431541

C -3.481662 5.459453 0.927547

H -4.092628 6.066143 1.592032

**INT2A-1**

P -1.317910 0.204706 0.051532

C -1.194837 2.058539 -0.006740

C -0.680395 4.831282 -0.180728

C -0.685403 2.771142 1.092386

C -1.414598 2.761943 -1.203290

C -1.165192 4.133236 -1.288471

C -0.436890 4.141971 1.009168

H -0.481344 2.246977 2.021545

H -1.786704 2.232660 -2.075809

H -1.349788 4.655532 -2.224694

H -0.047980 4.670982 1.876465

H -0.486278 5.899392 -0.246419

C -2.822987 -0.149135 -0.996621

C -5.049369 -0.872333 -2.557802

C -3.940393 0.696170 -1.086809

C -2.841574 -1.371791 -1.687794

C -3.951561 -1.729189 -2.457856

C -5.042022 0.342690 -1.868363

H -3.950440 1.642414 -0.552636

H -1.990331 -2.043001 -1.594411

H -3.953283 -2.681669 -2.982695

H -5.895422 1.014626 -1.935613

H -5.908082 -1.149662 -3.165780

C -2.042637 -0.110534 1.743405

C -1.732852 -1.341336 2.346283

C -2.908060 0.771334 2.410241

C -2.275008 -1.671549 3.591062

H -1.085439 -2.039650 1.819026

C -3.436733 0.443473 3.660459

H -3.165690 1.725938 1.959765

C -3.120264 -0.780240 4.254992

H -2.030255 -2.630359 4.042146

H -4.097922 1.143184 4.168013

H -3.533040 -1.037131 5.228436

Rh 0.491556 -1.021400 -0.441477

C 0.824398 -3.506590 -0.397159

O -0.335945 -2.863871 -0.151756

O 1.785428 -2.641560 -0.705479

Br 2.100872 0.903554 -0.993364

O 0.949245 -4.722818 -0.335877

C 3.805583 0.525453 -0.148740

C 4.114326 -0.783503 0.214874

C 5.359408 -1.017758 0.807135

H 3.404912 -1.591940 0.034376

C 5.924180 1.327831 0.648425

C 6.261992 0.026277 1.026062

H 5.618562 -2.032980 1.096912

H 6.618878 2.147525 0.817347

H 7.225458 -0.172918 1.489257

C 4.686383 1.586010 0.054338

H 4.409476 2.593754 -0.239889

**TS3A-1**

P -1.068860 0.055678 0.048472

C -1.402066 1.811940 -0.469981

C -1.712376 4.453374 -1.417677

C -1.453465 2.895167 0.420224

C -1.493071 2.081592 -1.847989

C -1.657162 3.383965 -2.316588

C -1.604254 4.204098 -0.049785

H -1.378903 2.718148 1.488503

H -1.429702 1.259409 -2.556140

H -1.731725 3.566119 -3.386248

H -1.640589 5.028155 0.659581

H -1.831787 5.470880 -1.782708

C -2.631950 -0.807197 -0.484988

C -4.910427 -2.265167 -1.256427

C -3.832028 -0.148423 -0.794018

C -2.583562 -2.210658 -0.570866

C -3.719851 -2.928802 -0.947467

C -4.963350 -0.872689 -1.181043

H -3.884674 0.935620 -0.746869

H -1.650611 -2.726143 -0.345454

H -3.668841 -4.013421 -1.007948

H -5.883894 -0.345919 -1.424773

H -5.790088 -2.829653 -1.558802

C -1.313663 0.121802 1.893856

C -0.213748 -0.188113 2.706254

C -2.538894 0.436108 2.504445

C -0.332204 -0.167325 4.098705

H 0.723537 -0.463929 2.230991

C -2.656504 0.459502 3.894605

H -3.408939 0.653848 1.890757

C -1.550554 0.159168 4.695336

H 0.528982 -0.415665 4.714127

H -3.612578 0.703355 4.353157

H -1.643671 0.170721 5.779264

C 1.560011 -3.350547 -0.252873

O 0.454132 -2.782149 0.271164

O 2.213390 -2.483199 -1.014046

Rh 0.809082 -0.990613 -0.635943

O 1.890486 -4.509467 -0.034408

Br 2.041483 0.851247 -2.016051

C 2.721811 0.772871 -0.133371

C 2.404555 1.860179 0.690041

C 4.462751 0.095040 1.373678

C 3.109709 2.029647 1.882869

H 1.608449 2.542119 0.411673

C 4.139830 1.153254 2.232800

H 5.252803 -0.602970 1.641099

H 2.849725 2.859190 2.537193

H 4.687460 1.292548 3.161477

C 3.776247 -0.096611 0.176027

H 3.978566 -0.948155 -0.463048

**INT3A-1**

P -0.924343 0.205028 0.021782

C -1.466135 -0.015113 1.775036

C -2.216791 -0.279017 4.468707

C -2.820240 -0.109514 2.132308

C -0.490767 -0.070218 2.781904

C -0.867631 -0.194611 4.120166

C -3.193326 -0.239551 3.471411

H -3.585778 -0.087199 1.362306

H 0.559876 -0.029953 2.516585

H -0.100582 -0.238986 4.889008

H -4.246325 -0.314879 3.732957

H -2.506510 -0.383862 5.511805

C -1.164428 2.015349 -0.281201

C -1.500752 4.741993 -0.877078

C -1.807751 2.869752 0.626617

C -0.676363 2.545925 -1.489672

C -0.851393 3.899531 -1.782641

C -1.973323 4.224844 0.329899

H -2.178950 2.481109 1.569236

H -0.126755 1.907415 -2.176243

H -0.464069 4.295977 -2.717801

H -2.470739 4.874802 1.046225

H -1.629085 5.797390 -1.106684

C -2.316578 -0.552329 -0.946059

C -2.346643 -1.951911 -1.082840

C -3.324056 0.210348 -1.557337

C -3.366728 -2.566136 -1.810590

H -1.566922 -2.552962 -0.619557

C -4.341007 -0.410570 -2.286687

H -3.315174 1.291789 -1.470386

C -4.365396 -1.799868 -2.415468

H -3.373063 -3.648819 -1.909162

H -5.111503 0.196044 -2.757073

H -5.154685 -2.281902 -2.987827

C 2.585916 -0.177366 -2.724668

O 2.719614 -1.228380 -1.919697

O 1.550881 0.582481 -2.303706

Rh 1.121891 -0.677398 -0.761496

O 3.266378 0.078977 -3.703267

Br 0.772922 -2.842012 0.643648

C 2.273863 0.393010 0.471701

C 3.026904 -0.264114 1.457544

C 2.415159 1.778236 0.316293

C 3.872110 0.467814 2.299075

H 2.943685 -1.338796 1.569103

C 3.262418 2.500719 1.163942

H 1.884958 2.290275 -0.477144

C 3.990200 1.852594 2.162873

H 4.444819 -0.056992 3.062069

H 3.356167 3.577156 1.030369

H 4.649838 2.416887 2.819058

**TS4A-1**

P 1.108340 0.106866 0.187532

C 1.980478 1.684152 -0.192103

C 3.161148 1.712631 -0.950605

C 1.414188 2.902541 0.224000

C 3.773030 2.927099 -1.267633

H 3.606734 0.783444 -1.290885

C 2.032424 4.112853 -0.088648

H 0.485825 2.903738 0.784939

C 3.214541 4.129868 -0.832990

H 4.688637 2.929729 -1.854230

H 1.581879 5.044694 0.243505

H 3.692643 5.075237 -1.078262

C 0.669385 0.163769 1.966905

C -0.533470 -0.561896 2.146492

C 1.226229 0.933131 2.993664

C -1.164642 -0.484015 3.397754

H -0.794582 -1.714966 1.504422

C 0.594188 0.959486 4.239658

H 2.122485 1.525111 2.822724

C -0.598657 0.255156 4.439400

H -2.095622 -1.023169 3.560133

H 1.024969 1.540224 5.052621

H -1.088645 0.292237 5.410456

C 2.390118 -1.191028 -0.044797

C 3.493850 -1.307104 0.815446

C 2.240107 -2.098228 -1.103761

C 4.440713 -2.310451 0.613538

H 3.606794 -0.618923 1.649056

C 3.194772 -3.099021 -1.303758

H 1.371811 -2.023548 -1.754668

C 4.292501 -3.206734 -0.449307

H 5.289007 -2.398288 1.288306

H 3.068316 -3.801936 -2.122941

H 5.028323 -3.992715 -0.603192

C -2.401012 -2.774442 0.588348

O -2.860252 -1.617538 0.135058

O -1.039111 -2.753985 0.754100

Rh -1.099144 -0.611169 -0.180310

Br -1.091003 -0.989223 -2.658726

C -1.895304 1.231059 -0.411409

C -2.996931 1.535002 0.405595

C -1.459316 2.204250 -1.323360

C -3.619878 2.785476 0.335824

H -3.384448 0.779198 1.082400

C -2.084405 3.454456 -1.393523

H -0.637455 1.981999 -1.996434

C -3.163778 3.755087 -0.560465

H -4.471246 2.996659 0.981409

H -1.726110 4.192308 -2.110258

H -3.649868 4.727415 -0.615706

O -3.088088 -3.747806 0.853018

**INT4A-1**

P -1.114295 -0.069969 0.339567

C -2.198033 -1.522330 0.025319

C -3.783880 -3.783997 -0.443595

C -3.438917 -1.665826 0.668714

C -1.757930 -2.517628 -0.859387

C -2.555837 -3.642549 -1.090082

C -4.226813 -2.792268 0.437497

H -3.792756 -0.892806 1.346550

H -0.807202 -2.396721 -1.374866

H -2.209853 -4.407884 -1.780132

H -5.184560 -2.896865 0.942361

H -4.399014 -4.662741 -0.625071

C -0.576218 -0.030124 2.078800

C 1.057335 -0.110124 4.293004

C -1.169927 0.038247 3.342228

C 0.817137 -0.143330 1.887833

C 1.637878 -0.181931 3.022358

C -0.331560 0.001399 4.459844

H -2.248754 0.120299 3.461866

H 2.715832 -0.278973 2.916321

H -0.755226 0.056447 5.460431

H 1.694247 -0.144165 5.176237

C -2.207070 1.366128 -0.011500

C -3.002815 1.372435 -1.168733

C -2.208532 2.494709 0.821096

C -3.794431 2.478966 -1.476138

H -3.000763 0.510974 -1.830477

C -3.004657 3.598970 0.512294

H -1.584545 2.508294 1.709305

C -3.800420 3.594050 -0.634599

H -4.404466 2.470375 -2.376161

H -2.997042 4.466321 1.167925

H -4.417574 4.456482 -0.875332

Br 1.050986 -0.823853 -2.739799

C 1.484863 1.643790 -0.314372

C 0.817095 2.465832 -1.235284

C 2.540039 2.204426 0.423682

C 1.186310 3.804050 -1.405142

H 0.024729 2.051215 -1.848786

C 2.910898 3.542112 0.252089

H 3.093729 1.585301 1.123056

C 2.232122 4.352684 -0.660054

H 0.654855 4.416393 -2.132423

H 3.736394 3.948717 0.835235

H 2.518333 5.394562 -0.792964

Rh 1.129010 -0.329524 -0.107506

O 3.186401 -0.985654 0.131146

C 2.863375 -2.207535 0.232298

O 1.682653 -2.639102 0.238559

O 3.893007 -3.098857 0.354449

H 3.451540 -3.964287 0.391917
